# Structural characteristics and contractual terms of specialist palliative homecare in Germany

**DOI:** 10.1186/s12904-023-01274-6

**Published:** 2023-10-31

**Authors:** Maximiliane Jansky, Lia Heyl, Michaela Hach, Steven Kranz, Thomas Lehmann, Antje Freytag, Ulrich Wedding, Winfried Meißner, Sabine H. Krauss, Werner Schneider, Friedemann Nauck, for the SAVOIR Study Group, Anna Bauer, Bianka Ditscheid, Cornelia Eichhorn, Elke Gaser, Ulrike Hammer, Aicko Helbig, Beata Hennig, Michelle Kaufmann, Markus Krause, Isabel Kruschel, Helmut L’hoest, Srikanth Maddela, Ursula Marschall, Martial Mboulla, Heiner Melching, Florian Mühler, Cornelia Nageler, Sara Parhizkari, Judith Rothaug, Joachim Saam, Sven Schulz, Kathleen Stichling, Horst C. Vollmar, Julia von Hayek

**Affiliations:** 1 Department of Palliative Medicine, University Medical Center, Goettingen, Germany; 2 German Association for Palliative Medicine (DGP), Berlin, Germany; 3 Bundesarbeitsgemeinschaft SAPV (BAG), Wiesbaden, Germany; 4 Center for Clinical Studies, Jena University Hospital, Jena, Germany; 5 Institute of General Practice and Family Medicine, Jena University Hospital, Jena, Germany; 6 Department of Palliative Care, Jena University Hospital, Jena, Germany; 7 Center for Interdisciplinary Health Research, University of Augsburg, Augsburg, Germany

**Keywords:** Palliative homecare, Health services research, Specialist palliative care, Health care regulations

## Abstract

**Background:**

Multi-professional specialist palliative homecare (SPHC) teams care for palliative patients with complex symptoms. In Germany, the SPHC directive regulates care provision, but model contracts for each federal state are heterogeneous regarding staff requirements, cooperation with other healthcare providers, and financial reimbursement. The structural characteristics of SPHC teams also vary.

**Aim:**

We provide a structured overview of the existing model contracts, as well as a nationwide assessment of SPHC teams and their structural characteristics. Furthermore, we explore whether these characteristics serve to find specifc patterns of SPHC team models, based on empirical data.

**Methods:**

This study is part of the multi-methods research project “SAVOIR”, funded by the German Innovations Fund. Most model contracts are publicly available.

Structural characteristics (e.g. number, professions, and affiliations of team members, and external cooperation) were assessed via an online database (“*Wegweiser Hospiz- und Palliativversorgung*”) based on voluntary information obtained from SPHC teams. All the data were updated by phone during the assessment process.

Data were descriptively analysed regarding staff, cooperation requirements, and reimbursement schemes, while latent class analysis (LCA) was used to identify structural team models.

**Results:**

Model contracts have heterogeneous contract partners and terms related to staff requirements (number and qualifications) and cooperation with other services. Fourteen reimbursement schemes were available, all combining different payment models. Of the 283 SPHC teams, 196 provided structural characteristics. Teams reported between one and 298 members (mean: 30.3, median: 18), mainly nurses and physicians, while 37.8% had a psychosocial professional as a team member. Most teams were composed of nurses and physicians employed in different settings; for example, staff was employed by the team, in private practices/nursing services, or in hospitals. Latent class analysis identified four structural team models, based on the team size, team members’ affiliation, and care organisation.

**Conclusion:**

Both the contractual terms and teams’ structural characteristics vary substantially, and this must be considered when analysing patient data from SPHC. The identified patterns of team models can form a starting point from which to analyse different forms of care provision and their impact on care quality.

**Supplementary Information:**

The online version contains supplementary material available at 10.1186/s12904-023-01274-6.

## Introduction

Palliative care at home can be provided both on a general and a specialist care level [1]. Primary palliative care (PPC)—as provided by general practitioners (GPs), mobile nursing services, nursing homes, and hospitals—is sufficient for most patients with a life-limiting disease [2, 3]. For patients with complex needs who require the support of specialists in palliative medicine and nursing care, specialist palliative homecare (SPHC) teams provide multi-professional care that aims to improve their quality of life and help patients to stay at home, in nursing homes and facilities for people with disabilities [1]. In Germany, in 2019 16.0% of patients in their last year of life received SPHC and 23.9% received PPC [4]. The European Association for Palliative Care (EAPC) describes palliative homecare teams as multi-professional teams offering a graded approach that ranges from an advisory function to full, holistic, and round-the-clock palliative care. Regarding staff requirements, the EAPC recommends that four to five full-time professionals deliver this care, including physicians, nurses, social workers, and administrative staff. Staff members should have a high level of professional palliative care training. SHPC teams should guarantee patients’ easy access to other disciplines and professionals (e.g., physiotherapists, psychologists, and spiritual care workers, among others) through their network [1]. A review from 2018 identified the key components of quality palliative care at home as integrated teamwork; the management of pain and physical symptoms; holistic care; caring, compassionate, and skilled providers; timely and responsive care; and patient and family preparedness [5].

### SPHC in Germany – the SPHC directive

In Germany, SPHC was introduced in 2007 by §132d and §37b of the German Social Code Book V [6]. Statutory and private health insurance funds fully cover these services. The SPHC directive of the Federal Joint Committee regulates SPHC conditions [7]. Teams providing SPHC must include at least physicians and nurses who are professionally trained and experienced in palliative care ([7], §5; [8]). Whereas cooperation with volunteer hospice services is mandatory, cooperation with other professionals such as social workers and psychologists is not ([7], §6). The SPHC services include consultation, coordination of care, partial and full care, as well as the provision of a 24/7 call service ([7], §5). Patients are eligible if they suffer from a severe, incurable, progressive, and life-limiting disease with a life expectancy of months, weeks, or days ([7], §3). Moreover, they have to experience a complex symptom burden for which primary palliative care is not sufficient ([7], §4). Usually, primary care or hospital physicians make the decision to involve SPHC ([7], §7).

### SPHC model contracts and structures

The SPHC directive provides a generally worded framework to enable single or groups of health insurance funds and care providers/representative organisations to make agreements based on regional specifications and the existing healthcare structures (selective contracting) [7]. Contracts encompass heterogeneous terms for care provision and services, staff requirements, cooperation with other healthcare providers, quality assurance requirements, and financial reimbursement [5].

SPHC reimbursement schemes usually combine different payment methods, such as case-based lump sums (one payment per case), daily fees, or service fees (such as for home visits). They may differ between federal states, health insurance funds, and even teams from the same state [9]. As they determine the revenues obtained by a team to cover the cost of care provision, the reimbursement schemes may influence the type and quality of care [10, 11].

To date, only regionally limited descriptive analyses of structural and contractual characteristics in SPHC have been undertaken [12–15]. The structural characteristics of SPHC teams are heterogeneous [9, 13, 16, 17], varying in contract terms and many other factors, such as the pre-existing regional healthcare structures or networks, the teams’ ownership (e.g., hospital-based vs. independent), and their location (e.g., urban vs. rural setting).

We aim to provide an overview of these characteristics and their distribution across the various SPHC models in Germany, which may help to improve quality assessment and increase comparability. To reduce complexity and facilitate future analyses, a typology of different “team models” based on their structural characteristics may provide a simplified yet empirically based approach that encompasses the complex heterogeneous structures of SPHC.

## Aims and research questions

We aim to answer the following questions:

- What preconditions do model contracts set for the provision and organisation of SPHC in Germany?
- What are the structural characteristics of SPHC teams in Germany?
- Can these characteristics serve to create a structural typology of SPHC team models, based on empirical data?

In the Discussion section, we assess the extent to which the model contracts and team structures adhere to the requirements set by the nationwide SPHC directive and the professional recommendations and standards set by the EAPC. Furthermore, we elaborate on how the model contract specifications and empirically identified types of SPHC team models may influence care provision, based on the international literature.

## Method

This study is part of the multi-methods research project “SAVOIR—an evaluation of specialist palliative homecare (SAPV) in Germany: outcomes, interactions, regional differences” [16], which has been funded by the German Innovations Fund (launched by the Federal Joint Committee) to evaluate the execution of the SPHC directive and present recommendations for its revision (01VSF16005). Ethics approval was sought from the research ethics committee at the University Medical Center Göttingen (No. 31/8/17).

### Data assessment

#### Model contracts

In Germany, 17 model contracts for SPHC exist (see Table 1). Most of the contracts are publicly available (all publicly available contracts are provided in Additional file 1, available only in German). The model contracts were analysed using the following categories:

- Staff requirements
  - ◦ Team size, team members’ professions and qualifications
  - ◦ Activity emphasis of team members
- Cooperation requirements
- Reimbursement
  - ◦ Type of schemes (according to a generic overview of the reimbursement schemes by Amelung [18], we differentiated between case-based lump sums, service fees, and daily/weekly rates as the reimbursement schemes applied in terms of SPHC)
  - ◦ Excluded services
  - ◦ Other aspects of reimbursement

**Table 1:** Characteristics of model contracts

	**Version**	**Contract partners**	**Staff requirements**	**Mandatory cooperation**	**24-h service**
**Bavaria** [19]	09/2010	HIF^a^, SP^b^	Four permanent posts; qualifications as defined by directive^d^	Not specified	Physician
**Baden-Württemberg** [20]	01/2010	HIF^a^, SP^b^	Qualifications as defined by directive; newly established teams may employ staff for three years while they are in training	Not specified	Nurse and physician
**Berlin, physicians** [21]	12/2016	AIHP^c^, Home Care Society, Nursing Association, HIF^a^	Qualifications as defined by directive	Specialist mobile palliative care nursing service	Physician
**Berlin, nursing care** [21]	12/2016	AIHP^c^, Home Care Society, nursing association, HIF^a^	Team manager and three full-time nurses; qualifications as defined by directive	Specialist palliative care physician	Nurse
**Brandenburg** [22]	04/2009	HIF^a^, SP^b^	Physicians, nurses, coordinating nurse; physician qualification: advanced training in palliative medicine, (coordinating) nurse: qualifications as defined by directive	Not specified	Nurse or physician
**Bremen** [23]	08/2009	HIF^a^, SP^b^	Three physicians, four nurses; qualifications as defined by directive	Not specified	Nurse and physician
**Hamburg** [24]	02/2010	HIF^a^, SP^b^	Three physicians, four nurses; qualifications as defined by directive Four FTE nurses from a nursing service; at least two full-time nurses	Pharmacy	Nurse and physician
**Hesse**^**e**^ [25]	05/2009	HIF^a^, SP^b^	Qualifications as defined by directive. Possibility to employ staff while they are in training	Not specified	Nurse and physician
**Hesse AOK health insurance fund**	**Not available**				
**Mecklenburg-Western Pomerania** [26]	unclear	HIF^a^, SP^b^	Qualifications as defined by directive	Not specified	Nurse or physician
**Lower Saxony** [27]	01/2010	HIF^a^, SP^b^	Qualifications as defined by directive, cooperating physicians: basic training	Pharmacy	Nurse or physician
**North Rhine** [28]	06/2009	HIF^a^, AHIP^c^	Three physicians, four nurses. Qualifications as defined by directive; exclusive or main activity in SPHC	Pharmacy	Nurse and physician
**Rhineland Palatinate** [29]	**Contract was canceled at time of assessment**				
**Saarland**	**Contract not publicly available**				
**Saxony Anhalt** [30]	03/2010	AHIP^c^, SP^b^	Not available		
**Saxony/Thuringia** [31]		HIF^a^, SP^b^	At least five physicians with activity emphasis in palliative medicine, two of whom work at least 19 h/week exclusively in SPHC Four FTE nurses employed by SPHC team; at least two full-time nurses; the other FTE can be split, with each working at least 19h/week Qualifications as defined by directive	Palliative care unit	Nurse and physician
**Schleswig–Holstein** [32]	2009	HIF^a^, SP^b^	Qualifications as defined by directive		Nurse or physician
**Westphalia** [33]	04/2009	AHIP^c^, SP^b^	At least three physicians; at least one coordinating nurse; qualifications as defined by directive	General and specialist ambulatory physicians	Physician

^a^*HIF* Health insurance fund; ^b^*SP* service provider (SPHC team); ^c^*AHIP* Association of Statutory Health Insurance Physicians;^d^ the SHPC directive demands that physicians have certified training in palliative medicine and have cared for at least 75 palliative care patients during the previous three years. Nurses must have certified training in palliative care and at least two years of practical experience of caring for palliative patients for at least six months in a specialised facility. All other professionals in the team should have the respective training in palliative care or practical experience with palliative care patients.^e^ Hesse has three model contracts for different groups of health insurance funds, which differ only in their reimbursement schemes; therefore, they have been combined in this table

#### Structural characteristics of SPHC teams

To assess the structural characteristics, we used an online database provided by the German Association for Palliative Medicine (DGP), which is based on voluntary information obtained from SPHC teams (“*Wegweiser Hospiz- und Palliativversorgung*”). At the time of the assessment (July 2017 to January 2018), it contained data of 270 teams providing SPHC for adult patients. Due to regulatory differences, teams from the Westphalia region were separately assessed. We added 13 teams from Westphalia to the population; these had been missing in the database [34].

The *Wegweiser* database includes the teams’ structural information. Table 1 in Additional file 2 lists in detail the variables used for our analysis. The database already contained some items (e.g., year of establishment, total full-time equivalents (FTE) of staff members, and patient data), while other items were added for the study to provide more detailed information (e.g. different professional groups, affiliation of staff members, additional cooperation partners). The list of items was discussed by the multi-professional research team (SAVOIR study group, see acknowledgements) through an online discussion. All the data were updated during the assessment process. The DGP first emailed all the teams in the *Wegweiser* in July 2017 and asked them to update their information. Afterward, LH assessed all data by phone between July 2017 and January 2018.

In brief, we analyse and present data on the 1) management of SPHC teams, 2) team size, team members’ professions and qualifications, 3) activity emphasis of team members, 4) institutional affiliation of team members, 5) organisation of coordination and patient care, 6) cooperation with primary palliative care and other professionals, and 7) patients under care and 8) reimbursement schemes.

### Analysis

First, we descriptively analysed the model contracts and teams’ structural characteristics using SPSS 26 [35]. Latent class analysis (LCA) was used to identify team models based on the team size, institutional affiliation of team members, the presence of a psychosocial professional in the team, and the organisation of coordination and patient care (see the italicised variables in Additional file 2: Table 1). The variables for the LCA were dichotomised. Latent class models with different numbers of classes were fitted, and the model with the best fit according to the Bayesian Information Criterion (BIC) was selected [36]. Conditional item response probabilities were calculated for the selected model to evaluate the structure of each class.

Additionally, for each participating team, we estimated the posterior probability of each class for the selected model according to the team’s characteristics. In the next step, we assigned each team to the class with the highest posterior probability. LCA was performed in R 3.6.2 [37] using the poLCA package [38]. For statistical codes for LCA see Additional file 3.

## Results

We first report the descriptive results concerning the structural characteristics of SPHC teams. For each characteristic, we initially assess the respective content from the model contracts. At the end of the Results section, we report the identified structural typology of the team models.

### Sample

#### Model contracts

Table 1 shows the characteristics of the model contracts. Saxony and Thuringia have the same model contract [31]. In Hesse, three different health insurance fund groups have different contracts, of which only one is publicly available [25]. Berlin has different contracts for nurses and physicians [21]. The contract for Rhineland Palatinate had been canceled by insurance funds at the time of the assessment [29], and the contract of Saarland is not publicly available, so those contracts were not included in the analysis. The reimbursement scheme was only (at least partially) available for 13 of the 17 contracts (see Table 2).

**Table 2:** Reimbursement schemes

		BW	BV	BE P	BE N	BB	HB	HH	HE 1^f^	HE 2	MV	LS	NR	RP	SL	ST ^e^	SX/ TH	SH	WE	Sum
	Reimbursement scheme publicly available	x	-	x	x	x	-	-	x	x	x	x	x	contract cancelled	-	x	x	-	x	12
	Differentiated care levels	x	Individually negotiated	x		x			x	x	x		x				x			8
**Full care**	Case fee **w/o** performance day^a^	x		x		x					x	x	x			x	Individually negotiated		x	8
	Case fee **w/** performance day^b^				x				x	x										3
	Weekly rate				x	x										x				2
	Daily rate								x^c^		x	x	x						x^c^	5
	Daily rate w/ visit	x																		1
	Fee for service	x		x	x							x				x				5
	Nursing care (§37 SGB V) excluded if SPHC				x	x					x	x								4
**Partial care**	Case fee **w/o** performance day^a^	x		x		x					x	x	x			x	x		x	9
	Case fee **w/** performance day^b^				x				x	x							x			4
	Weekly rate				x	x										x				3
	Daily rate								x^c^		x	x								3
	Daily rate w/ visit	x																		1
	Fee for service	x		x	x						x	x	x			x	x^d^		x	9
	Nursing care (§37 SGB V) excluded if SPHC					x														1
**Coordination**	Case fee **w/o** performance day^a^			x		x					x	x	x			x	x		x	8
	Case fee **w/** performance day^b^				x				x	x							x			4
	Weekly rate				x											x				2
	Daily rate								x		x	x								3
	Daily rate w/ visit																			0
	Fee for service	x		x	x							x	x			x	x^d^		x	8
**Consultation**	Case fee **w/o** performance day^a^			x		x					x	x				x	x		x	7
	Case fee **w/** performance day^b^				x												x			2
	Weekly rate				x											x				2
	Daily rate										x	x								2
	Daily rate w/ visit																			0
	Fee for service	x		x	x				x	x		x	x			x	x^d^		x	10
	Transportation expenses										x								x	2
	Fees for GPs and other physicians					x										x			x	3

*BW* Baden-Württemberg, *BV* Bavaria, *BE P* Berlin, physicians, *BE N* Berlin, nursing care, *BB* Brandenburg, *HB* Bremen, *HH* Hamburg, *HE 1* Hesse (two contracts), *HE 2* Hesse AOK health insurance fund, *MV* Mecklenburg-Western Pomerania, *LS* Lower Saxony, *NR* North Rhine, *RP* Rhineland Palatinate, *SL* Saarland, *ST* Saxony Anhalt, *SX/T* Saxony/Thuringia, *SH* Schleswig–Holstein, *WE* Westphalia

^a^ Case-based lump sum is paid for days with and without patient-related contact ^b^ Case-based lump sum is only paid for days with patient-related contact

^c^if any kind of service is provided ^d^number of visits with cut-off ^e^only the regulations for physicians are publicly available ^f^Hesse has three model contracts for different groups of health insurance funds. Two of these model contracts share the same reimbursement scheme

Most versions of the model contracts date from 2009 and 2010, only Berlin contracts were updated (in 2016).

All the model contracts except for that of Westphalia are based on §132d and §37b of the German Social Code Book V. Westphalia operates with a different palliative homecare model based on general practitioners and palliative consulting teams that offer consultation and, if necessary, coordinate primary and specialist palliative care [33]. In most federal states, SPHC teams enter into individual contracts based on the respective model contracts. In Westphalia, Mecklenburg-Western Pomerania [26], Berlin [21], and North Rhine [28], health insurers and the Regional Association of Statutory Health Insurance Physicians are the main contract partners.

#### SPHC teams

A total of 196 SPHC teams from all regions provided valid datasets (Table 3). Most SPHC teams were established between 2008 and 2015, after the directive had been passed (see Table 4). Some may have existed previously in a different form but did not state this in our survey.

**Table 3:** Number of valid datasets and SPHC teams, according to *Wegweiser Hospiz- und Palliativversorgung* (November 2017)

Regions	Datasets in sample	Number of Teams	Response rate (%)
Baden-Württemberg	28	37	75.7
Bavaria	31	43	72.1
Berlin	4	10	40.0
Brandenburg	3	8	37.5
Bremen	2	2	100.0
Hamburg	6	8	75.0
Hesse	22	22	100.0
Mecklenburg-Western Pomerania	5	10	50.0
Lower Saxony	38	47	80.9
North Rhine	7	13	53.8
Rhineland Palatinate	6	7	85.7
Saarland	2	4	50.0
Saxony	10	12	83.3
Saxony Anhalt	5	8	62.5
Schleswig Holstein	8	9	88.9
Thuringia	7	9	77.8
Westphalia	11	34	32.4
**Total**	**196**	**283**	**69.3**

**Table 4:** Organisational and medical management of SPHC teams, psychosocial and other professions; other organisational characteristics (*n* = 196)

Item	Categories	Number	%
Year of establishment	Before 2008	14	7.1
	2008 to 2015	146	74.5
	After 2015	10	5.1
	Missing	26	13.3
Organisational management	Physician	49	25.0
	Nurse	68	34.7
	Others	27	13.8
	Physician and nurse	33	16.8
	Physician and others	3	1.5
	Nurses and others	7	3.6
	Physicians, nurses, and others	7	3.6
	Missing	2	1.0
Medical management (multiple answers possible)	General physician	41	20.9
	Hospital physician	34	17.3
	Medical specialist	51	26.0
	SPHC physician	103	52.6
	No medical management	13	6.6
	Missing	1	0.5
Psychosocial professions in team	Psychologist	30	15.3
	Religious worker	45	23.0
	Social worker	35	17.9
	At least one psychosocial profession	74	37.8
Other professions in team	Coordination	40	20.4
	Administration	84	42.9
	Office worker	12	6.1
	Other professions	13	6.6
Organisation of coordination and patient care	Centralised coordination and patient care	119	60.7
	Centralised coordination, decentralised patient care	57	29.1
	Decentralised coordination and patient care	9	4.6
	Physicians and nurses work separately	6	3.1
	Other	5	2.6

Different sources can be used to determine the current number of SPHC teams in Germany [39–41], resulting in between 270 and 326 teams (as of November 2018) [42, 43]. None of these sources is completely reliable, so our response rate was based on the data available in the *Wegweiser* database (270 teams + 13 teams from Westphalia*)* at the beginning of the assessment*.* The response rate across the different federal states varied from 32.4% to 100% (see Table 3).

### Management of SPHC teams

Model contracts give no specifications about team management.

Nurses were part of the organisational management in more than half of the teams, with one-third being managed by nurses alone (see Table 4). One-quarter were led exclusively by physicians. SPHC physicians held a medical director position in more than half of the teams, while 6.6% of the teams had no medical director.

### Team size, team members’ professions and qualifications

Seven of the 17 contracts regulate the minimum number of team members (see Table 1). All contracts demand that staff members must be certified in specialist palliative care or palliative medicine as defined by the directive. In Lower Saxony, cooperating physicians can provide care if they have basic training in palliative medicine [27] (see Table 1).

The teams had a mean number of 30.3 staff members and 10.9 full-time equivalents (FTE), with team sizes varying considerably between 1 and 298 staff members. Many teams did not have detailed knowledge of the proportion of time contributed by their members, especially if physicians from private practices or nurses from mobile nursing services provided SPHC (see Table 5).

**Table 5:** Full time equivalents (FTE) and number of staff members in total, physicians and nurses (*n* = 196)

		**Average**	**SD**	**Median**	**Range**	**Missing (%)**
Staff members	Full time equivalents (FTE)	10.9	8.7	9	1–48	103
	Number	30.3	35.0	18	1–298	3
Physicians	FTE	3.4	3.2	2.5	0–19	94
	Number	10.1	9.3	7	1 to 52	6
Nurses	FTE	6.7	10.8	4.5	0–113	38
	Number	19.2	28.3	9	1–239	13

On average, the teams consisted of 10.1 physicians, with the number ranging between 1 and 52, and 19.2 nurses, with a range of 1 to 239 nurses (see Table 5). 37.8% had at least one psychosocial professional as a team member. More than two-thirds of the teams (62.8%) had other employees like coordinators, administrative and office workers, or other therapists specified in free text entries (see Table 4).

In most teams, all the physicians and nurses were certified in palliative care (80.6/79.6%), only a minor proportion had no certified staff members at the time of the study: Two teams had no certified physicians and one team had no certified nurses (see Table 6).

**Table 6:** Qualifications, activity emphasis, and institutional affiliations of physicians and nurses in SPHC (*n* = 196)

		**Physicians**		**Nurses**	
**Item**	**Categories**	Number of teams	%	Number of teams	%
% of staff members trained in palliative care	100%	158	80.6	156	79.6
	≥ 50%	20	10.2	19	9.7
	< 50%	4	2.0	0	0.0
	0	2	1.0	1	0.5
	Missing	12	6.1	20	10.2
Staff members performing less than 50% of their activity in palliative care	100% of staff members	53	27.0	22	11.2
	99–70% of staff members	49	25.0	16	8.2
	69–50% of staff members	21	10.7	7	3.6
	49–30% of staff members	5	2.6	9	4.6
	29–0.1% of staff members	5	2.6	20	10.2
	0% of staff members	26	13.3	81	41.3
	Missing	37	18.9	41	20.9
Physicians/nurses with institutional affiliation in team (yes/no)	Hospital	86	43.9	39	19.9
	Private practice/nursing care service	132	67.3	64	32.7
	Team	79	40.3	125	63.8
	Other structures	36	18.4	21	10.7
	More than one structure	109	55.6	49	27
	Missing	7	4.1	13	6.6

### Activity emphasis of team members

Some contracts determine a minimum weekly working time (or a minimum number of full-time employees in the cases of Hamburg [24] and Saxony/Thuringia [31]) and activity emphasis (North Rhine [28], Saxony/Thuringia) for team members (see Table 1).

In some teams, all the physicians (27.0% of the teams) or nurses (11.2% of the teams) worked less than 50% of their time in SPHC. In 62.8% and 23.0% of the teams, more than half of the physicians and nurses, respectively, worked less than 50% in SPHC (see Table 6).

### Institutional affiliation of team members

SPHC teams can employ team members directly, but they can also incorporate physicians and nurses from hospitals or private practices/nursing services.

Model contracts like Bavaria [19] and Saxony/Thuringia determine [31] minimum permanent positions. The model contract of Hamburg [24] determines that at least four FTE nurses must be employed by a nursing service (see Table 1).

Only 40.8% of the teams worked with physicians from only one institutional setting, while 66.1% contained nurses with a single affiliation. Physicians from private practices provided SPHC in 67.3% of the teams, while about 40% of the teams worked with physicians employed by hospitals or directly by the team (Table 6, for more detailed information see Additional file 2: Table 2). Of all the teams, 63.8% employed at least some nurses and 39.8% directly employed all the nurses in the team (see Additional file 2: Table 2).

### Organisation of coordination and patient care

The model contracts indicate that on-call services must be provided by nurses and physicians (six contracts), by nurses or physicians (four contracts), by nurses (one contract), or by physicians (three contracts) (see Table 1). Most teams (60.7%) coordinated patient care centrally; those with a decentralised model used, for example, regional satellite teams (see Table 4).

### Cooperation with primary palliative care and other professionals

Cooperation with volunteer hospice services is mandatory, as defined in the directive. Other mandatory forms of cooperation are specified in Table 1.

**Fig. 1:**
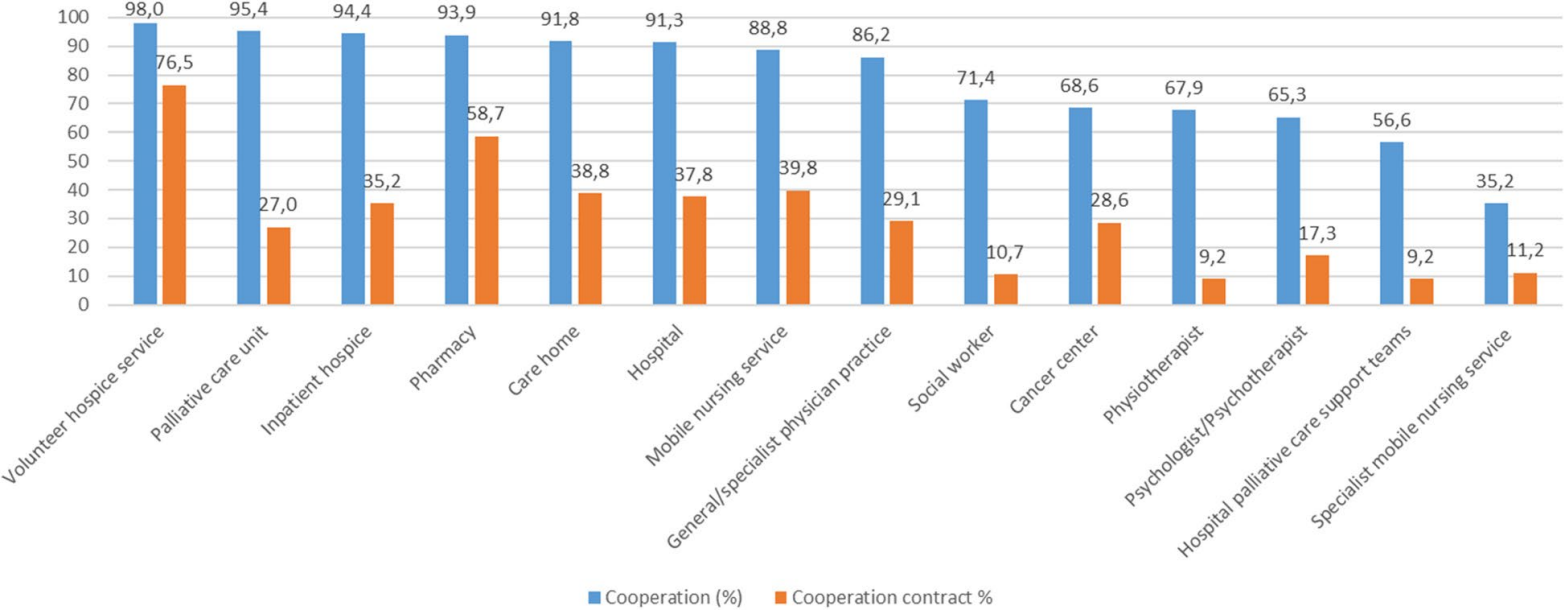
Cooperation and cooperation contracts in specialist palliative homecare teams (*n* = 196)

Almost all teams (98%) reported to cooperate with volunteer hospice services. Cooperation with other palliative care providers was also frequently reported (see Fig. 1). Cooperation with non-palliative care professionals or specialists was less frequent. However, formal cooperation contracts were less prevalent (see Fig. 1).

### Patients under care (in 2016)

For the analysis of patient data, all teams established after 2015 (*n* = 10) were excluded. Patient numbers should be understood as estimates since some teams could not give exact numbers. On average, the teams cared for 394 patients in 2016, with a considerable range of 40 to 1,712. Almost 80% of those patients died while receiving SPHC (see Table 7). On average, more than 80% of the teams’ patients had an oncological diagnosis. The mean length of care was 33.2 days. On average, the teams reported to drive 21.5 km (up to 60 km) and for 26.3 min (up to 60 min) for a patient visit (see Table 7).

**Table 7:** Patient care in 2016, *n* = 186; teams with a founding year ≥ 2016 were excluded

	**Mean**	**SD**	**Median**	**Range**		**Missing (n)**
Patients cared for in 2016	394.08	266.83	332.00	40	1712	17
% of above who died	78.24	14.03	79.38	8.56	100	32
% of above with oncological diseases	82.05	11.13	84.61	47.49	98.92	54
Minimum length of stay (days)	1.02	0.15	---	1.00	2.00	11
Mean length of stay (days)	33.17	16.66	30.00	9.00	90.00	34
Maximum length of stay (days)	295.62	144.96	---	31.00	1040.00	24
Average distance to patient (kilometers)	21.46	11.49	20.00	3	60	42
Average driving time to patient (minutes)	26.28	10.25	25.00	1	60	45

### Reimbursement schemes

Twelve out of the 18 reimbursement schemes are publicly available. Table 2 shows the attributes of these schemes [18]. Bavaria has a model contract, but teams negotiate reimbursement individually [19]. Thuringia and Saxonia have model reimbursement schemes, but the fees are individually negotiated [25, 31]. Hesse has two different reimbursement schemes from three model contracts.

All the reimbursement schemes combine different payment models. Case-based lump sums are used in up to nine schemes (depending on the SPHC care level). In some schemes, rates are paid for a certain number of days on the condition that some form of (specified) service is provided (a case-based lump sum per performance day; e.g. a sum is payed for the first 10 days of care, but days are only counted if service is provided). Some schemes are based on weekly (up to three weeks) or daily (up to eight days) rates, but daily rates may only apply when patients are visited. Up to 10 schemes contain fees for services, mainly home visits, but in some cases also phone calls or other services. Rates may be differentiated by the time of service delivery, for instance, during or outside office hours.

Reimbursement schemes in Berlin [21], Brandenburg [22], and Lower Saxony [27] explicitly exclude the funding of mobile nursing services for the provision of treatment care (§37 SGB V) parallel to SPHC for patients in full respectively also partial (Brandenburg) care, meaning that SPHC teams have to cover specialist palliative care nursing services as well as additional treatment nursing services. Basic nursing can be reimbursed parallel to SPHC.

Two schemes allow compensation for travel expenses, and three schemes include fees for GPs and other physicians who cooperate with the palliative homecare team.

### Identification of a typology of team models using latent class analysis

Due to our limited sample size, we could only include 14 dichotomised variables in the LCA. For the identification of team models, we chose variables that had minimal missing values, provided sufficient variance and remained meaningful when dichotomised. In total, 186 teams were included in the latent class analysis, 10 teams had to be excluded because of insufficient data. While a three-class model showed the best model fit according to the BIC (see Table 8), we chose the four-class model with a slightly lower fit because of its higher face validity.

**Table 8:** Latent class analysis—model fit for different class numbers (*n* = 186)

Number of classes	Maximum log-likelihood	AIC	BIC	Likelihood ratio	Chi-Squared fit
1	-1264.0	2552.0	2590.7	846.7	4598.4
2	-1130.4	2310.8	2391.5	579.6	2971.4
3	-1093.4	2262.8	2385.4	505.6	3986.7
4	**-1060.6**	**2223.3**	**2387.8**	**440.0**	**2647.0**
5	-1030.3	2190.7	2397.1	381.4	2022.8
6	-1018.5	2191.0	2439.4	355.8	1568.9

**Fig. 2:**
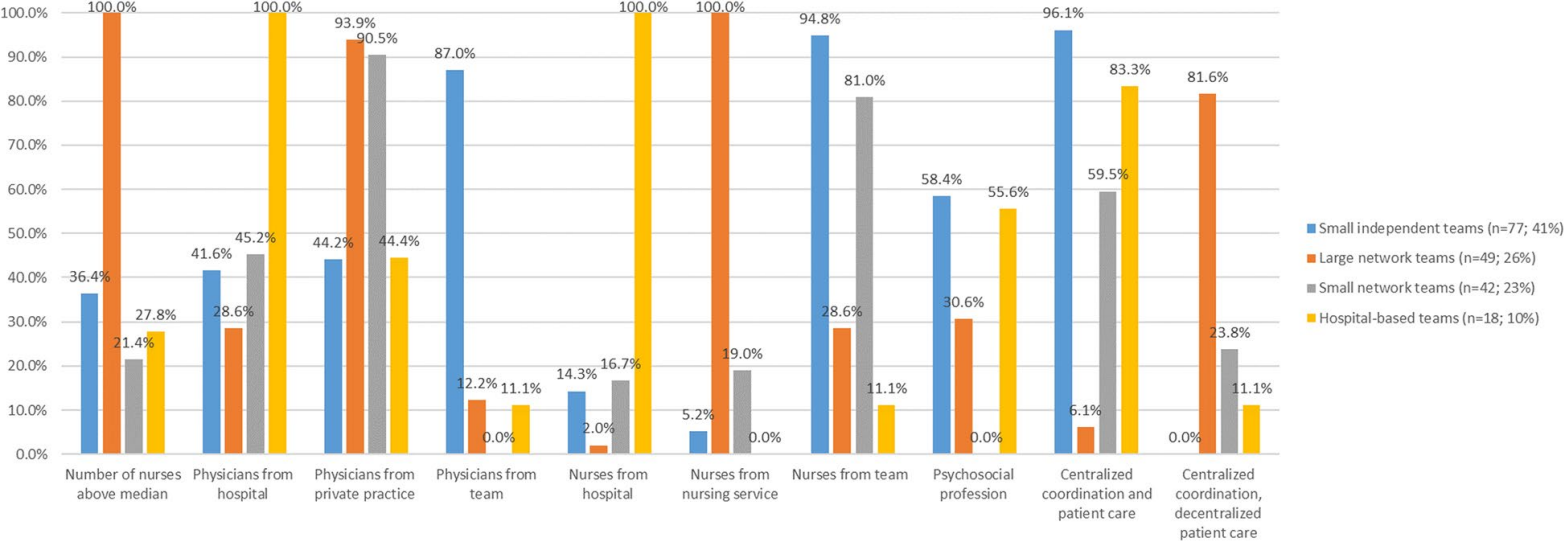
Team characteristics based on latent classes (*n* = 186). Percentage of teams (y-axis) with the specific characteristic (x-axis) from the latent classes

Table 9 shows the conditional item responses for the four-class model and Fig. 2 shows the variables in the different classes. Class 1 was identified as *small independent SPHC teams*, characterised by mainly directly employed physicians and nurses. Many of these teams included psychosocial professionals, and their coordination and patient care were centralised. With 77 teams, this was the largest class. Class 2 (*n* = 49) was identified as *large network teams* that all had more nurses than the median and worked predominantly with physicians from private practices and nurses from nursing services. Three-quarters reported centralised coordination and decentralised patient care. *Small network teams* (class 3, *n* = 42) employed nurses directly but the physicians came from private practices, while some also had decentralised patient care. In class 4 (*n* = 18), *hospital-based teams,* both the physicians and nurses were employed by hospitals. These teams showed a high rate of the inclusion of psychosocial professionals, as well as centralised coordination and patient care.

**Table 9:** Conditional item response probabilities (dichotomised variables)

Criteria for team structure	Class			
	**1 (small independent teams; *n* = 77)**	**2 (large network teams; *n* = 49)**	**3 (small network teams; *n* = 42)**	**4 (hospital-based teams; *n* = 18)**
**Physicians from hospital**	0.41	0.31	0.46	1.00
**Physicians from private practices**	0.45	0.94	0.92	0.44
**Physicians employed by team**	0.82	0.13	0.00	0.12
**Physicians from other structures**	0.18	0.24	0.14	0.00
**Number of nurses > = 9**	0.34	1.00	0.21	0.29
**Nurses from hospital**	0.15	0.03	0.17	1.00
**Nurses from mobile nursing services**	0.05	1.00	0.18	0.00
**Nurses employed by team**	0.93	0.31	0.82	0.10
**Nurses from other structures**	0.08	0.15	0.18	0.00
**Psychosocial profession in team**	0.55	0.31	0.00	0.56
**Centralised coordination and patient care**	0.96	0.09	0.56	0.82
**Centralised coordination, decentralised patient care**	0.00	0.79	0.27	0.13
**Other organisational structure**	0.04	0.13	0.17	0.05

## Discussion

In this study, a comprehensive set of the contractual preconditions and structural characteristics of SPHC teams in Germany was assessed for the first time. The results show a wide variance of these characteristics. We will first discuss whether they align with the nationwide requirements set by the SPHC directive, and the professional recommendations of the EAPC. We will subsequently elaborate on how the different structural types of SPHC teams may influence care provision.

### Requirements and recommendations for SPHC

#### Staff requirements and recommendations

The EAPC recommends that teams contain four to five full-time, specifically trained professionals, including physicians, nurses, social workers, and administrative staff [1]. German SPHC teams only partially meet these recommendations. In most teams, all the physicians and nurses had received specific palliative care training, as required by the SPHC directive [7]. In some teams, no physicians or nurse had their main focus of work in SPHC, and only a few contracts stipulate a mandatory number of staff members with SPHC as their main or exclusive activity, despite the EAPC recommandations stating that this would ensure patients experienced high-quality care [44]. Some model contracts (e.g. Hamburg [24]) also explicitly encourage specific team models, for instance, by making it obligatory to cooperate with mobile nursing services or general practitioners for SPHC provision.

Expert recommendations demand at least three different professions as part of a multi-professional team ([44, 45], recommendation 5.36), but neither the model contracts nor the actual team structures fully correspond to this. No model contract requires any other professionals than physicians and nurses, and they are not included in SPHC reimbursement schemes. A little over one-third of all the teams had a psychosocial professional as a team member. However, SPHC teams are free to realise their potential need for a third profession by cooperation contracts.

#### Cooperation with primary palliative care and other professionals

SPHC should complement rather than replace primary palliative care [7], and SPHC teams should focus on the tasks that require specialist knowledge. Cooperation with primary care providers is therefore essential. Most SPHC teams declared that they cooperated with primary care physicians and nursing services, mainly on an informal basis. Three contracts exclude parallel provision of SPHC and treatment nursing care at full or full and partial care levels. While this may offer advantages like the close monitoring of patients, and facilitated nursing management, it also increases the overall SPHC costs and may strain a team’s time and resources. Furthermore, an exclusion of parallel nursing services may lead to a discontinuity in nursing care for some patients.

SPHC patients should have easy access to other professionals such as physiotherapists, psychologists, or spiritual care workers, as needed [1]. The SPHC directive only defines cooperation with volunteer hospice services as mandatory [7]. Almost all the teams declared that they cooperated with volunteer hospice services. A substantial number of teams claimed to cooperate with psychosocial professionals. However, it remains unclear how this cooperation is realised and whether the cooperating partners’ services are in fact appropriately accessible for patients and their relatives.

#### Patient characteristics

On average, the teams cared for almost 400 patients in 2016 per team, with a high variation between the teams. Although many patients with non-malignant life-limiting diseases may have a high and complex symptom burden [46–49], and patients with any life-limiting, progressing disease are eligible for SPHC [7], our data show that most patients suffered from oncological diseases, which is consistent with other studies [12, 14, 17, 50, 51]. This points to a possible under-provision of SPHC for non-oncological patients in some areas, which needs further elaboration. Nevertheless, some teams cared for many non-oncological patients and it could be insightful to explore their patient flow, as well as their patient and care characteristics.

The average length of care of about a month is consistent with other studies, which demonstrate an average of between 19 [52] and 32 days [17], but lower than a recent Germany-wide study reported with an average of 57 days [53]. According to the SPHC directive, patients must have a life expectancy of not more than months, weeks, or days to be eligible for SPHC [7], but single teams reported individual patients being in care for up to three years. A systematic review has shown that the effects on quality of life are greater for patients receiving palliative care earlier [54], and the early integration of palliative care is an internationally recognised policy goal [55, 56]. However, our study suggests that most patients are admitted to SPHC late in the disease trajectory with short care durations.

#### Reimbursement

A large proportion of SPHC teams is small and not owned by larger providers. Compared to teams operating within large structures, these teams are under greater pressure to be economically efficient. Teams operating within larger structures may be better able to accommodate for temporal financial losses (fix cost degression). Teams that rely on network structures with primary physicians and nursing services for SPHC provision may pay these providers by performance (e.g. for each home visit) and due to the higher share of variable costs be less impacted by issues such as lower patient numbers than teams that continuously pay salaries to their employees.

The financial incentives that arise from the type of reimbursement can influence team structures and the type of services delivered [11], which may subsequently influence the quality of patient care. Cut-offs for the number of home visits, for example, may entail the risk that home visits are under-provided since they are not recompensated beyond the cut-off. On the other hand, unrestricted fee-for-service payments may set the financial incentive to invoice maximum amounts of services.

Care network coordination as an essential task of SPHC teams [7, 44] requires own resources, but is not explicitly covered by reimbursement items in most contracts [17].

Each reimbursement scheme entails its own framework for business decisions such as contracting personnel, and inner reimbursement rules within SPHCteams. In any case, management know-how is essential for SPHC-teams to be economically efficient.

### Implications of identified classes of SPHC teams

Hereinafter, we discuss the implications of structural characteristics in relation to the four types of SPHC teams identified by latent class analysis.

Most SPHC teams belong to the class of ‘small independent teams’, and only a small percentage of the teams are ‘hospital-based’. ‘Large’ and ‘small network teams’ mainly work with physicians from private practices, and nurses who are from mobile nursing services or employed by the team.

#### Network teams

Large network teams usually have many team members from different institutions. One team stated that they worked with almost 250 nurses. To put this into perspective, the only international study on SPHC team structures (from Sweden) found that teams had 14 members on average (with a range of three to 40), while the German teams had 30 staff members on average [57]. Working with physicians and nurses from several institutions (e.g., hospitals or private practices) could also mean that team managers must accommodate different institutional requirements, such as hospital schedules and office hours. Teams operating as networks need additional resources, both personnel (such as coordinators) and materials (such as rooms large enough for team meetings, or adequate communication devices). Teamwork as an integral part of SPHC could be challenging to be established in large networks comprising many members with part-time SPHC contracts. Timely and effective inter-professional information exchange is essential for providing high-quality palliative care across different settings [26], which may pose a challenge for network teams. Furthermore, care quality may differ between team members, and is more difficult to measure in large network teams.

Small network teams work with physicians from private practices, but directly employ nurses. This model may lead to more centralised coordination and care, as well as a more “nurse-based” palliative care model.

Network teams may be able to provide timely and responsive care [5] in settings with low population density and long distances. The strong collaboration between primary and specialist palliative care in network teams may be an advantage in terms of the continuity of care and smooth transitions from primary to specialist palliative care in outpatient settings. Primary palliative care patients may also benefit from network models because more GPs also work in SPHC and thus have greater expertise in this field. On the other hand, GPs working in SPHC may also be more motivated to refer patients to SPHC even if primary care would be sufficient.

#### Small independent teams and hospital-based teams

Small independent teams and hospital-based teams follow a “classical” team approach. While communication between team members may be easier, they often have to rely more on external patient care providers, like GPss and nursing services, and ensure that the information flow with them is adequate. They are more likely than network teams to contain psychosocial professionals, indicating that such professions are more available in certain team models. Including psychosocial professionals in SPHC teams may facilitate the realisation of a holistic approach to palliative care that addresses all the dimensions of care [5]. Their “core-team” structure makes it easier to implement and perform quality assurance measurements.

Hospital-based teams, which are part of a palliative care centre with a palliative care unit may have better access to in-patient facilities, as well as a variety of professions and medical disciplines through the hospital’s infrastructure. This facilitates a smooth transition between in- and outpatient settings but may also lead to more hospital admissions as well as prescription of SPHC upon discharge, even if primary care would be sufficient.

#### Overarching considerations

Team models and the underlying team criteria should be considered when process and outcomes data are analysed. In a study that aimed to create a typology of German SPC settings, experts in focus groups agreed on (among other factors) structural attachment, care organisation, size and additional professions as variables structuring a typology of SPHC teams [58]. In own research we found no connection between team structures and patient-reported care quality [59]. Still, the question of whether team models result in differences in patient care and care quality needs further elaboration.

## Limitations

Our sample was limited to 196 of the (approximately) 283 SPHC teams active in Germany at the time of the study. Due to a technical problem, our first data extraction was skewed, and we had to ask the affected teams to validate their data. This limited our sample further because 245 teams had initially contributed data, but data from only 196 were available for data validation.

The response rate is difficult to determine, as the numbers vary between sources [39, 41, 43] and none of these are completely reliable. We included only teams established until 2017. In the meantime, new teams may have been established and the characteristics of existing teams may have changed, although a recent analysis of spatial accessibility found a comparable number (289) of SPHC teams in Germany [60]. When taking structures into account, data should always be updated to accommodate changes in teams. Nevertheless, our analysis still contributes important information regarding structural characteristics and, while details such as staff member numbers may change, SPHC teams are presumably consistent in terms of their underlying type.

We had difficulty in assessing some staff characteristics, such as FTE. In particular, teams working with physicians from private practices and/or nurses from nursing services did not know how much of their time these physicians and nurses spent working in SPHC. Therefore, the FTE numbers are skewed towards smaller teams.

As the data in the *Wegweiser* databank are voluntary, and not regularly updated, we recommend that the databank is only used for scientific purposes if the data are reassessed specifically for study purposes as we did in this study.

The sample size and therefore the power might not be sufficient considering the number of classes in the model. In particular, the model can fail to uncover classes with low memberships [36]. However, to estimate the necessary sample size, the true (but unknown) model would need to be specified, so the results of the LCA are exploratory only.

## Conclusions

The characteristics of SPHC differ widely across Germany. Different regional and contractual factors contribute to this variance. Studies have shown that SPHC may improve patient satisfaction, symptom control, and quality of life; reduce unnecessary healthcare utilisation and potentially aggressive interventions [51, 61, 62]; and ultimately be cost-saving for end-of-life patients [63]. However, no data are available to indicate how these services should be organised to ensure the best outcomes, and the potential consequences of different organisational aspects for patient care remain unclear. The heterogeneity of teams structures and organisation complicate comparisons between teams individually and between regions. To understand and interpret the patient-related process and outcome data of specialist palliative homecare, the effects arising from the respective contractual, structural, and organisational characteristics must be understood [16]. The four team model types we identified can facilitate comparisons between teams to determine which team model might be the most suitable for different settings and regional conditions both in terms of effectiveness regarding outcomes and efficiency regarding outcomes in relation to costs. When analysing patient data from SPHC, team models should be assessed as structural information. Further research is needed regarding the consequences of the team models on care organisation, teamwork, regional care networks, and quality of care, including the perspective of patients and relatives. Moreover, underlying variables like team size and composition should be considered. Additionally, specific contractual terms regarding, for example, the exclusion of treatment nursing care in some contracts, or cut-offs for home visits, should be taken into account when analysing and interpreting process and outcomes data. Recently, a national framework contract was published [64]. It includes preconditions that every SPHC team has to adhere. Among others, 2 FTE physicians, 4 FTE nurses directly employed by teams, with at least 18 h/week in SPHC are mandatory; one nurse and one physician with each at least 0.75 FTE has to provide professional leadership. Both medical and nursing services must be available 24/7. Reimbursement should be based on daily, weekly, monthly or case rates. The future will show how these new preconditions, effective from January 1, 2023, will influence SPHC structures, and what effect they will have on service provision.

### Supplementary Information

**Additional file 1. **Model Contracts (in German).

**Additional file 2: ** Additional tables (**Table 1.** Variables assessed in the Wegweiser Hospiz- und Palliativversorgung databank. Variables in italics were used in the latent class analysis. **Table 2.** Detailed description of institutional affiliations of physicians and nurses in SPHC (n=196)).

**Additional file 3. **SAVOIR LCA statistical codes. 

## Data Availability

Data can be obtained from the corresponding author on reasonable request.
