# Supplementary material for: Structural characteristics and contractual terms of specialist palliative homecare in Germany
Source: BMC Palliat Care. 2023 Oct 31;22:166. doi: 10.1186/s12904-023-01274-6 (PMC10617175; doi:10.1186/s12904-023-01274-6)
Supplement: Supplementary file 1 — Additional file 1. Model Contracts (in German). [file 12904_2023_1274_MOESM1_ESM.pdf]

Additional file 1: Model Contracts (in German)

**Content**

Bavaria........................................................................1
Baden-Württemberg ....................................................44
Berlin ........................................................................84
Brandenburg ...............................................................123
Bremen ......................................................................152
Hamburg.....................................................................173
Hesse ........................................................................209
Lower Saxony .............................................................242
Mecklenburg-Western Pomerania...............................257
North Rhine ................................................................297
Rhineland-Palatinate ..................................................431
Saxony-Anhalt ............................................................505
Saxony/Thuringia .......................................................516
Schleswig-Holstein.......................................................541
Westfalia-Lippe ..........................................................574

BAVARIA

# Muster-Vertrag über die Erbringung

# Spezialisierter Ambulanter Palliativversorgung (SAPV) gemäß §§ 132 d i. V. m. 37 b SGB V im Freistaat Bayern

**zwischen**

**den Landesverbänden der Krankenkassen**

der AOK Bayern - Die Gesundheitskasse,
dem BKK Landesverband Bayern
handelnd für die dem Vertrag beitretenden Betriebskrankenkassen,
der Vereinigten IKK,
dem Funktionellen Landesverband der landwirtschaftlichen Kranken- und Pflegekassen in Bayern,
der Knappschaft - Regionaldirektion München,

**den Ersatzkassen**

Barmer Ersatzkasse,
Techniker Krankenkasse,
Deutsche Angestellten-Krankenkasse,
KKH Allianz,
Gmünder ErsatzKasse – GEK,
HEK - Hanseatische Krankenkasse,
Hamburg Münchener Krankenkasse
hkk,

**gemeinsamer Bevollmächtigter mit Abschlussbefugnis**
**Verband der Ersatzkassen e. V. (vdek),**
**vertreten durch den/die Leiter/in der Landesvertretung Bayern**

**im Folgenden Krankenkassen genannt**

**und**

***Name, Rechtsform,***
***Sitz, vertreten durch ......***

**im Folgenden „Leistungserbringer“ genannt**

Präambel ... 3
§ 1 Vertragsziel ... 4
§ 2 Versorgungskonzept ... 4
§ 3 Versorgungsgebiet ... 5
§ 4 Qualifikationsvoraussetzungen ... 6
§ 5 Zulassungsvoraussetzung ... 6
§ 6 Sächliche und räumliche Ausstattung ... 7
§ 7 Anzeigepflichten / Mitteilungspflichten ... 7
§ 8 Patientendokumentation ... 8
§ 9 Qualitätssicherungsmaßnahmen ... 9
§ 10 Fortbildung ... 9
§ 11 Qualitätsprüfung ... 10
§ 12 Grundsätze der Leistungserbringung ... 10
§ 13 Inhalt der Leistung / Leistungskategorien ... 11
§ 14 Verordnung von Arzneimitteln, Heil- und Hilfsmitteln ... 12
§ 15 Anspruchsvoraussetzungen ... 12
§ 16 Verordnung / Genehmigung / Beginn der SAPV ... 13
§ 17 Kostenübernahme ... 13
§ 18 Dauer der Versorgung ... 14
§ 19 Zuzahlungen ... 14
§ 20 Vermittlungsverbot, Werbung und Einflussnahme ... 14
§ 21 Institutionskennzeichen ... 15
§ 22 Leistungsnachweis ... 15
§ 23 Abrechnung ... 15
§ 24 Statistik ... 17
§ 25 Datenschutz, Schweigepflicht ... 17
§ 26 Maßnahmen bei Vertragsverstößen des Leistungserbringers ... 17
§ 27 Vergütung ... 18
§ 28 Inkrafttreten / Kündigung ... 18
§ 29 Salvatorische Klausel ... 19
Anlage 1 Richtlinie des Gemeinsamen Bundesausschusses ... 21
Anlage 2 Gemeinsamen Empfehlungen der Spitzenverbände der Krankenkassen gemäß § 132 d Abs. 2 SGB V ... 25
Anlage 3 Versorgungsgebiet ... 30
Anlage 4 Vorzulegende Unterlagen bei Zulassungsantrag ... 31
Anlage 5 Evaluation ... 33
Anlage 6 Formular für den Leistungsnachweis ... 36
Anlage 7 Vergütungsvereinbarung ... 37
Anlage 8 Übergangsregelung ... 38
Anlage 9 Statistik ... 39
Anlage 10 SAPV-BSNR-Vereinbarung ... 40

## Präambel

Die spezialisierte ambulante Palliativversorgung (SAPV) dient dem Ziel, die Lebensqualität und die Selbstbestimmung schwerstkranker Menschen zu erhalten, zu fördern und zu verbessern und ihnen ein menschenwürdiges Leben bis zum Tod in ihrer häuslichen Umgebung oder in stationären Pflegeeinrichtungen nach § 72 SGB XI, der Eingliederungshilfe für behinderte Menschen, der Kinder- und Jugendhilfe zu ermöglichen. Versorgungsunsicherheiten, die zu unnötigen und die Versicherten stark belastenden Krankenhauseinweisungen führen, sollen durch die in der SAPV vorgehaltenen besonderen Expertisen vermieden werden. Symptome und Leiden sollen einzelfallgerecht und gemäß dem Willen des Versicherten und gemäß den Grundsätzen von Palliative Care gelindert oder behoben werden.

Die Versorgung des Versicherten bedarf einer besonderen Fachkompetenz in der Versorgung Schwerstkranker und Sterbender. Die Leistungen müssen ausreichend und zweckmäßig sein, sie orientieren sich an dem individuellen Hilfebedarf des Versicherten, dürfen das Maß des Notwendigen nicht überschreiten und müssen wirtschaftlich erbracht werden.
Das soziale Umfeld wird als wesentlicher Faktor für das Verbleiben im gewohnten Umfeld in die Versorgung mit einbezogen.

Die SAPV ergänzt das bestehende Versorgungsangebot, insbesondere das der Vertragsärzte, Krankenhäuser und Pflegedienste einschließlich der Versorgungsangebote der allgemeinen ambulanten palliativmedizinischen Versorgung (AAPV). Je nach Umfang der Patientenversorgung, vor allem aber bei der Vollversorgung i.R. der SAPV, kann die SAPV im Einzelfall die Regelversorgung nach dem SGB V ersetzen.

## § 1 Vertragsziel

(1) Dieser Vertrag dient der Sicherstellung der bedarfsgerechten individuellen Versorgung der anspruchsberechtigten Versicherten mit SAPV gemäß § 37 b SGB V in dem vertraglich vereinbarten Versorgungsgebiet nach § 3 durch den Leistungserbringer.

(2) [1]Die SAPV umfasst die Behandlung von Patienten mit einer nicht heilbaren, progredienten und weit fortgeschrittenen Erkrankung und einem ausgeprägten Symptomgeschehen, die eine besonders aufwändige Versorgung benötigen. [2]Sie soll erträgliche Lebenszeit trotz schwerster Krankheit und drohendem Tod im ambulanten Bereich, insbesondere auch das Sterben zu Hause, ermöglichen.

(3) [1]Bei der SAPV handelt es sich um eine interdisziplinäre Aufgabe. Sie wird durch ein Team mit mindestens palliativpflegerischer und palliativmedizinischer Fachkompetenz erbracht. [2]Dieser Leistungserbringer nach § 132d SGB V (im Folgenden Palliative-Care-Team (PCT) genannt) ist Teil einer multiprofessionell vernetzten Versorgungsstruktur im regionalen Gesundheits- und Sozialsystem [3]Das PCT arbeitet gemeinsam mit den je nach Bedarf an der Versorgung beteiligten Personen unterschiedlicher Berufsgruppen, wie z. B. Haus-/Fachärzten, Pflegediensten, Seelsorgern, Sozialarbeitern, Physiotherapeuten, Psychologen, Ehrenamtlichen und ambulanten Hospizdiensten gemäß § 39 a Abs. 2 SGB V, ggf. in Form von Kooperationen eng zusammen.

(4) Bestandteil dieses Vertrages sind:

- die „Richtlinie des Gemeinsamen Bundesausschusses zur Verordnung von spezialisierter ambulanter Palliativversorgung“ (Spezialisierte Ambulante Palliativversorgungs-Richtlinie/SAPV-RL – **Anlage 1**) und
- die „Gemeinsamen Empfehlungen der Spitzenverbände der Krankenkassen nach § 132 d Abs. 2 SGB V für die spezialisierte ambulante Palliativversorgung“ (**Anlage 2**)

in ihrer jeweils aktuellen Fassung.

## § 2 Versorgungskonzept

(1) [1]Das PCT handelt nach einem verbindlichen, strukturierten und schriftlich dargelegten Versorgungskonzept. [2]Dieses beschreibt den inhaltlichen und organisatorischen Rahmen der Leistungserbringung (inklusive der personellen und sächlichen Ausstattung) sowie die Einbindung in die regionale Versorgungsstruktur. [3]Weitere Bestandteile sind die Beschreibung der Erbringung von Beratungs- und Koordinationsleistung, Teil- und Vollversorgung. [4]Das Versorgungskonzept verdeutlicht, wie die qualifizierte Leistungserbringung erfolgt, so dass die in § 1 dieses Vertrages genannten Vertragsziele erreicht werden. [5]Das Versorgungskonzept ist Grundlage des Versorgungsvertrages.

(2) Die regionalen Netzwerkstrukturen und deren Besonderheiten sind zu beschreiben, in die sich der Leistungserbringer im Rahmen der Vernetzung integriert.

(3) Das Versorgungskonzept beinhaltet folgende Aspekte:

a) Standort des Leistungserbringers,
b) Versorgungsgebiet,
c) Versorgungsprozess,
d) Kooperationspartner,
e) Mitarbeiterkapazität,
f) Personelle Zuständig- und Verantwortlichkeiten,
g) Qualifikationsnachweise der Mitarbeiter des PCT gemäß § 4,
h) Sächliche Ausstattung gemäß § 6,
i) Dokumentationssystem,
j) Qualitätssicherungsmaßnahmen gemäß § 9,
k) Konzept zur Sicherstellung der reibungslosen Versorgung mit Arznei-, Heil- und Hilfsmitteln,
l) Konzept zur ganzjährigen Sicherstellung der 24-Stunden-Einsatzbereitschaft für die in der Versorgung befindlichen Patienten und Angehörigen inklusive Notfallmanagement in Absprache mit der Primärversorgung,
m) Überleitungsmanagement zwischen ambulanter und stationärer Versorgung,
n) Fallzahleinschätzung pro Kategorie,
o) Beschreibung der Infrastruktur zur Sicherstellung der ständigen Kommunikation innerhalb des PCT und zu allen an der Versorgung des jeweiligen Versicherten Beteiligten.

(4) Der Leistungserbringer ist in der Ausführung der verordneten Leistung weisungsunabhängig und trägt die alleinige Verantwortung für das Versorgungskonzept.

## § 3 Versorgungsgebiet

(1) Das Versorgungsgebiet umfasst den in der **Anlage 3** beschriebenen Einzugsbereich.

(2) Der Leistungserbringer stellt im Rahmen seiner Kapazitäten die Versorgung der Versicherten in seinem Versorgungsgebiet mit Leistungen der SAPV sicher.

(3) [1]Der Versicherte ist in der Wahl des Leistungserbringers frei, soweit dieser zur Erbringung der SAPV im Versorgungsgebiet gemäß Abs. 1 berechtigt ist. [2]Auf diese Wahlfreiheit darf der Leistungserbringer keinen Einfluss nehmen. [3]Wählt er einen anderen Leistungserbringer , kann die Krankenkasse die Übernahme der hierdurch entstehenden Mehrkosten ablehnen. [4]Über etwaige Mehrkosten hat der Leistungserbringer den Versicherten und die Krankenkasse vor Beginn der Leistungserbringung zu informieren.

## § 4 Qualifikationsvoraussetzungen

Die Leistungen der SAPV werden durch Personen erbracht, die folgende Qualifikationsvoraussetzungen erfüllen:

a) Ärztinnen und Ärzte,

   I. die über eine anerkannte Zusatzweiterbildung Palliativmedizin nach der aktuell gültigen Weiterbildung der jeweiligen Landesärztekammer verfügen und

   II. die Erfahrung aus der ambulanten palliativen Behandlung von mindestens 75 Palliativpatienten haben, z.B. in der häuslichen Umgebung oder in einem stationären Hospiz, innerhalb der letzten drei Jahre oder aus einer mindestens einjährigen klinischen palliativmedizinischen Tätigkeit in einer Palliativabteilung in einem Krankenhaus innerhalb der letzten drei Jahre;

b) Pflegefachkräfte,

   I. die über die Erlaubnis zur Führung einer der Berufsbezeichnungen Gesundheits- und Krankenpflegerin / Gesundheits- und Krankenpfleger, Gesundheits- und Kinderkrankenpflegerin / Gesundheits- und Kinderkrankenpfleger oder Altenpflegerin / Altenpfleger (dreijährige Ausbildung aufgrund einer landesrechtlichen Regelung) entsprechend den gesetzlichen Bestimmungen in der jeweils gültigen Fassung verfügen und

   II. die den Abschluss einer Palliative-Care-Weiterbildungsmaßnahme im Umfang von mindestens 160 Stunden vorweisen können und

   III. die Erfahrung durch mindestens eine zweijährige praktische Tätigkeit als Pflegefachkraft in der Betreuung von Palliativpatienten in den letzten drei Jahren, davon mindestens sechsmonatige Mitarbeit in einer spezialisierten Einrichtung der Hospiz- und Palliativversorgung, nachweisen können.

c) Soweit weitere Fachkräfte (z.B. Sozialarbeiterinnen / Sozialarbeiter, Sozialpädagoginnen oder Sozialpädagogen, Psychologinnen oder Psychologen) vertraglich eingebunden werden, haben diese eine Zusatzweiterbildung Palliative Care für andere Berufsgruppen oder eine mehrjährige Erfahrung in der Palliativversorgung nachzuweisen.

## § 5 Zulassungsvoraussetzung

(1) [1]Das PCT ist eine räumlich getrennte, personell und finanziell eigenständige Organisationseinheit. [2]Der Leistungserbringer stellt sicher, dass das für die SAPV erforderliche und geeignete Personal zur Verfügung steht.

(2) Das PCT übernimmt nach Maßgabe der gesetzlichen Vorschriften die Haftung für Schäden, welche die Mitarbeiter des PCT in Ausübung ihrer Tätigkeit verursachen.

(3) Die Nachweise nach **Anlage 4** sind bei Beantragung der Zulassung bei der Arbeitsgemeinschaft der Krankenkassenverbände in Bayern c/o AOK Bayern die Gesundheitskasse, Carl-Wery-Straße 28, 81739 München vorzulegen.

(4) [1]Die Leistungserbringung ist durch den Leistungserbringer in Form geeigneter Maßnahmen unter Einsatz entsprechender personeller und sächlicher Mittel für die Dauer des Vertrages sicherzustellen. [2]Die Mitarbeiter des PCTs sind in allen medizinischen und pflegerischen Fragen nur gegenüber dem jeweils leitenden Arzt bzw. der leitenden Pflegefachkraft weisungsgebunden.

(5) Das PCT besteht regelhaft aus mindestens 4 Planstellen.

## § 6 Sächliche und räumliche Ausstattung

(1) [1]Eine Versorgung mit Arznei, Heil- und Hilfsmitteln im Notfall (Notfallversorgung) ist selbst oder durch geeignete Maßnahmen, z. B. durch eine Kooperation mit einem Sanitätshaus, sicherzustellen. [2]Von besonderer Bedeutung ist die Vorhaltung ausreichender und geeigneter Arzneimittel/BtM für die Versorgung im Not- und Kriseninterventionsfall. [3]Das PCT muss jederzeit auf die Mittel der Notfallversorgung zurückgreifen können.

(2) Die an die Aufbewahrung von BtM gestellten Voraussetzungen nach dem BtMG sind zu berücksichtigen.

(3) Der Leistungserbringer hat darüber hinaus als Mindestanforderung an die sächliche Ausstattung Folgendes vorzuhalten bzw. sicherzustellen:

a) eine geeignete, aktuell geführte und für die an der Versorgung Beteiligten jederzeit zugängliche Patientendokumentation,
b) eine ausreichende und geeignete Mobilität zur zeitnahen häuslichen Versorgung der Patienten,
c) Arzt-/Pflegekoffer/Bereitschaftstasche,
d) eine geeignete administrative Infrastruktur, u.a. Büro, Kommunikationstechnik.

(4) Der Leistungserbringer muss über eine eigene Postadresse und geeignete Räumlichkeiten für

e) die Beratung von Patienten und Angehörigen,
f) Teamsitzungen und Besprechungen,
g) die Lagerhaltung von eigenen Medikamenten für Notfall- / Krisenintervention und von Hilfsmitteln

verfügen.

## § 7 Anzeigepflichten / Mitteilungspflichten

(1) [1]Der Leistungserbringer ist verpflichtet, der Arbeitsgemeinschaft der Krankenkassenverbände in Bayern innerhalb von vier Wochen Änderungen wesentlicher Umstände im Versorgungskonzept schriftlich mitzuteilen. [2]Dies sind insbesondere:

a) Änderungen der betrieblichen/organisatorischen Gegebenheiten, insbesondere Personaländerungen, soweit die Qualifikation von den Vorgaben des § 4 (Qualifikationsvoraussetzungen) abweicht,

b) Änderungen von Kooperationsvereinbarungen (Neuabschlüsse, Kündigung, inhaltliche Änderungen),

c) Eingeleitete staatsanwaltschaftliche Ermittlungsverfahren gegen Mitarbeiter des PCT,

d) Bestehen oder Übernahme einer Betreuerstellung im Sinne des BGB eines Mitarbeiters im PCT zum Patienten.

(2) Die Krankenkasse ist berechtigt, bei begründetem Anlass zum Nachweis der organisatorischen und/oder personellen Voraussetzungen weitere Unterlagen zu verlangen.

## § 8 Patientendokumentation

(1) [1]Der Leistungserbringer hat eine Patientendokumentation zu führen, aus der zu jeder Zeit alle notwendigen Informationen über den Versicherten und die erbrachten Leistungen hervorgehen. [2]Das PCT aktualisiert regelmäßig die Patientendokumentation.

(2) Im kooperativen Netzwerk hat der Leistungserbringer zu gewährleisten, dass die von ihm angewandte Dokumentation kompatibel zu bestehenden Dokumentationssystemen der an der Versorgung beteiligten Leistungserbringer ist.

(3) Die Dokumentation durch das PCT beinhaltet in Abhängigkeit der Leistungskategorie insbesondere:

a) ein Versichertenstammblatt (z. B. mit Angaben zum Versicherten und zu Angehörigen, Hausarzt, Pflegedienst und weiteren involvierten Diensten, Diagnosen, Absprachen, Behandlungsort, Medikation, Maßnahmen, u. w.),

b) ein Assessment mit in der Regel folgenden Inhalten – unabhängig vom Leistungsanspruch gegenüber der Krankenkasse:

i. Körperlicher und psychischer Zustand des Versicherten,
ii. Wünsche des Versicherten / der Angehörigen,
iii. Familiäre Belastungssituation,
iv. Stand der Aufklärung des Versicherten / der Angehörigen,
v. Medikation,
vi. Hilfsmittel,
vii. Physikalische und andere Therapien,
viii. Beratungs- und/oder Behandlungsbedarf,

c) einen vorausschauenden Behandlungsplan einschließlich Notfallplan,

d) einen Nachweis über durchgeführte Maßnahmen (Datum, Dauer, Handzeichen),

e) einen aktuellen Medikamentenplan,

f) Untersuchungsbefunde,

g) Verlaufsbögen zur Erfassung aktueller Probleme und deren Lösung.

(4) Die versorgungsrelevante Dokumentation verbleibt beim Patienten.

(5) Die Dokumentation ist dem MDK und – soweit der Versicherte oder sein Vertreter schriftlich eingewilligt hat (§67 b Abs. 1 SGB X) – der Krankenkasse auf Anforderung zugänglich zu machen.

(6) Ist ein SAPV-Fall beendet, wird eine anonymisierte Erfassung zur bundesweiten Evaluation durchgeführt (siehe **Anlage 5**).

(7) Die Aufbewahrungsfrist für die Dokumentation beträgt 10 Jahre nach Ende des Kalenderjahres der Leistungserbringung.

## § 9 Qualitätssicherungsmaßnahmen

(1) [1]Der Leistungserbringer ist verpflichtet, ein einrichtungsinternes Qualitätsmanagement durchzuführen, das im Versorgungskonzept darzustellen ist. [2]Zur Qualitätssicherung zählen interne und externe Maßnahmen.

(2) Zu den Qualitätssicherungsmaßnahmen gehören insbesondere:

a) ein interdisziplinär abgestimmter individueller Behandlungsplan,
b) regelmäßige, mindestens einmal wöchentlich stattfindende interdisziplinäre Fallbesprechungen (gemeinsame Dienstbesprechungen),
c) ein geeignetes Dokumentationssystem,
d) regelmäßige gemeinsame externe Supervisionen,
e) die Teilnahme an halbjährlichen multidisziplinären Qualitätszirkeln,
f) ein Fortbildungskonzept.

(3) Die Kooperationspartner des Leistungserbringers werden in Maßnahmen zur Qualitätssicherung eingebunden.

## § 10 Fortbildung

(1) [1]Der Leistungserbringer ist verpflichtet, die Fachlichkeit aller Mitarbeiter des PCT, die länger als ein Jahr beim Leistungserbringer tätig sind, durch regelmäßige Teilnahme an berufs- bzw. aufgabenbezogenen, internen und externen Fortbildungsmaßnahmen sicherzustellen. [2]Die Maßnahmen sollen insbesondere den aktuellen palliativ-medizinischen/ -pflegerischen Stand vermitteln und nicht ausschließlich als interne Schulungen aller Beschäftigten durchgeführt werden. [3]Fachbezogene Literatur ist vorzuhalten.

(2) [1]Der Umfang der Fortbildungen beträgt mindestens 20 Unterrichtsstunden pro Vollzeitstelle innerhalb von zwei Kalenderjahren. [2]Im Einzelfall können fachbezogene Weiterbildungen als Fortbildung anerkannt werden.

(3) [1]Der Nachweis hierüber hat bei Aufforderung durch eine vertragschließende Krankenkasse oder der Arbeitsgemeinschaft der Krankenkassenverbände innerhalb eines Monats für die zurückliegenden zwei Kalenderjahre zu erfolgen. [2]Art, Inhalt und

Umfang sowie die namentliche Auflistung der teilnehmenden Mitarbeiter der einzelnen Fortbildungsmaßnahmen sind nachvollziehbar zu dokumentieren.

(4) Kommt der Leistungserbringer seiner Nachweispflicht nicht oder nicht vollständig innerhalb eines Monats nach, wird eine Nachfrist von einem weiteren Monat gesetzt.

(5) Weist der Leistungserbringer die Erfüllung der Fortbildungsverpflichtung nach Absatz 1 und 2 nicht oder nicht vollständig nach, sind die Krankenkassen berechtigt, für alle Leistungen, die in den ersten 4 Kalendermonaten nach Mitteilung des Prüfungsergebnisses durch das PCT erbracht werden, die Abrechnungen um 5 %, ab dem folgenden Kalendermonat um 25 % zu kürzen.

(6) [1]Fehlende Fortbildungszeiten sind nach Aufforderung nachzuholen; die nachgeholte Fortbildung wird auf den folgenden Zweijahreszeitraum nicht angerechnet. [2]Die Vergütungskürzung gilt längstens für die Leistungen, die in dem Monat erbracht werden, in dem der vollständige Fortbildungsnachweis der auffordernden Krankenkasse oder der Arbeitsgemeinschaft der Krankenkassenverbände vorgelegt wird.

(7) Erbringt der Leistungserbringer den Fortbildungsnachweis nicht spätestens ein halbes Jahr, nachdem er durch die Krankenkasse oder die Arbeitsgemeinschaft der Krankenkassenverbände in Bayern auf Defizite bei der Fortbildung hingewiesen wurde, kann der Vertrag gekündigt werden.

## § 11 Qualitätsprüfung

(1) Die Krankenkassen und ihre Verbände sind berechtigt, die Struktur-, Prozess- und Ergebnisqualität der Leistungserbringer mit Hilfe des MDK auch unangemeldet zu prüfen, ggf. auch in den Räumlichkeiten des Leistungserbringers.

(2) Die Kooperationspartner nach § 2 Abs. 3des Vertrages können in alle Maßnahmen zur Qualitätsprüfung eingebunden werden.

(3) Maßstäbe für die Beurteilung der Leistungsfähigkeit eines PCT und die Qualität der Leistungen sind die für sie verbindlichen Anforderungen in den Richtlinien des Gemeinsamen Bundesausschusses nach § 92 Abs. 1 Satz 2 Nr. 14 SGB V in Verbindung mit § 37 b Abs. 3 SGB V und in den Gemeinsamen Empfehlungen der Spitzenverbände der Krankenkassen gemäß § 132 d Abs. 2 SGB V.

(4) Die anlässlich der Prüfung gewonnenen Daten dürfen nur im Rahmen des Prüfungszweckes und der gesetzlichen Aufgabenerfüllung verwendet werden.

## § 12 Grundsätze der Leistungserbringung

(1) [1]Der Leistungserbringer stellt sicher, dass die Leistungen nach diesem Vertrag nur im notwendigen Umfang in Anspruch genommen und ausreichend, zweckmäßig und wirtschaftlich erbracht werden. [2]Er gewährleistet, dass die Weiterführung der SAPV rund um die Uhr sicher gestellt ist.

(2) [1]Der Leistungserbringer haftet gegenüber dem Versicherten und der Krankenkasse nach den Bestimmungen des Bürgerlichen Gesetzbuches (§ 61 SGB X). [2]Er stellt sicher, dass erforderliche Versicherungen in ausreichender Höhe abgeschlossen sind und stellt die Krankenkasse von Ansprüchen frei, die gegenüber dieser im Zusammenhang mit der Erbringung der Leistungen nach diesem Vertrag von Dritten geltend gemacht werden.

(3) [1]Die SAPV ergänzt das bestehende Versorgungsangebot. [2]Die AAPV hat Vorrang vor der SAPV.

(4) Die vorzeitige Beendigung der Leistung wird innerhalb von drei Arbeitstagen angezeigt.

## § 13 Inhalt der Leistung / Leistungskategorien

(1) Der Leistungserbringer erbringt die jeweils ärztlich verordnete SAPV-Leistung intermittierend oder durchgängig nach Bedarf als

a) Beratungsleistung,
b) Koordination der Versorgung,
c) additiv unterstützende Teilversorgung,
d) Vollversorgung.

(2) Zur Beratungsleistung gehören:

I. Telefonat bzw. persönliches Gespräch mit Betroffenen (Kranken oder deren Angehörige) und/oder,
II. Telefonat bzw. persönliches Gespräch mit einem der Leistungserbringer der Primärversorgung und,
III. Dokumentation und Evaluation.

(3) Die Koordination der Versorgung umfasst:

I. den direkten persönlichen Kontakt zum Betroffenen (Kranken oder deren Angehörige),
II. die ressourcenfokussierte Versorgungsplanung des PCT,
III. das Assessment, die Beratung für Therapie und das Vorgehen im Notfall,
IV. eine Vernetzung mit ambulant tätigen und/oder stationären Leistungserbringern,
V. die Dokumentation und Evaluation.

(4) 1Zur additiv unterstützenden Teilversorgung gehören:

I. Beratungs- und Koordinationsleistungen,
II. Hausbesuche,
III. bei Bedarf einzelne Leistungen des in § 5 Abs. 3 der SAPV-Richtlinie des G-BA aufgeführten Leistungskatalogs,
IV. 24-Stunden-Bereitschaft für die verordnete Leistung,
V. die Dokumentation und Evaluation.

2Die 24-Stunden-Bereitschaft kann nicht als alleinige Teilleistung verordnet werden.

(5) Zur Vollversorgung gehören:

I. Beratung und Koordination sowie 24-Stunden-Bereitschaft,
II. Hausbesuche,
III. bei Bedarf alle Leistungen des in § 5 Abs.3 der SAPV-Richtlinie des G-BA aufgeführten Leistungskatalogs ergänzend zu den ambulanten Leistungserbringern,
IV. die Dokumentation und Evaluation.

(6) Angehörige und dem Versicherten nahestehende Personen werden in die Pflege sowie in die psychosoziale Unterstützung und Sterbebegleitung mit einbezogen.

## § 14 Verordnung von Arzneimitteln, Heil- und Hilfsmitteln

(1) [1]Die Verordnung von Arznei-, Heil- und Hilfsmitteln erfolgt unter Verwendung der für die vertragsärztliche Versorgung geltenden Vordrucke (Muster 13, 14, 16 und 18). [2]Details zur Verordnung von Arznei-, Heil- und Hilfsmittel können der jeweils aktuellen Fassung der „Vereinbarung zwischen dem GKV-Spitzenverband und der KBV über die Vergabe von Betriebsstättennummern und einer Pseudo-Arzt-Nummer an Leistungserbringer der SAPV gemäß § 132 d Abs. 1 SGB V zur Verordnung von Arznei-, Heil- und Hilfsmitteln im Rahmen der SAPV“ (SAPV-BSNR-Vereinbarung) in der jeweils geltenden Fassung (Fassung vom Mai 2009 siehe Anlage 10) entnommen werden.

(2) [1]Die Vordrucke nach Abs. 1 dürfen im Rahmen der SAPV nur von Ärzten verwendet werden, die für ein PCT mit rechtsgültigem Vertrag nach § 132 d SGB V tätig sind. [2]Auf jeder Verordnung im Rahmen dieser Vereinbarung sind die Betriebsstättennummer und die Arztnummer anzugeben. [3]Diese wird von der Kassenärztliche Bundesvereinigung auf Antrag des Leistungserbringers vergeben. Details zur Antragsstellung der Betriebsstättennummer und der Arztnummer können der jeweils aktuellen Fassung der SAPV-BSNR-Vereinbarung (Fassung vom Mai 2009 siehe Anlage 10) entnommen werden.

## § 15 Anspruchsvoraussetzungen

Der Versicherte hat gemäß der Richtlinie des Gemeinsamen Bundesausschusses zur Verordnung von spezialisierter ambulanter Palliativversorgung und den gemeinsamen Empfehlungen der Spitzenverbände der Krankenkassen gemäß § 132 d Abs.2 SGB V für die spezialisierte Palliativversorgung Anspruch auf SAPV,

a) wenn er an einer nicht heilbaren, fortschreitenden und so weit fortgeschrittenen Erkrankung leidet, dass sich dadurch das Lebensende abzeichnet sowie

b) wenn er unter Berücksichtigung der in der Richtlinie des Gemeinsamen Bundesausschusses zur Verordnung von spezialisierter ambulanter Palliativversorgung und in den gemeinsamen Empfehlungen der Spitzenverbände der Krankenkassen gemäß § 132 d Abs.2 SGB V für die spezialisierte Palliativversorgung genannten Ziele eine besonders aufwändige Versorgung (§ 4 SAPV-RL) benötigt, die nach den medizinischen und pflegerischen Erfordernissen auch ambulant oder in Einrichtungen der Kinder- und Jugendhilfe, Behinderteneinrichtung oder in einer stationären Pflegeeinrichtung erbracht werden kann.

## § 16 Verordnung / Genehmigung / Beginn der SAPV

(1) Art, Umfang und Dauer der vom Leistungserbringer zu erbringenden Leistungen ergeben sich aus der von der Krankenkasse genehmigten Verordnung (Muster 63) der behandelnden Vertragsärztin/des behandelnden Vertragsarztes oder der Krankenhausärztin/des Krankenhausarztes.

(2) [1]Der Versicherte bestätigt mit Unterschrift, dass er mit SAPV einverstanden ist. [2]Kann die Unterschrift des Versicherten nicht erbracht werden, weil dieser bzw. sein Vertreter nicht in der Lage ist zu unterschreiben, bleibt das Unterschriftsfeld auf der Verordnung frei. [3]Der Leistungserbringer gibt zur Verordnung die Gründe hierfür an.

(3) [1]Die SAPV-RL ist für die Verordnung sowie für die Genehmigung durch die Krankenkassen maßgeblich. [2]Dort nicht aufgeführte Inhalte bedürfen einer nachvollziehbaren Begründung, damit sie von den Krankenkassen genehmigt werden können.

(4) [1]Der verordnende Arzt/ die verordnende Ärztin legt nach aktuellem Versorgungsbedarf die Dauer der Verordnung fest. [2]Der Krankenhausarzt / die Krankenhausärztin kann Leistungen in der Regel jedoch längstens für 7 Tage verordnen. [3]Sollte eine Versorgung für einen längeren Zeitraum als angegeben erforderlich sein, ist rechtzeitig eine Folgeverordnung bei der zuständigen Krankenkasse einzureichen.

(5) [1]Der PC-Arzt nimmt die Verordnung entgegen. [2]Er überzeugt sich davon, dass eine palliative Situation vorliegt und überprüft Indikation und Umfang der verordneten Leistung (Assessment). [3]Sollte sich herausstellen, dass der Patient für SAPV nicht geeignet ist oder die definierten Versorgungsziele auch durch die allgemeine ambulante Palliativversorgung sicher gestellt werden können, ist nach unverzüglicher Koordination einer geeigneten Anschlussversorgung die weitere Behandlung durch die spezialisierte ambulanten Palliativversorgung nicht zu beginnen bzw. zu beenden. [4]Leistungsausweitungen bedürfen der Unterschrift der/des verordnenden Ärztin/Arztes. [5]Bei Leistungseinschränkungen hat das PCT den/die verordnenden/verordnende Arzt/Ärztin zu informieren.

## § 17 Kostenübernahme

(1) [1]Die Krankenkasse übernimmt vom ärztlich festgelegten Leistungsbeginn an bis zu einer Entscheidung über die weitere Kostenübernahme, die Kosten für die verordneten und vom Leistungserbringer erbrachten Leistungen entsprechend der Vergütung gemäß diesem Vertrag, wenn die ordnungsgemäß ausgefüllte Verordnung spätestens am dritten der Ausstellung folgenden Arbeitstag der Krankenkasse vorliegt (vorläufige Kostenübernahme). [2]Sofern eine Verordnung verspätet eingereicht wird, werden die Kosten frühestens ab Eingangsdatum übernommen.

(2) [1]Ergibt die interne Prüfung durch die Krankenkasse, dass Zweifel an der Indikation zur Verordnung oder an deren Umfang bestehen, wird umgehend der MDK eingeschaltet. [2]Bei Unklarheiten ist ein Kontakt zwischen MDK und PCT wünschenswert.

(3) Über die endgültige Kostenübernahme oder (Teil-)Ablehnung informiert die Krankenkasse den Versicherten und den Leistungserbringer; bei (Teil-) Ablehnung auch den verordnenden Arzt.

## § 18 Dauer der Versorgung

(1) SAPV wird nach Bedarf intermittierend oder durchgängig erbracht, soweit das bestehende ambulante Versorgungsangebot, insbesondere die allgemeine Palliativversorgung, aus palliativmedizinischen und palliativpflegerischen Gründen nicht ausreicht.

(2) Bestehen nach Einschätzung des Leistungserbringers im individuellen Fall nicht oder nicht mehr die Voraussetzungen zur Erbringung von SAPV, wird der Patient in die passende Versorgungsstruktur übergeleitet.

(3) Bestehen nach Einschätzung des Leistungserbringers im individuellen Fall nicht mehr die Voraussetzungen zur Erbringung der verordneten Leistungskategorie von SAPV, wird der Patient durch eine dann niedrigere Leistungskategorie der SAPV versorgt.

(4) Handelt es sich um eine intermittierende Leistungserbringung, werden in Abhängigkeit der Vergütungsgestaltung nur diejenigen Tage als SAPV-Leistungstage vergütet, an denen SAPV erbracht wurde.

## § 19 Zuzahlungen

(1) [1]SAPV wird als Sachleistung zur Verfügung gestellt. [2]Zuzahlungen vom Versicherten im Rahmen der Vertragsleistungen dürfen weder gefordert noch angenommen werden.

(2) Zuzahlungen dürfen auch nicht verlangt werden, wenn die Verordnung bei der Krankenkasse verspätet eingereicht wird, da Kosten erst ab Eingang der Verordnung bei der Krankenkasse gemäß § 6 dieses Vertrages übernommen werden und deshalb die vorläufige Kostenzusage entfällt (§ 17 Abs. 1).

(3) Verstöße berechtigen zu Maßnahmen gemäß § 26.

## § 20 Vermittlungsverbot, Werbung und Einflussnahme

(1) Die Veranlassung einer Verordnung gegen Entgelt ist ein Vertragsverstoß.

(2) Die Weitergabe von angenommenen Verordnungen zur Versorgung anspruchsberechtigter Personen dieses Vertrages an Dritte gegen Entgelt oder zur Erlangung geldwerter Vorteile, ist ein Vertragsverstoß.

(3) Vermittlung im Sinne dieser Bestimmung ist auch die Weitergabe von in eigenem Namen angenommenen Aufträgen zur Versorgung anspruchsberechtigter Personen dieses Vertrages über das PCT hinaus an dritte Nicht-Kooperationspartner gegen Kostenerstattung.

(4) Der Leistungserbringer darf nicht mit der Leistungspflicht der Krankenkasse werben.

## § 21 Institutionskennzeichen

(1) Der Leistungserbringer verfügt gemäß § 293 SGB V über ein Institutionskennzeichen (IK), das er bei der Abrechnung mit den Krankenkassen verwendet.

(2) [1]Das IK ist bei der Sammel- und Verteilungsstelle IK der Arbeitsgemeinschaft Institutionskennzeichen (SVI), Alte Heerstr. 111, 53757 St. Augustin, zu beantragen. [2]Änderungen der unter dem IK gespeicherten Daten sind der SVI unverzüglich mitzuteilen. [3]Mitteilungen an die Krankenkassen oder ihre mit der Abrechnungsprüfung beauftragten Dienstleister werden nicht berücksichtigt.

(3) [1]Das gegenüber den Krankenkassen eingesetzte IK ist sowohl bei Vertragsabschluss als auch bei Änderungen mitzuteilen. [2]Abrechnungen mit den Krankenkassen erfolgen ausschließlich unter diesem IK. [3]Das IK ist in jeder Abrechnung anzugeben. [4]Abrechnungen ohne IK oder mit fehlerhaftem IK werden von den Krankenkassen abgewiesen. [5]Gleiches gilt für Abrechnungen mit einem der Krankenkasse unbekannten IK.

(4) [1]Die unter dem gegenüber den Krankenkassen verwendeten IK bei der SVI gespeicherten Angaben einschließlich der Bank- und Kontoverbindung sind für die Abrechnungsbegleichung durch die Krankenkassen verbindlich. [2]Andere Bank- und Kontoverbindungen werden von den Krankenkassen bei der Abrechnung nicht berücksichtigt.

## § 22 Leistungsnachweis

(1) Der Leistungsnachweis enthält folgende Angaben:

a) Name, Anschrift und Institutionskennzeichen des Leistungserbringers,
b) Name und Vorname, Versichertennummer, Anschrift und Geburtsdatum des Versicherten,
c) Positionsnummer, Art und Anzahl der erbrachten Leistungen (Positionsnummernverzeichnis der Richtlinien nach § 302 SGB V soweit festgelegt und relevant),
d) Angabe der beteiligten Kooperationspartner,
e) die nach der Vergütungsregelung notwendigen Angaben.

(2) Der leitende Arzt des PCT oder sein ärztlicher Vertreter bestätigt durch seine Unterschrift die Richtigkeit der Angaben im Leistungsnachweis.

## § 23 Abrechnung

(1) Für die Abrechung wird die Anwendung der Richtlinien der Spitzenverbände der Krankenkassen, jetzt Spitzenverband Bund, über Form und Inhalt des Abrechnungsverfahrens mit sonstigen Leistungserbringern nach § 302 Abs. 2 SGB V in der jeweils aktuellen Fassung angestrebt.

(2) Die Umsetzung der Abrechung nach § 302 Abs. 2 SGB V kann abhängig von den einzelnen Krankenkassen zu unterschiedlichen Zeitpunkten erfolgen.

(3) Die Rechnung muss folgende Mindestangaben enthalten:
   a) Name, Vorname, Geburtsdatum und Krankenversicherungsnummer des Leistungsbeziehers,
   b) Angabe des Versorgungszeitraumes (in Abhängigkeit von der Vergütungsgestaltung), des Versorgungsortes sowie der dafür anzusetzenden Vergütung gemäß **Anlage 7**,
   c) die Verordnungsblätter,
   d) den Leistungsnachweis als **Anlage 6**,
   e) Name, Anschrift, Unterschrift und Institutionskennzeichen des Leistungserbringers,
   f) Rechnungsbetrag,
   g) Betriebsstättennummer,
   h) Leistungserbringergruppenschlüssel (LEGS).

(4) [1]Überträgt der Leistungserbringer die Abrechnung einerAbrechnungsstelle, so hat der Leistungserbringer die zuständige Krankenkasse unverzüglich schriftlich hierüber zu informieren. [2]Der Beginn und das Ende des Auftragsverhältnisses, der Name der beauftragten Abrechnungsstelle und das Institutionskennzeichen, unter dem die Abrechnungsstelle die Rechnungslegung vornimmt, ist mitzuteilen.

(5) [1]Hat der Leistungserbringer dem Abrechnungszentrum eine Inkasso-Vollmacht erteilt, erfolgt die Zahlung an das Abrechnungszentrum für die Krankenkassen mit schuldbefreiender Wirkung. [2]Wird dem Abrechnungszentrum die Inkasso-Vollmacht entzogen, ist der Leistungserbringer für die Einhaltung des Entzuges der Vollmacht verantwortlich.

(6) [1]Sofern die Rechnungslegung einer Abrechnungsstelle übertragen werden soll, ist der Auftragnehmer unter besonderer Berücksichtigung der von ihm getroffenen technischen und organisatorischen Maßnahmen zur Sicherstellung der Maßgaben dieses Vertrages und des § 6 Abs 1 BDSG durch den Leistungserbringer auszuwählen. [2]Die getroffene Vereinbarung über Datenschutz und Datensicherung beim Auftragnehmer (Abrechnungsstelle) ist den Krankenkassen bzw. deren Verband vorzulegen.

(7) Der Leistungserbringer ist für die Einhaltung der gesetzlichen und vertraglichen Regelungen durch das Abrechnungszentrum verantwortlich.

(8) [1]Die Rechnung erfolgt versichertenbezogen in der Regel einmal im Monat und ist bei den von den Krankenkassen benannten Daten- und Papierannahmestellen einzureichen. [2]Die Krankenkassen sind zur Rechnungsbegleichung nur verpflichtet, sofern eine versicherungs-, leistungs- und vertragsrechtliche Anspruchsberechtigung gegeben ist.

(9) [1]Werden Nachberechnungen gestellt, sind der Nachberechnung Kopien der Erstrechnung und der Verordnung beizulegen. [2]Darauf ist "Nachberechnung" zu vermerken.

(10) [1]Maßgeblich für die Berechnung des Zahlungsziels ist der Tag, an dem alle zu einem Abrechnungsfall gehörenden Unterlagen bei der Krankenkasse vorliegen. [2]Zusammengehörige Rechnungsunterlagen müssen in unmittelbarem zeitlichem Zusammenhang angeliefert werden. [3]Die Krankenkasse behält sich vor, nicht korrekt gestellte Rechnungen unbezahlt abzuweisen. In diesem Fall beginnt die Zahlungsfrist ab Eingang einer korrekten Rechnung erneut zu laufen.

(11) [1]Die Bezahlung der Rechnung erfolgt spätestens innerhalb von 21 Kalendertagen nach Eingang der Rechnung bei der zuständigen Krankenkasse bzw. bei der von ihr benannten Abrechnungsstelle. [2]Bei Zahlung durch Überweisung gilt die Frist als gewahrt, wenn der Auftrag innerhalb dieser Frist dem Geldinstitut erteilt wurde.

(12) [1]Von den SAPV-Leistungserbringern und deren Kooperationspartnern dürfen nicht parallel die zur gleichen Einsatzzeit erbrachten Leistungen abgerechnet werden. [2]Die zusätzliche Abrechnung von deckungsgleichen Leistungen nach SGB V (z.B. §§ 27, 37, 37a, 43 Abs. 2 SGB V) sowie SGB XI (z.B. §§ 36, 39) ist demnach ausgeschlossen.

(13) Nicht erbrachte Leistungen können nicht abgerechnet werden.

(14) Bis zur Einführung des DTA gilt die übliche Papierrechnung. Die Rechnung muss dabei die Mindestangaben des Absatzes 3 dieses § enthalten.

## § 24 Statistik

(1) Der Leistungserbringer ist verpflichtet, an Auswertungen mitzuwirken.

(2) [1]Die Auswertungen beziehen sich in der Regel auf die in der **Anlage 9** aufgezählten Inhalte. [2]Die Meldung hat bis spätestens zum 30. April für das abgelaufene Kalenderjahr an die von den Krankenkassen zu bestimmende Stelle zu erfolgen.

## § 25 Datenschutz, Schweigepflicht

(1) [1]Der Leistungserbringer ist verpflichtet, die Bestimmungen über den Schutz der Sozialdaten (SGB X 2. Kapitel) zu beachten, insbesondere personenbezogene Daten nur zur Erfüllung der sich aus diesem Vertrag ergebenden Aufgaben zu verarbeiten, bekannt zu geben, zugänglich zu machen oder sonst zu nutzen. [2]Ausnahmen bedürfen der schriftlichen Einwilligung des Patienten oder seines Vertreters.

(2) [1]Der Leistungserbringer unterliegt hinsichtlich der Person des Versicherten und dessen Krankheiten der Schweigepflicht. [2]Ausgenommen hiervon sind Angaben gegenüber der leistungspflichtigen Krankenkasse und/oder dem MDK, soweit sie zur Erfüllung der gesetzlichen Aufgaben der Krankenkasse erforderlich sind.

(3) [1]Der Leistungserbringer hat die im PCT tätigen Mitarbeiter und die Kooperationspartner zur Beachtung der Datenschutzbestimmungen und der Schweigepflicht zu verpflichten. [2]Die Verpflichtung ist zu dokumentieren.

## § 26 Maßnahmen bei Vertragsverstößen des Leistungserbringers

(1) [1]Vertragsverstöße sind dem Leistungserbringer durch die Verbände bzw. den jeweiligen Verband der Krankenkassen in schriftlicher Form mitzuteilen. [2]Hierbei ist dem Leistungserbringer Gelegenheit zur Stellungnahme innerhalb von 28 Tagen zu geben (Anhörung).

(2) [1]Die Verbände bzw. der jeweilige Verband der Krankenkassen entscheiden nach Prüfung der Stellungnahme, welche geeigneten Maßnahmen festzulegen sind. [2]Diese sind:
   a) Abmahnung,
   b) Vertragsstrafe bis zu 10% des Jahresumsatzes des PCT.

(3) Über die Entscheidung ist dem Vertragspartner ein schriftlicher Bescheid zu erteilen.

## § 27 Vergütung

(1) Die Vergütung richtet sich nach **Anlage 7**.

(2) Eine im Rahmen der SAPV vergütete Leistung kann nicht anderweitig in Rechnung gestellt werden.

## § 28 Inkrafttreten / Kündigung

(1) Diese Vereinbarung tritt am ... 2009 in Kraft.

(2) [1]Der Vertrag kann von jedem Vertragspartner mit einer Frist von 3 Monaten zum Ende eines Kalenderjahres gekündigt werden (ordentliche Kündigung). [2]Eine teilweise Kündigung ist nur zulässig, soweit es in einzelnen Anlagen vorgesehen ist.[3]Die Kündigung bedarf der Schriftform.

(3) Die Kündigung durch einen Vertragspartner auf Krankenkassenseite berührt nicht die Gültigkeit des Vertrages zwischen den verbleibenden Vertragsbeteiligten.

(4) [1]Eine außerordentliche Kündigung kann ohne Einhaltung von Kündigungsfristen erfolgen, wenn der Leistungserbringer seine vertraglichen Pflichten gegenüber einer Krankenkasse derart gröblich verletzt, dass ein Festhalten an diesem Vertrag nicht mehr zumutbar ist. [2]Eine gröbliche Pflichtverletzung in diesem Sinn liegt insbesondere vor:

   a) bei fortgesetzter Nichterfüllung wesentlicher fachlicher Voraussetzungen,

   b) bei wiederholter Abrechnung von nicht oder teilweise nicht erbrachten Leistungen,

   c) bei einer Pflichtverletzung durch den Leistungserbringer, bei der ein Versicherter zu Schaden kommt,

   d) bei einer Forderung bzw. einer Annahme von unzulässigen Zahlungen oder Zuzahlungen vom Versicherten für von den Krankenkassen genehmigten Leistungen,

   e) bei einer Zahlung oder einer Annahme von Vergütungen oder Provisionen für Zuweisungen von Versicherten oder Vermittlung von Aufträgen,

f) bei wiederholten oder schweren Verstößen gegen Datenschutzbestimmungen oder sonstige Vertragspflichten.

(5) Einvernehmlich vereinbarte Änderungen dieses Vertrages in schriftlicher Form sind möglich, ohne dass es einer Kündigung bedarf.

## § 29 Salvatorische Klausel

(1) Sollten einzelne Bestimmungen dieses Vertrages unwirksam oder undurchführbar sein oder nach Vertragsschluss unwirksam oder undurchführbar werden, bleibt davon die Wirksamkeit des Vertrages im Übrigen unberührt.

(2) An die Stelle der unwirksamen oder undurchführbaren Bestimmung soll eine Regelung treten, die der Zielsetzung zur Erbringung von SAPV am nächsten kommt.

**Anlagenübersicht:**

Anlage 1 Richtlinie des Gemeinsamen Bundesausschuss nach § 92 Abs. 1
Anlage 2 Gemeinsame Empfehlungen der Spitzenverbände der Krankenkassen
Anlage 3 Versorgungsgebiet
Anlage 4 Vorzulegende Unterlagen bei Zulassungsantrag
Anlage 5 Evaluation
Anlage 6 Formular für den Leistungsnachweis
Anlage 7 Vergütungsvereinbarung
Anlage 8 Übergangsregelung
Anlage 9 Statistik
Anlage 10 SAPV-BSNR-Vereinbarung

Ort, Datum

**Leistungserbringer**

**Für die Krankenkassen**
AOK Bayern - Die
Gesundheitskasse, Zentrale

BKK Landesverband Bayern

Knappschaft
-Regionaldirektion München-

Vereinigte IKK in Bayern

Funktioneller Landesverband der
Landwirtschaftlichen
Krankenkassen und Pflegekassen
in Bayern (LdL/LdLP)

Verband der Ersatzkassen e.V.
Der Leiter der Landesvertretung
Bayern

**Anlage 1 Richtlinie des Gemeinsamen Bundesausschusses**

**Richtlinie des Gemeinsamen Bundesausschusses zur Verordnung von spezialisierter ambulanter Palliativversorgung (Spezialisierte Ambulante Palliativversorgungs-Richtlinie / SAPV-RL)**
vom 20. Dezember 2007,
veröffentlicht im Bundesanzeiger 2008, S. 911

zuletzt geändert am 15. April 2010,
veröffentlicht im Bundesanzeiger, S. 2 190,
in Kraft getreten am 25. Juni 2010

## § 1 Grundlagen und Ziele

(1) [1]Die spezialisierte ambulante Palliativversorgung gemäß § 37b SGB V (SAPV) dient dem Ziel, die Lebensqualität und die Selbstbestimmung schwerstkranker Menschen zu erhalten, zu fördern und zu verbessern und ihnen ein menschenwürdiges Leben bis zum Tod in ihrer vertrauten häuslichen oder familiären Umgebung zu ermöglichen. [2]Im Vordergrund steht anstelle eines kurativen Ansatzes die medizinisch-pflegerische Zielsetzung, Symptome und Leiden einzelfallgerecht zu lindern.

(2) [1]SAPV kann im Haushalt des schwerstkranken Menschen oder seiner Familie oder in stationären Pflegeeinrichtungen (§72 Abs. 1 des Elften Buches Sozialgesetzbuch – SGB XI) erbracht werden. [2]Darüber hinaus kann SAPV auch erbracht werden
– in Einrichtungen der Eingliederungshilfe für behinderte Menschen im Sinne von §55 SGB XII und der Kinder- und Jugendhilfe im Sinne von §34 SGB VIII,
– an weiteren Orten, an denen
- sich der schwerstkranke Mensch in vertrauter häuslicher oder familiärer Umgebung dauerhaft aufhält und
- diese Versorgung zuverlässig erbracht werden kann
wenn und soweit nicht andere Leistungsträger zur Leistung verpflichtet sind.

(3) In stationären Hospizen besteht ein Anspruch auf die Teilleistung der erforderlichen ärztlichen Versorgung im Rahmen der SAPV, wenn die ärztliche Versorgung im Rahmen der vertragsärztlichen Versorgung aufgrund des besonders aufwändigen Versorgungsbedarfs (siehe §4) nicht ausreicht.

(4) Den besonderen Belangen von Kindern ist Rechnung zu tragen.

(5) [1]Die individuellen Bedürfnisse und Wünsche der Patientin oder des Patienten sowie die Belange ihrer oder seiner vertrauten Personen stehen im Mittelpunkt der Versorgung. [2]Der Patientenwille, der auch durch Patientenverfügungen zum Ausdruck kommen kann, ist zu beachten.

(6) [1]Die SAPV ergänzt das bestehende Versorgungsangebot, insbesondere das der Vertragsärzte, Krankenhäuser und Pflegedienste. [2]Sie kann als alleinige Beratungsleistung, additiv unterstützende Teilversorgung oder vollständige Patientenbetreuung erbracht werden. [3]Andere Sozialleistungsansprüche bleiben unberührt.

## § 2 Anspruchsvoraussetzungen

Versicherte haben Anspruch auf SAPV, wenn
– sie an einer nicht heilbaren, fortschreitenden und so weit fortgeschrittenen Erkrankung leiden, dass dadurch ihre Lebenserwartung begrenzt ist (§ 3) und

– sie unter Berücksichtigung der in § 1 genannten Ziele eine besonders aufwändige Versorgung (§ 4) benötigen, die nach den medizinischen und pflegerischen Erfordernissen auch ambulant oder an den in §1 Abs. 2 und 3 genannten Orten erbracht werden kann.

## § 3 Anforderungen an die Erkrankungen

(1) Eine Erkrankung ist nicht heilbar, wenn nach dem allgemein anerkannten Stand der medizinischen Erkenntnisse Behandlungsmaßnahmen nicht zur Beseitigung dieser Erkrankung führen können.

(2) Sie ist fortschreitend, wenn ihr Verlauf trotz medizinischer Maßnahmen nach dem allgemein anerkannten Stand der medizinischen Erkenntnisse nicht nachhaltig aufgehalten werden kann.

(3) [1]Eine Erkrankung ist weit fortgeschritten, wenn die Verbesserung von Symptomatik und Lebensqualität sowie die psychosoziale Betreuung im Vordergrund der Versorgung stehen und nach begründeter Einschätzung der verordnenden Ärztin oder des verordnenden Arztes die Lebenserwartung auf Tage, Wochen oder Monate gesunken ist. [2]Insbesondere bei Kindern sind die Voraussetzungen für die SAPV als Krisenintervention auch bei einer länger prognostizierten Lebenserwartung erfüllt.

## § 4 Besonders aufwändige Versorgung

[1]Bedarf nach einer besonders aufwändigen Versorgung besteht, soweit die anderweitigen ambulanten Versorgungsformen sowie ggf. die Leistungen des ambulanten Hospizdienstes nicht oder nur unter besonderer Koordination ausreichen würden, um die Ziele nach § 1 Abs. 1 zu erreichen. [2]Anhaltspunkt dafür ist das Vorliegen eines komplexen Symptomgeschehens, dessen Behandlung spezifische palliativmedizinische und / oder palliativpflegerische Kenntnisse und Erfahrungen sowie ein interdisziplinär, insbesondere zwischen Ärzten und Pflegekräften in besonderem Maße abgestimmtes Konzept voraussetzt. [3]Ein Symptomgeschehen ist in der Regel komplex, wenn mindestens eines der nachstehenden Kriterien erfüllt ist:

- ausgeprägte Schmerzsymptomatik
- ausgeprägte neurologische / psychiatrische / psychische Symptomatik
- ausgeprägte respiratorische / kardiale Symptomatik
- ausgeprägte gastrointestinale Symptomatik
- ausgeprägte ulzerierende / exulzerierende Wunden oder Tumore
- ausgeprägte urogenitale Symptomatik

## § 5 Inhalt und Umfang der spezialisierten ambulanten Palliativversorgung

(1) [1]Die SAPV umfasst je nach Bedarf alle Leistungen der ambulanten Krankenbehandlung soweit diese erforderlich sind, um die in § 1 Abs. 1 genannten Ziele zu erreichen. [2]Sie umfasst zusätzlich die im Einzelfall erforderliche Koordination der diagnostischen, therapeutischen und pflegerischen Teilleistungen sowie die Beratung, Anleitung und Begleitung der verordnenden oder behandelnden Ärztin oder des verordnenden oder behandelnden Arztes sowie der sonstigen an der allgemeinen Versorgung beteiligten Leistungserbringer, der Patienten und ihrer Angehörigen durch Leistungserbringer nach § 132d SGB V.

(2) [1]SAPV wird ausschließlich von Leistungserbringern nach §132d SGB V erbracht, die in einer interdisziplinären Versorgungsstruktur, bestehend insbesondere aus qualifizierten Ärzten und Pflegefachkräften unter Beteiligung der ambulanten Hospizdienste und ggf. der stationären Hospize, organisiert sind. [2]Sie wird nach Bedarf intermittierend oder durchgängig erbracht, soweit das bestehende ambulante Versorgungsangebot (§ 1 Abs. 4), insbesondere die allgemeine Palliativversor-gung nicht ausreicht, um die Ziele nach § 1 Abs. 1 zu erreichen. [3]Sie kann dem jeweiligen aktuellen Versorgungsbedarf entsprechend als

- Beratungsleistung,
- Koordination der Versorgung,
- additiv unterstützende Teilversorgung,
- vollständige Versorgung

erbracht werden. [4]Die Leistungen müssen ausreichend und zweckmäßig sein, dürfen das Maß des Notwendigen nicht überschreiten und sind wirtschaftlich zu erbringen.

(3) Inhalte der SAPV sind insbesondere:
- Koordination der spezialisierten palliativmedizinischen und palliativpflegerischen Versorgung unter Einbeziehung weiterer Berufsgruppen und von Hospizdiensten im Rahmen einer multiprofessionellen Zusammenarbeit
- Symptomlinderung durch Anwendung von Medikamenten oder anderen Maßnahmen
- apparative palliativmedizinische Behandlungsmaßnahmen (z. B. Medikamentenpumpe)
- palliativmedizinische Maßnahmen, die nach ihrer Art, Schwere oder Komplexität eine Kompetenz erfordern, die der einer Ärztin oder eines Arztes mit Zusatzweiterbildung Palliativmedizin entspricht
- spezialisierte palliativpflegerische Leistungen, die nach ihrer Art, Schwere oder Komplexität eine Kompetenz erfordern, die der einer Pflegefachkraft mit einer curricularen Weiterbildung zu Palliative Care entspricht
- Führung eines individuellen Behandlungsplans, vorbeugendes Krisenmanagement, Bedarfsinterventionen
- Ruf-, Notfall- und Kriseninterventionsbereitschaft rund um die Uhr für die im Rahmen der SAPV betreuten Patienten zur Sicherstellung der im Rahmen der SAPV erforderlichen Maßnahmen
- Beratung, Anleitung und Begleitung der Patienten und ihrer Angehörigen zur palliativen Versorgung einschließlich Unterstützung beim Umgang mit Sterben und Tod
- spezialisierte Beratung der betreuenden Leistungserbringer der Primärversorgung
- psychosoziale Unterstützung im Umgang mit schweren Erkrankungen in enger Zusammenarbeit z. B. mit Seelsorge, Sozialarbeit und ambulanten Hospizdiensten
- Organisation regelmäßiger Fallbesprechungen
- Dokumentieren und Evaluieren der wesentlichen Maßnahmen im Rahmen der SAPV

## § 6 Zusammenarbeit der Leistungserbringer

(1) [1]Im Rahmen der SAPV ist zu gewährleisten, dass die an der Versorgung beteiligten Leistungserbringer die erforderlichen Maßnahmen aufeinander abgestimmt und bedarfsgerecht erbringen; die diesbezügliche Koordination ist sicherzustellen. [2]Hierüber sind verbindliche Kooperationsvereinbarungen schriftlich oder mündlich zu treffen. [3]Kooperationspartner ist auch der ambulante Hospizdienst, der auf Wunsch der Patientin oder des Patienten an der Versorgung beteiligt wird. [4]Bei Bedarf und entsprechender Qualifikation kann die dauerbehandelnde Ärztin oder der dauerbehandelnde Arzt im Einzelfall Kooperationspartnerin oder Kooperationspartner werden. [5]Das Nähere regeln die Verträge nach § 132d SGB V.

(2) Die vorhandenen Versorgungsstrukturen sind zu beachten.

(3) Es ist zu gewährleisten, dass zwischen den an der Patientenversorgung beteiligten Leistungserbringern zeitnah alle notwendigen Informationen über die vorhergehende Behandlung unter Berücksichtigung datenschutzrechtlicher Regelungen ausgetauscht werden.

(4) Bei der SAPV ist der ärztlich und pflegerisch erforderliche Entscheidungsspielraum für die Anpassung der Palliativversorgung an die Besonderheiten des Einzelfalls zu berücksichtigen.

(5) [1]Für die notwendigen koordinativen Maßnahmen ist vernetztes Arbeiten innerhalb der gewachsenen Strukturen der Palliativversorgung unabdingbar. [2]Dieses ist unter Berück-

sichtigung medizinischer, pflegerischer, physiotherapeutischer, psychologischer, psychosozialer und spiritueller Anforderungen zur lückenlosen Versorgung über die Sektorengrenzen hinweg zu fördern und auszubauen.

## § 7 Verordnung von SAPV

(1) [1]SAPV wird von der behandelnden Vertragsärztin oder von dem behandelnden Vertragsarzt nach Maßgabe dieser Richtlinie verordnet. [2]Satz 1 gilt für die Behandlung durch die Krankenhausärztin oder den Krankenhausarzt bei einer oder einem von ihr oder ihm ambulant versorgten Patientin oder Patienten entsprechend. [3]Hält eine Krankenhausärztin oder ein Krankenhausarzt die Entlassung einer Patientin oder eines Patienten für möglich und ist aus ihrer oder seiner Sicht SAPV erforderlich, kann die Krankenhausärztin oder der Krankenhausarzt die Verordnung ausstellen, in der Regel jedoch längstens für 7 Tage.

(2) Die ärztliche Verordnung erfolgt auf einem zu vereinbarenden Vordruck, der der Leistungserbringung nach dem jeweiligen aktuellen Versorgungsbedarf (§ 5 Abs. 2) Rechnung zu tragen hat und Angaben zur Dauer der Verordnung enthält.

## § 8 Prüfung der Leistungsansprüche durch die Krankenkasse

[1]Die Krankenkasse übernimmt bis zu einer Entscheidung über die weitere Leistungserbringung die Kosten für die verordneten und von den Leistungserbringern nach § 132d SGB V erbrachten Leistungen entsprechend der vereinbarten Vergütung nach § 132d SGB V, wenn die Verordnung gemäß § 7 Abs. 2 spätestens an dem dritten der Ausstellung folgenden Arbeitstag der Krankenkasse vorgelegt wird. [2]Das Nähere regeln die Vertragspartner nach § 132d SGB V.

**Anlage 2 Gemeinsamen Empfehlungen der Spitzenverbände der Krankenkassen gemäß § 132 d Abs. 2 SGB V**

# Gemeinsame Empfehlungen

des AOK-Bundesverbandes, Bonn,
des BKK Bundesverbandes, Essen,
des Bundesverbandes der landwirtschaftlichen Krankenkassen, Kassel,
des IKK-Bundesverbandes, Bergisch Gladbach,
der Knappschaft, Bochum,
des Verbandes der Angestellten-Krankenkassen e.V., Siegburg,
des AEV Arbeiter-Ersatzkassen-Verband e.V., Siegburg

# nach § 132d Abs.2 SGB V für die spezialisierte ambulante Palliativversorgung

**vom 23.06.2008**

unter Beteiligung
des Arbeiterwohlfahrt Bundesverbandes e.V., Berlin,
des Arbeitgeber- und BerufsVerbandes Privater Pflege e.V., Hannover,
der Bundesarbeitsgemeinschaft Hauskrankenpflege e.V., Berlin,
der Bundesarbeitsgemeinschaft der PatientInnenstellen, München,
des Bundesverbandes Ambulante Dienste und Stationäre Einrichtungen e.V., Essen,
der Bundesinitiative Ambulante Psychiatrische Pflege e.V., Berlin,
des Bundesverbandes Deutscher Privatkliniken e.V. Berlin,
des Bundesverbandes Häusliche Kinderkrankenpflege e.V., Köln,
des Bundesverbandes Kinderhospiz e.V., Freiburg,
des Bundesverbandes privater Anbieter sozialer Dienste e.V., Berlin,
der Deutschen Arbeitsgemeinschaft Selbsthilfegruppen e.V., Gießen,
des Deutschen Bundesverbandes für Pflegeberufe, Berlin,
des Deutschen Caritasverbandes e.V., Freiburg,
der Deutschen Gesellschaft für Palliativmedizin e.V., Berlin,
der Deutschen Hospiz Stiftung, Dortmund,
des Deutschen Hospiz- und PalliativVerbandes e.V., Berlin,
des Deutschen Kinderhospizvereines e.V., Olpe,
der Deutschen Krankenhaus-Gesellschaft, Berlin,
des Deutschen Paritätischen Wohlfahrtsverbandes, Gesamtverband e.V., Berlin,
des Deutschen Pflegerates, Berlin,
des Deutschen Roten Kreuzes, Generalsekretariat, Berlin,
des Diakonischen Werkes der EKD e.V., Berlin,
der Kassenärztlichen Bundesvereinigung, Berlin,
des Sozialverbandes VdK Deutschland e.V., Berlin,
des Verbandes Deutscher Alten- und Behinderten Hilfe e.V., Essen,
der Verbraucherzentrale Bundesverband e.V., Berlin,
die Zentralwohlfahrtsstelle der deutschen Juden in Deutschland e.V., Frankfurt am Main

## 1. Zielsetzung

1.1 Die spezialisierte ambulante Palliativversorgung (SAPV) dient dem Ziel, die Lebensqualität und die Selbstbestimmung schwerstkranker Menschen zu erhalten, zu fördern und zu verbessern und ihnen ein menschenwürdiges Leben bis zum Tod in ihrer vertrauten häuslichen Umgebung oder in einer stationären Pflegeeinrichtung zu ermöglichen.

1.2 Die SAPV ist fachlich kompetent nach den allgemein anerkannten medizinischen und pflegewissenschaftlichen Erkenntnissen bedarfsgerecht und wirtschaftlich zu erbringen. Die individuellen Bedürfnisse und Wünsche der Patientin oder des Patienten sowie die Belange ihrer oder seiner vertrauten Personen stehen im Mittelpunkt der Versorgung.

1.3 Die Spitzenverbände der Krankenkassen werden die Erfahrungen mit der Umsetzung dieser Empfehlungen fortlaufend auswerten und diese erforderlichenfalls weiterentwickeln.

**2. Zulassungsvoraussetzungen**

2.1 SAPV wird von Leistungserbringern erbracht, mit denen die Krankenkasse zur Sicherung einer bedarfsgerechten Versorgung einen Vertrag geschlossen hat. Sie wird intermittierend oder durchgängig nach Bedarf als

- Beratungsleistung,
- Koordination der Versorgung,
- additiv unterstützende Teilversorgung,
- vollständige Versorgung

erbracht. Den besonderen Belangen von Kindern und Jugendlichen ist Rechnung zu tragen.

2.2 Bei der Entwicklung einer bedarfsgerechten Versorgung sind die bereits bestehenden Strukturen so weit wie möglich einzubeziehen. Auf die bereits tätigen Leistungserbringer ist zurückzugreifen, soweit sie die erforderlichen Anforderungen erfüllen. Verträge sind nur in dem Umfang abzuschließen, wie sie für eine bedarfsgerechte Versorgung erforderlich sind. Ein Anspruch auf Vertragsabschluss besteht nicht.

2.3 Eine bedarfsgerechte Versorgung mit SAPV ist insbesondere dann gegeben, wenn sie wohnortnah ausgerichtet ist und die Palliativpatienten, die einen besonderen Versorgungsbedarf haben, der durch die allgemeine Palliativversorgung nicht gewährleistet werden kann, ausreichend und zweckmäßig mit der Leistung der SAPV versorgt werden können. Die bedarfsgerechte Versorgung zeigt sich auch darin, dass es mit der Leistung der SAPV mehr Menschen als bisher ermöglicht wird, in ihrer vertrauten häuslichen Umgebung oder in stationären Pflegeeinrichtungen ein menschenwürdiges Leben bis zum Tod zu führen.

2.4 Der Versorgungsbedarf kann in einzelnen Regionen unterschiedlich sein. Indikatoren für die Bedarfslage können insbesondere sein,

- die regionale Siedlungsstruktur,
- die Altersstruktur,
- epidemiologisch relevante Erkrankungen sowie
- die demografische Entwicklung.

So ist zwischen Regionen geringer, mittlerer und hoher Bevölkerungsdichte zu unterscheiden. Die Unterscheidung ist ggf. nicht allein auf der Ebene der Bundesländer

durchzuführen, sondern kann bis auf die Ebene der Kreise und kreisfreien Städte vollzogen werden, um dem erheblichen Unterschied in der Bevölkerungsdichte auch innerhalb der Bundesländer gerecht zu werden. Nach derzeit vorliegenden Schätzungen haben bis zu 10 Prozent aller Sterbenden einen solchen besonderen Versorgungsbedarf, der im Rahmen der SAPV abzudecken ist.

**3. Inhalt und Umfang der Leistungen**

3.1 Inhalt und Umfang der zu erbringenden SAPV-Leistungen ergeben sich aus der gemäß der SAPV-Richtlinie nach § 37b SGB V in Verbindung mit § 92 Abs.1 Satz 2 Nr. 14 SGB V ausgestellten und genehmigten Verordnung. Die Krankenkasse übernimmt bis zu einer Entscheidung über die weitere Leistungserbringung die Kosten für die verordneten und von den Leistungserbringern nach § 132d SGB V erbrachten Leistungen entsprechend der vereinbarten Vergütung nach § 132d SGB V, wenn die Verordnung gemäß § 7 Abs. 2 der SAPV-Richtlinie spätestens an dem dritten der Ausstellung folgenden Arbeitstag der Krankenkasse vorgelegt wird.

3.2 Die Leistungen müssen ausreichend und zweckmäßig sein, dürfen das Maß des Notwendigen nicht überschreiten und sind wirtschaftlich zu erbringen. Soweit die allgemeine Palliativversorgung ausreichend ist, darf SAPV nicht erbracht werden.

3.3 Im Falle der Besserung bzw. einer Stabilisierung im Krankheitsverlauf ist der Versorgungsumfang der SAPV so weit wie möglich zu reduzieren und eine Weiterversorgung im Rahmen der allgemeinen Palliativversorgung anzustreben.

3.4 In den Vereinbarungen über die SAPV sind Regelungen zu treffen, die sicherstellen, dass für den Palliativpatienten eine reibungslose Versorgung mit Arznei- Heil- und Hilfsmitteln auch weiterhin gewährleistet ist.

**4. Organisatorische Voraussetzungen**

4.1 Die Leistungen der SAPV werden durch spezialisierte Leistungserbringer erbracht. Spezialisierte Leistungserbringer sind nach Nr. 5.2 qualifizierte Ärztinnen oder Ärzte und nach Nr. 5.3 qualifizierte Pflegefachkräfte, die orientiert an der Konzeption eines Palliativ-Care-Teams fachübergreifend ggf. auch in Kooperation mit anderen Professionen (s. Nr. 5.4) eng zusammenarbeiten. Sie erfüllen ergänzend besondere sächliche Voraussetzungen nach Nr. 4.4 und Nr. 4.5, die für eine spezialisierte palliativmedizinische und palliativpflegerische Versorgung erforderlich sind. Eine psychosoziale Unterstützung ist in enger Zusammenarbeit z.B. mit ambulanten Hospizdiensten nach § 39a Abs. 2 SGB V, Seelsorge und Sozialarbeit zu gewährleisten.

4.2 Die spezialisierten Leistungserbringer sind Teil einer multiprofessionell vernetzten Versorgungsstruktur im regionalen Gesundheits- und Sozialsystem. Sie arbeiten mit den an der Versorgung beteiligten Leistungserbringern, wie Hausärzten und ambulanten Hospizdiensten gemäß § 39a Abs. 2 SGB V eng zusammen (integrativer Ansatz). Mit den regelhaft an der Versorgung beteiligten Leistungserbringern sind Kooperationsvereinbarungen schriftlich abzuschließen und der Krankenkasse vorzulegen.

4.3 Die spezialisierten Leistungserbringer arbeiten nach einem verbindlichen, strukturierten und schriftlich dargelegten Konzept, in dem der inhaltliche und organisatorische Rahmen der Leistungserbringung (inkl. der personellen und

sächlichen Ausstattung) sowie die Einbindung in die regionale Versorgungsstruktur beschrieben sind. Das Konzept ist der Krankenkasse vorzulegen.

4.4 Die spezialisierten Leistungserbringer haben als Mindestanforderung an die sächliche Ausstattung Folgendes vorzuhalten bzw. sicherzustellen:

• eine geeignete, aktuell geführte und für die an der Versorgung Beteiligten jederzeit zugängliche Patientendokumentation
• Arzneimittel (inkl. BtM) für die Notfall/Krisenintervention
• Arzt-/Pflegekoffer/Bereitschaftstasche (Berücksichtigung der Kompatibilität der Verbrauchsmaterialien zu Medizinprodukten unterschiedlicher Hersteller, z.B. bei Portsystemen oder Infusionspumpen)
• eine geeignete administrative Infrastruktur, z. B. Büro, Kommunikationstechnik.

4.5 Die spezialisierten Leistungserbringer müssen über eine eigenständige Adresse und geeignete Räumlichkeiten für

• die Beratung von Patienten und Angehörigen
• Teamsitzungen und Besprechungen
• die Lagerhaltung von eigenen Medikamenten für Notfall- / Krisenintervention und Hilfsmitteln

verfügen. Sofern eine Aufbewahrung von Medikamenten erfolgt, die unter das Betäubungsmittelgesetz (BtM-G) fallen, ist ein BtM-Schrank (§ 15 BtM-G) erforderlich.
.

**5. Personelle Anforderungen**

5.1 Die spezialisierten Leistungserbringer stellen sicher, dass das für die spezialisierte ambulante Palliativversorgung erforderliche und geeignete Personal zur Verfügung steht. Dabei ist zu berücksichtigen, dass durch den spezialisierten Leistungserbringer eine tägliche telefonische Rund-um-die-Uhr-Erreichbarkeit für die Patienten, deren Angehörigen und die an der Versorgung Beteiligten sicherzustellen ist. Die ständige Verfügbarkeit mindestens einer Ärztin/eines Arztes und/oder einer Pflegefachkraft ist zu gewährleisten. Die Verfügbarkeit schließt notwendige Hausbesuche ein.

5.2 Die nach Nr. 4.1 tätigen Ärztinnen und Ärzte verfügen über

• eine anerkannte Zusatzweiterbildung Palliativmedizin nach der aktuell gültigen Weiterbildung der jeweiligen Landesärztekammer (grds. 160 Std. Weiterbildung) und
• Erfahrung aus der ambulanten palliativen Behandlung von mindestens 75 Palliativpatienten,
z. B. in der häuslichen Umgebung oder in einem stationären Hospiz, innerhalb der letzten drei Jahre oder aus einer mindestens einjährigen klinischen palliativmedizinischen Tätigkeit in einer Palliativabteilung in einem Krankenhaus innerhalb der letzten drei Jahre.

5.3 Die nach Nr. 4.1 tätigen Pflegefachkräfte verfügen über:

• die Erlaubnis zur Führung einer der Berufsbezeichnungen Gesundheits- und Krankenpflegerin / Gesundheits- und Krankenpfleger, Gesundheits- und Kinderkrankenpflegerin / Gesundheits- und Kinderkrankenpfleger oder Altenpflegerin / Altenpfleger
(dreijährige Ausbildung aufgrund einer landesrechtlichen Regelung) entsprechend den gesetzlichen Bestimmungen in der jeweils gültigen Fassung und
• den Abschluss einer Palliative-Care-Weiterbildungsmaßnahme im Umfang von mindestens 160 Stunden
und
• Erfahrung durch mindestens eine zweijährige praktische Tätigkeit als Pflegefachkraft in der Betreuung von Palliativpatienten in den letzten drei Jahren; davon mindestens sechsmonatige Mitarbeit in einer spezialisierten Einrichtung der Hospiz und Palliativversorgung.

5.4 Soweit weitere Fachkräfte (z.B. Sozialarbeiterinnen / Sozialarbeiter, Sozialpädagoginnen oder Sozialpädagogen, Psychologinnen oder Psychologen) vertraglich eingebunden werden, haben diese eine Zusatzweiterbildung Palliative Care für andere Berufsgruppen oder eine mehrjährige Erfahrung in der Palliativversorgung nachzuweisen.

**6. Qualitätssicherung**

6.1 Die spezialisierten Leistungserbringer sind verpflichtet, ein internes Qualitätsmanagement durchzuführen. Sie nehmen regelmäßig an palliativmedizinischen/- pflegerischen Fortbildungen teil und führen möglichst halbjährlich multidisziplinäre Qualitätszirkel durch, an denen auch die übrigen in der Versorgung Tätigen teilnehmen. Die spezialisierten Leistungserbringer sollen sich außerdem an Maßnahmen der externen Qualitätssicherung beteiligen.

6.2 Ein geeignetes Dokumentationssystem ist sachgerecht und kontinuierlich zu führen und auf Wunsch der Krankenkasse als Konzept vorzulegen. Das Dokumentationssystem muss patientenbezogene Daten und - soweit vorhanden - allgemein anerkannte Indikatoren für eine externe Qualitätssicherung enthalten und eine bundesweite Evaluation ermöglichen. Es soll kompatibel zu den bestehenden Dokumentationssystemen der an der Versorgung beteiligten Leistungserbringer sein.

6.3 Die Teilnahme an Supervision ist zu ermöglichen. Regelmäßige multiprofessionelle Fallbesprechungen sind in überschaubaren Intervallen durchzuführen; die inhaltliche und zeitliche Organisation ist den Beteiligten überlassen.

6.4 Die spezialisierten Leistungserbringer erstellen einen jeweils individuellen Behandlungsplan, der mit den übrigen an der Versorgung beteiligten Leistungserbringern abzustimmen ist.

## Anlage 3 Versorgungsgebiet

Anmerkung: hier wird das konkrete Versorgungsgebiet für jedes PCT geregelt (konkrete Angabe der Kommunen mit Einwohnerzahl, Landkarte)

**Anlage 4 Vorzulegende Unterlagen bei Zulassungsantrag**

Mit dem Zulassungsantrag des Leistungserbringer für ein PCT sind folgende Unterlagen einzureichen:

Versorgungskonzept (Inhalte siehe § 2 des Vertrages zur Erbringung von SAPV im Freistaat Bayern)

Angaben zum Versorgungsgebiet (Kommunen mit Einwohnerzahl, Landkarte)

<u>für den verantwortlichen und den stellvertretenden verantwortlichen Palliativmediziner</u>

☐ Beglaubigte Abschrift/Kopie des Nachweises über eine anerkannte Zusatzweiterbildung Palliativmedizin nach der aktuell gültigen Weiterbildung der jeweiligen Landesärztekammer

☐ Nachweise zur Erfahrung aus der ambulanten palliativen Behandlung von mindestens 75 Palliativpatienten, z. B. in der häuslichen Umgebung oder in einem stationären Hospiz, innerhalb der letzten drei Jahre oder aus einer mindestens einjährigen klinischen palliativmedizinsichen Tätigkeit in einer Palliativabteilung in einem Krankenhaus innerhalb der letzten drei Jahre

☐ Behördliches Führungszeugnis gem. § 30 BZRG (nicht älter als 6 Monate) *)

☐ Arbeitsverträge *)

☐ Stellenbeschreibungen

<u>für die verantwortliche und die stellvertretende verantwortliche Palliativpflegefachkraft</u>

☐ Beglaubigte Abschrift/Kopie der Urkunde über die Erlaubnis zur Führung einer der Berufsbezeichnungen Gesundheits- und Krankenpflegerin / Gesundheits- und Krankenpfleger, Gesundheits- und Kinderkrankenpflegerin / Gesundheits- und Kinderkrankenpfleger oder Altenpflegerin / Altenpfleger (dreijährige Ausbildung aufgrund einer landesrechtlichen Regelung) entsprechend den gesetzlichen Bestimmungen in der jeweils gültigen Fassung

☐ Nachweis über den Abschluss einer Palliative-Care-Weiterbildungsmaßnahme im Umfang von mindestens 160 Stunden

☐ Nachweise über Erfahrung durch mindestens eine zweijährige praktische Tätigkeit als Pflegefachkraft in der Betreuung von Palliativpatienten in den letzten drei Jahren, davon mindestens sechsmonatige Mitarbeit in einer spezialisierten Einrichtung der Hospiz- und Palliativversorgung

☐ Behördliches Führungszeugnis gem. § 30 BZRG (nicht älter als 6 Monate) *)

☐ Arbeitsverträge *)

☐ Stellenbeschreibungen

<u>für das sonstige Personal</u>

☐ Arbeitsverträge *)

☐ Stellenbeschreibungen

<u>für den Dienst</u>

☐ Schriftliche Kooperationsvereinbarungen mit den regelhaft an der Versorgung beteiligten Leistungserbringern. *)

☐ Kooperationsvereinbarungen mit an der Versorgung beteiligten ambulanten Hospizdiensten

☐ Kostenkalkulation für die ersten drei Wirtschaftsjahre

☐ Abschluss einer ausreichenden Betriebs- und Berufshaftpflichtversicherung für Personen-, Sach- und Vermögensschäden *)

☐ Meldung bei der Berufsgenossenschaft für Gesundheitsdienste und Wohlfahrtspflege *)

☐ Meldung beim Gesundheitsamt *)

☐ Meldung beim Finanzamt *)

*) Vorlage spätestens zum Vertragsschluss

<u>Hinweis für den Antragsteller:</u>
Die Bearbeitung des Antrages auf Zulassung als Leistungserbringer von Spezialisierter Ambulanter Palliativversorgung nach dem SGB V benötigt etwas Zeit. Erfahrungsgemäß sind ca. 3 bis 6 Monate erforderlich. Wir bitten dies zu berücksichtigen, zumal die Zulassung erst ausgefertigt werden kann, wenn alle Voraussetzungen vorliegen und auch nachgewiesen sind. Eine rückwirkende Vertragsausfertigung sowie eine rückwirkenden Kostenübernahme ist leider nicht möglich.

**Anlage 5 Evaluation**

Bis zum Vorliegen einer bundesweiten Vorlage für die Evaluation kommt in Bayern der beiliegende Vordruck zum Einsatz. Bis zur Einrichtung einer Annahmestelle für Erfassung und Auswertung der Evaluationsbögen werden diese beim jeweiligen PCT gesammelt.

| Krankenkasse bzw. Kostenträger | | Kassen-Nr. |
|---|---|---|
| Betriebsstätten-Nr. | Institutskennzeichen | Datum |
| | | |

# SAPV- Einzelfallevaluation

gemäß Anlage ... - Mustervertrag

## I. Beginn der spez. ambulanten Palliativversorgung (SAPV)

Patienten-ID: ___________ Geschlecht: ❑ w ❑ m Geburtsjahr: ___________

Grundkrankheit ICD10:____________________

**Folgende Maßnahme wurden durch:** ❑ KH-Arzt ❑ Vertragsarzt **verordnet:**

❑ Beratung ❑ Koordination ❑ Teilversorgung ❑ Vollversorgung

**Erstkontakt:** ❑ im Krankenhaus ❑ Ambulant/Pflegeheim/Hospiz

**Wohnsituation:** ❑ zu Hause, allein ❑ zu Hause mit Angehörigen
❑ im stat.Hospiz ❑ in stat.Pflegeeinrichtung ❑ ____________________

**Pflegestufe:** ❑ 1 ❑ 2 ❑ 3 ❑ beantragt ❑ nicht beantragt

**Wunsch des Pat. zu Beginn:** ______________________________________________________

Wunsch identisch mit SAPV Behandlungsziel: ❑ ja ❑ nein

## II. Durchgeführte spez. ambulanten Palliativversorgung (SAPV)

**Anzahl Verordnungen:** Beratung(en):|_|_| Koordination:|_|_| Teilversorgung:|_|_| Vollversorgung:|_|_|

**Betreuungsrelevante Nebendiagnosen (ICD):** ❑ __________ ❑ __________ ❑ __________

**Komplexes Symptomgeschehen**

A V
❑❑ Schmerzsymptomatik ❑❑ gastrointestinale Symptomatik ❑❑ urogenitale Symptomatik
❑❑ neurologische/psychiatrische/psychische Symptomatik ❑❑ respiratorische/kardiale Symptomatik
❑❑ ulzerierende/exulzerierende Wunden oder Tumore ❑❑ Sonstiges: ____________________

**Weiteres komplexes Geschehen**

A V
❑❑ ethische Konflikte ❑❑ Unterstützungsbedarf des Bezugssystems
❑❑ sozial-rechtliche Probleme ❑❑ existentielle Krisensituation Patient

**Quantitative Aufwandsverteilung (gesamt: 100%) für die Tätigkeiten des Teams**

Am Patient: _____ % Für Angehörige: _____ % Systemische Tätigkeiten: _____ %

**Betreuungsnetz**

A V
❑❑ PC-Pflegekraft ❑❑ Palliativmediziner ❑❑ Hausarzt ❑❑ Facharzt
❑❑ ambulanter Hospizdienst ❑❑ Pflegedienst ❑❑ Sozialstation ❑❑ Sozialarbeit
❑❑ stationäres Hospiz ❑❑ Krankenhaus ❑❑ Palliativstation ❑❑ Stationäre Pflege
❑❑ Physiotherapie ❑❑ Psychologe ❑❑ Apotheke/San.-haus ❑❑ weitere Berufe
❑❑ Angehörige mit Grundpflege ❑❑ Angehörige mit Behandlungspflege ❑❑Seelsorge

**Pat.-Wunsch erfüllt:** ❑ ja ❑ teilweise ❑ nein ❑ unbekannt

## III: Abschluß der spez. ambulanten Palliativversorgung (SAPV)

**Beendigung der SAPV wegen:**

❑ Stabilisierung ❑ Kausaler Therapieansatz ❑ Verlegung ❑ Ablauf der Verordnung
❑ verstorben ❑ Sonstiges: __________________________

**Pflegestufe b. Abschluss:** ❑ 1 ❑ 2 ❑ 3 ❑ beantragt ❑ nicht beantragt

**Zusätzliche Angaben bei Verstorbenen:** ❑ im Krankenhaus verstorben
❑ unter SAPV verstorben ❑ im stat. Hospiz verstorben ❑ im Heim verstorben ❑ zu Hause verstorben

ggf. Grund der letzten stationären Einweisung: ❑ Dekomp. Pat. ❑ Dekomp. Umfeld ❑ Wunsch des Patienten

ggf. weitere Angaben: __________________________________________

Sterbeort n. Wunsch: ❑ ja ❑ nein ❑ unbekannt/unbestimmt

## IV. Leistungskennzahlen

Behandlungsdauer in Tagen: __________

Besuche PC-Team, gesamt: __________

Notarzteinsätze: ❑ keine ❑ 1 ❑ 2 ❑ 3 ❑ 4 ❑ 5 ❑ > 5

KH-Einweisungen: ❑ keine ❑ 1 ❑ 2 ❑ 3 ❑ 4 ❑ 5 ❑ > 5

Anfahrtsweg in km: ❑ 0-10 ❑ 11-20 ❑ 21-30 ❑ 31-50 ❑ > 50

Palliativarzt / Palliative Care Fachkraft

SAPV-Einzelfallevaluation Bayern (30.06.2009)

# SAPV- Erläuterungen zur Einzelfallevaluation

gemäß Anlage ... - Mustervertrag

## Einleitung

Der vorliegende Bogen soll sowohl die fachlich notwendige wie gesetzlich geforderte Evaluation ermöglichen. Ihm zugrunde liegen die publizierten Daten der DGP, des DHPV und anderer sowie die eigenen Erfahrungen der Herausgeber. Ziel war es, eine ausreichende und aussagefähige Evaluation auf maximal einer Din A4-Seite oder elektronischem Korrelat zu erreichen. Angesichts einer Evaluation von bundesweit bis zu 100.000 Bögen / Jahr muss von Anfang an auf Maschinenlesbarkeit bzw. digitale Eingabe der Daten Wert gelegt werden.

Die Eingabe der Daten erfolgt zwar anonymisiert durch den Leistungserbringer; trotzdem kann durch eine Kombination von Evaluations- und Abrechnungsdaten auf die Identität eines Patienten rückgeschlossen werden. Daraus folgt, dass die Evaluation der SAPV durch einen unabhängigen Dienstleister erfolgen muss, der sicherstellt, dass die Ausgabe der Daten so erfolgt, dass ein Rückschluss auf einzelne Patienten nicht möglich ist.

## Abschnitt 1: Beginn der spez. ambulanten Palliativversorgung (SAPV)

Die Angaben zur Betriebsstättennummer und dem Institutskennzeichens des Palliative-Care-Teams (PCT) wie zur Krankenkasse sind notwendige Stammdaten zur Zuordnung einer Evaluation zu einer Krankenkasse bzw. zum PCT.

Patienten-ID: anonymisierte Eingabe eines Identifier, der vom PC-Team intern festgelegt wird; dieser ergibt in Kombination mit dem Institutskennzeichen einen eindeutigen Identifier in der Evaluation.

Grundkrankheit in ICD10-Kodierung: Es wird nur die Grundkrankheit erfragt, welche zur palliativen Situation führt. Die SAPV-relevanten Nebendiagnosen werden erst im Verlauf der Behandlung offensichtlich und sind deshalb unter II. zu finden.

Erfassung der Verordnung zwischen Krankenhausarzt und niedergelassenem Arzt zur Differenzierung; ebenso wird der zunächst durchgeführte Verordnungsumfang erfasst.

Der Ort des Erstkontakts unterscheidet nur zwischen stationärem und ambulanten Sektor (Krankenhaus oder Ambulant/Pflegeheim/Hospiz)

Die normale Wohnsituation des Patienten wird zusätzlich zum Erstkontakt des PCT erfasst, da diese für die SAPV-Betreuung von entscheidender Bedeutung ist.

An Stelle des ECOG oder Karnowsky-Index wurde zur orientierenden Beurteilung der noch möglichen Selbständigkeit des Patienten die Pflegestufe gewählt; es muss entweder eine bereits vorhandene Pflegestufe oder eines der beiden Kästchen angekreuzt werden Gemeinsam mit der Abfrage unter III wird auch dargestellt, ob hier ein erhöhter Organisationsaufwand bestand / finanzielle Unsicherheit für die Familie.

Mit "Wunsch des Patienten zu Beginn der SAPV" ist der „realistische Wunsch des Patienten gegenüber dem PCT" gemeint. Ausdrücklich soll hier der Patient zu Wort kommen. Zur Erleichterung des Eintrages werden folgende Kategorien vorgegeben, die nach bisherigen Ergebnissen (Auswertung von 100 Bögen bei PALLIAMO) etwa 80% der Wünsche abdecken: 1. Zu Hause bleiben können / Kein KH / Autonomie; 2. Leidenslinderung; 3. Palliative Rehabilitation („wieder aufstehen können", „noch eine Reise machen"); 4. In Ruhe gelassen werden. Als fünfte Möglichkeit ist auch die Angabe eines Freitextes möglich, falls keine dieser Kategorien zutrifft.

Wenn das PCT diesen Wunsch für realistisch bzw. realisierbar hält und deshalb mitverfolgt, wird unter „Wunsch identisch mit Behandlungsziel" „ja" angegeben, wenn nicht, dann „nein". Inwieweit die Erreichung des Wunsches erfolgt ist, wird unter II. angegeben.

## Abschnitt 2: Durchgeführte spez. ambulanten Palliativversorgung (SAPV)

Dieser Bogen wird einmal pro abgeschlossenem SAPV-Fall, also je Erstverordnung, ausgefüllt. Die Häufigkeit der Folgeverordnungen ist für den Verwaltungsaufwand von Interesse. Zudem kann sich im Verlauf einer SAPV die geforderte Kategorie ändern, weshalb die Anzahl der erhaltenen Verordnungen je Kategorie erfasst werden. Da in einigen SAPV-Verträgen eine Herabstufung ohne erneute Verordnung möglich ist, soll bei der Erstverordnung, wie unter Abschnitt 1 angegeben, die tatsächlich durchgeführte Versorgung angegeben werden. Bei den Folgeverordnungen dürften in aller Regel Verordnung und durchgeführte Kategorie übereinstimmen.

Das zu Beginn bestehende komplexe Symptomgeschehen kann sich, besonders bei einer Vollversorgung, im Verlauf von der tatsächlichen Versorgungsnotwendigkeit unterscheiden. Aus diesem Grunde wird beides erfasst: das initiale komplexe Symptomgeschehen (A), welches also in der Erstverordnung benannt wurde, sowie das Symptomgeschehen im weiteren Verlauf(V) - was also alles durch das PCT gelindert werden musste. Der Gesetzgeber hat weitere Gründe für eine SAPV-Verordnung zugelassen, also sollten wir sie auch erfassen. Neben dem Freitext unter „Sonstiges" werden im folgenden einige in Realität ebenfalls häufige komplexe Probleme bereits vorgegeben.

Das größte Problem der Evaluation von SAPV besteht darin, dass wir die tatsächliche Ergebnisleistung des PCT nicht erfassen können. Sie besteht darin, die äußeren Umstände so zu gestalten, dass Patient und Familie auf ihre Weise mit der letzten Lebenszeit zurechtkommen. Dies kann im Moment noch nicht valide abgebildet werden.

Ersatzweise wird der quantitative Aufwand der Tätigkeiten des PCT dargestellt. Hierzu wird dieser in Prozent auf die drei Items Patient, Angehörige und systemische Tätigkeiten verteilt, um einerseits das Verhältnis von patientennahen zu patientenfernen Tätigkeiten und andererseits die Differenzierung zwischen Patient und Umfeld zu ermöglichen.

Auch die Beteiligung am Betreuungsnetz kann sich im Verlauf einer SAPV verändern oder erweitern. Unter „A" (Anfang der SAPV) werden alle beim Erstkontakt an der Versorgung Beteiligten markiert. Bei „V" (Verlauf der SAPV) werden alle markiert, die während der SAPV-Versorgung weiterhin oder neu involviert waren. Die pflegerische und medizinische Versorgung durch das SAPV-Team wird als Minimum vorausgesetzt. Sind im PCT-Team weitere Berufsgruppen tätig (z.B. ein Sozialarbeiter) und wird dieser am Patienten tätig, wird dies unter Sozialarbeit mit „V" markiert analog zu einem Sozialarbeiter, der extern zugezogen wurde. Seelsorge meint nicht nur einen Geistlichen, sondern auch andere Professionelle mit Befähigung zu seelsorgerischen Tätigkeit, wenn sie in dieser Funktion tätig waren. (Beispiel: Eine Hospizbegleiterin wird explizit angefordert, um spirituelle Probleme zu thematisieren. Es kommt eine Ehrenamtliche zum Einsatz, die Ausbildung und Erfahrung aus ihrer Tätigkeit im Besuchsdienst der Krankenhaus-Seelsorge mitbringt. Dann wird im Evaluationsbogen „ambulanter Hospizdienst" angekreuzt, gleichzeitig auch „Seelsorge").

Als weitere qualitative Aussage soll das Erreichen des unter I. beschriebenen Wunsch des Patienten kategorisierend erfasst werden.

## Abschnitt 3. Abschluß der spez. ambulanten Palliativversorgung (SAPV)

Die Beendigung der SAPV erfolgt wegen Stabilisierung (was automatisch die Weiterbetreuung durch die AAPV bedeutet), wegen Verlegung (also nicht Stabilisierung, sondern z.B. Umzug, auch: Aufnahme ins stat. Hospiz ohne Weiterbetreuung durch SAPV), oder weil der Patient verstorben ist (weitere Differenzierung dazu etwas später). Ein Freitext für evt. noch nicht erfasste Gründe ist vorgesehen. Explizit ist noch der Ablauf der Verordnung ohne Folgeverordnung aufgeführt. Hier ist nicht die Stabilisierung gemeint, sondern die hoffentlich seltene Situation, dass trotz Bedarfs keine Folgeverordnung ausgestellt wird (z. B. nach Erstverordnung durch Klinikarzt).

Krankenhausaufenthalte unterbrechen eine SAPV-Betreuung (ein Grund für intermittierende Betreuung), sie beenden sie nur, wenn der Patient im Krankenhaus stirbt. Nur dann ist nach dem Grund für diese letzte Einweisung gefragt.

Für wichtig wird erachtet, zum Sterbeort anzugeben, ob dieser dem Wunsch des Patienten entsprach (ja /nein) bzw. ob es keine Angaben des Patienten gab bzw. diese unbekannt waren.

Zur Erfassung von Leistungskennzahlen pro SAPV-Fall wurden die Behandlungsdauer in Tagen sowie der Anzahl der Besuche aufgenommen.

Während einer Vollversorgung sollten Notarzteinsätze und Krankenhauseinweisungen unabhängig von der Struktur des PC-Teams selten sein. Hier kann die Zahl einen Rückschluss auf die Qualität und Verfügbarkeit des PC-Teams liefern. Diese Zahlen werden nur für eine Vollversorgung erfasst, da in den anderen Kategorien dieser Zusammenhang nicht eindeutig ist.

## Anlage 6 Formular für den Leistungsnachweis

**Leistungsnachweis über erbrachte Leistungen im Rahmen der SAPV**
**nach § 132 d SGB V_Entwurf: Stand 29.06.09**

**Versicherter (Name, Vorname):** ____________________
**Anschrift:** ____________________
**Geburtsdatum:**
**Versichertennummer:** ____________________
**Krankenkasse:** ____________________

**LEGS:**
**Leistungserbringer:**
**Anschrift:**

**Institutionskennzeichen**

| Betreuungsart | Beginn | Ende | Gesamte Tage |
|---|---|---|---|
| | | | |
| | | | |
| | | | |
| | | **Gesamttage** | |

**Monat:** ____________________ **Monat:** ____________________

| | Voll-/Teil-Vers. 24-h-Bereitschaft | Besuchsanzahl | Besuchsdauer | Fahrdauer | | | | | | | | Voll-/Teil-Vers. 24-h-Bereitschaft | Besuchsanzahl | Besuchsdauer | Fahrdauer | | | | | | |
|---|---|---|---|---|---|---|---|---|---|---|---|---|---|---|---|---|---|---|---|---|---|
| 1 | | | | | | | | | | | 1 | | | | | | | | | | |
| 2 | | | | | | | | | | | 2 | | | | | | | | | | |
| 3 | | | | | | | | | | | 3 | | | | | | | | | | |
| 4 | | | | | | | | | | | 4 | | | | | | | | | | |
| 5 | | | | | | | | | | | 5 | | | | | | | | | | |
| 6 | | | | | | | | | | | 6 | | | | | | | | | | |
| 7 | | | | | | | | | | | 7 | | | | | | | | | | |
| 8 | | | | | | | | | | | 8 | | | | | | | | | | |
| 9 | | | | | | | | | | | 9 | | | | | | | | | | |
| 10 | | | | | | | | | | | 10 | | | | | | | | | | |
| 11 | | | | | | | | | | | 11 | | | | | | | | | | |
| 12 | | | | | | | | | | | 12 | | | | | | | | | | |
| 13 | | | | | | | | | | | 13 | | | | | | | | | | |
| 14 | | | | | | | | | | | 14 | | | | | | | | | | |
| 15 | | | | | | | | | | | 15 | | | | | | | | | | |
| 16 | | | | | | | | | | | 16 | | | | | | | | | | |
| 17 | | | | | | | | | | | 17 | | | | | | | | | | |
| 18 | | | | | | | | | | | 18 | | | | | | | | | | |
| 19 | | | | | | | | | | | 19 | | | | | | | | | | |
| 20 | | | | | | | | | | | 20 | | | | | | | | | | |
| 21 | | | | | | | | | | | 21 | | | | | | | | | | |
| 22 | | | | | | | | | | | 22 | | | | | | | | | | |
| 23 | | | | | | | | | | | 23 | | | | | | | | | | |
| 24 | | | | | | | | | | | 24 | | | | | | | | | | |
| 25 | | | | | | | | | | | 25 | | | | | | | | | | |
| 26 | | | | | | | | | | | 26 | | | | | | | | | | |
| 27 | | | | | | | | | | | 27 | | | | | | | | | | |
| 28 | | | | | | | | | | | 28 | | | | | | | | | | |
| 29 | | | | | | | | | | | 29 | | | | | | | | | | |
| 30 | | | | | | | | | | | 30 | | | | | | | | | | |
| 31 | | | | | | | | | | | 31 | | | | | | | | | | |

An der Versorgung waren folgende Kooperationspartner beteiligt:
1
2
3

Hiermit bestätige ich die Richtigkeit der Angaben auf dem Leistungsnachweis: ____________________ Ort, Datum ____________________ Leitender Arzt des PCT

## Anlage 7

**zum Vertrag über die Erbringung Spezialisierter Ambulanter Palliativversorgung (SAPPV) gemäß §§ 132 d i. V. m. 37 b SGB V im Freistaat Bayern (Vertrag zur Erbringung von SAPV)**

**zwischen**

**den Landesverbänden der Krankenkassen**

der AOK Bayern - Die Gesundheitskasse,
dem BKK Landesverband Bayern,
der Vereinigten IKK,
dem Funktionellen Landesverband der landwirtschaftlichen Kranken- und Pflegekassen in Bayern,
der Knappschaft - Regionaldirektion München,

**den Ersatzkassen**

Barmer Ersatzkasse,
Techniker Krankenkasse,
Deutsche Angestellten-Krankenkasse,
KKH Allianz,
Gmünder ErsatzKasse – GEK,
HEK - Hanseatische Krankenkasse,
Hamburg Münchener Krankenkasse
hkk,

**gemeinsamer Bevollmächtigter mit Abschlussbefugnis**
**Verband der Ersatzkassen e. V. (vdek),**
**vertreten durch den/die Leiter/in der Landesvertretung Bayern**

**im Folgenden Krankenkassen genannt**

**und**

**......................................**
**.......................................**
**..................................**
**.................................**
**im Folgenden „Leistungserbringer“ genannt**

-

wird unter Hinweis auf § 27 des Vertrages zur Erbringung von SAPPV folgende Vergütungsvereinbarung geschlossen:

**...........................................................................................................................................**
**...........................................................................................................................................**

**Anlage 8 Übergangsregelung**

Abweichend vom § 4 vereinbaren deshalb die Vertragspartner folgende Übergangsregelungen:

Die Übergangsregelung läuft vom......... bis..........

## Anlage 9 Statistik

Name des Leistungserbringers

Betriebsstätten-Nr. | Datum

# SAPV - Statistische Angaben

gemäß Anlage ... - Mustervertrag

vom T T M M J J

### 1. Angaben zum Team

Besetzte Stellen insgesamt [VK-Stellen], ____,__

davon Ärzte [VK-Stellen] ____,__

davon Palliative Care Pflegekräfte [VK-Stellen] ____,__

davon Sozialarbeiter [VK-Stellen] ____,__

davon weitere Berufsgruppe [VK-Stellen]: ____________ ____,__

davon Verwaltung [VK-Stellen] ____,__

Anzahl der Kooperationsparter [Anzahl] ____,__

Summe der Fortbildungenstunden des Teams [h/a] ____,__

Gesamtanzahl Supervisionen [n/a] ____

Weitere Leistungen (z.B. Einzelsupervisionen) [n/a] ____

### 2. Zusätzliches Angebot

Anzahl der Fortbildungsstunden durch Teammitglieder (auch außerhalb Arbeitszeit) [n/a] ____,__

Erinnerungsfeier(n) für Verstorbene (auch außerhalb Arbeitszeit) [n/a] ____,__

Weitere ehrenamtliche Besuche durch Teammitglieder [n/a] ____,__

### 3. Sterbeorte

Gesamtanzahl der in der SAPV Verstorbenen [n/a], ____

davon zu Hause verstorben [n/a]: ____ davon im stat. Hospiz verstorben [n/a]: ____

davon im Krankenhaus verstorben [n/a]: ____ davon im Pflegeheim verstorben [n/a]: ____

### 4. Leistungszahlen

Insgesamt eingegangene SAPV-Verordnungen [n/a] ____

davon Anzahl der nicht ausführbaren/ nicht ausgeführten Verordnungen [n/a] ____

Durchgeführte Anzahl der Leistungen [n/a], ____

davon Beratungen [n/a] ____

davon Koordinationen [n/a] ____

davon Teilversorgungen [n/a] ____

davon Vollversorgungen [n/a] ____

| | | | | |
|---|---|---|---|---|
| Nur Koordination: | Ø Koordinationsdauer [d]: | ____,__ | Ø Kontakte [n]: | ____,__ |
| Durchschnittl. Behandlungsdauer [d] | Teilversorgung: | ____,__ | Vollversorgung: | ____,__ |
| Durchschnittliche Anzahl Besuche [n] | Teilversorgung: | ____,__ | Vollversorgung: | ____,__ |
| Durchschnittl. Anzahl der Notarzteinsätze [n] | Teilversorgung: | ____,__ | Vollversorgung: | ____,__ |
| Durchschnittl. Anzahl der KH-Einweisungen [n] | Teilversorgung: | ____,__ | Vollversorgung: | ____,__ |

Durchschnittl. Anfahrtsweg bei Teil- oder Vollversorgung [km] ____,__

### 5. Vergütungen & Kostenübernahme

Anzahl der genehmigten SAPV-Verordnungen [n/a] ____

Anzahl der abgelehnten SAPV-Verordnungen [n/a], ____

davon Teilablehnungen [n/a] ____

Anzahl verzögerter Zahlungseingänge [n/a] ____

Palliativarzt / Palliative Care Fachkraft

SAPV-Teamevaluation Bayern (Version vom 30.06.2009)

Legende: Angabe der Einheiten in eckigen Klammern VK = Vollkraftstellen; h = Stunden; d = Tage; a = annum (Jahr); n = Anzahl; Ø = Durchschnitt von ...

## Anlage 10 SAPV-BSNR-Vereinbarung

Vereinbarung zwischen dem GKV-Spitzenverband und der kassenärztlichen Bundesvereinigung über die Vergabe von Betriebsstätten-Nummern und einer Pseudo-Arztnummer an Leistungserbringer der SAPV gemäß § 132d Abs.1 SGB V zur Verordnung von Arznei-, Heil- und Hilfsmitteln im Rahmen der SAPV (SAPV-BSNR-Vereinbarung, in Kraft getreten am 1.5.2009)

sihe
www.dgpalliativmedizin.de/**sapv**.html

## Glossar / Abkürzungsverzeichnis (kein Vertragsinhalt)

Auf der Website der Deutschen Gesellschaft für Palliativmedizin e.V. (http://www.dgpalliativmedizin.de) und des Deutschen Hospiz- und Palliativverbandes e.V. (http://www.hospiz.net/sapv/index.html) kann das stetig aktualisierte Glossar heruntergeladen werden.

Baden-Württemberg

**Vertrag gemäß § 132d Abs. 1 i. V. m. § 37b SGB V**
**über die**
**spezialisierte ambulante Palliativversorgung (SAPV)**

zwischen der
vertreten durch
für das
**Palliative-Care-Team in XXXXXXXX**
(nachfolgend PCT genannt)

und

AOK Baden-Württemberg, Stuttgart

BKK-Vertragsarbeitsgemeinschaft Baden-Württemberg
Geschäftsstelle beim BKK Landesverband Baden-Württemberg
Stuttgarter Straße 105, 70806 Kornwestheim
vertreten durch die Vorsitzenden der Mitgliederversammlung

IKK classic, Hauptverwaltung Ludwigsburg
vertreten durch den Vorsitzenden des Vorstandes

Den Ersatzkassen
Barmer GEK
Techniker Krankenkasse (TK)
Deutsche Angestellten-Krankenkasse (Ersatzkasse)
KKH-Allianz (Ersatzkasse)
HEK - Hanseatische Krankenkasse
Hamburg Münchener Krankenkasse
hkk
gemeinsamer Bevollmächtigter mit Abschlussbefugnis gem. § 212 (5) S.6 SGB V
Verband der Ersatzkassen e. V. (vdek),
vertreten durch den Leiter der vdek-Landesvertretung Baden-Württemberg, Stuttgart,

Landwirtschaftlichen Krankenkasse Baden-Württemberg, Stuttgart

Knappschaft, Regionaldirektion München

(nachfolgend Krankenkasse genannt)

**Inhaltsverzeichnis:** **Seitenzahl**


| | | |
|---|---|---|
| | Präambel | Seite 3 |
| § 1 | Ziel und Gegenstand | Seite 3 |
| § 2 | Anspruchsvoraussetzungen des Versicherten | Seite 4 |
| § 3 | Leistungsempfänger | Seite 4 |
| § 4 | Grundsätze der Leistungserbringung | Seite 5 |
| § 5 | Verordnung und Genehmigung, vorl. Kostenzusage | Seite 6 |
| § 6 | Strukturanforderungen an das PCT | Seite 8 |
| § 7 | Personelle Anforderungen | Seite 8 |
| § 8 | Sächliche Anforderungen | Seite 10 |
| § 9 | Aufgaben des PCT | Seite 11 |
| § 10 | Qualitätssicherung | Seite 12 |
| § 11 | Vergütung und Abrechnung | Seite 13 |
| § 12 | Verordnung von Arznei-, Heil- und Hilfsmittel | Seite 13 |
| § 13 | Datenschutz / Schweigepflicht | Seite 14 |
| § 14 | Sonstige Bestimmungen | Seite 14 |
| § 15 | Inkrafttreten/Kündigung | Seite 15 |
| § 16 | Salvatorische Klausel | Seite 15 |


**Anlagen:**


| | | |
|---|---|---|
| Anlage 1 | Strukturerhebungsbogen, Konzeptanforderungen | Seite 20 |
| Anlage 2 | Vergütungsvereinbarung | Seite 32 |
| Anlage 3 | Rechnungsblatt | Seite 36 |
| Anlage 4 | Leistungsnachweis | Seite 37 |
| Anlage 5 | Mustervordruck zur Beantragung der Vergabe der Betriebsstättennummer (BSNR) bei der KBV | Seite 38 |
| Anlage 6 | Liste der teilnehmenden Betriebskrankenkassen | Seite 39 |

## Präambel

Die spezialisierte ambulante palliative Versorgung dient dem Ziel, die Lebensqualität und die Selbstbestimmung schwerstkranker Menschen zu erhalten, zu fördern und zu verbessern und ihnen ein menschenwürdiges Leben bis zum Tod in ihrer häuslichen Umgebung oder in stationären Pflegeeinrichtungen zu ermöglichen. Das Sterben zu Hause stellt heute noch eine Ausnahme dar, obwohl 70 % der Betroffenen diesen Wunsch haben. Die Vertragspartner stimmen in der Einschätzung überein, dass die Voraussetzungen für ein menschenwürdiges Leben bis zum Tod in der häuslichen Umgebung durch zielgerichtetes Zusammenwirken von Haus- und Fachärzten (nachfolgend Vertragsärzte genannt), Leistungserbringern der SAPV, ambulanten Pflege- und Hospizdiensten sowie psychoonkologischen Betreuungsdiensten verbessert werden können und müssen. Die individuellen Bedürfnisse und Wünsche dieser Menschen sowie die Belange ihrer Angehörigen oder vertrauten Personen stehen dabei im Mittelpunkt der Versorgung. Der Patientenwille, der auch durch Patientenverfügungen zum Ausdruck kommen kann, ist zu beachten.

## § 1
## Ziel und Gegenstand

(1) Ziel der Vereinbarung ist es, eine ambulante Versorgung unheilbar kranker Menschen in der letzten Phase ihres Lebens in häuslicher Umgebung zu sichern, ihre Lebensqualität unter Berücksichtigung des Krankheitsstadiums zu verbessern bzw. zu erhalten und ihnen ein menschenwürdiges Leben bis zum Tod in ihrer häuslichen Umgebung, in einer stationären Pflegeeinrichtung oder einem Hospiz zu ermöglichen. Weitere Ziele sind die Strukturierung der Behandlungsabläufe und deren Anpassung an den besonderen Bedarf der betroffenen Versicherten im ambulanten Bereich. Die Leistungserbringung soll ausreichend und wirtschaftlich sein und das Maß des Notwendigen nicht überschreiten.

(2) Gegenstand des Vertrages sind

- die „Richtlinie des Gemeinsamen Bundesausschusses zur Verordnung spezialisierter ambulanter Palliativversorgung“ (SAPV-RL) vom 20. Dezember 2007 in ihrer jeweils gültigen Fassung.
- die „Gemeinsamen Empfehlungen der Spitzenverbände der Krankenkassen nach § 132 d Abs. 2 SGB V für die spezialisierte ambulante Palliativversorgung“ vom 23.06.2008.

## § 2
## Anspruchsvoraussetzungen des Versicherten

(1) Der Anspruch des Versicherten wird durch § 37 b SGB V und die „Richtlinie des Gemeinsamen Bundesausschusses zur Verordnung von ambulanter und spezialisierter Palliativversorgung“ (SAPV-RL) vom 20. Dezember 2007 in ihrer jeweils gültigen Fassung definiert.

(2) Versicherte haben Anspruch auf SAPV, wenn

- sie an einer nicht heilbaren, fortschreitenden und so weit fortgeschrittenen Erkrankung leiden, dass dadurch ihre Lebenserwartung begrenzt ist (§ 3 der Richtlinie des Gemeinsamen Bundesausschusses zur Verordnung von SAPV) und
- sie (unter Berücksichtigung der in § 1 genannten Ziele) eine besonders aufwändige Versorgung (§ 4 der Richtlinie des Gemeinsamen Bundesausschusses zur Verordnung von SAPV) benötigen, die nach den medizinischen und pflegerischen Erfordernissen auch ambulant oder in stationären Pflegeeinrichtungen (§ 72 SGB XI) erbracht werden kann.

Anhaltspunkt für eine besonders aufwändige Versorgung ist das Vorliegen eines komplexen Symptomgeschehens mit ausgeprägter Symptomatik, bei dem kurzfristig notwendige Anpassungen der Therapie durch kompetente Palliativfachkräfte erforderlich werden können.

(3) Das PCT hat nach Erhalt der Verordnung zu prüfen, ob bereits aus der Verordnung selbst hervorgeht, dass die Voraussetzungen für die Inanspruchnahme gemäß der in Absatz 2 genannten Definition offensichtlich nicht vorliegen.

(4) Die allgemeine ambulante Palliativ-Versorgung obliegt grundsätzlich den die Versicherten betreuenden Vertragspartnern und Leistungserbringern im Rahmen der ambulanten Versorgung und ist nicht Gegenstand dieses Vertrages.

(5) Sofern Kinder und Jugendliche versorgt werden, sind deren Belange besonders zu berücksichtigen. Bezüglich der Anspruchsvoraussetzungen gilt Absatz 2.

## § 3
## Leistungsempfänger

Aufgrund dieses Vertrags erhalten SAPV-Leistungen die nach § 2 Abs. 2 (ggf. § 2 Abs. 5) berechtigten Versicherten der AOK Baden-Württemberg, der IKK classic sowie weiterer Innungskrankenkassen, die diesem Vertrag beigetreten sind, der Knappschaft Regionaldi-

rektion München, der Landwirtschaftlichen Krankenkasse Baden-Württemberg, der Krankenkasse für den Gartenbau, der im Rubrum genannten Ersatzkassen sowie der in der Anlage 6 aufgeführten Betriebskrankenkassen. Eine Veröffentlichung der jeweils aktuellen Liste der teilnehmenden Betriebskrankenkassen erfolgt im Internet auf der Homepage des BKK Landesverbandes Baden-Württemberg.

## § 4
## Grundsätze der Leistungserbringung

(1) Spezialisierte ambulante Palliativversorgung wird dem Versicherten als Sachleistung zur Verfügung gestellt und wird vom PCT intermittierend oder durchgängig nach Bedarf erbracht als

- Beratungsleistung
  - Telefonat bzw. persönliches Gespräch mit Betroffenen
  - Telefonat bzw. persönliches Gespräch mit einem/einer der Leistungserbringer der Primärversorgung
  - Dokumentation
- Koordination der Versorgung
  - Persönlicher oder telefonischer Kontakt mit den an der Versorgung Beteiligten
  - Ressourcenfokussierte Versorgungsplanung
  - Vernetzung mit ambulanten und/oder stationären Leistungserbringern
  - Dokumentation
- Additiv unterstützende Teilversorgung/vollständige Versorgung
  - Beratungs- und Koordinationsleistung
  - Hausbesuche zu Tages- und zu Nachtzeit
  - Einzelne bzw. alle Leistungen der in § 5 Abs. 3 der SAPV-Richtlinie aufgeführten Leistungen
  - Für die verordnete Teilversorgung/vollständige Versorgung besteht eine 24-Stunden-Rufbereitschaft und Einsatzbereitschaft.
    Die Rufbereitschaft kann nicht als alleinige Teilleistung verordnet werden.
  - Dokumentation und Evaluation

Sie ergänzt die allgemeine Palliativversorgung und das bestehende ambulante und stationäre Versorgungsangebot, insbesondere das der Vertragsärzte, Krankenhäuser, ambulanten und stationären Pflegeeinrichtungen und Hospize.

(2) Die Versorgung der Versicherten muss ausreichend und zweckmäßig sein, sie orientiert sich am individuellen Hilfebedarf des Versicherten, darf das Maß des Notwendigen nicht überschreiten und muss wirtschaftlich erbracht werden.

(3) Beratungsleistungen können nur in engem Zusammenhang mit dem die SAPV begründenden Krankheitsbild erbracht werden. Sie ergänzen die palliativpflegerische Beratung der ambulanten Hospizdienste sowie die Beratungspflichten des behandelnden Arztes bzw. Pflegedienstes und ersetzen diese nicht.

(4) Die Annahme von Aufträgen und deren Weitergabe durch Vermittlung an Dritte gegen Entgelt oder zur Erlangung geldwerter Vorteile ist unzulässig. Vermittlung im Sinne dieser Bestimmung ist auch die regelmäßige Weitergabe von in eigenem Namen angenommenen Aufträgen an Dritte gegen Kostenerstattung.

(5) Der Versicherte ist in der Wahl des PCT frei.

## § 5
## Verordnung und Genehmigung, vorläufige Kostenzusage

(1) Art, Umfang, Inhalt und Dauer der vom PCT zu erbringenden Leistungen ergeben sich aus der Verordnung der/des behandelnden Vertragsärztin/-arztes sowie ggf. der/des Krankenhausärztin/-arztes. Bei Verordnung durch eine/einen Krankenhausärztin/-arztes ist die Dauer der Verordnung auf längstens sieben Tage begrenzt. Auf Grundlage der Verordnung und ggf. des Ergänzungsfragebogens zur ärztlichen Verordnung leitet das PCT das Genehmigungsverfahren ein.

(2) Solange die Genehmigung als Grundlage der weiteren Leistungserbringung nicht vorliegt, erfolgt die Leistungserbringung im Rahmen der vorläufigen Kostenübernahme. Rechtzeitig vor Ablauf der Erstverordnung ist ggf. eine notwendige Folgeverordnung einzureichen und zur Genehmigung der Krankenkasse vorzulegen.

(3) Rückwirkende Verordnungen sind grundsätzlich nicht zulässig. Ausnahmefälle sind besonders zu begründen und können im Einzelfall genehmigt werden.

(4) Die Krankenkasse übernimmt vom ärztlich festgelegten Leistungsbeginn an bis zur Entscheidung über die Genehmigung die Kosten für die von der /dem Vertragsärztin/-arzt verordneten und vom PCT erbrachten Leistungen, wenn die vollständig ausgefüllte Verordnung (insbesondere die verordnungsrelevanten Diagnosen als medizinische Begründung, der Behandlungs-, Betreuungs- und/oder Hilfebedarf des Versicherten, der Beginn und die Dauer der zu erbringenden Leistungen sowie das Ausstellungsdatum) der zu-

ständigen Krankenkasse vorliegt. Näheres zur Vergütung der Kosten ist in § 11 dieses Vertrages und der dazugehörigen Anlage 2 geregelt. Ggf. sollte die Verordnung und sofern vorhanden der Ergänzungsfragebogen vorab per Telefax (Empfangsbereitschaft wird seitens der Krankenkassen jederzeit sichergestellt) vollständig übersandt werden (Original ist dann unverzüglich nachzureichen).

(5) Über die Leistungserbringung entscheidet die jeweils zuständige Krankenkasse grundsätzlich innerhalb einer Woche. Wird von der Krankenkasse eine Ablehnung der ärztlich verordneten Leistung für erforderlich gehalten oder genehmigt sie Leistungen nicht im vollen ärztlich verordneten Umfang, wird diese Entscheidung der/dem Versicherten und dem PCT schriftlich bekannt gegeben.

(6) Bei Ablehnung der Leistung durch die zuständige Krankenkasse übernimmt die Krankenkasse vom ärztlich festgelegten Leistungsbeginn an bis zum Tag der Ablehnung der Kostenübernahme die Kosten für die vom Vertragsarzt/Krankenhausarzt verordneten und vom Leistungserbringer erbrachten Leistungen, wenn die Verordnung am dritten der auf die Ausstellung folgenden Arbeitstag der zuständigen Krankenkasse vorliegt. Näheres zur Vergütung der Kosten ist in § 11 dieses Vertrages und der dazugehörigen Anlage 2 geregelt. Sofern die Verordnung verspätet eingereicht wird, werden die Kosten ab Eingangsdatum übernommen.

(7) Kann die Durchführung der verordneten Leistung nicht oder nicht mehr erfolgen, z.B. weil der/die Versicherte in ein Krankenhaus eingewiesen wurde, informiert der Leistungserbringer die zuständige Krankenkasse und den behandelnden Arzt.
Im Falle der Besserung bzw. einer Stabilisierung im Krankheitsverlauf ist der Versorgungsumfang der SAPV so weit wie möglich zu reduzieren und eine Weiterversorgung im Rahmen der allgemeinen Palliativversorgung anzustreben. Das PCT hat den verordnenden Arzt und die Krankenkasse hierüber unverzüglich zu informieren.

(8) Änderungen oder Ergänzungen der Verordnung im Rahmen der Anpassung des bereits genehmigten Versorgungsumfangs obliegen dem behandelnden Vertragsarzt bzw. dem Krankenhausarzt in Abstimmung mit dem PCT und bedürfen einer erneuten Bestätigung mit Arztunterschrift, Stempel und Datum sowie der Genehmigung durch die Krankenkasse.

(9) Im Sinne der Wirtschaftlichkeit sind verordnete Leistungen der SAPV und der Häuslichen Krankenpflege sowie Leistungen der Pflegeversicherung, soweit möglich und sinnvoll, innerhalb eines Einsatzes zu erbringen.

## § 6
## Strukturanforderungen an das PCT

(1) Grundlage für den Vertragsabschluss bildet der Strukturerhebungsbogen nach Anlage 1 und ein verbindliches, strukturiertes, schriftliches Konzept, in dem der inhaltliche und organisatorische Rahmen der Leistungserbringung (inkl. den personellen und sächlichen Anforderungen), das Versorgungsgebiet sowie die Einbindung in die regionale Versorgungsstruktur beschrieben sind. Bestehende Strukturen sind zu berücksichtigen und einzubinden. Regionale Gebietsgrenzen sind ggf. zu überbrücken.

(2) Das Konzept ist den Krankenkassen vor Vertragsabschluss einzureichen.

(3) Das PCT stellt im Rahmen seiner Kapazitäten die Versorgung der Versicherten in seinem Versorgungsraum mit Leistungen der SAPV sicher.

(4) Die Festlegung des Einzugsbereiches schließt den Abschluss von Verträgen mit anderen Leistungserbringern zur Versorgung der Versicherten mit SAPV im selben Einzugsbereich nicht aus.

(5) Durch Kooperation aller am Versorgungsprozess Beteiligten ist auf eine effiziente Leistungserbringung und eine vertrauensvolle Zusammenarbeit mit dem Versicherten und seinen Bezugspersonen hinzuwirken.

(6) Für die Erfüllung der Leistungen ist in einem PCT eine Koordinationsstelle erforderlich, die im Sinne einer Leitstelle als Ansprechpartner für die verschiedenen Leistungserbringer und für alle Beteiligten als Schnittstelle tätig ist.

## § 7
## Personelle Anforderungen

(1) Die Leistungen der SAPV werden durch Personen erbracht, die folgende Qualifikationsvoraussetzungen erfüllen:

a) Ärzte

- eine anerkannte Zusatzweiterbildung Palliativmedizin nach der aktuell gültigen Weiterbildungsordnung der Landesärztekammer

  und

  Erfahrung aus der ambulanten palliativen Behandlung von mindestens 75 Palliativpatienten, z. B. in der häuslichen Umgebung oder in einem stationären Hospiz, innerhalb der letzten drei Jahre oder aus einer mindestens einjährigen klinischen palliativmedizinischen Tätigkeit in einer Palliativabteilung in einem Krankenhaus innerhalb der letzten drei Jahre.

b) Pflegefachkräfte

- Erlaubnis zur Führung einer der Berufsbezeichnungen Gesundheits- und Krankenpflegerin/Gesundheits- und Krankenpfleger, Gesundheits- und Kinderkrankenpflegerin/Gesundheits- und Kinderkrankenpfleger, Altenpflegerin/Altenpfleger,
- Abschluss einer Palliativ-Weiterbildungsmaßnahme im Umfang von mindestens 160 Stunden, die vergleichbar mit dem Basis-Curriculum von Kern/Müller/Aurnhammer ist
  und
- eine nachweisbare mindestens zweijährige berufspraktische Tätigkeit innerhalb der letzten drei Jahre in der Palliativversorgung, davon mindestens sechsmonatige Mitarbeit in einer spezialisierten Einrichtung der Hospiz- und Palliativversorgung.

c) Koordinator/Koordinatoren (§ 6 Abs. 6 und § 9 Abs. 5)

- soweit es sich um einen Arzt oder eine Pflegefachkraft handelt, eine Qualifikation nach den Buchstaben a oder b oder eine nachweisbare mehrjährige Erfahrung in der Palliativversorgung
- soweit es sich um einen Angehörigen einer anderen geeigneten Berufsgruppe wie z.B. Sozialarbeiterinnen/Sozialarbeiter, Psychologinnen/Psychologen, Sozialpädagoginnen/Sozialpädagogen handelt, eine für diese Berufsgruppe geeignete Zusatz-Weiterbildung Palliative Care oder eine nachweisbare mehrjährige Erfahrung in der Palliativversorgung.

(2) Zur Sicherstellung der SAPV im Versorgungsraum XXXXXXXXX können durch das PCT weitere Leistungserbringer vertraglich eingebunden werden.

- Ärztinnen/Ärzte, die die Voraussetzungen gemäß § 7 Abs. 1 Buchst. a erfüllen
- zugelassene Pflegedienste mit Vertrag nach § 132 a SGB V zur Häuslichen Krankenpflege bzw. mit Versorgungsvertrag nach § 72 SGB XI zur ambulanten Pflege , die Pflegefachkräfte in einem sozialversicherungspflichtigen Beschäftigungsverhältnis angestellt haben, die die Voraussetzungen gemäß § 7 Abs. 1 Buchst. b erfüllen. Diese Pflegedienste haben zu garantieren, dass die Leistungen im Rahmen der SAPV ausschließlich durch diese qualifizierten Pflegefachkräfte erbracht werden.

(3) Soweit weitere Fachkräfte wie z. B. Sozialarbeiterinnen/Sozialarbeiter, Psychologinnen/Psychologen, Sozialpädagoginnen/Sozialpädagogen vertraglich eingebunden werden, haben diese eine Zusatz-Weiterbildung Palliative Care für andere Berufsgruppen oder eine mehrjährige Erfahrung in der Palliativversorgung nachzuweisen.

(4) Zur Sicherstellung der psychosozialen Unterstützung und zur allgemeinen palliativpflegerischen Beratung sind soweit möglich ambulante Hospizdienste gemäß § 39 a Abs. 2 SGB V im Versorgungsgebiet kooperativ einzubinden.

## § 8
## Sächliche Anforderungen

(1) Das PCT hat als Mindestanforderung an die sächliche Ausstattung Folgendes vorzuhalten bzw. sicher zu stellen:

- eine geeignete, aktuell geführte und für die an der Versorgung Beteiligten jederzeit zugängliche Patientendokumentation
- eine ausreichende und geeignete Mobilität zur zeitnahen häuslichen Versorgung der Patienten
- Arzneimittel (inkl. Betäubungsmittelgesetz – BtM –) für die Notfall/Krisenintervention
- Arzt-/Pflegekoffer/Bereitschaftstasche (Berücksichtigung der Kompatibilität der Verbrauchsmaterialien zu Medizinprodukten unterschiedlicher Hersteller, z. B. bei Portsystemen oder Infusionspumpen)
- eine geeignete administrative Infrastruktur, z. B. Büro, Kommunikationstechnik

(2) Das PCT muss über eine eigenständige Adresse mit eigener Telefonnummer und geeignete Räumlichkeiten für

- die Beratung von Patienten und Angehörigen,
- Teamsitzungen und Besprechungen,
- die Lagerhaltung von eigenen Medikamenten für Notfall-/Krisenintervention und Hilfsmitteln verfügen.

(3) Sofern eine Aufbewahrung von Medikamenten erfolgt, die unter das BtM fallen, ist ein BtM-Schrank erforderlich.

(4) Das PCT hat ein geeignetes, dem aktuellen Standard entsprechendes Pflegedokumentationssystem anzuwenden, das die übersichtliche und jederzeit nachvollziehbare Dokumentation der Stammdaten und des Pflegeprozesses in all seinen Schritten sowie eine Evaluation ermöglicht. Alle Eintragungen sind nachvollziehbar und eindeutig mit Handzeichen abzuzeichnen. Die Pflegedokumentation ist, von begründeten Ausnahmefällen abgesehen, beim Versicherten aufzubewahren.

## § 9
## Aufgaben des PCT

(1) Das PCT wird beratend, koordinierend und behandelnd tätig, wenn spezielle palliativmedizinische Kenntnisse bzw. spezielle palliativpflegerische Kenntnisse für die Versorgung erforderlich sind. Das PCT arbeitet eng mit den die betreffenden Versicherten im Rahmen der Regelversorgung betreuenden Vertragsärzten, den Pflegediensten und weiteren Kooperationspartnern zusammen.

(2) Das PCT erstellt für jeden zu versorgenden Versicherten einen individuellen Behandlungsplan, der mit den übrigen an der Versorgung beteiligten Leistungserbringern abzustimmen ist.

(3) Die ständige Verfügbarkeit mindestens einer Palliativärztin/eines Palliativarztes und einer Palliativpflegefachkraft ist zu gewährleisten. Die Verfügbarkeit umfasst erforderlichenfalls auch das umgehende Aufsuchen des Versicherten. Eine Hilfsfrist von maximal 120 Minuten ist anzustreben.

(4) Durch den hohen Grad der Erreichbarkeit und fachlicher Kompetenz muss das PCT in dem Versorgungsraum eine reibungslos funktionierende Schnittstelle zwischen stationärem und ambulantem Sektor darstellen.

(5) Die Koordinationsstelle übernimmt sowohl eine koordinierende als auch beratende Funktion. Sie ist erste Ansprechpartnerin. Es wird gewährleistet, dass die an der Versorgung beteiligten Leistungserbringer die erforderlichen Maßnahmen aufeinander abgestimmt und bedarfsgerecht erbringen und dass zwischen den an der Versorgung des Versicherten beteiligten Leistungserbringern zeitnah alle wichtigen Informationen über die vorhergehende Behandlung unter Berücksichtigung datenschutzrechtlicher Regelungen ausgetauscht werden.

(6) Die Dokumentation der versichertenbezogenen Leistungen durch die an der Versorgung beteiligten Leistungserbringer/Personen ist entsprechend der jeweiligen Berufsordnung sicherzustellen. Das PCT verfügt aufgrund eines einheitlichen Patientendokumentationssystems zu jeder Zeit über die notwendigen Informationen zum Versicherten.

(7) Die im PCT mitwirkenden Ärzte und Pflegefachkräfte nehmen an palliativmedizinischen bzw. palliativpflegerischen Fortbildungen in einem Umfang von 20 Zeitstunden (entsprechend 20 Punkten im Rahmen der ärztlichen Fortbildung) innerhalb von zwei Kalenderjahren teil.

(8) Das PCT stellt sicher, dass die notwendigen krankenpflegerischen Informationen auf der Grundlage der Pflegedokumentation (Pflegeüberleitungsbogen) bei Einweisung des Versicherten in ein Krankenhaus oder eine andere Einrichtung diesen sowie dem Vertragsarzt zur Verfügung gestellt werden.

(9) Das PCT berücksichtigt bei der Planung und Durchführung der spezialisierten Palliativversorgung den vom Krankenhaus bei der Entlassung des Versicherten erstellten Bericht oder Überleitungsbogen.

(10) Ist nach Auffassung des PCT der Versicherte erheblich pflegebedürftig, hat aber noch keinen Antrag auf Leistungen der Pflegeversicherung gestellt, wirkt das PCT auf die erforderliche Antragstellung hin.

## § 10
## Qualitätssicherung

(1) Das PCT verpflichtet sich interne Qualitätssicherungsmaßnahmen durchzuführen. Das PCT ist für die Qualität seiner Leistungen verantwortlich.

(2) Das interne Qualitätsmanagement umfasst alle Managementprozesse (Verantwortung der Leitung, Ressourcenmanagement, Leistungserstellung, Analyse/Verbesserung), die Qualität entwickeln, festlegen und sichern. Es bezieht sich auf alle Handlungen und Leistungen, die einer zielorientierten, fachgerechten und effektiven Leistungserbringung dienen. Die Verantwortlichkeit für das PCT-interne Qualitätsmanagement liegt auf der Leitungsebene des PCT´s, und zwar auch dann, wenn ein Qualitätsmanagementbeauftragter benannt ist.

(3) Das PCT ist bereit, das Dokumentationssystem daraufhin auszulegen, dass patientenbezogene Daten und – soweit vorhanden – allgemein anerkannte Indikatoren für eine externe Qualitätssicherung berücksichtigt werden.

(4) Maßnahmen des PCT zur internen Qualitätssicherung:
   a) Das PCT ist dafür verantwortlich, dass Maßnahmen zur internen Sicherung der Struktur-, Prozess- und Ergebnisqualität festgelegt und durchgeführt werden.
   b) Die Durchführung der Qualitätssicherung wird vom PCT dokumentiert und aufbewahrt.
   c) Das PCT hat die Durchführung von und die Beteiligung an Qualitätssicherungsmaßnahmen auf Anforderung der Krankenkasse nachzuweisen.
   d) Das PCT hat die Teilnahme an der Supervision zu ermöglichen und regelmäßige multiprofessionelle Fallbesprechungen durchzuführen.

(5) Das PCT soll sich an Maßnahmen der externen Qualitätssicherung beteiligen.

(6) Verfahren zur Durchführung von Qualitätsprüfungen:
Wird von der Krankenkasse die Notwendigkeit einer anlassbezogenen Qualitätsprüfung als gegeben angesehen ist sie berechtigt, die Qualität der Leistungserbringung des PCT durch den MDK oder andere Sachverständige überprüfen zu lassen.

## § 11
## Vergütung und Abrechnung

(1) Die Vertragspartner vereinbaren für Leistungen der SAPV eine pauschalierte Vergütungsregelung. Für diese Leistungen kann daneben keine anderweitige Vergütung in Ansatz gebracht werden.
Näheres dazu wird in Anlage 2 bestimmt.

(2) Das PCT stellt den Krankenkassen versichertenbezogen die erbrachten Leistungen gemäß Anlage 3 in Rechnung. Leistungsbegründende Unterlagen sind nach Anlage 4 beizufügen.

(3) Die Krankenkassen vergüten die Leistungen innerhalb von vier Wochen nach Rechnungseingang bei der Krankenkasse.

(4) Für die Abrechnung wird mittelfristig die Anwendung der Richtlinien der Spitzenverbände der Krankenkassen, jetzt Spitzenverband Bund, über Form und Inhalt des Abrechnungsverfahrens mit sonstigen Leistungserbringern nach § 302 Abs. 2 SGB V in der jeweils aktualisierten Fassung angestrebt. Näheres wird zu gegebener Zeit zwischen PCT und Krankenkassen vereinbart.

## § 12
## Verordnung von Arznei-, Heil- und Hilfsmitteln

(1) Die Verordnung von Arznei-, Heil- und Hilfsmitteln im Rahmen der SAPV erfolgt unter Verwendung der für die vertragsärztliche Versorgung geltenden Vordrucke (Muster 13, 14, 16 und 18). Bei der (zeitgleichen) Verordnung von Arzneimitteln einerseits und Hilfsmitteln andererseits sind jeweils getrennte Vordrucke nach Muster 16 zu verwenden. Die Verordnungen sind auf Basis der geltenden Richtlinien des G-BA auszuführen. Das Wirtschaftlichkeitsgebot nach SGB V ist zu beachten. Für die verordneten Arznei-, Heil- und Hilfsmittel gelten die nach §§ 300, 302 SGB V vereinbarten Abrechnungsverfahren entsprechend.

(2) Jeder Vertragspartner einer Krankenkasse nach § 132 d Abs. 1 SGB V (PCT) erhält eine Betriebsstätten-Nummer, die die Zuordnung von Verordnungen zu diesem gewährleistet. Die Betriebsstätten-Nummer ist bei Verordnungen immer in das vorgesehene Feld auf den Vordrucken einzutragen. Die Vergabe der Betriebsstätten-Nummer erfolgt durch die Kassenärztliche Bundesvereinigung auf Antrag des PCT. Der Antrag ist mit dem beigefügten Mustervordruck (Anlage 5) zu stellen. Der Leistungserbringer hat die Krankenkassen über die von der KBV vergebene Betriebsstätten-Nummer zu informieren.

(3) Bei der Verordnung von Arznei-, Heil- und Hilfsmitteln im Rahmen der SAPV wird auf den zu verwendenden Vordrucken im entsprechenden Feld einheitlich die Pseudo-Arzt-Nummer 333 333 300 eingetragen.
Auf jeder Verordnung im Rahmen dieser Vereinbarung sind die Betriebsstätten-Nummer und die Pseudo-Arztnummer anzugeben.

(4) Die Krankenkassen teilen dem PCT mit, wo die Vordrucke bezogen werden können.

## § 13
## Datenschutz / Schweigepflicht

(1) Die Vertragspartner sind verpflichtet, die gesetzlichen Bestimmungen zur ärztlichen Schweigepflicht und zum Datenschutz, insbesondere den Schutz der Sozialdaten (SGB X 2. Kapitel) zu beachten. Personenbezogene Daten sind nur zur Erfüllung der sich aus diesem Vertrag ergebenden Aufgaben zu verarbeiten, bekannt zu geben, zugänglich zu machen oder sonst zu nutzen.

(2) Der Leistungserbringer hat die im Team tätigen Mitarbeiter zur Beachtung der Datenschutzbestimmungen und der Schweigepflicht zu verpflichten. Die Verpflichtung ist zu dokumentieren.

## § 14
## Sonstige Bestimmungen

(1) Die Vertragspartner werden die Versorgungsabläufe nach diesem Vertrag konstruktiv beobachten und sind bereit, erforderliche Anpassungen aufgrund der Praxiserfahrungen bei neuen Vertragsverhandlungen vorzunehmen.

(2) Die Vertragspartner werden gemeinsam prüfen, inwieweit die Ziele nach § 1 Abs. 1 erreicht worden sind.

## § 15
## Inkrafttreten/Kündigung

(1) Dieser Vertrag tritt zum XX.XX.XXXX in Kraft.

(2) Er kann ordentlich von jedem Vertragspartner mit einer Frist von sechs Monaten zum Quartalsende gekündigt werden. Die Mindestlaufzeit des Vertrags beträgt ein Jahr.

(3) Das Recht zur außerordentlichen Kündigung dieses Vertrages aus wichtigem Grund bleibt unberührt.

(4) Wirken Rechtsänderungen auf die Inhalte dieses Vertrages, treten die Vertragsparteien unverzüglich in Verhandlungen, ohne dass es einer Kündigung bedarf.

## § 16
## Salvatorische Klausel

(1) Sollten einzelne Bestimmungen dieses Vertrages unwirksam oder undurchführbar sein oder werden, bleibt sie im Übrigen dennoch gültig, es sei denn, die unwirksame Bestimmung war für eine Vertragspartei derart wesentlich, dass ihr ein Festhalten an der Vertrag nicht zugemutet werden kann. In allen anderen Fällen werden die Vertragsparteien die unwirksame Bestimmung durch Regelungen ersetzen, die dem ursprünglichen Regelungsziel unter Beachtung sonstiger rechtlicher Vorgaben am nächsten kommt.

(2) Erweist sich der Vertrag als lückenhaft, sind die Vertragsparteien verpflichtet, sie unter Beachtung der erkennbaren Zielsetzung und der sonstigen rechtlichen Vorgaben zu ergänzen.

Stuttgart, Kornwestheim, Ludwigsburg, München, XXX, den XX.XX.XXXX

Verantwortlicher PCT

AOK Baden-Württemberg

Verband der Ersatzkassen e. V. (vdek),
der Leiter der vdek-Landesvertretung
Baden-Württemberg

Vorsitzender / Stv. Vorsitzender
BKK-Vertragsarbeitsgemeinschaft
Baden-Württemberg,
vertreten durch die Vorsitzenden
der Mitgliederversammlung

IKK classic,
vertreten durch den Vorsitzenden
des Vorstandes

Landwirtschaftliche Krankenkasse
Baden-Württemberg

Knappschaft
Regionaldirektion München

## Protokollnotiz

### 1. Übergangsregelungen für Qualifikationsvoraussetzungen nach § 7 Abs. 1 des Vertrages nach § 132d Abs. 1 i.V.m. § 37b SGB V der Leistungserbringer SAPV / Mitarbeiter PCT

Gültig für neu gegründete Palliative-Care-Teams in den ersten drei Jahren nach Abschluss eines Vertrages nach § 132d Abs. 1 i.V.m. § 37b SGB V.

Nach Ablauf der Übergangsregelung müssen Mitarbeiter des Palliative-Care-Teams, die SAPV-Leistungen erbringen, grundsätzlich alle Qualifikationsvoraussetzungen nach § 7 Abs. 1 des Vertrages nach § 132d Abs. 1 i.V.m. § 37b SGB V erfüllen.

Ab Vertragsabschluss

- muss mindestens eine vollbeschäftigte Pflegefachkraft oder zwei teilzeitbeschäftigte Pflegefachkräfte mit einem Beschäftigungsumfang von insgesamt 100 % alle Voraussetzungen gemäß § 7 Abs. 1 Buchst. b des Vertrages nach § 132d Abs. 1 i.V.m. § 37b SGB V erfüllen
- muss mindestens ein Arzt/eine Ärztin alle Voraussetzungen gemäß § 7 Abs. 1 Buchst. a des Vertrages nach § 132d Abs. 1 i.V.m. § 37b SGB V erfüllen
- sind Abwesenheitszeiten wie Urlaub, Tagungsteilnahme oder Krankheit durch qualitativ gleichwertige Ärzte / Pflegefachkräfte abzudecken.

Für die übrigen Mitarbeiter gilt:

Weiterbildungsmaßnahmen:
Qualifizierungsmaßnahmen zur Erreichung der Qualifikationsvoraussetzungen nach § 7 Abs. 1 des Vertrages nach § 132d Abs. 1 i.V.m. § 37b SGB V (Palliativ-Weiterbildungsmaßnahme für Pflegekräfte bzw. Zusatzweiterbildung Palliativmedizin) müssen bei Vertragsabschluss nachweislich begonnen sein. Das voraussichtliche Datum des Abschlusses der Weiterbildung muss benannt werden.

Es sind folgende berufspraktische Erfahrungen in der Palliativversorgung nachzuweisen:

- Pflegefachkräfte:
  Eine Vollzeit-Tätigkeit im Umfang von mindestens 5 Wochen in einer spezialisierten Einrichtung der Hospiz- und Palliativversorgung (Palliativstation, stationäres Hospiz, spezieller Palliativpflegedienst) innerhalb der letzten fünf Jahre vor Vertragsabschluss.

- Ärzte/Ärztinnen:
  Die Behandlung von mindestens 40 Palliativpatienten/innen ambulant oder stationär oder alternativ eine klinische palliativmedizinische Vollzeit-Tätigkeit im Umfang von mindestens 10 Wochen innerhalb der letzten fünf Jahre vor Vertragsabschluss.

Bei Teilzeitbeschäftigung verlängert sich die qualifizierende Beschäftigungszeit entsprechend.

Sofern die oben genannten Mindestvoraussetzungen für die berufspraktische Erfahrung erfüllt sind, kann die zur Erfüllung der Voraussetzungen gemäß § 7 Abs. 1 des Vertrages nach § 132d Abs. 1 i.V.m. § 37b SGB V noch fehlende berufspraktische Erfahrung im Rahmen der Arbeit im PCT erworben werden. Dabei muss das Tätigwerden unter der fachlichen Verantwortung eines voll ausgebildeten Mitarbeiters der entsprechenden Berufsgruppe des PCT stehen.

Unter Berücksichtigung dieser Rahmenbedingungen wird für jedes PCT eine individuelle Übergangsregelung angestrebt.

Die Vertragspartner erklären einvernehmlich, spätestens nach Ablauf von 2 ½ Jahren nach Inkrafttreten dieser Vereinbarung Gespräche auf der Landesebene aufzunehmen, um gemeinsam die Notwendigkeit ggf. erforderlicher Anpassungen der Übergangsregelung zu prüfen.

**2. Erfüllung der Qualifikation nach § 7 Abs. 1 Buchst. a des Vertrages nach § 132d Abs. 1 i.V.m. § 37b SGB V**

Einem Arzt/einer Ärztin mit der Zusatzbezeichnung Palliativmedizin gleichgestellt wird ein Arzt/eine Ärztin, der/die in den letzten 5 Jahren mindestens 3 Jahre eine palliativmedizinische Tätigkeit ausgeübt hat und an der Weiterbildung Palliativmedizin (40 Stunden Kurs-Weiterbildung in Palliativmedizin und 120 Stunden Fallseminar einschl. Supervision, letzteres ersetzbar durch 12 Monate Weiterbildungszeit in einer ausbildungsberechtigten Weiterbildungsstätte) nachweislich vollständig und erfolgreich teilgenommen hat, der/die aber wegen des Fehlens einer Facharztanerkennung nicht die Zusatzbezeichnung erwerben kann.

Stuttgart, Kornwestheim, Ludwigsburg, München, XXX, den XX.XX.XXXX

Unterschriftsblatt zur Protokollnotiz
Übergangsregelungen für Qualifikationsvoraussetzungen nach § 7 Abs. 1 gemäß Ziffer 1 und Erfüllung der Qualifikation nach § 7 Abs. 1 Buchst. a gemäß Ziffer 2
zum SAPV-Vertrag mit XXX

______________________________
Verantwortlicher PCT

| | |
|---|---|
| AOK Baden-Württemberg | Verband der Ersatzkassen e. V. (vdek),<br>der Leiter der vdek-Landesvertretung<br>Baden-Württemberg |
| Vorsitzender / Stv. Vorsitzender<br>BKK-Vertragsarbeitsgemeinschaft<br>Baden-Württemberg,<br>vertreten durch die Vorsitzenden<br>der Mitgliederversammlung | IKK classic,<br>vertreten durch den Vorsitzenden<br>des Vorstandes |
| Landwirtschaftliche Krankenkasse<br>Baden-Württemberg | Knappschaft<br>Regionaldirektion München |

Anlage 1

## SAPV-Strukturerhebungsbogen

Voraussetzung für einen Vertragsabschluss ist die Vorlage eines verbindlichen, strukturierten und schriftlich dargelegten Konzeptes, in dem der inhaltliche und organisatorische Rahmen der Leistungserbringung sowie die Einbindung in die regionale Versorgungsstruktur beschrieben sind. Das Konzept und der Strukturerhebungsbogen ist den Krankenkassen vor Vertragsabschluss einzureichen.

Es muss eine Beschreibung folgender Inhalte vorliegen:

- Bevölkerungsbezogene Aussagen zum Versorgungsraum
- Aufbauorganisation des Palliative-Care-Teams.
  Die eigenständige inhaltliche und organisatorische Struktur des PCT muss ersichtlich sein, ebenso das Verhältnis zu den vertraglich eingebundenen Kooperationspartnern
- Aufgabenverteilung und Verantwortlichkeiten innerhalb des PCT und zwischen PCT und Kooperationspartnern
- Ablauforganisation / Versorgungsprozess,
  Sicherstellung der Kommunikation innerhalb des Teams und mit den Kooperationspartnern zur Abstimmung von Behandlungsplänen,
  Team- und Fallbesprechungen
- Präsenzzeiten der Koordinationsstelle
- Sicherstellung der Versorgung mit Arznei-, Heil- und Hilfsmitteln
- Personelle Ausstattung, Qualifikationsnachweise
- Sächliche und räumliche Ausstattung (gemäß der Gemeinsamen Empfehlungen nach § 132 d Abs. 2 SGB V für die SAPV – 23.06.2008)
- Qualitätssicherung,
  Qualitätsmanagement, Einarbeitungs- und Fortbildungskonzepte, Supervision, Dokumentationssystem

In Ihrem Konzept sind die Schnittstellen zwischen den ggf. vertraglich eingebundenen Kooperationspartnern nach § 7 Abs. 2 und 3 des Vertrages nach § 132d Abs. 1 i.V.m. § 37b SGB V und dem PCT zu beschreiben.

Bitte legen Sie Qualifikationsnachweise für die im Palliative-Care-Team vorgesehenen Fachkräfte und Teammitglieder vor.

Die nachfolgenden Ausführungen und Fragestellungen sollen die Erstellung bzw. die Bewertung eines Konzeptes vereinfachen. Bitte übersenden Sie daher den Vertragspartnern auf Krankenkassenseite eine ausführliche Konzeption und den nachfolgenden SAPV-Strukturerhebungsbogen ausgefüllt zurück.

## 1. Anbieter der SAPV

Name des Dienstes: ______________________

Straße, Hausnummer: ______________________

PLZ, Ort: ______________________

Ansprechpartner: ______________________

Emailadresse: ______________________

Telefonnummer: ______________________

Faxnummer: ______________________

Name des Trägers: ______________________

Straße, Hausnummer: ______________________

PLZ, Ort: ______________________

Ansprechpartner: ______________________

Emailadresse: ______________________

Telefonnummer: ______________________

Faxnummer: ______________________

## 2. Versorgungsbedarf

2.1 Welche Versorgungsregion wollen Sie versorgen?

2.2 Wie hoch ist die Einwohnerzahl Ihrer potentiellen Versorgungsregion?

2.3 Gibt es Besonderheiten in Ihrer Region?

## 3. Personelle Anforderungen

Das PCT verfügt über das für die spezialisierte ambulante Palliativversorgung erforderliche und geeignete Personal. Dabei wird berücksichtigt, dass durch das PCT eine tägliche telefonische Rund-um-die-Uhr-Erreichbarkeit für die Patienten, deren Angehörigen und die an der Versorgung Beteiligten sichergestellt wird. Die ständige Verfügbarkeit mindestens einer Palliativärztin/eines Palliativarztes und einer Palliativpflegefachkraft wird gewährleistet. Die Verfügbarkeit schließt notwendige Hausbesuche ein.

## 3.1 Ärztinnen und Ärzte

Über welche Qualifikationen gemäß § 7 Abs. 1 Buchst. a verfügen die Ärzte für das geplante PCT?

| | | | | |
|---|---|---|---|---|
| **Name** | | | | |
| **Vorname** | | | | |
| **Anteil einer Vollzeitstelle** | % | % | % | % |
| **Andere Beschäftigung, Wo** | | | | |
| **Zusatzbezeichnung Palliativmedizin ja/nein** | | | | |
| **Nachweis mind. 75 Palliativpatienten in letzten drei Jahren ja/nein** | | | | |
| **Mind. 1 Jahr Tätigkeit Palliativabteilung ja/nein** | | | | |
| **Gesamtvoraussetzungen erfüllt ja/nein** | | | | |
| **Voraussetzungen voraussichtlich erfüllt bis** | | | | |
| **Bemerkungen** | | | | |

## 3.2 Pflegefachkräfte

Über welche Qualifikationen gemäß § 7 Abs. 1 Buchst. b verfügen die Pflegekräfte für das geplante PCT?

| | | | | |
|---|---|---|---|---|
| **Name** | | | | |
| **Vorname** | | | | |
| **Anteil einer Vollzeitstelle** | % | % | % | % |
| **Andere Beschäftigung, Wo?** | | | | |
| **Weiterbildung Palliative Care ja/nein** | | | | |
| **2-jährige Tätigkeit in Palliativversorgung in letzten drei Jahren ja/nein** | | | | |
| **Mind. 6 Monate Tätigkeit in spez. Palliativeinrichtung ja/nein** | | | | |
| **Gesamtvoraussetzungen erfüllt Ja/nein** | | | | |
| **Voraussetzungen erfüllt bis** | | | | |
| **Bemerkungen** | | | | |

Über welche Qualifikationen gemäß § 7 Abs. 1 Buchst. b verfügen die Pflegekräfte für das geplante PCT?

| | | | | |
|---|---|---|---|---|
| **Name** | | | | |
| **Vorname** | | | | |
| **Anteil einer Vollzeitstelle** | % | % | % | % |
| **Andere Beschäftigung, Wo?** | | | | |
| **Weiterbildung Palliative Care ja/nein** | | | | |
| **2-jährige Tätigkeit in Palliativversorgung in letzten drei Jahren ja/nein** | | | | |
| **Mind. 6 Monate Tätigkeit in spez. Palliativeinrichtung ja/nein** | | | | |
| **Gesamtvoraussetzungen erfüllt ja/nein** | | | | |
| **Voraussetzungen erfüllt bis** | | | | |
| **Bemerkungen** | | | | |

## 3.3 Koordinatorin/Koordination

Über welche Qualifikationen gemäß § 7 Abs. 1 Buchst. c verfügen die im geplanten PCT tätige/n Koordinatorin/Koordinator/en?

| | | |
|---|---|---|
| **Name** | | |
| **Vorname** | | |
| **Berufsbezeichnung** | | |
| **Anteil einer Vollzeitstelle** | % | % |
| **Andere Beschäftigung, Wo?** | | |
| **Weiterbildung Palliative Care ja/nein** | | |
| **2-jährige Tätigkeit in Palliativbetreuung in letzten drei Jahren ja/nein** | | |
| **Mind. 6 Monate Tätigkeit in spez. Palliativeinrichtung ja/nein** | | |
| **Mehrjährige Erfahrung in Palliativversorgung ja/nein** | | |
| **Gesamtvoraussetzungen erfüllt ja/nein** | | |
| **Voraussetzungen erfüllt bis** | | |
| **Bemerkungen** | | |

## 4. Kooperationspartner gemäß § 7 Abs. 2

Zur Sicherstellung der SAPV können durch das PCT weitere Leistungserbringer bzw. Fachkräfte mit entsprechender fachlicher Qualifikation eingebunden werden.

| Name der Einrichtung | Ansprechpartner | Pflegedienst / Arzt/Ärztin |
|---|---|---|
| | | |
| | | |
| | | |
| | | |
| | | |
| | | |
| | | |
| | | |

## 5. Kooperationspartner gemäß § 7 Abs. 3 und 4

Weitere Leistungserbringer, Fachkräfte oder sonstige Dienste können vertraglich eingebunden werden.

| Name der Einrichtung | Ansprechpartner | Psychologinnen / Psychologe, Sozialarbeiterinnen / -arbeiter, Sozialpädagoginnen / -pädagoge, ambulanter Hospizdienst |
|---|---|---|
| | | |
| | | |
| | | |
| | | |
| | | |

## 5. Sächliche Ausstattung

5.1 Welche Räumlichkeiten stehen zur Verfügung?

5.2 Welche administrative Infrastruktur steht zur Verfügung?

5.3 Anzahl der Dienstfahrzeuge?

5.4 Stehen Arzneimittel für die Notfall/Krisenintervention zur Verfügung?

5.5 Steht ein BTM-Schrank zur Verfügung?

5.6 Steht ein Arzt-/Pflegekoffer/Bereitschaftstasche zur Verfügung?

## 6. Qualitätssicherung

6.1 Wie wird die interne Qualitätssicherung (Struktur- Prozess- und Ergebnisqualität) umgesetzt? Beschreibung der Maßnahmen

______________________________________________________________________

______________________________________________________________________

______________________________________________________________________

______________________________________________________________________

______________________________________________________________________

______________________________________________________________________

______________________________________________________________________

______________________________________________________________________

6.2 Bitte nennen bzw. beschreiben Sie Ihr Dokumentationssystem

______________________________________________________________________

______________________________________________________________________

______________________________________________________________________

______________________________________________________________________

______________________________________________________________________

6.3 Wie oft finden Teambesprechungen statt?

______________________________________________________________________

______________________________________________________________________

6.4 Wie oft findet Supervision statt?

______________________________________________________________________

______________________________________________________________________

6.5 Wie oft finden multiprofessionelle Fallbesprechungen / Qualitätszirkel statt?

______________________________________________________________________

______________________________________________________________________

6.6 Liegt ein Einarbeitungskonzept vor?

______________________________________________________________________

6.7 Liegt ein Fortbildungskonzept vor?

______________________________________________________________________

Ich bestätige die Richtigkeit der gemachten Angaben.

______________________________________________________________________

Ort, Datum Unterschrift des verantwortlichen Vertreters des PCT

Anlage 2

## Vergütungsvereinbarung

### 1. Allgemeines

Für die Leistungen im Rahmen der SAPV können ausschließlich die in dieser Vergütungsvereinbarung ausgewiesenen Pauschalen abgerechnet werden. Für die SAPV-Leistungen können dann daneben keine anderweitigen Vergütungen in Ansatz gebracht werden.

### 2. Vollständige Versorgung / additiv unterstützende Versorgung

2.1 für SAPV-Leistungen außerhalb eines stationären Hospizes:

| | |
|---|---|
| a) Festbetrag bei einer Versorgungsdauer bis zu 7 Tagen, auch wenn die Versorgungsdauer weniger als 7 Tage beträgt: | **1260,00 EUR** |
| b) Tagespauschale ab dem 8. Versorgungstag, abrechenbar an Tagen, an denen ein Hausbesuch stattfindet: | **150,00 EUR** |

2.2 für SAPV-Leistungen innerhalb eines stationären Hospizes:

| | |
|---|---|
| a) Festbetrag bei einer Versorgungsdauer bis zu 7 Tagen, auch wenn die Versorgungsdauer weniger als 7 Tage beträgt: | **560,00 EUR** |
| b) Tagespauschale ab dem 8. Versorgungstag, abrechenbar an Tagen, an denen ein Einsatz im Hospiz stattfindet: | **80,00 EUR** |

2.3 Während einer „vollständigen Versorgung“ oder einer „additiv unterstützenden Versorgung“ ist eine Erstkoordinationspauschale bzw. Erstberatungspauschale nicht abrechenbar. An Tagen, an denen kein Festbetrag oder keine Tagespauschale abgerechnet werden kann, aber eine Beratung oder Koordination stattgefunden hat, ist die Folgeberatungspauschale bzw. Folgekoordinationspauschale abrechenbar.

2.4 Hinweis: In stationären Hospizen kann im Rahmen der SAPV nur die ärztliche Versorgung vergütet werden.

2.5 Die medizinisch/pflegerischen Regelleistungen nach SGB V werden separat vergütet. Dies betrifft beispielsweise die Leistungen nach §§ 37 und 38 SGB V sowie vertragsärztliche Leistungen.

2.6 Bei mehr als vier Wochen Unterbrechung der spezialisierten ambulanten Palliativversorgung des Versicherten kann bei erneutem Beginn einer vollständigen Versorgung/additiv unterstützenden Versorgung wiederum der unter 2.1 a) bzw. 2.2 a) angegebene Festbetrag abgerechnet werden.
Dies gilt nicht bei Unterbrechung aufgrund stationären Krankenhaus- und stationären Reha-Aufenthalten.

## 3. Beratung

| | | | |
|---|---|---|---|
| a) | Erstberatungspauschale<br>als Eingangsberatung/Bestandsaufnahme | | **200,00 EUR** |
| b) | Folgeberatungspauschale | pro Beratung<br>maximal pro Kal.Tag | **25,00 EUR**<br>**50,00 EUR** |

Zu a): Neben der Erstberatungspauschale kann eine Erstkoordinationspauschale nach Ziffer 4 a) nicht abgerechnet werden.

Zu b): Neben der Folgeberatungspauschale ist ggf. auch eine Folgekoordinationspauschale nach Ziffer 4 b) abrechenbar.

## 4. Koordination

a) Erstkoordinationspauschale als Eingangskoordination/Bestandsaufnahme **200,00 EUR**

kann nur abgerechnet werden, wenn keine Erstberatungspauschale nach Ziffer 3 a) in Ansatz gebracht wird.

b) Folgekoordinationspauschale **100,00 EUR**

abrechenbar einmal pro Kalenderwoche.

Neben der Folgekoordinationspauschale ist ggf. auch eine Folgeberatungspauschale nach Ziffer 3 b) abrechenbar.

## 5. Sonstiges

a) Arzneimittel, Heilmittel und Hilfsmittel sind mit der Vergütung der Leistung der SAPV nicht abgegolten und werden gesondert abgerechnet.

b) Mit diesen Preisen sind auch Fahrtkosten abgegolten.

c) Wird eine Verordnung zu SAPV von der Krankenkasse nicht genehmigt, kann ein Festbetrag nach Ziffer 2.1 a) bzw. 2.2 a), eine Erstberatungspauschale nach Ziffer 3 a) und eine Erstkoordinationspauschale nach Ziffer 4 a) nicht abgerechnet werden.
In solchen Fällen sind vom 1. Tag bis zum Tag der Ablehnung lediglich die einsatzbezogenen Pauschalen nach Ziffer 2.1 b) bzw. 2.2 b) und ggf. die Folgeberatungspauschale nach Ziffer 3 b) und die Folgekoordinationspauschale nach Ziffer 4 b) abrechenbar.

d) Eine Erstberatungspauschale nach Ziffer 3 a) bzw. eine Erstkoordinationspauschale nach Ziffer 4 a) kann abgerechnet werden, wenn sich im Rahmen der Bestandsaufnahme herausstellt, dass eine vollständige Versorgung oder additiv unterstützende Versorgung nicht erforderlich ist.

Die Vertragspartner werden die Vergütungsvereinbarung nach einer Laufzeit von einem Jahr gemeinsam überprüfen und erklären ihre Bereitschaft, erforderliche Anpassungen aufgrund der Praxiserfahrung vorzunehmen.

Diese Vereinbarung tritt ab xx.xx.xxxx in Kraft und gilt für mindestens ein Jahr. Sie kann ordentlich von jedem Vertragspartner mit einer Frist von drei Monaten zum Quartalsende gekündigt werden.

Wird die Vereinbarung gekündigt, gelten die Preise dieser Vereinbarung bis zu einer Neuregelung weiter.

Stuttgart, Kornwestheim, Ludwigsburg, München, XXX, den XX.XX.XXXX
Unterschriftsblatt zur Vergütungsvereinbarung zum SAPV-Vertrag mit XXX

_______________________________
Verantwortlicher PCT

_______________________________
AOK Baden-Württemberg

_______________________________
Verband der Ersatzkassen e. V. (vdek),
der Leiter der vdek-Landesvertretung
Baden-Württemberg

_______________________________
Vorsitzender / Stv. Vorsitzender
BKK-Vertragsarbeitsgemeinschaft
Baden-Württemberg,
vertreten durch die Vorsitzenden
der Mitgliederversammlung

_______________________________
IKK classic,
vertreten durch den Vorsitzenden
des Vorstandes

_______________________________
Landwirtschaftliche Krankenkasse
Baden-Württemberg

_______________________________
Knappschaft
Regionaldirektion München

Anlage 3

## Rechnungsblatt

Mit der Rechnungsstellung müssen folgende Inhalte übermittelt werden:

a) die Summe der im Abrechnungszeitraum erbrachten Leistungen nach Art, Menge und Preis,
b) die IK des Leistungserbringers, ggf. die Betriebsstättennummer sowie die IK des Zahlungsempfängers,
c) den Namen und die Versichertennummer des Leistungsempfängers.

Ferner die im allgemeinen Zahlungsverkehr darüber hinaus erforderlichen Angaben (Name und Anschrift des Leistungserbringers, Rechnungsdatum und -nummer sowie Angaben zur Umsatzsteuer, Bankverbindung).

Als Anlage ist der Leistungsnachweis gemäß Anlage 4 beizufügen.

**- Muster (Beispiel) -**

**Rechnungsnummer:** **Datum:**
**IK Leistungserbringer:** **IK Zahlungsempfänger (sofern abweichend):**
**Betriebsstättennummer:**
**Bankverbindung:**

☐ **Erstabrechnung** ☐ **Zwischenabrechnung** ☐ **Endabrechnung**

**Name, Vorname:** **Geb.datum:**
**KV-Nummer:**

**Leistungszeitraum:**

Folgende Pauschalen werden für den oben genannten Leistungszeitraum abgerechnet:

| **erbrachte Leistung, Ziffer** | **Anzahl** | **Gesamtpreis** |
|---|---|---|
| Festbetrag 2.1 a | | |
| Festbetrag 2.2 a | | |
| Tagespauschale 2.1 b | | |
| Tagespauschale 2.2 b | | |
| Erstberatungspauschale 3 a | | |
| Folgeberatungspauschale 3 b | | |
| Erstkoordinationspauschale 4 a | | |
| Folgekoordinationspauschale 4 b | | |
| **Gesamt** | | |

Ein Leistungsnachweis für diesen Zeitraum liegt dieser Abrechung bei.

Anlage 4

## Leistungsnachweis (SAPV)

Die erbrachten Leistungen sind vom PCT jeweils mit der Rechnungsstellung zu bestätigen.

Der Leistungsnachweis muss folgende Mindestinhalte aufweisen:

- Zeitraum
- Krankenkasse des Leistungsempfängers
- Name, Vorname des Leistungsempfängers
- KV-Nummer des Leistungsempfängers
- Geburtsdatum des Leistungsempfängers
- IK des Leistungserbringers
- Betriebsstättennummer des Leistungserbringers
- Tabellarische Übersicht der erbrachten Leistungen je Kalendertag

Beispiel:

| Datum | Hausbesuch | Beratung | Koordination |
|---|---|---|---|
| | | | |
| | | | |
| | | | |

- Unterschriftenzeile PCT:

  „Ich bestätige die vertragsgemäße Ausführung der oben angegebenen Leistungen“
  - Datum
  - Unterschrift Verantwortlicher Leistungserbringer PCT
- Unterschriftenzeile Versicherter / Bezugsperson:

  „Ich bestätige die Durchführung der oben angegebenen Hausbesuche“
  - Datum
  - Unterschrift Versicherter / Bezugsperson

Anlage 5

## Antrag auf Vergabe einer SAPV-spezifischen Betriebsstättennummer für Leistungserbringer nach § 132 d Abs. 1 SGB V

### Leistungserbringer nach § 132 d Abs. 1 SGB V

Name: ........................................................
Ansprechpartner: ........................................................
Anschrift: ........................................................
........................................................
Telefon: ........................................................
Fax: ........................................................
E-Mail: ........................................................
Vertragsbeginn: ........................................................

............................................ ........................................................
Ort, Datum Unterschrift des Leistungserbringers

### Bescheinigung der Antragsberechtigung

Krankenkasse: ........................................................
Name des Ansprechpartners: ........................................................
Anschrift: ........................................................
........................................................
Telefon: ........................................................
Fax: ........................................................
E-Mail: ........................................................

............................................ ........................................................
Ort, Datum Unterschrift der Krankenkasse

............................................
Stempel der Krankenkasse

Anlage 6

## Liste der Betriebskrankenkassen, die Mitglieder der BKK-Vertragsarbeitsgemeinschaft Baden-Württemberg sind und einer Teilnahme nicht widersprochen haben

Wird noch erstellt

Berlin

**Rahmenvertrag**

**über die spezialisierte ambulante Palliativversorgung gemäß § 132d SGB V**

**in Berlin**

**zwischen**

**der Kassenärztlichen Vereinigung Berlin**
**- im Folgenden KV Berlin genannt -**

**und dem**
**Home Care Berlin e. V.**
**- im Folgenden HC e. V. genannt -**

**und dem**
**Berliner Aktionsbündnis ambulante Palliativpflege e.V.**
**- im Folgenden BAAP e.V. genannt -**

**sowie**

**der AOK Nordost - Die Gesundheitskasse**

**den Ersatzkassen:**

- **BARMER GEK**
- **Techniker Krankenkasse (TK)**
- **DAK - Gesundheit**
- **Kaufmännische Krankenkasse - KKH**
- **HEK - Hanseatische Krankenkasse**
- **Hkk**

**gemeinsamer Bevollmächtigter mit Abschlussbefugnis:**
**Verband der Ersatzkassen e. V. (vdek),**
**vertreten durch die Leiterin der vdek-Landesvertretung Berlin,**

**dem BKK Landesverband Mitte**
**Siebstraße 4**
**30171 Hannover,**

**der BIG direkt gesund,**

**der IKK Brandenburg und Berlin,**

**der Knappschaft, Regionaldirektion Berlin,**

**Sozialversicherung für Landwirtschaft, Forsten und Gartenbau (SVLFG)**
**als Landwirtschaftliche Krankenkasse, Hoppegarten**

**- im Folgenden Krankenkassen genannt -**

## Präambel

Die spezialisierte ambulante Palliativversorgung (SAPV) dient dem Ziel, die Lebensqualität und die Selbstbestimmung von Palliativpatienten so weit wie möglich zu erhalten, zu fördern und zu verbessern und diesen Patienten ein menschenwürdiges Leben bis zum Tod in ihrer gewohnten Umgebung, in stationären Pflegeeinrichtungen und stationären Hospizen zu ermöglichen. Die SAPV richtet sich an Palliativpatienten und deren soziales Umfeld, wenn die Intensität oder Komplexität der aus dem kurativ nicht mehr behandelbaren Krankheitsverlauf resultierenden Probleme den Einsatz von besonders spezialisierten Leistungserbringern erfordert. Die Partner dieser Vereinbarung sind sich einig, dass es sich bei diesen Leistungen nicht um solche handelt, die bereits durch den EBM erfasst und vergütet werden.

## § 1 Geltungsbereich

Diese Vereinbarung gilt im Bereich der KV Berlin für

- Vertragsärzte (im folgenden spezialisierte Palliativärzte genannt), die an der vertragsärztlichen Versorgung teilnehmen und die an dem Vertrag nach abgeschlossenem Teilnahmeverfahren gemäß § 8 teilnehmen.
- Versicherte der am Vertrag beteiligten Krankenkassen unabhängig vom Wohnort des Versicherten.
- Für die Betriebskrankenkassen wird diese Vereinbarung mit dem BKK Landesverband Mitte als Rahmenvereinbarung geschlossen; sie gilt insoweit nur für Betriebskrankenkassen die der Vereinbarung ausdrücklich beitreten. Eine Liste der beigetretenen Betriebskrankenkassen wird der KV Berlin und dem Home Care Berlin e. V. vom BKK Landesverband Mitte regelmäßig zur Verfügung gestellt. Der Beitritt ist mit Wirkung zum Quartalsbeginn möglich. Der Beitritt muss spätestens 6 Wochen vor Quartalsbeginn gegenüber dem BKK Landesverband Mitte und der KV Berlin erklärt werden. Für beigetretene oder fusionierte Betriebskrankenkassen gelten die Kündigungsvorschriften des §18. Kündigungen sind gegenüber dem BKK Landesverband Mitte und der KV Berlin auszusprechen.
- Zwischen den Partnern dieses Vertrages besteht Einvernehmen, dass die Krankenkassen des IKK-Systems sich an diesem Vertrag durch einseitige Erklärung gegenüber den im Rubrum genannten Vertragsparteien beteiligen können. Der Beitritt ist mit Wirkung zum Quartalsbeginn möglich. Der Beitritt muss spätestens 6 Wochen vor Quartalsbeginn gegenüber den Vertragspartnern erklärt werden. Für beigetretene oder fusionierte Innungskrankenkassen gelten die Kündigungsvorschriften des § 18. Kündigungen sind gegenüber den Vertragspartnern zu erklären. Die Beteiligung beschränkt sich auf die Leistungsinhalte des Vertrages und beinhaltet keine gesonderte Mitwirkung bei Entscheidungen zu § 8 Abs. 2 des Vertrages.
- spezialisierte Leistungserbringer Palliativpflege (im folgenden spezialisierte Palliativpflegedienste genannt), welche die Voraussetzungen für die pflegerischen SAPV Leistungserbringer nach Anlage 1b erfüllen und an dem Vertrag nach abgeschlossenem Teilnahmeverfahren gemäß § 8 teilnehmen.
- Die Vertragspartner sind sich darin einig, dass bei Fusionen von Krankenkassen, gemäß §§ 144 Abs. 4, 150 Abs. 2 Satz 1, 160 Abs. 1 Satz 3, 168 a Abs. 1 Satz 3 und 171 a Abs. 1 Satz 3 SGB V die neue Krankenkasse in die Rechte und Pflichten der bisherigen Krankenkassen eintritt.

- Die Vertragspartner sind sich darin einig, dass bei Insolvenz oder Schließung einer Krankenkasse der § 171d SGB V einschlägig ist.

## § 2 Gegenstand des Vertrages

(1) Gegenstand der Vereinbarung ist die spezialisierte ambulante Palliativversorgung gemäß der Richtlinie des Gemeinsamen Bundesausschusses zur Verordnung von spezialisierter ambulanter Palliativversorgung nach § 37b SGB V in der jeweils geltenden Fassung (SAPV-RL). Die Leistungen müssen ausreichend und zweckmäßig sein, dürfen das Maß des Notwendigen nicht überschreiten und sind wirtschaftlich zu erbringen. Im Falle einer Besserung bzw. einer Stabilisierung im Krankheitsverlauf ist der Versorgungsumfang der SAPV so weit wie möglich zu reduzieren.

(2) Die Leistungserbringung in stationären Hospizen kann ausschließlich als ergänzende ärztliche Teilleistung gemäß § 37b Absatz 1 Satz 4 SGB V im Rahmen der SAPV nach diesem Vertrag erfolgen.

## § 3 Grundsätze der Leistungserbringung

(1) Die spezialisierten Palliativärzte sowie die spezialisierten Palliativpflegedienste arbeiten nach einem verbindlichen, strukturierten und schriftlich dargelegten Konzept, in dem der inhaltliche und organisatorische Rahmen der Leistungserbringung (inkl. der personellen und sächlichen Ausstattung) sowie die Einbindung in die regionale Versorgungsstruktur beschrieben sind.

(2) Die Leistungen der SAPV nach diesem Vertrag werden den Versicherten als ärztliche und pflegerische Sachleistung zur Verfügung gestellt. Sie sind dem jeweiligen aktuellen Versorgungsbedarf entsprechend durch den spezialisierten Palliativpflegedienst sowie den spezialisierten Palliativarzt als
- Beratungsleistung
- Koordination der Versorgung
- additiv unterstützende Teilversorgung,
- vollständige Versorgung

zu erbringen. Die Leistungen müssen ausreichend und zweckmäßig sein, dürfen das Maß des Notwendigen nicht überschreiten und sind wirtschaftlich zu erbringen.

(3) Sofern Kinder und Jugendliche versorgt werden, sind deren Belange besonders zu berücksichtigen.

(4) Die Versorgung mit Arznei-, Verband-, Heil- und Hilfsmitteln ist sicherzustellen. Näheres zu den Verordnungen ist in § 12 geregelt.

## § 4 Anspruchsberechtigte Versicherte

Anspruchsberechtigt gemäß § 37b SGB V sind Versicherte:
- die an einer nicht heilbaren zum Tode führenden, fortschreitenden Erkrankung leiden, die soweit fortgeschritten ist, dass krankheitsspezifische Therapien mit lebensverlängernder Zielsetzung ausgeschöpft oder nicht mehr sinnvoll sind - ausgenommen in Einzelfällen krankheitsspezifische Therapieformen, die auf eine Symptomlinderung abzielen und bei denen die Verbesserung von Symptomatik und Lebensqualität im Vordergrund steht (z.B. Strahlentherapie von Knochenmetastasen zur Schmerzlinderung),

- und deren Lebenserwartung auf Tage, Wochen oder Monate gesunken ist.

Diese Versicherten haben einen Anspruch auf SAPV-Leistungen, wenn sie wegen eines komplexen Symptomgeschehens (§ 4 SAPV-RL) eine besonders aufwändige Versorgung benötigen, die nur durch spezialisierte Ärzte gewährleistet werden und die ambulant, im stationären Hospiz, in einer vollstationären Pflegeeinrichtung, in Wohneinrichtungen und Einrichtungen der Eingliederungshilfe für behinderte Menschen im Sinne des § 55 SGB XII und Einrichtungen der Kinder- und Jugendhilfe im Sinne § 34 SGB VIII (§ 1 Abs. 2 und 3 der SAPV-RL) erbracht werden kann. Eine zusätzlich zum Vorhandensein eines komplexen Symptomgeschehens vorliegende dekompensierte häusliche Situation mit Überforderung der Angehörigen kann Kombinationsleistungen und den Einsatz anderer palliativmedizinisch tätiger Berufsgruppen notwendig machen.

Zusätzlich zum Vorhandensein eines komplexen Symptomgeschehens können die Versicherten ihre häusliche Umgebung ohne erheblichen Aufwand nicht mehr verlassen.

## § 5 Leistungen der SAPV

(1) Die Leistungen der SAPV gemäß § 5 der Richtlinie des G-BA, die durch den spezialisierten Palliativarzt sowie durch den spezialisierten Palliativpflegedienst zu erbringen sind, umfassen:

1. Beratung (persönlich und/oder telefonisch)
   Erst-Beratung
   (a) des behandelnden Haus- bzw. Facharztes,
   (b) des Versicherten und/oder dessen Angehörigen,
   (c) der ausführenden (SAPV-)Pflegefachkraft.
2. Koordination:
   - Koordination der Versorgung durch regelmäßige Abstimmung mit allen beteiligten Leistungserbringern,
   - Erstellung / Führung eines individuellen Behandlungs- bzw. Pflegeplans
3. additiv unterstützende Teilversorgung:
   - konsiliarische Beratung des behandelnden Haus- bzw. Facharztes,
   - Koordination, Teilnahme an Fallbesprechungen
   - Hausbesuch(e) bedarfsgerecht
4. Vollversorgung
   - kontinuierliche konsiliarische Anleitung, Koordination und Monitoring der gesamten Palliativversorgung, auch im Rahmen von Visiten und Fallbesprechungen.
   - Hausbesuch(e) bedarfsgerecht.

(2) Leistungen im Rahmen des SGB XI sind nicht Inhalt der SAPV. Hierfür gelten die jeweiligen gesetzlichen und vertraglichen Regelungen.

## § 6 Verordnung und Genehmigung der SAPV

(1) Die Leistungen der SAPV sind entsprechend der Richtlinie zu verordnen, der zuständigen Krankenkasse einzureichen und bedürfen entsprechend der Richtlinie der Genehmigung durch die zur Leistung verpflichtete Krankenkasse. Art, Inhalt, Umfang und Dauer ergeben sich aus der Verordnung. Die Verordnung hat auf dem aktuell gültigen Muster 63 des Vordruckes für die Verordnung/ Genehmigung der SAPV gemäß Vordruck - Vereinbarung (Anlage 2/2a der Bundesmantelverträge) zu erfolgen. Eine Verordnung für einen

zurückliegenden Zeitraum vor Datum der Verordnung ist unzulässig.

(2) Die Krankenkasse übernimmt gemäß § 8 der SAPV-RL bis zu einer Entscheidung über die Leistungserbringung die Kosten für die verordneten und erbrachten SAPV - Leistungen entsprechend der vereinbarten Vergütung nach diesem Vertrag, wenn die Verordnung spätestens an dem dritten der Ausstellung folgenden Arbeitstag der Krankenkasse vorgelegt wird. Im Falle der Übermittlung per Fax, ist das Original nach Satz 1 unverzüglich nachzureichen. Die Kostenübernahme ist ab Beginn abzulehnen, wenn die Genehmigungsvoraussetzungen zum Zeitpunkt der Verordnung offensichtlich nicht vorlagen und ein SAPV - Leistungsanspruch gegenüber der Krankenkasse erkennbar nicht bestand.

(3) Für die Folgeverordnung gelten die Absätze 1 bis 2 entsprechend.

(4) Die Vertragspartner vereinbaren, dass SAPV-Folgeverordnungen mittels Muster 63 auch von spezialisierten Palliativärzten im Angestelltenverhältnis ausgestellt werden können und die Abrechnung der EBM-GOP 01426 durch diese zulässig ist.

## § 7 Teilnahmevoraussetzungen

(1) Voraussetzungen zur Teilnahme an diesem Vertrag als spezialisierter Palliativarzt sind:

a. Zulassung als Vertragsarzt im KV-Bereich Berlin oder angestellter Arzt in einer zugelassenen Praxis (Vertragsärzte, Ärzte in Berufsausübungsgemeinschaften, in MVZ)

   und

b. abgeschlossene Weiterbildung Palliativmedizin gemäß der Weiterbildungsordnung der Landesärztekammern

   und

c. selbstständige ambulante Versorgung von mindestens 75 Palliativpatienten innerhalb der letzten 3 Jahre oder mindestens eine einjährige klinische palliativmedizinische Tätigkeit in einer Palliativabteilung eines Krankenhauses innerhalb der letzten 3 Jahre

   und

d. Nachweis einer Kooperationsvereinbarung nach Anlage 1a mit mindestens einem von den Krankenkassen zugelassenen spezialisierten Palliativpflegedienst.

e. Sofern die geforderte Anzahl von mindestens 75 Palliativpatienten gemäß lit. c. nicht vorliegt, ist eine Teilnahme an diesem Vertrag dennoch möglich. Vorausgesetzt, die Fallzahl gemäß der Empfehlung des GKV Spitzenverbandes in der Fassung vom 05.11.2012 wird in einem Zeitraum von 12 Monaten erworben und nachgewiesen.

f. Angestellte Ärzte die über eine Abrechnungsgenehmigung vor dem 30.06.2010 (Rahmenvertrag nach § 132d SGB V) der KV Berlin verfügen und die Voraussetzungen nach lit. a und lit. b nicht erfüllen, dürfen weiterhin teilnehmen, solange und soweit sie in einem Anstellungsverhältnis bleiben.

g. Abweichend von den Voraussetzungen (a-f) können sich die Vertragspartner bei begründetem Bedarf zur Sicherstellung der Versorgung in Form von Einzelfallentscheidungen verständigen.

(2) Voraussetzungen zur Teilnahme an diesem Vertrag als spezialisierter Palliativpflegedienst und als spezialisierter Leistungserbringer Palliativpflege in Trägerschaft vollstationärer Pflegeheime sind:
  a. mindestens 12 Monate Vertragspartner der Krankenkassen in Berlin gemäß § 132 a Abs. 2 SGB V (häusliche Krankenpflege),
  b. Vorhalten eines SAPV-Fachpflegeteams gemäß den personellen, organisatorischen und qualitativen Anforderungen gemäß Anlage 1b,
  c. Nachweis einer Kooperationsvereinbarung nach Anlage 1a mit mindestens einem von den Krankenkassen zugelassenen spezialisierten Palliativarzt.
  d. Für spezialisierte Leistungserbringer Palliativpflege in Trägerschaft vollstationärer Pflegeheime mit einem Versorgungsvertrag gemäß § 72 SGB XI mit den Pflegekassen in Berlin kann der Nachweis eines Vertrages gemäß § 132a Abs.2 SGB V (häusliche Krankenpflege) entfallen, soweit und solange dies zur Bedarfsdeckung erforderlich ist. Voraussetzung dafür ist, dass der Leistungserbringer einen regionalen Versorgungsauftrag über die Heimbewohner hinaus im Versorgungsgebiet seiner Betriebsstätte übernimmt und die wirtschaftliche Selbstständigkeit des SAPV - Leistungserbringerteams nachgewiesen ist.

(3) Soweit weitere Fachkräfte (z. B. Sozialarbeiter; Sozialpädagogen, Psychologen) eingebunden werden, sollen diese über eine Zusatzweiterbildung Palliative Care für andere Berufsgruppen oder über eine mehrjährige Erfahrung in der Palliativversorgung verfügen.

(4) Die Qualifikationen gemäß Absätzen 1 und 2 sind mit der Teilnahmeerklärung zum Vertrag (Anlagen 4 und 5) gegenüber der KV Berlin bzw. gegenüber den Krankenkassen nachzuweisen.

(5) der spezialisierte Palliativarzt hat als Mindestanforderung an die sächliche Ausstattung Folgendes vorzuhalten bzw. sicherzustellen:
- eine geeignete, aktuell geführte und für die an der Versorgung Beteiligten jederzeit zugängliche Patientendokumentation
- Arztkoffer / Bereitschaftstasche (Berücksichtigung der Kompatibilität der Verbrauchsmaterialien zu Medizinprodukten unterschiedlicher Hersteller, z.B. bei Portsystemen oder Infusionspumpen)
- eine geeignete administrative Infrastruktur, z.B. Büro, Kommunikationstechnik
- Arzneimittel (inklusive Betäubungsmittel) für die Notfall-/Krisenintervention.

(6) Der spezialisierte Palliativarzt muss über eine eigenständige Adresse und geeignete Räumlichkeiten für
- die Beratung von Versicherten und Angehörigen
- Teamsitzungen und Besprechungen
- die Lagerhaltung von eigenen Medikamenten für Notfall-/ Krisenintervention und Hilfsmittel verfügen.
- Die Einhaltung der Anforderungen an den Umgang mit Betäubungsmitteln im Sinne des BTMG sind durch den Palliativarzt sicherzustellen.

(7) Die bestehenden gesetzlichen und berufsrechtlichen Dokumentationspflichten werden durch diesen Vertrag nicht berührt.

(8) Die weiteren Dokumentationserfordernisse nach diesem Vertrag und eine entsprechende Berichterstattung gemäß § 11 sind zu beachten.

## § 8 Teilnahmeverfahren

(1)a. Der spezialisierte Palliativarzt erklärt seine Teilnahme an diesem Vertrag mittels der Teilnahmeerklärung nach Anlage 4. Im Auftrag der Krankenkassen prüft die KV Berlin die Teilnahmevoraussetzungen nach § 7. Die Genehmigung zur Teilnahme wird von der KV Berlin im Auftrag der Krankenkassen erteilt, wenn alle Voraussetzungen erfüllt sind. Die KV Berlin übermittelt nach Abschluss des Teilnahmeverfahrens den Krankenkassen die unterzeichnete Teilnahmeerklärung nach Anlage 4 in Kopie.

b. Der spezialisierte Palliativpflegedienst erklärt seine Teilnahme an diesem Vertrag mittels Teilnahmeerklärung nach Anlage 5. Die Krankenkasse prüft die Teilnahmevoraussetzungen nach § 7. Die Genehmigung zur Teilnahme wird von den Krankenkassen erteilt, wenn alle Voraussetzungen erfüllt sind.

(2) Eine Teilnahme an diesem Vertrag ist nur zulässig, soweit diese zur Bedarfsdeckung erforderlich ist. Die Krankenkassen teilen der KV Berlin schriftlich mit, ob der Bedarf gedeckt ist und keine weiteren Genehmigungen nach Abs.1 in ihrem Auftrag zu erteilen sind. Die Teilnahme an diesem Vertrag endet mit Wegfall der Teilnahmevoraussetzungen nach § 7 sowie mit der Mitteilung der KV Berlin im Auftrag der Krankenkassen über das Ende der Teilnahme.

(3) Der spezialisierte Palliativarzt kann seine Teilnahme an diesem Vertrag gegenüber der KV Berlin mit einer Frist von 4 Wochen zum Quartalsende schriftlich kündigen.

(4) Der spezialisierte Palliativpflegedienst kann seine Teilnahme an diesem Vertrag gegenüber der Krankenkasse mit einer Frist von 4 Wochen zum Quartalsende schriftlich kündigen.

(5) Zur Verwaltungsvereinfachung vereinbaren die Vertragspartner, dass die nachfolgend aufgeführten Fallkonstellationen grundsätzlich genehmigungsfähig sind und nicht unter eine nach § 8 Abs. 2 Satz 1 der Vereinbarung ausgesprochene Bedarfsdeckung fallen:

- Genehmigung der Teilnahme eines angestellten Arztes, der über die persönliche Genehmigung verfügt, bei Arbeitgeber-Wechsel, vorausgesetzt, die neu anstellende Vertragsarztpraxis verfügt über eine Genehmigung zur Anstellung eines Sicherstellungsassistenten für die Abrechnung der SAPV - Leistungen.
- Genehmigung der Teilnahme des Praxisnachfolgers bei vorheriger Beendigung der vertragsärztlichen Tätigkeit des ehemaligen Praxisinhabers, welcher selbst über eine Abrechnungsgenehmigung verfügte. Voraussetzung ist, dass der Praxisnachfolger die Teilnahmevoraussetzungen erfüllt.
- Genehmigung der Teilnahme bei vertragsärztlicher Zulassung von bisher angestellten SAPV - Assistenten.

Die KV Berlin informiert die Vertragspartner entsprechend den Regelungen in § 8 Abs. 1 Satz 3 über veränderte Genehmigungen.

## § 9 Wegfall der Teilnahmevoraussetzungen

(1) Die Teilnahme des spezialisierten Palliativarztes endet, ohne dass es einer separaten schriftlichen Kündigung bedarf, mit

a. dem Ableben des spezialisierten Palliativarztes,

b. der Beendigung der Zulassung bzw. des Anstellungs- oder Beschäftigungsverhältnisses,

c. der Einstellung des Praxisbetriebes (z. B. Auf-/Übergabe oder Verkauf der Praxis).

(2) Die Teilnahme des spezialisierten Palliativpflegedienstes endet, ohne dass es einer separaten schriftlichen Kündigung bedarf, mit

a. der Beendigung des Vertrages gemäß § 132a Abs. 2 SGB V (häusliche Krankenpflege),

b. der Einstellung des Betriebes.

## § 10 Aufgaben der Leistungserbringer

(1) Der spezialisierte Palliativarzt und/oder der spezialisierte Palliativpflegedienst koordiniert/koordinieren zur ziel- und qualitätsorientierten Erfüllung des Vertrages die Einbindung der an der Versorgung beteiligten Leistungserbringer des Versorgungsnetzes.

(2) Der spezialisierte Palliativarzt und der spezialisierte Palliativpflegedienst kooperieren zur Sicherstellung der psychosozialen Unterstützung mit ambulanten Hospizdiensten gemäß § 39 a Abs. 2 SGB V.

(3) Der spezialisierte Palliativarzt sowie der spezialisierte Palliativpflegedienst gewährleisten jeweils eine 24-Stunden-Bereitschaft. Der behandelnde spezialisierte Palliativarzt stellt bei z.B. urlaubs- oder krankheitsbedingter Abwesenheit die ständige Verfügbarkeit eines spezialisierten Palliativarztes sicher. Für Vertragsärzte sowie für an diesem Vertrag teilnehmende Ärzte, die keine Vertragsärzte sind, gelten die gleichen Vertretungsregelungen gemäß § 32 Abs. 1, 2 und 4 Ärzte-Zulassungsverordnung. Ebenso gelten die Melde- und Genehmigungspflichten gegenüber der Kassenärztlichen Vereinigung Berlin.

(4) Der spezialisierte Palliativarzt:

- führt - soweit notwendig - eine Erst-Beratung
  (a) des behandelnden Haus- bzw. Facharztes,
  (b) des Versicherten und/oder der Angehörigen,
  (c) der ausführenden (SAPV-)Pflegefachkraft durch,
- erstellt im Rahmen der Koordination den individuellen Behandlungsplan;
- stimmt sich im Rahmen der Koordination mit den beteiligten Leistungserbringern ab,
- berät im Rahmen der **additiv unterstützenden Teilversorgung** bei Bedarf konsiliarisch den behandelnden Haus- bzw. Facharzt, nimmt je nach Bedarf an Fallbesprechungen teil und führt Hausbesuche bedarfsgerecht durch;
- koordiniert im Rahmen der **Vollversorgung** die Übernahme der spezialisierten ambulanten Palliativversorgung, ggf. in Absprache mit dem verordnenden Arzt und bezieht die nach individuellem Bedarf des Palliativpatienten erforderlichen Kooperationspartner in die Versorgung ein und führt Hausbesuche bedarfsgerecht durch;
- dokumentiert die Leistungen gemäß § 11 und übersendet zum Zweck der Erstellung des Qualitätsberichts sämtliche Dokumentationsbögen gemäß Anlage 3a bis spätestens zum 15.01. des Folgejahres an den HC e. V.

(5) Der spezialisierte Palliativpflegedienst führt im Rahmen ärztlich angeordneter Palliativpflege eine

- Erst-Beratung der (SAPV) - Pflegefachkräfte des Pflegedienstes/Pflegeheims sowie des Versicherten und/oder dessen Angehörigen durch,
- stimmt sich im Rahmen der Koordination mit den beteiligten Leistungserbringern ab,

- berät bei Bedarf die Pflegefachkräfte des Pflegedienstes bzw. des Pflegeheims sowie die Patienten und deren Angehörige zu den Maßnahmen der Diagnostik und Symptomlinderung,
- übernimmt in Absprache mit dem verordnenden Arzt die Durchführung spezialisierter palliativpflegerischer Leistungen, die nach ihrer Art, Schwere oder Komplexität die Kompetenz einer Pflegefachkraft mit einer curricularen Weiterbildung zu Palliative Care erfordern; bezieht die nach individuellem Bedarf des Palliativpatienten erforderlichen Kooperationspartner in die Versorgung ein und führt Hausbesuche bedarfsgerecht durch,
- wirkt mit an Maßnahmen zur Symptomlinderung, insbesondere in Form von:
  - Diagnostik symptomverstärkender Probleme sowie individueller Ressourcen auch in Bezug auf AeDLs,
  - Einsicht vermitteln in symptomverstärkende Faktoren und Problemlagen sowie motivieren für eine Problemlösung,
  - Stärken der Selbstverantwortlichkeit, Sicherheit vermitteln,
  - Bewältigungsstrategien in Bezug auf die Symptomlast entwickeln,
  - konkrete Lösungsschritte vereinbaren und überprüfen,
  - weitere professionelle palliativtherapeutische Hilfe anbieten und organisieren,
  - Kriseninterventionen,
  - ärztlich verordneten Behandlungspflegen,
- dokumentiert die Leistungen gemäß § 11 und übersendet zum Zweck der Erstellung des Qualitätsberichts sämtliche Dokumentationsbögen gemäß Anlage 3b bis spätestens zum 31.3. des Folgejahres an den HC e.V..

(6) Der HC e.V.
- erstellt einen jährlichen Qualitätsbericht gemäß § 11 Absatz 5,
- organisiert Fortbildungen, Qualitätszirkel und multidisziplinäre / multiprofessionelle Fallbesprechungen in Eigenregie und unterstützt die sich in allen Teilen Berlins gebildeten Palliativnetze bei der Organisation eigener Fortbildungen, Qualitätszirkel und Fallbesprechungen, die nach § 11 vorgeschrieben sind,
- vertritt die Interessen aller SAPV - Leistungserbringer.

(7) Der BAAP e.V.
- vertritt mit HC e. V. gemeinsam die Interessen der am SAPV - Rahmenvertrag teilnehmenden spezialisierten palliativpflegerischen Leistungserbringer für die pflegerischen Bestandteile des SAPV - Rahmenvertrages, wie die fachlichen Anforderungen sowie die Vergütung der spezialisierten palliativpflegerischen Leistungserbringer. Dabei sollen die Interessen der spezialisierten palliativpflegerischen Leistungserbringer durch HC e. V. und BAAP e.V. einheitlich vertreten werden.

## § 11 Qualitätssicherung

(1) Die Leistungserbringer erstellen einen jeweils individuellen Behandlungsplan bzw. Pflegeplan, der mit den übrigen an der Versorgung beteiligten Leistungserbringern abzustimmen ist. Dies gilt nicht, wenn der spezialisierte Palliativarzt ausschließlich nur berät.

(2) Die zur Leistungserbringung und Abrechnung von Leistungen nach diesem Vertrag berechtigten Leistungserbringer sind verpflichtet, ein internes Qualitätsmanagement durchzuführen. Sie nehmen regelmäßig an multiprofessionellen Fortbildungen zur Palliativmedizin/-pflege teil und führen möglichst halbjährlich multidisziplinäre Fallbesprechungen

durch, an denen die übrigen an der Versorgung Tätigen teilnehmen. Sie sollen sich außerdem an Maßnahmen der externen Qualitätssicherung beteiligen.

(3) Die Leistungserbringung ist sachgerecht und kontinuierlich zu dokumentieren.

(4) Der BAAP e.V. unterstützt HC e. V. im Rahmen seiner vertraglichen Aufgaben bei Maßnahmen der Qualitätssicherung und deren Weiterentwicklung sowie bei der optimalen Vernetzung aller im Rahmen der SAPV tätigen Leistungserbringer.

(5) Der Qualitätsbericht über die SAPV nach diesem Vertrag ist einmal jährlich, auf Grundlage der Anlagen 3a und 3b, bis zum 30.06. des Folgejahres durch HC e.V. zu erstellen und an die Vertragspartner zu übermitteln. Der Qualitätsbericht hat mindestens Aufschluss zu geben über
- Anzahl der mit Leistungen nach diesem Vertrag versorgten Versicherten unter Benennung des Anteils verstorbener Versicherter,
- Anzahl der spezialisierten Palliativärzte sowie der kooperierenden Leistungserbringer,
- Krankenhauseinweisungen,
- durchschnittliche Betreuungsdauer,
- Maßnahmen des internen Qualitätsmanagements,
- Teilnahme an Maßnahmen der externen Qualitätssicherung,
- Durchführung multidisziplinärer Fallbesprechungen.

(6) Die Teilnahme an Supervision ist zu ermöglichen.

(7) Die Notwendigkeit und Wirtschaftlichkeit der Verordnung und der Leistungen nach diesem Vertrag können von den Krankenkassen durch den Medizinischen Dienst der Krankenversicherung einzelfallbezogen und im Rahmen von Stichprobenprüfungen im Umfang von 10 Prozent der verordneten Fälle eines Quartals überprüft werden. Die zur Überprüfung durch den Medizinischen Dienst der Krankenversicherung erforderlichen Unterlagen sind auf Anforderung des MDK unter Berücksichtigung des Datenschutzes von den Leistungserbringern zur Verfügung zu stellen. Bei im Ergebnis der Prüfung durch den MDK festgestellten Verstößen gegen die Notwendigkeit und Wirtschaftlichkeit der Verordnung und der Leistungen nach diesem Vertrag wird der Leistungserbringer von der weiteren Teilnahme am Vertrag ausgeschlossen.

## § 12 Verordnung von Arzneimitteln, Verband-, Heil- und Hilfsmitteln

(1) Die Verordnungen sind vollständig und ordnungsgemäß (u. a. auf den Namen des/der Versicherten) auszustellen. Dabei hat der spezialisierte Palliativarzt die von der KBV zu vergebende spezifische Betriebsstättennummer und die einheitliche Pseudo-Arztnummer 333 333 300 anzugeben. Diese Ziffern sind auch auf Betäubungsmittel-Rezepten anzugeben. Solange das BtM-Muster noch nicht angepasst ist, ist die SAPV-spezifische BSNR in das Feld „Vertragsarztnummer“ und die Pseudo-Arztnummer in das Feld „VK gültig bis“ einzutragen.

(2) Für die Verordnung von Arznei-, Verband-, Heil- und Hilfsmitteln im Rahmen der SAPV sind ausschließlich die für die vertragsärztliche Versorgung jeweils aktuell gültigen Verordnungsmuster gemäß den Anlagen des Bundesmantelvertrages Ärzte (Muster 13, 14, 16 oder 18) zu verwenden. Bei der zeitgleichen Verordnung von Arzneimitteln und Hilfsmitteln sind jeweils gesonderte Muster 16 zu verwenden.

(3) Die Verordnungen von Arznei-, Verband-, Heil- und Hilfsmitteln im Rahmen der SAPV haben insbesondere den gesetzlichen Vorschriften der §§ 31-36 SGB V, den jeweils geltenden Richtlinien des Gemeinsamen Bundesausschusses nach § 92 SGB V sowie der

einschlägigen Rechtsprechung des Bundessozialgesetzes (z.B. zum Off-Label-Use und zu Import-Arzneimitteln) zu entsprechen. Ferner ist das Wirtschaftlichkeitsgebot des § 12 SGB V zu beachten. Den Krankenkassen bleiben entsprechende Prüfungen vorbehalten.

(4) Für die im Rahmen der SAPV verordneten Arznei-, Verband-, Heil- und Hilfsmittel gelten die nach §§ 300, 302 SGB V vereinbarten Abrechnungsverfahren entsprechend.

(5) Die Krankenkassen verpflichten sich, verordnungs- und bewilligungsfähige Hilfsmittel für SAPV - Patienten zügig zu bearbeiten.

## § 13 Abrechnung und Vergütung

(1) Die Leistungsbeschreibung und Höhe der Vergütung ergibt sich aus Anlage 2.

(2) a) Der spezialisierte Palliativarzt rechnet die Leistungen gegenüber der KV Berlin ab. Es gilt die jeweils gültige Abrechnungsordnung der KV Berlin. Die KV Berlin ist berechtigt, die Verwaltungskosten in der jeweils gültigen Höhe einzubehalten.
b) Der spezialisierte Palliativpflegedienst rechnet die Leistungen gegenüber der Krankenkasse ab.

(3) Die Krankenkassen entrichten an die KV Berlin die Vergütungen außerhalb der morbiditätsbedingten Gesamtvergütung und außerhalb jeder mengenbegrenzenden Regel auf Anforderung. Für die Zahlungen (Abschlags- sowie Restzahlungen), insbesondere Fristen und Zinsregelungen gelten die Bestimmungen des aktuell gültigen Honorarvertrages zwischen den Gesamtvertragspartnern entsprechend. Die Vergütung an den Arzt erfolgt erst nach und nur in Höhe der von den Krankenkassen geleisteten Zahlungen. Abschlagszahlungen sind vom Arzt bei der KV Berlin anzufordern, vorgenannter Satz gilt entsprechend.

(4) Honorarrückforderungen und evtl. Regresse können nicht verrechnet werden.

(5) Die Vertragspartner können sich für den Fall, dass die Abrechnung von Leistungen weiterer Leistungserbringer (z.B. Pflegeleistungen) über die KV Berlin erfolgen soll, über die Bedingungen und Regeln (bilateral) verständigen.

## § 14 Haftungsfreistellung

(1) Das Haftungsrisiko aus den im Auftrag der Krankenkassen durch die KV Berlin durchgeführten Aufgaben tragen die Krankenkassen.

(2) Kosten für Verwaltung, Aufwendungen der KV Berlin, insbesondere Steuern, Gebühren und Kosten der Rechtsverfolgung werden von den beteiligten Krankenkassen der KV Berlin auf Anforderung unverzüglich erstattet, soweit sie nicht durch die einbehaltenen Verwaltungskosten gedeckt sind. Die Höhe der Verzugszinsen beträgt 3,8 Prozent. Die Krankenkassen haften gesamtschuldnerisch.

## § 15 Anzeige- und Mitteilungspflichten

Der spezialisierte Palliativarzt sowie der spezialisierte Palliativpflegedienst informiert jeweils die vertragsschließenden Krankenkassen über vertragsrelevante Änderungen.

## § 16 Vertragsverstöße

Verstößt ein Vertragspartner bzw. ein Leistungserbringer nach diesem Vertrag gegen eine Pflicht aus diesem Vertrag, ist er zum Ersatz des daraus entstandenen Schadens verpflichtet.

## § 17 Datenschutz

(1) Der zur Leistungserbringung und Abrechnung von Leistungen nach diesem Vertrag berechtigte Leistungserbringer verpflichtet sich, die gesetzlichen Bestimmungen über den Schutz der Sozialdaten zu beachten und die Daten nur zweckgebunden zu verwenden. Entsprechende Verpflichtungen sind für alle Kooperationspartner in der Kooperationsvereinbarung, Anlage 1 geregelt.

(2) Der zur Leistungserbringung und Abrechnung von Leistungen nach diesem Vertrag berechtigte Leistungserbringer ist darüber hinaus verpflichtet, bei der Verarbeitung personenbezogener Daten ausschließlich Personen einzusetzen, die auf das Datengeheimnis nach § 5 Bundesdatenschutzgesetz (BDSG) und § 8 Landesdatenschutzgesetz Berlin (BLnDGS) sowie § 78 SGB X verpflichtet sind. Ferner ist sicherzustellen, dass das von den zur Leistungserbringung und Abrechnung von Leistungen nach diesem Vertrag, durch den berechtigten Leistungserbringer sowie das von seinen Kooperationspartnern, eingesetzte Personal im Sinne der Datenschutzvorschriften ausreichend informiert und angewiesen sind. Entsprechende Verpflichtungserklärungen sind bei berechtigtem Interesse den Vertragspartnern auf Verlangen vorzulegen.

## § 18 Inkrafttreten und Kündigung

(1) Der Vertrag vom 06.07.2010 wird zum 01.10.2013 mit den vorgenommenen Änderungen in der vorliegenden geänderten Fassung fortgeführt

(2) Dieser Vertrag kann von jedem Vertragspartner mit einer Frist von drei Monaten zum Ende eines Quartals, frühestens zum 30.09.2015 gekündigt werden. Die Kündigung hat mit eingeschriebenem Brief oder per Boten zu erfolgen. Die Kündigung eines Vertragspartners berührt die Weitergeltung des Vertrages zwischen den übrigen Vertragspartnern nicht.

(3) Das Recht zur außerordentlichen Kündigung aus wichtigem Grund bleibt unberührt. Die Vertragspartner vereinbaren, unverzüglich in Verhandlungen über eine Anpassung des Vertrages einzutreten, wenn Rechtsänderungen auf die Inhalte dieses Vertrages wirken.

## § 19 Salvatorische Klausel

Sollte eine Bestimmung dieser Vereinbarung unwirksam sein oder werden oder die Vereinbarung unvollständig sein, so werden die übrigen Inhalte der Vereinbarung hiervon nicht berührt, es sei denn, die unwirksame Bestimmung ist für eine Vertragspartei derart wesentlich, dass ihr ein Festhalten am Vertrag nicht zugemutet werden kann. Die unwirksame oder fehlende Bestimmung wird durch eine solche Bestimmung ersetzt, die dem Sinn und Zweck unwirksamer Bestimmungen in rechtswirksamer Weise und wirtschaftlich am nächsten kommt. Gleiches gilt für etwaige Vertragslücken.

## § 20 Sonstige Bestimmungen

(1) Die Vertragspartner verpflichten sich zu regelmäßigen Konsultationen, um sich über die Versorgungsabläufe nach diesem Vertrag sowie die in diesem Zusammenhang auftretenden Probleme und Schwierigkeiten zeitnah auszutauschen und gemeinsam nach bedarfsgerechten Lösungen zu suchen.

(2) Die Vertragspartner werden gemeinsam prüfen, inwieweit die Ziele der Präambel erreicht worden sind.

(3) Die Vertragspartner werden die Versorgungsabläufe nach diesem Vertrag konstruktiv beobachten und sind bereit, erforderliche Anpassungen aufgrund der Praxiserfahrungen bei neuen Vertragsverhandlungen vorzunehmen.

**Anlagen**

| | |
|---|---|
| Anlage 1 | Kooperation |
| Anlage 1a | Kooperationsvereinbarung - Muster |
| Anlage 1b | Anforderungen an die spezialisierten Palliativpflegedienste |
| Anlage 2a | Vergütung spezialisierte Palliativärzte |
| Anlage 2b | Vergütung spezialisierte Palliativpflegedienste |
| Anlage 3a | Patientendokumentation „Arzt“ in der Berliner SAPV |
| Anlage 3b | Patientendokumentation „Pflege“ in der Berliner SAPV |
| Anlage 4 | Teilnahmeerklärung des spezialisierten Palliativarztes |
| Anlage 5 | Teilnahmeerklärung des spezialisierten Palliativpflegedienstes |

Berlin, Potsdam, Hoppegarten den 16. SEP. 2013

| | |
|---|---|
| Kassenärztliche Vereinigung Berlin<br>Für den Vorstand | AOK Nordost - Die Gesundheitskasse |
| Home Care Berlin e.V.<br>**Der Vorstand** | Verband der Ersatzkassen e.V. (vdek)<br>Die **Leiterin** der vdek-Landesvertretung Berlin/Brandenburg |
| Berliner Aktionsbündnis<br>**ambulante Palliativpflege e.V.**<br>**Der Vorstand** | BKK Landesverband Mitte<br>**Landesvertretung Berlin-Brandenburg** |
| | BIG direkt gesund |
| | IKK Brandenburg und Berlin |
| | SVLFG als Landwirtschaftliche Krankenkasse |
| | Knappschaft Regionaldirektion Berlin |

## Anlagen

## zum Rahmenvertrag über die spezialisierte ambulante Palliativversorgung (SAPV) gemäß § 132 d SGB V,

**Anlage 1 – Kooperation**

**Anlage 1 a – Kooperationsvereinbarung - Muster**

**Anlage 1 b – Anforderungen an die spezialisierten Palliativpflegedienste**

## Anlage 1: Kooperation

### § 1 Allgemeine Grundsätze der Kooperation

(1) Zur Sicherstellung der Versorgung mit Leistungen der SAPV wirken der spezialisierte Palliativarzt und die spezialisierten Palliativpflegedienste eng zusammen. Für die Erbringung von Leistungen der SAPV ist eine enge interdisziplinäre und professionsübergreifende Zusammenarbeit unerlässlich. Dazu kooperiert der Vertragspartner mit geeigneten Pflegeeinrichtungen nach Anlage 1 b und § 7 des Rahmenvertrages. Die Leistungserbringer sind Teil eines multiprofessionellen Versorgungsnetzes.

(2) Der spezialisierte Palliativarzt und der spezialisierte Palliativpflegedienst haben sich im Rahmen ärztlich verordneter spezialisierter Palliativpflege davon zu überzeugen, dass die jeweiligen Qualifikationen und Fachkompetenzen vorhanden sind.

(3) Die Voraussetzungen zur Leistungserbringung gemäß Anlage 1 b des Rahmenvertrages werden durch die Arbeitsgemeinschaft der Krankenkassenverbände in Berlin geprüft.

(4) Über Änderungen der organisatorischen und personellen Voraussetzungen informiert der spezialisierte Palliativpflegedienst die Krankenkassen direkt.

### § 2 Pflichten der Kooperationspartner

(1) Die spezialisierten Leistungserbringer informieren sich regelmäßig über den Bedarf des Patienten, über Veränderungen des Gesundheitszustandes und in der häuslichen Pflegesituation.

(2) Der spezialisierte Palliativarzt verpflichtet sich

a) die Pflegesituation im Rahmen der ärztlichen Therapie angemessen zu berücksichtigen und sich zu den pflegerischen und medizinischen Belangen mit der verantwortlichen Pflegefachkraft des spezialisierten Palliativpflegedienstes abzustimmen.

b) dem spezialisierten Palliativpflegedienst zur Optimierung des Informationsflusses grundsätzlich die Teilnahme an ärztlichen Hausbesuchen zu ermöglichen und ärztliche Anweisungen in der beim Patienten befindlichen Pflegedokumentation zu vermerken.

(3) Die gegenseitige Erreichbarkeit ist jederzeit sicherzustellen.

(4) Die telefonische Betreuung und die Durchführung von dringend erforderlichen Hausbesuchen rund um die Uhr sind für die betreuten Versicherten zu gewährleisten.

### § 3 Kooperationsvertrag

(1) Der Vertrag zwischen den Kooperationspartnern ist mit den Mindestinhalten des Musters der Anlage 1a des Rahmenvertrages für die SAPV schriftlich zu schließen.

(2) Der Kooperationsvertrag nach Absatz 1 kann auch als mehrseitiger Vertrag zwischen mehreren spezialisierten Palliativärzten und einem spezialisierten Palliativpflegedienst abgeschlossen werden.

(3) Bei Kündigung des Rahmenvertrages oder Beendigung des durch die Teilnahmeerklärung begründeten Vertragsverhältnisses endet die Kooperationsvereinbarung im Rahmen der SAPV, ohne dass es einer gesonderten Kündigung bedarf.

## Anlage 1 a) Kooperationsvereinbarung (Muster)

**Vereinbarung zur Erbringung von Leistungen der SAPV**

**zwischen**

**den Leistungserbringern gemäß § 1 des Rahmenvertrages SAPV**

____________________________________

____________________________________

____________________________________

(Name , Adresse der Betriebsstätte)

____________________________________

LANR, BSNR

**im folgenden spezialisierter Palliativarzt**

**und**

____________________________________

____________________________________

____________________________________

(Träger, Einsatzstelle, Adresse)

____________________________________

Institutionskennzeichen

**im folgenden spezialisierter Palliativpflegedienst**

### § 1 Gegenstand des Vertrages

Diese Kooperationsvereinbarung regelt die fachübergreifende Zusammenarbeit der spezialisierten Palliativärzte und dem spezialisierten Palliativpflegedienst zur Sicherstellung der Versorgung der Versicherten der Krankenkassen in Berlin mit Leistungen der SAPV.

### § 2 Rechte und Pflichten

Die Vertragspartner wirken gemeinsam auf eine hohe, dem medizinisch und pflegefachlich anerkannten Wissensstand entsprechende, Qualität der Versorgung hin.

Die Vertragspartner erfüllen die Anforderungen gemäß § 7 des Rahmenvertrages. Der spezialisierte Palliativpflegedienst beantragt unter Vorlage dieser Kooperationsvereinbarung und der Nachweise gemäß Anlage 1 b) des Rahmenvertrages die Anerkennung als spezialisierter Palliativpflegedienst bei der Arbeitsgemeinschaft der Krankenkassenverbände in Berlin (bis auf weiteres federführend AOK Nordost).

Der spezialisierte Palliativpflegedienst ist verpflichtet, die Anerkennung der Krankenkassen gemäß Anlage 1 b dem spezialisierten Palliativarzt nach Erhalt vorzulegen. Die Delegation von SAPV - Leistungen ist erst nach Bestätigung der fachlichen Eignung durch die Krankenkassen zulässig.

Wird die Anerkennung durch die Krankenkassen entzogen, hat der spezialisierte Palliativpflegedienst seinen Kooperationspartner unverzüglich zu informieren.

Der spezialisierte Palliativpflegedienst informiert den spezialisierten Palliativarzt über gravierende Änderungen in der Besetzung der fachlichen Leitung und bei Unterschreitung der Personalmindestvorhaltung.

## § 3 Abrechnung

Der spezialisierte Palliativpflegedienst ist für die Dauer des Kooperationsvertrages berechtigt, die erbrachten Leistungen direkt gegenüber dem jeweiligen Kostenträger abzurechnen. Für den Abrechnungsverkehr gilt folgendes Kennzeichen:

IK ____________________

## § 4 Kündigung

Jeder Vertragspartner kann die Kooperationsvereinbarung mit einer Frist von drei Monaten zum Ende eines Quartals kündigen.

Das Recht zur außerordentlichen Kündigung der Vereinbarung aus wichtigem Grund bleibt unbenommen. Dieses Recht besteht insbesondere, wenn der Kooperationspartner die ihm übertragenen Pflichten aus dem Rahmenvertrag gröblich verletzt.

Über die ausgesprochene Kündigung des Vertrages sind die Krankenkassen schriftlich zu informieren.

## § 5 Laufzeit

Der Kooperationsvertrag tritt mit Wirkung ab ________________________ in Kraft. Er endet automatisch, wenn die Anforderungen nach § 7 und 8 bzw. Anlage 1b des Rahmenvertrages nicht mehr gegeben sind oder wenn die Krankenkassen die Anerkennung als geeigneter spezialisierter Palliativpflegedienst entzogen haben.

Berlin, den ________________

| ______________________________ | ______________________________ |
|---|---|
| Spezialisierter Palliativarzt | Spezialisierter Palliativpflegedienst |

## Anlage 1 b zum Rahmenvertrag SAPV Anforderungen an die spezialisierten Palliativpflegedienste

### § 1 Fachliche Eignung

Geeignet sind Pflegeeinrichtungen, die neben den Voraussetzungen des § 7 des Rahmenvertrages folgende qualitative Anforderungen dauerhaft gewährleisten:

(1) Für die Durchführung der Leistungen SAPV dürfen ausschließlich Pflegefachkräfte eingesetzt werden, welche

a) eine rechtsgültige Erlaubnis zur Führung der Berufsbezeichnung "Krankenschwester" oder "Krankenpfleger", "Kinderkrankenschwester" oder "Kinderkrankenpfleger" / Gesundheits- und Krankenpfleger/in, Gesundheits- und Kinderkrankenpfleger/in Altenpfleger/Altenpflegerin entsprechend den gesetzlichen Bestimmungen in der jeweils gültigen Fassung besitzen

**und**

b) den Abschluss einer mindestens 160 Stunden umfassenden Palliative - Care - Weiterbildung (auf der Grundlage des Basiscurriculums nach Kern/Müller/Aurnhammer oder vergleichbares) nachweisen können,

**und**

c) Erfahrung durch eine mindestens einjährige praktische Tätigkeit als Pflegefachkraft in der Betreuung von Palliativpatienten in den letzten drei Jahren, davon mindestens eine sechsmonatige Mitarbeit in einer spezialisierten Einrichtung der Hospiz – oder Palliativversorgung[1] nachweisen können.

(2) fachliche Leitung

a) Die fachliche Leitung (Pflegedienstleitung nach dem Vertrag gemäß § 132 a Abs. 2 SGB V oder eine entsprechend benannte Person gemäß Absatz 2c) verfügt neben ihrer Ausbildung zur leitenden Pflegefachkraft über eine abgeschlossene Weiterbildung gemäß Absatz 1 b).

b) Verfügt die Pflegedienstleitung nicht über eine abgeschlossene Palliative - Care-Weiterbildung nach Absatz 1 b), muss die fachliche Leitung für die SAPV - Leistungserbringung durch eine Pflegefachkraft als Teamleitung, welche die Anforderungen nach Absatz 1 erfüllt, wahrgenommen werden.

c) Für den Vertretungsfall der fachlichen Leitung ist eine Stellvertretung zu benennen, welche die Voraussetzungen nach Absatz 1 erfüllt.

(3) Personalmindestvorhaltung

---

[1] Protokollnotiz: Für die Tätigkeit bei spezialisierten Palliativpflegediensten und/ oder spezialisierten Pflegeeinrichtungen im Rahmen des Home - Care Dienstes wird anerkannt, wenn der spezialisierte Palliativarzt das Vorliegen der erforderlichen Erfahrung der ihm bekannten Pflegefachkräfte bestätigt. Alternativ kann die berufspraktische Erfahrung gemäß § 1 Absatz 1 c) auch im Rahmen der SAPV Leistungserbringung erworben werden, sofern im Team insgesamt ausreichende Erfahrung zur SAPV vorliegt (Bezug: Empfehlungen des GKV Spitzenverbandes nach § 132 d Abs 2 SGB V für die SAPV von 5.11.2012, Ziffer 5.6.).

a) neben einer ständig verantwortlichen Pflegefachkraft bzw. Teamleitung sind mindestens drei weitere sozialversicherungspflichtige Pflegefachkräfte (Vollzeit) vorzuhalten, welche die Voraussetzungen gemäß Absatz 1 erfüllen.

b) Bis auf die fachliche Leitung können die beschäftigten Pflegefachkräfte auch in sozialversicherungspflichtiger Teilzeit beschäftigt werden, wobei sich die Beschäftigtenanzahl dann entsprechend erhöht.

c) Bei Verhinderung der Pflegefachkräfte nach Absatz 3 a) ist entsprechendes Vertretungspersonal einzusetzen. Beschäftigte, die der Pflegeeinrichtung ununterbrochen länger als drei Monate nicht zur Verfügung stehen (z. B. Elternzeit), werden ab diesem Zeitpunkt nicht mehr auf die Personalmindestvorhaltung angerechnet, der Personalbestand ist zu diesem Zeitpunkt anzupassen.

(4) Qualitätssicherung

Der spezialisierte Palliativpflegedienst gewährleistet:

a) die Dokumentation der SAPV in einem der Spezifik dieser Leistungen angepassten Dokumentationssystem, dass den Verlauf des Pflegeprozesses widerspiegelt;

b) eine geeignete, aktuell geführte und für alle an der Versorgung Beteiligten jederzeit zugängliche Patientendokumentation;

c) die bereits veröffentlichten und in Zukunft erarbeiteten Expertenstandards unter Berücksichtigung der Lebenssituation des Patienten anzuwenden;

d) ein internes Qualitätsmanagement entsprechend des PDCA - Zyklus (Plan – Do – Check - Act - Zyklus) durchzuführen und sich an Maßnahmen der externen Qualitätsentwicklung zu beteiligen;

e) mindestens halbjährliche Multidisziplinäre Qualitätszirkel / Fallbesprechungen zu Patienten mit Leistungen der SAPV (vgl. § 11 des Rahmenvertrages);

f) Im Auftrag der Krankenkassen kann der MDK Berlin-Brandenburg mit der Prüfung der Qualität der Leistungserbringung beauftragt werden.

g) Supervisionen für die Mitarbeiter;

h) durch regelmäßige Fortbildungen im Gesamtumfang von 16 Stunden jährlich seine Beschäftigten auf dem neuesten Stand der medizinischen und pflegefachlichen Erkenntnisse bei der Versorgung und Begleitung schwerkranker Menschen im Rahmen der SAPV zu halten;

## § 2 Voraussetzungen zur Anerkennung/ Anerkennungsverfahren

(1) Die Anerkennung als spezialisierter Palliativpflegedienst erfolgt durch die Krankenkassen. Hierzu sind folgende Unterlagen bei der Arbeitsgemeinschaft der Krankenkassenverbände in Berlin einzureichen:

a) Beglaubigte Kopien der jeweiligen rechtsgültigen Erlaubnis zur Führung der Berufsbezeichnung der Pflegefachkräfte,

b) Qualifikationsnachweise der Pflegefachkräfte,

c) Kopien der Arbeitsverträge der Pflegefachkräfte ab Beschäftigungsbeginn beim Leistungserbringer bzw. eine jeweils von Arbeitnehmer und Arbeitgeber unterschriebene Bescheinigung über das Beschäftigungsverhältnis,

d) Kopien von geeigneten Unterlagen für die Erfüllung der Mindestberufserfahrung der angestellten Pflegefachkräfte, die eine mindestens einjährige praktische Tätigkeit in der Betreuung von Palliativpatienten in den letzten drei Jahren, davon eine mindestens sechsmonatige Mitarbeit in einer spezialisierten Einrichtung der Hospiz- oder Palliativversorgung, belegen (siehe Protokollnotiz zu § 1),

e) Kopie des schriftlichen Kooperationsvertrages gemäß Muster Anlage 1 a,

f) Aufstellung der am Versorgungsnetz im Rahmen der SAPV Beteiligten mit schriftlichen Kooperationsverträgen.

(2) Die Krankenkassen bestätigen spätestens innerhalb von zwei Monaten nach vollständiger Vorlage der Unterlagen schriftlich die Anerkennung der fachlichen Eignung, die zur SAPV - Leistungserbringung berechtigt. Dies gilt soweit für die bedarfsgerechte Versorgung der Versicherten ein begründeter Bedarf besteht.

(3) Über Änderungen in der pflegefachlichen Leitung /Teamleitung bzw. bei Unterschreitung der Personalmindestvorhaltung sind die Krankenkassen unverzüglich zu informieren. Die Unterlagen gemäß § 2 Abs. 1 a) bis d) sind dann umgehend nachzureichen.

(4) Die dauerhafte Einhaltung der Personalmindestvorhaltung ist mit Angabe der Zusatzqualifikation in der Personalmeldung (Vertrag gemäß § 132 a Abs. 2 SGB V) nachzuweisen.

## § 3 Beendigung der Anerkennung / Maßnahmen bei Vertragsverstößen

(1) Die Anerkennung als spezialisierter Palliativpflegedienst kann durch die Krankenkassen mit einer Frist von drei Monaten zum Quartalsende entzogen oder durch den Leistungserbringer zurückgegeben werden. Die Erklärung bedarf der Schriftform.

(2) Bei Beendigung des Vertrages gemäß § 132 a Abs. 2 SGB V oder nach § 72 SGB XI endet die Anerkennung als spezialisierter Palliativpflegedienst zeitgleich. Einer separaten Kündigung bedarf es nicht.

(3) Das Recht zur fristlosen Aberkennung der Eignung als spezialisierter Palliativpflegedienst durch die Krankenkassen aus wichtigem Grund bleibt unberührt. Ein wichtiger Grund liegt vor,

- wenn der Leistungserbringer seine vertraglichen Verpflichtungen gegenüber den Krankenkassen derart gröblich verletzt, dass seine weitere Einbeziehung in die SAPV - Leistungserbringung nicht zumutbar ist,
- im Ergebnis der Qualitätsprüfung gemäß § 1 Absatz 4 f) im Gesamtergebnis gravierende Mängel auftreten,
- die Zusammenarbeit mit dem Kooperationspartner beendet wurde oder
- wenn ein schwerer Vertragsverstoß des Vertrages gemäß § 132 a Absatz 2 SGB V vorliegt.

Der Grundsatz der Verhältnismäßigkeit ist zu beachten.

# Anlage 2 a
# Vergütung spezialisierte Palliativärzte

Die folgenden Leistungen nach § 5 können von den am Vertrag teilnehmenden spezialisierten Palliativärzten abgerechnet werden:

**1. Beratung** — **30,00 EUR pro Beratung im SAPV-Behandlungsfall[1]**

Der spezialisierte Palliativarzt kann folgende Beratungsleistungen abrechnen:

**SNR 99060**: die Beratung des behandelnden Haus- bzw. Facharztes
**SNR 99061**: die Beratung des Versicherten und/oder dessen Angehörigen
**SNR 99062**: die Beratung der ausführenden (SAPV)-Pflegefachkraft

**2. Koordination** — **82,00 EUR einmal im SAPV-Behandlungsfall**

**SNR 99063**

Die Koordination beinhaltet die laufende Abstimmung, die Erstellung des Behandlungsplans sowie erforderliche Beratungsleistungen. Die Leistung ist einmal im SAPV-Behandlungsfall abrechenbar. Sind Beratung und/oder additiv unterstützende Teilversorgung verordnet, ist die Koordinationspauschale daneben abrechenbar.
Eine Abrechnung der Koordinationspauschale ist hingegen ausgeschlossen, wenn Vollversorgung verordnet wurde.

**3. additiv unterstützende Teilversorgung** — **105,00 EUR einmal in 7 Kalendertagen (Behandlungswoche)**

**SNR 99064**

Die Vergütung der additiv unterstützenden Teilversorgung erfolgt als Wochenpauschale und ist nicht neben der Vollversorgung nach Nr. 4 abrechenbar.

**4. vollständige Versorgung** — **68,00 EUR je Hausbesuch einmal am Tag**

**SNR 99065**

Für die vollständige SAPV-Versorgung erhält der ärztliche SAPV-Leistungserbringer 68,00 EUR je Hausbesuch einmal am Tag.
Neben der Vollversorgung ist zusätzlich die Beratungspauschale nach Nr. 1 für die Beratung des Haus- bzw. Facharztes und/oder für die Beratung des Versicherten und/oder dessen Angehörigen und/oder die Beratung der ausführenden (SAPV)-Pflegefachkraft in Höhe von jeweils 30,00 EUR einmalig im SAPV-Behandlungsfall abrechenbar.

---

[1] Der SAPV-Behandlungsfall umfasst alle durch die Krankenkasse genehmigten Zeiträume der SAPV und ist nicht mit dem vertragsärztlichen Behandlungsfall/Krankheitsfall gemäß BMV-Ä und EBM gleichzusetzen.

Eine weitere Abrechnung der Koordinationspauschale und/oder der Wochenpauschale für die additiv unterstützende Teilversorgung ist ausgeschlossen. Ist im Ausnahmefall mehr als ein Hausbesuch am Tag notwendig, so ist die Uhrzeit und eine Begründung für die Notwendigkeit eines weiteren Hausbesuches anzugeben.

**5. Hospizwochenpauschale** **124,00 EUR einmal in 7 Kalendertagen (Behandlungswoche)**

**SNR 99066**

Die Hospizwochenpauschale ist bei Vollversorgung durch den spezialisierten Palliativarzt je Behandlungszeitraum von 7 Kalendertagen (Behandlungswoche) im Hospiz abrechenbar. Neben der Hospizwochenpauschale ist vollständige Versorgung nach Nr. 4 nicht abrechenbar. Bei direktem Übergang des Patienten von der stationären Krankenhausbehandlung in eine Hospizversorgung ist die Beratung nach Nr. 1 nicht abrechenbar.

**6. HB-Zuschlag für SAPV-Assistenten**

**SNR 99067** **25,00 EUR je Besuchsleistung**

Für die Erbringung von Besuchsleistungen einschließlich der Wege erhält der angestellte SAPV-Assistent je Besuchsleistung 25 EUR. Die Besuchsleistung ist neben den SNR 99064, 99065 und 99066 abrechenbar. Neben der SNR 99066 ist die Besuchsleistung in Höhe von 25 EUR höchstens zweimal pro Behandlungswoche abrechenbar.

**7. Sonstiges**

Die Dokumentation gemäß § 11 Absatz 3 ist mit der Vergütung nach dieser Anlage abgegolten.

Eine Abrechnung der weiteren ärztlichen Leistungen auf der Grundlage des EBM und der jeweils gültigen Honorarvereinbarung zur vertragsärztlichen Versorgung für den KV-Bereich Berlin ist neben den Leistungen nach dieser Vereinbarung nur den Vertragsärzten vorbehalten. Spezialisierten Palliativärzten im Anstellungsverhältnis ist neben den genannten Symbolnummern lediglich die Abrechnung der SAPV-Folgeverordnung mittels Muster 63 der EBM-GOP 01426 zulässig.

Ab dem Zeitpunkt, ab dem die Leistungen nach Nr. 4 und 5 erbracht werden, ist die Abrechnung der Kostenpauschale 86518 nicht neben den Leistungen nach Nr. 4 und 5 dieser Vereinbarung abrechenbar.

## Anlage 2 b) Vergütung für die spezialisierten Palliativpflegedienste

(1) Vom **01.07.2013** bis mindestens 30.06.2015 sind für die im Rahmen ärztlich verordneter spezialisierter Palliativpflege und von spezialisierten Palliativpflegediensten erbrachten Leistungen folgende Entgelte abrechnungsfähig:

| Nr. | Leistung | Einheit | DTA Gebühren-positions-nummern (§ 302 SGB V)[1] | Entgelt-pauschale |
|---|---|---|---|---|
| 1 | **Erstbesuchspauschale**<br>- Beratung, Anleitung und Begleitung der Patienten und ihrer Angehörigen zur palliativpflegerischen Versorgung einschließlich Unterstützung beim Umgang mit Sterben und Tod<br>- psychosoziale Unterstützung des Patienten und anderer am Prozess Beteiligten im persönlichen Umfeld im Umgang mit schweren Erkrankungen in enger Zusammenarbeit z. B. mit Seelsorge, Sozialarbeit und ambulanten Hospizdiensten.<br>- Die Beratungsleistung kann sowohl von Patienten und Angehörigen als auch von an der allgemeinen Versorgung beteiligten Leistungserbringern als Fachberatung in Anspruch genommen werden.<br>Die Beratungsleistung umfasst mindestens ein Beratungsassessment beim anspruchsberechtigten Versicherten. Sie wird mit einer Handlungsempfehlung abgeschlossen. | einmal je SAPV Behandlungsfall[2] | xxxxxxxxxx | **120,00 EUR** |
| 2 | **Koordination und Fallbesprechungen** | | | |
| 2a | **Aufwendungen für:**<br>- die Koordination der spezialisierten palliativpflegerischen Versorgung unter Einbeziehung weiterer Berufsgruppen und von Hospizdiensten im Rahmen einer multiprofessionellen Zusammenarbeit,<br>- das Abstimmen der erforderlichen Maßnahmen auf den individuellen Interventionsbedarf unter Berücksichtigung des aus ärztlicher und pflegerischer Sicht erforderlichen Entscheidungsspielraums, sowie deren Dokumentation | je angefangene Behandlungswoche[3] | xxxxxxxxxx | **25,00 EUR** |

[1] Das bundeseinheitliche Positionsnummernverzeichnis für Leistungen der SAPV wurde in den Richtlinien des GKV Spitzenverbandes vom 05.09.2011 veröffentlicht, zuletzt geändert am 05.12.2012; hier als Platzhalter aufgenommen. Die Anwendung auf den Rechnungen ist bis auf weiteres noch nicht erforderlich.

[2] Der SAPV Behandlungsfall umfasst alle durch die Krankenkasse genehmigten Zeiträume der SAPV und ist nicht mit dem vertragsärztlichen Behandlungsfall/Krankheitsfall gemäß BMV –Ä und EBM gleichzusetzen.

[3] Die Wochenpauschale bezieht sich auf einen Zeitraum von bis zu 7 Tagen und stellt nicht auf eine Kalenderwoche ab.

| Nr. | Leistung | Einheit | DTA Gebühren-positions-nummern (§ 302 SGB V)[1] | Entgelt-pauschale |
|---|---|---|---|---|
| 2b | **Patientenbezogene interdisziplinäre Fallbesprechungen**<br>Telefonische Abstimmungen gelten nicht als Fallbesprechung, siehe Ziffer 2a Koordinierung. | je angefangene Behandlungs-woche | xxxxxxxxxx | 25,00 EUR |
| 3 | **Spezialisierte palliativpflegerische Leistungen** | | | |
| 3a | spezialisierte palliativpflegerische Leistungen, die nach ihrer Art, Schwere oder Komplexität eine Kompetenz erfordern, die der einer Pflegefachkraft mit einer curricularen Weiterbildung zu Palliative Care entspricht. Durchführung von medikamentösen oder anderen Maßnahmen zur Symptomlinderung. | je angefangene 35 Minuten Anwesenheit im Haushalt[4] | xxxxxxxxxx | 19,44 EUR |
| 3b | Einsätze zur Krisenintervention | je Einsatz | xxxxxxxxxx | 35,50 EUR |
| 3c | Zuschlag zum Entgelt gemäß Position 3a bei Infusionstherapie oder bei Überwachung bei / nach Aszitispunktion / Pleurapunktion mit notwendiger Anwesenheitspflicht der Pflegefachkraft | je Einsatz | xxxxxxxxxx | 7,50 EUR |

(2) Die Vergütung nach Absatz 1 gilt für erbrachte SAPV Pflegeleistungen im Haushalt des schwerstkranken Menschen oder seiner Familie sowie in stationären Pflegeeinrichtungen (§ 72 Abs. 1 SGB XI). § 1 Absatz 2 der SAPV-RL gilt entsprechend.

(3) Während eines Aufenthaltes des Versicherten in einem stationären Hospiz sind die Entgelte nach Absatz 1 nicht abrechnungsfähig.

(4) Einsätze zur Krisenintervention nach Absatz 1 Nr. 3b dieser Vereinbarung sind abrechnungsfähig, wenn sich die Pflegesituation des Versicherten akut so verschlechtert hat, dass ohne eine sofortige Intervention die weitere ambulante Versorgung nicht mehr möglich ist (z.B. akutes Auftreten von Atemnot, unbeherrschbare Schmerzen, akute Somnolenz usw.) und der spezialisierte Leistungserbringer Palliativpflege außerplanmäßig einen Hausbesuch durchführt.

---

[4] In den der Abrechnung beizufügenden Leistungsnachweisen ist Beginn und Ende der Leistungserbringung gemäß Position 3a aufzuzeichnen.

(5) Mit den genannten Entgelten nach Absatz 1 sind jeweils sämtliche im Zusammenhang mit den bewilligten und erbrachten Leistungen der SAPV erforderlichen Aufwendungen abgegolten. Im Sinne einer umfassenden qualitätsgesicherten Versorgung der Versicherten ist die im Rahmen der SAPV ggf. erforderliche Behandlungspflege nach § 37 Abs. 2 SGB V als Bestandteil der verordneten SAPV Leistungen durch den spezialisierten Palliativpflegedienst sicherzustellen.[5]

(6) Die Krankenkassen begleichen die Rechnungen gegenüber dem spezialisierten Leistungserbringer Palliativpflege innerhalb von 28 Tagen nach Rechnungseingang mit befreiender Wirkung unter Angabe der Rechnungsnummer und des Rechnungsdatums. Als Zahltag gilt der Tag der Überweisung oder der Tag der Übergabe des Überweisungsauftrages an ein Geldinstitut.

(7) Der Inhalt der Abrechnung richtet sich grundsätzlich nach den Richtlinien der Spitzenverbände der Krankenkassen über Form und Inhalt des Abrechnungsverfahrens mit den sonstigen Leistungserbringern nach § 302 Absatz 2 SGB V (Datenträgeraustausch) und der technischen Anlagen in der jeweils gültigen Fassung und umfasst:

1. Abrechnungsdaten, je Abrechnungsbeleg sind mindestens zu übermitteln:
   - Institutionskennzeichen des spezialisierten Palliativpflegedienstes
   - Name, Anschrift, Telefonnummer des Leistungserbringers
   - Rechnungsnummer
   - Krankenversicherungsnummer des Versicherten, Name, Vorname, Geburtsdatum, Versichertenstatus
   - Institutionskennzeichen des Kostenträgers
   - abzurechnender Zeitraum
   - abzurechnende Leistungen nach Art, Menge, Einzel- und Gesamtvergütungsbetrag
2. Urbelege (Verordnungen im Original)
3. Leistungszusagen (im Original)
4. Leistungsnachweis (im Original)

Der einsatzbezogene Erhalt der Leistungen ist vom Versicherten, ggf. bevollmächtigten Angehörigen oder Vertreter, auf dem Leistungsnachweis mit Unterschrift und Datum zu bestätigen. In zu begründenden Ausnahmefällen kann der Leistungserbringer den Leistungsnachweis unterschriftlich bestätigen. Vordatierungen oder Globalbestätigungen über den Empfang von Leistungen sind unzulässig. Im Leistungsnachweis sind alle erbrachten Leistungen nach Art, Menge, Datum und Uhrzeit aufzuschlüsseln.

5. Gesamtaufstellung der Abrechnung

Der spezialisierte Palliativpflegedienst hat auf dem Abrechnungsbeleg mit seiner Unterschrift zu bestätigen, dass die in Rechnung gestellten Leistungen nach Art und Häufigkeit erbracht worden sind.

Rechnungen, die den Anforderungen nicht entsprechen, können unter Angabe des Grundes zur Berichtigung an den Leistungserbringer zurückgegeben werden. Offensichtliche Rechen- oder Schreibfehler sollten unbürokratisch abgeklärt werden. Rechnungsbeanstandungen muss die Krankenkasse innerhalb von sechs Monaten nach Rechnungseingang erheben. Einsprüche gegen Beanstandungen können vom Leistungserbringer innerhalb von drei Monaten nach Mitteilung geltend gemacht werden.

---

[5] Protokollnotiz zu Absatz 5:
Im Interesse der Klarstellung wird folgendes abgestimmt

1. SAPV Pflegeleistungen werden bei der Teilversorgung in der Regel ergänzend zu den HKP- Leistungen eines anderen Pflegedienstes erbracht.

2. Im Rahmen der SAPV-Vollversorgung sind die ggf. erforderlichen Behandlungspflegen nach § 37 Absatz 2 SGB V als Bestandteil der verordneten SAPV Leistungen durch den spezialisierten SAPV Pflegedienst sicher zu stellen.

**Patientendokumentation SAPV** **Home Care Berlin e.V.** **201**

## Praxisrelevante Angaben

**Praxis-Stempel**

**verantwortliche(r )Arzt / Ärzte SAPV**

1.____________________

2.____________________

## Situation bei Aufnahme in die SAPV

**Geburtsjahr Patient** ☐☐☐☐

**Geschlecht** ● männlich ● weiblich

**Wohnverhältnis beim Erstbesuch**
- ● Wohnung, allein
- ● Wohnung, mit Angehörigen
- ● Senioren-/Pflegeheim
- ● sonstiges

**Krankenkasse oder KK-Betriebsnummer**

____________________ ☐☐☐☐☐☐☐☐

**SAPV verordnet durch** ★
- ● Facharzt
- ● Hausarzt
- ● Heimarzt
- ● Palliativstation
- ● andere Khs.-abteilung
- ● selbst (SAPV-Arzt)

sonstiger Zugang: ____________________

**Ziel der Versorgung** ★
- ● Optimierung der Schmerztherapie
- ● Therapie anderer Symptome
- ● Behandlung von Begleiterkrankungen
- ● psychische Stabilisierung des Patienten
- ● Stabilisierung der Angehörigen
- ● Organisation des SAPV-Netzes

**Umfang der SAPV** ★
- ● telefonische Beratung von Betroffenen
- ● Beratung von Betroffenen vor Ort
- ● telefonische Beratung des primärvers. Arztes
- ● Beratung primärvers. Arzt vor Ort (Konsil)
- ● Teilversorgung mit dem primärvers. Arzt
- ● Vollversorgung
- ● Koordination der ambulanten Versorgung

## Diagnose(n), die die SAPV begründen

**Hauptdiagnose** **ICD** ☐☐☐

## Symptomgeschehen beim ersten Besuch

- ● ausgeprägte Schmerzsymptomatik
- ● ausgeprägte neurologische/psychiatrische/psychische Symptomatik
- ● ausgeprägte respiratorische/kardiale Symptomatik
- ● ausgeprägte gastrointestinale Symptomatik
- ● ausgeprägte ulcerierende/ exulcerierende Wunden oder Tumore
- ● ausgeprägte urogenitale Symptomatik
- ● ausgeprägte Überforderung des sozialen Umfelds
- ● sonstiges komplexes Symptomgeschehen

## Betreuungsverlauf

**Datum des ersten Kontakts** ☐☐☐☐☐☐

**Datum des ersten Besuchs** ☐☐☐☐☐☐

**Ort des ersten Besuchs**
- ● (eigene) Wohnung
- ● Senioren-/Pflegeheim
- ● stationäres Hospiz
- ● Kurzzeitpflege
- ● Krankenhaus

**Datum des letzten Kontakts** ☐☐☐☐☐☐

**Datum des letzten Besuchs** ☐☐☐☐☐☐

**Ort des letzten Besuchs**
- ● (eigene) Wohnung
- ● Senioren-/Pflegeheim
- ● stationäres Hospiz
- ● Kurzzeitpflege
- ● Krankenhaus

**SAPV abgeschlossen am:** ☐☐☐☐☐☐

**Begründung des Abschlusses** ★
- ● Tod des Patienten
- ● Änderung des Wohnorts des Patienten
- ● Stabilisierung des Gesundheitszustandes
- ● neue kausale Therapieoptionen
- ● Krankenhauseinweisung
- ● Wechsel des SAPV-Arztes / Teams

**Sterbedatum** ☐☐☐☐☐☐

**Sterbeort**
- ● (eigene) Wohnung
- ● Senioren-/Pflegeheim
- ● stationäres Hospiz
- ● Kurzzeitpflege
- ● Palliativstation
- ● andere Krkhs.-Station
- ● unbekannt
- ● sonstiges (Transport...)

## Temporäre Krankenhauseinweisungen während der gesamten SAPV - Zeit

**Gesamtzahl der Krankenhauseinweisungen *zwischen erstem und letztem Besuch*** ☐☐

**Summe der im Krankenhaus verbrachten Tage *zwischen erstem und letztem Besuch*** ☐☐

## Letzte Einweisung / Verlegung, die mit dem Tod des Patienten endete (ausfüllen, wenn der Sterbeort nicht der Versorgungsort zu Beginn ist!)

**Datum der Einweisung / Verlegung ins Krankenhaus / Heim / stationäre Hospiz:** ☐☐☐☐☐☐

**Tageszeit** ● zwischen 6 und 18 Uhr ● übrige Zeit

**Wer hat die Einweisung veranlasst?**
- ● Patient / Angehörige
- ● SAPV Arzt
- ● Hausarzt
- ● Notarzt
- ● Pflegedienst / Pflegepersonal
- ● unbekannt

**Was war(en) die Ursache(n)?** ★
- ● medizinische Notfallsituation
- ● sonstige medizinische Indikation
- ● Dekompensation Patient/Angehörige
- ● (häusliche) Pflege nicht gesichert
- ● SAPV-Arzt nicht verfügbar
- ● diagnostische Abklärung
- ● unbekannt

## Versorgungsziffern

**Zahl der konsiliarischen Telefonate** ☐☐

**Zahl der Konsiliarbesuche** ☐☐

**Zahl der Hausbesuche im Rahmen einer Teilversorgung** ☐☐

**Zahl der Hausbesuche im Rahmen einer Vollversorgung** ☐☐

## Einbezogene Leistungserbringer während der SAPV-Zeit ★

- ● Physiotherapie
- ● ambulanter Hospizdienst
- ● Seelsorger
- ● Psychologen / -therapeuten
- ● Sozialarbeiter
- ● nichtspez. Pflegedienst
- ● SAPV-Pflegedienst: (welcher) ____________________

**Datum** **Unterschrift**

★= *(alles Zutreffende ankreuzen)*

Copyright by Home Care Berlin e.V.

## Anlage 3 b) SAPV - Patientendokumentation Pflege

Die SAPV - Patientendokumentation Pflege gemäß §§ 10 und 11 Abs. 5 wird zunächst im Rahmen der bisher in den SAPV Pflegediensten einrichtungs- und patientenbezogen geführten Pflegedokumentation erfasst.

Der bisherige Entwurf zur SAPV - Patientendokumentation Pflege in der Fassung vom 20.08.2013 wird bis 31.12.2013 in Analogie zur SAPV - Patientendokumentation Arzt überarbeitet und gemeinsam mit diesem eingeführt.

Eine Weiterleitung dieser Patientendokumentationen an Dritte erfolgt erst, wenn die datenschutzrechtlichen Bedenken der Beteiligten ausgeräumt sind.

Kassenärztliche Vereinigung Berlin
Abteilung Qualitätssicherung
Masurenallee 6A
14057 Berlin

Telefon (030) 31003 - 524, Fax (030) 31003 - 305

Praxisstempel

# Teilnahmeerklärung
# an der spezialisierten ambulanten Palliativversorgung (SAPV)

**nach dem Rahmenvertrag gemäß 132 d SGB V vom 06.07.2010, fortgeführt in der Fassung zum 01.10.2013**

Name des Antragstellers: ______________________________

Betriebsstättennummer (BSNR): ☐☐☐☐☐☐☐☐☐

**Die Antragstellung erfolgt:** ☐ für mich

☐ für den angestellten Arzt/ Job-Sharer ______________
(nicht Zutreffendes bitte streichen) (Name des Arztes)

☐ Niedergelassener Arzt in ☐ Einzelpraxis ☐ Berufsausübungsgemeinschaft ☐ MVZ ☐ ÜBAG ☐ Sonstige

☐ Angestellter Arzt in ☐ Einzelpraxis ☐ Berufsausübungsgemeinschaft ☐ MVZ ☐ ÜBAG ☐ Sonstige

Lebenslange Arztnummer (LANR):
(wenn vorhanden) ☐☐☐☐☐☐☐☐☐

Telefon (tagsüber): ______________ E-Mail Adresse ______________

**Hiermit beantrage ich für mich bzw. für den angegebenen angestellten Arzt die Teilnahme an dem o.g. Vertrag über die spezialisierte ambulante Palliativversorgung und**

☐ besitze bzw. der angestellte Arzt besitzt die abgeschlossene Weiterbildung Palliativmedizin gemäß der Weiterbildungsordnung der Landesärztekammern *(§ 7 Abs. 1 Buchstabe b)*

*(Die Urkunde ist in Kopie der Teilnahmeerklärung beigefügt)*

**und**

☐ habe den Nachweis über die selbständige ambulante Versorgung von mind. 75 Palliativpatienten innerhalb der letzten 3 Jahre beigefügt *(§ 7 Abs. 1 Buchstabe c)*

*(formlose Patientenliste, aus der die Krankenkasse, Versichertennummer, Diagnose und der palliativmedizinische Betreuungszeitraum der Palliativpatienten hervorgehen, ist beigefügt)*

**oder**

☐ habe den Nachweis über eine mind. einjährige klinische palliativmedizinische Tätigkeit in einer Palliativabteilung eines Krankenhauses innerhalb der letzten 3 Jahre beigefügt *(§ 7 Abs. 1 Buchstabe c)*

*(Bescheinigung/Zeugnis in Kopie ist beigefügt)*

**oder**

☐ verpflichte mich bzw. der angestellte Arzt verpflichtet sich, den Nachweis über die selbständige ambulante Versorgung von mind. 75 Palliativpatienten in einem Zeitraum von 12 Monaten ab Teilnahmebestätigung gegenüber der KV Berlin nachzuholen *(§ 7 Abs. 1 Buchstabe e)*

**und**

☐ habe den Nachweis einer Kooperationsvereinbarung nach Anlage 1a mit mind. einem von den Krankenkassen zugelassenen spezialisierten Palliativpflegedienst beigefügt *(§ 7 Abs. Buchstabe d)*

*(Kooperationsvereinbarung ist beigefügt)*

---

**Hiermit versichere ich als Palliativarzt, dass folgende Mindestanforderungen an die sächliche Ausstattung gemäß § 7 Abs. 5 des o.g. Vertrages vorgehalten werden:**

a) Eine geeignete, aktuell geführte und für die an der Versorgung Beteiligten jederzeit zugängliche Patientendokumentation ☐

b) Arztkoffer/Bereitschaftstasche (Berücksichtigung der Kompatibilität der Verbrauchsmaterialien zu Medizinprodukten unterschiedl. Hersteller, z.B. bei Portsystemen oder Infusionspumpen) ☐

c) Eine geeignete administrative Infrastruktur, z.B. Büro, Kommunikationstechnik ☐

d) Arzneimittel (inklusive Betäubungsmittel) für die Notfall-/Krisenintervention ☐

**Hiermit verpflichte ich mich als Palliativarzt, dass eine eigenständige Adresse und geeignete Räumlichkeiten für**

- die Beratung von Versicherten und Angehörigen,
- Teamsitzungen und Besprechungen und
- die Lagerhaltung von eigenen Medikamenten für Notfall-/Krisenintervention und Hilfsmittel zur Verfügung stehen (*§ 7 Abs. 6*).

**Ich bin bzw. der angestellte Palliativarzt ist umfassend über die Ziele und Inhalte des Vertrages über die SAPV in Berlin informiert. Der Vertragsinhalt ist mir bzw. dem angestellten Palliativarzt bekannt.**

1. **Mir bzw. dem angestellten Palliativarzt ist insbesondere bekannt,**
   - *dass die Einhaltung der Anforderungen an den Umgang mit Betäubungsmitteln im Sinne des BTMG durch den Palliativarzt sicherzustellen ist (§ 7 Abs. 6),*
   - *dass eine 24-Stunden-Bereitschaft zu gewährleisten ist und auch bei z.B. urlaubs- oder krankheitsbedingter Abwesenheit die ständige Verfügbarkeit eines spezialisierten Palliativarztes sichergestellt sein muss (§ 10 Abs.3),*
   - *dass der spezialisierte Palliativarzt im Rahmen der Koordination den individuellen Behandlungsplan erstellen und sich mit den beteiligten Leistungserbringern abstimmen muss (§ 10 Abs. 4),*
   - *dass die Leistungen gemäß § 11 dokumentiert und die Dokumentationsbögen gemäß Anlage 3a bis spätestens zum 15.01. des Folgejahres an den HC e.V. zur Erstellung des Qualitätsberichts übersandt werden müssen (§ 10 Abs. 4),*
   - *dass der spezialisierte Palliativarzt im Rahmen der Qualitätssicherung verpflichtet ist, ein internes Qualitätsmanagement durchzuführen, regelmäßig an multiprofessionellen Fortbildungen zur Palliativmedizin teilzunehmen, halbjährlich multidisziplinäre Fallbesprechungen durchzuführen und sich an Maßnahmen der externen Qualitätssicherung zu beteiligen (§ 11 Abs. 2),*
   - *dass die Teilnahme an dem Vertrag endet, wenn die Teilnahmevoraussetzungen des Vertrages nicht mehr erfüllt sind oder eine Kündigung des Vertrages erfolgt (§ 9).*
2. **Ich stimme der namentlichen Veröffentlichung** (Titel, Vorname und Name zusammen mit meiner Praxisanschrift und meiner Praxistelefonnummer) im Verzeichnis über die „SAPV-Ärzte" bzw. „SAPV-Praxen" **auf der Homepage der KV Berlin zu.**
3. Notwendigen Vertragsänderungen oder –anpassungen stimme ich zu.
4. Mir ist bekannt, dass die Ausführung und Abrechnung der genehmigungspflichtigen Leistungen der spezialisierten ambulanten Palliativversorgung in der vertragsärztlichen Versorgung erst **nach Erteilung der Teilnahmebestätigung durch die KV Berlin im Auftrag der Krankenkassen zulässig** ist.

**Ich versichere die Richtigkeit der Angaben.**

Berlin, den ...........................................................

| | |
|---|---|
| ............................................................. | ............................................................. |
| Unterschrift des Antragstellers | ggf. Unterschrift des Leiters (Einrichtung/MVZ) |

**Anlage 5: Teilnahmeerklärung des Leistungserbringers „Spezialisierter Palliativpflegedienst**

AOK Nordost – Die Gesundheitskasse
Geschäftsbereich Pflege
Team Spezialisierte Pflegeangebote
14456 Potsdam

Stempel des spezialisierten Palliativpflegedienstes

**Teilnahmeerklärung**

**zum Rahmenvertrag nach § 132d SGBV über die spezialisierte ambulante Palliativversorgung,**

**gültig ab dem 01.10.2013**

Name der verantwortlichen Leitung: ______________________________

IK: ____________________

Die Antragstellung erfolgt für

☐ den SAPV – Pflegedienst (Anschrift, Tel., Fax., E-Mail) :

______________________________

______________________________

______________________________

Träger:

______________________________

☐ Die Voraussetzungen gemäß § 8 Abs. 1.b des SAPV - Rahmenvertrages liegen vor.
(Nachweise dazu liegen der Teilnahmeerklärung bei)

Hiermit beantrage ich verbindlich die Teilnahme zum SAPV - Rahmenvertrag in Berlin.

1. Ich bin umfassend über die Ziele und Inhalte des SAPV - Rahmenvertrages in Berlin informiert. Der Vertragsinhalt ist mir bekannt.
2. Mir ist besonders bekannt,
   a. dass die Anforderungen gemäß § 7 und Anlage 1 zu erfüllen sind.
   b. ein Anspruch auf Teilnahme nicht besteht.
   c. dass die Teilnahme an diesem Vertrag mit schriftlicher Bestätigung der Teilnahme gemäß § 8 Abs. 1.b beginnt und damit die Berechtigung und Verpflichtung zur Leistungserbringung nach diesem Vertrag besteht,
   d. dass die Teilnahme an diesem SAPV- Rahmenvertrag endet, wenn die Voraussetzungen gemäß § 8 Abs. 2 Satz 3 und § 9 des Vertrages erfüllt sind, oder eine Kündigung gemäß § 18 des Vertrages erfolgte.

______________________________

Ort, Datum Unterschrift des antragstellenden Trägers

**2. Änderungsvereinbarung**

**zum**
**Rahmenvertrag über die spezialisierte ambulante Palliativversorgung**
**gemäß § 132d SGB V in Berlin vom 16.09.2013**

**zwischen**

**der Kassenärztlichen Vereinigung Berlin**
**- im Folgenden KV Berlin genannt -**
**und**

**dem**
**Home Care Berlin e. V.**
**- im Folgenden HC e. V. genannt-**

**und dem**

**Berliner Aktionsbündnis ambulante Palliativpflege e. V.**
**- im Folgenden BAAP e. V. genannt -**

**sowie**

**der AOK Nordost - Die Gesundheitskasse**

**Mit den Ersatzkassen:**
- **Techniker Krankenkasse (TK)**
- **BARMER GEK**
- **DAK - Gesundheit**
- **Kaufmännische Krankenkasse – KKH**
- **HEK - Hanseatische Krankenkasse**
- **Handelskasse (hkk)**

**gemeinsamer Bevollmächtigter mit Abschlussbefugnis:**
**Verband der Ersatzkassen e. V. (vdek),**
**vertreten durch den Leiter der vdek-Landesvertretung**
**Berlin/Brandenburg**

**dem BKK Landesverband Mitte,**
**Siebstraße 4, 30171 Hannover,**

**der BIG direkt gesund,**

**der IKK Brandenburg und Berlin,**

**der Knappschaft - Regionaldirektion Berlin,**

**der Sozialversicherung für Landwirtschaft, Forsten und Gartenbau**
**(SVLFG) als Landwirtschaftliche Krankenkasse, Hoppegarten**

**- im Folgenden Krankenkassen genannt -**

## Anlage 2 b) Vergütung für die spezialisierten Palliativpflegedienste

(1) Vom **01.01.2016** bis mindestens **31.12.2017** sind für die im Rahmen ärztlich verordneter spezialisierter Palliativpflege und von spezialisierten Palliativpflegediensten erbrachten Leistungen die nachfolgenden Entgelte abrechnungsfähig.

| Nr. | Leistung | Einheit | DTA Gebühren-positions-nummern (§ 302 SGB V)[1] | Entgelt-pauschale |
|---|---|---|---|---|
| 1 | **Erstbesuchspauschale**<br>- Beratung, Anleitung und Begleitung der Patienten und ihrer Angehörigen zur palliativpflegerischen Versorgung einschließlich Unterstützung beim Umgang mit Sterben und Tod.<br>- Psychosoziale Unterstützung des Patienten und anderer am Prozess Beteiligten im persönlichen Umfeld im Umgang mit schweren Erkrankungen in enger Zusammenarbeit z. B. mit Seelsorge, Sozialarbeit und ambulanten Hospizdiensten.<br>- Die Beratungsleistung kann sowohl von Patienten und Angehörigen als auch von an der allgemeinen Versorgung beteiligten Leistungserbringern als Fachberatung in Anspruch genommen werden.<br>Die Beratungsleistung umfasst mindestens ein Beratungsassessment beim anspruchsberechtigten Versicherten. Sie wird mit einer Handlungsempfehlung abgeschlossen. | einmal je SAPV-Behandlungsfall[2] | 003010 1002 Privater Haushalt<br>103010 1002 Stationäre Pflegeeinrichtung<br>203010 1002 Teilstationäre Pflegeeinrichtung<br>403010 1002 Einrichtungen der Eingliederungshilfe<br>503010 1002 Einrichtungen der Kinder – und Jugendhilfe<br>603010 1002 Sonstiger Ort | **135,00 EUR** |
| 2 | **Koordination und Fallbesprechungen** | | | |
| 2a | **Aufwendungen für:**<br>- die Koordination der spezialisierten palliativpflegerischen Versorgung unter Einbeziehung weiterer Berufsgruppen und von Hospizdiensten im Rahmen einer multiprofessionellen Zusammenarbeit,<br>- das Abstimmen der erforderlichen Maßnahmen auf den individuellen Interventionsbedarf unter Berücksichtigung des aus ärztlicher und pflegerischer Sicht erforderlichen Entscheidungsspielraums, sowie deren Dokumentation. | je angefangene Behandlungswoche[3] | 003020 5100<br>103020 5100<br>203020 5100<br>403020 5100<br>503020 5100<br>603020 5100 | **30,00 EUR** |

[1] Das bundeseinheitliche Positionsnummernverzeichnis für Leistungen der SAPV wurde in den Richtlinien des GKV-Spitzenverbandes veröffentlicht, zuletzt vom 17.06.2015 (Bundeseinheitliches Positionsnummernverzeichnis SAPV).

[2] Der SAPV-Behandlungsfall umfasst alle durch die Krankenkasse genehmigten Zeiträume der SAPV und ist nicht mit dem vertragsärztlichen Behandlungsfall/Krankheitsfall gemäß BMV –Ä und EBM gleichzusetzen.

[3] Die Wochenpauschale bezieht sich auf einen Zeitraum von bis zu sieben Tagen und stellt nicht auf eine Kalenderwoche ab.

| Nr. | Leistung | Einheit | DTA Gebühren-positions-nummern (§ 302 SGB V)[1] | Entgelt-pauschale |
|---|---|---|---|---|
| 2b | **Patientenbezogene interdisziplinäre Fallbesprechungen**<br><br>Telefonische Abstimmungen gelten nicht als Fallbesprechung, siehe Ziffer 2a Koordinierung. | je angefangene Behandlungswoche | 003020 5004<br>103020 5004<br>203020 5004<br>403020 5004<br>503020 5004<br>603020 5004 | **30,00 EUR** |
| 3 | **Spezialisierte palliativpflegerische Leistungen** | | | |
| 3a | spezialisierte palliativpflegerische Leistungen, die nach ihrer Art, Schwere oder Komplexität eine Kompetenz erfordern, die der einer Pflegefachkraft mit einer curricularen Weiterbildung zu Palliative-Care entspricht. Durchführung von medikamentösen oder anderen Maßnahmen zur Symptomlinderung. | je angefangene 35 Minuten Anwesenheit im Haushalt[4] | 003040 3101<br>103040 3101<br>203040 3101<br>403040 3101<br>503040 3101<br>603040 3101 | **22,75 EUR** |
| 3b | Einsätze zur Krisenintervention | je Einsatz | 003040 2104<br>103040 2104<br>203040 2104<br>403040 2104<br>503040 2104<br>603040 2104 | **39,00 EUR** |
| 3c | Zuschlag zum Entgelt gemäß Position 3a bei Infusionstherapie oder bei Überwachung bei/nach Aszitispunktion/Pleurapunktion mit notwendiger Anwesenheitspflicht der Pflegefachkraft. | je Einsatz | 003040 8003<br>103040 8003<br>203040 8003<br>403040 8003<br>503040 8003<br>603040 8003 | **9,00 EUR** |

(2) Mit der Vergütungserhöhung für die Jahre 2016 und 2017 ist dauerhaft eine Erhöhung der Entgelte der in der SAPV tätigen Pflegefachkräfte von jeweils durchschnittlich 2,5 % vorzunehmen.

[4] In den der Abrechnung beizufügenden Leistungsnachweisen ist Beginn und Ende der Leistungserbringung gemäß Position 3a aufzuzeichnen.

(3) Die Vergütung nach Absatz 1 gilt für erbrachte SAPV-Pflegeleistungen im Haushalt des schwerstkranken Menschen oder seiner Familie sowie in stationären Pflegeeinrichtungen (§ 72 Abs. 1 SGB XI). § 1 Absatz 2 der SAPV-RL in der jeweils geltenden Fassung gilt entsprechend.

(4) Während eines Aufenthaltes des Versicherten in einem stationären Hospiz sind die Entgelte nach Absatz 1 nicht abrechnungsfähig.

(5) Einsätze zur Krisenintervention nach Absatz 1 Nr. 3b dieser Vereinbarung sind abrechnungsfähig, wenn sich die Pflegesituation des Versicherten akut so verschlechtert hat, dass ohne eine sofortige Intervention die weitere ambulante Versorgung nicht mehr möglich ist (z. B. akutes Auftreten von Atemnot, unbeherrschbare Schmerzen, akute Somnolenz usw.) und der spezialisierte Leistungserbringer Palliativpflege außerplanmäßig einen Hausbesuch durchführt.

(6) Mit den genannten Entgelten nach Absatz 1 sind jeweils sämtliche im Zusammenhang mit den bewilligten und erbrachten Leistungen der SAPV erforderlichen Aufwendungen abgegolten. Im Sinne einer umfassenden qualitätsgesicherten Versorgung der Versicherten ist die im Rahmen der SAPV ggf. erforderliche Behandlungspflege nach § 37 Abs. 2 SGB V als Bestandteil der verordneten SAPV-Leistungen durch den spezialisierten Palliativpflegedienst sicherzustellen.[5]

(7) Die Krankenkassen begleichen die Rechnungen gegenüber dem spezialisierten Leistungserbringer Palliativpflege innerhalb von 28 Tagen nach Rechnungseingang mit befreiender Wirkung unter Angabe der Rechnungsnummer und des Rechnungsdatums. Als Zahltag gilt der Tag der Überweisung oder der Tag der Übergabe des Überweisungsauftrages an ein Geldinstitut.

(8) Der Inhalt der Abrechnung richtet sich grundsätzlich nach den Richtlinien der Spitzenverbände der Krankenkassen über Form und Inhalt des Abrechnungsverfahrens mit den sonstigen Leistungserbringern nach § 302 Absatz 2 SGB V (Datenträgeraustausch) und der technischen Anlagen in der jeweils gültigen Fassung und umfasst:

1. Abrechnungsdaten, je Abrechnungsbeleg sind mindestens zu übermitteln:
   - Institutionskennzeichen des spezialisierten Palliativpflegedienstes
   - Name, Anschrift, Telefonnummer des Leistungserbringers
   - Rechnungsnummer
   - Krankenversicherungsnummer des Versicherten, Name, Vorname, Geburtsdatum, Versichertenstatus
   - Institutionskennzeichen des Kostenträgers
   - abzurechnender Zeitraum
   - abzurechnende Leistungen nach Art, Menge, Einzel- und Gesamtvergütungsbetrag
2. Urbelege (Verordnungen im Original)
3. Leistungszusagen (im Original)

---

[5] Protokollnotiz zu Absatz 6:

Im Interesse der Klarstellung wird folgendes abgestimmt

1. SAPV-Pflegeleistungen werden bei der Teilversorgung in der Regel ergänzend zu den HKP-Leistungen des SAPV-Pflegedienstes oder eines anderen Pflegedienstes erbracht.
2. Im Rahmen der SAPV-Vollversorgung sind die ggf. erforderlichen Behandlungspflegen nach § 37 Absatz 2 SGB V als Bestandteil der verordneten SAPV-Leistungen durch den spezialisierten SAPV-Pflegedienst sicher zu stellen.

4. Leistungsnachweis (im Original)
   Der einsatzbezogene Erhalt der Leistungen ist vom Versicherten, ggf. bevollmächtigten Angehörigen oder Vertreter, auf dem Leistungsnachweis mit Unterschrift und Datum zu bestätigen. In zu begründenden Ausnahmefällen kann der Leistungserbringer den Leistungsnachweis unterschriftlich bestätigen. Vordatierungen oder Globalbestätigungen über den Empfang von Leistungen sind unzulässig. Im Leistungsnachweis sind alle erbrachten Leistungen nach Art, Menge, Datum und Uhrzeit aufzuschlüsseln.
5. Gesamtaufstellung der Abrechnung
   Der spezialisierte Palliativpflegedienst hat auf dem Abrechnungsbeleg mit seiner Unterschrift zu bestätigen, dass die in Rechnung gestellten Leistungen nach Art und Häufigkeit erbracht worden sind.

Rechnungen, die den Anforderungen nicht entsprechen, können unter Angabe des Grundes zur Berichtigung an den Leistungserbringer zurückgegeben werden. Offensichtliche Rechen- oder Schreibfehler sollten unbürokratisch abgeklärt werden. Rechnungsbeanstandungen muss die Krankenkasse innerhalb von sechs Monaten nach Rechnungseingang erheben. Einsprüche gegen Beanstandungen können vom Leistungserbringer innerhalb von drei Monaten nach Mitteilung geltend gemacht werden.

**1.**

Der Rahmenvertrag über die spezialisierte ambulante Palliativversorgung gemäß § 132d SGB V in Berlin vom 16.09.2013 wird mit Wirkung ab 01.01.2016 wie folgt geändert:

Die Vergütung für die spezialisierten Palliativpflegedienste gemäß Anlage 2b wird durch die Fassung vom 01.12.2015 mit Wirkung ab 01.01.2016 ersetzt. Die Anlage 2b vom 01.12.2015 ist Bestandteil dieser Änderungsvereinbarung.

**2.**

Im Übrigen bleibt der Rahmenvertrag unverändert.

**3.**

Diese Änderung tritt am 01.01.2016 in Kraft.

Berlin, Potsdam, Hoppegarten, den 16.12.2015

Kassenärztliche Vereinigung Berlin
Für den Vorstand

AOK Nordost - Die Gesundheitskasse

Home Care Berlin e. V.
Der Vorstand

Verband der Ersatzkassen e. V. (vdek)
Der Leiter der vdek-Landesvertretung
Berlin/Brandenburg

Berliner Aktionsbündnis
ambulante Palliativpflege e. V.
Der Vorstand

BKK Landesverband Mitte
Regionalvertretung Berlin und Brandenburg

BIG direkt gesund

IKK Brandenburg und Berlin

Knappschaft - Regionaldirektion Berlin

SVLFG
als Landwirtschaftliche Krankenkasse

Brandenburg

**Vereinbarung**
**gemäß § 132d Abs. 1 i. V. m. § 37b SGB V**

**über die**
**spezialisierte ambulante Palliativversorgung (SAPV)**
**von unheilbar erkrankten Menschen**
**im häuslichen Umfeld**

zwischen der

[redacted]

(nachfolgend PCT genannt)

und

[redacted]

(nachfolgend Krankenkassen genannt)

**Inhaltsverzeichnis:**


| | |
|---|---|
| | Präambel |
| § 1 | Ziel, Geltungsbereich und Gegenstand |
| § 2 | Grundsätze der Leistungserbringung |
| § 3 | Strukturanforderungen an das PCT |
| § 4 | Aufgaben des PCT |
| § 5 | Mitwirkung von Vertragsärzten im Rahmen der palliativmedizinischen Versorgung |
| § 6 | Aufgaben der mitwirkenden Vertragsärzte |
| § 7 | Qualitätssicherung |
| § 8 | Vergütung und Abrechnung |
| § 9 | Datenschutz |
| § 10 | Haftung |
| § 11 | Sonstige Bestimmungen |
| § 12 | Inkrafttreten/Kündigung |
| | Protokollnotizen |


**Anlagen:**


| | |
|---|---|
| Anlage 1 | Verordnung spezialisierter ambulanter Palliativversorgung (SAPV) |
| Anlage 2 | Mitwirkungserklärung des koordinierenden Vertragsarztes |
| Anlage 3 | Datenerhebungsbogen |
| Anlage 4 | Pflegekurzbericht |
| Anlage 5 | Basisdokumentation des PCT (Teil 1 und 2) |
| Anlage 6 | Vergütung des PCT sowie der nach § 5 mitwirkenden Vertragsärzte |
| Anlage 7 | Abrechnung gegenüber den Krankenkassen |

## Präambel

Unheilbar erkrankte Menschen wünschen oftmals nicht in der teilweise als anonym erlebten Umgebung eines Krankenhauses zu sterben, sondern in eigener häuslicher Umgebung. Das Sterben zu Hause stellt heute allerdings eher eine Ausnahme dar. Die Vertragspartner stimmen in der Einschätzung überein, dass die Voraussetzungen für ein menschenwürdiges Leben bis zum Tod in ihrer häuslichen Umgebung durch zielgerichtetes Zusammenwirken von Haus- und Fachärzten (nachfolgend Vertragsärzte genannt), die u. a. auch die allgemeine ambulante Palliativversorgung sicherstellen, Leistungserbringern der SAPV und ambulanten Hospizdiensten sowie psychoonkologischen Betreuungsdiensten verbessert werden können. Die individuellen Bedürfnisse und Wünsche dieser Menschen sowie die Belange ihrer oder vertrauten Personen stehen dabei im Mittelpunkt der Versorgung. Der Patientenwille, der auch durch Patientenverfügung zum Ausdruck kommen kann, ist zu beachten.

## § 1
## Ziel, Geltungsbereich und Gegenstand

(1) Ziel der Vereinbarung ist es, eine ambulante Versorgung unheilbar kranker Menschen in der letzten Phase ihres Lebens in häuslicher Umgebung zu sichern, ihre Lebensqualität unter Berücksichtigung des Krankheitsstadiums zu verbessern bzw. zu erhalten und ihnen ein menschenwürdiges Leben bis zum Tod in ihrer häuslichen Umgebung zu ermöglichen. Weitere Ziele sind die Strukturierung der Behandlungsabläufe und deren Anpassung an den besonderen Bedarf der betroffenen Versicherten im ambulanten Bereich.

(2) Gegenstand der Vereinbarung ist die spezialisierte ambulante Palliativversorgung gemäß der Richtlinie des Gemeinsamen Bundesausschusses zur Verordnung spezialisierter ambulanter Palliativversorgung (SAPV-RL) unheilbar kranker Menschen in der letzten Phase ihres Lebens. Die allgemeine ambulante palliativmedizinische Versorgung obliegt grundsätzlich den die Versicherten betreuenden Vertragsärzten im Rahmen der ambulanten Versorgung und ist nicht Gegenstand dieser Vereinbarung.

(3) Diese Vereinbarung gilt
- für das PCT sowie die im Rahmen dieser Vereinbarung kooperierenden Leistungserbringer und komplementären Einrichtungen und
- für Versicherte der AOK Brandenburg sowie der IKK Brandenburg und Berlin mit Wohnsitz im Versorgungsgebiet Potsdam (Stadt Brandenburg an der Havel, anliegende Gemeinden des Landkreises Potsdam-Mittelmark, Landkreis Havelland), die der SAPV gemäß der SAPV-RL bedürfen.

## § 2
## Grundsätze der Leistungserbringung

(1) Spezialisierte ambulante Palliativversorgung wird dem Versicherten als Sachleistung zur Verfügung gestellt und ist intermittierend oder durchgängig nach Bedarf als
- Beratungsleistung,
- Koordination der Versorgung,
- additiv unterstützende Teilversorgung,
- vollständige Versorgung

vom PCT zu erbringen, soweit die allgemeine Palliativversorgung und das bestehende ambulante Versorgungsangebot, insbesondere das der Vertragsärzte, Krankenhäuser und Pflegedienste, nicht ausreicht.

(2) Die Versorgung der Versicherten muss ausreichend und zweckmäßig sein, sie orientiert sich am individuellen Hilfebedarf des Versicherten, darf das Maß des Notwendigen nicht überschreiten und muss wirtschaftlich erbracht werden.

(3) Sofern Kinder und Jugendliche versorgt werden, sind deren Belange besonders zu berücksichtigen.

(4) a) Art, Umfang, Inhalt und Dauer der vom PCT zu erbringenden Leistungen ergeben sich aus der Verordnung der/des behandelnden Vertragsärztin/-arztes sowie ggf. der/des Krankenhausärztin/-arztes (Anlage 1). Die Verordnung sollte inhaltlich mit dem PCT abgestimmt werden. Bei Verordnung durch eine/einen Krankenhausärztin/-arztes ist die Dauer der Verordnung auf längstens sieben Tage begrenzt. Auf Grundlage der Verordnung wird das PCT bis zur Entscheidung der Krankenkasse über die Genehmigung tätig. Solange die Genehmigung als Grundlage der weiteren Leistungserbringung nicht vorliegt, erfolgt die Leistungserbringung im Rahmen der vorläufigen Kostenübernahme, es sei denn, die Voraussetzungen für eine vorläufige Kostenübernahme liegen nicht vor (Abs. 4). Rechtzeitig vor Ablauf der Erstverordnung ist ggf. eine notwendige Folgeverordnung einzureichen und zur Genehmigung der Krankenkasse vorzulegen.

b) Die ärztliche Verordnung bzw. Folgeverordnung gemäß Abs. 1 ist grundsätzlich der zuständigen Krankenkasse zur Prüfung und Genehmigung, ggf. zur Einschaltung des Medizinischen Dienstes, vorzulegen (für AOK Brandenburg-Versicherte dem zuständigen AOK-Pflege-Center; sofern der Antrag durch das PCT einer unzuständigen Geschäftsstelle zugeleitet wurde, gilt für den internen Postweg ein Tag). Der Folgeverordnung ist im Rahmen einer Teil- oder Vollversorgung des Versicherten gemäß Abs. 1 grundsätzlich der Pflegekurzbericht (Anlage 4) für den vorangegangenen Bewilligungszeitraum beizufügen.

c) Die Krankenkasse übernimmt vom ärztlich festgelegten Leistungsbeginn an bis zur Entscheidung über die Genehmigung die Kosten für die von der /dem Vertragsärztin/-arzt verordneten und vom PCT erbrachten Leistungen, wenn die vollständig ausgefüllte Verordnung

(insbesondere die verordnungsrelevanten Diagnosen als medizinische Begründung, der Behandlungs-, Betreuungs- und/oder Hilfebedarf des Versicherten, der Beginn und die Dauer der zu erbringenden Leistungen sowie das Ausstellungsdatum) am dritten der Ausstellung folgenden Arbeitstag der unter Buchstabe c) genannten Stelle der Krankenkasse vorliegt (vorläufige Kostenübernahme). Ggf. kann die Verordnung vorab per Telefax (Empfangsbereitschaft wird seitens der Krankenkassen jederzeit sichergestellt) vollständig übersandt werden (Original ist dann unverzüglich nachzureichen).

(5) Über die Leistungserbringung entscheidet die jeweils zuständige Krankenkasse grundsätzlich innerhalb einer Woche. Wird von der Krankenkasse eine Ablehnung der ärztlich verordneten Leistung für erforderlich gehalten oder genehmigt sie Leistungen nicht im vollen ärztlich verordneten Umfang, wird diese Entscheidung der/dem Versicherten schriftlich bekannt gegeben. Das PCT wird über die Entscheidung der Krankenkasse schriftlich informiert. Es hat sicher zu stellen, dass die ggf. an der Versorgung/Betreuung beteiligten Pflegedienste sowie der/die Vertragsarzt/-ärztin informiert werden.

(6) Eine vorläufige Kostenübernahme ist ausgeschlossen, wenn

- für das PCT offensichtlich erkennbar ist, dass die Verordnung keine Leistungen nach § 37b Abs. 1 und 2 SGB V enthält,
- Leistungen verordnet wurden, die nicht im Verzeichnis der Richtlinien nach § 92 Abs. 1 Satz 2 Nr. 14 SGB V in der jeweils gültigen Fassung enthalten sind.

(7) Im Falle der Besserung bzw. einer Stabilisierung im Krankheitsverlauf ist der Versorgungsumfang der SAPV so weit wie möglich zu reduzieren und eine Weiterversorgung im Rahmen der allgemeinen Palliativversorgung anzustreben.

(8) Jegliche Änderungen oder Ergänzungen der Verordnung im Rahmen der Anpassung des bereits genehmigten Versorgungsumfangs obliegen dem behandelnden Vertragsarzt in Abstimmung mit dem PCT und bedürfen einer erneuten Bestätigung mit Arztunterschrift, Stempel und Datum sowie der Genehmigung durch die Krankenkasse gemäß Abs. 4.

(9) Die Sicherstellung der reibungslosen Versorgung mit Arznei, Heil- und Hilfsmitteln obliegt den betreuenden Vertragsärzten im Rahmen der allgemeinen Palliativversorgung, so weit es sich nicht um eine Krisensituation handelt.

(10) Im Sinne der Wirtschaftlichkeit sind verordnete Leistungen der SAPV und der Häuslichen Krankenpflege sowie Leistungen der Pflegeversicherung, soweit möglich und sinnvoll, innerhalb eines Einsatzes zu erbringen.

(11) Durch Kooperation aller am Versorgungsprozess Beteiligten ist auf eine effiziente Leistungserbringung und eine vertrauensvolle Zusammenarbeit mit dem Versicherten und seinen Bezugspersonen hinzuwirken.

## § 3
## Strukturanforderungen an das PCT

(1) Grundlage für den Vertragsabschluss bildet ein Konzept, in dem der inhaltliche und organisatorische Rahmen der Leistungserbringung sowie die Einbindung in die regionale Versorgungsstruktur beschrieben sind. Das Konzept ist den Krankenkassen vor Vertragsabschluss einzureichen.

(2) Personelle Anforderungen

a) Das PCT – als Kernteam muss folgende Fachlichkeit nachweisen:

- Arzt/Ärzte mit der Zusatzweiterbildung Palliativmedizin nach der Weiterbildungsordnung der Landesärztekammer,
- Pflegefachkraft/-kräfte, die
  - die Erlaubnis zur Führung einer der Berufsbezeichnungen Gesundheits- und Krankenpflegerin/Gesundheits- und Krankenpfleger, Gesundheits- und Kinderkrankenpflegerin/Gesundheits- und Kinderkrankenpfleger, Altenpflegerin/Altenpfleger besitzen,
  - den Abschluss einer Palliative-Care-Weiterbildungsmaßnahme nach dem Curriculum der Deutschen Gesellschaft für Palliativmedizin (DGP) im Umfang von mindestens 160 Stunden nachweisen können,
  - eine mindestens zweijährige berufspraktische Tätigkeit innerhalb der letzten drei Jahre in der Palliativversorgung, davon mindestens sechsmonatige Mitarbeit in einer spezialisierten Einrichtung der Hospiz- und Palliativversorgung nachweisen können.

  **Protokollnotiz 1*
- Koordinatorin/Koordinator, die/der
  - die Erlaubnis zur Führung einer der Berufsbezeichnungen Gesundheits- und Krankenpflegerin/Gesundheits- und Krankenpfleger, Gesundheits- und Kinderkrankenpflegerin/Gesundheits- und Kinderkrankenpfleger, Altenpflegerin/Altenpfleger besitzen, Sozialarbeiterin/Sozialarbeiter besitzt,
  - den Abschluss einer Palliative-Care-Weiterbildungsmaßnahme nach dem Curriculum der DGP im Umfang von mindestens 160 Stunden nachweisen kann,
  - eine mindestens zweijährige berufspraktische Tätigkeit innerhalb der letzten drei Jahre in der Palliativversorgung, davon mindestens sechsmonatige Mitarbeit in einer spezialisierten Einrichtung der Hospiz- und Palliativversorgung nachweisen können.

(3) Zur Sicherstellung der SAPV im Versorgungsgebiet Potsdam sind durch das PCT verpflichtend weitere Leistungserbringer vertraglich einzubinden **Protokollnotiz 2*:

[redacted]

- Ärztinnen/Ärzte, die die Voraussetzungen gemäß § 3 Abs. 1 erfüllen **Protokollnotiz 3*
- zugelassene Pflegedienste mit Vertrag nach § 132 a SGB V zur Häuslichen Krankenpflege bzw. mit Versorgungsvertrag nach § 72 SGB XI zur ambulanten Pflege , die Pflegefachkräfte in einem sozialversicherungspflichtigen Beschäftigungsverhältnis angestellt haben, die die Voraussetzungen gemäß § 3 Abs. 1 erfüllen. **Protokollnotiz 4* Diese Pflegedienste haben zu garantieren, dass die Leistungen im Rahmen der SAPV ausschließlich durch diese qualifizierten Pflegefachkräfte erbracht werden.

Weitere vertraglich einzubindende Fachkräfte wie z. B. Sozialarbeiterinnen/Sozialarbeiter, Psychologinnen/Psychologen, Sozialpädagoginnen/Sozialpädagogen haben ebenfalls den Abschluss einer Palliative-Care-Weiterbildungsmaßnahme nach dem Curriculum der DGP für anderen Berufsgruppen im Umfang von mindestens 160 Stunden **Protokollnotiz 5* oder mehrjährige Erfahrung in der Palliativversorgung nachzuweisen.

Zur Sicherstellung der psychosozialen Unterstützung sind ambulante Hospizdienste gemäß § 39 a Abs. 2 SGB V im Versorgungsgebiet kooperativ einzubinden.

Die Nachweise gemäß Ziffer (2) und (3) sind bei den Krankenkassen in beglaubigter Form einzureichen.

(4) Sächliche Grundausstattung:

- Das PCT hat als Mindestanforderung an die sächliche Ausstattung Folgendes vorzuhalten bzw. sicher zu stellen:
  - eine geeignete, aktuell geführte und für die an der Versorgung Beteiligten jederzeit zugängliche Patientendokumentation
  - eine ausreichende und geeignete Mobilität zur zeitnahen häuslichen Versorgung der Patienten
  - Arzneimittel (inkl. Betäubungsmittelgesetz – BtM –) für die Notfall/Krisenintervention **Protokollnotiz 6*
  - Arzt-/Pflegekoffer/Bereitschaftstasche (Berücksichtigung der Kompatibilität der Verbrauchsmaterialien zu Medizinprodukten unterschiedlicher Hersteller, z. B. bei Portsystemen oder Infusionspumpen)
  - eine geeignete administrative Infrastruktur, z. B. Büro, Kommunikationstechnik
- Das PCT muss über eine eigenständige Adresse und geeignete Räumlichkeiten für
  - die Beratung von Patienten und Angehörigen,
  - Teamsitzungen und Besprechungen,
  - die Lagerhaltung von eigenen Medikamenten für Notfall-/Krisenintervention und Hilfsmitteln verfügen.
- Sofern eine Aufbewahrung von Medikamenten erfolgt, die unter das BtM fallen, ist ein BtM-Schrank erforderlich.

[redacted]

## § 4
## Aufgaben des PCT

(1) Das PCT wird auf Verordnung (Anlage 1) des behandelnden Vertragsarztes tätig. Es wird vorrangig beratend, koordinierend und ggf. mitbehandelnd in Fällen tätig, bei denen spezielle palliativmedizinische Kenntnisse für die Symptomkontrolle oder -intervention bzw. spezielle palliativpflegerische Kenntnisse erforderlich sind. Das PCT arbeitet eng mit den die betreffenden Versicherten im Rahmen der Regelversorgung betreuenden Vertragsärzten, den Pflegediensten und weiteren Kooperationspartnern zusammen. Nur im Ausnahmefall und phasenbezogen, insbesondere zur Vermeidung einer ansonsten gebotenen stationären Krankenhausbehandlung, sowie mit Zustimmung des Versicherten und in Abstimmung mit dem ihn behandelnden Vertragsarzt kann die Behandlung auf das PCT übergehen.

(2) Das PCT hat die zuständige Krankenkasse über neu aufgenommene Versicherte und deren betreuende Vertragsärzte, die im Rahmen der SAPV nach dieser Vereinbarung mitwirken, zu informieren.

(3) Das PCT erstellt für jeden zu versorgenden Versicherten einen individuellen Behandlungsplan, der mit den übrigen an der SAPV beteiligten Leistungserbringern abzustimmen ist.

(4) Die ständige Verfügbarkeit mindestens einer Palliativärztin/eines Palliativarztes und/oder einer Palliativpflegefachkraft gemäß den Anforderungen des § 3 Abs. 1 ist zu gewährleisten. Die Verfügbarkeit umfasst auch das umgehende Aufsuchen des Versicherten bei telefonischer Anforderung.

Durch den hohen Grad der Erreichbarkeit und fachlicher Kompetenz muss das PCT in der Region eine reibungslos funktionierende Schnittstelle zwischen stationärem und ambulantem Sektor darstellen.

(5) Die Koordinatorin/Der Koordinator des PCT übernimmt sowohl eine koordinierende als auch beratende Funktion. Sie/Er ist erste Ansprechpartnerin/erster Ansprechpartner in der Regelarbeitszeit und übernimmt die im Einzelfall erforderliche Koordination im Sinne des Case-Managements. Durch sie/ihn ist zu gewährleisten, dass zwischen den an der SAPV-Versorgung des Versicherten beteiligten Leistungserbringern zeitnah alle wichtigen Informationen über die vorhergehende Behandlung unter Berücksichtigung datenschutzrechtlicher Regelungen ausgetauscht werden.

Der Koordinatorin/Dem Koordinator des PCT obliegt das Monitoring der versichertenbezogenen Dokumentation durch die jeweiligen Leistungserbringer, die Organisation der psychosozialen Unterstützung und ggf. die Einschaltung weiterer Berufsgruppen wie Seelsorge, Sozialarbeit und Psycho(onko)logen im Sinne des System-Managements in Abstimmung mit dem Arzt/Ärztin des PCT.

(6) Die Dokumentation der versichertenbezogenen Leistungen durch die an der Versorgung beteiligten Leistungserbringer/Personen ist entsprechend der jeweiligen Berufsordnung sicherzustellen. Das PCT verfügt aufgrund eines einheitlichen Patientendokumentationssystems zu jeder Zeit über die notwendigen Informationen zum Versicherten.
Die Krankenkassen erhalten über den Medizinischen Dienst der Krankenkassen bei Bedarf Einsicht in die Patientendokumentation.

(7) Zu den Schwerpunktaufgaben des PCT gehören insbesondere:

- Beratung des mitwirkenden Vertragsarztes bei der
  - Einstellung der Schmerztherapie/Symptomkontrolle,
  - Verordnung von Medikamentenapplikationen über Pumpensysteme,
  - künstlichen Ernährung und Flüssigkeitsgabe in der letzten Lebensphase,
  - Beurteilung und Einleitung einer qualitativen adäquaten Wundbehandlung,
  - gezielten Einbeziehung des spezialisierten Palliativpflegedienstes.
- Koordination der spezialisierten palliativmedizinischen und palliativpflegerischen Versorgung unter Einbeziehung weiterer Berufsgruppen und von ambulanten Hospizdiensten im Rahmen einer multiprofessionellen Zusammenarbeit
- Symptomlinderung durch Anwendung von Medikamenten oder anderen Maßnahmen
- apparative palliativmedizinische Behandlungsmaßnahmen (z. B. Medikamentenpumpe)
- palliativmedizinische Maßnahmen, die nach ihrer Art, Schwere und Komplexität die Kompetenz erfordern, die der einer Ärztin oder eines Arztes mit Zusatzweiterbildung Palliativmedizin entspricht
- spezialisierte palliativpflegerische Leistungen, die nach ihrer Art, Schwere und Komplexität eine Kompetenz erfordern, die der einer Pflegefachkraft mit einer curricularen Weiterbildung zu Palliative-Care entspricht
- Führung eines individuellen Behandlungsplans, vorbeugendes Krisenmanagement, Bedarfsinterventionen
- Ruf-, Notfall- und Kriseninterventionsbereitschaft rund um die Uhr für die im Rahmen der SAPV betreuten Anspruchsberechtigten (einschließlich Hausbesuche) zur Sicherstellung der im Rahmen der SAPV erforderlichen Maßnahmen
- Beratung, Anleitung und Begleitung der Anspruchsberechtigten und ihrer Angehörigen zur palliativen Versorgung einschließlich Unterstützung beim Umgang mit Sterben und Tod
- spezialisierte Beratung weiterer betreuender Leistungserbringer der Primärversorgung
- psychosoziale Unterstützung im Umgang mit schweren Erkrankungen in enger Zusammenarbeit z. B. mit Seelsorge, Sozialarbeit und ambulanten Hospizdiensten
- Organisation regelmäßiger Fallbesprechungen
- Dokumentierung und Evaluierung der wesentlichen Maßnahmen im Rahmen der SAPV

(8) Die im PCT mitwirkenden Ärzte und Pflegefachkräfte nehmen an palliativmedizinischen bzw. palliativpflegerischen Fortbildungen in einem Umfang von 20 Zeitstunden (entsprechend 20 Punkten im Rahmen der ärztlichen Fortbildung) innerhalb von zwei Kalenderjahren teil.

(9) Das PCT verpflichtet sich, während der Laufzeit dieser Vereinbarung abgestimmte statistische Daten zu erfassen und den vertragsschließenden Krankenkassen zur Verfügung zu stellen (Anlage 5).

## § 5
## Mitwirkung von Vertragsärzten im Rahmen der SAPV

(1) Die Mitwirkung an dieser Vereinbarung ist für Vertragsärzte freiwillig.

(2) Die fallbezogene Mitwirkung an dieser Vereinbarung erklären Vertragsärzte schriftlich gegenüber dem PCT (Anlage 2).

(3) Die Pflichten im Rahmen ihrer vertragsärztlichen Tätigkeit bleiben unberührt.

## § 6
## Aufgaben der mitwirkenden Vertragsärzte

(1) Dem betreuenden Vertragsarzt obliegt die Koordination diagnostischer, therapeutischer und pflegerischer Maßnahmen der gemäß § 1 betroffenen Versicherten im Rahmen der vertragsärztlichen Versorgung, insbesondere im Rahmen der allgemeinen palliativmedizinischen Versorgung.

(2) Der mitwirkende Vertragsarzt hat bei Vorliegen der medizinischen Voraussetzungen des betroffenen Versicherten entsprechend der Richtlinie zur SAPV die Überweisung des betroffenen Versicherten zur palliativen Mitbetreuung an das PCT gemäß Verordnung vorzunehmen.

(3) Darüber hinaus hat der mitwirkende Vertragsarzt im Rahmen dieser Vereinbarung folgende zusätzliche Aufgaben zu übernehmen:

- Betreuung sowie Beratung des Versicherten sowie dessen Angehörigen zum Angebot des PCT
- grundsätzlich telefonische Rückkopplung mit einem Palliativarzt des PCT vor einer stationären Einweisung aufgrund einer palliativmedizinischen Krisensituation
- Anforderung und Teilnahme an Konsilien mit dem PCT auch im Rahmen von Hausbesuchen
- Information des PCT über den Todestag des Versicherten

## § 7

## Qualitätssicherung

(1) Das PCT ist verpflichtet, ein internes Qualitätsmanagement durchzuführen.

(2) Das PCT bildet einen multidisziplinären Qualitätszirkel und tauscht sich regelmäßig mit den Vertragsärzten sowie allen übrigen in der SAPV Tätigen über Erfahrungen und Optimierungsmöglichkeiten aus. Es stellt sicher, dass die Vertragsärzte und alle in der SAPV Tätigen über die Termine des multidisziplinären Qualitätszirkel informiert werden und ermöglichen eine Teilnahme. Der multidisziplinäre Qualitätszirkel tritt mindestens halbjährlich zusammen.

Inhalt und Ziel der multidisziplinären Qualitätszirkel sind insbesondere die qualifizierte Umsetzung von Behandlungsleitlinien bezogen auf die relevanten Erkrankungsbilder von Palliativpatienten mit Beschreibung der Schnittstellen und der Aufgabenverteilung der nach dieser Vereinbarung beteiligten Leistungserbringer u. a. für folgende Schwerpunkte:

- Schnittstellen/Anlässe zur Einbeziehung der Palliativärzte
- Schnittstellen/Anlässe zur Einbeziehung von spezialisierten Palliativpflegediensten mit Weiterbildung in Palliative-Care
- systematischer und abgestufter Einsatz von Arznei-, Heil- und Hilfsmitteln
- Kriterien für die Notwendigkeit der Einweisung von Palliativpatienten in ein Krankenhaus

Ferner ist ein geeignetes Verfahren zur Schmerzmessung und der daraus abzuleitenden medikamentösen Maßnahmen für eine sachgerechte Schmerztherapie abzustimmen. Die in den Qualitätszirkeln entwickelten Umsetzungsempfehlungen werden den Krankenkassen in geeigneter Weise zur Kenntnis gegeben.

(3) Das PCT hat sich außerdem an Maßnahmen der externen Qualitätssicherung zu beteiligen.

(4) Ein geeignetes Dokumentationssystem ist sachgerecht und kontinuierlich zu führen und auf Wunsch der Krankenkasse als Konzept vorzulegen. Das Dokumentationssystem muss versichertenbezogene Daten und – soweit vorhanden – allgemein anerkannte Indikatoren für eine externe Qualitätssicherung enthalten und eine bundesweite Evaluation ermöglichen. Es soll kompatibel zu den bestehenden Dokumentationssystemen der an der Versorgung beteiligten Leistungserbringer sein.

(5) Die Teilnahme an Supervision ist zu ermöglichen. Regelmäßige multiprofessionelle Fallbesprechungen sind in überschaubaren Intervallen durchzuführen; die inhaltliche und zeitliche Organisation ist den Beteiligten zu überlassen.

## § 8
## Vergütung und Abrechnung

(1) Die Vertragspartner vereinbaren eine pauschalierte Vergütungsregelung. Näheres dazu wird in Anlage 6 bestimmt.

(2) Für den besonderen Aufwand, der mit der Durchführung dieser Vereinbarung für die gemäß § 4 mitwirkenden Vertragsärzte entsteht, erhalten diese eine Vergütung vom PCT gemäß Anlage 6. Bei der Berechnung der Pauschale für gemeinsam durchgeführte Konsilien mit dem PCT im Rahmen von Hausbesuchen zur Vermeidung von ansonsten gebotener stationärer Krankenhausbehandlung (Anlage 6: PCT 05, HF 02) ist die Begründung für den Hausbesuch prüfbar zu dokumentieren.

(3) Das PCT stellt den Krankenkassen monatlich versichertenbezogen die Leistungsvergütungen gemäß Anlage 6 unter Angabe der Namen der mitbetreuenden Ärzte und deren Leistungsumfang in Rechnung.

(4) Die Krankenkassen vergüten die Leistungen nach Prüfung innerhalb von vier Wochen nach Rechnungseingang.

## § 9
## Datenschutz

Für die Erhebung, Verarbeitung und Nutzung von personenbezogenen Daten gelten die Regelungen über die Einhaltung der ärztlichen Schweigepflicht nach der Berufsordnung und dem allgemeinen Strafrecht sowie für die verschiedenen Phasen der Verarbeitung personenbezogener Daten und der Datensicherheit gemäß SGB X in der jeweils gültigen Fassung.

## § 10
## Haftung

(1) Die Haftung richtet sich nach den allgemeinen gesetzlichen Bestimmungen.

(2) Aus dieser Vereinbarung können gegen die Krankenkassen keine haftungsrechtlichen Ansprüche hergeleitet werden. Für den Fall, dass eine Krankenkasse dennoch haftungsrechtlich in Anspruch genommen wird, stellen die für die Versorgungsaufgaben nach dieser Vereinbarung jeweils zuständigen Beteiligten für von ihnen zu vertretende haftungsbegründende Umstände dieser Krankenkasse im Innen- und Außenverhältnis von der Haftung frei.

## § 11
## Sonstige Bestimmungen

(1) Die Vertragspartner werden die Versorgungsabläufe nach dieser Vereinbarung konstruktiv beobachten und sind bereit, erforderliche Anpassungen aufgrund der Praxiserfahrungen bei neuen Vertragsverhandlungen vorzunehmen.

(2) Die Vertragspartner werden gemeinsam prüfen, inwieweit die Ziele nach § 1 Abs. 1 erreicht worden sind. Das PCT stellt hierzu die Dokumentationen nach Anlage 5 zur Verfügung.

(3) Für die mitwirkenden Vertragsärzte, die nachweislich aufgrund einer intensiveren Betreuung von Palliativ-Patienten die Toleranzgrenzen der Wirtschaftlichkeitsprüfung überschreiten, stellt die AOK und IKK Brandenburg und Berlin geeignete Verordnungsdaten zur Verfügung, um ihnen so den Nachweis der sachlichen Notwendigkeit zu erleichtern.

(4) Sollte eine Bestimmung dieser Vereinbarung unwirksam sein oder werden oder die Vereinbarung unvollständig sein, so wird die Vereinbarung in ihren übrigen Inhalten davon nicht berührt. Die unwirksame oder fehlende Bestimmung wird durch eine solche Bestimmung ersetzt, die dem Sinn und Zweck unwirksamer Bestimmungen in rechtswirksamer Weise und wirtschaftlich am nächsten kommt. Gleiches gilt für etwaige Vertragslücken.

## § 12
## Inkrafttreten/Kündigung

(1) Dieser Vertrag und die Vergütungsregelung (Anlage 6) treten zum 01.04.2009 in Kraft.

(2) Sie können ordentlich von jeder Vertragspartei mit einer Frist von sechs Monaten ganz oder teilweise und unabhängig voneinander gekündigt werden, der Vertrag jedoch nicht vor dem 31.03.2011 und die Vergütungsregelung (Anlage 6) nicht vor dem 31.03.2010.

(3) Das Recht zur außerordentlichen Kündigung dieser Vereinbarung aus wichtigem Grund bleibt unberührt.

(4) Wirken Rechtsänderungen auf die Inhalte dieses Vertrages, treten die Vertragsparteien unverzüglich in Verhandlungen, ohne dass es einer Kündigung des Vertrages bedarf.

[redacted] Brandenburg, den [redacted]

[redacted]

Protokollnotiz 1 zu § 3 Abs. 2 der Vereinbarung

Der Abschluss der Vereinbarung erfolgt mit der Maßgabe, dass folgende Voraussetzungen erfüllt werden:

Der Nachweis der Palliative-Care-Weiterbildungsmaßnahme von 160 Stunden für die [redacted] ist nach erfolgreichem Abschluss am [redacted] bei den Krankenkassen umgehend in beglaubigter Form einzureichen. Sofern eine viermonatige Tätigkeit in einer spezialisierten Einrichtung der Hospiz- und Palliativversorgung vo[redacted]bis Vertragsabschluss nachgewiesen werden kann, erfolgt eine Anerkennung der geforderten Berufserfahrung.

Protokollnotiz 2 zu § 3 Abs. 3 der Vereinbarung

Innerhalb von 15 Monaten nach Vertragsabschluss ist die vertragliche Sicherstellung der flächendeckenden Versorgung mit SAPV im Versorgungsgebiet gegenüber den vertragschließenden Krankenkasse nachzuweisen.

Protokollnotiz 3 zu § 3 Abs. 3 der Vereinbarung

Die Zusatzweiterbildung Palliativmedizin muss vor Vertragsabschluss begonnen worden sein. Diese Übergangsregelung verliert ihre Gültigkeit am [redacted]

Protokollnotiz 4 zu § 3 Abs. 3 der Vereinbarung

Sofern eine mindestens sechswöchige Tätigkeit in einer spezialisierten Einrichtung der Hospiz- und Palliativversorgung vor Vertragsabschluss nachgewiesen werden kann, erfolgt eine Anerkennung der geforderten Berufserfahrung der Pflegefachkraft.
Diese Übergangsregelung verliert ihre Gültigkeit am [redacted]

Protokollnotiz 5 zu § 3 Abs. 3 der Vereinbarung

Die Palliative-Care-Weiterbildungsmaßnahme nach dem Curriculum der DGP für anderen Berufsgruppen im Umfang von mindestens 160 Stunden ist innerhalb von zwei Jahren nach Vertragsabschluss nachzuweisen.

Protokollnotiz 6 zu § 3 Abs. 4 der Vereinbarung

Bis zum Inkrafttreten einer bundesweit einheitlichen Regelung zur Verordnungsfähigkeit von begleitenden Leistungen der SAPV sind die notwendigen Arzneimittel im Rahmen der palliativmedizinischen Teil- und Vollversorgung von Versicherten entsprechend der vertraglichen Vereinbarung direkt über eine Apotheke, vorzugsweise Krankenhausapotheke (§ 129a SGB V), zu beziehen und der jeweiligen Krankenkasse gesondert gemäß Anlage 7 Ziffer 7 der Vereinbarung in Rechnung zu stellen.

| Krankenkasse bzw. Kostenträger | | |
|---|---|---|
| Name, Vorname des Versicherten<br><br>geb. am | | |
| Kassen-Nr. | Versicherten-Nr. | Status |
| Betriebsstätten-Nr. | Arzt-Nr. | Datum |

# Verordnung spezialisierter ambulanter Palliativversorgung (SAPV)

**Per Fax an das PCT senden!**

☐ Vertragsarzt

☐ Erst-verordnung ☐ Folge-verordnung

vom ☐☐☐☐☐☐ bis ☐☐☐☐☐☐ ☐ Unfall Unfallfolgen

**Verordnungsrelevante Diagnose(n):** ______________________
ICD-10; ggf. Organmanifestationen)

Die Krankheit ist nicht heilbar, sie ist fortschreitend und weit fortgeschritten.

**Komplexes Symptomgeschehen:** ☐ Ausgeprägte Schmerzsymptomatik

☐ Ausgeprägte, urogenitale Symptomatik

☐ ausgeprägte respiratorische/kardiale Symptomatik

☐ ausgeprägte gastrointestinale Symptomatik

☐ ausgeprägte ulzerierende/ exulzerierende Wunden oder Tumore

☐ ausgeprägte neurologische / psychiatrische/psychische Symptomatik

☐ Sonstiges komplexes Symptomgeschehe[illegible]

**Nähere Beschreibung** des komplexen Symptomgeschehens und des besonderen Versorgungsbedarfs zur Begründung, warum spezialisierte ambulante Palliativversorgung notwendig ist (z. B. therapierefraktäre Schmerzen, Ruhedyspnoe/Erstickungsanfälle, nicht beherrschbares Erbrechen/Durchfälle):

______________________

**Aktuelle Medikation (ggf. einschließlich BtM):**

______________________

**Folgende Maßnahmen sind notwendig:**

☐ Beratung
a. *des behandelnden Arztes* ☐
b. *der behandelnden Pflegefachkraft* ☐
c. *des Patienten/der Angehörigen* ☐

☐ Koordination der Palliativversorgung

mit folgender inhaltlicher Ausrichtung (Gegenstand, Häufigkeit, evtl. Beratung für Sonstige):

______________________

| **Additiv unterstützende Teilversorgung** | **Nähere Angaben zu den notwendigen Maßnahmen der SAPV:** | |
|---|---|---|
| ☐ | | |
| **Vollständige Versorgung**<br>☐ | | Arztstempel / Unterschrift des Arztes |

## Antrag des Versicherten

Die spezialisierte ambulante Palliativversorgung wird wie vom behandelnden Arzt verordnet beantragt:

Datum ______________ Unterschrift des Versicherten ______________

Datenschutzhinweis (§ 67a Abs. 3 SGB X): Damit wir Ihren Antrag auf SAPV-Leistungen bearbeiten können, ist Ihr Mitwirken nach § 60 SGB I erforderlich. Ihre Daten sind im vorliegenden Falle aufgrund § 37b SGB V i. V. m. § 92 Abs. 1 SGB V zu erheben.

## Angaben des Leistungserbringers für die SAPV

Die verordnete spezialisierte ambulante Palliativversorgung wird für den Zeitraum vom/bis ______________ erbracht.

Name des Leistungserbringers / ggf. Stempel ______________

Anschrift (Straße, Haus-Nr., PLZ, Wohnort)/ ggf. Stempel ______________

Institutionskennzeichen des Leistungserbringers ______________ Ansprechpartner (Name) ______________

Vorwahl Telefon-Nr. / ./ Fax- Nr. ______________ E-Mail ______________

Datum ______________ Unterschrift des Leistungserbringers ______________

## Genehmigung der Krankenkasse

Die spezialisierte ambulante Palliativversorgung wird nach den vereinbarten Sätzen

- ☐ entsprechend der Verordnung übernommen
- ☐ In folgendem Umfang übernommen

______________

______________

______________

Die Genehmigung der Krankenkasse ist der Abrechnung beizufügen. Endet die Notwendigkeit vor Ablauf des angegebenen Zeitraums, so erlischt damit auch die Kostenverpflichtung.

Sollte die SAPV über den letzten Bewilligungstag hinaus erforderlich sein, so ist **rechtzeitig vor Ablauf** des genehmigten Zeitraums eine erneute Verordnung bei der Krankenkasse einzureichen.

Auszufüllen, soweit von Seiten der Krankenkasse Bedarf besteht:

Name, Vorname des Versicherten ______________ Straße, Hausnummer, PLZ, Wohnort ______________

Versicherten-Nr. ______________ geb. am ______________

| Datum | Unterschrift | Stempel der Krankenkasse |
|---|---|---|
| | | |

**Anlage 2**

zur Vereinbarung über die spezialisierte ambulante Palliativversorgung von unheilbar erkrankten Menschen im häuslichen Umfeld

## Mitwirkungserklärung des betreuenden Haus-/Facharztes

## an das Palliativ-Care-Team

Stempel des Arztes:

Bankverbindung des Arztes:

Ich bin über die Inhalte und den Verlauf der Vereinbarung über die spezialisierte ambulante Palliativversorgung von unheilbar erkrankten Versicherten im häuslichen Umfeld umfassend informiert worden. Ich bin mit den Inhalten der o. a. Vereinbarung einverstanden und bereit, die mich betreffenden Versorgungsaufgaben nach § 6 der Vereinbarung zu übernehmen. Im Zusammenhang mit der Verordnung von Leistungen der SAPV gemäß Anlage 1 der Vereinbarung (Verordnungsvordruck) stimme ich mich versichertenbezogen mit dem PCT ab. Insbesondere beachte ich die in § 9 der Vereinbarung normierten datenschutzrechtlichen Regelungen.

Hiermit erkläre ich meine Teilnahme an der o. a. Vereinbarung.

| | |
|---|---|
| ______________________ | ______________________ |
| Ort, Datum | Unterschrift des Arztes |

Anlage 3

Vereinbarung über die spezialisierte ambulante Palliativversorgung von unheilbar erkrankten Menschen im häuslichen Umfeld

[ ] Neuantrag
[ ] Änderung zum ________________

IK.: ________________
(Bitte immer angeben!)

# Datenerhebungsbogen

## zum Abschluss/zur Änderung eines Vertrages gemäß § 132d Absatz 1 SGB V

Der **Träger** (Name) ________________

Geschäftsführer/in / Inhaber/in ________________

Straße, Hausnummer ________________

Postleitzahl, Ort ________________ Tel. ________________

erklärt für das **Palliativ-Care-Team (PCT)**

Name ________________

Straße, Hausnummer ________________

Postleitzahl, Ort ________________

Telefon: ________________ Fax: ________________

die **Personalstruktur des PCT** wie folgt:

| Personal gem. § 3 des Vertrages | a) Name, Vorname<br>b) Niederlassung in .../ angestellt im Krankenhaus | a) berufl. Qualifikation<br>b) Palliative-Care-Ausbildung (160 h) ja/nein | Berufserfahrung gem. § 3 des Vertrages<br>ja/nein | tätig für das PCT seit |
|---|---|---|---|---|
| Koordinator/-in | | a)<br>b) | | |
| Palliativarzt/-ärztin | a)<br>b) | a)<br>b) | | |
| weitere:<br>Palliativarzt/-ärztin | a)<br>b) | a)<br>b) | | |
| Palliativarzt/-ärztin | a)<br>b) | a)<br>b) | | |
| Palliativarzt/-ärztin | a)<br>b) | a)<br>b) | | |
| Palliativarzt/-ärztin | a)<br>b) | a)<br>b) | | |

[redacted]

Anlage 3

Vereinbarung über die spezialisierte ambulante Palliativversorgung von unheilbar erkrankten Menschen im häuslichen Umfeld

| Personal gem. § 3 des Vertrages | a) Name des zugelassenen Pflegedienstes in<br>b) Name, Vorname | a) berufl. Qualifikation<br>b) Palliative-Care-Ausbildung (160 h) ja/nein | Berufserfahrung gem. § 3 des Vertrages<br>ja/nein | tätig für das PCT seit |
|---|---|---|---|---|
| Pflegefachkraft | a)<br>b) | a)<br>b) | | |
| Pflegefachkraft | a)<br>b) | a)<br>b) | | |
| Pflegefachkraft | a)<br>b) | a)<br>b) | | |
| Pflegefachkraft | a)<br>b) | a)<br>b) | | |

| weitere Fachkräfte gem. § 3 Abs. 2 Buchstabe c) des Vertrages: | Name, Vorname | Palliative-Care-Ausbildung (160 h)<br>ja/nein | Berufserfahrung gem. § 3 des Vertrages<br>ja/nein | tätig für das PCT seit |
|---|---|---|---|---|
| Sozialarbeiter/-in | | | | |
| Psychologe/ Psychologin | | | | |
| Sozialpädagoge/ Sozialpädagogin | | | | |
| anderer Berufe: | | | | |

**Kooperierender ambulanter Hospizdienst:**

Name ____________________

Straße, Hausnummer ____________________

Postleitzahl, Ort ____________________

Ort, Datum

Unterschrift Koordinator/in des PCT

Stempel/Unterschrift des Trägers des PCT

[redacted]

**Anlage 4**

zur Vereinbarung über die spezialisierte ambulante Palliativversorgung von unheilbar erkrankten Menschen im häuslichen Umfeld

# Pflegekurzbericht

Krankenversichertennummer: _______________ Krankenkasse: __________________

Name, Vorname: _________________________ Geburtsdatum: __________________

Leistungsbeginn: ________________________________________________________

Pflegeziel: _____________________________________________________________

_________________________________________________________________________

Warum wurde das Pflegeziel bisher nicht erreicht? / Pflegeprobleme

_________________________________________________________________________

_________________________________________________________________________

_________________________________________________________________________

_________________________________________________________________________

Geplante Pflegemaßnahmen/Durchführungshinweise/Ressourcen:

_________________________________________________________________________

_________________________________________________________________________

_________________________________________________________________________

_________________________________________________________________________

| | |
|---|---|
| ______________________________ | ______________________________ |
| Ort, Datum | Stempel des Pflegedienstes,<br>Unterschrift der verantwortlichen<br>Pflegefachkraft |

[redacted]

Anlage 5 (Teil 1)

zur Vereinbarung über die spezialisierte ambulante Palliativversorgung von unheilbar erkrankten Menschen im häuslichen Umfeld

# Basisdokumentation des Palliative-Care-Teams

Datum: ............. Uhrzeit: ...............

(Name/Institution): ..........................................................................................................

**Anfrage durch:**

..........................................................................................................................................

Name/Tel./Funktion (Notarzt/Hausarzt/Fachpflegedienst)

**Versicherter:**

Name, Vorname ............................................... KV-Nummer /Geburtsdatum:........................

**Grunderkrankung:**

❑ Tumorerkrankung ❑ Nicht-Tumorerkrankung

**Allgemeinbefinden:**

❑ sehr schlecht ❑ schlecht ❑ mittel ❑ gut ❑ sehr gut

**Patientenverfügung vorhanden:** ❑ unbekannt ❑ nein
❑ ja (Ausstellungsdatum) ..........................................

**Aktuelle Probleme (Hauptgrund der Anfrage bitte ankreuzen!)**

| | Hauptgrund | nein | leicht | mittel | stark |
|---|---|---|---|---|---|
| durchschnittlicher Schmerz | | | | | |
| maximaler Schmerz | | | | | |
| Übelkeit | | | | | |
| Erbrechen | | | | | |
| Obstipation / Diarrhoe | | | | | |
| Atemnot | | | | | |
| Schwäche | | | | | |
| Angst | | | | | |
| Hautprobleme | | | | | |
| | | | | | |
| | | | | | |

**Relevante Begleiterkrankungen / erkrankte Organe:**

❑ Herz/Kreislauf ❑ neurologisch ❑ psychiatrisch
❑ Lunge ❑ Leber ❑ Niere
❑ Sonstige ...................................

**bereits erfolgte Versorgungen:**

❑ PEG-Sonde ❑ Port ❑ ZVK ❑ Pumpe ❑ Dauerkatheter

[redacted]

## Anlage 5 (Teil 1)

zur Vereinbarung über die spezialisierte ambulante Palliativversorgung von unheilbar erkrankten Menschen im häuslichen Umfeld

### Vorbehandlung erfolgt wegen:

| | nein | ja | bei Bedarf |
|---|---|---|---|
| Schmerz | | | |
| Übelkeit | | | |
| Erbrechen | | | |
| Obstipation / Diarrhoe | | | |
| Atemnot | | | |
| Schwäche | | | |
| Angst | | | |
| Hautprobleme | | | |
| | | | |

**Soziale Situation:**

- ☐ unbekannt
- ☐ allein lebend
- ☐ mit Partner/Familie lebend
- ☐ keine Unterstützung durch Partner/Familie möglich
- ☐ Pflegedienst vorhanden

Therapieempfehlung
s. angefügten Therapieplan

Anlage 5 (Teil 2)

zur Vereinbarung über die spezialisierte ambulante Palliativversorgung von unheilbar erkrankten Menschen im häuslichen Umfeld

## Verlaufsbogen Nr.

**Name, Vorname des Versicherten:** ____________________ **KV-Nummer / Geburtsdatum:** ____________________

| | Ausgangs-Medikation | Woche 1 | Woche 2 | Woche 3 | Woche 4 | Woche 5 |
|---|---|---|---|---|---|---|
| Schmerz | | | | | | |
| Übelkeit/ Erbrechen | | | | | | |
| Atemnot | | | | | | |
| Angst | | | | | | |
| Obstipation/ Diarrhoe | | | | | | |

[redacted]

# Vergütungsregelung gemäß § 132d SGB V
# über die
# spezialisierte ambulante Palliativversorgung (SAPV)
# gemäß § 37b SGB V

Die spezialisierten Leistungen sind vom Vertragsarzt bzw. entsprechend qualifizierten Krankenhausarzt zu verordnen und von der Krankenkasse zu genehmigen. Es werden grundsätzlich nur die nachfolgenden Leistungen von der Krankenkasse finanziert, die Gegenstand dieser Vergütungsvereinbarung sind.

Das Palliative-Care-Team (PCT) hat eine fachlich kompetente und bedarfsgerechte spezialisierte ambulante Palliativversorgung nach den allgemein anerkannten medizinischen und pflegefachlichen Erkenntnissen zu wirtschaftlich vertretbaren Bedingungen sicher zu stellen. Im Mittelpunkt der Betrachtung steht der Versicherte/Betreute mit seinem individuellen Hilfebedarf. Die notwendigen Hilfen sind koordiniert sicherzustellen, so dass ein nahtloses und störungsfreies Ineinandergreifen – auch im Sinne der Bezugspflege – gewährleistet ist. Unter diesen Gesichtspunkten ist eine zeitliche Koordination aller medizinischen und pflegerischen Leistungen sowie Betreuungsleistungen vorzunehmen.

Die Erbringung der SAPV ist unter Berücksichtigung des behandlungs- und pflegeunterstützenden Umfeldes des Versicherten darauf auszurichten, dem sterbenden Menschen ein möglichst würdevolles und selbstbestimmtes Leben bis zum Ende zu ermöglichen, wobei Wünsche und Bedürfnisse der Sterbenden und ihrer Angehörigen im Zentrum des Handelns stehen. Ziel ist es, dass der Versicherte in der eigenen Häuslichkeit verbleiben kann, soweit dies seinem eigenen Wunsch entspricht und medizinisch vertretbar ist.

Alle Leistungen beinhalten

- die Wahrnehmung und Beobachtung,
- die Kommunikation,
- die erforderliche Information der am Versorgungsprozess Beteiligten,
- Phasen der Vor- und Nachbereitung der Versorgungsleistungen im Einzelfall,
- bei Erbringen spezialisierter ambulanter Palliativpflege das Bereitstellen der notwendigen Arbeitsmaterialien sowie deren Entsorgung und ggf. Säuberung des Pflegebereichs nach Verunreinigungen,
- den Behandlungsplan und die Dokumentation.

Die Leistungen und die hierfür vereinbarten Entgelte im Sinne des Vertrages sind auf Basis des durchschnittlich notwendigen Aufwands bei der

- Einstellung der Schmerztherapie/Symptomkontrolle,
- Verordnung von Medikamentenapplikationen über Pumpensysteme,
- künstlichen Ernährung und Flüssigkeitsgabe in der letzten Lebensphase,
- Beurteilung und Einleitung einer qualitativen adäquaten Wundbehandlung,
- gezielten Einbeziehung des spezialisierten Palliativpflegedienstes.

bewertet. Der Leistungsaufwand kann in Abhängigkeit von der individuellen Versorgungssituation des Versicherten unterschiedlich sein. Er ist jedoch mit der pauschalen durchschnittlichen Bewertung abgedeckt.

## Vergütung des PCT

1. Grund- und Beratungspauschale bei Überweisung an das PCT

| | | |
|---|---|---|
| je Versicherten*1 | PCT 01 | 100,00 EUR |

2. Pauschale für die Koordination der im Einzelfall erforderlichen diagnostischen, therapeutischen und pflegerischen Teilleistungen unter Einbeziehung weiterer Berufsgruppen und von Hospizdiensten

| | | |
|---|---|---|
| je Versicherten*1 | PCT 02 | 300,00 EUR |

*1 Die Leistungen nach Ziffer 1 und 2 können je Versicherten im Verlauf der SAPV jeweils einmal abgerechnet werden und sind auch neben den Leistungen nach Ziffer 3a, 3b und 4 abrechenbar.

3. a) Wochenpauschale*2 für die additiv unterstützende spezialisierte palliativmedizinische Teilversorgung

| | | |
|---|---|---|
| je Versicherten | PCT 03a | 120,00 EUR |

3. b) Wochenpauschale*2 für die additiv unterstützende spezialisierte palliativpflegerische Teilversorgung einschließlich aller Leistungen der häuslichen Krankenpflege gemäß § 37 SGB V

| | | |
|---|---|---|
| je Versicherten | PCT 03b | 190,00 EUR |

**Die Ziffern 3a und 3b sind nicht neben Ziffer 4 abrechenbar.**

4. Wochenpauschale*2 für die vollständige spezialisierte Palliativversorgung

| | | |
|---|---|---|
| je Versicherten | PCT 04 | 430,00 EUR |

davon:
- 240,00 Euro für die spezialisierte palliativmedizinische Versorgung
- 190,00 Euro für die spezialisierte palliativpflegerische Versorgung einschließlich aller Leistungen der häuslichen Krankenpflege gemäß § 37 SGB V

*2 Die Wochenpauschale bezieht sich auf einen Zeitraum von bis zu 7 Tagen und stellt nicht auf eine Kalenderwoche ab.

5. Pauschale für einen vom koordinierenden Vertragsarzt für einen von ihm betreuten Palliativpatienten im Ausnahmefall angeforderten Besuch bei hausärztlich-ambulant nicht beherrschbaren Symptomen zur Vermeidung stationärer Krankenhausbehandlung (insbesondere bei Schmerzkrisen trotz Einstellung nach WHO III, Atemnotanfällen, therapierefraktärem Erbrechen, Krampfanfall, Darmverschlusssymptomatik), einschließlich Begründung in der Dokumentation

| | | |
|---|---|---|
| je Versicherten | PCT 05 | 50,00 EUR |

## Vergütung der nach § 5 mitwirkenden Haus- und Fachärzte

Die nach § 5 mitwirkenden Haus- und Fachärzte erhalten für die nach dieser Vereinbarung zu erfüllenden Aufgaben folgende Pauschalvergütungen:

1. für die Anmeldung des Versicherten beim PCT gemäß Anlage 1, die Aktivierung des PCT mittels vollständig ausgefüllter Verordnung gemäß Anlage 2, die Abstimmung mit dem PCT bezüglich der Koordination und Betreuung eines Palliativ-Patienten sowie die fallbezogene Mitwirkung bei der Erstellung des Behandlungsplanes durch das PCT sowie die Mitwirkung im Qualitätszirkel gemäß dieser Vereinbarung

   je Quartal HF 01 50,00 EUR*

   * Vergütung nach Abschluss der Vereinbarung auf Basis des § 7 der SAPV-Richtlinie vom 20.12.2007 zur Vereinbarung eines Verordnungsvordrucks für die spezialisierte ambulante Palliativversorgung

   je Quartal HF 01 30,00 EUR*

2. für gemeinsam durchgeführte Konsilien mit dem PCT im Rahmen von Hausbesuchen zur Vermeidung einer ansonsten gebotenen stationären Krankenhausbehandlung HF 02 20,00 EUR

Anlage 7

zur Vereinbarung über die spezialisierte ambulante Palliativversorgung von unheilbar erkrankten Menschen im häuslichen Umfeld

## Abrechnung gegenüber der Krankenkasse

für den Zeitraum vom ____________________ bis ________________________

1. Versichertendaten:

   Name: ______________________ Vorname: ______________________ Vers. Nr.____________________

2. **Datum der Aufnahme in die SAPV / Datum des Erstkontakts:**
   (PCT 01, HF 01)

   ________________________________________________

3. **Daten der Behandlungstage, an denen abrechnungsfähige gemeinsame Hausbesuche von Hausarzt und PCT durchgeführt wurden** (PCT 05, HF 02):

| | | | | | | | | | | | | | | |
|---|---|---|---|---|---|---|---|---|---|---|---|---|---|---|
| Monat | | | | | | | | | | | | | | |
| Tag | | | | | | | | | | | | | | |

4. **Name und IK des mitbetreuenden Hausarztes:** ____________________________________________

   ____________________________________________

5. **Abgerechnete Pauschalen für den o. g. Versicherten:**

| Pauschale | PCT 01 | PCT 02 | PCT 03a | PCT 03b | PCT 04 | PCT 05 |
|---|---|---|---|---|---|---|
| Anzahl | 1 | 1 | | | | 1 |
| Summe | | | | | | |

| Pauschale | HF 01 | HF 02 |
|---|---|---|
| Anzahl | | |
| Summe | | |

Gesamtsumme: ___________________ Euro

6. **ggf. Sterbedatum:** _________________

7. **Für den o. g. Versicherten abgegebene Arzneimittel aus der Krankenhausapotheke*:**

| Name des Arzneimittels | Pharmazentralnummer PZN | Einzelpreis* in Euro |
|---|---|---|
| | | |
| | | |
| | | |
| | | |
| | | |

*Preise gemäß Vertrag nach § 129 a SGB V

Arzneimittelgesamtpreis im Monat: _______________ Euro

*Sofern die Verordnungsfähigkeit von Arzneimitteln durch die Palliativärzte des PCT geregelt wird, entfällt diese Abrechnungsziffer.

____________________________________ ____________________________________

Datum Stempel, Unterschrift PCT

[redacted]

Bremen

# Vertrag über die Erbringung Spezialisierter ambulanter Palliativversorgung (SAPV) gemäß §§ 132 d i. V. m. 37 b SGB V

**zwischen**

der AOK Bremen/Bremerhaven,

dem BKK-Landesverband Niedersachsen-Bremen,
zugleich für die Knappschaft,

der IKK-gesundplus Bremen,(ggf. IKK-Landesverband)
zugleich für die Krankenkasse für den Gartenbau,
handelnd als Landesverband für die landwirtschaftliche Krankenversicherung in Bremen

den Ersatzkassen:
Barmer Ersatzkasse
Techniker Krankenkasse (TK)
Deutsche Angestellten-Krankenkasse (Ersatzkasse)
KKH-Allianz (Ersatzkasse)
Gmünder ErsatzKasse - (GEK)
HEK - Hanseatische Krankenkasse
Hamburg Münchener Krankenkasse
hkk

gemeinsamer Bevollmächtigter mit Abschlussbefugnis:
Verband der Ersatzkassen e. V. (vdek),
vertreten durch den Leiter der Landesvertretung Bremen

**im Folgenden " Verbände" genannt**

**und**

***Träger/GbR gemeinsam handelnd als***
***vertreten durch den ...***
***Name/IK***

**im Folgenden „Leistungserbringer“ genannt**

## Präambel

Die spezialisierte ambulante Palliativversorgung (SAPV) dient dem Ziel, die Lebensqualität und die Selbstbestimmung schwerstkranker Menschen zu erhalten, zu fördern und zu verbessern und ihnen ein menschenwürdiges Leben bis zum Tod in ihrer häuslichen Umgebung oder in stationären Pflegeeinrichtungen, Einrichtungen der Kinder- und Jugendhilfe oder in Einrichtungen der Eingliederungshilfe zu ermöglichen. Versorgungsunsicherheiten, die zu unnötigen und die Versicherten stark belastenden Krankenhauseinweisungen führen, sollen durch die in der SAPV vorgehaltenen besonderen Fachkenntnisse vermieden werden. Symptome und Leiden sollen einzelfallgerecht und nach den Wünschen der Versicherten durch palliativmedizinisch-palliativpflegerische Maßnahmen gelindert oder behoben werden.

Die Versorgung des Versicherten/der Versicherten bedarf einer besonderen SAPV-Fachkompetenz. Sie muss darüber hinaus ausreichend und zweckmäßig sein, sie orientiert sich an dem individuellen Hilfebedarf des Versicherten, darf das Maß des Notwendigen nicht überschreiten und muss wirtschaftlich erbracht werden.

Der Versicherte/die Versicherte und möglichst auch sein/ihr soziales Umfeld (seine vertrauten Personen) sollen in die Versorgung mit einbezogen und über die Krankheit und den Krankheitsverlauf aufgeklärt werden.

Anspruch auf die Leistung der SAPV und damit einer besonderen (aufwändigen) Versorgung besteht, soweit die anderweitigen ambulanten ärztlichen und pflegerischen Versorgungsformen (z. B. häusliche Krankenpflege) sowie ggf. die Leistungen des ambulanten Hospizdienstes nicht oder nur unter besonderer Koordination ausreichen. Die SAPV ergänzt insoweit das bestehende Versorgungsangebot, insbesondere das der Vertragsärzte, Krankenhäuser und Pflegedienste einschließlich der Versorgungsangebote der allgemeinen ambulanten palliativmedizinischen Versorgung (AAPV). Sowohl im Rahmen einer Teilversorgung als auch bei einer der Vollversorgung ersetzt die SAPV die Regelversorgung weitgehend oder vollständig.

## § 1
## Vertragsziele und Vertragsgrundlagen

(1) Dieser Vertrag dient der Sicherstellung der bedarfsgerechten individuellen Versorgung des/der anspruchsberechtigten Versicherten mit SAPV durch den Leistungserbringer in dem im Versorgungskonzept ausgewiesenen Versorgungsgebiet.

(2) Bei der SAPV handelt es sich um eine interdisziplinäre Aufgabe, die nur dann bedarfsgerecht erbracht werden kann, wenn der Leistungserbringer Teil einer multiprofessionell vernetzten Versorgungsstruktur im regionalen Gesundheits- und Sozialsystem ist. Er arbeitet mit den an der Regelversorgung beteiligten Leistungserbringern unterschiedlicher Berufsgruppen (z. B. Haus-/Fachärzten, Pflegediensten, Seelsorgern, Sozialarbeitern, Physiotherapeuten, Psychologen und Ehrenamtlichen sowie den gemäß § 39 a Abs. 2 SGBV geförderten ambulanten Hospizdiensten in Form einer Netzwerkfunktion (vgl. § 3 Abs. 3) zusammen.

(3) Die palliativmedizinisch-palliativpflegerische Versorgung soll durch abgestimmte Behandlungs- und Kommunikationsstrukturen der Netzwerkpartner erfolgen.

(4) Grundlage dieses Vertrages sind:

- die „Richtlinie des Gemeinsamen Bundesausschusses zur Verordnung von spezialisierter ambulanter Palliativversorgung“ (Spezialisierte Ambulante Palliativversorgungs-Richtlinie/SAPV-RL) vom 20. Dezember 2007 (Anlage 1) und

- die „Gemeinsamen Empfehlungen der Spitzenverbände der Krankenkassen nach § 132 d Abs. 2 SGB V für die spezialisierte ambulante Palliativversorgung“ vom 23.06.2008 (Anlage 2)

in ihrer jeweils aktuellen Fassung.

## § 2
## Anspruchsvoraussetzungen

(1) Der/die Versicherte hat gemäß SAPV-RL Anspruch auf SAPV, wenn

1. er/sie an einer nicht heilbaren, fortschreitenden und bereits so weit fortgeschrittenen Erkrankung leidet, dass dadurch seine Lebenserwartung auf Tage, Wochen oder wenige Monate begrenzt ist und eine Krankenhausbehandlung im Sinne des § 39 SGB V nicht erforderlich ist.

2. er/sie unter Berücksichtigung der in der SAPV-RL genannten Ziele eine besonders aufwändige Versorgung (§ 4 SAPV-RL) benötigt, die nach den medizinischen und pflegerischen Erfordernissen auch in der häuslichen Umgebung (ambulant), in stationären Einrichtungen gemäß § 43 SGB XI i.V.m. § 72 SGB XI, in Einrichtungen der Kinder- und Jugendhilfe oder in Einrichtungen der Eingliederungshilfe erbracht werden kann.

(2) Liegt ein besonders aufwändiger Versorgungsbedarf nicht vor, besteht kein Anspruch auf SAPV.

(3) Die individuelle Versorgung des/der Versicherten mit SAPV wird nicht unterbrochen, sofern der/die Versicherte einzelne Maßnahmen der Versorgung ablehnt. Der Anspruch auf Versorgung mit SAPV bleibt hierdurch unberührt.

(4) Der/die Versicherte ist in der Wahl des Leistungserbringers frei.

## § 3
## Versorgungskonzept

(1) Der Leistungserbringer ist ein einheitlicher Leistungserbinger im Sinne der Konzeption eines Palliative-Care-Teams (PCT) und versorgt auf der Grundlage eines verbindlichen, strukturierten und schriftlich dargelegten Versorgungskonzeptes.

(2) Das Versorgungskonzept formuliert alle palliativmedizinisch-palliativpflegerischen Maßnahmen klar und eindeutig unter Beachtung von Maßnahmen zur Qualitätssicherung. Es muss darüber hinaus verdeutlichen, wie eine qualifizierte Leistungserbringung zu erfolgen hat, damit die in § 1 dieses Vertrages genannten Vertragsziele erreicht werden können.

(3) Die regionalen Netzwerkstrukturen sind zu beschreiben, in die sich der Leistungserbringer im Rahmen der Vernetzung integriert.

(4) Das Versorgungskonzept ist den Verbänden zur Verfügung zu stellen. Die Vertragsziele gemäß § 1 dieses Vertrages werden mit dem Versorgungskonzept umgesetzt und erfüllt. Der Leistungserbringer stellt die SAPV-Versorgung für sein Versorgungsgebiet sicher.

(5) Das Versorgungskonzept beinhaltet insbesondere folgende Aspekte:

1. Standort des Leistungserbringers,
2. Versorgungsgebiet,
3. Versorgungsprozess,
4. Kooperationspartner,
5. Kooperationsvereinbarungen mit im Rahmen des Netzwerkes (vgl. § 1 Abs. 3 und § 3 Abs. 3) mit an der Versorgung Beteiligten im Versorgungsgebiet (z. B. ambulante Hospizdienste, Seelsorge und Sozialarbeit) zur Sicherung der psychosozialen Unterstützung - Anlage 3,
6. Personelle Zuständig- und Verantwortlichkeiten im SAPV-Team (u. a. Weisungsbefugnisse, Weisungsgebundenheit, u. a.),
7. Qualifikationsnachweise der Personen gemäß § 10,
8. sächliche Ausstattung gemäß § 12,
9. Qualitätssicherungsmaßnahmen gemäß § 17,
10. Konzept zur Sicherstellung der reibungslosen Versorgung mit Arznei-, Heil- und Hilfsmitteln,
11. Konzept zur Sicherstellung der 24-Stunden-Ruf-und Einsatzbereitschaft,
12. Fallzahleinschätzung.

(6) Die Leistungserbringung ist durch den Leistungserbringer in Form geeigneter Maßnahmen unter Einsatz entsprechender personeller und sachlicher Mittel für die Dauer des Vertrages sicherzustellen. Die Verbände sind umgehend zu informieren, wenn die Funktionsfähigkeit des Leistungserbringers nicht mehr besteht.

(7) Der Leistungserbringer ist weisungsunabhängig.

(8) Der Leistungserbringer trägt die alleinige Verantwortung für das Versorgungskonzept.

## § 4
## Anzeigepflichten / Mitteilungspflichten

(1) Der Leistungserbringer ist verpflichtet, den Verbänden_anstehende Änderungen wesentlicher Umstände im Versorgungskonzept schriftlich innerhalb von 4 Wochen mitzuteilen. Dies sind insbesondere:

a. jede wesentliche Änderung der betrieblichen/organisatorischen Gegebenheiten.
b. Änderungen von Kooperationsvereinbarungen (Neuabschlüsse, Kündigung, inhaltliche Änderungen).
c. eingeleitete polizeiliche oder staatsanwaltliche Ermittlungsverfahren gegen den Leistungserbringer.

(2) Abweichend davon sind personelle Änderungen in Bezug auf die palliativmedizinische sowie pallitivpflegerische Qualifikation umgehend mitzuteilen.

(3) Die Verbände sind berechtigt, weitere Unterlagen zum Nachweis der organisatorischen und/oder personellen Voraussetzungen zur Einsichtnahme zu verlangen. Der Leistungserbringer ist verpflichtet, diese zur Verfügung zu stellen.

## § 5
## Verordnung / Genehmigung der SAPV

(1) Art, Umfang und Dauer der vom Leistungserbringer zu erbringenden Leistungen ergeben sich aus der von der Krankenkasse genehmigten Verordnung der behandelnden Vertragsärztin/des behandelnden Vertragsarztes oder der Krankenhausärztin/des Krankenhausarztes.

(2) Die Verordnung ist nicht übertragbar. Sie gilt nur für die Person, für die sie ausgestellt ist. Änderungen und Ergänzungen bedürfen der Unterschrift der/des verordnenden Vertragsärztin/Vertragsarztes.

(3) Leistungen der SAPV werden von der Krankenkasse nur genehmigt, wenn eine ordnungsgemäß ausgefüllte ärztliche Verordnung gemäß der zwischen den Spitzenverbänden der Krankenkassen und der Kassenärztlichen Bundesvereinigung geschlossenen Vereinbarung (Muster 63) vorliegt. Der/die Versicherte oder sein/ihr gesetzlicher Vertreter hat die Genehmigung der Verordnung schriftlich auf der Rückseite der Verordnung zu beantragen.

(4) Die Unterschrift durch den Leistungserbringer bzw. dessen Mitarbeiter ist ausgeschlossen. Kann die Unterschrift nicht erbracht werden, weil der Versicherte bzw. sein gesetzlicher Vertreter nicht in der Lage ist zu unterschreiben, bleibt das Unterschriftsfeld auf der Verordnung frei. Der Leistungserbringer gibt dort oder auf einer Anlage zur Verordnung die Gründe hierfür an.

(5) Die SAPV-RL ist für die Verordnung sowie für die Genehmigung durch die Krankenkasse maßgeblich.

(6) Der verordnende Arzt/die verordnende Ärztin legt nach aktuellem Versorgungsbedarf die Dauer der Verordnung fest. Die Krankenhausärztin/der Krankenhausarzt kann Leistungen in der Regel jedoch längsten für 7 Tage verordnen. Sollte eine Versorgung für einen längeren Zeitraum als 7 Tage erforderlich sein, ist rechtzeitig eine Folgeverordnung für den Zeitraum über den 7. Tag hinaus bei der zuständigen Krankenkasse einzureichen.

(7) Die Verordnung für einen zurückliegenden Zeitraum ist nicht zulässig.

(8) Der Leistungserbringer prüft im Rahmen des Eingangsassessments innerhalb von drei Arbeitstagen nach Erhalt der Verordnung die Notwendigkeit und Eignung der besonderen Mittel der SAPV. Das Eingangsassessment (Mindestangaben - siehe Muster Anlage 4) ist der zuständigen Krankenkasse mit dem Antrag auf Genehmigung der Verordnung vorzulegen. Sofern für eine bedarfsgerechte Versorgung die Mittel der Regelversorgung ausreichen, ist die die Krankenkasse hierüber innerhalb von drei Arbeitstagen zu informieren. Neben der Krankenkasse sind auch der/die Versicherte und der Erstverordner über die Ergebnisse des Assessments zu informieren.

(9) Kann die Durchführung der verordneten Leistung nicht mehr erfolgen, z. B. weil der/die Versicherte in ein Krankenhaus eingewiesen wurde, informiert der Leistungserbringer die zuständige Krankenkasse und den behandelnden Arzt innerhalb von drei Arbeitstagen. Gleiches gilt, wenn nach initialer Aufnahme in die SAPV, im Rahmen eines anlassbezogenen (z. B. Befundverbesserung) oder regelmäßig durchzuführenden Re-Assessments festgestellt wird, dass die besonderen Mittel der SAPV nicht mehr notwendig sind und die Mittel der Regelversorgung ausreichen.

## § 6
## Kostenzusage

(1) Die Krankenkasse übernimmt vom ärztlich festgelegten Leistungsbeginn an bis zu einer Entscheidung über die weitere Leistungserbringung die Kosten für die verordneten und vom Leistungserbringer erbrachten Leistungen entsprechend der Vergütung gemäß dieses Vertrages, wenn die ordnungsgemäß ausgefüllte Verordnung spätestens am dritten der Ausstellung folgenden Arbeitstag der Krankenkasse vorliegt. Sie informiert hierüber den Leistungserbringer.

(2) Soweit die verordneten Leistungen nicht oder nur zum Teil übernommen werden, informiert die Krankenkasse den/die Versicherte/n oder ggf. den gesetzlichen Vertreter, den Leistungserbringer und den verordnenden Arzt.

(3) Sofern die Verordnung verspätet eingereicht wird, werden die Kosten erst ab Eingangsdatum übernommen. Für die Fristwahrung ist ein Telefax (Vorder- und Rückseite der Verordnung) ausreichend. Die Originalverordnung ist unverzüglich vorzulegen.

(4) Liegt der Krankenkasse innerhalb von 7 Arbeitstagen ab Verordnungsbeginn zusätzlich zur Erstverordnung kein Ergebnis über das SAPV-Eingangsassessment des Palliativmediziners vor, endet die Kostenzusage mit Ablauf dieses Tages. Die Krankenkasse informiert den/die Versicherte/n oder ggf. den gesetzlichen Vertreter, den Leistungserbringer und den verordnenden Arzt.

## § 7
## Inhalt der Leistung

(1) Der Leistungserbringer erbringt die jeweils ärztlich verordnete SAPV-Leistung intermittierend oder durchgängig nach Bedarf als

1. **Beratungsleistung beinhaltet insbesondere:**

    a. Telefonat bzw. persönliches Gespräch mit Betroffenen (Versicherten oder deren Angehörige)

    b. Telefonat bzw. persönliches Gespräch mit einem/einer der Leistungserbringer der Primärversorgung

    c. Dokumentation

2. **Koordination der Versorgung beinhaltet insbesondere:**

    a. persönlicher/telefonischer Kontakt mit den an der Versorgung Beteiligten

    b. Ressourcenfokussierte Versorgungsplanung

    c. Assessment, Therapie und Notfallplanung, Vernetzung mit ambulanten und/oder stationären Leistungserbringern

    d. Für die verordnete Koordination der Versorgung besteht 24-Stunden-Rufbereitschaft in Absprache mit den Leistungserbringern der Primärversorgung. Die Rufbereitschaft kann nicht als alleinige Teilleistung verordnet werden.

    e. Dokumentation und Evaluation

3. **Additiv unterstützende Teilversorgung beinhaltet insbesondere:**

    a. Beratungs- und Koordinationsleistung

    b. Hausbesuche zu jeder Tages- und Nachtzeit

    c. Einzelne Leistungen der in § 5 Abs. 3 der SAPV-Richtlinie aufgeführten Leistungen

    d. Für die verordnete Teilversorgung besteht 24-Stunden-Rufbereitschaft und Einsatzbereitschaft in Absprache mit den Leistungserbringern der Primärversorgung. Die Rufbereitschaft kann nicht als alleinige Teilleistung verordnet werden.

    e. Dokumentation und Evaluation

4. **Vollständige Versorgung beinhaltet insbesondere:**

    a. Beratungs- und Koordinationsleistung

    b. Hausbesuche zu jeder Tages- und Nachtzeit

    c. Grundsätzlich alle Leistungen der in § 5 Abs. 3 der SAPV-Richtlinie aufgeführten Leistungen.

    d. Für die verordnete Vollversorgung besteht 24-Stunden-Rufbereitschaft und Einsatzbereitschaft in Absprache mit den Leistungserbringern der Primärversorgung. Die Rufbereitschaft kann nicht als alleinige Teilleistung verordnet werden.

    e. Dokumentation und Evaluation.

(2) Angehörige und dem/der Versicherten nahe stehende Personen sollen in die Pflege sowie in die psychosoziale Unterstützung und Sterbebegleitung mit einbezogen werden.

## § 8
## Zuzahlungen

(1) SAPV wird als Sachleistung zur Verfügung gestellt. Jegliche Zuzahlungen zu den Vertragsleistungen dürfen vom/von der Versicherten weder gefordert noch angenommen werden (nicht gemeint § 61 SGB V).

(2) Zuzahlungen vom/von der Versicherten dürfen auch in dem Fall nicht verlangt werden, wenn die Verordnung bei der Krankenkasse verspätet eingereicht wird, da Kosten erst ab Eingang der Verordnung bei der Krankenkasse gemäß § 6 dieses Vertrages übernommen werden.

(3) Zuwiderhandlungen stellen einen schweren Vertragsverstoß dar und berechtigen zur außerordentlichen Kündigung gemäß § 23 des Vertrages.

## § 9
## Vermittlungsverbot

(1) Die Annahme von Aufträgen zur Versorgung anspruchsberechtigter Personen dieses Vertrages und deren Weitergabe (Vermittlung an Dritte gegen Entgelt oder zur Erlangung geldwerter Vorteile) stellt einen schweren Vertragsverstoß (§ 23) dar.

(2) Vermittlung im Sinne dieser Bestimmung ist auch die Weitergabe von in eigenem Namen angenommenen Aufträgen zur Versorgung anspruchsberechtigter Personen dieses Vertrages an Dritte gegen Kostenerstattung.

## § 10
## Qualifikationsvoraussetzungen

(1) Die Leistungen der SAPV werden ausschließlich durch geeignete Personen erbracht, die nachfolgende Qualifikationsvoraussetzungen erfüllen. Der Leistungserbringer weist mindestens drei geeignete Ärztinnen/Ärzte sowie mindestens vier geeignete Pflegefachkräfte nach.

a. Ärztinnen und Ärzte nach Absatz 1 verfügen über:

- eine anerkannte Zusatzweiterbildung Palliativmedizin nach der aktuell gültigen Weiterbildung der jeweiligen Landesärztekammer (grds. 160 Std. Weiterbildung) und

- die Erfahrung aus der ambulanten palliativen Behandlung von mindestens 75 Palliativpatienten nachweisen können, z. B. in der häuslichen Umgebung oder in einem stationären Hospiz, innerhalb der letzten drei Jahre oder aus einer mindestens einjährigen klinischen palliativmedizinischen Tätigkeit in einer Palliativabteilung in einem Krankenhaus innerhalb der letzten drei Jahre.

b. Pflegefachkräfte nach Absatz 1 verfügen über:

- die Erlaubnis zur Führung einer der Berufsbezeichnungen Gesundheits- und Krankenpflegerin / Gesundheits- und Krankenpfleger, Gesundheits- und Kinderkranken-

pflegerin / Gesundheits- und Kinderkrankenpfleger oder Altenpflegerin / Altenpfleger entsprechend den gesetzlichen Bestimmungen in der jeweils gültigen Fassung und

- den Abschluss einer Palliative-Care-Weiterbildungsmaßnahme im Umfang von mindestens 160 Stunden vorweisen und

- die Erfahrung durch mindestens eine zweijährige praktische Tätigkeit als Pflegefachkraft in der Betreuung von Palliativpatienten in den letzten drei Jahren, davon mindestens sechsmonatige Mitarbeit in einer spezialisierten Einrichtung der Hospiz- und Palliativversorgung, nachweisen können.

c. Soweit weitere Fachkräfte (z. B. Sozialarbeiterinnen / Sozialarbeiter, Sozialpädagoginnen oder Sozialpädagogen, Psychologinnen oder Psychologen) vertraglich eingebunden werden, haben diese eine Zusatzweiterbildung Palliative Care für andere Berufsgruppen oder eine mehrjährige Erfahrung in der Palliativversorgung nachzuweisen.

(2) Die Personalstärke des Teams bestimmt sich durch die Anforderungen zur Erbringung ärztlicher und pflegerischer Leistungen, der 24-Stunden-Erreichbarkeit für betreute Versicherte, der qualifizierten Vertretung in Urlaubs- und Krankheitszeiten. Werden weitere Personen, als die in Absatz 1 genannten, eingestellt, sind für diese die Qualitätsanforderungen nach Absatz 1und Absatz 4 (Punkte 1-4) den Verbänden nachzuweisen.

(3) Wenn Kinder versorgt werden, müssen die Anforderungen an die ärztlichen und pflegerischen Personen und an die weiteren Fachkräfte den besonderen Belangen der Kinder gerecht werden.

(4) Der Leistungserbringer hat vor Vertragsabschluss zusätzlich zu den Voraussetzungen gemäß Abs.1 Folgendes unter Verwendung der Anlagen 5 und 6 vorzulegen bzw. nachzuweisen. Auf Anforderung der Verbände sowie unverzüglich bei Personalwechsel sind die Voraussetzungen erneut den Verbänden_vorzulegen bzw. nachzuweisen (mittels Anlage 6).

- amtlich beglaubigte Kopien der Auszüge vom Bundesamt für Justiz (nicht älter als drei Monate) für die Pflegefachkräfte und deren Vertretung sowie für die Ärzte des SAPV-Teams,

- aktuelle ärztliche Gesundheitszeugnisse der Pflegefachkräfte,

- amtlich beglaubigte Kopien der Qualifikationsnachweise der eingesetzten Pflegefachkräfte,

- Kopien der Arbeitsverträge mit den eingesetzten Pflegefachkräften aus denen mindestens Name, Geburtsdatum, Funktion sowie zeitlicher Umfang und Beginn der Tätigkeit ersichtlich sein müssen,

- Beitritt zur Berufsgenossenschaft für Gesundheitsdienst und Wohlfahrtspflege,

- Abschluss einer ausreichenden Betriebs- und Berufshaftpflichtversicherung für Personen- Sach- und Vermögensschäden, die jährlich an die Betriebsgröße (Durchschnittszahl der Mitarbeiter und Jahreslohn- und -gehaltssummen) angepasst wird,

- Handzeichenliste (Anlage 6)

## § 11
## Verordnung von Arznei-, Heil- und Hilfsmitteln im Rahmen der SAPV

(1) Für die Verordnung von Arznei-, Heil- und Hilfsmitteln durch Ärzte im SAPV-Team sind ausschließlich die im Rahmen der vertragsärztlichen Versorgung vereinbarten Muster 13, 14, 16 und 18 zu verwenden.

(2) Die Verordnungen sind auf der Basis der geltenden Richtlinien des G-BA auszuführen. Dabei ist das Wirtschaftlichkeitsgebot nach § 12 SGB V zu beachten.

(3) Für die im Rahmen der SAPV verordneten Arznei-, Heil- und Hilfsmittel gelten die nach §§ 300, 302 SGB V vereinbarten Abrechnungsverfahren entsprechend.

(4) Auf den Verordnungen sind die - von der KBV vergebene - SAPV - spezifische Betriebsstätten-Nummer sowie die Pseudo-Arztnummer 333333300 anzugeben. Diese Nummern dürfen nur für Verordnungen im Rahmen der SAPV auf der Basis einer gültigen SAPV-Verordnung verwendet werden.

(5) Bei der zeitgleichen Verordnung von Arzneimitteln einerseits und Hilfsmitteln andererseits sind jeweils getrennte Vordrucke nach Muster 16 zu verwenden.

(6) Damit eine schnelle Sicherstellung der Belieferung von verordneten Hilfsmitteln realisiert werden kann, sollte die leistungspflichtige Krankenkasse über die Notwendigkeit der Hilfsmittelverordnung sofort informiert werden. Die Belieferung von verordneten Hilfsmitteln erfolgt grundsätzlich im Rahmen des jeweiligen Hilfsmittelverfahrens der einzelnen Krankenkasse.

(7) Auch auf Betäubungsmittel-Rezepten sind die SAPV-Betriebsstätten-Nummer sowie die Pseudo-Arztnummer anzugeben. Solange das BtM-Muster noch nicht angepasst ist, ist die Betriebsstätten-Nummer in das Feld "Vertragsarztnummer" und die Pseudo-Arztnummer in das Feld "VK gültig bis" einzutragen.

(8) Der Leistungserbringer informiert die Verbände über die von der KBV vergebene Betriebsstätten-Nummer.

## § 12
## Sächliche Ausstattung

(1) Eine Versorgung mit Arznei- und Hilfsmitteln im Notfall (Notfallversorgung) ist durch geeignete Maßnahmen z. B. durch eine Kooperation mit einer Apotheke bzw. einem Sanitätshaus sicherzustellen. Von besonderer Bedeutung ist die Vorhaltung ausreichender und geeigneter Arzneimittel/BtM für die Versorgung im Not- und Kriseninterventionsfall im Arzt-/Pflegekoffer/Bereitschaftstasche.

(2) Die an die Aufbewahrung von BtM gestellten Voraussetzungen nach dem BtMG sind zu berücksichtigen.

(3) Der Leistungserbringer hat die Mindestanforderungen an die Ausstattung seiner Räumlichkeiten gemäß Ziffer 4.4 und 4.5 der Gemeinsamen Empfehlungen nach § 132 d Abs. 2 SGB V vom 23.06.2008 nachzuweisen (siehe Anlage 2).

(4) Die Verbände haben das Recht, jederzeit selbst und /oder durch Beauftragung des Medizinischen Dienstes der Krankenversicherung nach § 275 SGB V vor Ort die Erfüllung der Mindestanforderungen an die sächliche Ausstattung zu überprüfen.

## § 13
## Organisation

(1) Der Leistungserbringer verpflichtet sich zu einer 24-Stunden-Rufbereitschaft/Einsatzbereitschaft. Die ständige Verfügbarkeit eines qualifizierten Palliativarztes/der qualifizierten Palliativärztin und qualifizierter Pflegefachkräfte gemäß § 10 dieser Vereinbarung ist auch für notwendige Hausbesuche zu gewährleisten.

(2) Zur Gewährleistung eines zeitnahen und systematischen Informationstransfers bei Überleitung in eine stationäre Einrichtung (z. B. Krankenhaus, stationäre Pflegeeinrichtung) ist ein geeignetes Überleitungsmanagement sicherzustellen.

(3) Mit den Partnern (z. B. Sanitätshäuser, Apotheken), mit denen regelmäßig zusammengearbeitet wird, sind schriftliche Kooperationsvereinbarungen zu schließen. Um Struktur und Abläufe zu regeln und für alle Beteiligten transparent zu machen, ist ein Konzept gemäß § 3 dieses Vertrages vorzulegen.

(4) Es erfolgt eine enge Kooperation des Leistungserbringers mit dem behandelnden Haus- oder Facharzt hinsichtlich der Therapieplanung. Insbesondere erfolgt vor einer stationären Krankenhauseinweisung eine Abstimmung zwischen dem qualifizierten Palliativarzt/der qualifizierten Palliativärztin des Leistungserbringers und dem/der behandelnden Haus- oder Facharzt/- ärztin.

(5) Der Leistungserbringer stellt durch eine geeignete Infrastruktur sicher, dass die ständige Kommunikation innerhalb des Teams und zu allen an der Versorgung des jeweiligen Versicherten Beteiligten gewährleistet ist.

## § 14
## Patientendokumentation

(1) Der Leistungserbringer hat eine Patientendokumentation zu führen, aus der zu jeder Zeit die notwendigen Informationen über den Versicherten und die erbrachten Leistungen (Behandlungsverlauf) hervorgehen. Die Patientendokumentation wird regelmäßig von dem SAPV-Team aktualisiert.

(2) Das Dokumentationssystem hat den jeweils aktuellen Standards zu entsprechen und allgemein anerkannte Kriterien für eine externe Qualitätssicherung zu enthalten. Es muss eine bundesweite Evaluation ermöglichen. Einzelheiten der Dokumentation ergeben sich aus der Anlage 7 (Kerndatensatz) dieses Vertrages.

(3) Weiterhin hat der Leistungserbringer zu gewährleisten, dass die von ihm angewandte Dokumentation kompatibel zu bestehenden Dokumentationssystemen der an der Versorgung beteiligten Leistungserbringer ist.

(4) Die Dokumentation beinhaltet insbesondere:

a. ein Versichertenstammblatt (z. B. mit Angaben zum/zur Versicherten und Angehörigen, Hausarzt/-ärztin, Pflegedienst, Diagnosen, Absprachen, Behandlungsort, Medikation, Maßnahmen, u. w.),

b. ein Assessment mit folgenden Inhalten:

- Aufklärung des/der Versicherten / der Angehörigen,
- Therapieverzicht,

- Palliative Sedierung,
- Ethische Konflikte,
- Funktionale Selbstständigkeit,
- Familiäre Belastungssituation,
- Einschätzung Symptomkontrolle,
- Medikation,
- Bedarfsmedikation,
- Hilfsmittel,
- Physikalische und andere Therapien (z. B. Lymphdrainage),
- Beratungsbedarf

c. einen Maßnahmen-Plan / Durchführungsnachweis

d. Nachweise über die durchgeführten Maßnahmen in den Bereichen Beratung, Koordination, Pflege, Medizin, psychosoziale Unterstützung (Datum, Uhrzeit, Dauer)

e. einen Notfallplan / vorausschauender Behandlungsplan

f. Untersuchungsbefunde

g. das vertragsärztliche Verordnungsblatt für die Dokumentation der medizinischen Diagnostik und Therapie

h. eine Pflege- und Therapieplanung (Therapiebeginn, medizinische / pflegerische / psychosoziale Versorgungssituation, Therapie- und Pflegeziele,)

i. einen Bericht zur Beurteilung der medizinischen, pflegerischen und psychosozialen Maßnahmen.

(5) Jede an dem/der Versicherten bzw. für dessen/deren Angehörige erbrachte Leistung wird durch die nach § 10 behandelnde qualifizierte Person am Tag der Leistungserbringung dokumentiert und durch Handzeichen auf dem Leistungsnachweis gegengezeichnet. Der/die Versicherte, sein/ihr gesetzlicher Vertreter oder die Angehörigen und der qualifizierte Arzt/die qualifizierte Ärztin bestätigen ebenfalls nach erfolgter Leistungserbringung einmal monatlich durch Unterschrift und Datum die Richtigkeit und Vollständigkeit der dokumentierten Leistungen (Leistungsnachweis).

(6) Sämtliche zur Versorgung gehörende Dokumente verbleiben beim Versicherten.

(7) Die Dokumentation ist dem MDK und (- soweit der Versicherte eingewilligt hat (§ 67 b Abs. 1 SGB X -) im Einzelfall der Krankenkasse auf Anforderung zugänglich zu machen.

(8) Die Aufbewahrungsfrist für die Dokumentation beträgt 10 Jahre nach Ende des Kalenderjahres der Leistungserbringung.

## § 15
## Datenschutz, Schweigepflicht

(1) Der Leistungserbringer ist verpflichtet, die Bestimmungen über den Schutz der Sozialdaten (SGB X 2. Kapitel) zu beachten, insbesondere personenbezogene Daten nur zur Erfüllung der sich aus diesem Vertrag ergebenden Aufgaben zu verarbeiten, bekannt zu geben, zugänglich zu machen oder sonst zu nutzen.

(2) Der Leistungserbringer unterliegt hinsichtlich der Person des Versicherten und dessen Krankheiten der Schweigepflicht. Ausgenommen hiervon sind Angaben gegenüber der leistungspflichtigen Krankenkasse und/oder dem MDK, soweit sie zur Erfüllung der gesetzlichen Aufgaben der Krankenkasse erforderlich sind.

(3) Der Leistungserbringer hat die im Team tätigen Mitarbeiter zur Beachtung der Datenschutzbestimmungen und der Schweigepflicht zu verpflichten. Die Verpflichtung ist zu dokumentieren.

## § 16
## Vergütung

(1) Die palliativärztlichen und palliativpflegerischen bzw. kombinierten Leistungen im Rahmen der SAPV werden als Komplexpauschalen vergütet. Die Höhe der Vergütung richtet sich nach den in der Anlage 8 aufgeführten Pauschalen.

(2) Einsätze, die wegen fehlender Mitwirkung des Versicherten nicht erbracht werden können, sind nicht abrechnungsfähig.

(3) Mit den Komplexpauschalen nach Anlage 8 sind alle Aufwendungen abgegolten, die im Zusammenhang mit der SAPV-Leistung stehen. Eine zeitgleiche Abrechnung von Gebührenordnungspositionen des Einheitlichen Bewertungsmaßstabes (EBM) über die Kassenärztliche Vereinigung ist daneben ausgeschlossen.

(4) Die Vergütung erfolgt ausschließlich gegenüber dem Leistungserbringer.

## § 17
## Qualitätssicherungsmaßnahmen

(1) Der Leistungserbringer ist verpflichtet, ein einrichtungsinternes Qualitätsmanagement durchzuführen, das im Versorgungskonzept darzustellen ist. Er soll sich an externen Maßnahmen der Qualitätssicherung beteiligen.

(2) Zu den Qualitätssicherungsmaßnahmen gehören insbesondere:

1. interdisziplinarisch abgestimmter individueller Behandlungsplan,
2. regelmäßige interdisziplinäre Fallbesprechungen,
3. sachgerechte und kontinuierliche Führung eines geeignetes Dokumentationssystems,
4. Supervision,
5. halbjährliche multidisziplinäre Qualitätszirkel,
6. Fort- und Weiterbildungskonzept,
7. Abrechnungsstatistik,
8. Statistik über Sterbeorte (gemäß § 2 Abs. 1 Ziffer 2, Krankenhäuser).

(3) Die Verbände haben das Recht, jederzeit selbst und/oder durch Beauftragung des Medizinischen Dienstes der Krankenkassen nach § 275 SGB V vor Ort die Erfüllung der Mindestanforderungen an die sächliche Ausstattung gem. § 12, die Dokumentation gem. § 14 und Leistungserbringung nach diesem Vertrag zu überprüfen.

(4) Die Qualität der Leistungen kann durch die Verbände auch unangekündigt, ggf. unter Hinzuziehung des Medizinischen Dienstes der Krankenversicherung geprüft werden, Hierzu hat der Leistungserbringer alle erforderlichen Unterlagen zur Bewertung der Struktur- und Prozessqualität vorzulegen.

## § 18
## Abrechnung

(1) Die Abrechnung der in der Anlage 8 aufgeführten Komplexpauschalen erfolgt direkt mit den Krankenkassen.

(2) Der Leistungserbringer kann seine Leistungen nur abrechnen, wenn er über ein IK gemäß § 19 verfügt.

(3) Für die Abrechnung gelten die Richtlinien der Spitzenverbände der Krankenkassen über Form und Inhalt des Abrechnungsverfahrens mit sonstigen Leistungserbringern nach § 302 Abs. 2 SGB V (im Folgenden Richtlinien genannt) in der jeweils aktuellen Fassung. Die Abrechnung hat folgende Bestandteile:

- Abrechnungsdaten,
- Urbelege (Verordnungsblätter, im Original),
- ggf. Leistungszusagen der Krankenkassen im Original,
- Leistungsnachweis im Original (Anlage 9),
- Gesamtaufstellung der Abrechnung (Gesamtrechnung, ggf. Sammelrechnung)

Nach § 303 Abs. 3 SGB V dürfen die Krankenkassen Abrechnungen der Leistungserbringer nur vergüten, wenn die jeweils gesetzlich vorgeschriebenen Daten nach § 2 Abs. 1 Buchstabe a) (Abrechnungsdaten) und d) (Gesamtaufstellung der Abrechnung) der Richtlinien im zugelassenen Umfang maschinenlesbar oder auf maschinell verwertbaren Datenträgern angegeben oder übermittelt und die Daten nach Buchstaben b) (Urbelege) und d) (Leistungszusagen) sowie Leistungsnachweise in der dem Leistungserbringer vorliegenden Form vorgelegt worden sind.

Über den Einführungstermin des maschinellen Abrechnungsverfahrens entscheidet die jeweilige Krankenkasse. Ab dem Zeitpunkt der Einführung sind die folgenden Abrechnungsanforderungen zu beachten. Abrechnungen, die nicht den Richtlinien nach § 302 SGB V entsprechen, werden ab diesem Zeitpunkt von den Ersatzkassen abgewiesen.

(4) Der Leistungserbringer ist verpflichtet, sich vor der erstmaligen Datenlieferung nach Abs. 1 bei der zuständigen Institution bzw. den Krankenkassen anzumelden. Dies gilt auch, wenn ein Abrechnungszentrum mit der Erstellung der Abrechnung beauftragt wurde.

Zur Sicherstellung der Vergütung erbrachter Leistungen ist bei der Aufnahme des maschinellen Abrechnungsverfahrens vom Leistungserbringer zunächst eine Erprobungsphase mit den einzelnen Krankenkassen durchzuführen. In der Erprobungsphase erfolgt eine parallele Übermittlung von maschinellen Abrechnungsdaten sowie Papierabrechnungen nach dem bisherigen Abrechnungsverfahren. Dabei sind die maschinellen Daten mit der Kennung "TSOL" als Testdaten zu kennzeichnen. Die maschinellen Abrechnungsdaten und die Papierabrechnungen müssen identisch und vergleichbar sein.

Der Leistungserbringer kann die Erprobungsphase mit einer Krankenkasse beenden, wenn er der Daten annehmenden Stelle der Ersatzkassen dreimal hintereinander technisch und inhaltlich einwandfreie maschinelle Daten übermittelt hat. Dies gilt dann als erfüllt, wenn die betreffende Ersatzkasse dem Leistungserbringer keine Rückmeldung über Fehler in den Daten gibt.

Nach der Beendigung der Erprobungsphase werden vom Leistungserbringer ausschließlich maschinell verwertbare Datenträger übermittelt. Als maschinell verwertbar in diesem Sinne gelten dabei ausschließlich Daten auf elektronischen Datenträgern nach der Technischen Anlage zu den Richtlinien der Spitzenverbände der Krankenkassen. Die Daten sind durch die Kennung "ESOL" als "Echtdaten" zu kennzeichnen.

Die Rechnungslegung erfolgt für alle Versorgungs-/Abrechnungsfälle monatlich. Die maschinell verwertbaren Daten sind an die von den Krankenkassen benannten Stellen zu liefern.

Es werden nur syntaktisch einwandfreie Daten gemäß den Richtlinien angenommen. Fehlerhafte Datenlieferungen sowie nicht korrekt vom Leistungserbringer ausgefüllte Urbelege werden an den Absender mit einem entsprechenden Fehlerhinweis zurückgesendet.

(5) Sofern noch Papierrechnungen akzeptiert werden oder eine Parallelphase vereinbart ist, gelten als maschinell verwertbar ausschließlich Daten auf elektronischen Datenträgern nach der Technischen Anlage zu den Richtlinien der Spitzenverbände der Krankenkassen.

(6) Die Rechnungsbegründenden Unterlagen nach § 2 Abs.1 Buchstabe b) (Urbelege) und d) (Leistungszusagen) der Richtlinie sowie Leistungsnachweise sind jeweils zeitgleich mit der Rechnungslegung (Übermittlung der maschinellen Abrechnungsdaten nach § 2 Abs. 1 Buchstabe a) und e) der Richtlinie) einmal im Monat an die von den Krankenkassen benannten Stellen zu liefern. Die Unterlagen sind im Original in der in den Richtlinien beschriebenen Sortierreihenfolge zu übermitteln. Die Leistungsnachweise sind den zugrunde liegenden Verordnungen zuzuordnen. Nicht ordnungsgemäße oder fehlerhafte Angaben auf den Urbelegen führen zur Abweisung der Rechnung.

Den Rechnungsbegründenden Unterlagen ist bei maschineller Abrechnung ein Begleitzettel gemäß § 2 Abs. 1 Buchstabe f) der Richtlinie beizufügen.

(7) Der Versicherte, ggf. der gesetzliche Vertreter, hat die Abgabe der verordneten Leistungen einmal monatlich durch Unterschrift und Datum auf dem Leistungsnachweis zu bestätigen. Quittierungen im Voraus sind unzulässig.
Der Leistungsnachweis (Muster gemäß Anlage 9) enthält folgende Angaben:

- Name, Anschrift und Institutionskennzeichen des Leistungserbringers
- Name und Vorname, Versichertennummer und Geburtsdatum des Versicherten
- Positionsnummer, Art und Anzahl der erbrachten Leistungen (Positionsnummern gemäß der Richtlinien nach § 302 SGB V)
- Datum und Uhrzeit des Einsatzes (Ende und Beginn)
- monatlich eine Bestätigung der durchgeführten Leistungen durch Unterschrift und Datum eines Verantwortlichen des SAPV-Teams.

Ist der Versicherte nicht in der Lage, den Leistungsnachweis selbst zu unterschreiben, kann die Unterschrift durch einen Vertretungsberechtigten erfolgen. Hierüber ist die Krankenkasse in der Abrechnung schriftlich zu unterrichten. Kann die Unterschrift im Ausnahmefall weder durch den Versicherten noch durch einen Vertretungsberechtigten erbracht werden, bleibt das Unterschriftsfeld auf dem Leistungsnachweis frei. Der Leistungserbringer gibt auf dem Leistungsnachweis, ggf. auf einer Anlage zum Leistungsnachweis, die Gründe hierfür an. Eine Unterschrift für den Versicherten durch den Leistungserbringer oder seine Mitarbeiter ist ausgeschlossen.

(8) In der Abrechnung ist der Leistungserbringergruppenschlüssel (LEGS) gemäß der Richtlinien nach § 302 SGB V anzugeben.

(9) Bei Differenzen bzw. begründeten Beanstandungen der Abrechnung kann die Krankenkasse dem Leistungserbringer die eingereichten Unterlagen oder die Datensätze zur Prüfung bzw. Korrektur zurückgeben.

Beanstandungen müssen innerhalb von 6 Monaten nach Rechnungseingang schriftlich geltend gemacht werden. Rückforderungen können - auch ohne Einverständnis des Zugelassenen - mit der nächsten Abrechnung verrechnet werden. Spätere Rückforderungen können nur mit dem Einverständnis des Leistungserbringers verrechnet werden; es sei denn, es liegt eine unerlaubte Handlung des Leistungserbringers vor.

Die Rechnungen sind - vorbehaltlich einer abschließenden Rechnungsprüfung - grundsätzlich innerhalb von 21 Kalendertagen nach Eingang bei der zuständigen Ersatzkasse/Krankenkasse oder der von ihr benannten Abrechnungsstelle zu bezahlen. Bei Rechnungskürzung gilt dies auch für den unstreitigen Rechnungsbetrag. Als Zahltag gilt der Tag der Überweisung oder der Tag der Übergabe des Überweisungsauftrages an ein Geldinstitut.

Die Bezahlung der Rechnungen auf maschinell verwertbaren Datenträgern erfolgt innerhalb von 14 Tagen nach Eingang der vollständigen Abrechnungsunterlagen bei den von den Ersatzkassen benannten Stellen. Bei Zahlung durch Überweisung gilt die Frist als gewahrt, wenn der Auftrag innerhalb dieser Zeit dem Geldinstitut erteilt wurde.

Abrechnungen auf anderen als nach den Richtlinien definierten Wegen bzw. Datenträgern dürfen die Krankenkassen zurückweisen.

Überträgt der Leistungserbringer die Abrechnung einer Abrechnungsstelle, so hat der Leistungserbringer den zuständige Verband und die Krankenkassen unverzüglich schriftlich hierüber zu informieren. Der Beginn und das Ende des Auftragsverhältnisses, der Name der beauftragten Abrechnungsstelle und das Institutionskennzeichen, unter dem die Abrechnungsstelle die Rechnungslegung vornimmt, ist mitzuteilen. Das Abrechnungszentrum ist verpflichtet, sich ebenfalls gemäß Abs. 2 zum maschinellen Datenaustausch anzumelden. Abrechnungszentren liefern die Abrechnung ausschließlich auf maschinell verwertbaren Datenträgern nach Abs. 1.

Der Leistungserbringer ist für die Einhaltung der gesetzlichen und vertraglichen Regelungen durch das Abrechnungszentrum verantwortlich.

Hat der Leistungserbringer dem Abrechnungszentrum eine Inkasso-Vollmacht erteilt, erfolgt die Zahlung an das Abrechnungszentrum für die Krankenkassen mit schuldbefreiender Wirkung. Wird dem Abrechnungszentrum die Inkasso-Vollmacht entzogen, ist der Leistungserbringer für die Einhaltung des Entzugs der Vollmacht verantwortlich.

Sofern die Rechnungslegung einer Abrechnungsstelle übertragen werden soll, ist der Auftragnehmer unter besonderer Berücksichtigung der von ihm getroffenen technischen und organisatorischen Maßnahmen zur Sicherstellung der Maßgaben dieses Vertrages und des § 6 Abs. 1 BDSG durch den Leistungserbringer auszuwählen. Die getroffene Vereinbarung über Datenschutz und Datensicherung beim Auftragnehmer (Abrechnungsstelle) ist den Verbänden/ Krankenkassen vorzulegen.

(10) Die Abrechnung von Arzneimitteln erfolgt nach der Vereinbarung über die Übermittlung von Daten im Rahmen der Arzneimittelabrechnung gemäß § 300 SGB V und dessen technische Anlagen.

## § 19
## Institutionskennzeichen

(1) Der Leistungserbringer verfügt gemäß § 293 SGB V über ein Institutionskennzeichen (IK), das er bei der Abrechnung mit den Krankenkassen verwendet.

Das IK ist bei der Sammel- und Verteilungsstelle IK der Arbeitsgemeinschaft Institutionskennzeichen (SVI), Alte Heerstr. 111, 53757 St. Augustin, zu beantragen.

Änderungen der unter dem IK gespeicherten Daten sind der SVI unverzüglich mitzuteilen. Mitteilungen an die Krankenkassen oder ihre mit der Abrechnungsprüfung beauftragten Dienstleister werden nicht berücksichtigt.

(2) Das gegenüber den Krankenkassen eingesetzte IK sind den Verbänden sowohl bei Vertragsabschluss als auch bei Änderungen mitzuteilen. Abrechnungen mit den Krankenkassen erfolgen ausschließlich unter diesem IK.

Das IK ist in jeder Abrechnung anzugeben. Abrechnungen ohne IK oder mit fehlerhaftem IK werden von den Krankenkassen abgewiesen. Gleiches gilt für Abrechnungen mit einem der Krankenkassen unbekannten IK.

Die unter dem gegenüber den Krankenkassen verwandten IK bei der SVI gespeicherten Angaben, einschließlich der Bank- und Kontoverbindung sind verbindlich für die Abrechnungsbegleichung durch die Krankenkassen. Andere Bank- und Kontoverbindungen werden von den Krankenkassen bei der Abrechnung nicht berücksichtigt.

## § 20
## Statistik

(1) Der Leistungserbringer ist verpflichtet, an Auswertungen mitzuwirken. Hierzu übermittelt er halbjährlich (30.06., 31.12. d. J.) folgende Daten dem Beirat (vgl. § 21).

(2) Die zu übermittelnden Daten beziehen sich auf folgende Bereiche:

- Fallzahlen (Anzahl der behandelten Patienten mit Angabe der Leistungsarten (gemäß Anlage 8), davon die Anzahl der Krankenhauseinweisungen,
- SAPV leistungsbegründende Diagnosen aufgrund vorliegender ärztlicher Verordnung,
- Versorgungsbereich,
- Zahl der beteiligten Vertragsärzte,
- Zahl der an der SAPV-Versorgung beteiligten Ehrenamtlichen,
- Anzahl der Patienten, die in der Häuslichkeit verstorben sind,
- durchschnittliche Betreuungsdauer,
- Einstufungen der Patienten in Pflegestufen nach dem SGB XI,
- Ort der Leistungserbringung, differenziert nach § 2 Abs. 1, Ziffer 2, Krankenhäuser

## § 21
## Beirat, Aufgaben

(1) Es wird ein Beirat gebildet.

(2) Der Beirat wird mit jeweils 4 Mitgliedern des Leistungserbringers und der Kassenseite besetzt.

(3) Zu den Aufgaben des Beirates gehören u.a. die Begleitung dieser Vereinbarung und Abgabe von Empfehlungen insbesondere hinsichtlich der Qualität der Versorgungsprozesse.

(4) Der Beirat tagt grundsätzlich zweimal jährlich. Die Organisation und Versendung der Einladung zur Sitzung erfolgt durch die Verbände.

(5) Die Aufwendungen für die Teilnahme an den Beiratssitzungen werden durch die jeweiligen Vertragspartner selbst getragen.

## § 22
## Maßnahmen bei Gesetzes- bzw. Vertragsverstößen,

(1) Vertragsverstöße und/oder Schäden sind dem Leistungserbringer durch die Verbände in schriftlicher Form mitzuteilen. Hierbei ist dem Vertragspartner Gelegenheit zur Stellungnahme innerhalb von 14 Tagen nach Zugang des Schreibens zu geben (Anhörung).

(2) Die Verbände entscheiden nach Prüfung der Stellungnahme, welche geeignete Maßnahmen festzulegen sind. Diese könnten sein:

- Verwarnung
- Vertragsstrafe bis zu 10.000 Euro
- außerordentliche Kündigung aus wichtigem Grund, z. B. bei wiederholten oder schwerwiegenden bzw. gröblichen Verstößen.

(4) Die Entscheidung wird dem Leistungserbringer schriftlich mitgeteilt.

## § 23
## Ordentliche / außerordentliche Kündigung

(1) Der Vertrag kann von jedem Vertragspartner unter Angabe des Grundes mit einer Frist von 3 Monaten zum Ende eines Kalendervierteljahres ganz oder teilweise gekündigt werden (ordentliche Kündigung). Die Kündigung bedarf der Schriftform.

(2) Für die Anlagen gelten die dort ggf. gesonderte festgelegten Laufzeiten und Kündigungsfristen.

(3) Eine außerordentliche Kündigung des Vertrages ist nur aus wichtigem Grund möglich. Ein wichtiger Grund, der die Vertragsparteien zur Kündigung berechtigen, liegt insbesondere vor;

a) wenn die Voraussetzungen dieses Vertrages aus Gründen der Rechtsentwicklung, wesentlicher medizinisch-wissenschaftlicher oder tatsächlicher Gründe entfallen,

b) wenn die Leistungen, die Gegenstand dieser Vertrages sind, nicht erbracht oder in erheblichem Umfang mangelhaft, unwirtschaftlich oder unvollständig erbracht werden,

c) bei schwerem Verstoß gegen die Inhalte dieses Vertrages.

(4) Eine außerordentliche Kündigung kann ohne Einhaltung von Kündigungsfristen erfolgen, wenn der Leistungserbringer seinen vertraglichen Pflichten gegenüber den Verbänden/der Krankenkasse derart gröblich verletzt, dass ein Festhalten an diesem Vertrag nicht zumutbar ist.

## § 24
## Inkrafttreten/Laufzeit

(1) Dieser Vertrag tritt am 15.09.2009 in Kraft und kann unter Einhaltung der Kündigungsfrist gemäß § 23 Absatz 1 frühestens zum 30.09.2010 gekündigt werden.

## § 25
## Salvatorische Klausel

(1) Sollten einzelne Bestimmungen dieses Vertrages unwirksam oder undurchführbar sein oder nach Vertragsschluss unwirksam oder undurchführbar werden, bleibt davon die Wirksamkeit des Vertrages im Übrigen unberührt.

(2) An die Stelle der unwirksamen oder undurchführbaren Bestimmung soll diejenige wirksame und durchführbare Regelung treten, deren Wirkungen der Zielsetzung zur Erbringung von SAPV am nächsten kommen, die die Vertragsparteien mit der unwirksamen bzw. undurchführbaren Bestimmung verfolgt haben.

(3) Die vorstehenden Bestimmungen gelten entsprechend für den Fall, dass sich der Vertrag als lückenhaft erweist.

(4) Die Vertragspartner verständigen sich dazu unverzüglich über notwendige neue Regelungen.

Bremen, 25.08.2009

Leistungserbringer

AOK Bremen/Bremerhaven

BKK-Landesverband Niedersachsen-Bremen, zugleich für die Knappschaft, Fachbereich Seekrankenvers.

IKK-gesundplus Bremen (ggf. LV)
zugleich für die Krankenkasse für den Gartenbau, handelnd als Landesverband für die landwirtschaftliche Krankenversicherung in Bremen

Verband der Ersatzkassen e.V. (vdek)
Der Leiter der Landesvertretung Bremen

**Anlagen zum Vertrag nach § 132 d SGB V über die Versorgung mit SAPV im Land Bremen vom 25.08.2009**

Protokollnotiz

Anlage 1 - "Richtlinie zur Verordnung von spezialisierter ambulanter Palliativversorgung gemäß § 37 b SGB V i.V.m. § 92 Abs. 1 Satz 2 Nr. 14 SGB V"
Anlage 2 - "Gemeinsame Empfehlung nach § 132d Abs. 2 SGB V für die spezialisierte ambulante Palliativversorgung"
Anlage 3 - Übersicht Kooperationsvereinbarungen
Anlage 4 - Muster Eingangsassessment
Anlage 5 - Nachweis der vertraglichen Voraussetzungen
Anlage 6 - Meldung Personalwechsel/Handzeichenliste
Anlage 7 - Kerndatensatz
Anlage 8 - Vergütungsvereinbarung
Anlage 9 - Muster Leistungsnachweis

Hamburg

# Vertrag

## über die spezialisierte ambulante palliativmedizinische sowie palliativpflegerische Versorgung in Hamburg gemäß § 132 d SGB V i. V. m. § 37 b SGB V

zwischen

……

- nachfolgend Palliative-Care-Team genannt -

und

der AOK Rheinland/Hamburg - Die Gesundheitskasse
dem BKK Landesverband NORD
der IKK classic
der Knappschaft Regionaldirektion Hamburg

den nachfolgend benannten Ersatzkassen:

BARMER GEK
Techniker Krankenkasse (TK)
Deutsche Angestellten-Krankenkasse (Ersatzkasse)
KKH – Allianz (Ersatzkasse)
HEK – Hanseatische Krankenkasse
hkk

gemeinsamer Bevollmächtigter mit Abschlussbefugnis
Verband der Ersatzkassen e.V. (vdek)
vertreten durch den Leiter der vdek- Landesvertretung Hamburg

- nachfolgend Krankenkassen genannt -

wird folgender Vertrag geschlossen:

**Inhaltsverzeichnis:**


Präambel
§ 1 Gegenstand und Ziele des Vertrages
§ 2 Geltungsbereich
§ 3 Anspruchsvoraussetzungen der SAPV
§ 4 Inhalt und Umfang der spezialisierten ambulanten Palliativversorgung
§ 5 Pflichten der Vertragspartner
§ 6 Palliative-Care-Team
§ 7 Organisatorische, personelle und sächliche Voraussetzungen an das Palliative-Care-Team
§ 8 Weitere Koordinationsaufgaben und Anforderungen
§ 9 Ergänzende Voraussetzungen für Pflegefachkräfte
§ 10 Qualitätssicherung
§ 11 Patientendokumentation
§ 12 Anzeige-/Mitteilungspflichten
§ 13 Kooperation/Sicherstellung der Ehrenamtlichen
§ 14 Fort- und Weiterbildung
§ 15 Grundsätze der Leistungserbringung
§ 16 Verordnung von Arznei- Heil- und Hilfsmitteln im Rahmen der SAPV
§ 17 Verordnung/Genehmigung von SAPV
§ 18 Vorläufige Kostenzusage
§ 19 Prüfung der Leistungsansprüche durch die Krankenkasse
§ 20 Vergütung
§ 21 Leistungsnachweis
§ 22 Abrechnung
§ 23 Rechnungslegung durch Abrechnungsstellen
§ 24 Zahlungsfrist
§ 25 Ausschlussfrist
§ 26 Einflussnahme-, Werbungs- und Vermittlungsverbot
§ 27 Datenschutz, Schweigepflicht
§ 28 Statistik
§ 29 Vertrags-/ Gesetzesverstöße
§ 30 Außerordentliche Kündigung
§ 31 In-Kraft-Treten, Kündigung
§ 32 Änderungen und Ergänzungen
§ 33 Salvatorische Klausel

## Anlagen:

(1) Strukturerhebungsbogen zum Nachweis der vertraglichen Voraussetzungen
(2) Nachweis des eingesetzten Personals, Handzeichenliste
(3) Muster zum Leistungsnachweis
(4) Patienteninformation und Entbindung von der Schweigepflicht
(5) Vergütungsvereinbarung
(6) „Richtlinie des Gemeinsamen Bundesausschusses zur Verordnung von spezialisierter ambulanter Palliativversorgung“ (Spezialisierte Ambulante Palliativversorgungs-Richtlinie/SAPV-RL)
(7) „Gemeinsamen Empfehlungen der Spitzenverbände der Krankenkassen nach § 132 d Abs. 2 SGB V für die spezialisierte ambulante Palliativversorgung“
(8) Gemeinsame Hinweise von DHPV und DGP, Stand 28.11.08 – Punkt I.4
(9) Dokumentation gemäß einer erweiterten Fassung des Datensatzes zur SAPV-Dokumentation der Deutschen Gesellschaft für Palliativmedizin e. V. und des Deutschen Hospiz- und PalliativVerbandes e. V.
(10) Schnellbegutachtungsbogen des Medizinischen Dienstes der Krankenkassen Nord

## Präambel

Das Ziel der spezialisierten ambulanten Palliativversorgung (SAPV) ist, es den Patientinnen und Patienten zu ermöglichen, die letzte Phase ihres Lebens in häuslicher Umgebung zu verbringen. Mit diesem Vertrag soll dem sterbenden Menschen ein würdevolles und selbst bestimmtes Leben in häuslicher Umgebung ermöglicht werden, daher sollen alle an der Versorgung Beteiligten rund um die Uhr eine aufeinander abgestimmte effiziente und wirksame palliativmedizinische und palliativpflegerische Versorgung erbringen. Dieser Vertrag gewährleistet eine optimale spezialisierte ambulante Palliativversorgung und erhöht zugleich die Lebensqualität der Betroffenen und der Angehörigen.

## § 1
## Gegenstand und Ziele des Vertrages

(1) Die spezialisierte ambulante Palliativversorgung gemäß § 37 b SGB V (SAPV) dient dem Ziel, die Lebensqualität und die Selbstbestimmung schwerstkranker Menschen zu erhalten, zu fördern und zu verbessern und ihnen ein menschwürdiges Leben bis zum Tod in ihrer vertrauten häuslichen Umgebung oder in stationären Pflegeeinrichtungen (§ 72 Abs. 1 SGB XI) zu ermöglichen. Versorgungsunsicherheiten, die zu unnötigen und die Versicherten stark belastenden Krankenhauseinweisungen führen, sollen durch das in der SAPV vorgehaltene besondere Angebot vermieden werden. Im Vordergrund steht anstelle eines kurativen Ansatzes die palliativmedizinisch sowie die palliativpflegerische Zielsetzung, Symptome und Leiden einzelfallgerecht zu lindern.

(2) Die individuellen Bedürfnisse und Wünsche der Patientinnen und Patienten sowie die Belange ihrer oder seiner vertrauten Personen stehen im Mittelpunkt der Versorgung. Der Patientenwille, der auch durch Patientenverfügung oder Behandlungsplan zum Ausdruck kommt und allen Beteiligten (Ärztin/Arzt, Angehörigen etc.) bekannt sein muss, ist zu beachten.

(3) Die SAPV ergänzt das bereits bestehende Versorgungsangebot, insbesondere das der Vertragsärzte, Krankenhäuser und Pflegedienste. Sie kann als alleinige Beratungs- und Koordinationsleistung, additiv unterstützende Teilversorgung oder vollständige Patientenbetreuung erbracht werden. Je nach Umfang der Patienten-Teilversorgung vor allem aber bei der Vollversorgung im Rahmen der SAPV, ersetzt diese die ambulante Regelversorgung nach dem SGB V weitgehend oder vollständig. Andere Sozialleistungsansprüche bleiben unberührt (insbesondere SGB XI).

(4) Den besonderen Belangen von Kindern ist Rechnung zu tragen.

(5) Die Anlagen 1 bis 10 sind in der jeweils gültigen Fassung Bestandteil dieses Vertrages.

## § 2
## Geltungsbereich

(1) Dieser Vertrag gilt für die im Rubrum genannten Vertragspartner für das Land Hamburg. Ein Beitritt weiterer Vertragspartner zu dem Palliative-Care-Team ist nur möglich, wenn alle Vertragspartner schriftlich zustimmen.

(2) Der BKK-Landesverband NORD unterzeichnet diesen Vertrag stellvertretend für die teilnehmenden Betriebskrankenkassen. Er stellt dem Palliative-Care-Team zu Beginn und bei Änderungen eine Liste der teilnehmenden Betriebskrankenkassen zur Verfügung.

## § 3
## Anspruchsvoraussetzungen der SAPV

(1) Versicherte haben Anspruch auf SAPV, wenn

a) sie an einer nicht heilbaren, fortschreitenden und so weit fortgeschrittenen Erkrankung leiden, dass dadurch ihre Lebenserwartung begrenzt ist und

b) sie unter Berücksichtigung der in § 1 genannten Ziele eine besonders aufwändige Versorgung benötigen, die nach den medizinischen und pflegerischen Erfordernissen auch ambulant oder in stationären Pflegeeinrichtungen (§ 72 SGB XI) erbracht werden kann.

(2) Versicherte in stationären Hospizen, die unter den in § 1 genannten Zielen eine besonders aufwändige Versorgung benötigen, haben Anspruch auf die Teilleistung der erforderlichen ärztlichen Versorgung im Rahmen der SAPV.

(3) Eine Erkrankung ist nicht heilbar, wenn nach dem allgemein anerkannten Stand der medizinischen Erkenntnisse Behandlungsmaßnahmen nicht zur Beseitigung dieser Erkrankung führen können.

(4) Sie ist fortschreitend, wenn ihr Verlauf trotz medizinischer Maßnahmen nach dem allgemein anerkannten Stand der medizinischen Erkenntnisse nicht nachhaltig aufgehalten werden kann.

(5) Eine Erkrankung ist weit fortgeschritten, wenn die Verbesserung von Symptomatik und Lebensqualität sowie die psychosoziale Betreuung im Vordergrund der Versorgung stehen und nach begründeter Einschätzung der verordnenden Ärztin/des verordnenden Arztes die Lebenserwartung auf wenige Tage, Wochen oder Monate gesunken ist.

(6) Bedarf nach einer besonders aufwändigen Versorgung besteht, soweit die anderweitigen ambulanten Versorgungsformen sowie ggf. die Leistungen des ambulanten Hospizdienstes nicht oder nur unter besonderer Koordination ausreichen würden, um die Ziele nach § 1 Abs. 1 zu erreichen. Anhaltspunkt dafür ist das Vorliegen eines komplexen Symptomgeschehens, dessen Behandlung spezifische palliativmedizinische und/oder palliativpflegerische Kenntnisse und Erfahrungen sowie ein interdisziplinär, insbesondere zwischen Ärzten und Pflegekräften in besonderem Maße abgestimmtes Konzept voraussetzt. Ein Symptomgeschehen ist in der Regel komplex, wenn mindestens eines der nachstehenden Kriterien erfüllt ist:

- ausgeprägte Schmerzsymptomatik
- ausgeprägte neurologische/psychiatrische/psychische Symptomatik
- ausgeprägte respiratorische/kardiale Symptomatik
- ausgeprägte gastrointestinale Symptomatik
- ausgeprägte ulzerierende/exulzerierende Wunden oder Tumore
- ausgeprägte urogenitale Symptomatik

(7) Versicherte, die Leistungen durch das Palliative-Care-Team in Anspruch nehmen möchten, erklären dies mit einer Patienteneinverständniserklärung (Anlage 4).

## § 4
## Inhalt und Umfang der spezialisierten ambulanten Palliativversorgung

(1) Die SAPV umfasst je nach Bedarf alle Leistungen der ambulanten Krankenbehandlung soweit diese erforderlich sind, um die in § 1 Abs. 1 genannten Ziele zu erreichen. Sie umfasst zusätzlich die im Einzelfall erforderliche Koordination der diagnostischen, therapeutischen und pflegerischen Teilleistungen sowie die Beratung, Anleitung und Begleitung der verordnenden oder behandelnden Ärztin/des verordnenden oder behandelnden Arztes sowie der sonstigen an der allgemeinen Versorgung beteiligten Leistungserbringer, der Patienten und ihrer Angehörigen durch Leistungserbringer nach § 132 d SGB V.

(2) SAPV wird ausschließlich von Leistungserbringern nach § 132 d SGB V erbracht. Sie wird nach Bedarf intermittierend oder durchgängig erbracht, soweit das bestehende ambulante Versorgungsangebot (§ 1 Abs. 3), insbesondere die allgemeine Palliativversorgung nicht ausreicht, um die Ziele nach § 1 Abs. 1 zu erreichen. Sie kann dem jeweiligen aktuellen Versorgungsbedarf entsprechend als

1. Beratungsleistung

    (a) Telefonat bzw. persönliches Gespräch mit Betroffenen (Kranken oder deren Angehörige)
    (b) Telefonat bzw. persönliches Gespräch mit einem/einer der Leistungserbringer der Primärversorgung
    (c) Dokumentation und Evaluation

2. Koordination der Versorgung

    (a) Direkter persönlicher Kontakt erforderlich
    (b) Ressourcenfokussierte Versorgungsplanung
    (c) Assessment, Therapie und Notfallplanung
    (d) Vernetzung mit ambulanten und/oder stationären Leistungserbringern
    (e) Dokumentation und Evaluation

3. Additiv unterstützende Teilversorgung

    (a) Beinhaltet Beratungs- und Koordinationsleistung
    (b) Hausbesuche zur Tag- und Nachtzeit erforderlich
    (c) Einzelne Leistungen, insbesondere des in § 5 Abs.3 der SAPV-Richtlinie des G-BA aufgeführten Leistungskatalogs werden bei Bedarf erbracht

(d) Für die verordnete Teilversorgung besteht 24-Stunden-Rufbereitschaft in Absprache mit den Leistungserbringern der Primärversorgung. Die Rufbereitschaft kann nicht als alleinige Teilleistung verordnet werden.
(e) Dokumentation und Evaluation

4. Vollständige Versorgung

(a) Beinhaltet Beratungs- und Koordinationsleistung
(b) Hausbesuche zur Tag- und Nachtzeit erforderlich
(c) Alle Leistungen, insbesondere des in § 5 Abs.3 der SAPV-Richtlinie des G-BA aufgeführten Leistungskatalogs werden bei Bedarf erbracht
(d) Dokumentation und Evaluation

erbracht werden. Die Leistungen müssen ausreichend und zweckmäßig sein, dürfen das Maß des Notwendigen nicht überschreiten und sind wirtschaftlich zu erbringen.

(3) Inhalte der SAPV sind insbesondere:

- Koordination der spezialisierten palliativmedizinischen und palliativpflegerischen Versorgung unter Einbeziehung weiterer Berufsgruppen und von Hospizdiensten im Rahmen einer multiprofessionellen Zusammenarbeit
- Symptomlinderung durch Anwendung von Medikamenten oder anderen Maßnahmen
- Apparative palliativmedizinische Behandlungsmaßnahmen (z. B. Medikamentenpumpe)
- Palliativmedizinische Maßnahmen, die nach ihrer Art, Schwere oder Komplexität eine Kompetenz erfordern, die der einer Ärztin oder eines Arztes mit Zusatzweiterbildung Palliativmedizin entspricht
- Spezialisierte palliativpflegerische Leistungen, die nach ihrer Art, Schwere oder Komplexität eine Kompetenz erfordern, die der einer Pflegefachkraft mit einer curricularen Weiterbildung zu Palliative Care entspricht
- Führung eines individuellen Behandlungsplans, vorbeugendes Krisenmanagement, Bedarfsinterventionen
- Ruf-, Notfall- und Kriseninterventionsbereitschaft rund um die Uhr für die im Rahmen der SAPV betreuten Patienten zur Sicherstellung der im Rahmen der SAPV erforderlichen Maßnahmen
- Beratung, Anleitung und Begleitung der Patienten und ihrer Angehörigen zur palliativen Versorgung einschließlich Unterstützung beim Umgang mit Sterben und Tod
- Spezialisierte Beratung der betreuenden Leistungserbringer der Primärversorgung
- Psychosoziale Unterstützung im Umgang mit schweren Erkrankungen in enger Zusammenarbeit z. B. mit Seelsorge, Sozialarbeit und ambulanten Hospizdiensten
- Organisation regelmäßiger Fallbesprechungen
- Dokumentieren und Evaluieren der wesentlichen Maßnahmen im Rahmen der SAPV

## § 5
## Verpflichtungen der Vertragspartner

(1) Aufgabe des Palliative-Care-Teams ist es, Leistungen der SAPV durch spezialisierte Leistungserbringer, die sich zu einem Palliative-Care-Team zusammengeschlossen haben und Teil einer multiprofessionell vernetzen Versorgungsstruktur im regionalen Gesundheits- und Sozialsystem sind, zu erbringen. Ein Vergütungsanspruch besteht daher erst, wenn das Palliative-Care-Team die nachfolgend formulierten Voraussetzungen erfüllt und die Prüfung, ob das Palliative-Care-Team geeignet und bedarfsgerecht ist, durch die Krankenkasse abgeschlossen ist und dem Palliative-Care-Team das Ergebnis dieser Prüfung mitgeteilt wurde. Erst dann ist das Palliative-Care-Team berechtigt, Leistungen der SAPV zu erbringen und erhält dann die hierfür vereinbarte Vergütung.

(2) Aufgabe der Krankenkasse ist es, zu prüfen, ob das Palliative-Care-Team zur Erreichung der in § 1 genannten Ziele geeignet und bedarfsgerecht ist und im Falle der vertragsgemäßen Leistungserbringung, die vereinbarte Vergütung zu zahlen. Die Krankenkassen prüfen die Geeignetheit des Palliative-Care-Teams anhand der nachfolgend formulierten Voraussetzungen. Darüber hinaus prüfen die Krankenkassen die Bedarfsnotwendigkeit. Um prüfen zu können, ob das Palliative-Care-Team bedarfsnotwendig ist, sind die Kriterien der Gemeinsamen Empfehlung der Spitzenverbände der Krankenkassen nach § 132d Abs. 2 SGB V für die spezialisierte ambulante Palliativversorgung vom 23.06.2008 anzuwenden. Die Grundzüge der Bedarfserhebung und –Feststellung ergeben sich darüber hinaus aus den Gemeinsamen Hinweisen von DHPV und DGP, Stand 28.11.08 – Punkt I.4 (Anlage 8). Eine Abdeckung von 250.000 Einwohnern pro Palliative-Care-Team ist grundsätzlich als ausreichend zu betrachten. Sofern eine bedarfsgerechte Versorgung von SAPV durch die bereits tätigen Palliative-Care-Teams gegeben ist, besteht kein Anspruch auf Vertragsabschluss in der jeweiligen Region.

## § 6
## Palliative-Care-Team

(1) Die Leistungen der SAPV sind nur dann Vertragsgegenstand, wenn sie ausschließlich in einer multiprofessionell vernetzten Versorgungsstruktur erbracht werden. Das Palliative-Care-Team besteht aus mindestens 3 spezialisierten/qualifizierten Ärzten und mindestens 4 qualifizierten Pflegefachkräften, die orientiert an der Konzeption eines Palliative-Care-Teams fachübergreifend in Kooperation mit anderen Professionen eng zusammenarbeiten. Kooperationen mit einer Apotheke und einem ambulanten Hospizdienst, die die Voraussetzungen des § 7 I. Abs. 2 erfüllen, sind verpflichtend. Das Palliative-Care-Team ist ausschließlich oder schwerpunktmäßig in der SAPV tätig. Es gewährleistet eine weitgehende personelle Kontinuität und verfügt über eine besondere Ausstattung, die eine palliativmedizinische und palliativpflegerische Versorgung im Zusammenwirken mit den weiteren Leistungserbringern gewährleistet. Es muss in der Lage sein, in seinem gesamten Versorgungsgebiet sowohl ärztlich als auch pflegerisch eine aufsuchende Behandlung und Pflege sowohl tagsüber als auch nachts, an Wochenenden und

Feiertagen in Form einer 24-Stunden-Bereitschaft zu gewährleisten. Die organisatorischen, personellen und sächlichen Voraussetzungen, die das Palliative-Care-Team ständig erfüllen muss, sind in § 7 dieses Vertrages definiert.

(2) Das Palliative-Care-Team hat der Krankenkasse ein schriftliches Konzept vor Vertragsschluss vorzulegen. Das Konzept hat darzustellen, in welcher Form die Koordination durch das Palliative-Care-Team erfolgt. Hierzu muss das Palliative-Care-Team ein verbindlich strukturiertes und schriftlich dargelegtes Versorgungskonzept vorlegen, das alle medizinisch-pflegerischen Maßnahmen klar und eindeutig unter Beachtung von Maßnahmen zur Qualitätssicherung formuliert und darüber hinaus verdeutlicht, wie eine qualifizierte Leistungserbringung zu erfolgen hat, damit die in § 1 dieses Vertrages genannten Vertragsziele erreicht werden. Ein Vergütungsanspruch auf der Grundlage dieses Vertrages ist nur dann gegeben, wenn die Krankenkasse das vorgelegte Konzept für geeignet erachtet.

(3) Das Palliative-Care-Team handelt aufgrund eines verbindlichen, strukturierten und schriftlich dargelegten Versorgungskonzepts, das mindestens folgende Punkte beinhaltet:

(a) Standort des Leistungserbringers,
(b) Versorgungsgebiet,
(c) Versorgungsprozess,
(d) Kooperationspartner,
(e) Kooperationsvereinbarungen mit an der Versorgung Beteiligten im Versorgungsgebiet; verpflichtend mit einem ambulanten Hospizdienst nach § 39a Abs. 2 SGB V, zur Sicherung der psychosozialen Unterstützung sowie einer Apotheke, die die Voraussetzungen gem. § 7 I. Abs. 2 erfüllt,
(f) Mitarbeiterkapazität (ggf. Stellenplan),
(g) personelle Zuständig- und Verantwortlichkeiten (u.a. Weisungsbefugnisse, Weisungsgebundenheit, u.a.),
(h) Qualifikationsnachweise der Leistungserbringer gem. § 7,
(i) sächliche Ausstattung gem. § 7,
(j) Qualitätssicherungsmaßnahmen gemäß § 10,
(k) Konzept zur Sicherstellung der reibungslosen Versorgung mit Arznei-, Heil-, und Hilfsmitteln,
(l) Konzept zur Sicherstellung der 24-Stunden-Rufbereitschaft,
(m) ein Fort- und Weiterbildungskonzept.

(4) Die regionalen Vernetzungsstrukturen sind zu beschreiben, in die sich der Leistungserbringer im Rahmen der Vernetzung integriert.

## § 7
## Organisatorische, personelle und sächliche Voraussetzungen an das Palliative-Care-Team

### I. Organisatorische Voraussetzungen

(1) Das Palliative-Care-Team muss seine rechtsfähige Organisationsform den Krankenkassen in geeigneter Form nachweisen. Hierzu ist insbesondere die Anlage 1 vollständig auszufüllen.

(2) Das Palliative-Care-Team kooperiert mindestens mit einer Apotheke, um eine Lagerhaltung an Medikamenten für den Notfall sicherzustellen, die mindestens folgende Voraussetzungen erfüllt:

- Zulassung als Apotheke
- Vorhaltung von palliativmedizinisch relevanten Arzneimitteln
- Fortbildung zum Fachapotheker für Offizin- oder onkologische Pharmazie

sowie einem ambulanten Hospizdienst, der die Voraussetzungen gem. § 39 a Abs. 2 SGB V erfüllt.

### II. Personelle Voraussetzungen

(1) Die Leistungen der SAPV werden ausschließlich durch geeignete Personen erbracht, welche die nachfolgend formulierten Qualifikationen erfüllen. Das Palliative-Care-Team stellt sicher, dass diese geeigneten Personen im Bedarfsfall hauptsächlich für die Leistungen der SAPV zur Verfügung stehen. Das Palliative-Care-Team weißt mindestens drei geeignete Ärztinnen/Ärzte sowie mindestens vier geeignete Pflegefachkräfte nach.

a) Geeignete Ärztinnen/Ärzte nach Abs. 1 verfügen über folgende Qualifikationen:

- eine anerkannte Zusatzweiterbildung Palliativmedizin nach der aktuell gültigen Weiterbildung der jeweiligen Landesärztekammer (grds. 160 Std. Weiterbildung), (vergl. Protokollnotiz 1)
- Erfahrungen aus der ambulanten palliativen Behandlung von mindestens 75 Palliativpatienten, z. B. in der häuslichen Umgebung oder in einem stationären Hospiz, innerhalb der letzten drei Jahre oder aus einer mindestens einjährigen klinischen palliativmedizinischen Tätigkeit in einer Palliativabteilung in einem Krankenhaus innerhalb der letzten drei Jahre.

b) Geeignete Pflegefachkräfte nach Abs. 1 verfügen über folgende Qualifikationen:

- die Erlaubnis zur Führung einer der Berufsbezeichnungen Gesundheits- und Krankenpflegerin/Gesundheits- und Krankenpfleger, Gesundheits- und Kinderkrankenpflegerin/Gesundheits- und Kinderkrankenpfleger oder Altenpflegerin/Altenpfleger (Berechtigung zur Führung der Berufsbezeichnung nach dem Gesetz über die Berufe in der Altenpflege vom 25.08.2003) entsprechend den gesetzlichen Bestimmungen in der jeweils gültigen Fassung
- den Abschluss einer Palliative-Care-Weiterbildungsmaßnahme im Umfang von mindestens 160 Stunden; die Weiterbildung hat inhaltlich den Vorgaben des Basiscurriculum Palliativ Pflege des Vereins zur Betreuung und Begleitung von Schwerstkranken und Tumorpatienten e. V., Pallia Med Verlag, Bonn, zu entsprechen, (vergl. Protokollnotiz 2)
- Erfahrungen durch mindestens eine zweijährige praktische Tätigkeit als Pflegefachkraft in der Betreuung von Palliativpatienten in den letzten drei Jahren; davon mindestens sechsmonatige Mitarbeit in einer spezialisierten Einrichtung der Hospiz- und Palliativversorgung.

c) Soweit weitere Fachkräfte (z. B. Sozialarbeiterinnen/Sozialarbeiter, Sozialpädagoginnen/Sozialpädagogen, Psychologinnen/Psychologen) vertraglich eingebunden werden, z. B. im Rahmen des vorzulegenden Versorgungskonzepts als Kooperationspartner genannt sind, weist das Palliative-Care-Team nach, dass diese Fachkräfte über eine Zusatzweiterbildung Palliative Care für andere Berufsgruppen oder eine mehrjährige Erfahrung in der Palliativversorgung verfügen.

(2) Eine tägliche telefonische Rund-um-die-Uhr-Erreichbarkeit für die Patientin/den Patienten, deren Angehörigen und die an der Versorgung Beteiligten ist durch das Palliative-Care-Team sicherzustellen. Die ständige Verfügbarkeit mindestens einer Ärztin/eines Arztes und einer Pflegefachkraft ist zu garantieren. Diese Verfügbarkeit schließt notwendige Hausbesuche ein.

## III. Sachliche Voraussetzungen

(1) Das Palliative-Care-Team hat als Mindestanforderung an die sächliche Ausstattung Folgendes vorzuhalten bzw. sicherzustellen:

- das Palliative-Care-Team hat unter der Voraussetzung, dass eine entsprechende Patienteninformation und Entbindung von der Schweigepflicht (s. § 3 Abs. 6/Anlage 4) vorliegt, eine Patientendokumentation zu erstellen, die den Anforderungen der Gemeinsamen Empfehlungen der Spitzenverbände der Krankenkassen nach § 132 d Abs. 2 SGB V für die SAPV, Punkt 6.2. entspricht (Anlage 7),
- Arzneimittel (inkl. BtM) für die Notfall/Krisenintervention,

- Arzt-/Pflegekoffer/Bereitschaftstasche (Berücksichtigung der Kompatibilität der Verbrauchsmaterialien zu Medizinprodukten unterschiedlicher Hersteller, z. B. bei Portsystemen oder Infusionspumpen),
- eine geeignete administrative Infrastruktur, z. B. Büro, Kommunikationstechnik.

(2) Das Palliative-Care-Team muss über eine eigenständige Adresse und geeignete Räumlichkeiten für

- die Beratung von Patientinnen/Patienten und Angehörigen
- Teamsitzungen und Besprechungen
- die Lagerhaltung von eigenen Medikamenten für Notfall-/Krisenintervention und Hilfsmitteln

verfügen.

## IV. Nachweis der Voraussetzungen

Zum Nachweis der organisatorischen und personellen Voraussetzungen hat das Palliative-Care-Team den Krankenkassen zur Überprüfung, ob die Voraussetzungen zum Abschluss dieses Vertrages erfüllt werden, mindestens die nachfolgenden Unterlagen vor Vertragsabschluss und auf Anforderung einzureichen:

(a) Nachweis der aktuell beschäftigten Mitarbeiterinnen/Mitarbeiter sowie die Qualifikationsnachweise des eingesetzten Personals inkl. Nachweis zur Dokumentation der Unterschriften/Handzeichen,
(b) Nachweis eines Institutionskennzeichens (IK) Palliative-Care-Team spezifisch,
(c) Nachweis der Berufserfahrung für den Arzt und die leitende und stellvertretende leitende Pflegefachkraft (vgl. § 9),
(d) bei der Rechtsform:

1. einer Gesellschaft bürgerlichen Rechts (GbR) einen Auszug aus dem Gesellschaftsvertrag in Kopie mit Angabe der Gesellschafter, Geschäftsführung, Unternehmenszweck,
2. bei einer Gesellschaft mit beschränkter Haftung (GmbH) einen Auszug aus dem notariell beurkundeten Gesellschaftsvertrag in Kopie mit Angabe der Gesellschafter, Geschäftsführung, Unternehmenszweck sowie einen beglaubigten Auszug aus dem Handelsregister des zuständigen Amtsgerichts,
3. einem eingetragen Verein (e. V.) einen Auszug aus der Vereinssatzung in Kopie mit Angabe der Vorstandsmitglieder, Geschäftsführung, Vereinszweck sowie einen beglaubigten Auszug aus dem Vereinsregister,
4. für andere Gesellschaftsformen und Körperschaften, Anstalten und Stiftungen gelten die Nachweispflichten entsprechend.

(6) ggf. bestehende Kooperationsverträge.

## § 8
## Weitere Koordinationsaufgaben und Anforderungen

(1) An das Palliative-Care-Team werden folgende weitere Koordinationsaufgaben und Anforderungen gestellt:

- Klare Benennung und Zuordnung der Koordinationsfunktionen,
- Zentrale Anlaufstelle,
- Sicherstellung eines örtlichen abgestimmten Notfallplanes,
- Koordinierung einer Vertretungsregelung und einer Rund-um-die-Uhr-Bereitschaft,
- Organisation der Initial- und Folgebesuche gemäß Abs. 3,
- Fallbesprechungen nach Abs. 2 sowie der Qualitätssicherungsmaßnahmen nach § 10,
- Das Palliative-Care-Team ist zudem verpflichtet, eine additiv unterstützende Teilversorgung in Form von ärztlicher und pflegerischer Leistung zu erbringen, und zwar in folgen Fällen:
  - Sofern ein Vertragsärztin/ein Vertragsarzt Leistungen der APV (allgemeine Palliativversorgung) erbringt und additiv unterstützende Hilfe beim Palliative-Care-Team anfordert;
  - Sofern eine Ärztin/ein Arzt, die/der im stationären Hospizdienst tätig ist, beim Palliative-Care-Team additiv unterstützende Hilfe anfordert/verordnet, ist diese in Form von ärztlicher Leistung im Rahmen dieses Vertrages zu erbringen,
  - Das Palliative-Care-Team ist verpflichtet, eine additiv unterstützende Teilversorgung zu erbringen, sofern Ärzte, die in stationären Pflegeeinrichtungen tätig sind, solche Leistungen beim Palliative-Care-Team anfordern.

(2) Im Rahmen des gemeinsamen Initialbesuches durch das Palliative-Care-Team (qualifizierte Palliativ-Ärztin/qualifizierter Palliativ-Arzt und ambulanter Palliativ-Pflegedienst) bei der Patientin/beim Patienten erfolgt die integrative Abstimmung der notwendigen Maßnahmen und des Behandlungs- und Therapieplanes. Gleichzeitig wird ein Zeitpunkt (innerhalb von 14 Tagen) festgelegt, an dem eine erneute gemeinsame Fallbesprechung erfolgt, um ggf. die notwendigen Maßnahmen sowie den Behandlungs- und Therapieplan anzupassen. Die behandelnde Hausärztin/der behandelnde Hausarzt sollte beim Initialbesuch sowie bei der Fallbesprechung mit eingebunden werden.

(3) Die qualifizierte Palliativ-Ärztin/der qualifizierte Palliativ-Arzt des Palliative-Care-Teams übernimmt insbesondere folgende Aufgaben:

- Durchführung der beim Initialbesuch abgestimmten ärztlichen Maßnahmen,
- Einstellung und/oder Beratung zur Schmerztherapie und Symptomkontrolle,
- Verordnung von Medikamentenapplikationen über Pumpensysteme unter besonderer Berücksichtigung des Wirtschaftlichkeitsgebotes,
- Beratung zur künstlichen Ernährung und Flüssigkeitsgabe in der letzten Lebensphase,
- Beurteilung und Einleitung einer palliativen Wundbehandlung,

- Palliativmedizinische Betreuung und Beratung von Patientinnen/Patienten und deren Angehörigen,
- Unterstützung in der Aufklärung der Patientin/des Patienten, der Vermittlung „schlechter Nachrichten“ und Information über die Änderung des Therapiezieles,
- Beratung bei ethischen Konflikten (ethisches Fallgespräch).

## § 9
## Ergänzende Voraussetzungen für Pflegefachkräfte

(1) Gemäß § 7 II. Abs. 2 b müssen im Palliative-Care-Team mindestens vier Vollzeitstellen mit Pflegefachkräften besetzt werden, die einen Arbeitsvertrag mit dem bzw. einem Leistungserbringer abgeschlossen haben, der mit den Krankenkassen einen Vertrag nach § 132 a SGB V hat. Davon müssen mindestens zwei Pflegefachkräfte sozialversicherungspflichtig in Vollzeit beschäftigt sein. Die verbleibenden zwei Vollzeitstellen können auch durch eine entsprechende Anzahl von sozialversicherungspflichtigen Teilzeitbeschäftigten mit einer wöchentlichen Arbeitszeit von mindestens 18 Stunden besetzt werden. Inhaber oder Gesellschafter, die hauptberuflich im Palliativbereich mitarbeiten und die Qualifikationsvoraussetzungen erfüllen, sind Vollzeitbeschäftigten gleichgestellt. (Protokollnotiz 3)

(2) Das Palliative-Care-Team hält eine pflegefachliche Leitung vor. Diese Funktion der leitenden Pflegefachkraft muss von einer gemäß Abs. 1 qualifizierten Pflegefachkraft in Vollzeitbeschäftigung wahrgenommen werden, die die im § 7 II. Abs. 2 geforderten Voraussetzungen erfüllen.

(3) Als Stellvertretung der leitenden Pflegefachkraft hält das Palliative-Care-Team eine Pflegefachkraft mit einem Beschäftigungsumfang von mindestens 18 Std. Wochenarbeitszeit vor, die die Voraussetzungen des Abs. 2 und des Abs. 3 Satz 1 erfüllt. Im Falle der planbaren Abwesenheitsvertretung der leitenden Pflegefachkraft ist eine Vollzeitbesetzung durch die Stellvertretung sicherzustellen.

## § 10
## Qualitätssicherung

(1) Das Palliative-Care-Team ist verpflichtet, ein internes Qualitätsmanagement durchzuführen. Es nimmt regelmäßig an palliativmedizinischen/-pflegerischen Fortbildungen teil und führt möglichst halbjährlich multidisziplinäre Qualitätszirkel durch, an denen auch die übrigen in der Versorgung Tätigen teilnehmen können / sollen. Das Palliative-Care-Team soll sich außerdem an Maßnahmen der externen Qualitätssicherung beteiligen. Ergänzend gelten die Regelungen des § 14.

(2) Die Teilnahme an Supervisionen ist zu ermöglichen. Regelmäßige multiprofessionelle Fallbesprechungen sind in überschaubaren Intervallen durchzuführen; die inhaltliche und zeitliche Organisation ist den Beteiligten entsprechend der Konzeption zu überlassen.

(3) Das Palliative-Care-Team erstellt jeweils nach gemeinsam erfolgtem Initialbesuch einen individuellen Behandlungsplan, der mit den übrigen an der Versorgung beteiligten Leistungserbringern abzustimmen ist.

(4) Vor jeder Krankenhauseinweisung hat zwingend eine Begutachtung der Patientin/des Patienten durch die qualifizierte Palliativ-Ärztin/den qualifizierten Palliativ-Arzt des Palliative-Care-Teams zu erfolgen. Ausgenommen hiervon sind sämtlich notfallmedizinische Einweisungen. Das Wohl und der Wille der Patientin/des Patienten sind hierbei zu berücksichtigen und stehen daher im Vordergrund.

(5) Die Krankenkassen haben das Recht, durch Beauftragung des Medizinischen Dienstes der Krankenversicherung vor Ort die Erfüllung der Mindestanforderungen an die sächliche Ausstattung, die Dokumentation sowie die Qualität der Leistungserbringung nach diesem Vertrag zu überprüfen.

## § 11
## Patientendokumentation

(1) Das Palliative-Care-Team hat eine Patientendokumentation zu führen, aus der zu jeder Zeit die notwendigen Informationen über die Versicherte/den Versicherten und die erbrachten Leistungen hervorgehen. Es soll kompatibel zu den bestehenden Dokumentationssystemen der an der Versorgung beteiligter Leistungserbringer sein und eine bundesweite Evaluation ermöglichen. Bevorzugt ist der gemeinsame Datensatz zur SAPV-Dokumentation der Deutschen Gesellschaft für Palliativmedizin e. V. und des Deutschen Hospiz- und PalliativVerbandes e. V. (Anlage 9) zu verwenden. Die Patientendokumentation wird regelmäßig von dem Palliative-Care-Team aktualisiert.

(2) Die Dokumentation beinhaltet insbesondere:

a) ein Versichertenstammblatt
(z. B. mit Angaben zur Versicherten/zum Versicherten und Angehörigen, Hausärztin/Hausarzt, Pflegedienst, Diagnosen, Absprachen, Behandlungsort, Medikation, Maßnahmen, u. s. w.),

b) ein Assessment mit folgenden Inhalten:

- Aufklärung der Versicherten/des Versicherten und deren Angehörigen,
- Therapieplanung,
- Ethische Konflikte,
- Funktionale Selbstständigkeit,
- Familiäre Belastungssituation,
- Einschätzung Symptomkontrolle,
- Medikation,
- Bedarfsmedikation,
- Hilfsmittel,
- Physikalische und andere Therapien (z. B. Lymphdrainage),
- Beratungsbedarf.

c) einen Maßnahmen-Plan/Durchführungsnachweis
d) Nachweise über die durchgeführten Maßnahmen in den Bereichen Beratung, Koordination, Pflege, Medizin, psychosoziale Unterstützung (Datum, Uhrzeit)
e) einen Notfallplan/vorausschauender Behandlungsplan
f) Untersuchungsbefunde
g) das vertragsärztliche Verordnungsblatt für die Dokumentation der medizinischen Diagnostik und Therapie
h) eine Pflege- und Therapieplanung (Therapiebeginn, medizinische/pflegerische/psychosoziale Versorgungssituation, Therapie- und Pflegeziele).

(3) Jede an der Versicherten/am Versicherten erbrachte Leistung wird durch die behandelnde qualifizierte Person dokumentiert und durch Handzeichen gegengezeichnet. Zusätzlich werden in dieser Patientenakte die Besuche durch die Hausärztin/den Hausarzt, wie auch allen kooperierenden Mitwirkenden dokumentiert.

(4) Sämtliche zur Dokumentation gehörende Dokumente verbleiben beim Versicherten.

(5) Die Dokumentation ist dem Medizinischen Dienst der Krankenversicherung - soweit die Versicherte/der Versicherte eingewilligt hat - auf Anforderung zugänglich zu machen

(6) Die Aufbewahrungsfrist für die Dokumentation beträgt zehn Jahre nach Ende des Kalenderjahres der Leistungserbringung.

## § 12
## Anzeige-/Mitteilungspflichten

(1) Jeweils zum 31.03. eines Jahres hat die Pflegeeinrichtung den Nachweis der aktuell beschäftigten Mitarbeiter einschließlich der Unterschrift/Handzeichen (Anlage 2) sowie Kopien der Qualifikationsnachweise für das jeweils eingesetzte Personal den von den Krankenkassen genannten zuständigen Stellen vorzulegen. Soweit Qualifikationsnachweise bereits vorgelegt wurden, sind diese nicht erneut einzureichen.

(2) Das Palliative-Care-Team ist verpflichtet, den Krankenkassen alle wesentlichen Umstände unverzüglich schriftlich mitzuteilen, die die vertragsgemäße Durchführung dieses Vertrages sowie dessen Fortbestand berühren. Unverzüglich anzuzeigen sind hiernach insbesondere:

- (a) das Ausscheiden einer qualifizierten Palliativ-Ärztin/eines qualifizierten Palliativ-Arztes, sowie einer qualifizierten Pflegefachkraft
- (b) Änderungen der betrieblichen/organisatorischen Gegebenheiten, die im Zusammenhang mit dem Versorgungskonzept stehen,
- (c) der Abschluss, die Kündigung bzw. Änderungen von Kooperationsvereinbarungen,
- (d) jede Änderung der Trägerschaft/Betriebsinhaberschaft sowie der Rechtsform des Palliative-Care-Teams,
- (e) den Umstand, dass Antrag auf Eröffnung eines Insolvenzverfahrens gegen das Palliative-Care-Team oder gegen einen Leistungserbringer gestellt wurde,
- (f) eingeleitete strafrechtliche Ermittlungsverfahren gegen einen Partner des Palliative-Care-Teams oder das Palliative-Care-Team, soweit sie im Zusammenhang mit der Vertragserfüllung stehen.
- (g) Aufnahme weiterer Leistungserbringer in das Palliative-Care-Team
- (h) Wechsel der leitenden Pflegefachkraft oder deren Stellvertretung
- (i) Unterschreitung der personellen Mindestanforderungen

## § 13
## Kooperation/Sicherstellung der Ehrenamtlichen

Die Sicherstellung der psychosozialen Unterstützung (§ 4 Abs. 3, 10. Spiegelstrich) hat das Palliative-Care-Team durch eine Kooperation mit ambulanten Hospizdiensten, die nach § 39 a Abs. 2 SGB V gefördert werden, nachzuweisen. Es ist eine schriftliche Kooperationsvereinbarung mit folgenden Mindestinhalten abzuschließen:

- (a) Erklärung des/der ambulanten Hospizdienste(s) über seine/ ihre Förderungsfähigkeit nach § 39 a Abs. 2 SGB V.
- (b) Sofern Ehrenamtliche Leistungen nach dem SGB V/XI erbringen, sind diese nicht abrechnungsfähig
- (c) Kooperationen, die ihrem Zweck nach dazu dienen, fehlende organisatorische oder personelle Voraussetzungen zu ersetzen, sind unzulässig.

## § 14
## Fort- und Weiterbildung

(1) Das Palliative-Care-Team hat auf der Grundlage des vorgelegten Fort- und Weiterbildungskonzeptes die Mitarbeiterinnen und Mitarbeiter fortzubilden. Die Inhalte der Fortbildungen sind so auszurichten, dass die Mitarbeiterinnen/die Mitarbeiter auf dem aktuellen Stand der allgemein anerkannten ärztlich-medizinischen und pflegewissenschaftlichen Erkenntnissen sind. Die Leitungskräfte des Palliative-Care-Teams sind verpflichtet, externe fachbezogene Fortbildungen oder Inhouse-Schulungen von kalenderjährlich jeweils 8 Stunden nachzuweisen. Hierbei ist darauf zu achten, dass diese Fortbildungen die Bereiche Dokumentation, Management und Pflege abdecken. Für die übrigen Mitarbeiterinnen/Mitarbeiter sind regelmäßige Fortbildungen, die sich an den Inhalten des Satzes 1 orientieren sollten, durchzuführen.

Als Nachweis dienen die entsprechenden Zertifikate bzw. Kopien der Veranstaltungsprotokolle (Teilnehmerliste). Der Nachweis der kalenderjährlich durchgeführten Fortbildung ist bis zum 31.03. des Folgejahres den Krankenkassen vorzulegen.

(2) Werden Fortbildungen, wie in Absatz 1 beschrieben, nicht fristgerecht nachgewiesen, werden vom Folgemonat an die Vergütungssätze nach Maßgabe des § 20 Abs. 4 gekürzt. Dem Palliative-Care-Team ist eine Frist zu setzen, innerhalb derer es die Fortbildung nachholen kann. Als Frist hierfür ist längstens ein Zeitraum von sechs Monaten möglich.

(3) Wird die Fortbildung vor Beendigung der Frist nachgewiesen, entfällt vom nächsten Monatsersten an die Kürzung der Vergütung.

## § 15
## Grundsätze der Leistungserbringung

(1) Das Palliative-Care-Team hat darauf zu achten, dass die Leistungen wirksam und wirtschaftlich erbracht und nur im notwendigen Umfang in Anspruch genommen werden (§ 2 Abs. 4 SGB V).

(2) Die Leistungen müssen ausreichend, zweckmäßig und wirtschaftlich sein; sie dürfen das Maß des Notwendigen nicht überschreiten. Leistungen, die nicht notwendig oder unwirtschaftlich sind, können Versicherte nicht beanspruchen, darf das Palliative-Care-Team nicht bewirken und die Krankenkasse nicht bewilligen (§ 12 Abs. 1 SGB V).

(3) Das Palliative-Care-Team gewährleistet, dass die Versicherten aller vertragsschließenden Krankenkassen nach gleichen Grundsätzen behandelt werden.

(4) Zur Optimierung der Versorgung mit Leistungen, insbesondere um einen reibungslosen und unverzüglichen Übergang von der stationären Krankenhausbehandlung zur ambulanten palliativmedizinischen und palliativpflegerischen Versorgung zu gewährleisten, hat das Palliative-Care-Team mit

(a) Vertragsärzten,
(b) ambulanten Hospizdiensten,
(c) stationären Hospizen,
(d) Vertragskrankenhäusern,
(e) Rehabilitationseinrichtungen,
(f) und sonstigen innerhalb des Versorgungsspektrums Beteiligten

integrativ zusammenzuarbeiten.

(5) Das Palliative-Care-Team gewährleistet in seinem Geltungsbereich (vgl. § 2 und § 6 Abs. 3), dass zur Sicherstellung einer fach- und sachgerechten ambulanten palliativmedizinischen und palliativpflegerischen Versorgung nach diesem Vertrag fachlich qualifiziertes Personal eingesetzt wird und eine fach- und sachgerechte ambulante palliativmedizinische und palliativpflegerische Versorgung erfolgt.

(6) Das Palliative-Care-Team trägt die fachliche Verantwortung für das eingesetzte Personal. Über den Einsatz des Personals im Einzelfall entscheidet das Palliative-Care-Team unter Beachtung der medizinischen Notwendigkeit sowie Art und Schwere der Erkrankung der Versicherten/des Versicherten im Rahmen des Wirtschaftlichkeitsgebotes. Die Erbringung der vertraglichen Leistungen hat mit möglichst geringem Wechsel des Personals zu erfolgen.

## § 16
## Verordnung von Arznei-, Heil- und Hilfsmitteln im Rahmen der SAPV

(1) Der qualifizierte Palliativarzt des Palliative-Care-Teams verordnet die notwendigen Arznei-, Heil- und Hilfsmittel unter Verwendung der für die vertragsärztliche Verordnung geltenden Muster (Muster 13, 14, 16 und 18) auf den Namen des Versicherten.

(2) Das Palliative-Care-Team benötigt für die Abrechnung der Verordnungen eine Betriebsstättennummer, die neben der Pseudo-Arzt-Nummer auf den verwendeten Vordrucken einzutragen ist. Diese wird von der Kassenärztlichen Bundesvereinigung vergeben. Der Leistungserbringer ist verpflichtet, sich um den Erhalt der Betriebsstättennummer zu kümmern.

(3) Die Vertragsparteien vereinbaren, dass die Wirtschaftlichkeit der Arznei-, Heil- und Hilfsmittelverordnungen des Palliative-Care-Teams in entsprechender Anwendung der

nach §§ 106 a, 106 Abs. 2 und 3 geltenden Regelungen von den Krankenkassen geprüft werden können. Das Nähere zur Umsetzung der Wirtschaftlichkeitsprüfung regeln die Krankenkassen.

## § 17
## Verordnung/Genehmigung von SAPV

(1) Art, Umfang und Dauer der vom Palliative-Care-Team zu erbringenden Leistungen ergeben sich aus der von der Krankenkasse genehmigten Verordnung der behandelnden Vertragsärztin/des behandelnden Vertragsarztes oder der Krankenhausärztin/des Krankenhausarztes.

(2) Die Kostenübernahme ist vor Beginn der SAPV bei der zuständigen Krankenkasse mit dem hierfür vereinbarten Vordruck zu beantragen, soweit dieser Vordruck noch nicht vorliegt auf der vertragsärztlichen Verordnung nach Muster 12. Der aktuelle Schnellbegutachtungsbogen für SAPV (Anlage 10) ist entsprechend des Ergebnisses des Eingangsassessment vom Palliative-Care-Team mit einzureichen.

(3) Die Verordnung ist nicht übertragbar. Sie gilt nur für die Person, für die sie ausgestellt ist. Änderungen und Ergänzungen bedürfen der Unterschrift der verordnenden Vertragsärztin/des verordnenden Vertragsarztes.

(4) Leistungen der SAPV werden von den Krankenkasse nur genehmigt, wenn eine ordnungsgemäß ausgefüllte ärztliche Verordnung (Muster 63) vorliegt. Die Versicherte/der Versicherte oder ihr/sein gesetzlicher Vertreter hat die Genehmigung der Verordnung schriftlich mittels des vorgegebenen Formulars zu beantragen.

(5) Die Unterschrift durch eine Mitarbeiterin/einen Mitarbeiter des Teams ist ausgeschlossen. Kann die Unterschrift nicht erbracht werden, weil die Versicherte/der Versicherte bzw. ihr/sein gesetzlicher Vertreter nicht in der Lage ist zu unterschreiben, bleibt das Unterschriftsfeld auf der Verordnung frei. Das Palliative-Care-Team gibt zur Verordnung die Gründe hierfür an.

(6) Die SAPV-RL ist für die Verordnung sowie für die Genehmigung durch die Krankenkasse maßgeblich. Dort nicht aufgeführte Inhalte bedürfen einer nachvollziehbaren Begründung, damit sie von den Krankenkassen genehmigt werden können.

(7) Die verordnende Ärztin/der verordnende Arzt legt nach aktuellem Versorgungsbedarf die Dauer der Verordnung fest. Die Krankenhausärztin/der Krankenhausarzt kann Leistungen in der Regel jedoch längstens für sieben Tage verordnen. Sollte eine Versorgung für einen längeren Zeitraum als sieben Tage erforderlich sein, ist rechtzeitig eine Folgeverordnung für den Zeitraum über den siebten Tag hinaus bei der zuständigen Krankenkasse einzureichen.

(8) Der qualifizierte Palliativmediziner prüft im Rahmen eines Eingangsassessment, innerhalb der ersten 3 Arbeitstage nach Erhalt der Verordnung die Notwendigkeit und Eignung der besonderen Mittel der SAPV in den beantragten Einzelfällen. Sofern die Notwendigkeiten für SAPV nicht oder nur für einen kürzeren als erst- verordneten Zeitraum gegeben sind, somit die Mittel der Regelversorgung einschließlich der AAPV ausreichen, informiert er unverzüglich Krankenkassen, Versicherte (ggf. deren Angehörige) und Erstverordner über die Ergebnisse des SAPV-Assessments. Ggf. sind koordinierende Gespräche nötig und Empfehlungen an die weiter behandelnden Personen zu geben.

Für die Benachrichtigung an die Kasse ist auf schnelle Medien wie Fax, E-Mail oder Telefon zurück zu greifen. Das schriftliche Ergebnis des Eingangsassessments ist auf dem Postweg an die Kasse nach zu reichen.

(9) Die Verordnung für einen zurückliegenden Zeitraum ist nur in begründeten Ausnahmefällen zulässig.

(10) Kann die Durchführung der verordneten Leistung nicht mehr erfolgen, z. B. weil die Versicherte/der Versicherte in ein Krankenhaus eingewiesen wurde, informiert das Palliative-Care-Team die zuständige Krankenkasse und die behandelnde Ärztin/den behandelnden Arzt unverzüglich. Gleiches gilt, wenn nach initialer Aufnahme in die SAPV, im Rahmen eines anlassbezogenen (z.B. Befundverbesserung) oder regelmäßig, in der Regel 14-tägig, durchzuführenden Re-Assessments festgestellt wird, dass die besonderen Mittel der SAPV nicht mehr notwendig sind und die Mittel der Regelversorgung ausreichen.

## § 18
## Vorläufige Kostenzusage

(1) Die Krankenkasse übernimmt vom ärztlich festgelegten Leistungsbeginn an bis zu einer Entscheidung über die weitere Leistungserbringung die Kosten für die verordneten und vom Palliative-Care-Team erbrachten Leistungen entsprechend der Vergütung gemäß dieses Vertrages, wenn die ordnungsgemäß ausgefüllte Verordnung spätestens am dritten der Ausstellung folgenden Arbeitstag der Krankenkasse vorliegt. Sie informiert hierüber das Palliative-Care-Team.

(2) Soweit die verordneten Leistungen nicht oder nur zum Teil übernommen werden, informiert die Krankenkasse die Versicherten/den Versicherten oder eine ihm nahe stehende Person, das Palliative-Care-Team und die verordnende Ärztin/den verordnenden Arzt.

(3) Sofern die Erstverordnung verspätet eingereicht wird, werden die Kosten erst ab Eingangsdatum übernommen.

(4) Liegt der Krankenkasse innerhalb von 7 Arbeitstagen ab Verordnungsbeginn zusätzlich zur Erstverordnung kein Ergebnis über das SAPV-Eingangsassessment des qualifizierten Palliativmediziners vor, endet die vorläufige Kostenzusage mit Ablauf dieses Tages. Die Krankenkasse informiert den Versicherten oder eine ihm nahe stehende Person, den Leistungserbringer und den verordnenden Arzt.

## § 19
## Prüfung der Leistungsansprüche durch die Krankenkasse

(1) Der Krankenkasse obliegt die Überprüfung der leistungsrechtlichen Voraussetzungen der Verordnungsfähigkeit der SAPV. Besteht aus Sicht der Krankenkasse in Einzelfällen Anlass, die Notwendigkeit und Dauer der SAPV zu überprüfen, so kann sie vor Beauftragung des Medizinischen Dienstes der Krankenversicherung unter Angabe des Überprüfungsanlasses eine kurze Stellungnahme des Palliative-Care-Teams zur Frage der Versorgungs-/Pflegesituation der Palliativpatientin/des Palliativpatienten anfordern.

(2) Dem Medizinischen Dienst der Krankenversicherung sind auf Verlangen alle benötigten Auskünfte, die zur Beurteilung der Leistungspflicht der Krankenkassen benötigt werden, zu erteilen.

(3) Bestehen aus Sicht des Medizinischen Dienstes der Krankenversicherung Bedenken gegen den Fortbestand der leistungsrechtlichen Voraussetzungen bezüglich der (weiteren) Notwendigkeit und Dauer der SAPV, so werden diese gegenüber dem Palliative-Care-Team dargelegt und mit diesem erörtert. In einem solchen Fall wird das Palliative-Care-Team über das Ergebnis der Überprüfung und die daraus resultierende Entscheidung der Krankenkasse informiert und hat zu veranlassen, dass die gebotene Versorgung im Rahmen der allgemeinen Palliativversorgung sichergestellt wird.

## § 20
## Vergütung

(1) Die ärztlichen und pflegerischen bzw. kombinierten Leistungen im Rahmen der SAPV werden als Komplexpauschalen vergütet. Die Höhe der Vergütung richtet sich nach den in Anlage 5 aufgeführten Pauschalen.

(2) Der Vergütungsanspruch besteht nur für entsprechend den Vorgaben dieses Vertrages tatsächlich erbrachte und dokumentierte Vertragsleistungen. Für nicht vertragsmäßig erbrachte und auch für nicht dokumentierte Vertragsleistungen besteht weder ein Vergütungsanspruch gegenüber dem Kostenträger noch gegenüber der Versicherten/dem Versicherten.

(3) Leistungen, die wegen fehlender Mitwirkung des Versicherten nicht erbracht werden können, sind nicht abrechnungsfähig.

(4) Kommt das Palliative-Care-Team seiner Verpflichtung zum Nachweis der Fortbildung für einen Leistungserbringer des Teams (§ 14) nicht nach, erfolgen Vergütungsabschläge für die nach diesem Vertrag zu vergütenden Leistungen in Höhe von 10 v. H. für die ersten drei Monate, danach in Höhe von 25 v. H.

(5) Mit den für die Vertragsleistungen gemäß Anlage 5 des Vertrages vereinbarten Vergütungssätzen sind zugleich sämtliche Aufwendungen des Leistungserbringers abgegolten, die im unmittelbaren und mittelbaren Zusammenhang mit der jeweils vergüteten Vertragsleistung stehen.

(6) Dem Palliative-Care-Team sowie dessen Personal ist es untersagt, für Vertragsleistungen Zahlungen von dem Versicherten oder einem sonstigen Dritten zu fordern oder anzunehmen.

## § 21
## Leistungsnachweis

(1) Jede vom Palliative-Care-Team erbrachte ärztliche oder pflegerische Leistung ist unmittelbar nach ihrer Durchführung im Leistungsnachweis durch die Palliativ-Ärztin/den Palliativ-Arzt bzw. die ausführende Pflegefachkraft einzutragen und durch Handzeichen zu bestätigen. Maschinelle Eintragungen im Leistungsnachweis sowie Vordatierungen oder Globalbestätigungen, sind unzulässig. Gleiches gilt für Eintragungen im Leistungsnachweis vor Erbringung der Leistung. Der Leistungsnachweis hat die folgende Mindestinhalte aufzuweisen:

(a) Krankenversichertennummer,
(b) Name und Vorname der Patientin/des Patienten,
(c) Geburtsdatum der Patientin/des Patienten,
(d) Tag der Leistungserbringung,
(e) Uhrzeit (Beginn) der Leistungserbringung,
(f) Art der Leistung
(g) Monatlich eine Bestätigung durch Unterschrift eines Verantwortlichen des Palliative-Care-Teams,
(h) nach erfolgter Leistungserbringung einmal monatlich Datum und Unterschrift der Patientin/des Patienten, des gesetzlichen Vertreters oder Bevollmächtigten sowie
(i) Institutionskennzeichen, Name und Anschrift des Palliative-Care-Teams.

Eine Durchschrift des Leistungsnachweises hat auf Wunsch bei der Patientin/beim Patienten zu verbleiben.

(2) Die Leistungsnachweise haben dem Muster nach Anlage 3 zu entsprechen.

## § 22
## Abrechnung

(1) Die nach diesem Vertrag zu vergütenden Leistungen sind vom Leistungserbringer oder Palliative-Care-Team grundsätzlich im Folgemonat mit der Krankenkasse abzurechnen. Die Genehmigung der Krankenkasse - soweit hierauf nicht verzichtet wird - und der Leistungsnachweis (§ 21) sind beizufügen. Abgerechnet werden dürfen nur genehmigte, tatsächlich erbrachte und dokumentierte Leistungen.

(2) Das Rechnungsblatt muss folgende Mindestinhalte aufweisen:

(a) Krankenversichertennummer,
(b) Name und Vorname der Patientin/des Patienten,
(c) Geburtsdatum der Patientin/des Patienten,
(d) Anschrift der Patientin/des Patienten,
(e) Leistungszeitraum (von/bis),
(f) Art der Leistung (z. B. Koordination, Beratung etc.),
(g) Einzelpreis der Leistung,
(h) Anzahl der Leistung,
(i) Gesamtbetrag,
(j) Rechnungsnummer,
(k) Rechnungsdatum,
(l) Institutionskennzeichen des Leistungserbringers sowie
(m) ) Institutionskennzeichen des Zahlungsempfängers, sofern
(n) vom Leistungserbringer abweichend.

(3) Für den Abrechnungsverkehr ist das für das Palliative-Care-Team maßgebende Institutionskennzeichen (IK) zu verwenden, das von der Arbeitsgemeinschaft Institutionskennzeichen (SVI) beim Hauptverband der gewerblichen Berufsgenossenschaften e. V., Alte Heerstraße 111, 53757 St. Augustin, vergeben wird. Dies gilt auch, wenn die Abrechnung über eine Abrechnungsstelle/Verrechnungsstelle erfolgt.

(4) Forderungen der Krankenkasse gegen das Palliative-Care-Team können gegenüber demselben aufgerechnet werden.

(5) Rechnungen, die den Anforderungen nach den Absätzen 1 bis 4 nicht entsprechen, können von der Krankenkasse berichtigt oder unter Angabe von Gründen zur Berichtigung innerhalb von 20 Arbeitstagen nach Eingang zurückgegeben werden.

(6) Sofern die DTA Richtlinien nach § 302 SGB V eine Abrechnung für die Palliative-Care-Teams ermöglichen bzw. vorsehen, ist dieses Verfahren anzuwenden. Die Richtlinien gem. § 302 Abs. 2 SGB V sind zu beachten. Die technischen Voraussetzungen sind hierfür durch das Palliative-Care-Team zu schaffen.

## § 23
## Rechnungslegung durch Abrechnungsstellen

(1) Sofern die Abrechnung vom Palliative-Care-Team einer Abrechnungsstelle/Verrechnungsstelle übertragen werden soll, hat dieses unter besonderer Berücksichtigung der von ihr getroffenen technischen und organisatorischen Maßnahmen zur Sicherstellung der Maßgaben dieses Vertrages und der einschlägigen datenschutzrechtlichen Bestimmungen die Abrechnungsstelle/Verrechnungsstelle auszuwählen.

(2) Das Palliative-Care-Team ist verpflichtet, von allen Versicherten schriftliche Einwilligungserklärungen für die Datenweitergabe an die Abrechnungsstelle einzuholen. Die Einwilligungserklärungen sind der Krankenkasse und der Abrechnungsstelle auf Verlangen vorzulegen.

(3) Die Abtretung ist vier Wochen vorher anzuzeigen. Zahlungen an eine durch das Palliative-Care-Team ermächtigte Abrechnungsstelle/Verrechnungsstelle setzen voraus, dass der zuständigen Krankenkasse der Vertrag und eine Ermächtigungserklärung des Palliative-Care-Teams vorliegen. Eine weitere Ermächtigungserklärung setzt den Widerruf der zuvor erteilten Ermächtigungserklärung voraus.

(4) Das Palliative-Care-Team verpflichtet sich, seine Forderungen gegen die Krankenkassen nicht an Dritte abzutreten, wenn und soweit die Krankenkassen gegen ihn Ansprüche wegen rückständiger Gesamtsozialversicherungsbeiträge, Überzahlungen (Rückforderungsansprüche) oder sonstiger Forderungen hat. Zur Sicherung derartiger Forderungen besteht zugunsten der Krankenkassen ein Abtretungsausschluss nach § 399 BGB. Die Vertragspartner gehen davon aus, dass die Krankenkassen aufgrund der Bestimmungen des § 354 a HGB auch im Fall einer verbotswidrigen Abtretung berechtigt sind, gegenüber dem Abrechnungszentrum die Zahlung zu verweigern und mit Ihren Ansprüchen gegen das Palliative-Care-Team aufzurechnen.

(5) Zahlungen an eine Abrechnungsstelle/Verrechnungsstelle erfolgen mit schuldbefreiender Wirkung für die Krankenkasse, wenn die abrechnende Stelle Originalabrechnungsunterlagen einreicht, es sei denn, der zahlungspflichtigen Krankenkasse liegt bei Eingang der Originalabrechnungsunterlagen ein schriftlicher Widerruf des Palliative-Care-Teams vor. Die schuldbefreiende Wirkung tritt auch dann ein, wenn die Rechtsbeziehung zwischen der Abrechnungsstelle/Verrechnungsstelle und dem Palliative-Care-Team mit einem Rechtsmangel behaftet ist. Schädigt die Abrechnungsstelle anlässlich der Abrechnung die Krankenkasse, so haften Palliative-Care-Team und Abrechnungsstelle/Verrechnungsstelle gesamtschuldnerisch.

(6) Für Abrechnungsstellen/Verrechnungsstellen gelten die §§ 22 und 23 entsprechend. Mit etwaigen Abrechnungsstellen/Verrechnungsstellen wird das Palliative-Care-Team diesbezügliche Absprachen treffen.

## § 24
## Zahlungsfrist

(1) Die Rechnungen sind - vorbehaltlich einer abschließenden Rechnungsprüfung - grundsätzlich innerhalb von 15 Arbeitstagen nach Eingang bei der zuständigen Krankenkasse bzw. dem genannten Rechenzentrum zu bezahlen. Bei Rechnungskürzungen gilt dies nur für den unstreitigen Rechnungsbetrag. Als Zahltag gilt der Tag der Überweisung oder der Tag der Übergabe des Überweisungsauftrages an ein Geldinstitut.

(2) Die Fälligkeit für die vertraglich geschuldete Vergütung tritt nach der gem. Abs. 1 vereinbarten Zahlungsfrist nur bei vorschriftsmäßiger Rechnungslegung (§§ 22 bis 24) ein.

## § 25
## Ausschlussfrist

Vertragsleistungen, für die entgegen der hierzu in § 22 Absatz 1 Satz 1 vereinbarten Frist eine Rechnungslegung verspätet erfolgt, können nur in begründeten Ausnahmefällen abgerechnet werden. Diese Ausnahmefälle sind nur abrechnungsfähig, wenn gegenüber der Krankenkasse innerhalb einer Ausschlussfrist von zwölf Monaten ab dem Zeitpunkt der Leistungserbringung eine Rechnungslegung erfolgt ist.

## § 26
## Einflussnahme-, Werbungs- und Vermittlungsverbot

(1) Eine Beeinflussung der verordnenden Ärztin/des verordnenden Arztes mit dem Ziel, diesen zu unwirtschaftlichen Verordnungen zu veranlassen, ist unzulässig.

(2) Die Gewährung oder Annahme von Zuwendungen gleich welcher Art (z. B. Prämien, Provisionen o. ä.) für die Zuweisung von Versicherten an das Palliative-Care-Team ist unzulässig.

(3) Soweit Versicherte gezielt zur Inanspruchnahme dieser Leistungen aufgefordert werden, ist Werbung für die im Rahmen dieses Vertrages zur Verfügung stehenden Leistungen nicht zulässig. Das Palliative-Care-Team ist jedoch berechtigt, die Versicherte/den Versicherten an die Krankenkasse zwecks Beratung über die Möglichkeit der Inanspruchnahme von Leistungen nach diesem Vertrag zu verweisen.

(4) Die Vermittlung an Dritte gegen Entgelt oder zur Erlangung geldwerter Vorteile ist nicht zulässig. Vermittlung im Sinne dieser Bestimmung ist auch die Weitergabe von in eigenem Namen angenommenen Pflegeaufträgen an Dritte (z. B. freie Mitarbeiter) gegen Kostenerstattung.

## § 27
## Datenschutz, Schweigepflicht

(1) Das Palliative-Care-Team ist verpflichtet, die Bestimmungen über den Schutz der Sozialdaten (SGB X, 2. Kapitel) zu beachten, insbesondere personenbezogene Daten nur zur Erfüllung der sich aus diesem Vertrag ergebenden Aufgaben zu erheben, zu verarbeiten oder zu nutzen.

(2) Das Palliative-Care-Team unterliegt hinsichtlich der Person des Versicherten und dessen Krankheiten der Schweigepflicht. Ausgenommen hiervon sind Angaben gegenüber der leistungspflichtigen Krankenkasse und/oder dem Medizinischen Dienst Krankenversicherung, soweit sie zur Erfüllung der gesetzlichen Aufgaben der Krankenkassen erforderlich sind.

(3) Das Palliative-Care-Team hat die im Team tätigen Mitarbeiterinnen/Mitarbeiter zur Beachtung der Datenschutzbestimmungen und der Schweigepflicht zu verpflichten. Die Verpflichtung ist zu dokumentieren.

(4) Die Weitergabe und Nutzung der die Versicherte/den Versicherten betreffenden Behandlungsdaten und Befunde bedürfen der schriftlichen Einwilligung der Versicherten/des Versicherten.

## § 28
## Statistik

(1) Das Palliative-Care-Team ist verpflichtet, an Auswertungen mitzuwirken. Hierzu übermittelt es halbjährlich die Entwicklungen den Krankenkassen.

(2) Die Auswertungen beinhalten u. a. folgende Kennzahlen:

a) Fallzahlen (Anzahl der behandelten Patientinnen/Patienten),
b) Kassenzugehörigkeit
c) Diagnosen,
d) Versorgungsbereich,
e) Zahl der Vertragsärzte,
f) Zahl der Kooperationspartner,
g) Zahl an der Versorgung beteiligter Angehöriger,
h) Zahl der Ehrenamtlichen,
i) prozentualer Anteil von Patientinnen/Patienten, bei denen Ehrenamtliche eingesetzt werden,
j) Krankenhauseinweisungen,
k) Überlebenszeit,

l) prozentualer Anteil von Patientinnen/Patienten, die in der Häuslichkeit verstorben sind,
m) durchschnittliche Betreuungsdauer und durchschnittliche Einsatzzeit
n) Einstufungen der Patientinnen/Patienten in Pflegestufen nach dem SGB XI.

(3) Das Palliative-Care-Team erhält das Ergebnis der Auswertung.

## § 29
## Vertrags-/Gesetzesverstöße

(1) Erfüllt das Palliative-Care-Team seine gesetzlichen und/oder vertraglichen Pflichten nicht in der gebotenen Weise, entscheiden die Krankenkassen gemeinsam oder einzeln über geeignete Maßnahmen. Als solche kommen in Betracht:

a) Verweis
b) Abmahnung
c) Vertragsstrafe
d) Außerordentliche Kündigung des Vertrages.

(2) Die Vertragsstrafe wird durch die Krankenkassen gemeinsam oder einzeln festgesetzt, und zwar insbesondere bei wiederholt fehlerhaften Abrechnungen durch das Palliative-Care-Team in Höhe von bis zu 10.000,00 EUR. Die vorstehende Vertragsstrafe gilt für jeden einzelnen Vertragsverstoß. Der Krankenkasse/ den Krankenkassen bleibt es unbenommen, daneben einen weiteren Schadensersatz geltend zu machen bzw. die Kündigungsrechte gem. § 24 wahrzunehmen.

(3) Zur Aufklärung des Sachverhalts hat das Palliative-Care-Team der Krankenkasse/ den Krankenkassen die erforderlichen Unterlagen zu übersenden bzw. die Einsichtnahme und ggf. Ablichtung in seinen Geschäftsräumen zu gewähren.

(4) Verstöße gegen die gesetzlichen und/oder vertraglichen Bestimmungen sind dem Palliative-Care-Team schriftlich mitzuteilen. Ihm ist die Gelegenheit zu einer Stellungnahme innerhalb angemessener Frist zu geben. Die Vertragspartner bemühen sich, eventuelle Meinungsverschiedenheiten über Vertragsverstöße gemeinsam mit dem Ziel einer gütlichen Einigung zu klären.

(5) Die Entscheidung wird dem Palliative-Care-Team schriftlich mitgeteilt.

(6) Unabhängig von den Maßnahmen nach Absatz 1 ist der durch die Vertragsverletzung verursachte Schaden durch das Palliative-Care-Team zu ersetzen.

## § 30
## Außerordentliche Kündigung

(1) Der Vertrag kann von den vertragsschließenden Krankenkassen gemeinsam oder einzeln gegenüber dem Palliative-Care-Team ohne Einhaltung einer Kündigungsfrist gekündigt werden, wenn ein Vertragspartner seine gesetzlichen und/oder vertraglichen Pflichten gegenüber den Versicherten oder der Krankenkasse derart gröblich verletzt, dass ein Festhalten an diesem Vertrag nicht zumutbar ist. Eine gröbliche Pflichtverletzung in diesem Sinne liegt insbesondere vor bei:

- Nichterfüllung der organisatorischen, personellen oder sächlichen Voraussetzungen gemäß § 7 über einen Zeitraum von mehr als drei Monaten; für den Nachweis der Geeignetheit der Pflegefachkraft gilt eine Frist von 9 Monaten, vgl. § 9 Abs. 1 ,
- Wiederholte Leistungserbringung durch dafür fachlich nicht qualifiziertes Personal entgegen § 7,
- Schwerwiegende pflegebedingte Qualitätsmängel, die medizinisch oder pflegerisch festgestellt werden,
- Abrechnung von Leistungen entgegen § 20 Abs. 2,
- Forderungen von Versicherten entgegen § 20 Abs. 6,
- Verstoß gegen § 27,
- Kein Nachweis, dass die Fortbildung innerhalb der in § 14 Abs. 2 geregelten Frist nachgeholt wurde.

(2) Ein Recht zur außerordentlichen Kündigung besteht ferner bei Vertragsverstößen des Palliative-Care-Teams oder eines Leistungserbringer des Palliative-Care-Teams, die zur außerordentlichen Kündigung eines mit diesem Leistungserbringer bestehenden Versorgungsvertrages berechtigen, vgl. z.B. § 74 SGB XI.

(3) Vor Ausspruch der außerordentlichen Kündigung i. S. d. Absatz 1 teilt die Krankenkasse(n) dem Palliative-Care-Team ihre Kündigungsabsicht mit und gibt/geben dem Palliative-Care-Team Gelegenheit, binnen drei Wochen nach Zugang dieser Mitteilung schriftlich zu den erhobenen Vorwürfen Stellung zu nehmen. Entscheidend zur Fristwahrung ist der Zugang der Stellungnahme des Palliative-Care-Teams bei der/den Krankenkasse(n).

(4) Die in Absatz 3 geregelte Verpflichtung besteht nicht, wenn das Wohl der Versicherten/des Versicherten der Krankenkasse(n) unverzügliches Handeln der Krankenkasse(n) erfordert, insbesondere wenn die Versorgung der Versicherten/des Versicherten mit Leistungen nach diesem Vertrag durch das Palliative-Care-Team nicht (mehr) sicher gestellt ist und dadurch Leben oder Gesundheit von Versicherten gefährdet ist (Gefahr im Verzuge).

(5) Die Kündigung muss schriftlich erfolgen.

## § 31
## In-Kraft-Treten, Kündigung

(1) Dieser Vertrag tritt einschließlich seiner Anlagen am **1. Dezember 2009** in Kraft.

(2) Der Vertrag kann zum Ende eines Kalenderhalbjahres mit dreimonatiger Frist durch eingeschriebenen Brief von und gegenüber jedem Vertragspartner einzeln gekündigt werden. Die Kündigung von oder gegenüber einem Vertragspartner lässt die Wirksamkeit des Vertrages im Verhältnis zu den übrigen beteiligten Vertragspartnern unberührt.

(3) Abweichend von Abs. 2 gilt für die Vergütungsvereinbarung nach Anlage 5 die dort vorgesehene besondere Kündigungsregelung.

## § 32
## Änderungen und Ergänzungen

Änderungen oder Ergänzungen des Vertrages bedürfen der Schriftform.

## § 33
## Salvatorische Klausel

(1) Sollte eine Bestimmung des Vertrages unwirksam sein oder werden oder der Vertrag unvollständig sein, so wird der Vertrag in seinem übrigen Inhalt davon nicht berührt. Die unwirksame Bestimmung wird durch eine solche Bestimmung ersetzt, die dem Sinn und Zweck der unwirksamen Bestimmung in rechtswirksamer Weise und wirtschaftlich am nächsten kommt. Gleiches gilt für etwaige Vertragslücken.

(2) Im Übrigen gilt § 59 SGB X.

Hamburg, Düsseldorf, den

PALLIATIVTEAM

AOK Rheinland/Hamburg
- Die Gesundheitskasse,

Knappschaft,
Regionaldirektion Hamburg

BKK-Landesverband NORD,

Verband der Ersatzkassen e.V. (vdek),
Der Leiter der vdek-Landesvertretung
Hamburg

IKK classic

Protokollnotizen

**zu dem Vertrag über die spezialisierte ambulante palliativmedizinische sowie palliativpflegerische Versorgung in Hamburg gemäß § 132 d SGB V i. V. m. § 37 b SGB V**

Protokollnotiz 1 zu § 7 II. Personelle Voraussetzungen Abs. 1

Sind die im Palliativteam tätigen Ärzte zugleich vertragsärztlich tätig, stellen sie durch geeignete wechselseitige Vertretung sicher, dass sie Ihrem vorrangigen Versorgungsauftrag in der vertragsärztlichen Versorgung jederzeit gerecht werden.

Protokollnotiz 2 zu § 7 II. Personelle Voraussetzungen Abs. 1 a)

Ärztinnen und Ärzte gelten ebenfalls als geeignet nach diesem Vertrag, wenn die anerkannte Zusatzweiterbildung Palliativmedizin spätestens bis 30.4.2009 begonnen wurde und voraussichtlich bis zum 31.12.2009 abgeschlossen sein wird.

Protokollnotiz 3 zu § 7 II. Personelle Voraussetzungen Abs. 1 b)

Pflegefachkräfte gelten ebenfalls als geeignet nach diesem Vertrag, wenn die Palliativ-Care-Weiterbildungsmaßnahme im Umfang von mindestens 160 Stunden spätestens bis 30.4.2009 begonnen wurde und voraussichtlich bis zum 31.12.2009 abgeschlossen sein wird. Im Rahmen des Bestandsschutzes gilt dieses auch für dreijährig examinierte Altenpfleger/Altenpflegerinnen, die ihre Berechtigung zur Führung der Berufsbezeichnung nach den jeweils geltenden gesetzlichen Bestimmungen vor Inkrafttreten / vor Umsetzung des Gesetzes über die Berufe in der Altenpflege vom 25.08.2003 erworben haben.

Protokollnotiz  4 zu § 7 II. Personelle Voraussetzungen in Verbindung mit Anlage 5 Vergütungen Punkt III. Vollversorgung

Bei einer Vollversorgung können durch das PCT keine weiteren Leistungen nach dem SGB V, insbesondere keine Leistungen der Häuslichen Krankenpflege gemäß den Verträgen nach § 132 a SGB V abgerechnet werden. Soweit nach ärztlicher Anordnung/ Verordnung entsprechende Leistungen durch das PCT erbracht  werden, kann hierzu nach der folgenden Maßgabe qualifiziertes Personal, d.h. 3-jährig examinierte Pflegefachkräfte eingesetzt werden:.

Maßnahmen nach § 37 .1 SGB V und § 37.2 SGB V entsprechend den HKP- Richtlinien des Gemeinsamen Bundesausschusses, dürfen durch das PCT im Rahmen der Vollversorgung nicht abgerechnet werden, können aber durch qualifiziertes weitergebildetes Pflegefachpersonal, das noch keine Palliative Care Weiterbildung abgeschlossen hat, am Patienten erbracht werden.

Hierbei ist es für jede am PCT beteiligte Pflegeeinrichtung möglich, Pflegefachkräften, die noch nicht über eine Palliative Weiterbildung verfügen in einem Verhältnis von 1:1 zu Pflegefachkräften nach § 7 II. Abs. 1 b) einzusetzen.  Diese Mitarbeiter dürfen ausschließlich Leistungen der HKP und nicht der SAPV erbringen.
Beispielhaft kann ein Dienst, der für das PCT 4 Palliative Fachpflegekräfte zur Verfügung stellt, weitere 4 Pflegefachkräfte benennen.
Die verbindlich zu benennenden Pflegefachkräfte des jeweiligen Dienstes, sind damit assoziierte Mitglieder des PCT und haben an den Team- und Fallbesprechungen teilzunehmen.
Für sie erfolgt die Anmeldung zu der Palliativ –Care-Weiterbildung von mindestens 160 Stunden, die möglichst bis zum 30.06.2011 abzuschließen ist.

Protokollnotiz 5 zu § 9 Abs. 1
In begründeten Ausnahmefällen kann bezüglich der geeigneten Pflegefachkräfte nach § 7 II. Abs. 1 b) von der Beschäftigung bei einem Leistungserbringer nach § 132 a Abs. 2 SGB V abgesehen werden. Voraussetzung hierfür ist, dass das Palliative-Care-Team im Rahmen des Konzepts nach § 6 Abs. 3  Aussagen zur Teambildung bezüglich der palliativ-pflegerischen Versorgung trifft. Darüber hinaus ist sicherzustellen, dass im Rahmen der fachlichen Leitung nach § 9 Abs. 2 für die palliativ-pflegerischen Leistungen eine Leitungsqualifikation  (Umfang von mindestens 460 Std.) in Vollzeit vorgehalten wird.

Protokollnotiz 6 zu § 18 Abs. 3
Die Vertragspartner stimmen überein, dass auch Folgeverordnungen nur in absoluten Ausnahmefällen verspätet eingereicht werden sollen. Zur Sicherstellung der Kontinuität der Versorgung wir von Sanktionen abgesehen.

Protokollnotiz zu der Kooperationsvereinbarung mit den Pflegeeinrichtungen  DRK-Sozialstation Eimsbüttel-Nord gGmbH und
DRK- Sozialstation Lokstedt/Stellingen gGmbH
DAS PALLIATIVTEAM GbR erklärt die Absicht, den beiden Kooperationspartnern noch im Laufe des Jahres 2010 den Beitritt zur bestehenden GbR zu ermöglichen.

Vertrag über die Erbringung

# Spezialisierter ambulanter Palliativversorgung (SAPV) gemäß § 132 d i. V. m. § 37 b SGB V

zwischen

den Ersatzkassen

Barmer Ersatzkasse
Techniker Krankenkasse
Deutsche Angestellten-Krankenkasse
KKH-Allianz
Gmünder ErsatzKasse - GEK
HEK - Hanseatische Krankenkasse
Hamburg Münchener Krankenkasse
hkk

gemeinsamer Bevollmächtigter mit Abschlussbefugnis:
Verband der Ersatzkassen e. V. (vdek),
vertreten durch den Leiter der vdek-Landesvertretung Hessen

im Folgenden der „vdek“ genannt

und

*Name des Palliative Care Teams, Rechtsform, Sitz*
*vertreten durch ...*

im Folgenden „Leistungserbringer“ genannt

<u>Institutionskennzeichen:</u>

## Präambel

Die spezialisierte ambulante Palliativversorgung (SAPV) dient dem Ziel, die Lebensqualität und die Selbstbestimmung schwerstkranker Menschen zu erhalten, zu fördern und zu verbessern und ihnen ein menschenwürdiges Leben bis zum Tod in ihrer häuslichen Umgebung oder in stationären Pflegeeinrichtungen, Einrichtungen der Kinder- und Jugendhilfe oder in Einrichtungen der Eingliederungshilfe zu ermöglichen. Versorgungsunsicherheiten, die zu unnötigen und die Versicherten stark belastenden Krankenhauseinweisungen führen, sollen durch die in der SAPV vorgehaltenen besonderen Fachkenntnisse vermieden werden. Symptome und Leiden sollen durch SAPV einzelfallgerecht und nach den Wünschen der Versicherten durch palliativmedizinisch-palliativpflegerische Maßnahmen gelindert oder behoben werden.

Die Versorgung des Versicherten / der Versicherten bedarf einer besonderen, multiprofessionellen SAPV-Fachkompetenz, erbracht durch ein Palliative-Care-Team (PCT). Die SAPV muss ausreichend und zweckmäßig sein. Sie orientiert sich jederzeit flexibel an dem individuellen Hilfebedarf des Versicherten, darf allerdings das Maß des Notwendigen nicht überschreiten und muss wirtschaftlich erbracht werden.

Der Versicherte / die Versicherte und auch sein / ihr soziales Umfeld (seine / ihre vertrauten Personen) sollen in die Versorgung mit einbezogen und über die Krankheit und den Krankheitsverlauf aufgeklärt werden. Die Arbeit des PCT integriert dabei auch die ehrenamtlichen Kräfte der ambulanten Hospizdienste.

Anspruch auf die Leistung der SAPV und damit einer besonderen (aufwändigen) Versorgung besteht, wenn die anderweitigen ambulanten Versorgungsformen nicht ausreichen, Die SAPV ergänzt das bestehende Versorgungsangebot, insbesondere das der Vertragsärzte, Krankenhäuser und Pflegedienste.

Die Vertragsparteien stimmen darin überein, dass die SAPV für Kinder und Jugendliche einer weiteren ergänzenden Vereinbarung bedarf.

## § 1

## Vertragsziele und Vertragsgrundlagen

(1) Dieser Vertrag dient der Sicherstellung der bedarfsgerechten individuellen Versorgung des/der anspruchsberechtigten Versicherten mit SAPV in dem im

Versorgungskonzept ausgewiesenen Versorgungsgebiet durch den Leistungserbringer.
Die Bedarfsgerechtigkeit einer Region bedeutet u. a., dass eine SAPV-Versorgung qualitativ gleichwertig bei unterschiedlichen Bevölkerungsdichten zu erfolgen hat.

(2) Bei der SAPV handelt es sich um eine Versorgungsleistung, die von multiprofessionellen Palliative Care Teams (PCT) erbracht wird. Es handelt sich um eine Aufgabe, die nur dann bedarfsgerecht erbracht werden kann, wenn der Leistungserbringer Teil einer multiprofessionell vernetzten Versorgungsstruktur im regionalen Gesundheits- und Sozialsystem ist. Das PCT arbeitet mit den an der Regelversorgung beteiligten Leistungserbringern unterschiedlicher Berufsgruppen (z. B. Haus-/Fachärzten, Pflegediensten, Seelsorgern, Sozialarbeitern, Physiotherapeuten, Psychologen und Ehrenamtlichen sowie ambulanten Hospizdiensten, insbesondere den gem. § 39a Abs.2 SGB V geförderten) in Form einer Netzwerkfunktion (vgl. § 3 Abs. 3) zusammen.

(3) Die palliativmedizinisch-palliativpflegerische Versorgung soll durch abgestimmte Behandlungs- und Kommunikationsstrukturen des PCT mit seinen Netzwerkpartnern erfolgen.

(4) Grundlagen dieses Vertrages sind:

- die „Richtlinie des Gemeinsamen Bundesausschusses zur Verordnung von spezialisierter ambulanter Palliativversorgung“ (Spezialisierte Ambulante Palliativversorgungs-Richtlinie/SAPV-RL) vom 20. Dezember 2007 und

- die „Gemeinsamen Empfehlungen der Spitzenverbände der Krankenkassen nach § 132 d Abs. 2 SGB V für die spezialisierte ambulante Palliativversorgung“ vom 23.06.2008

in ihrer jeweiligen aktuellen Fassung.

## § 2

## Anspruchsvoraussetzungen

(1) Der/die Versicherte hat gemäß SAPV-RL Anspruch auf SAPV, wenn

1. er/sie an einer nicht heilbaren, fortschreitenden und so weit fortgeschrittenen Erkrankung leidet, dass dadurch seine/ihre Lebenserwartung auf Tage, Wochen oder Monate begrenzt ist,

2. er/sie unter Berücksichtigung der in der SAPV-RL genannten Ziele eine besonders aufwändige Versorgung (§ 4 SAPV-RL) benötigt, die nach den medizinischen und pflegerischen Erfordernissen in der häuslichen Umgebung (ambulant), in stationären Einrichtungen gemäß § 43 SGB XI i.V.m. § 72 SGB XI, sowie in Einrichtungen der Kinder- und Jugendhilfe oder in Einrichtungen der Eingliederungshilfe erbracht werden kann.

3. er/sie unter Berücksichtigung der in der SAPV-RL genannten Ziele eine besonders aufwändige Versorgung (§ 4 SAPV-RL) benötigt, die nach den medizinischen und pflegerischen Erfordernissen im stationären Hospiz erbracht werden kann. Diese Leistungserbringung der spezialisierten Palliativversorgung im stationären Hospiz mittels der SAPV gilt vorbehaltlich einer dazu auf Bundesebene anstehenden abschließenden Entscheidung.

(2) Liegt ein besonders aufwändiger Versorgungsbedarf nicht vor, besteht kein Anspruch auf SAPV.

(3) Die individuelle Versorgung des/der Versicherten gem. SAPV-RL wird nicht unterbrochen, sofern der/die Versicherte einzelne Maßnahmen der Versorgung ablehnt. Der Anspruch auf Versorgung mit SAPV bleibt hierdurch unberührt.

(4) Der/die Versicherte ist in der Wahl des Leistungserbringers frei.

## § 3

## Versorgungskonzept

(1) Der Leistungserbringer ist ein Palliative-Care-Team (PCT) und versorgt auf der Grundlage eines verbindlichen, strukturierten und schriftlich dargelegten Versorgungskonzeptes.

(2) Das Versorgungskonzept formuliert alle palliativmedizinisch-palliativ-pflegerischen Maßnahmen klar und eindeutig unter Beachtung von Maßnahmen zur Qualitätssicherung. Es muss darüber hinaus verdeutlichen, wie eine qualifizierte Leistungserbringung zu erfolgen hat, damit die in § 1 dieses Vertrages genannten Vertragsziele erreicht werden können.

(3) Die regionalen Netzwerkstrukturen sind zu beschreiben, in die sich der Leistungserbringer als PCT im Rahmen der Vernetzung integriert.

(4) Das Versorgungskonzept ist dem vdek über die Landesarbeitsgemeinschaft für Palliativversorgung Hessen (LAPH) zur Verfügung zu stellen. Die Vertragsziele gemäß § 1 dieses Vertrages werden mit dem Versorgungskonzept umgesetzt und erfüllt. Der Leistungserbringer stellt die SAPV-Versorgung für sein Versorgungsgebiet sicher.

(5) Das Versorgungskonzept beinhaltet insbesondere folgende Aspekte:

1. Standort des Leistungserbringers,
2. Versorgungsgebiet,
3. Versorgungsprozess,
4. Teammitglieder des PCT,
5. Personelle Zuständig- und Verantwortlichkeiten im PCT (u. a. Weisungsbefugnisse, Weisungsgebundenheit, u. a.),
6. Qualifikationsnachweise der Mitarbeiter im PCT gemäß § 10,
7. sächliche Ausstattung gemäß § 12,
8. Qualitätssicherungsmaßnahmen gemäß § 17,
9. Konzept zur Sicherstellung der reibungslosen Versorgung mit Arznei-, Heil- und Hilfsmitteln, unter Maßgabe der regulären Verfügbarkeit von Verordnungsblättern (Rezepten)
10. Konzept zur Sicherstellung der 24-Stunden-Rufbereitschaft/Einsatzbereitschaft,
11. Fallzahleinschätzung.
12. Kooperationsvereinbarungen des PCT mit im Rahmen des Netzwerkes (vgl. § 1 Abs. 3 und § 3 Abs. 3) mit an der Versorgung Beteiligten im Versorgungsgebiet (z. B. ambulante Hospizdienste, Seelsorge und Sozialarbeit) zur Sicherung der psychosozialen Unterstützung,

(6) Die Leistungserbringung ist durch den Leistungserbringer in Form geeigneter Maßnahmen unter Einsatz entsprechender personeller und sachlicher Mittel für die Dauer des Vertrages sicherzustellen. Die Ersatzkassen sind umgehend zu informieren, wenn die Funktionsfähigkeit des Leistungserbringers nicht mehr besteht.

(7) Der Leistungserbringer ist weisungsunabhängig.

(8) Die Personalstärke eines Teams bestimmt sich u. a. durch die Anforderungen zur Erbringung ärztlicher und pflegerischer Leistungen, der 24-Stunden-Erreichbarkeit für betreute Versicherte, der qualifizierten Vertretung in Urlaubs- und Krankheitszeiten und der regionalen strukturellen Gegebenheiten.

(9) Der Leistungserbringer trägt die alleinige Verantwortung für das Versorgungskonzept.

## § 4

## Anzeigepflichten / Mitteilungspflichten

(1) Der Leistungserbringer ist verpflichtet, dem vdek anstehende Änderungen wesentlicher Umstände im Versorgungskonzept schriftlich innerhalb von 4 Wochen mitzuteilen. Dies sind insbesondere:

a. jede wesentliche Änderung der betrieblichen/organisatorischen Gegebenheiten.
b. Änderungen von Kooperationsvereinbarungen des PCT mit Netzwerkpartnern (Neuabschlüsse, Kündigung, inhaltliche Änderungen).
c. eingeleitete polizeiliche oder staatsanwaltliche Ermittlungsverfahren gegen den Leistungserbringer.

(2) Abweichend davon sind personelle Änderungen in Bezug auf die palliativmedizinische sowie palliativpflegerische Qualifikation umgehend mitzuteilen.

(3) Der vdek ist berechtigt, bei begründetem Anlass weitere Unterlagen zum Nachweis der organisatorischen und/oder personellen Voraussetzungen zur Einsichtnahme zu verlangen. Der Leistungserbringer ist verpflichtet, diese zur Verfügung zu stellen.

## § 5

## Verordnung / Genehmigung der SAPV

(1) Art, Umfang und Dauer der vom Leistungserbringer zu erbringenden Leistungen ergeben sich aus der von der Ersatzkasse genehmigten Verordnung der

behandelnden Vertragsärztin/des behandelnden Vertragsarztes oder der Krankenhausärztin/des Krankenhausarztes.

(2) Die Verordnung ist nicht übertragbar. Sie gilt nur für die Person, für die sie ausgestellt ist. Änderungen und Ergänzungen bedürfen der Unterschrift der/des verordnenden Vertragsärztin/Vertragsarztes.

(3) Leistungen der SAPV werden von der Ersatzkasse nur genehmigt, wenn eine ordnungsgemäß ausgefüllte ärztliche Verordnung gemäß der zwischen den Spitzenverbänden der Krankenkassen und der Kassenärztlichen Bundesvereinigung geschlossenen Vereinbarung (Anlage 3) per Telefax vorgelegt wird. Der/die Versicherte oder sein/ihr gesetzlicher Vertreter hat die Genehmigung der Verordnung schriftlich auf einem gesonderten Formular der Vereinbarung zu beantragen.

(4) Die Unterschrift durch den Leistungserbringer bzw. dessen Mitarbeiter ist ausgeschlossen. Kann die Unterschrift nicht erbracht werden, weil der Versicherte bzw. sein gesetzlicher Vertreter nicht in der Lage ist zu unterschreiben, bleibt das Unterschriftsfeld auf der Verordnung frei. Der Leistungserbringer gibt dort oder auf einer Anlage zur Verordnung die Gründe hierfür (z.B. Unzumutbarkeit) an.

(5) Die SAPV-RL ist für die Verordnung sowie für die Genehmigung durch die Ersatzkasse maßgeblich. Dort nicht aufgeführte Inhalte bedürfen einer nachvollziehbaren Begründung, damit sie von den Ersatzkassen genehmigt werden können.

(6) Der verordnende Arzt/die verordnende Ärztin legt nach aktuellem Versorgungsbedarf die Dauer der Verordnung fest. Die Krankenhausärztin/der Krankenhausarzt kann Leistungen in der Regel jedoch längsten für 7 Tage verordnen. Sollte eine Versorgung für einen längeren Zeitraum als 7 Tage erforderlich sein, ist durch das PCT rechtzeitig eine Folgeverordnung für den Zeitraum über den 7. Tag hinaus bei der zuständigen Krankenkasse einzureichen.

(7) Die Verordnung für einen zurückliegenden Zeitraum ist nicht zulässig.

(8) Der Leistungserbringer prüft im Rahmen des Eingangsassessments innerhalb von 3 Arbeitstagen nach Erhalt der Verordnung die Notwendigkeit und Eignung der besonderen Mittel der SAPV. Sofern für eine bedarfsgerechte Versorgung die Mittel der Regelversorgung ausreichen, ist die Ersatzkasse hierüber innerhalb

von 2 Werktagen zu informieren. Neben der Ersatzkasse sind auch der/die Versicherte und der Erstverordner über die Ergebnisse des Assessments zu informieren.

(9) Kann die Durchführung der verordneten Leistung nicht mehr erfolgen, z. B. weil der/die Versicherte in ein Krankenhaus eingewiesen wurde, informiert der Leistungserbringer die zuständige Ersatzkasse und den behandelnden Arzt innerhalb von 2 Werktagen. Gleiches gilt, wenn nach initialer Aufnahme in die SAPV, im Rahmen eines anlassbezogenen (z. B. Befundverbesserung) oder regelmäßig durchzuführenden Re-Assessments festgestellt wird, dass die besonderen Mittel der SAPV nicht mehr notwendig sind und die Mittel der Regelversorgung ausreichen.

## § 6

### Kostenzusage

(1) Die Ersatzkasse übernimmt vom ärztlich festgelegten Leistungsbeginn an bis zu einer Entscheidung über die weitere Leistungserbringung die Kosten für die verordneten und vom Leistungserbringer erbrachten Leistungen entsprechend der Vergütung gemäß dieses Vertrages, wenn die ordnungsgemäß ausgefüllte Verordnung spätestens am dritten der Ausstellung folgenden Arbeitstag der Ersatzkasse vorliegt. Sie informiert hierüber den Leistungserbringer.

(2) Soweit die verordneten Leistungen nicht übernommen werden, informiert die Ersatzkasse zeitgleich den/die Versicherte/n, ggf. gesetzlichen Vertreter, den Leistungserbringer und den verordnenden Arzt.

(3) Sofern die Verordnung verspätet eingereicht wird, werden die Kosten erst ab Eingangsdatum übernommen.

(4) Liegt der Ersatzkasse innerhalb von 7 Werktagen ab Verordnungsbeginn zusätzlich zur Erstverordnung kein Ergebnis über das SAPV-Eingangsassessment des Palliativmediziners vor, endet die Kostenzusage mit Ablauf dieses Tages. Die Ersatzkasse informiert zeitgleich den/die Versicherte/n, ggf. gesetzlichen Vertreter, den Leistungserbringer und den verordnenden Arzt.

## § 7

## Inhalt der Leistung

(1) Der Leistungserbringer erbringt die jeweils ärztlich verordnete SAPV-Leistung als multiprofessionelles PCT intermittierend oder durchgängig nach Bedarf als

1. **Beratungsleistung**:

    a. Telefonat bzw. persönliches Gespräch mit Betroffenen (Versicherten oder deren Angehörige)

    b. Telefonat bzw. persönliches Gespräch mit einem/einer der Leistungserbringer der Primärversorgung

    c. Dokumentation

2. **Koordination der Versorgung**:

    a. Persönlicher, ggf. ergänzender telefonischer Kontakt mit den an der Versorgung Beteiligten

    b. Ressourcenfokussierte Versorgungsplanung

    c. Assessment, Therapie und Notfallplanung, Vernetzung mit ambulanten und/oder stationären Leistungserbringern

    d. Für die verordnete Koordination besteht 24-Stunden-Rufbereitschaft und Einsatzbereitschaft in Absprache mit den Leistungserbringern der Primärversorgung. Die Rufbereitschaft kann nicht als alleinige Teilleistung verordnet werden.

    e. Dokumentation und Evaluation

3. **Additiv unterstützende Teilversorgung**:

    a. Beratungs- und Koordinationsleistung

    b. Hausbesuche zur Tages- und Nachtzeit

c. Einzelne Leistungen der in § 5 Abs. 3 der SAPV-RL aufgeführten Leistungen

d. Für die verordnete Teilversorgung besteht 24-Stunden-Rufbereitschaft und Einsatzbereitschaft in Absprache mit den Leistungserbringern der Primärversorgung. Die Rufbereitschaft kann nicht als alleinige Teilleistung verordnet werden.

e. Dokumentation und Evaluation

4. **Vollständige Versorgung** :

a. Beratungs- und Koordinationsleistung

b. Hausbesuche zur Tages- und Nachtzeit

c. Alle Leistungen der in § 5 Abs. 3 der SAPV-RL aufgeführten Leistungen.

d. Für die verordnete Vollversorgung besteht 24-Stunden-Rufbereitschaft und Einsatzbereitschaft in Absprache mit den Leistungserbringern der Primärversorgung. Die Rufbereitschaft kann nicht als alleinige Teilleistung verordnet werden.

e. Dokumentation und Evaluation

(2) Angehörige und dem/der Versicherten nahe stehende Personen sollen in die Pflege sowie in die psychosoziale Unterstützung und Sterbebegleitung mit einbezogen werden.

## § 8

## Zuzahlungen

(1) SAPV wird als Sachleistung zur Verfügung gestellt. Jegliche Zuzahlungen vom/von der Versicherten im Rahmen der Vertragsleistungen dürfen weder gefordert noch angenommen werden.

(2) Zuzahlungen dürfen auch in dem Fall nicht verlangt werden, wenn die Verordnung bei der Ersatzkasse verspätet eingereicht wird, da Kosten erst ab

Eingang der Verordnung bei der Ersatzkasse gemäß § 6 dieses Vertrages übernommen werden.

(3) Zuwiderhandlungen stellen einen schweren Vertragsverstoß dar

## § 9

### Vermittlungsverbot

(1) Die Annahme von Aufträgen zur Versorgung anspruchsberechtigter Personen dieses Vertrages und deren Weitergabe (Vermittlung an Dritte, die nicht Teammitglieder des PCT sind, gegen Entgelt oder zur Erlangung geldwerter Vorteile) stellt einen schweren Vertragsverstoß dar.

(2) Vermittlung im Sinne dieser Bestimmung ist auch die Weitergabe von in eigenem Namen angenommenen Aufträgen zur Versorgung anspruchsberechtigter Personen dieses Vertrages an Dritte, die nicht Teammitglieder des PCT sind, gegen Kostenerstattung.

## § 10

### Qualifikationsvoraussetzungen

(1) Die Leistungen der SAPV werden ausschließlich durch Personen erbracht, die folgende Qualifikationsvoraussetzungen erfüllen:

a. Ärztinnen und Ärzte, die

- über eine anerkannte Zusatzweiterbildung Palliativmedizin nach der aktuell gültigen Weiterbildung der jeweiligen Landesärztekammer (grds. 160 Std. Weiterbildung) verfügen und
- die Erfahrung aus der ambulanten palliativen Behandlung von mindestens 75 Palliativpatienten nachweisen können, z. B. in der häuslichen Umgebung oder in einem stationären Hospiz, innerhalb der letzten drei Jahre oder aus einer mindestens einjährigen klinischen palliativmedizinischen Tätigkeit in einer Palliativabteilung in einem Krankenhaus innerhalb der letzten drei Jahre.

b. Pflegefachkräfte, die

- über die Erlaubnis zur Führung einer der Berufsbezeichnungen Gesundheits- und Krankenpflegerin / Gesundheits- und Krankenpfleger, Gesundheits- und Kinderkrankenpflegerin / Gesundheits- und Kinderkrankenpfleger oder Altenpflegerin / Altenpfleger entsprechend den gesetzlichen Bestimmungen in der jeweils gültigen Fassung verfügen und

- den Abschluss einer Palliative-Care-Weiterbildungsmaßnahme im Umfang von mindestens 160 Stunden vorweisen und

- die Erfahrung durch mindestens eine zweijährige praktische Tätigkeit als Pflegefachkraft in der Betreuung von Palliativpatienten in den letzten drei Jahren, davon mindestens sechsmonatige Mitarbeit in einer spezialisierten Einrichtung der Hospiz- oder Palliativversorgung, nachweisen können.

c. Soweit weitere Fachkräfte (z. B. Sozialarbeiterinnen / Sozialarbeiter, Sozialpädagoginnen oder Sozialpädagogen, Psychologinnen oder Psychologen) vertraglich eingebunden werden, haben diese eine Zusatzweiterbildung Palliative Care für andere Berufsgruppen oder eine mehrjährige Erfahrung in der Palliativversorgung nachzuweisen.

d. Ärzte, Pflegefachkräfte und weitere Fachkräfte, die

- sich in Palliative-Care-Weiterbildung gemäß der jeweils zutreffenden Ziffer (1) a - c befinden und

- ambulante Palliativleistungen unter Supervision eines Mitarbeiters der entsprechenden Berufsgruppe des PCT erbringen, der die Palliative-Care-Weiterbildung gemäß Ziffer (1) a - c abgeschlossen hat und darüber hinaus über eine weitere 1-jährige Erfahrung in der Palliativversorgung verfügt. Berufsgruppen der weiteren Fachkräfte unter Ziffer (1) c können ihre Leistungen auch unter Supervision eines Arztes des PCT mit dieser Qualifikation erbringen.

(2) Wenn Kinder versorgt werden, müssen die Anforderungen an die ärztlichen und pflegerischen Personen und an die weiteren Fachkräfte den besonderen Belangen der Kinder gerecht werden.

## § 11

### Verordnung von Arznei-, Heil- und Hilfsmitteln im Rahmen der SAPV

(1) Der Palliativarzt/die Palliativärztin des PCT verordnet die notwendigen Arzneimittel unter Verwendung des Musters 16 auf den Namen des/der Versicherten.

(2) Der Palliativarzt/die Palliativärztin des PCT benötigt für die Abrechnung der auf Muster 16 verordneten Arzneimittel eine Betriebsstättennummer. Diese wird von der zuständigen Kassenärztlichen Vereinigung vergeben.

(3) Verfügt der Palliativarzt/die Palliativärztin des PCT über keine Vertragsarztnummer, die auf dem Muster 16 neben der Betriebsstättennummer anzugeben ist, erteilt die Kassenärztliche Vereinigung eine Dummy-Nummer.

(4) Der Leistungserbringer ist verpflichtet, die Betriebsstättennummer sowie ggf. für Nicht-Vertragsärzte eine Dummy-Nummer zu beantragen.

(5) Heilmittel: Die Verordnung von Heilmitteln erfolgt gem. Heilmittelrichtlinie

(6) Hilfsmittel: Für die Versorgung mit Hilfsmitteln ist das Versorgungmanagement (Pflegeberater) zu nutzen. Für Fälle, in denen eine Hilfsmittelversorgung notfällig indiziert und die Bereitstellung über das Versorgungsmanagement des zuständigen Kostenträgers nicht möglich ist, treffen die Vertragsparteien noch gesonderte Absprachen.

## § 12

### Sächliche Ausstattung

(1) Eine Versorgung mit Arznei- und Hilfsmitteln im Notfall (Notfallversorgung) ist durch geeignete Maßnahmen z. B. durch eine Kooperation mit einer Apotheke bzw. einem Sanitätshaus sicherzustellen. Von besonderer Bedeutung ist die Vorhaltung ausreichender und geeigneter Arzneimittel/BtM für die Versorgung im Not- und Kriseninterventionsfall im Arzt-/Pflegekoffer/Bereitschaftstasche.

(2) Die an die Aufbewahrung von BtM gestellten Voraussetzungen nach dem BtMG sind zu berücksichtigen.

(3) Der Leistungserbringer hat die Mindestanforderungen an die Ausstattung seiner Räumlichkeiten gemäß Ziffer 4.4 und 4.5 der Gemeinsamen Empfehlungen nach § 132 d Abs. 2 SGB V vom 23.06.2008 nachzuweisen.

(4) Der vdek hat das Recht, jederzeit selbst und/oder durch Beauftragung des Medizinischen Dienstes der Krankenkassen nach § 275 SGB V vor Ort die Erfüllung der Mindestanforderungen an die sächliche Ausstattung gem. § 12 zu überprüfen.

## § 13

## Organisation

(1) Der Leistungserbringer verpflichtet sich zu einer 24-Stunden-Rufbereitschaft und Einsatzbereitschaft durch das PCT. Die ständige Verfügbarkeit eines Arztes/Ärztin und einer Pflegefachkräfte gemäß § 10 dieser Vereinbarung ist auch für notwendige Hausbesuche zu gewährleisten.

(2) Zur Gewährleistung eines zeitnahen und systematischen Informationstransfers bei Überleitung aus/in stationäre Einrichtungen (z. B. Krankenhaus, stationäre Pflegeeinrichtung) ist ein geeignetes Überleitungsmanagement sicherzustellen.

(3) Es erfolgt eine enge Kooperation des Leistungserbringers mit dem behandelnden Haus- oder Facharzt hinsichtlich der Therapieplanung. Insbesondere erfolgt vor einer stationären Krankenhauseinweisung eine Abstimmung zwischen dem PCT und dem/der behandelnden Haus- oder Facharzt/ärztin.

(4) Der Leistungserbringer stellt durch eine geeignete Infrastruktur sicher, dass die ständige Kommunikation innerhalb des PCT und zu allen an der Versorgung des jeweiligen Versicherten Beteiligten gewährleistet ist.

## § 14

## Patientendokumentation

(1) Der Leistungserbringer hat eine Patientendokumentation zu führen, aus der zu jeder Zeit der Behandlungsverlauf hervorgeht. Die Patientendokumentation wird regelmäßig von dem PCT aktualisiert.

(2) Das Dokumentationssystem hat allgemein anerkannte Kriterien für eine externe Qualitätssicherung zu berücksichtigen, um eine bundesweite Evaluation zu ermöglichen. Einzelheiten der Dokumentation ergeben sich aus der **Anlage 2** dieser Vereinbarung.

(3) Weiterhin hat der Leistungserbringer anzustreben, dass die von ihm angewandte Dokumentation kompatibel zu bestehenden Dokumentationssystemen der an der Versorgung beteiligten Leistungserbringer ist.

(4) Einzelheiten zur Dokumentation und ihrer Weiterentwicklung sind in Anlage 2 ausgeführt

(5) Die versorgungsrelevante Dokumentation verbleibt beim Patienten.

(6) Die Dokumentation ist dem MDK und – soweit der Versicherte eingewilligt hat (§ 67 b Abs. 1 SGB X) – der Ersatzkasse auf Anforderung zugänglich zu machen.

(7) Die Aufbewahrungsfrist für die Dokumentation beträgt 10 Jahre nach Ende des Kalenderjahres der Leistungserbringung.

## § 15

## Datenschutz, Schweigepflicht

(1) Der Leistungserbringer ist verpflichtet, die Bestimmungen über den Schutz der Sozialdaten (SGB X 2. Kapitel) zu beachten, insbesondere personenbezogene Daten nur zur Erfüllung der sich aus diesem Vertrag ergebenden Aufgaben zu verarbeiten, bekannt zu geben, zugänglich zu machen oder sonst zu nutzen.

(2) Der Leistungserbringer unterliegt hinsichtlich der Person des Versicherten und dessen Krankheiten der Schweigepflicht. Ausgenommen hiervon sind Angaben gegenüber der leistungspflichtigen Ersatzkasse und/oder dem MDK, soweit sie zur Erfüllung der gesetzlichen Aufgaben der Ersatzkasse erforderlich sind.

(3) Der Leistungserbringer hat die im Team tätigen Mitarbeiter zur Beachtung der Datenschutzbestimmungen und der Schweigepflicht zu verpflichten. Die Verpflichtung ist zu dokumentieren.

## § 16

### Vergütung

(1) Die SAPV-Leistungen des PCT werden als Komplexpauschalen vergütet. Die Höhe der Vergütung richtet sich nach den in der Anlage 1 aufgeführten Pauschalen.

(2) Mit den Komplexpauschalen nach Anlage 1 sind alle Aufwendungen des Leistungserbringers abgegolten, die im Zusammenhang mit der SAPV-Leistung stehen. Eine zeitgleiche Abrechnung von Gebührenordnungspositionen des Einheitlichen Bewertungsmaßstabes (EBM) über die Kassenärztliche Vereinigung ist daneben ausgeschlossen.

(3) Die Vergütung erfolgt ausschließlich gegenüber dem Leistungserbringer.

(4) Wird mit einer anderen Krankenkasse oder einem anderen Krankenkassen-Verband eine Vereinbarung abgeschlossen, welche die gleichen Leistungen zur Geschäftsgrundlage hat und für die günstigere Preise oder eine alternative Vergütungssystematik zur Anwendung kommen, so gilt diese auch optional für die an diesem Vertrag teilnehmenden Ersatzkassen. Über den Abschluss einer günstigeren oder alternativen Vergütungsvereinbarung ist die vdek-Landesvertretung unverzüglich zu informieren

## § 17

### Qualitätssicherungsmaßnahmen

(1) Der Leistungserbringer ist verpflichtet, ein Qualitätsmanagement durchzuführen, das im Versorgungskonzept darzustellen ist. Zur Qualitätssicherung zählen interne und externe Maßnahmen.

(2) Die Einzelheiten zu den Qualitätssicherungsmaßnahmen sind in Anlage 4 geregelt.

## § 18

### Abrechnung

(1) Die Abrechnung der in der Anlage 1 aufgeführten Komplexpauschalen erfolgt direkt mit den Krankenkassen.

(2) Der Leistungserbringer kann seine Leistungen nur abrechnen, wenn er über ein IK gemäß § 19 verfügt.

(3) Für die Abrechnung wird mittelfristig die Anwendung der Richtlinien der Spitzenverbände der Krankenkassen, jetzt Spitzenverband Bund, über Form und Inhalt des Abrechnungsverfahrens mit sonstigen Leistungserbringern nach § 302 Abs. 2 SGB V in der jeweils aktuellen Fassung angestrebt.

(4) Bis dahin erfolgt die Abrechnung in Papierform und enthält folgende Angaben:

- Name, Anschrift und Institutionskennzeichen des Leistungserbringers
- Name, Vorname, Versichertennummer und Geburtsdatum des Versicherten
- Verordnungsblätter im Original
- Ggf. Leistungszusagen der Krankenkassen im Original,
- Angabe des Versorgungszeitraums, Versorgungsortes sowie der dafür anzusetzenden Komplexpauschalen gem. Anlage 1.
- Rechnungsbetrag und Bankverbindung

Die Rechnungslegung erfolgt auf einem Formular gem. Anlage 5 jeweils nach Fallabschluss. Nach Überschreiten eines dreimonatigen Versorgungszeitraums erfolgt eine Zwischenabrechnung.

(5) In der Abrechnung ist der Leistungserbringergruppenschlüssel (LEGS), soweit eingeführt, gemäß der Richtlinien nach § 302 SGB V anzugeben.

(6) Bei Differenzen bzw. begründeten Beanstandungen der Abrechnung kann die Ersatzkasse dem Leistungserbringer die eingereichten Unterlagen oder die Datensätze zur Prüfung bzw. Korrektur zurückgeben.

Die Bezahlung der Rechnungen erfolgt innerhalb von maximal 30 Tagen nach Eingang der vollständigen Abrechnungsunterlagen bei den von den Ersatzkassen benannten Stellen. Bei Zahlung durch Überweisung gilt die Frist als gewahrt, wenn der Auftrag innerhalb dieser Zeit dem Geldinstitut erteilt wurde.

Überträgt der Leistungserbringer die Abrechnung einer Abrechnungsstelle, so hat der Leistungserbringer die zuständige vdek-Landesvertretung und die Ersatzkassen unverzüglich schriftlich hierüber zu informieren. Der Beginn und das Ende des Auftragsverhältnisses, der Name der beauftragten Abrechnungsstelle und das Institutionskennzeichen, unter dem die Abrechnungsstelle die Rechnungslegung vornimmt, ist mitzuteilen. Das

Abrechnungszentrum ist verpflichtet, sich ebenfalls gemäß § 302 Abs. 2 SGB V zum maschinellen Datenaustausch anzumelden. Abrechnungszentren liefern die Abrechnung ausschließlich auf maschinell verwertbaren Datenträgern nach § 302 Abs. 1 SGB V.

Der Leistungserbringer ist für die Einhaltung der gesetzlichen und vertraglichen Regelungen durch das Abrechnungszentrum verantwortlich.

Hat der Leistungserbringer dem Abrechnungszentrum eine Inkasso-Vollmacht erteilt, erfolgt die Zahlung an das Abrechnungszentrum für die Ersatzkassen mit schuldbefreiender Wirkung. Wird dem Abrechnungszentrum die Inkasso-Vollmacht entzogen, ist der Leistungserbringer für die Einhaltung des Entzugs der Vollmacht verantwortlich.

Sofern die Rechnungslegung einer Abrechnungsstelle übertragen werden soll, ist der Auftragnehmer unter besonderer Berücksichtigung der von ihm getroffenen technischen und organisatorischen Maßnahmen zur Sicherstellung der Maßgaben dieses Vertrages und des § 6 Abs. 1 BDSG durch den Leistungserbringer auszuwählen. Die getroffene Vereinbarung über Datenschutz und Datensicherung beim Auftragnehmer (Abrechnungsstelle) ist der zuständigen vdek-Landesvertretung vorzulegen.

(7) Die Abrechnung von Arzneimitteln erfolgt nach der Vereinbarung über die Übermittlung von Daten im Rahmen der Arzneimittelabrechnung gemäß § 300 SGB V und dessen technische Anlagen.

## § 19

### Institutionskennzeichen

(1) Der Leistungserbringer verfügt gemäß § 293 SGB V über ein Institutionskennzeichen (IK), das er bei der Abrechnung mit den Ersatzkassen verwendet.

Das IK ist bei der Sammel- und Verteilungsstelle IK der Arbeitsgemeinschaft Institutionskennzeichen (SVI), Alte Heerstr. 111, 53757 St. Augustin, zu beantragen.

Änderungen der unter dem IK gespeicherten Daten sind der SVI unverzüglich mitzuteilen. Mitteilungen an die Ersatzkassen oder ihre mit der Abrechnungsprüfung beauftragten Dienstleister werden nicht berücksichtigt.

(2) Das gegenüber den Ersatzkassen eingesetzte IK ist der zuständigen vdek-Landesverhandlungskommission, der Landesvertretung sowohl bei Vertragsabschluss als auch bei Änderungen mitzuteilen. Abrechnungen mit den Ersatzkassen erfolgen ausschließlich unter diesem IK.

Das IK ist in jeder Abrechnung anzugeben. Abrechnungen ohne IK oder mit fehlerhaftem IK werden von den Ersatzkassen abgewiesen. Gleiches gilt für Abrechnungen mit einem der Ersatzkasse unbekannten IK.

Die unter dem gegenüber den Ersatzkassen verwandten IK bei der SVI gespeicherten Angaben, einschließlich der Bank- und Kontoverbindung sind verbindlich für die Abrechnungsbegleichung durch die Ersatzkassen. Andere Bank- und Kontoverbindungen werden von den Ersatzkassen bei der Abrechnung nicht berücksichtigt.

## § 20

### Statistik

Der Leistungserbringer ist verpflichtet, an Auswertungen mitzuwirken. Hierzu übermittelt er halbjährlich (30.06., 31.12. d. J.) folgende Leistungsdaten über die LAPH an den Beirat (vgl. § 22).

(1) Die zu übermittelnden Daten beziehen sich auf folgende Bereiche:

- Fallzahlen (Anzahl der behandelten Patienten),
- SAPV leistungsbegründende Diagnosen aufgrund vorliegender ärztlicher Verordnung,
- Versorgungsbereich gemäß Versorgungskonzept,
- Anzahl der Krankenhauseinweisungen,
- Anzahl der Patienten, die in der Häuslichkeit verstorben sind,
- Versichertenbezogene Versorgungstage in Mittelwert und Median,
- Einstufungen der Patienten in Pflegestufen nach dem SGB XI

## § 21

### Beirat, Aufgaben

(1) Es wird ein landesweiter Beirat gebildet.

(2) Der Beirat wird mit Mitgliedern der Leistungserbringer und der Kassenseite besetzt.

(3) Zu den Aufgaben des Beirates gehören die Begleitung dieser Vereinbarung und das Aussprechen von Empfehlungen insbesondere hinsichtlich der Qualität der Versorgungsprozesse sowie zur Anpassung an regionale Versorgungs-bedürfnisse.

(4) Der Beirat tagt grundsätzlich einmal im Jahr, auf Verlangen jeweils eines Vertragspartners auch ein weiteres mal. Die Organisation und Versendung der Einladung zur Sitzung erfolgt durch die LAPH.

(5) Die Aufwendungen für die Teilnahme an den Beiratssitzungen werden durch die Vertragspartner jeweils selbst getragen.

## § 22

### Maßnahmen bei Vertragsverstößen

(1) Vertragsverstöße und/oder Schäden sind dem Leistungserbringer durch die Ersatzkassen in schriftlicher Form mitzuteilen. Hierbei ist dem Leistungserbringer Gelegenheit zur Stellungnahme innerhalb einer angemessenen Frist zu geben (Anhörung).

## § 23

### Ordentliche / außerordentliche Kündigung

(1) Der Vertrag kann von jedem Vertragspartner unter Angabe des Grundes mit einer Frist von 3 Monaten zum Ende eines Kalenderjahres ganz oder teilweise gekündigt werden (ordentliche Kündigung). Die Kündigung bedarf der Schriftform. Die Vergütungsvereinbarung gem. Anlage 1 erhält eine gesonderte Laufzeit und Kündigungsfrist.

(2) Eine außerordentliche Kündigung dieses Vertrages ist nur aus wichtigem Grund möglich. Ein wichtiger Grund, der die Vertragsparteien zur Kündigung berechtigt, liegt insbesondere vor,

a. wenn die Voraussetzungen dieses Vertrages aus Gründen der Rechtsentwicklung, wesentlicher medizinisch-wissenschaftlicher oder tatsächlicher Gründe entfallen,

b. wenn die Leistungen, die Gegenstand dieses Vertrages sind, nicht erbracht oder in erheblichem Umfang mangelhaft, unwirtschaftlich oder unvollständig erbracht werden,

c. bei schwerem Verstoß gegen die Inhalte dieses Vertrages,

d. wenn auf Grund einer Weisung der jeweils zuständigen Aufsichtsbehörde eine Fortsetzung des Vertrages untersagt wird.

## § 24

### Inkrafttreten, Vertragslaufzeit

(1) Diese Vereinbarung tritt am 01. April 2009 in Kraft.

(2) Die Laufzeit dieser Vereinbarung erstreckt sich bis zum 31.12.2009 und verlängert sich jeweils um ein Jahr, sofern sie nicht von einer der Vertragsparteien gem. § 23 gekündigt wird.

## § 25

### Salvatorische Klausel

(1) Sollten einzelne Bestimmungen dieses Vertrages unwirksam oder undurchführbar sein oder nach Vertragsschluss unwirksam oder undurchführbar werden, bleibt davon die Wirksamkeit des Vertrages im Übrigen unberührt.

(2) An die Stelle der unwirksamen oder undurchführbaren Bestimmung soll diejenige wirksame und durchführbare Regelung treten, deren Wirkungen der Zielsetzung zur Erbringung von SAPV am nächsten kommen, die die Vertragsparteien mit der unwirksamen bzw. undurchführbaren Bestimmung verfolgt haben.

(3) Die vorstehenden Bestimmungen gelten entsprechend für den Fall, dass sich der Vertrag als lückenhaft erweist.

(4) Die Vertragsparteien verständigen sich dazu unverzüglich über notwendige neue Regelungen.

| | |
|---|---|
| Datum, Unterschrift<br>Der Leiter der<br>Landesvertretung Hessen | Datum, Unterschrift<br>Leistungserbringer |

## Anlage 1

## Vergütungsvereinbarung

Komplexpauschalen gemäß § 7 i.V.m. § 16 und § 18 dieser Vereinbarung

(1) Für die SAPV-Leistungen Koordination, Teilversorgung und Vollversorgung inklusive Eingangsassessment (siehe § 5 (8)) wird eine einheitliche Pauschalierung (P1-P3) vereinbart. Die Versorgung wird im stationären Hospiz mit den Pauschalen H1-H2, die Beratungsleistung mit den Pauschalen B1-B2. vergütet.
Die Anwendung von Komplexpauschalen ermöglicht eine flexible Anpassung des Leistungsumfangs an den individuellen Versorgungsbedarf und zugleich Erfüllung des Versorgungsanspruchs des Versicherten gem. SAPV-RL.

(2) Die Höhe der SAPV-Komplexpauschale für Koordination, Teilversorgung und Vollversorgung wird abhängig von der SAPV-Versorgungsdauer degressiv gestaffelt. Für die Anwendung der Degressionsstufen sind ausschließlich SAPV-Versorgungstage zu berücksichtigen.

(3) Wird die SAPV unterbrochen, dann entspricht die SAPV-Versorgungsdauer der Summe aller SAPV-Versorgungstage des betreffenden Patienten.

(4) Werden die SAPV-Leistungen im Versorgungsverlauf vollständig außerhalb eines stationären Hospizes erbracht, erhält der Leistungserbringer für Koordination, Teilversorgung und Vollversorgung

a. **P1**: 1500 € als Festbetrag bei einer Versorgungsdauer bis zu 10 Tagen, auch wenn die Versorgungsdauer weniger als 10 Tage beträgt,

b. **P2**: 120 € pro Versorgungstag bei einer Versorgungsdauer bis zu 56 Tagen ab dem 11. Versorgungstag zusätzlich zur Vergütung aus (4a),

c. **P3**: 80 € pro Versorgungstag bei einer Versorgungsdauer von mehr als 56 Tagen ab dem 57. Versorgungstag zusätzlich zur Vergütung aus (4a) und (4b)

(5) Werden die SAPV-Leistungen im Versorgungsverlauf vollständig innerhalb eines stationären Hospizes erbracht, erhält der Leistungserbringer für Koordination, Teilversorgung und Vollversorgung

a. **H1**: 750 € als Festbetrag bei einer Versorgungsdauer bis zu 10 Tagen, auch wenn die Versorgungsdauer weniger als 10 Tage beträgt,

b. **H2**: 60 € pro Versorgungstag bei einer Versorgungsdauer von mehr als 10 Tagen ab dem 11. Versorgungstag zusätzlich zur Vergütung aus (5a)

(6) Werden die SAPV-Leistungen im Versorgungsverlauf wechselnd inner- und außerhalb eines stationären Hospizes erbracht, erhält der Leistungserbringer für Koordination, Teilversorgung und Vollversorgung

a. **P1**: 1500 € als Festbetrag bei einer Versorgungsdauer bis zu 10 Tagen, auch wenn die Versorgungsdauer weniger als 10 Tage beträgt,

b. bei einer Versorgungsdauer von mehr als 10 Tagen: ab dem 11. Versorgungstag die für den Versorgungsort nach Ziffer (4) bzw. (5) zutreffende Vergütung des jeweiligen Versorgungstages. Für den Tag des Wechsels in das bzw. aus dem Hospiz gilt die Vergütung nach Ziffer (4).

(7) Für die SAPV-Leistung Beratung erhält der Leistungserbringer

a. **B1**: 170 € bei der ersten Beratung

b. **B2**: 75 € bei jeder weiteren Beratung

(8) Diese Vergütungsvereinbarung tritt zum 01.04.2009 in Kraft. Sie kann mit einer Frist von 3 Monaten zum 30. Juni oder 31. Dezember eines jeden Jahres gekündigt werden. Wird die Vereinbarung gekündigt, gelten die Preise dieser Vereinbarung bis zu einer Neuregelung weiter.

## Anlage 2

## Dokumentation

(1) Für sämtliche SAPV-Leistungen (Beratung, Koordination, Teilversorgung und Vollversorgung) wird eine einheitliche Dokumentationsgrundlage vereinbart.

(2) Zugrundeliegende Kategorien der Dokumentation sind

a. Verlaufsstabile Grund- und Stammdaten

b. Prozessbezogene inhaltliche Verlaufsdokumentation

c. Beim Leistungsempfänger zu verbleibende Unterlagen

d. Dokumentation von Kennzahlen zur Beurteilung der Prozess-, Ergebnis- und Strukturqualität.

(3) Die Grund- und Stammdaten gemäß 2a schließen folgende Punkte ein

a. Name, Vorname, Geburtsdatum, Adresse, telefonische Erreichbarkeit des Leistungsempfängers

b. Name, Vorname, Geburtsdatum, Adresse, telefonische Erreichbarkeit des gesetzlichen Vertreters und/oder Bevollmächtigten und/oder wichtiger Bezugspersonen des Leistungsempfängers

c. Name, Adresse, telefonische Erreichbarkeit der mit dem PCT kooperierenden Leistungserbringer der Regelversorgung, z.B. Hausärzte, Fachärzte, Pflegedienste, Hospizdienste u.a.

d. Patientenbezogene Daten zur Beschreibung der Ausgangssituation zum Versorgungsbeginn gemäß „Gemeinsamer Datensatz zur SAPV-Dokumentation: von DGP (Deutsche Gesellschaft für Palliativmedizin) und DHPV (Deutscher Hospiz und Palliativverband) Teil 1: Kerndatensatz für Palliativpatienten in ihrer jeweils aktuellen Fassung (aktuell: Version 1; 15.01.2009)

e. Angaben zur Palliativfall begründenden Diagnose

f. Angaben zur besonderen Komplexität und/oder besonderen Aufwand gemäß §37b SGB V und SAPV-RL

(4) Prozessbezogene inhaltliche Verlaufsdokumentation gemäß 2b schließt ein

a. Ergebnisse der Erhebung des Ganzkörperstatus mittels körperlicher Untersuchung zum Zeitpunkt der SAPV Erstverordnung

b. Ergebnisse der Erhebung der Pflegeanamnese zum Zeitpunkt der SAPV Erstverordnung

c. Ergebnisse der Analyse der Selbsthilfefähigkeit zum Zeitpunkt der SAPV Erstverordnung

d. Ergebnisse der Umfeldanalyse des privaten häuslichen Betreuungsumfeldes zum Zeitpunkt der SAPV Erstverordnung

e. Ergebnisse der ausführlichen Anamnese inklusive ergänzender fremdanamnestischer Auskünfte bzgl. der Dimensionen medizinische, pflegerische und psychosoziale krankheitsassoziierte Belastetheit zum Zeitpunkt der SAPV-Erstverordnung

f. Ergebnisse der multiprofessionellen Teambesprechung und Fallanalyse mit Formulierung eines Behandlungsplanes.

g. Bericht über die Ergebnisse der Ersterhebung und konsekutive Behandlungsmaßnahmen /-empfehlungen an die Kooperationspartner des PCT.

h. Ergebnisse des fortlaufenden Monitorings der Belastetheit des Patienten im Behandlungsverlauf bei SAPV Komplexleistung

i. Ergebnisse der fortlaufenden multiprofessionellen Teambesprechungen bei SAPV Komplexleistung

j. Adaption und Fortschreibung des individuellen Therapieplanes bei SAPV Komplexleistung

k. Adaption und Fortschreibung des individuellen Notfallplanes bei SAPV Komplexleistung

(5) Beim Leistungsempfänger zu verbleibende Unterlagen gemäß 2c sind insbesondere

a. Bericht über die Ergebnisse der Ersterhebung und konsekutive Behandlungsmaßnahmen /-empfehlungen

b. Aktueller Behandlungsplan

c. Aktueller Notfallplan

d. Unterlagen mit eindeutiger Nennung der Erreichbarkeitsdaten des PCT bei SAPV Komplexleistung

(6) Dokumentation von Kennzahlen zur Beurteilung der Prozess-, Ergebnis- und Strukturqualität gemäß 2d umfasst insbesondere

a. Alle in Anlage 4 (Qualitätssicherung) festgeschriebenen Kennzahlen zur der Prozess-, Ergebnis- und Strukturqualität

b. Alle Items des „Gemeinsamen Datensatzes zur SAPV-Dokumentation: von DGP und DHPV in der jeweils gültigen Fassung.

- Teil 1: Kerndatensatz für Palliativpatienten in ihrer jeweils aktuellen Fassung (aktuell: Version 1; 15.01.2009) zu Beginn und Abschluss der Versorgung

- Angaben zum weiteren Verlauf / Abschluss (Ergebnisqualität) zum Ende der SAPV Versorgung

- Teil 2: SAPV Struktur Modul in jeweils aktueller Fassung (aktuell: Version 1; 15.01.2009) bei jeder Verordnung

c. Der „Gemeinsame Datensatz zur SAPV-Dokumentation von DGP und DHPV“ wird gemäß der „Einführung von DHPV und DGP zu den Dokumentationsgrundlagen für die SAPV“ in Ihrer jeweils gültigen Fassung verwendet (Stand: 15.01.2009)

**Gemeinsamer Datensatz zur SAPV-Dokumentation** (Version 1 / 15.01.2009)

<table>
<tr><th colspan="2">I. Kerndatensatz für Palliativpatienten:<br>Erfassung zu Beginn und Ende der Palliativversorgung</th></tr>
<tr><th>Items</th><th>Antworten, Kategorien</th></tr>
<tr><th colspan="2">Demographische Angaben</th></tr>
<tr><td>Geburtsdatum</td><td>Datum</td></tr>
<tr><td>Aufnahmedatum</td><td>Datum</td></tr>
<tr><td>Geschlecht</td><td>| weiblich | männlich</td></tr>
<tr><td>Wohnsituation</td><td>| allein | Heim | mit Angehörigen | Sonstige</td></tr>
<tr><th colspan="2">Angaben zur Erkrankung</th></tr>
<tr><td>Hauptdiagnose/Grundkrankheit<br>(palliativmedizinisch relevant)</td><td>ICD</td></tr>
<tr><td>Funktionsstatus (ECOG)</td><td>| Normale Aktivität | Gehfähig, leichte Arbeit möglich<br>| Nicht arbeitsfähig, kann > 50% der Wachzeit aufstehen<br>| Begrenzte Selbstversorgung, > 50% Wachzeit bettlägerig<br>| Pflegebedürftig, permanent bettlägerig</td></tr>
<tr><th colspan="2">Angaben zur Versorgung (Strukturqualität)</th></tr>
<tr><td>Behandlung, Begleitung<br>(bei Behandlungsbeginn<br>oder bei Übernahme des Patienten)</td><td>ambulant:<br>| SAPV-Team | Hausarzt | Ambulante Pflege<br>| Palliativarzt (QPA) | Palliativpflege (AHPP, APD)<br>| Palliativberatung (AHPB) | Ehrenamtlicher Dienst<br>stationär:<br>| KH (Allgemeinstation) | KH (Palliativstation)<br>| Heim | Stationäres Hospiz | Sonstige</td></tr>
<tr><td>Absprache / Vorlage</td><td>| Patientenverfügung | Vorsorgevollmacht | Betreuung</td></tr>
<tr><td>Datum der Erfassung</td><td>Datum</td></tr>
<tr><th colspan="2">Angaben zum Behandlungsbedarf</th></tr>
<tr><td>Schmerzen</td><td>| kein | leicht | mittel | stark</td></tr>
<tr><td>Übelkeit</td><td>| kein | leicht | mittel | stark</td></tr>
<tr><td>Erbrechen</td><td>| kein | leicht | mittel | stark</td></tr>
<tr><td>Luftnot</td><td>| kein | leicht | mittel | stark</td></tr>
<tr><td>Verstopfung</td><td>| kein | leicht | mittel | stark</td></tr>
<tr><td>Schwäche</td><td>| kein | leicht | mittel | stark</td></tr>
<tr><td>Appetitmangel</td><td>| kein | leicht | mittel | stark</td></tr>
<tr><td>Müdigkeit</td><td>| kein | leicht | mittel | stark</td></tr>
<tr><td>Pflegeprobleme wegen Wunden / Dekubitus</td><td>| kein | leicht | mittel | stark</td></tr>
<tr><td>Hilfebedarf bei Aktivitäten des tägl. Lebens</td><td>| kein | leicht | mittel | stark</td></tr>
<tr><td>Depressivität</td><td>| kein | leicht | mittel | stark</td></tr>
<tr><td>Angst</td><td>| kein | leicht | mittel | stark</td></tr>
<tr><td>Anspannung</td><td>| kein | leicht | mittel | stark</td></tr>
<tr><td>Desorientiertheit, Verwirrtheit</td><td>| kein | leicht | mittel | stark</td></tr>
<tr><td>Probleme mit Organisation der Versorgung</td><td>| kein | leicht | mittel | stark</td></tr>
<tr><td>Überforderung der Familie, des Umfeldes</td><td>| kein | leicht | mittel | stark</td></tr>
<tr><th colspan="2">Angaben zur Therapie (Prozessqualität)</th></tr>
<tr><td>Was ist der Grund des Kontaktes, was das Behandlungsziel?</td><td>Text</td></tr>
<tr><td>Opioide der WHO Stufe III</td><td>ja / nein</td></tr>
<tr><td>Steroide</td><td>ja / nein</td></tr>
<tr><td>Chemotherapie</td><td>nein / fortgesetzt / initiiert</td></tr>
<tr><td>Strahlentherapie</td><td>nein / fortgesetzt / initiiert</td></tr>
<tr><td>besonderer Aufwand mit:</td><td>Text</td></tr>
</table>

| **Angaben zum weiteren Verlauf, Abschluss (Ergebnisqualität)** | |
|---|---|
| Welches Problem konnte besonders gut gelöst werden? | Text |
| Welches Problem konnte nicht ausreichend gelöst werden? | Text |
| Datum (Entlassung / Änderung der Betreuung / Tod) | Datum |
| Therapieende | \| Verstorben \| Verlegung, Entlassung<br>\| Sonstiges: |
| Sterbeort | \| zuhause \| Heim \| Krankenhaus<br>\| Palliativstation \| Hospiz \| unbekannt |
| Zufriedenheit mit der Behandlung für die gesamte Behandlung | \| sehr schlecht \| schlecht \| mittel<br>\| gut \| sehr gut |

| II. SAPV-Struktur-Modul: Erfassung bei jeder Verordnung bzw. Intensitätswechsel | |
|---|---|
| **Items** | **Antworten, Kategorien** |
| **Identifikationsnummer** | Fortlaufende Nr. wie bei Kerndatensatz |
| **Angaben zur Versorgung (Strukturqualität)** | |
| SAPV | \| Beratung \| Koordination<br>\| additive Teilversorgung \| vollständige Versorgung |
| Verordnung Datum | Datum |
| Art der Verordnung | \| Erstverordnung \| Wiederverordnung |
| Verordnung durch | \| Hausarzt \| niedergelassener Facharzt \| KH-Arzt<br>\| andere \| keine Angabe |
| Übernahme aus | \| Eigene Häuslichkeit \| Ambulantes Umfeld<br>\| Stationäre Pflegeeinrichtung \| Stationäres Hospiz<br>\| KH (Allgemeinstation) \| KH (Palliativstation) |
| Beteiligte assoziierte Partner in der SAPV-Erbringung | Ärztlich:<br>\| Hausarzt \| niedergelassener Facharzt (für_______)<br>\| KH-Arzt \| Sonstige<br><br>Pflegerisch:<br>\| Ambulanter Pflegedienst<br>\| Ambulanter Palliativpflegedienst<br>\| Stationäre Pflegeeinrichtung<br>\| Stationäres Hospiz<br><br>Ambulanter Hospizdienst:<br>\| Ambulante Palliativberatung (AHD, AHPB)<br>\| Begleitung durch Ehrenamtliche<br><br>Weitere Professionen:<br>\| Case Management \| Ernährungsberatung<br>\| Physiotherapie \| Psychologie \| Seelsorge<br>\| Sozialarbeit \| Andere:______________________ |
| Regel-Anfahrtsweg in km | |
| **Angaben zum Verlauf** | |
| Anzahl der Teambesprechungen zur Problemlösung (Definition Teambesprechung in Expose) | |
| Krankenhauseinweisungsgrund | Text |
| Datum Ende der SAPV | |
| **Angaben zum Team** | |
| Zahl der Vollzeitäquivalente (multiprofessionell) im SAPV Team | |
| Zahl der zeitgleich maximal betreuten Patienten je Versorgungsstufe | |
| Datum der Erfassung | |

## Anlage 3

## Verordnungsformular für die SAPV

Der Vordruck wird gem. Rundschreiben GKV-Spitzenverband RS 2009/164 vom 06.04.2009 erst zum 3. Quartal 2009 in die Anlage 2/2a der Bundesmantelverträge aufgenommen. Für das 2. Quartal 2009 haben sich KBV und SpiBund darauf verständigt, dass der Verordnungsvordruck für den Arzt zum Download in dem geschützten Bereich der Kassenärztlichen Vereinigung bereit steht.

## Anlage 4

## Qualitätssicherung

(1) Ziel der Qualitätssicherung ist die analytische Bewertung und Einordnung der Struktur, Prozesse und Ergebnisse eines PCT in der Einzelbetrachtung und im Vergleich mit anderen PCTs. Maßnahmen zur Qualitätssicherung sind gem. § 132d (2)2. SGB V zu entwickeln und umzusetzen. An den dazu erforderlichen Datenerhebungen und -lieferungen werden sich die Leistungserbringer beteiligen.

(2) Geforderte Kennzahlen bzgl. der Qualitätssicherung sind im einzelnen

- Komplette Erfassung der fallbezogenen Daten gemäß Kerndatensatz für Palliativpatienten der Deutschen Gesellschaft für Palliativmedizin (DGP) und des Deutschen Hospiz- und Palliativverbandes (DHPV) in seiner jeweils gültigen Fassung bestehend aus Teil 1 Kerndatensatz für Palliativpatienten und Teil 2 Modul SAPV Struktur.

- Der Nachweis (Verlaufsdokumentation) regelmäßiger multiprofessioneller Fallbesprechungen

- Die regelmäßige Supervision des multiprofessionellen PCT

- Die Teilnahme der PCT Mitglieder mindestens halbjährlich an regionalen multiprofessionellen Qualitätszirkeln

- Das Vorliegen eines Fort- und Weiterbildungskonzeptes für die Mitglieder des PCT

- Das Vorliegen einer kompletten Dokumentation gemäß den Ausführungen unter Anlage 2.

Lower Saxony

*Niedersachsen Stand 02.01.2010*

# Vertrag

## nach § 132 d Abs. 1 SGB V

## über die spezialisierte ambulante Palliativversorgung (SAPV) nach § 37 b SGB V

zwischen

**dem Träger des Krankenhauses X**
**dem Träger des Krankenpflegedienstes Y**
**der niedergelassenen Arztpraxis Z**
**als gemeinsamer Träger**

**für die Einrichtung „_________________“**

**IK-Nr. ________________**

- nachfolgend Leistungserbringer genannt -

und

**AOK - Die Gesundheitskasse für Niedersachsen*,**

**BKK Landesverband Mitte,**

**IKK Niedersachsen,**

**Landwirtschaftlichen Krankenkasse Niedersachsen-Bremen*,**

**Knappschaft - Regionaldirektion Hannover*,**

**BARMER GEK**

**Techniker Krankenkasse (TK)**

**Deutsche Angestellten-Krankenkasse (Ersatzkasse)**

**KKH-Allianz (Ersatzkasse)**

**HEK – Hanseatische Krankenkasse**

**hkk**

**gemeinsamer Bevollmächtigter mit Abschlussbefugnis:**

**Verband der Ersatzkassen e.V. (vdek), Berlin**

**vertreten durch den Leiter der Landesvertretung Niedersachsen**

* in Wahrnehmung der Aufgaben eines Landesverbandes

- nachfolgend Krankenkassen genannt -

## Präambel

(1) Die SAPV dient dem Ziel, die Lebensqualität und die Selbstbestimmung schwerstkranker Menschen zu erhalten, zu fördern und zu verbessern und ihnen ein menschenwürdiges Leben bis zum Tod in ihrer vertrauten häuslichen Umgebung oder in einer stationären Pflegeeinrichtung zu ermöglichen.

(2) Die SAPV ist fachlich kompetent nach anerkannten medizinischen und pflegewissenschaftlichen Erkenntnissen bedarfsgerecht und wirtschaftlich zu erbringen. Die individuellen Bedürfnisse und Wünsche der Patientin oder des Patienten sowie die Belange ihrer oder seiner vertrauten Person stehen im Mittelpunkt der Versorgung.

(3) Von besonderer Bedeutung bei der Erbringung von SAPV ist die vernetzte Zusammenarbeit aller Beteiligten. Zu integrieren sind nicht nur die klassischen medizinischen und pflegerischen Bereiche, sondern auch die Seelsorge, die psychosoziale Beratung und die ehrenamtlichen Dienste.

(4) Die SAPV ergänzt das bestehende Versorgungsangebot, insbesondere das der Vertragsärzte, Krankenhäuser und Pflegedienste, wenn eine besonders aufwändige Versorgung besteht und die anderweitigen ambulanten Versorgungsformen nicht ausreichen. Sie kann als alleinige Beratungsleistung, additiv unterstützende Teilversorgung oder vollständige Patientenversorgung erbracht werden.

(5) Die SAPV von Kindern und Jugendlichen bedarf einer ergänzenden Vereinbarung.

## § 1
## Gegenstand des Vertrages

(1) Gegenstand dieses Vertrages ist die Durchführung der SAPV gemäß § 37 b SGB V.

(2) Die Richtlinie des Gemeinsamen Bundesausschusses nach § 92 Abs. 1 Satz 2 Nr. 14 SGB V in Verbindung mit § 37 b Abs. 3 SGB V in der jeweils gültigen Fassung sowie die Gemeinsamen Empfehlungen der Spitzenverbände der Krankenkassen gemäß § 132 d Abs. 2 SGB V zu den Anforderungen an die Leistungserbringer für SAPV in der Fassung vom 23.06.2008 sind Bestandteil dieses Vertrages (s. Anlagen 1 und 2)

## § 2
## Allgemeine Anforderungen und Voraussetzungen

(1) Der Leistungserbringer ist eine auf Dauer angelegte selbständige organisatorische Zusammenfassung von Personen und Sachmitteln, mit denen unabhängig vom Bestand ihrer Mitarbeiter eine fachlich kompetente SAPV nach dem wissenschaftlich allgemein anerkannten Stand zu gewährleisten ist.

(2) Der Leistungserbringer erbringt sowohl pflegerische als auch ärztliche ambulante Palliativbehandlung sowie entsprechende Beratungs- und Koordinationsleistungen nach einem verbindlichen, strukturierten und schriftlich dargelegten Konzept.

(3) Der Leistungserbringer benennt einen Ansprechpartner für die Krankenkassen.

(4) Auf Wunsch des Leistungserbringers benennen die örtlich vertretenen Krankenkassen/ Landesverbände einen Ansprechpartner für die Leistungsgewährung.

## § 3
## Qualifikationsvoraussetzungen

(1) Der Leistungserbringer stellt sicher, dass das für die SAPV erforderliche und geeignete Personal zur Verfügung steht. Dabei ist zu berücksichtigen, dass durch den Leistungserbringer eine tägliche Rufbereitschaft für die Patienten, deren Angehörige sowie die an der Versorgung Beteiligten sicherzustellen ist. Die ständige Verfügbarkeit mindestens einer Ärztin / eines Arztes oder einer Pflegefachkraft nach Abs. 2 ist zu gewährleisten. Die Verfügbarkeit umfasst auch das zeitnahe Aufsuchen der Patienten je nach Dringlichkeit.

(2) Die Ärztinnen / Ärzte und Pflegefachkräfte des Leistungserbringers müssen über folgende Qualifikationen verfügen:

**Ärztinnen / Ärzte** verfügen über:

a) eine anerkannte Zusatzweiterbildung Palliativmedizin nach der je nach Bundesland aktuell gültigen Weiterbildungsordnung der jeweiligen Landesärztekammer (grds. 160 Std. Weiterbildung)

und

b) Erfahrung aus der ambulanten palliativen Behandlung von mindestens 75 Palliativpatienten, z. B. in der häuslichen Umgebung oder in einem stationären Hospiz, innerhalb der letzten drei Jahre. Regionale Abweichungen von der Fallzahl sind in Einzelfällen möglich.

oder

aus einer mindestens 1-jährigen klinischen palliativmedizinischen Tätigkeit, z. B. in einer Palliativabteilung in einem Krankenhaus, innerhalb der letzen drei Jahre.

**Pflegefachkräfte** verfügen über

a) die Erlaubnis zur Führung einer der Berufsbezeichnungen Gesundheits- und Krankenpflegerin / Gesundheits- und Krankenpfleger, Gesundheits- und Kinderkrankenpflegerin / Gesundheits- und Kinderkrankenpfleger oder Altenpflegerin / Altenpfleger entsprechend den gesetzlichen Bestimmungen in der jeweils gültigen Fassung

und

b) den Abschluss einer Palliative-Care-Weiterbildungsmaßnahme im Umfang von mindestens 160 Stunden und

c) Erfahrung durch mindestens eine 2-jährige praktische Tätigkeit als Pflegefachkraft in der Betreuung von Palliativpatienten in den letzten drei Jahren; davon grundsätzlich sechsmonatige Mitarbeit in einer spezialisierten Einrichtung der Hospiz- und Palliativversorgung.

(3) **Ärzte und Pflegefachkräfte**, die die Palliative-Care-Weiterbildung gemäß der Weiterbildungsordnung (160 Std.) abgeschlossen haben, können - sofern sie die weiteren Kriterien nach Absatz 2 noch nicht erfüllen - ambulante Palliativleistungen unter qualifizierter Praxisanleitung einer Mitarbeiterin / eines Mitarbeiters der entsprechenden unter Abs. 2 genannten Berufsgruppe des Leistungserbringers erbringen.

(4) **Fachkräfte weiterer Berufsgruppen**, können ihre Leistungen unter qualifizierter Praxisanleitung eines Arztes des Leistungserbringers mit Qualifikation gemäß Abs. 2 erbringen.

## § 4
## Sächliche und räumliche Mindestausstattung

(1) Die Leistungserbringer haben als Mindestanforderung an die sächliche Ausstattung Folgendes vorzuhalten bzw. sicherzustellen:

- eine geeignete, aktuell geführte und für die an der Versorgung Beteiligten jederzeit zugängliche Patientendokumentation,
- eine ausreichende und geeignete Mobilität zur zeitnahen häuslichen Versorgung der Patienten,
- Bedarfsgerecht bestückte Arzt- / Pflegekoffer / Bereitschaftstasche für den Akutfall und
- eine geeignete administrative Infrastruktur, u. a. Büro, Kommunikationstechnik.

(2) Der Leistungserbringer muss über eine eigene Postadresse und geeignete Räumlichkeiten für

- die Beratung von Patienten und Angehörigen,
- Teamsitzungen und Besprechungen sowie
- die Lagerhaltung von eigenen Medikamenten für Notfall- / Krisenintervention und Hilfsmitteln

verfügen.

## § 5
## Einzugsbereich

(1) Der Einzugsbereich umfasst die kreisfreie Stadt, Landkreis XXXXXX.

(2) Der Leistungserbringer stellt im Rahmen seiner Kapazitäten die Versorgung der Versicherten in seinem Einzugsbereich mit Leistungen der SAPV sicher. Hierzu können auch Anlaufstellen eingerichtet werden.

(3) Die Festlegung des Einzugsbereiches schließt den Abschluss von Verträgen mit anderen Leistungserbringern zur Versorgung der Versicherten mit SAPV im selben Einzugsbereich nicht aus.

(4) Der Versicherte ist in der Wahl des Leistungserbringers frei, soweit dieser zur Erbringung der SAPV berechtigt ist. Wählt er nicht einen der nächst erreichbaren Leistungserbringer, kann die Krankenkasse die Übernahme der hierdurch entstehenden Mehrkosten ablehnen.

## § 6
## Inhalt und Umfang der Leistungen

(1) SAPV besteht aus folgenden Bestandteilen:

- Steuerung der Verordnung gemäß Abs. 2 / Beratung sowie Koordination von Dritten gemäß Abs. 3,
- teilweise Übernahme der spezialisierten pflegerischen und / oder ärztlichen Palliativversorgung,

- vollständige Übernahme der spezialisierten pflegerischen und / oder ärztlichen Palliativversorgung.

(2) Der Leistungserbringer prüft im Rahmen des Eingangsassessments innerhalb von 3 Arbeitstagen nach Erhalt der Verordnung die Notwendigkeit und Eignung der besonderen Mittel der SAPV.

(3) Der Leistungserbringer prüft, ob eine Leistung selbst oder durch Kooperationspartner erbracht werden kann. Er beauftragt eventuell erforderliche Kooperationspartner.

(4) Die Beratung beinhaltet Gespräche mit anderen Leistungserbringern wie Ärzten und Krankenpflegediensten, Angehörigen und Patienten. Koordination meint die Vermittlung an geeignete dritte Leistungserbringer, aber auch die Beantragung von Hilfsmitteln, die Vermittlung spiritueller Betreuung etc. Beratung und Koordination werden weitmöglichst von Pflegefachkräften, bei Bedarf von Ärzten erbracht.

(5) Bestandteil der SAPV ist ebenfalls die Versorgung mit Arznei-, Heil- und Hilfsmitteln (einschließlich Betäubungsmitteln) im Rahmen einer Versorgung im akuten Krisenfall.

(6) Bestandteil der SAPV ist ebenfalls die spezialisierte ärztliche Teilleistung, nicht jedoch die pflegerische Palliativversorgung in stationären Hospizen, soweit sie nicht im Rahmen der vertragsärztlichen Versorgung erbracht werden kann. SAPV Leistungen in stationären Pflegeeinrichtungen nach § 72 Abs. 1 SGB XI sind Bestandteil dieses Vertrages.

(7) Von den SAPV-Leistungserbringern und deren Kooperationspartnern nach §§ 7a – 7b dürfen bei vollständiger Übernahme der spezialisierten pflegerischen und / oder ärztlichen Palliativversorgung zeitgleich keine komplementären Leistungen abgerechnet werden. Die zusätzliche Abrechnung von Leistungen nach den §§ 37, 37 a, 43 Abs. 2 SGB V ist demnach ausgeschlossen. Auch die in Anlage 3 genannten EBM-Positionen dürfen nicht zusätzlich zur SAPV abgerechnet werden. Leistungen, die nach dem SGB XI abgerechnet werden, bleiben von der SAPV unberührt.

## § 7
## Kooperationen

(1) Die Leistungserbringer sind Teil einer vernetzten Versorgungsstruktur im regionalen Gesundheits- und Sozialsystem, die eng mit den anderen an der palliativmedizinischen Versorgung beteiligten Vertragspartnern zusammenarbeiten. Dies gilt sowohl für Palliativeinrichtungen wie Hospize und Krankenhäuser mit Palliativstationen, aber auch für andere Leistungsanbieter im Gesundheitsmarkt wie Krankenpflegedienste, Ärzte, psychologische Psychotherapeuten, Kinder- und Jugendpsychotherapeuten, Apotheken sowie begleitende Angebote wie Seelsorge und ambulante Hospizdienste. Bestehende und abzuschließende Kooperationsvereinbarungen können von den Krankenkassen eingefordert werden. Sie erhalten jährlich zum 30.11. EDV-lesbare Listen der aktuellen Kooperationspartner nach § 7 a-c.

(2) Soweit Fachkräfte und / oder Institutionen, die in §§ 7a bis 7c nicht genannt werden, in die SAPV vertraglich eingebunden werden, haben diese eine Zusatzweiterbildung Palliative Care für andere Berufsgruppen oder eine mehrjährige Erfahrung in der Palliativversorgung nachzuweisen.

## § 7a
## Kooperation mit Krankenpflegediensten

(1) Der Leistungserbringer prüft, ob eine SAPV-Pflegeleistung teilweise oder vollständig durch einen Krankenpflegedienst durchgeführt werden kann und beauftragt diesen mit der Durchführung der SAPV–Leistung.

(2) Kooperierende Krankenpflegedienste müssen mindestens folgende Voraussetzungen erfüllen:

1. Zulassung als Krankenpflegedienst nach § 132 a Abs. 2 SGB V,
2. Bereitschaft, Palliativpatienten zu betreuen und
3. mindestens drei fest angestellte Pflegefachkräfte mit Abschluss einer Palliative-Care-Weiterbildungsmaßnahme im Umfang von mindestens 160 Stunden.

(3) In einem Kooperationsvertrag wird die Zusammenarbeit zwischen Leistungserbringer und kooperierendem Krankenpflegedienst festgeschrieben. Bestandteil sind u. a. die Einhaltung der Qualitätsanforderungen, die Rechnungslegung im Innenverhältnis und die Weitergabe der Vergütungsanteile gemäß § 10.

## § 7b
## Kooperation mit niedergelassenen Ärzten

(1) Der Leistungserbringer prüft, ob eine ärztliche Behandlung im Rahmen der SAPV teilweise oder vollständig durch eine niedergelassene Ärztin / einen niedergelassenen Arzt mit erbracht werden kann und beauftragt diesen ggf. mit der Durchführung der SAPV-Leistung.

(2) Kooperierende Ärzte müssen mindestens folgende Voraussetzungen erfüllen:

1. Zulassung zur vertragsärztlichen Versorgung,
2. Bereitschaft, Palliativpatienten zu betreuen und
3. Weiterbildung „Kursweiterbildung Palliativmedizin“ (40 Stunden).

(3) In einem Kooperationsvertrag wird die Zusammenarbeit zwischen Leistungserbringer und kooperierender Ärztin / kooperierendem Arzt festgeschrieben. Bestandteil sind u. a. die Einhaltung der Qualitätsanforderungen, die Rechnungslegung im Innenverhältnis und die Weitergabe der Vergütungsanteile gemäß § 10.

## § 7c
## Kooperation mit Apotheken / Arzneimittelversorgung im Notfall

(1) Der Leistungserbringer kooperiert mit einer oder mehreren Apotheken, um eine Lagerhaltung an Medikamenten für den Notfall sicherzustellen.

(2) Kooperierende Apotheken müssen mindestens folgende Voraussetzungen erfüllen:

1. Erlaubnis zum Betrieb einer Apotheke,
2. Vorhaltung von palliativmedizinisch relevanten Arzneimitteln sowie
3. möglichst Fortbildung zum Fachapotheker für Offizin- oder onkologische Pharmazie sowie möglichst Kursweiterbildung Palliativmedizin / Palliative Care (40 Stunden).

## § 8
## Qualitätssicherung

(1) Der Leistungserbringer ist verpflichtet, ein internes Qualitätsmanagement durchzuführen. Er beteiligt sich ferner an Maßnahmen der externen Qualitätssicherung.

(2) Ein geeignetes Dokumentationssystem ist sachgerecht und kontinuierlich zu führen und auf Anforderung dem MDK vorzulegen. Das Dokumentationssystem muss patientenbezogene Daten und - soweit vorhanden - allgemein anerkannte Indikatoren für eine externe Qualitätssicherung enthalten und eine bundesweite Evaluation ermöglichen. Es soll so weit wie möglich kompatibel zu den bestehenden Dokumentationssystemen der Netzwerkpartner gemäß Kerndatensatz für Palliativpatienten der Deutschen Gesellschaft für Palliativmedizin (DGP) und des Deutschen Hospiz- und Palliativverbandes (DHPV) in seiner jeweils gültigen Fassung bestehend aus Teil 1 Kerndatensatz für Palliativpatienten und Teil 2 Modul SAPV Struktur sein.

(3) Durch den Leistungserbringer sind bedarfsgerechte multiprofessionelle Fallbesprechungen und Netzwerktreffen durchzuführen. Er nimmt regelmäßig an fachspezifischen und sektorübergreifenden regionalen Besprechungen zur Erhöhung der Qualität teil, an denen möglichst alle anderen an der Patientenversorgung Beteiligten mitwirken.

(4) Der Leistungserbringer ist verpflichtet, den Krankenkassen wesentliche Änderungen der vertraglichen Voraussetzungen unverzüglich schriftlich mitzuteilen. Dies betrifft insbesondere:

- Trägerwechsel
- Änderung der Anschrift
- Betriebsaufgabe

Die Krankenkasse ist berechtigt, die Voraussetzungen nach §§ 2-7, § 8 Abs. 1 und 3 sowie § 9 zu prüfen, ggf. auch in den Räumlichkeiten der Leistungserbringer.

(5) Die Kooperationspartner nach §§ 7ff. sollen in alle Maßnahmen zur Qualitätssicherung eingebunden werden.

## § 9
## Fort- und/oder Weiterbildung

(1) Der Leistungserbringer ist verpflichtet, auf die Fachlichkeit aller unter § 3 beschäftigten spezialisierten Leistungserbringer, die länger als ein Jahr beim Leistungserbringer tätig sind, durch regelmäßige Teilnahme an berufs- bzw. aufgabenbezogenen, internen und externen Fort- und / oder Weiterbildungsmaßnahmen hinzuwirken.

Die Maßnahmen sollen insbesondere den aktuellen palliativmedizinischen / -pflegerischen Stand vermitteln und nicht ausschließlich als interne Schulungen aller Beschäftigten durchgeführt werden. Fachbezogene Literatur ist vorzuhalten.

(2) Der Umfang der Fort- und / oder Weiterbildungen beträgt durchschnittlich 20 Stunden pro Vollzeitstelle innerhalb von zwei Kalenderjahren.

## § 10
## Vergütung

(1) Die Vergütung erfolgt in Form von Pauschalen. Die konkrete Höhe der Vergütung ist in Anlage 4 geregelt.

(2) Für die Erstberatung enthält der Leistungserbringer einmalig pro Leistungsfall eine Eingangspauschale: Pauschale E.

(3) Für die Beratung und Koordination durch die Pflegekraft und / oder den Arzt (gemäß § 3) erhält der Leistungserbringer kalendertäglich die Pauschale B. Die Berechnung für einen Patienten erfolgt kalendertäglich und wird für die gesamte Dauer der Betreuung fällig. Die parallele Erbringung pflegerischer und ärztlicher Leistungen ist dafür nicht zwingend erforderlich. Die Pauschale B beinhaltet neben dem Aufwand für die Steuerung der Verordnung gemäß § 6 Abs. 1 und für die Weiterleitung von Vergütungsanteilen an Kooperationspartner auch Aufwendungen für die Vorhaltung der palliativärztlichen bzw. -pflegerischen Bereitschaft.

(4) Für die teilweise oder vollständige Übernahme der ambulanten palliativmedizinischen Pflege erhält der Leistungserbringer folgende Pauschalen:

- Umfangreiche Behandlungspflege inkl. Schmerzprotokolle etc. (Aufwand: bis zu 30 Minuten):
  Pauschale P1
- inkl. parenterale Ernährung oder Kurzinfusion (Aufwand: 31 bis 50 Minuten): Pauschale P2
- inkl. parenterale Ernährung und Kurzinfusionen (Aufwand: über 50 Minuten): Pauschale P3.

Eine Berechnung für einen Patienten kann maximal dreimal täglich erfolgen. Eine parallele Abrechnung der häuslichen Krankenpflege nach § 37 SGB V während des selben palliativmedizinischen Pflegeeinsatzes (P1 bis P3) ist durch denselben Pflegeanbieter nicht möglich.

(5) Für die teilweise oder vollständige Übernahme der ambulanten palliativmedizinischen ärztlichen Versorgung erhält der Leistungserbringer folgende Pauschalen:

- Arztbesuch im Regelfall (Aufwand: 16 bis 70 Minuten): Pauschale A1
- Arztbesuch mit erheblichem Aufwand (Aufwand: über 70 Minuten): Pauschale A2

In der Woche sind maximal sieben Einsätze abrechenbar, mehrere Einsätze an einem Tag sind möglich.

(6) Sämtliche Vergütungen beinhalten den notwendigen Verwaltungsaufwand, u. a. Vorbereitungs-, Nachbereitungs- und Dokumentationsaufwand, die Übersendung der Dokumentation oder Teilen der Dokumentation für die Mitwirkungspflicht nach § 276 Abs. 1 SGB V, Aufwand für den Datenträgeraustausch, Qualitätssicherung, Leistung nachts und an Wochenenden sowie gesetzlichen Feiertagen, Fahrtkosten sowie die Wegezeiten.

(7) Die Abrechnung erfolgt einheitlich für alle Institutionen, die gemeinsam die SAPV erbringen, sowie für alle Kooperationspartner ausschließlich durch eine vom Leistungserbringer zu benennende Abrechnungsstelle.

## § 11
## Verordnung und Entscheidung über die Leistungserbringung

(1) Für die Erbringung der SAPV ist eine Verordnung durch niedergelassene Vertrags- oder Krankenhausärzte sowie eine Kostenübernahmeerklärung der Krankenkasse erforderlich.

(2) Für die Verordnung wird das Muster der Vordruckkommission (Nr. 63) eingesetzt. Die Verordnung wird zunächst dem SAPV-Leistungserbringer vorgelegt. Er wirkt darauf hin, dass die Diagnose deutlich lesbar, umfassend und in der ICD-10-Systematik verschlüsselt auf der Verordnung vermerkt wird. Spätestens nach drei Arbeitstagen wird die Verordnung der zuständigen Krankenkasse einschließlich der Angaben zu den vorgesehenen Pauschalen nach § 10 (vgl. Anlage 5) vorgelegt.

(3) Für Verordnungen von Arznei-, Heil- und Hilfsmitteln durch Ärzte des Leistungserbringers sind ausschließlich die im Rahmen der vertragsärztlichen Versorgung vereinbarten Vordrucke (Muster 13, 14, 16 und 18) zu verwenden. Bei der zeitgleichen Verordnung von Arzneimitteln einerseits und Hilfsmitteln andererseits sind jeweils getrennte Vordrucke nach Muster 16 zu verwenden. Auf den Verordnungen ist die von der KBV vergebene SAPV-Betriebsstätten-Nummer (SAPV-BSNR) sowie die Pseudo-Arzt-Nummer 333333300 anzugeben. Die SAPV-BSNR und Pseudo-Arzt-Nummer dürfen nur für Verordnungen im Rahmen dieses SAPV-Vertrages auf Basis einer gültigen SAPV-Verordnung verwendet werden. Die Kosten für die Bearbeitung und Vergabe der SAPV-BSNR sind durch den antragstellenden Leistungserbringer zu tragen.

(4) Die Krankenkasse übernimmt bis zu einer Entscheidung über die weitere Leistungserbringung die Kosten für die verordneten und von den Leistungserbringern nach § 132 d SGB V erbrachten Leistungen entsprechend der vereinbarten Vergütung nach § 10, wenn die Verordnung gemäß Abs. 2 spätestens an dem dritten der Ausstellung folgenden Arbeitstag der Krankenkasse vorgelegt wird.

## § 12
## Rechnungslegung, Zahlungsfrist, Beanstandung, Verjährung

(1) Für Inhalt und Form der Abrechnung gelten die Richtlinien des Spitzenverbandes Bund nach § 302 Abs. 2 SGB V für sonstige Leistungserbringer in der jeweils gültigen Fassung.

(2) Die Rechnung muss folgende Mindestangaben enthalten (vgl. Anlage 6):

- Name, Anschrift, Unterschrift und Institutionskennzeichen des SAPV-Leistungserbringers,
- Name, Vorname, Geburtsdatum und Krankenversicherungsnummer des Leistungsbeziehers,
- Diagnoseverschlüsselung nach ICD-10,
- Art und Anzahl der kalendertäglich tatsächlich erbrachten Leistungen,
- Tag und Uhrzeit des Leistungsbeginns,
- Tag und Uhrzeit des Leistungsendes bzw. Dauer der Leistungserbringung,
- Angabe der beteiligten Kooperationspartner.

(3) Zahlungen an eine durch den Leistungserbringer ermächtigte Abrechnungsstelle / Verrechnungsstelle setzen voraus, dass der Krankenkasse eine Ermächtigungserklärung vorliegt. Zahlungen an eine Abrechnungsstelle erfolgen mit schuldbefreiender Wirkung, es sei denn, der Krankenkasse liegt ein schriftlicher Widerruf des Leistungserbringers vor.

(4) Die Abrechnung ist versichertenbezogen einmal monatlich unter Beifügung der Verordnungen zu erstellen und bei den von den Krankenkassen benannten Daten- und Papier-

annahmestellen einzureichen. Die Mindestinhalte ergeben sich aus Anlage 6. Die Krankenkassen sind zur Rechnungsbegleichung nur verpflichtet, sofern eine versicherungs-, leistungs- und vertragsrechtliche Anspruchsberechtigung gegeben ist. Die Abrechnung unter dem Namen des Zugelassenen ist mit dem speziellen SAPV-IK zu versehen und als solche deutlich zu kennzeichnen. Die Zahlung sämtlicher Vergütungsbestandteile gemäß § 10 erfolgt mit schuldbefreiender Wirkung an den Leistungserbringer. Die Umverteilung der einzelnen Vergütungsbestandteile an Kooperationspartner obliegt dem Leistungserbringer.

(5) Werden Nachberechnungen gestellt, sind der Nachberechnung Kopien der Erstrechnung und der Verordnung beizulegen. Darauf ist "Nachberechnung" und das Datum der Bezahlung der Erstrechnung zu vermerken.

(6) Maßgeblich für die Berechnung des Zahlungsziels ist der Tag, an dem alle nach Abs. 2 zu einem Abrechnungsfall gehörenden Unterlagen bei der Krankenkasse vorliegen. Zusammengehörige Rechnungsunterlagen müssen in unmittelbarem zeitlichem Zusammenhang angeliefert werden. Die Krankenkasse behält sich vor, nicht korrekt gestellte Rechnungen unbezahlt abzuweisen.

(7) Die Rechnungen sind innerhalb von 21 Tagen nach Eingang bei der Krankenkasse zu begleichen. Als Zahltag gilt der Tag der Überweisung oder Übersendung von Zahlungsmitteln oder der Tag der Übergabe des Überweisungsauftrages an ein Geldinstitut. Bei Abrechnung mittels DTA verkürzt sich die Zahlungsfrist auf 14 Tage.

(8) Beanstandungen müssen von der Krankenkasse innerhalb von 12 Monaten nach Rechnungseingang erhoben werden.

(9) Forderungen aus Vertragsleistungen können nach Ablauf eines Jahres, gerechnet vom Ende des Monats, in dem sie abgeschlossen worden sind, nicht mehr erhoben werden.

## § 13
## Zuzahlungen

(1) SAPV wird als Sachleistung zur Verfügung gestellt. Jegliche Zahlungen vom/von Versicherten im Rahmen der Vertragsleistungen dürfen weder gefordert noch angenommen werden.

(2) Zuzahlungen dürfen auch in dem Fall nicht verlangt werden, wenn die Verordnung bei der Krankenasse verspätet eingereicht wird, da Kosten erst ab Eingang der Verordnung bei der Krankenkasse gemäß § 11 dieses Vertrages übernommen werden.

## § 14
## Datenschutz und Schweigepflicht

(1) Die Vertragsparteien sind verpflichtet, die Bestimmungen über den Schutz der Sozialdaten (SGB X, 2. Kapitel) zu beachten, insbesondere personenbezogene Daten nur zur Erfüllung der sich aus diesem Vertrag ergebenden Aufgaben zu verarbeiten, bekannt zu geben, zugänglich zu machen oder zu nutzen.

(2) Der Leistungserbringer unterliegt hinsichtlich der Person des Versicherten und dessen Krankheit der Schweigepflicht. Ausgenommen hiervon sind Angaben gegenüber der leistungspflichtigen Krankenkasse, soweit sie zur Erfüllung der gesetzlichen Aufgaben der Krankenkasse erforderlich sind.

(3) Der Leistungserbringer hat seine Mitarbeiter zur Beachtung der Datenschutzbestimmungen und der Schweigepflicht zu verpflichten.

## § 15
## Außerordentliche Kündigung

(1) Das Vertragsverhältnis kann von den Krankenkassen bzw. einer einzelnen Krankenkasse ohne Einhaltung einer Kündigungsfrist gekündigt werden, wenn der Leistungserbringer seine gesetzlichen oder vertraglichen Pflichten gegenüber den Versicherten oder den Krankenkassen derart gröblich verletzt, dass ein Festhalten an dem Vertag nicht zumutbar ist. Eine gröbliche Pflichtverletzung in diesem Sinne liegt insbesondere vor bei:

   a) fortgesetzter Nichterfüllung wesentlicher fachlicher und qualitativer Voraussetzungen,

   b) wiederholter Abrechnung von nicht erbrachten Leistungen,

   c) Forderung bzw. Annahme von Zahlungen oder sonstiger geldwerter Vorteile von den Versicherten für Leistungen nach diesem Vertrag.

(2) Des Weiteren haben die Krankenkassen das Recht zur außerordentlichen Kündigung, wenn der Leistungserbringer zahlungsunfähig ist oder über sein Vermögen das Gesamtvollstreckungs- bzw. Insolvenzverfahren eröffnet ist.

## § 16
## Inkrafttreten, Kündigung

(1) Dieser Vertrag einschließlich seiner Anlagen tritt am _________ in Kraft. Der Vertrag kann von den Vertragspartnern mit einer Frist von drei Monaten zum Quartalsende gekündigt werden.
(2) Die Kündigung durch eine Krankenkasse berührt nicht die Gültigkeit des Vertrages zwischen den verbliebenen Vertragsbeteiligten.
(3) Die Anlage 4 kann eigenständig mit einer Frist von drei Monaten zum Quartalsende gekündigt werden, frühestens zum ______________*(31.12.2010)*.

(4) Einvernehmlich vereinbarte Änderungen dieses Vertrages in schriftlicher Form sind möglich, ohne dass es einer Kündigung bedarf.

## § 17
## Salvatorische Klausel

(1) Sollten einzelne Bestimmungen dieses Vertrages nichtig sein oder durch gesetzliche Neuregelungen ganz oder teilweise unwirksam werden, so wird hierdurch die Wirksamkeit dieses Vertrages im Übrigen nicht berührt. Tritt ein solcher Fall ein, verständigen sich die Vertragspartner unverzüglich über notwendige Neuregelungen.

(2) Bei Änderung der Richtlinien des Gemeinsamen Bundesausschusses nach § 92 Abs. 1 Satz 2 Nr. 14 SGB V in Verbindung mit § 37 b Abs. 3 SGB V oder der Gemeinsamen Empfehlungen nach § 132 d Abs. 2 SGB V (*vgl. § 1 Abs. 2*) prüfen die Vertragspartner, ob dieser Vertrag anzupassen ist.

(3) Änderungen oder Ergänzungen dieses Vertrages bedürfen zu ihrer Wirksamkeit der Schriftform.

_____________, den ___________________

______________________________
Einrichtungsträger

______________________________
AOK – Die Gesundheitskasse für Niedersachsen
Zugleich handelnd für den BKK Landesverband Mitte, Landesvertretung Niedersachsen

______________________________
Knappschaft, Regionaldirektion Hannover

______________________________
IKK Niedersachsen

______________________________
Landwirtschaftliche Krankenkasse Niedersachsen -Bremen

______________________________
Verband der Ersatzkassen e. V. ( vdek)
- Der Leiter der Landesvertretung Niedersachsen -

**Ergänzung zum Vertrag bzw. Nebenabsprache**

Übergangsregelungen sind in palliativmedizinisch noch nicht ausreichend entwickelten Regionen nach Vorlage des Versorgungskonzeptes zeitlich begrenzt in Einzelfällen möglich. Innerhalb von 2 Jahren müssen die Strukturen die Anforderungen der §§ 3,4,7 und 7a-c voll erfüllt sein.

Die Krankenkassen beziehen bei der Auswahl ihrer Vertragspartner die bereits bestehenden Strukturen, insbesondere die Palliativ-Stützpunkte, so weit wie möglich ein. Auf die bereits tätigen Leistungserbringer ist zurückzugreifen, soweit sie die vertraglichen Voraussetzungen erfüllen oder im Rahmen einer Übergangsregelung erfüllen werden.

**Anlagen zum Vertrag**

| | |
|---|---|
| **Anlage 1:** | **Richtlinien des Gemeinsamen Bundesausschusses nach § 92 Abs. 1 Satz 2 Nr. 14 SGB V in Verbindung mit § 37 b Abs. 3 SGB V** |
| **Anlage 2:** | **Gemeinsame Empfehlung der Spitzenverbände der Krankenkassen gemäß § 132 d Abs. 2 SGB V** |
| **Anlage 3:** | **Abrechnungsausschluss vertragsärztliche Leistungen (EBM-Ziffern)** |
| **Anlage 4:** | **Vergütung** |
| **Anlage 5:** | **Anlage zum Verordnungsformular** |
| **Anlage 6:** | **Abrechnungsformular** |

Mecklenburg-Western Pomerania

Muster

# Regionalvertrag - Region Versorgungsbereich

**über die**
**spezialisierte ambulante Palliativversorgung**
**(nachstehend SAPV genannt)**

**gemäß § 37 b i.V.m. § 132 d SGB V**

**zwischen**

**„... GbR"**
**(nachstehend SAPV Team genannt)**
**- AC/ TK ... -**

**und**

**Kassenärztliche Vereinigung Mecklenburg-Vorpommern**
**(nachstehend KVMV genannt)**

**und**

**AOK Nordost**
**Die Gesundheitskasse.**
**handelnd als Landesverband**

**BKK-Vertragsarbeitsgemeinschaft NORD**

**IKK Nord**
**in Vertretung des IKK-Landesverbandes Nord**

**Landwirtschaftliche Krankenkasse Mittel- und Ostdeutschland,**
**handelnd als Landesverband**

**Knappschaft**
**handelnd als Landesverband**

**sowie den nachfolgenden Ersatzkassen in Mecklenburg-Vorpommern:**
- Barmer GEK
- Techniker Krankenkasse (TK)
- Deutsche Angestellten-Krankenkasse (Ersatzkasse)
- KKH-Allianz (Ersatzkasse)
- HEK – Hanseatische Krankenkasse
- hkk

**gemeinsamer Bevollmächtigter mit Abschlussbefugnis**
**Verband der Ersatzkassen e.V. (vdek),**
**vertreten durch den Leiter der Landesvertretung Mecklenburg-Vorpommern**

(nachfolgend Krankenkassen)

- andererseits -

Muster

## Inhaltsverzeichnis


| | | |
|---|---|---|
| § 1 | Gegenstand und Ziele des Vertrages[1] | Seite 4 |
| § 2 | Geltungsbereich | Seite 4 |
| § 3 | Anspruchsberechtigte Personen | Seite 5 |
| § 4 | Versorgungskonzept | Seite 5 |
| § 5 | Anzeige- und Mitteilungspflichten | Seite 6 |
| § 6 | Verordnung der spezialisierten ambulanten Palliativversorgung | Seite 6 |
| § 7 | Prüfung der Leistungsansprüche durch die Krankenkassen | Seite 7 |
| § 8 | Inhalt und Umfang der spezialisierten ambulanten Palliativversorgung | Seite 7 |
| § 9 | Zuzahlungen | Seite 8 |
| § 10 | SAPV-Team | Seite 8 |
| § 11 | Verordnung von Arzneimittel im Rahmen der SAPV | Seite 9 |
| § 12 | Netzwerk der regionalen palliativmedizinischen Versorgung | Seite 10 |
| § 13 | Zusammenarbeit mit Krankenhäusern | Seite 10 |
| § 14 | Dokumentation | Seite 11 |
| § 15 | Datenschutz | Seite 12 |
| § 16 | Vergütung | Seite 12 |
| § 17 | Qualitätssicherung | Seite 13 |
| § 18 | Abrechnung/Beanstandung/Verjährung | Seite 13 |
| § 19 | Institutionskennzeichen | Seite 14 |
| § 20 | Vertragsbeirat | Seite 14 |
| § 21 | Statistik | Seite 14 |
| § 22 | Vertragsverletzung | Seite 15 |
| § 23 | Inkrafttreten/Kündigung | Seite 15 |
| § 24 | Änderungen und Ergänzungen | Seite 16 |
| § 25 | Salvatorische Klausel | Seite 16 |


[1] Im vorliegenden Vertrag wird vornehmlich die männliche Person als Bezeichnung verwandt. Selbstverständlich ist dabei auch immer das weibliche Geschlecht gemeint.

Muster

**Anlagen** **Seite**


| | | |
|---|---|---|
| Anlage 1 | Versorgungskonzept | 18 |
| Anlage 2 | Verordnungsvordruck<br>Muster 63 - Verordnung Spezialisierter ambulanter Palliativversorgung | 19 |
| Anlage 3 | Leistungen des SAPV-Teams | 20 |
| Anlage 4 | Vergütung (Vergütungsvereinbarung) | 25 |
| Anlage 5 | Abrechnungsspezifische Besonderheiten der Krankenkassen | 29 |
| Anlage 6 | Anforderungen an die Dokumentation | 35 |
| Anlage 7 | Leistungsnachweise | 37 |
| Protokollnotiz | | 38 |

## § 1
## Gegenstand und Ziele des Vertrages

(1) Bei der SAPV handelt es sich um eine interdisziplinäre Aufgabe, die nur dann bedarfsgerecht erbracht werden kann, wenn die spezialisierten Leistungserbringer Teil einer multiprofessionell vernetzten Versorgungsstruktur im regionalen Gesundheits- und Sozialsystem sind. Sie arbeiten mit den an der Versorgung beteiligten ambulanten und stationären Leistungserbringern wie z.B. Haus-/ Fachärzten, Psychologen, Pflegediensten, Seelsorgern, Sozialarbeitern, Physiotherapeuten, Ehrenamtlichen, stationären Hospizen und ambulanten Hospizdiensten bspw. in Form von Kooperationen eng zusammen.

(2) Die „Richtlinie des Gemeinsamen Bundesausschusses zur Verordnung von spezialisierter ambulanter Palliativversorgung“ (Spezialisierte Ambulante Palliativversorgungs-Richtlinie/SAPV-RL) vom 20. Dezember 2007 und die „Gemeinsamen Empfehlungen der Spitzenverbände der Krankenkassen nach § 132 d Abs.2 SGB V für die spezialisierte ambulante Palliativversorgung“ vom 23.06.2008 sind in ihrer jeweiligen aktuellen Fassung Bestandteil dieses Vertrages.

(3) Gegenstand des Vertrages ist die spezialisierte ambulante palliativmedizinisch und -pflegerische Versorgung und deren Koordination durch eine multiprofessionelle Versorgungsstruktur. Dieser Vertrag schafft die Basis für eine kooperative interdisziplinäre Versorgung, um Patienten mit einer begrenzten Lebenserwartung die letzte Phase ihres Lebens in häuslicher Umgebung zu ermöglichen.

(4) Im Vordergrund steht anstelle eines kurativen Ansatzes die palliativmedizinisch/pflegerische Zielsetzung, Symptome und Leiden einzelfallgerecht zu lindern.

(5) Die individuellen Wünsche und Bedürfnisse der Patienten sowie die Belange seiner vertrauten Personen stehen im Mittelpunkt der Versorgung. Der Patientenwille, der auch durch Patientenverfügungen zum Ausdruck kommen kann, ist zu beachten. Die SAPV ergänzt das bereits bestehende Versorgungsangebot, insbesondere das der in Absatz 1 genannten Leistungserbringer. Sie kann als alleinige Beratungsleistung, Koordination, additiv unterstützende Teilversorgung oder vollständige Patientenbetreuung erbracht werden. Andere Sozialleistungsansprüche bleiben unberührt.

(6) Weitere Ziele des Vertrages sind:

- die Entwicklung einer individuellen ambulanten Versorgung mit entsprechender palliativmedizinischer und -pflegerischer Infrastruktur im Vertragsbereich,
- die Vermeidung von regionalen Parallelvorhaltungen und Überkapazitäten.
- bei Notwendigkeit einer Krankenhauseinweisung ist die direkte Einweisung unter Umgehung der Notfallaufnahme/Rettungsstelle auf eine vorhandene Palliativ-Station im Rahmen freier Kapazitäten vorzunehmen.

## § 2
## Geltungsbereich

(1) Diese Vereinbarung dient der bedarfsgerechten Versorgung der anspruchsberechtigten Versicherten der teilnehmenden Krankenkassen mit spezialisierter ambulanter Palliativversorgung in dem aus dem Versorgungskonzept hervorgehenden Versorgungsgebiet.

(2) Die BKK-Vertragsarbeitsgemeinschaft NORD vertritt die teilnehmenden Betriebskrankenkassen. Die Geschäftsstelle der VAG stellt dem Palliativmedizinischem Netz zu Beginn und bei Änderungen eine Liste der teilnehmenden Betriebskrankenkassen zur Verfügung.

Muster

Muster

## § 3
## Anspruchsberechtigte Personen

(1) Versicherte haben gemäß der SAPV Richtlinie Anspruch auf die SAPV, wenn

1. sie an einer nicht heilbaren, fortschreitenden und so weit fortgeschrittenen Erkrankung leiden, dass dadurch ihre Lebenserwartung deutlich begrenzt ist.

2. sie unter Berücksichtigung der in der SAPV-Richtlinie genannten Ziele eine besonders aufwendige Versorgung (§ 4 SAPV-Richtlinie) benötigen, die nach den medizinischen und pflegerischen Erfordernissen in der vertrauten Umgebung des häuslichen oder familiären Bereichs erbracht werden kann.

(2) Liegt ein besonderer Versorgungsbedarf nach Abs. 1 nicht vor, besteht kein Anspruch auf die SAPV.

(3) Der Anspruch des Versicherten auf die Leistungen dieses Vertrages ruht, wenn eine zwischenzeitliche Aufnahme in ein Krankenhaus erfolgt.

(4) Im Falle der Besserung bzw. Stabilisierung im Krankheitsverlauf ist der Versorgungsumfang der SAPV soweit wie möglich zu reduzieren und eine Weiterversorgung im Rahmen der allgemeinen Palliativversorgung vorzunehmen. Soweit sich der Krankheitsverlauf wieder verschlechtert, sind die Anspruchsvoraussetzungen gem. Abs. 1 erneut zu prüfen.

## § 4
## Versorgungskonzept

(1) Das SAPV-Team handelt auf der Grundlage eines verbindlichen, strukturierten und schriftlich dargelegten Versorgungskonzeptes. Das Versorgungskonzept formuliert alle medizinisch-pflegerischen Maßnahmen klar und eindeutig unter Beachtung von Maßnahmen zur Qualitätssicherung. Das Versorgungskonzept muss darüber hinaus verdeutlichen, wie eine qualifizierte Leistungserbringung zu erfolgen hat, damit die in § 1 des Vertrages genannten Ziele erreicht werden können. Das Versorgungskonzept ist Bestandteil des Vertrages gem. Anlage 1.

(2) Gegenstand des Versorgungskonzeptes sind die insbesondere nachfolgend genannten Inhalte:

- Versorgungsgebiet,
- Versorgungsprozess,
- Kooperationspartner,
- Zuständig- und Verantwortlichkeiten,
- Mitarbeiterqualifikationen,
- sächliche Ausstattung,
- Qualitätssicherungsmaßnahmen,
- Mitarbeiterkapazität (z. B. Stellenplan),
- Standort,
- Sicherstellung der 24-Stunden-Rufbereitschaft,
- Schriftliche Kooperationsvereinbarungen mit den regelhaft an der allgemeinen und spezialisierten Palliativversorgung beteiligten Leistungserbringern,
- Konzept zur Sicherstellung der reibungslosen Versorgung mit Arznei-, Heil- und Hilfsmitteln

(3) Das SAPV-Team trägt die alleinige Verantwortung für das Versorgungskonzept.

Muster

(4) Die Leistungen der SAPV müssen ausreichend und zweckmäßig sein und dürfen das Maß des Notwendigen nicht überschreiten.

(5) Die Leistungserbringung ist in Form geeigneter Maßnahmen unter Einsatz entsprechender personeller und sachlicher Mittel für die Dauer des Vertrages sicherzustellen. Regelungen zur Information, Kommunikation, personellen Zuständig- und Verantwortlichkeiten (u. a. Weisungsbefugnisse, -gebundenheit) sind zu treffen.

## § 5
## Anzeige- und Mitteilungspflichten

Das SAPV-Team ist grundsätzlich verpflichtet, den Krankenkassen unverzüglich alle wesentlichen Umstände schriftlich mitzuteilen, die die vertragsgemäße Durchführung dieses Vertrages sowie dessen Fortbestand berühren. Unverzüglich anzuzeigen sind hiernach insbesondere:

- jede Änderung der betrieblichen Gegebenheiten im organisatorischen und/oder personellen Bereich,
- der Abschluss/die Kündigung bzw. Änderungen von Kooperationsvereinbarungen,
- Änderungen der Trägerschaft/Betriebsinhaberschaft sowie Rechtsform des SAPV-Teams,
- die Einstellung des Netzwerkes,
- eingeleitete polizeiliche oder staatsanwaltliche Ermittlungsverfahren gegen einen Netzwerkpartner.

## § 6
## Verordnung der spezialisierten ambulanten Palliativversorgung

(1) SAPV wird vom behandelnden Vertragsarzt oder durch den Krankenhausarzt nach Maßgabe der SAPV-Richtlinie verordnet.

(2) Art, Umfang und Dauer der vom SAPV-Team zu erbringenden Leistungen ergeben sich aus der von der Krankenkasse genehmigten Verordnung des behandelnden Vertragsarztes oder des Krankenhausarztes.

(3) Die Verordnung erfolgt auf dem Vordruckmuster 63 (Anlage 2) und ist nicht übertragbar. Die Verordnung gilt nur für den Versicherten, für den sie ausgestellt ist. Änderungen und Ergänzungen bedürfen der Unterschrift des verordnenden Arztes.

(4) Der verordnende Vertragsarzt legt nach aktuellem Versorgungsbedarf die Dauer der Versorgung fest.

Der Krankenhausarzt kann Leistungen in der Regel für 7 Tage verordnen. Danach muss ein Vertragsarzt die Folgeverordnung ausstellen. In den Fällen, wo der Palliativpatient nach der Entlassung aus dem Krankenhaus mit einer Lebenserwartung von nur noch wenigen Tagen entlassen wird, ist eine längere Verordnungsdauer durch den Krankenhausarzt möglich.

(5) Das SAPV-Team prüft im Rahmen des Eingangsassessments innerhalb von drei Arbeitstagen nach Erhalt der Verordnung die Notwendigkeit und Eignung der besonderen Mittel der SAPV. Sofern für eine bedarfsgerechte Versorgung die Mittel der Regelversorgung ausreichen, ist die Krankenkasse unverzüglich zu informieren. Neben der Krankenkasse sind auch der Versicherte und der Erstverordner über die Ergebnisse des Assessments zu informieren

Muster

## § 7
## Prüfung der Leistungsansprüche durch die Krankenkassen

(1) Leistungen der SAPV werden von den Krankenkassen nur genehmigt, wenn eine ordnungsgemäß ausgefüllte ärztliche Verordnung vorliegt.

(2) Die Krankenkasse übernimmt bis zu einer Entscheidung über die weitere Leistungserbringung die Kosten für die verordneten und erbrachten Leistungen entsprechend der vereinbarten Vergütung nach § 16, wenn die Verordnung gemäß § 6 spätestens an dem dritten der Ausstellung folgenden Arbeitstag der Krankenkasse vorgelegt wird.

(3) Liegt der Krankenkasse innerhalb von 7 Arbeitstagen ab Verordnungsbeginn zusätzlich zur Erstverordnung kein Ergebnis über das SAPV-Eingangsassessment des leitenden Palliativmediziners vor, endet die vorläufige Kostenzusage mit Ablauf dieses Tages. Die Krankenkasse informiert den Versicherten oder eine ihm nahe stehende Person, den Leistungserbringer und den verordnenden Arzt.

(4) Die Krankenkassen behalten sich zur Prüfung der Leistungsansprüche die Möglichkeiten der Prüfung durch den Medizinischen Dienst der Krankenkassen vor.

## § 8
## Inhalt und Umfang der spezialisierten ambulanten Palliativversorgung

(1) Gemäß der SAPV-Richtlinie hat die SAPV insbesondere folgenden Inhalten zu genügen:

- Koordination der spezialisierten palliativmedizinischen und palliativpflegerischen Versorgung unter Einbeziehung weiterer Berufsgruppen und von Hospizdiensten im Rahmen einer multiprofessionellen Zusammenarbeit,
- Symptomlinderung durch Anwendung von Medikamenten oder anderen Maßnahmen,
- Apparative palliativmedizinische Behandlungsmaßnahmen (z.B. Medikamentenpumpe),
- Palliativmedizinische Maßnahmen, die nach ihrer Art, Schwere oder Komplexität eine Kompetenz erfordern, die der einer Ärztin oder eines Arztes mit Zusatzweiterbildung Palliativmedizin entspricht,
- Spezialisierte palliativpflegerische Leistungen, die nach ihrer Art, Schwere oder Komplexität eine Kompetenz erfordern, die der einer Pflegefachkraft mit einer curricularen Weiterbildung zu Palliativ Care entspricht,
- Führung eines individuellen Behandlungsplanes, vorbeugendes Krisenmanagement, Bedarfsinterventionen,
- Ruf-, Notfall- und Kriseninterventionsbereitschaft rund um die Uhr für die im Rahmen der SAPV betreuten Patienten zur Sicherstellung der im Rahmen der SAPV erforderlichen Maßnahmen,
- Beratung, Anleitung und Begleitung der Patienten und Ihrer Angehörigen zur palliativen Versorgung einschließlich Unterstützung beim Umgang mit Sterben und Tod,
- Spezialisierte Beratung der betreuenden Leistungserbringer der Primärversorgung,
- Psychosoziale Unterstützung im Umgang mit schweren Erkrankungen in enger Zusammenarbeit z.B. mit Seelsorge, Sozialarbeit und ambulanten Hospizdiensten,
- Organisation regelmäßiger Fallbesprechungen,
- Dokumentieren und Evaluieren der wesentlichen Maßnahmen im Rahmen der SAPV.

(2) Die SAPV umfasst je nach Bedarf alle Leistungen der ambulanten Krankenbehandlung, soweit diese erforderlich sind. Sie umfasst zusätzlich die im Einzelfall erforderliche Koordination der diagnostischen, therapeutischen und pflegerischen Teilleistungen sowie die Beratung, Anleitung und Begleitung der verordnenden oder behandelnden Ärzte sowie der sonstigen an der allgemeinen Versorgung beteiligten Leistungserbringer, der Patienten und ihrer Angehörigen.

Muster

## § 9
## Zuzahlungen[2]

(1) SAPV wird als Sachleistung zur Verfügung gestellt. Jegliche Zuzahlungen vom Versicherten im Rahmen der Vertragsleistungen dürfen weder gefordert noch angenommen werden.

(2) Zuzahlungen dürfen auch in dem Fall nicht verlangt werden, wenn die Verordnung bei der Krankenkasse verspätet eingereicht wird, da Kosten erst ab Eingang der Verordnung bei der Krankenkasse gemäß § 11 dieses Vertrages übernommen werden.

(3) Verstöße berechtigen zu Maßnahmen gemäß § 22 dieser Vereinbarung.

## § 10
## SAPV-Team

(1) Die Leistungen der SAPV werden ausschließlich durch das SAPV-Team im Sinne der Gemeinsamen Empfehlungen der Spitzenverbände der Krankenkassen nach § 132 d Abs. 2 SGB V erbracht. Hierzu zählen qualifizierte Ärzte, qualifizierte Pflegefachkräfte sowie ggf. weitere qualifizierte Fachkräfte.

(2) Der inhaltliche und organisatorische Rahmen der Leistungserbringung (inkl. der personellen und sächlichen Ausstattung) ist Bestandteil des Versorgungskonzeptes gemäß Anlage 1.

(3) Das SAPV Team hat folgende Mindestanforderungen an die sächliche Ausstattung vorzuhalten:
- geeignete, aktuell geführte und für die an der Versorgung Beteiligten jederzeit zugängliche Patientendokumentation
- Arzneimittel (inkl. BtM[3]) für die Notfall-/Krisenintervention
- Arzt-/Pflegekoffer/Bereitschaftstasche (Berücksichtigung der Kompatibilität der Verbrauchsmaterialien zu Medizinprodukten unterschiedlicher Hersteller, z.B. bei Portsystemen oder Infusionspumpen)
- geeignete administrative Infrastruktur, z. B. Büro, Kommunikationstechnik.

Ferner muss das SAPV-Team über eine eigenständige Adresse und geeignete Räumlichkeiten für
- die Beratung von Patienten und Angehörigen
- Teamsitzungen und Besprechungen
- die Lagerhaltung von eigenen Medikamenten für Notfall-/Krisenintervention und Hilfsmitteln verfügen.

(4) Das SAPV-Team stellt sicher, dass das erforderliche und geeignete Personal zur Verfügung steht. Dabei ist zu berücksichtigen, dass das SAPV-Team eine tägliche telefonische Rund-um-die-Uhr-Erreichbarkeit für die Patienten, deren Angehörigen und die an der Versorgung Beteiligten sicherstellt. Die ständige Verfügbarkeit mindestens eines Arztes und/oder einer Pflegefachkraft ist zu gewährleisten. Die Verfügbarkeit schließt notwendige Hausbesuche ein.

Die personellen Anforderungen an die spezialisierten SAPV-Teams ergeben sich aus Gemeinsamen Empfehlungen der Spitzenverbände der Krankenkassen nach § 132 d Abs. 2 SGB V. Übergangsregelungen sind in den Empfehlungen nicht vorgesehen.

[2] Zuzahlungen nach § 61 SGB V bleiben unberührt.
[3] Gemäß dem BtM Gesetz.

Muster

a) <u>Ärzte verfügen über</u>

- eine anerkannte Zusatzweiterbildung Palliativmedizin nach der aktuell gültigen Weiterbildung der Landesärztekammer Mecklenburg-Vorpommern
  und
- Erfahrungen aus der ambulanten palliativen Behandlung von mindestens 75 Palliativpatienten, z. B. in der häuslichen Umgebung oder in einem stationären Hospiz, innerhalb der letzten drei Jahre oder aus einer mindestens einjährigen klinischen palliativmedizinischen Tätigkeit in einer Palliativabteilung in einem Krankenhaus innerhalb der letzten drei Jahre.

b) <u>Pflegefachkräfte verfügen über</u>

- die Erlaubnis zur Führung einer der Berufsbezeichnungen Gesundheits- und Krankenpfleger, Gesundheits- und Kinderkrankenpfleger oder Altenpfleger (dreijährige Ausbildung aufgrund einer landesrechtlichen Regelung) entsprechend den gesetzlichen Bestimmungen in der jeweils gültigen Fassung und
- den Abschluss einer curriculären Palliative-Care-Weiterbildungsmaßnahme im Umfang von mindestens 160 Stunden und
- Erfahrungen durch mindestens eine zweijährige praktische Tätigkeit als Pflegefachkraft in der Betreuung von Palliativpatienten in den letzten drei Jahren; davon mindestens sechsmonatige Mitarbeit in einer spezialisierten Einrichtung der Hospiz und Palliativversorgung.

<u>c) weitere Fachkräfte (z.B. Sozialarbeiter, Sozialpädagogen oder Psychologen)</u>

- eine Zusatzweiterbildung Palliative Care für andere Berufsgruppen
  oder
- eine mehrjährige Erfahrung in der Palliativversorgung nachzuweisen.

(5) Die vorgenannten Leistungserbringer können sowohl Mitglied des SAPV Teams sein als auch in untervertraglichen Kooperationsvereinbarungen eingebunden werden.

(6) Nachweise über die vorgenannten Qualifikationen sind Bestandteil des Versorgungskonzepts gemäß Anlage 1 und werden von den Krankenkassen geprüft.

(7) Die Einzelheiten zu den Leistungen des SAPV- Teams ergeben sich aus Anlage 3.

## § 11
## Verordnungen von Arzneimitteln im Rahmen der SAPV

(1) Der qualifizierte Palliativarzt des SAPV-Teams verordnet die notwendigen Arznei-, Heil- und Hilfsmittel ausschließlich auf den im Rahmen der vertragsärztlichen Versorgung zu verwendenden Mustern 13, 14, 16 und 18 auf den Namen des Versicherten.

(2) Die Verordnungen sind auf Basis der geltenden Richtlinien des G-BA auszuführen. Dabei ist das Wirtschaftlichkeitsgebot nach § 12 SGB V zu beachten.

(3) Für die im Rahmen der SAPV verordneten Arznei-, Heil- und Hilfsmittel gelten die nach §§ 300, 302 SGB V vereinbarten Abrechnungsverfahren entsprechend.

(4) Auf den Verordnungen sind die von der Kassenärztlichen Vereinigung Mecklenburg Vorpommern vergebene SAPV spezifische Betriebsstätten-Nummer (BSNR) sowie die Arztnummer (LANR) anzugeben. Diese Nummern dürfen nur für Verordnungen im Rahmen der SAPV auf Basis einer genehmigten SAPV-Verordnung verwendet werden.

Muster

(5) Bei der (zeitgleichen) Verordnung von Arzneimitteln einerseits und Hilfsmitteln andererseits sind jeweils getrennte Vordrucke nach Muster 16 zu verwenden.

(6) Auch auf Betäubungsmittel-Rezepten sind die SAPV Betriebsstätten-Nummer sowie die Arztnummer anzugeben. Solange das BtM-Vordruck-Muster noch nicht angepasst ist, ist die Betriebsstätten-Nummer in das Feld "Vertragsarztnummer" und die Arztnummer in das Feld "VK gültig bis" einzutragen.

## § 12
## Netzwerk der regionalen palliativmedizinischen Versorgung (Zusammenarbeit der Leistungserbringer)

(1) Die Umsetzung der regionalen Palliativversorgung kann nur dann qualitativ hochwertig sein, wenn insbesondere die vorhandenen Strukturen der Palliativversorgung und der psychosozialen Unterstützung, z.B.

- qualifizierte Vertragsärzte
- amb. Pflegedienste
- Palliativstationen
- amb./stat. Hospize
- Apotheken
- Psychoonkologische Beratung
- Seelsorge
- Sozialarbeit

in abgestimmter Vorgehensweise mit dem Versorgungsportfolio der SAPV vorgehalten und genutzt werden.

(2) Davon ausgehend ist das SAPV-Team verpflichtet, mit den regelhaft an der allgemeinen und spezialisierten Palliativversorgung beteiligten Leistungserbringern (z.B. amb./stat. Hospiz, Palliativstation) im Einzugsgebiet des SAPV- Teams auf der Grundlage schriftlicher Kooperationsverträge zusammenzuarbeiten.

(3) Die vorgenannten Kooperationen sind Bestandteil des Versorgungskonzepts gemäß Anlage 1. Es erfolgt eine enge Kooperation des Leistungserbringers mit dem behandelnden Haus- oder Facharzt hinsichtlich der Therapieplanung. Insbesondere erfolgt vor einer stationären Krankenhauseinweisung eine Abstimmung zwischen dem qualifizierten Arzt des SAPV- Teams und dem behandelnden Haus- oder Facharzt.

## § 13
## Zusammenarbeit mit Krankenhäusern

(1) Die Sicherstellung einer kooperativen sektorenübergreifenden Zusammenarbeit zwischen dem SAPV-Team und den im Versorgungsgebiet ansässigen Krankenhäusern ist sicherzustellen.

(2) Zur Sicherstellung der Überleitung der Palliativpatienten von der stationären Krankenhausbehandlung in die Häuslichkeit ist zwischen dem SAPV-Team und den Krankenhäusern ein „Überleitungsprocedere" abzustimmen.

Muster

## § 14
## Dokumentation

(1) Zur Dokumentation der Palliativfälle[4] ist das SAPV-Team verpflichtet, ein geeignetes Dokumentationssystem sachgerecht und kontinuierlich zu führen. Das Dokumentationssystem hat insbesondere den Anforderungen gemäß Anlage 6 zu genügen.

(2) Das SAPV-Team hat eine Patientendokumentation zu führen, aus der zu jeder Zeit die notwendigen Informationen über den Versicherten und die erbrachten Leistungen hervorgehen. Die Patientendokumentation wird regelmäßig von dem Team aktualisiert.

(3) Das Dokumentationssystem hat allgemein anerkannte Kriterien für eine externe Qualitätssicherung zu enthalten und muss eine bundesweite Evaluation ermöglichen.

(4) Weiterhin hat das SAPV-Team zu gewährleisten, dass die von ihm angewandte Dokumentation kompatibel zu bestehenden Dokumentationssystemen der an der Versorgung beteiligten Leistungserbringer ist.

(5) Die Dokumentation beinhaltet insbesondere:

a. ein Versichertenstammblatt (z. B. mit Angaben zum Versicherten und Angehörigen, Hausarzt, Pflegedienst, Diagnosen, Absprachen, Behandlungsort, Medikation, Maßnahmen, usw.),

b. ein Assessment mit folgenden Inhalten:

- Aufklärung des Versicherten / der Angehörigen,
- Therapieverzicht,
- Palliative Sedierung,
- Ethische Konflikte,
- Funktionale Selbstständigkeit,
- Familiäre Belastungssituation,
- Einschätzung Symptomkontrolle,
- Medikation,
- Bedarfsmedikation,
- Hilfsmittel,
- Physikalische und andere Therapien (z. B. Lymphdrainage),
- Beratungsbedarf

c. einen Maßnahmenplan / Durchführungsnachweis

d. Nachweise über die durchgeführten Maßnahmen in den Bereichen Beratung, Koordination, Pflege, Medizin, psychosoziale Unterstützung (Datum, Uhrzeit, Dauer)

e. einen Notfallplan / vorausschauender Behandlungsplan

f. Untersuchungsbefunde

g. das vertragsärztliche Verordnungsblatt für die Dokumentation der medizinischen Diagnostik und Therapie

h. eine Pflege- und Therapieplanung (Therapiebeginn, medizinische / pflegerische / psychosoziale Versorgungssituation, Therapie- und Pflegeziele)

---

[4] Ein Palliativfall umfasst alle durch die Krankenkasse genehmigten Zeiträume der SAPV.
Ein Bezug auf den Behandlungsfall/Krankheitsfall, etc. gem. § 21 BMV-Ä erfolgt in diesem Kontext nicht.

Muster

i. einen Bericht zur Beurteilung der medizinischen, pflegerischen und psychosozialen Maßnahmen.

(6) Jede für den Versicherten bzw. für dessen Angehörige erbrachte Leistung wird durch die durchführende qualifizierte Person dokumentiert und durch Handzeichen gegengezeichnet. Näheres regelt Anlage 6 zur EDV-gestützten Dokumentation.

Der Versicherte, sein gesetzlicher Vertreter oder die Angehörigen bestätigen wöchentlich durch ihre Unterschrift die Richtigkeit und Vollständigkeit der dokumentierten Leistungen, soweit sie in der Häuslichkeit erbracht wurden (Leistungsnachweis gemäß Anlage 7).

(7) Geeignete Dokumente für die Koordination der SAPV sind beim Versicherten vorzuhalten.

(8) Die Dokumentation ist dem MDK und – soweit der Versicherte eingewilligt hat (§ 67b Abs. 1 SGB X) – der Krankenkasse auf Anforderung zugänglich zu machen.

(9) Die Aufbewahrungsfrist für die Dokumentation beträgt 10 Jahre nach Ende des Kalenderjahres der Leistungserbringung.

## § 15
## Datenschutz

(1) Versicherten- und Leistungsdaten dürfen nur im Rahmen der in § 284 SGB V genannten Zwecke erhoben, verarbeitet, bekannt gegeben, zugänglich gemacht oder genutzt werden. Das SAPV-Team bzw. die von ihm beauftragte Abrechnungsstelle verpflichten sich, den Schutz der Sozialdaten (SGB X, 2. Kapitel; § 11 Abs. 3 BDSG) sicherzustellen.

(2) Das SAPV- Team sowie die von ihm beauftragte Abrechnungsstelle unterliegen hinsichtlich der Person des Versicherten der Schweigepflicht. Ausgenommen hiervon sind Angaben gegenüber der zur Leistung verpflichteten Krankenkasse und dem Medizinischen Dienst der Krankenversicherung, soweit diese zur Erfüllung der gesetzlichen Aufgaben der Krankenkassen erforderlich sind.

(3) Das SAPV-Team hat seine Mitarbeiter zur Beachtung der Datenschutzbestimmungen und der Schweigepflicht aktenkundig zu verpflichten und deren Beachtung sicherzustellen.

## § 16
## Vergütung

(1) Die Vergütung erfolgt in Form von Pauschalen. Die Vergütung ist in Anlage 4 geregelt.

(2) Mit den Pauschalen sind alle Aufwendungen abgegolten, die im Zusammenhang mit der SAPV- Leistung stehen. Sie beinhalten ebenso den notwendigen Verwaltungsaufwand, u. a. Vorbereitungs-, Nachbereitungs- und Dokumentationsaufwand, Qualitätssicherung, die Übersendung der Dokumentation oder Teilen der Dokumentation für die Mitwirkungspflicht nach § 276 Abs. 1 SGB V, Aufwand für den Datenträgeraustausch sowie die Wegezeiten.

(3) Die Abrechnung erfolgt einheitlich durch das SAPV- Team über die KVMV.

(4) Neben der abgerechneten SAPV- Leistung ist zeitgleich keine andere Leistungsabrechnung möglich.

(5) Kosten, die der KVMV bei der Umsetzung dieses Vertrages entstehen, werden über die Verwaltungskostenpauschale im Rahmen des Honorarbescheides des Kontoinhabers (SAPV Team) abgegolten.

## § 17
## Qualitätssicherung

(1) Das SAPV- Team ist verpflichtet, ein internes Qualitätsmanagement durchzuführen.

(2) Das SAPV- Team stellt sicher, dass dessen jeweilige Teammitglieder regelmäßig an palliativmedizinischen/- pflegerischen Fortbildungen teilnehmen. Die jeweiligen Nachweise dafür legt das SAPV-Team den Krankenkassen jährlich vor.

Halbjährlich ist durch das SAPV-Team ein multidisziplinärer Qualitätszirkel zum Thema „Palliativ-Medizin" durchzuführen, an dem auch die übrigen an der Palliativversorgung teilnehmenden Leistungserbringer teilnehmen.

(3) Das Palliativteam soll sich an Maßnahmen der externen Qualitätssicherung beteiligen.

(4) Das SAPV-Team hat seinen Teammitgliedern die Teilnahme an Supervision zu ermöglichen.

(5) Regelmäßige Fallbesprechungen sind innerhalb des SAPV-Teams in überschaubaren Intervallen durchzuführen.

(6) Die Krankenkasse hat das Recht, jeder Zeit selbst und/oder durch Beauftragung des MDK nach § 275 SGB V vor Ort die Erfüllung der Mindestanforderungen an die sächliche Ausstattung, die Dokumentation, die Qualifikationen und die Leistungserbringung nach diesem Vertrag zu überprüfen.

## § 18
## Abrechnung/ Beanstandung/ Verjährung

(1) Die Abrechnung der in Anlage 4 geregelten Vergütung erfolgt entsprechend der Regelung in § 16 Abs. 3.
Durch die KVMV als Abrechnungsstelle des SAPV- Teams ist das entsprechende Institutionskennzeichen, unter dem die Rechnungslegung vorgenommen wird, den Krankenkassen bzw. den von ihnen benannten Abrechnungsstellen mitzuteilen.

(2) Sobald die Bestimmungen zur Umsetzung der Abrechnung über DTA nach § 302 SGB V vorliegen, sind diese anzuwenden. Erforderliche Abstimmungen zur Anpassung des Vertrages und seiner Anlagen sind zwischen den Vertragspartnern vorzunehmen.

(3) Die abrechnungsspezifischen Besonderheiten für die Krankenkassen sind in Anlage 5 geregelt.

Muster

Muster

## § 19
## Institutionskennzeichen

(1) Das SAPV-Team verfügt gemäß § 293 SGB V über ein Institutionskennzeichen (IK), das es bei der Abrechnung mit den Krankenkassen verwendet. Für jede Filiale, Zweigstelle etc. ist ein gesondertes IK zu führen.

(2) Änderungen des eingesetzten IK sind den Vertragspartnern mitzuteilen.

(3) Das IK ist in jeder Abrechnung anzugeben. Abrechnungen ohne IK oder mit fehlerhaftem IK werden von den Krankenkassen abgewiesen. Gleiches gilt für Abrechnungen mit einem der Krankenkasse unbekannten IK.

## § 20
## Vertragsbeirat

(1) Die Partner des Vertrages bilden einen Vertragsbeirat. Der Vertragsbeirat begleitet die Vertragsumsetzung und wird bei Bedarf Anpassungen an die regionalen Versorgungsbedürfnisse vorschlagen und die dafür notwendigen Schritte zur Vertragsanpassung einleiten.

(2) Auf Verlangen eines Vertragspartners ist der Vertragsbeirat schriftlich einzuberufen.

(3) Zu den Aufgaben des Vertragsbeirates gehören insbesondere:
- Unterstützung zur Strukturierung erforderlicher Versorgungsabläufe,
- Schlichtung in Bezug auf Streitfragen der Vertragsumsetzung,
- Unterstützung zur Lösung von Problemfeldern,
- Begleitung des Vertrages sowie Empfehlungen zu Vertragserweiterungen,
- Festlegung von Korrekturmechanismen bei erkennbaren Fehlsteuerungen innerhalb der regionalen Palliativversorgung sowie
- Empfehlungen zum Abschluss von Kooperationsverträgen.

(4) Die Kosten werden durch die jeweiligen Vertragspartner selbst getragen.

## § 21
## Statistik

(1) Das SAPV-Team ist verpflichtet, an Auswertungen mitzuwirken und diese auf Anforderung den Vertragspartnern zu übergeben.

(2) Die Auswertungen beinhalten die Struktur- und anonymisierten Leistungskennzahlen (siehe Anlage 6).
Hierzu zählen u.a.:

- Fallzahlen (Anzahl der behandelten Patienten),
- Diagnosen,
- Zahl der Vertragsärzte,
- Zahl der Kooperationspartner,
- Anzahl der beteiligten Ehrenamtlichen - soweit bekannt

Muster

- Anzahl der durchgeführten SAPV- Leistungen gegliedert nach Anlage 3

(3) Die Daten nach Absatz 2 sind in maschinenlesbarer Form einzureichen.

## § 22
## Vertragsverletzung

(1) Besteht der Verdacht eines Verstoßes gegen die Strukturvoraussetzung, die Wirtschaftlichkeit und/oder das Versorgungskonzept aus diesem Vertrag, ist das SAPV-Team anzuhören. Das SAPV-Team hat dieser Anhörung innerhalb einer Frist von 14 Tagen nach Zugang des Anhörungsschreibens Folge zu leisten. Das SAPV-Team kann dabei eine Interessensvertretung hinzuziehen.

(2) Unabhängig von der Anhörung nach Absatz 1 ist der durch die Vertragsverletzung verursachte Schaden zu ersetzen. Bei Fortbestehen der Ursachen für die Vertragsverletzung sind die Krankenkassen berechtigt, die Zahlung einzustellen, bis Abhilfe geschaffen wird.

## § 23
## Inkrafttreten/ Kündigung

(1) Der Vertrag einschließlich seiner Anlagen tritt zum XX.XX.XXXX in Kraft. Er kann unter Einhaltung einer Frist von 6 Monaten zum Quartalsende, erstmals zum XX.XX.XXXX, gekündigt werden.

(2) Die Kündigung des Vertrages bedarf der Schriftform. Die Kündigung des Vertrages durch eine Partei berührt die Wirksamkeit des Vertrages gegenüber den anderen Parteien nicht.

(3) Die Kündigung der Anlagen kann nach den darin getroffenen Regelungen unabhängig von diesem Vertrag erfolgen. Soweit nichts anderes geregelt ist, können die Anlagen mit einer Frist von 3 Monaten zum Quartalsende gekündigt werden. Sie bedürfen der Schriftform.

(4) Das Recht zur außerordentlichen Kündigung dieses Vertrages aus wichtigem Grund bleibt davon unberührt. Zu diesen Gründen gehören insbesondere:

a) fortgesetzte Nichterfüllung wesentlicher fachlicher Voraussetzungen,

b) wiederholte Abrechnung von nicht oder teilweise nicht erbrachten Leistungen,

c) einer Pflichtverletzung durch das SAPV-Team, bei der ein Versicherter zu Schaden kommt,

d) Forderung bzw. Annahme von Zahlungen oder sonstiger geldwerter Vorteile von den Versicherten für Leistungen nach diesem Vertrag,

e) Zahlung oder Annahme von Vergütungen oder Provisionen für Zuweisungen von Versicherten oder die Vermittlung von Aufträgen außerhalb der Kooperationsvereinbarungen.

## § 24
## Änderungen und Ergänzungen

Änderungen und Ergänzungen dieses Vertrages sind möglich, ohne dass es einer Kündigung bedarf. Sie bedürfen zu ihrer Wirksamkeit der Schriftform und der Abstimmung zwischen dem SAPV-Team, der KVMV und den Krankenkassen.

## § 25
## Salvatorische Klausel

(1) Sollten einzelne Bestimmungen dieses Vertrages, einschließlich der zugehörigen Regelungen ganz oder teilweise unwirksam sein oder werden, oder sollte der Vertrag eine Regelungslücke enthalten, bleibt die Wirksamkeit der übrigen Bestimmungen oder Teilen dieser Bestimmungen davon unberührt.

(2) Anstelle der unwirksamen oder fehlenden Bestimmungen treten die jeweiligen gesetzlichen Regelungen bzw. Bestimmungen, die dem Sinn und Zweck der unwirksamen oder fehlenden Bestimmungen in rechtlicher und wirtschaftlicher Weise entsprechen.

(3) Die Vertragspartner verständigen sich dazu unverzüglich über notwendige neue Regelungen.

(4) Sie bedürfen zu ihrer Wirksamkeit der Schriftform.

Muster

**Versorgungsbereich, Schwerin, Hamburg, Hoppegarten, den XX.XX.XXXX**

„...GbR"

AOK Nordost
Die Gesundheitskasse.

Kassenärztliche Vereinigung
Mecklenburg-Vorpommern

BKK-Vertragsarbeitsgemeinschaft NORD
Vorsitzender Vertragsausschuss

IKK Nord, in Vertretung des IKK Landesverbandes Nord

LKK Mittel- und Ostdeutschland

Knappschaft Regionaldirektion Hamburg
handelnd als Landesverband

Verband der Ersatzkassen e.V. (vdek)
Der Leiter Landesvertretung

Muster

**Anlage 1 Versorgungskonzept**

Vertrag über die spezialisierte ambulante Palliativversorgung gemäß § 37 b i.V.m. § 132 d SGB V zwischen der „...GbR" und Kassenärztliche Vereinigung Mecklenburg-Vorpommern und AOK Nordost - Die Gesundheitskasse, BKK-Vertragsarbeitsgemeinschaft NORD-, IKK Nord in Vertretung des IKK Landesverbandes Nord, LKK Mittel- und Ostdeutschland, Knappschaft, BARMER GEK, Techniker Krankenkasse, Deutsche Angestellten-Krankenkasse, KKH-Allianz, HEK - Hanseatische Krankenkasse, hkk, gemeinsamer Bevollmächtigter mit Abschlussbefugnis, Verband der Ersatzkassen e.V. (vdek), Berlin, vertreten durch den Leiter der Landesvertretung Mecklenburg-Vorpommern

# Konzept zur

# Spezialisierten ambulanten palliativmedizinischen Versorgung

## der Versorgungsregion

erstellt von „...GbR"

Muster

Muster

## Anlage 2 Verordnungsvordruck (Muster 63 - Verordnung spezialisierter ambulanter Palliativversorgung)

Vertrag über die spezialisierte ambulante Palliativversorgung gemäß § 37 b i.V.m. § 132 d SGB V zwischen der „...GbR" und Kassenärztliche Vereinigung Mecklenburg-Vorpommern und AOK Nordost - Die Gesundheitskasse, BKK-Vertragsarbeitsgemeinschaft NORD-, IKK Nord in Vertretung des IKK Landesverbandes Nord, LKK Mittel- und Ostdeutschland, Knappschaft, BARMER GEK, Techniker Krankenkasse, Deutsche Angestellten-Krankenkasse, KKH-Allianz, HEK - Hanseatische Krankenkasse, hkk, gemeinsamer Bevollmächtigter mit Abschlussbefugnis, Verband der Ersatzkassen e.V. (vdek), Berlin, vertreten durch den Leiter der Landesvertretung Mecklenburg-Vorpommern

"Muster 63.pdf"

## Anlage 3 Leistungen des SAPV-Teams

Vertrag über die spezialisierte ambulante Palliativversorgung gemäß § 37 b i.V.m. § 132 d SGB V zwischen der GbR und der Kassenärztliche Vereinigung Mecklenburg-Vorpommern und AOK Nordost - Die Gesundheitskasse, BKK- Vertragsarbeitsgemeinschaft NORD, IKK Nord in Vertretung des IKK Landesverbandes Nord, LKK Mittel- und Ostdeutschland, Knappschaft, BARMER GEK, Techniker Krankenkasse, Deutsche Angestellten-Krankenkasse, KKH, hkk, gemeinsamer Bevollmächtigter mit Abschlussbefugnis, Verband der Ersatzkassen e.V. (vdek).

Die nachfolgend aufgeführten Komplexpauschalen beinhalten alle Anwendungen (u.a. Personal-, Sachkosten-, Fort- und Weiterbildungskosten, Supervision, Dokumentation, Qualitätssicherung) die im Zusammenhang mit der SAPV Leistung stehen.

# 1. Beratung und Koordination

## 1.1 Eingangsassessment

Erfolgt in der häuslichen Umgebung, damit eine Bewertung der häuslichen und familiären Strukturen erfolgen kann. Die Sicherstellung der ambulanten Versorgungssituation ist zu bewerten. (falls das Erstgespräch im Krankenhaus stattfindet, muss die Besichtigung des häuslichen Umfeldes nachgeholt werden). Über die einzusetzende Profession für das Eingangsassessment entscheidet das SAPV-Team im jeweiligen Einzelfall.

- Aufnahme der SAPV-Anamnese (körperlicher Zustand Grunderkrankungen, Nebendiagnosen, symptomatische Belastung in Fremd- und Selbstbeurteilung, Prioritätenliste des Patienten, bisherige krankheitsmodifizierende Therapie, Medikamentenanamnese etc.)
- ärztliche Entscheidung, ob SAPV-Fall (ja oder nein)
- palliativpflegerische Anamnese
- psychosoziale Anamnese
- Festlegung des Therapiekonzeptes im SAPV-Fall
- Formulierung des spezifisch palliativmedizinischen Behandlungsauftrages und Behandlungszieles
- Erstellung Therapiekonzeptes unter Berücksichtigung der medizinischen, pflegerischen und psychosozialen Bedürfnisse des Patienten / der Angehörigen
- Dokumentation des Therapiekonzeptes
- Abstimmung und ggf. Modifikation des Therapiekonzeptes mit bereits involvierten Leistungserbringern / Kooperationspartnern
- Ermittlung, Information und Einbeziehung potentieller neuer Kooperationspartner zur Umsetzung des Therapiekonzeptes
- Psychosoziale Betreuung
- Beratung im Kontext zu Unheilbarkeit und Tod
- inkl. Aufwand für die Fahrt in die Häuslichkeit des Patienten oder in einem Krankenhaus

Handelt es sich nicht um SAPV, sondern reichen die Mittel der Regelversorgung und allgemeinen Palliativversorgung aus, koordiniert das SAPV-Team den Rücktransfer des Patienten in die Regelversorgung (z.B. Kontaktaufnahme mit den behandelnden Haus- und Fachärzten). Auch in diesen Fällen wird der volle Pauschalbetrag vergütet.

## 1.2 Tagespauschale SAPV-Team

Beinhaltet alle beratenden, koordinierenden und organisatorischen Leistungen des SAPV-Teams.

### 1.2.1 Pflegekraft

- Telefonische Abstimmung oder Beratung des Patienten und/oder Angehörigen im Zusammenhang mit der palliativen Betreuung

- Telefonische Abstimmung zur Organisation der häuslichen Betreuung des Palliativpatienten
  a) Zusammenarbeit mit dem betreuendem Hausarzt/ Facharzt
    - Mitteilung Änderung Krankheitszustand
    - Anmeldung bei Bedarf für Hausbesuche

  b) Zusammenarbeit mit Pflegedienst
    - Spezialisierte Beratung der betreuenden Leistungserbringer der Primärversorgung
    - Sicherstellung gegenseitige Information zum amb. Pflegedienst

  c) Zusammenarbeit mit Hilfsmittelleistungserbringer
    - Kontaktaufnahme zum Hilfsmittellieferanten nach Bedarf zur Sicherung der Funktionsfähigkeit des Hilfsmittels
    - Mitteilung Wegfall der Notwendigkeit des Hilfsmittels

  d) Zusammenarbeit mit ambulanten Hospizdienst
    - Bei Kontaktaufnahme
    - Abstimmung zu Sitzdiensten bzw. Sitzwachen

- Anleitung von Pflegediensten
  - Beratung und Anleitung von Pflegediensten bei Bedarf zu Besonderheiten der Behandlungspflege bei Palliativpatienten

- Rufbereitschaft
  - Bereitschaftsdienst für 24-Stunden

- Dokumentation

### 1.2.2 Arzt

- Zusammenarbeit mit dem behandelndem Hausarzt/ Facharzt
  - Abstimmung zum Tätigwerden des SAPV-Teams
  - Abstimmung/ Zusammenarbeit für die gemeinsame palliative Betreuung
  - Interkollegiale Beratung
  - Empfehlung palliative Therapie
  - Empfehlung erforderliche und vorausschauende Medikation
  - Empfehlung erforderliche Hilfsmittel und HKP

- Rufbereitschaft
  - Bereitschaftsdienst für 24-Stunden

- Dokumentation

### 1.2.3 SAPV-Team (organisatorische Leistungen/ sächliche Kosten)

- Abstimmung innerhalb des SAPV-Teams
  - (telefonische Abstimmung) zwischen Arzt und Pflegekraft (z.B. bei Anleitung der Medikation)
  - Teambesprechungen/ Fallbesprechungen
  - Übergabe aus Bereitschafts- in Tagesdienst und umgekehrt

- Dokumentation
  - Dokumentation nach Kerndatensatz zwecks Evaluation

- Fortbildung
  - Regelmäßige Teilnahme an palliativmedizinischen/ -pflegerischen Fortbildungen

- Qualitätssicherung
  - Durchführung halbjährlicher multidisziplinärer Qualitätszirkel
  - Teilnahme an Maßnahmen der externen Qualitätssicherung

- Zusammenarbeit mit dem Kostenträger
  - Versenden von Unterlagen (Mitteilung über erforderliche Änderungen der patientenbezogenen SAPV (erweiterter/verminderter Interventionsbedarf; Abrechnungsunterlagen)
  - Abstimmung Hilfsmittelversorgung
  - Mitteilung des Todestages des Versicherten

- Administration
  - Arbeitsplatzorganisation
  - Erstellung der Dienstpläne

- Organisation
  - Vorhaltung der sächlichen und räumlichen Voraussetzungen gemäß den Empfehlungen nach § 132 d Abs. 2 SGB V für die spezialisierte ambulante Palliativversorgung in der jeweils gültigen Fassung.

### 1.2.4 Telefonische Beratung

- Beratung Angehöriger
- Beratung i. S. eines Konsilardienstes mit anderen Behandlern
- Beratung anderer, an der Versorgung Beteiligter
- Beratung außerhalb SAPV

## 2. Additiv unterstützende Teilversorgung

### 2.1 Pflegerischer Hausbesuch

- Einzelne Leistungen des in § 5 Abs. 3 der SAPV-Richtlinie des G-BA aufgeführten Leistungskatalogs werden bei Bedarf erbracht
- Überprüfung des Therapiekonzeptes und ggf. Modifikation in Abstimmung mit Arzt im SAPV-Team
- Beratung, Anleitung und Begleitung der Patienten und ihrer Angehörigen zur palliativen Versorgung einschließlich Unterstützung beim Umgang mit Sterben und Tod
- Spezialisierte palliativpflegerische Leistungen die nach ihrer Art, Schwere oder Komplexität Kompetenz erfordern die der einer Pflegefachkraft mit einer curricularen Weiterbildung zu Palliativcare entspricht
- Krisenmanagement und Bedarfsinterventionen
- Spezialisierte Beratung der betreuenden Leistungserbringer der Primärversorgung
- Unterstützung der Selbstversorgungskompetenz
- Psychosoziale Betreuung
- Mentale Unterstützung
- Zuwendung und Kommunikation
- Sicherung der Umsetzung im Gebrauch eines Hilfsmittels
- Einbeziehung anderer Akteure Anleitung am Krankenbett
- Dokumentation
- inkl. Aufwand für die Fahrt in die Häuslichkeit des Patienten (bis 30 km Einsatzradius)

### 2.2 Ärztlicher Hausbesuch

- Krisenintervention in Häuslichkeit des Patienten
- palliativmedizinische Maßnahmen, die nach ihrer Art, Schwere oder Komplexität eine Kompetenz erfordern, die der einer Ärztin oder eines Arztes mit Zusatzweiterbildung „Palliativmedizin“ entspricht, hierunter fallen u.a.:

    - Minimalinvasive Eingriffe wie Abtragung von Nekrosen
    - Anlage bzw. Wechsel eines Venenkatheters
    - Portpunktion (unter Verwendung eines tragbaren Ultraschallgerätes)
    - Ascitespunktion nur bei bekannter Genese und schon durchgeführter mehrfacher Punktion (unter Verwendung eines tragbaren Ultraschallgerätes)
    - Pleurapunktion (unter Verwendung eines tragbaren Ultraschallgerätes)
    - Bluttransfusionen
    - Infusionen
    - Intensivierung einer Schmerztherapie
    - Neuanlage / Neuprogrammierung einer Pumpe zur systemischen Schmerztherapie
    - Sondenanlage zur Ernährung

- Aktualisierung des Therapiekonzeptes
- Dokumentation
- inkl. Aufwand für die Fahrt in die Häuslichkeit des Patienten (bis 30 km Einsatzradius)

## 2.3 Aktiveinsätze im Rahmen der Rufbereitschaft

- „Unzeitenzuschlag" zwischen 20:00 Uhr und 6:00 Uhr ausschließlich bei Krisenintervention

# 3. Vollversorgung

## Vollversorgung durch das SAPV-Team

**Grundsatz**

- Neben der Vollversorgung durch das SAPV-Team erfolgt keine medizinische Behandlungspflege durch andere ambulante Pflegedienste.
- Eine Vollversorgung findet ausschließlich in der häuslichen Umgebung des Patienten statt.
- Die Vollversorgung erfolgt durch die pflegerischen und ärztlichen Teammitglieder.
- Ausgenommen aus der Pauschale sind: Heil- und Hilfsmittel, Medikamente zur Behandlung der Grunderkrankung, sonstige fachärztliche Konsile außerhalb des SAPV-Teams (z.B. Onkologe, Urologe, Zahnarzt, Gynäkologe, Neurologe)
- Neben der Vollversorgung sind die Leistungen nach Ziffer 2 nicht abrechenbar.
- Der Aufwand für die Fahrt in die Häuslichkeit des Patienten oder in ein Krankenhaus (bis 30 km Einsatzradius) ist inbegriffen.

**Aufgaben**

- Aktive ganzheitliche Behandlung zur Symptomkontrolle und psychosozialen Stabilisierung ohne kurative Intention und im allgemeinen ohne Beeinflussung der Grunderkrankung von Patienten mit einer progredienten, fortgeschrittenen Erkrankung und begrenzten Lebenserwartung unter Einbeziehung ihrer Angehörigen und unter Leitung eines SAPV-Arztes
- Aktivierende- oder begleitend-therapeutische Pflege durch die SAPV-Pflegefachkräfte (Sicherung der Umsetzung ärztlicher Verordnungen)
- Erstellung und Dokumentation eines individuellen Pflege- und Behandlungsplanes
- Im Krisenfall sofortige Abstimmung mit den Ärzten des Teams zur weiteren Versorgung (Anmeldung Hausbesuch)
- Mind. wöchentlich multidisziplinäre Teambesprechung und tagesbezogene Dokumentation bisheriger Ergebnisse und weitere Behandlungsziele und Maßnahmen in Pflege und Medizin
- Anleitung der Angehörigen bei der Pflege
- Sicherung der Umsetzung der Einweisung des Patienten und/oder Angehörigen im Gebrauch eines Hilfsmittels
- Psychosoziale Unterstützung des Patienten und seiner Angehörigen
  Patienten-, Angehörigen- und/oder Familiengespräche können von allen Berufsgruppen des Teams durchgeführt werden
- alle Leistungen des in § 5 Abs. 3 der Richtlinie des G-BA aufgeführten Leistungskatalog
- Grundpflege nach SGB XI ist nicht Bestandteil der SAPV, kann aber bei entsprechenden Voraussetzungen vom SAPV-Team erbracht und über SGB XI abgerechnet werden
- 24 Std. Rufbereitschaft

# 4. Sonstiges

- Zuschlag für Wegeaufwendungen bei An-/ Abfahrt ab 31. Entfernungskilometer (Nachweis per Fahrtenbuch)

**Hinsichtlich der Vergütung der Wegaufwendungen nach Anlage 4 Position 4 gelten ab 01.01.2012 folgende Grundsätze:**

1. Als Abfahrtsort zum Ort der Leistungserbringung (Häuslichkeit des Patienten) wird grundsätzlich vom Sitz des SAPV-Teams ausgegangen.

2. Das SAPV- Team ist verpflichtet, den Einsatz so zu gestalten, dass unter Beachtung der notwendigen Versorgung die Einsätze gem. dem Wirtschaftlichkeitsprinzip, § 4 Absatz 4 i. V. m. § 22 Absatz 1 des Vertrages, geplant und durchgeführt werden.

3. Abweichungen davon sind nur in Ausnahmefällen möglich und mit der Quartalsabrechnung entsprechend zu begründen.

   Der Abfahrtsort kann im Ausnahmefall und sofern dies wirtschaftlich ist (gemäß Satz 2 ), der Leistungsort, d.h. nicht der Sitz des Vertragspartners (SAPV Team) sondern der Standort der Vertragsarztpraxis bzw. des kooperierenden Pflegedienstes sein. Dies ist mit der Quartalsabrechnung entsprechend zu begründen.

## Anlage 4 Vergütung

Vertrag über die spezialisierte ambulante Palliativversorgung gemäß § 37b i.V.m. § 132d SGB V zwischen dem ... und der Kassenärztlichen Vereinigung Mecklenburg-Vorpommern und AOK Nordost - Die Gesundheitskasse, BKK-Landesverband NORDWEST, IKK Nord, LKK Mittel- und Ostdeutschland, Knappschaft, BARMER GEK, Techniker Krankenkasse, DAK-Gesundheit, Kaufmännische Krankenkasse (KKH), HEK, hkk, gemeinsamer Bevollmächtigter mit Abschlussbefugnis, Verband der Ersatzkassen e.V. (vdek).

### § 1
### Grundsätze der Vergütung und ihrer Strukturen

(1) Die Anlage 4 – Vergütung – ist Bestandteil des Vertrages.

(2) Die ärztlichen und pflegerischen bzw. kombinierten Leistungen im Rahmen der SAPV werden in Form von Komplexpauschalen vergütet.

(3) Mit den Komplexpauschalen sind alle Aufwendungen abgegolten, die im Zusammenhang mit der SAPV-Leistung stehen.

(4) Die Höhe der Vergütung richtet sich nach den in dieser Anlage aufgeführten Pauschalen.

(5) Die Abrechnung der Komplexpauschalen erfolgt entsprechend § 16 Abs. 3 des Vertrages mit der leistungspflichtigen Krankenkasse.

(6) Abrechnungsfähig sind nur tatsächlich erbrachte Vertragsleistungen.

(7) Der Zuschlag Wegeaufwendungen kann nur in Verbindung mit erbrachten Vertragsleistungen anfallen.

(8) Die Vertragspartner sind sich einig, dass Bestandteil dieser Anlage die Positionsnummern gem. dem bundeseinheitlichen Positionsnummernverzeichnis nach § 302 Absatz 2 SGB V sind, soweit sie vom Spitzenverband Bund der Krankenkassen bekannt gegeben werden.

### § 2
### Inkrafttreten/Kündigung

(1) Diese Anlage tritt mit Wirkung zum 01.12.2012 in Kraft und ersetzt die Anlage 4 in der Fassung vom ... . Sie kann mit einer Frist von 3 Monaten zum Quartalsende, erstmals zum 31.03.2016, gekündigt werden.

(2) Die Kündigung bedarf der Schriftform.

(3) Die Kündigung gegenüber einem Vertragspartner setzt deren Gültigkeit gegenüber den anderen Vertragspartnern nicht außer Kraft.

(4) Bis zum Abschluss einer neuen Vergütung gilt die bisherige Vergütung weiter.

Potsdam, Schwerin, Hamburg, Hoppegarten..., den

______________________________

SAPV-Team ...

______________________________

AOK Nordost – Die Gesundheitskasse

______________________________

Kassenärztliche Vereinigung Mecklenburg-Vorpommern

______________________________

BKK-Landesverband NORDWEST

______________________________

IKK Nord

______________________________

LKK Mittel- und Ostdeutschland

______________________________

Knappschaft Regionaldirektion Hamburg

______________________________

Verband der Ersatzkassen e.V. (vdek)
Der Leiter der vdek-Landesvertretung

**Anlage 4 Vergütung (Vergütungsvereinbarung)**
**Gültig vom 01.12.2012 bis 31.03.2016**

| GOP | | | Betrag | Anmerkung |
|---|---|---|---|---|
| **Pos-Nr** | **1.** | **Beratung/Koordination** | | |
| **SAPV11** | 1.1 | Eingangsassessment | 100,00 EUR | Einmalig |
| **SAPV12** | 1.2 | Tagespauschale SAPV-Team*[1] | 70,00 EUR | Pro Tag |

Grundsatz:
Grundsätzlich soll aus Wirtschaftlichkeitsgründen und wenn medizinisch vertretbar sowie bei Vorhandensein eines Hausarztes die Teilversorgung eingesetzt werden.

| | | | | |
|---|---|---|---|---|
| | **2.** | **Additive unterstützende Teilversorgung*[2]** | | |
| **SAPV21** | 2.1 | pflegerischer Hausbesuch | 40,00 EUR | |
| **SAPV22** | 2.2 | ärztlicher Hausbesuch | 60,00 EUR | |
| **SAPV23** | 2.3 | Aktiveinsätze im Rahmen der Rufbereitschaft (Zuschlag zwischen 20:00 Uhr und 6:00 Uhr) | 20,00 EUR | zzgl. zum Hausbesuch |
| **SAPV24** | 2.4 | ärztlicher Hausbesuch in einem stationären Hospiz | 80,00 EUR | |
| | **3.** | **Vollständige Versorgung*[3]** | | |
| **SAPV30** | 3. | Vollständige Versorgung durch das SAPV-Team | 185,00 EUR | |
| | **4.** | **Sonstiges** | | |
| **SAPV40** | 4.1 | Zuschlag Wegeaufwendungen ab 31. km | 0,30 EUR | ab 31. Entfernungskilometer |
| **SAPV42** | 4.2 | Zuschlag pflegerischer Hausbesuch ab 31.-50. km | 10,00 EUR | 31. – 50. Entfernungskilometer |
| **SAPV43** | 4.3 | Zuschlag ärztlicher Hausbesuch ab 31.-50. km | 15,00 EUR | 31. – 50. Entfernungskilometer |
| **SAPV44** | 4.4 | Zuschlag pflegerischer Hausbesuch ab 51. km | 20,00 EUR | ab 51. Entfernungskilometer |
| **SAPV45** | 4.5 | Zuschlag ärztlicher Hausbesuch ab 51. km | 30,00 EUR | ab 51. Entfernungskilometer |

*[1] Die Tagespauschale wird in der Häuslichkeit zusätzlich zur Teilversorgung gezahlt. In stationären Hospizen wird die Tagespauschale nicht neben der ärztlichen Teilleistung vergütet.
*[2] Additive Teilversorgung findet immer dort statt, wo der Hausarzt und/oder der Pflegedienst in die Behandlung integriert ist. In stationären Hospizen ist die SAPV22 nicht neben der SAPV24 abrechenbar.
*[3] Vollständige Versorgung findet immer dort statt, wo der Hausarzt die Behandlung an das SAPV-Team abgegeben hat und das SAPV-Team die Notwendigkeit festgestellt hat. Die Notwendigkeit ist begründet, wenn das SAPV-Team im Rahmen der additiv unterstützenden Teilversorgung innerhalb von 3 Tagen mehr als 8 Einsätze (davon mindestens 3 ärztliche Hausbesuche) durchgeführt hat. Neben der SAPV-Versorgung ist keine Behandlungspflege durch einen anderen Pflegedienst anrechenbar, Ansprüche nach SGB XI bleiben hiervon unberührt.

Muster

## Anlage 5 Abrechnungsspezifische Besonderheiten der Krankenkassen

Vertrag über die spezialisierte ambulante Palliativversorgung gemäß § 37 b i.V.m. § 132 d SGB V zwischen der „...GbR“ und Kassenärztliche Vereinigung Mecklenburg-Vorpommern und AOK Nordost - Die Gesundheitskasse, BKK-Vertragsarbeitsgemeinschaft NORD, IKK Nord in Vertretung des IKK Landesverbandes Nord, LKK Mittel- und Ostdeutschland, Knappschaft, BARMER GEK, Techniker Krankenkasse, Deutsche Angestellten-Krankenkasse, KKH-Allianz, HEK - Hanseatische Krankenkasse, hkk, gemeinsamer Bevollmächtigter mit Abschlussbefugnis, Verband der Ersatzkassen e.V. (vdek), Berlin, vertreten durch den Leiter der Landesvertretung Mecklenburg-Vorpommern

### Allgemeine Grundsätze zur Abrechnung der Leistungen

1. Die Vergütung der Leistung richtet sich nach der Vergütungsvereinbarung (Anlage 4).

2. Für den Abrechnungsverkehr ist das SAPV-Team und KVMV maßgebende Institutionskennzeichen (IK) zu verwenden, das von der Arbeitsgemeinschaft Institutionskennzeichen (SVI) beim Hauptverband der gewerblichen Berufsgenossenschaften e. V. vergeben wird.

3. Die Abrechnung ist versichertenbezogen je Quartal unter Beifügung der Verordnungen zu erstellen und bei den von den Krankenkassen benannten Daten- und Papierannahmestellen einzureichen. Die Krankenkassen sind zur Rechnungsbegleichung nur verpflichtet, sofern eine versicherungs-, leistungs- und vertragsrechtliche Anspruchsberechtigung gegeben ist.

4. Die Rechnung muss folgende Mindestinhalte aufweisen (vgl. Anhang – Musterabrechnung):
   - Name, Anschrift und Institutionskennzeichen des SAPV-Teams
   - Name und Vorname, Krankenversicherungsnummer, Geburtsdatum des Versicherten,
   - Gebührenpositionsnummer, Art und Anzahl der erbrachten Leistungen
   - Gesamtbetrag
   - Rechnungsnummer
   - Rechnungsdatum
   - Leistungserbringerschlüssel (sobald dieser bekannt ist)

   Die Rechnungen, einschließlich der Urbelege (Kostenzusage der Krankenkasse und unterschriebener Leistungsnachweis je Versicherter) sind an folgende Anschriften der Krankenkassen zu senden:

   - AOK = AOK Nordost, Am Grünen Tal 50, 19063 Schwerin
   - BKK BKK-Vertragsarbeitsgemeinschaft NORD = Anlistung der teilnehmenden Krankenkassen
   - IKK Nord = Moislinger Allee 19a, 23558 Lübeck
   - LKK Mittel- und Ostdeutschland= Hoppegartener Str. 100, 15366 Hoppegarten
   - Knappschaft = Millerntorplatz 1, 20359 Hamburg
   - vdek = Anlistung der einzelnen Mitgliedskassen[7]

5. Auf dem Leistungsnachweis (Anlage 7) sind die durchgeführten Leistungen durch Handzeichen der durchführenden Pflegekraft oder des SAPV Arztes zu bestätigen.

6. Zahlungen an eine durch das SAPV-Team/ KVMV ermächtigte Abrechnungsstelle/Verrechnungsstelle setzen voraus, dass der Krankenkasse eine Ermächtigungserklärung vorliegt. Zahlungen an eine Abrechnungsstelle erfolgen mit schuldbefreiender Wir-

[7] Datei am Ende der Anlage.

Muster

kung, es sei denn, der Krankenkasse liegt ein schriftlicher Widerruf des SAPV-Teams vor.

7. Maßgeblich für die Berechnung des Zahlungsziels ist der Tag, an dem alle zu einem Abrechnungsfall gehörenden Unterlagen bei der Krankenkasse bzw. bei den benannten Abrechnungsstellen vorliegen. Zusammengehörige Rechnungsunterlagen müssen in unmittelbarem zeitlichem Zusammenhang angeliefert werden. Die Krankenkasse behält sich vor, nicht korrekt gestellte Rechnungen unbezahlt abzuweisen.

8. Die Rechnungen sind grundsätzlich innerhalb von 28 Tagen nach Eingang bei der Krankenkasse bzw. bei den benannten Abrechnungsstellen zu begleichen. Als Zahltag gilt der Tag der Überweisung oder Übersendung von Zahlungsmitteln oder der Tag der Übergabe des Überweisungsauftrages an ein Geldinstitut. Ist der Zahltag kein Werktag, so verschiebt er sich auf den nachfolgenden Werktag.

9. Das SAPV-Team ist zu einer ordnungsgemäßen Abrechnung der erbrachten Leistungen gegenüber den Krankenkassen verpflichtet. Bei sachlichen und/oder rechnerischen Fehlern kann die Krankenkasse die strittigen Rechnungsbestandteile zur Berichtigung zurückgeben. Dies gilt auch für Rechnungen, die den Anforderungen dieser Vorschrift, trotz eines schriftlichen Hinweises, nicht entsprechen. Die Zahlungsfrist gilt in diesen Fällen nicht.

10. Werden Nachberechnungen gestellt, sind der Nachberechnung Kopien der Erstrechnung und der Verordnung beizulegen. Darauf ist "Nachberechnung" und das Datum der Erstrechnung zu vermerken.

11. Bei Differenzen bzw. begründeten Beanstandungen der Abrechnung kann die Krankenkasse dem SAPV-Team die strittigen eingereichten Unterlagen oder die strittigen Datensätze zur Prüfung bzw. Korrektur zurückgeben. Beanstandungen müssen von der Krankenkasse innerhalb von 12 Monaten nach Rechnungseingang schriftlich geltend gemacht werden. Rückforderungen können nur mit dem Einverständnis des SAPV-Teams / KVMV verrechnet werden.

12. Rechnungen können bis spätestens 12 Monate nach Abschluss der Leistungserbringung eingereicht werden.

13. Der Versicherte hat die Abgabe der verordneten Leistungen einmal wöchentlich durch Unterschrift auf dem Leistungsnachweis (Anlage 7) zu bestätigen. Quittierungen im Voraus sind unzulässig.

14. Ist der Versicherte nicht in der Lage, den Leistungsnachweis (Anlage7) selbst zu unterschreiben, kann die Unterschrift durch einen Vertretungsberechtigten erfolgen. Hierüber ist die Krankenkasse in der Abrechnung schriftlich zu unterrichten. Kann die Unterschrift im Ausnahmefall weder durch den Versicherten noch durch einen Vertretungsberechtigten erbracht werden, bleibt das Unterschriftsfeld auf dem Leistungsnachweis frei. Der Leistungserbringer gibt auf dem Leistungsnachweis, ggf. auf einer Anlage zum Leistungsnachweis, die Gründe hierfür an. Eine Unterschrift für den Versicherten durch den Leistungserbringer oder seine Mitarbeiter ist ausgeschlossen.

15. Werden Leistungen für Versicherte sowohl mit der Pflegekasse (Leistungsrecht nach SGBXI) als auch mit der Krankenkasse abgerechnet, sind diese Rechnungen getrennt einzureichen.

Muster

## Abrechnung der Leistungen nach Einführung DTA (Vereinbarung zu § 302 SGB V (Datenaustausch)

1. Das SAPV-Team kann seine Leistungen nur abrechnen, wenn es über ein gültiges IK verfügt.

2. Für die Abrechnung gelten grundsätzlich die Richtlinien der Spitzenverbände der Krankenkassen über Form und Inhalt des Abrechnungsverfahrens nach § 302 Abs. 2 SGB V in der jeweils aktuellen Fassung mit den dazu gehörigen Anlagen.

3. Die Abrechnung hat unter Berücksichtigung des § 303 SGB V insbesondere folgende Bestandteile:
   - Abrechnungsdaten,
   - Urbelege: - Leistungszusagen der Krankenkassen in Kopie<br>- unterzeichneter Leistungsnachweis
   - Gesamtaufstellung der Abrechnung (Gesamtrechnung, ggf. Sammelrechnung),
   - Begleitzettel für Urbelege (bei maschineller Abrechnung).

4. Über den Einführungstermin des maschinellen Abrechnungsverfahrens entscheidet die jeweilige Krankenkasse. Ab dem Zeitpunkt der Einführung sind die folgenden Abrechnungsanforderungen zu beachten. Abrechnungen, die nicht den Richtlinien nach § 302 SGB V entsprechen, werden ab diesem Zeitpunkt von den Krankenkassen abgewiesen.

5. Das SAPV-Team/ KVMV ist verpflichtet, sich vor der erstmaligen Datenlieferung bei der jeweiligen Krankenkasse anzumelden:

   - AOK = AOK Nordost, Am Grünen Tal 50, 19063 Schwerin
   - BKK BKK-Vertragsarbeitsgemeinschaft NORD = Anlistung der teilnehmenden Krankenkassen
   - IKK Nord = Moislinger Allee 19a, 23558 Lübeck
   - LKK Mittel- und Ostdeutschland= Hoppegartener Str. 100, 15366 Hoppegarten
   - Knappschaft = Millerntorplatz 1, 20359 Hamburg
   - vdek = Anlistung der einzelnen Mitgliedskassen[8]

6. Dies gilt auch, wenn ein Abrechnungszentrum mit der Erstellung der Abrechnung beauftragt wurde. Die Abgabe gilt als Anmeldung zum Datenaustausch.

7. Zur Sicherstellung der Vergütung erbrachter Leistungen ist bei der Aufnahme des maschinellen Abrechnungsverfahrens vom SAPV-Team/ KVMV zunächst eine Erprobungsphase mit den einzelnen Krankenkassen bzw. bei den benannten Abrechnungsstellen durchzuführen. In der Erprobungsphase erfolgt eine parallele Übermittlung von maschinellen Abrechnungsdaten sowie Papierabrechnungen nach dem bisherigen Abrechnungsverfahren. Dabei sind die maschinellen Daten als Testdaten zu kennzeichnen. Die maschinellen Abrechnungsdaten und die Papierabrechnungen müssen identisch und vergleichbar sein.

8. Mit der ersten Datenlieferung beginnt die sogenannte „Erprobungsphase. Die Erprobungsphase ist abgeschlossen, wenn drei Abrechnungen je SAPV- Team fehlerfrei geliefert wurden.

9. Das SAPV-Team/ KVMV kann die Erprobungsphase mit der Krankenkasse bzw. bei den benannten Abrechnungsstellen beenden, wenn es der datenannehmenden Stelle der Krankenkasse dreimal hintereinander technisch und inhaltlich einwandfreie maschinelle

[8] Datei am Ende der Anlage.

Daten übermittelt hat. Dies gilt dann als erfüllt, wenn die betreffende Krankenkasse dem SAPV-Team/ KVMV keine Rückmeldung über Fehler in den Daten gibt.

10. Nach der Beendigung der Erprobungsphase werden vom SAPV-Team/ KVMV ausschließlich maschinell verwertbare Datenträger übermittelt. Als maschinell verwertbar in diesem Sinne gelten dabei ausschließlich Daten auf elektronischen Datenträgern nach der Technischen Anlage zu den Richtlinien zum Datenaustausch der Spitzenverbände der Krankenkassen . Die Daten sind als "Echtdaten" zu kennzeichnen.

11. Erfolgt die Abrechnung des SAPV-Teams/ KVMV in begründeten Fällen übergangsweise nicht auf maschinellen Datenträgern, sondern auf standardisierten Abrechnungsformularen der Richtlinien, verlängert sich die Zahlungsfrist auf 30 Tage nach Eingang der vollständigen Abrechnungsunterlagen (Abrechnungsformulare und rechnungsbegründende Unterlagen). Dies ist notwendig, da die Daten vor einer Bearbeitung durch die zuständige Krankenkasse von den standardisierten Abrechnungsformularen in maschinell verwertbare Datensätze umgewandelt werden müssen.

12. Wenn das SAPV-Team/ KVMV unbegründet nach Aufforderung durch die Krankenkassen immer noch nicht mit der Datenlieferung begonnen hat, erfolgt die Umsetzung des § 303 Abs. 3 SGB V durch die Krankenkasse.

13. Die Rechnungslegung erfolgt für alle Versorgungs-/Abrechnungsfälle einmal im Quartal. Die maschinell verwertbaren Daten sind an die von den Krankenkassen benannten Stellen zu liefern.

14. Es werden nur syntaktisch einwandfreie Daten gemäß den Richtlinien angenommen. Fehlerhafte Datenlieferungen sowie nicht korrekt vom SAPV-Team ausgefüllte Urbelege werden an den Absender mit einem entsprechenden Fehlerhinweis zurückgesendet. Die Unterlagen sind im Original in der in den Richtlinien beschriebenen Sortierreihenfolge zu übermitteln. Die Leistungsnachweise sind den zugrunde liegenden Verordnungen zuzuordnen. Nicht ordnungsgemäße oder fehlerhafte Angaben auf den Urbelegen führen zur Abweisung der Rechnung. Den rechnungsbegründenden Unterlagen ist bei maschineller Abrechnung ein Begleitzettel gemäß § 2 Abs. 1 Buchstabe f) der Richtlinie zum Datenaustausch beizufügen.

15. Der Versicherte hat die Abgabe der verordneten Leistungen einmal wöchentlich durch Unterschrift auf dem Leistungsnachweis (Anlage 7) zu bestätigen. Quittierungen im Voraus sind unzulässig.

16. Ist der Versicherte nicht in der Lage, den Leistungsnachweis (Anlage 7) selbst zu unterschreiben, kann die Unterschrift durch einen Vertretungsberechtigten erfolgen. Hierüber ist die Krankenkasse in der Abrechnung schriftlich zu unterrichten. Kann die Unterschrift im Ausnahmefall weder durch den Versicherten noch durch einen Vertretungsberechtigten erbracht werden, bleibt das Unterschriftsfeld auf dem Leistungsnachweis frei. Der Leistungserbringer gibt auf dem Leistungsnachweis, ggf. auf einer Anlage zum Leistungsnachweis, die Gründe hierfür an. Eine Unterschrift für den Versicherten durch den Leistungserbringer oder seine Mitarbeiter ist ausgeschlossen.

17. Die Rechnung muss folgende Mindestinhalte aufweisen:
    - Name, Anschrift und Institutionskennzeichen des SAPV- Teams
    - Name und Vorname, Krankenversicherungsnummer, Geburtsdatum des Versicherten,
    - Gebührenpositionsnummer, Art und Anzahl der erbrachten Leistungen
    - Datum und Uhrzeit des Einsatzes (Ende und Beginn)
    - Gesamtbetrag
    - Rechnungsnummer

Muster

- Rechnungsdatum
- Leistungserbringerschlüssel (sobald dieser bekannt ist)

18. Bei Differenzen bzw. begründeten Beanstandungen der Abrechnung kann die Krankenkasse dem SAPV-Team/ KVMV die strittigen eingereichten Unterlagen oder die strittigen Datensätze zur Prüfung bzw. Korrektur zurückgeben. Beanstandungen müssen von der Krankenkasse innerhalb von 12 Monaten nach Rechnungseingang schriftlich geltend gemacht werden. Rückforderungen können nur mit dem Einverständnis des SAPV-Teams/ KVMV verrechnet werden.

19. Die Bezahlung der Rechnungen auf maschinell verwertbaren Datenträgern erfolgt innerhalb von 14 Tagen nach Eingang der vollständigen Abrechnungsunterlagen bei den von den Krankenkassen benannten Stellen. Bei Zahlung durch Überweisung gilt die Frist als gewahrt, wenn der Auftrag innerhalb dieser Zeit dem Geldinstitut erteilt wurde.

20. Überträgt das SAPV-Team/ KVMV die Abrechnung einer Abrechnungsstelle, so hat das SAPV-Team/ KVMV die Krankenkassen sowie die vdek-Landesvertretung unverzüglich schriftlich hierüber zu informieren. Der Beginn und das Ende des Auftragsverhältnisses, der Name der beauftragten Abrechnungsstelle und das Institutionskennzeichen, unter dem die Abrechnungsstelle die Rechnungslegung vornimmt, ist mitzuteilen. Das Abrechnungszentrum ist verpflichtet, sich ebenfalls gemäß Abs. 5 zum maschinellen Datenaustausch anzumelden. Abrechnungszentren liefern die Abrechnung ausschließlich auf maschinell verwertbaren Datenträgern nach Abs. 2.

21. Das SAPV-Team/KVMV ist für die Einhaltung der gesetzlichen und vertraglichen Regelungen durch das Abrechnungszentrum verantwortlich.

22. Hat das SAPV-Team/KVMV dem Abrechnungszentrum eine Inkasso-Vollmacht erteilt, erfolgt die Zahlung an das Abrechnungszentrum für die Krankenkassen mit schuldbefreiender Wirkung. Wird dem Abrechnungszentrum die Inkasso-Vollmacht entzogen, ist das SAPV-Team für die Einhaltung des Entzugs der Vollmacht verantwortlich.

23. Sofern die Rechnungslegung einer Abrechnungsstelle gemäß Absatz 20 übertragen werden soll, ist der Auftragnehmer unter besonderer Berücksichtigung der von ihm getroffenen technischen und organisatorischen Maßnahmen zur Sicherstellung der Maßgaben dieses Vertrages und des § 6 Abs. 1 BDSG durch das SAPV-Team auszuwählen. Die getroffene Vereinbarung über Datenschutz und Datensicherung beim Auftragnehmer (Abrechnungsstelle) ist den Krankenkassen vorzulegen. (neue Position)

**Abrechnungsstellen der Mitgliedskassen des vdek**

"SAPV_A5_Abrechnungsstellen vdek.xls"

Muster

Muster

Zusatz zur Anlage 5
Abrechnungsspezifische Besonderheiten der Krankenkassen

Leistungserbringergruppenschlüssel (LEGS) § 302 SGB V – Richtlinie für den Datenträgeraustausch (DTA) der Leistungserbringer mit den Krankenkassen sowie der Anlagen zur Richtlinie

Gemäß § 18 Abrechnung/ Beanstandung/ Verjährung Abs. 2 sowie der Anlage 5 des Vertrages sind sobald die Bestimmungen zur Umsetzung der Abrechnung über DTA nach § 302 SGB V für die Leistungserbringer der SAPV vorliegen, anzuwenden. Da einzelne Krankenkassen der Krankenkassenverbände/ des Verbands der Ersatzkassen e.V. den DTA anwenden bzw. die Abrechnung über eine Abrechnungsstelle erfolgt, übermitteln wir Ihnen im Rahmen des DTA einen Leistungserbringergruppenschlüssel (LEGS), der im Zuge der Abrechnung (auf der Abrechnung) an die Krankenkassen bzw. deren Abrechnungsstellen benannt werden muss.
Die Angabe des LEGS ist bei jeder Abrechnung zwingend erforderlich, da nur mit Hilfe dieses Schlüssels die für den jeweiligen Leistungserbringer gültige Preisliste ansteuerbar ist.

**Versorgungsbereich:**

⇨ **AC/ TK ..................**

Muster

## Anlage 6 Anforderungen an die Dokumentation

Vertrag über die spezialisierte ambulante Palliativversorgung gemäß § 37 b i.V.m. § 132 d SGB V zwischen der „...GbR" und Kassenärztliche Vereinigung Mecklenburg-Vorpommern und AOK Nordost - Die Gesundheitskasse, BKK-Vertragsarbeitsgemeinschaft NORD-, IKK Nord in Vertretung des IKK Landesverbandes Nord, LKK Mittel- und Ostdeutschland, Knappschaft, BARMER GEK, Techniker Krankenkasse, Deutsche Angestellten-Krankenkasse, KKH-Allianz, HEK - Hanseatische Krankenkasse, hkk, gemeinsamer Bevollmächtigter mit Abschlussbefugnis, Verband der Ersatzkassen e.V. (vdek), Berlin, vertreten durch den Leiter der Landesvertretung Mecklenburg-Vorpommern

### Das eingesetzte Dokumentationssystem beinhaltet die nachfolgenden Anforderungen:

<table>
<tr><th colspan="2">I. Kerndatensatz für Palliativpatienten:<br>Erfassung zu Beginn und Ende der Palliativversorgung</th></tr>
<tr><th>Items</th><th>Antworten, Kategorien</th></tr>
<tr><td colspan="2">Demographische Angaben</td></tr>
<tr><td>Geburtsdatum</td><td>Datum</td></tr>
<tr><td>Aufnahmedatum</td><td>Datum</td></tr>
<tr><td>Geschlecht</td><td>| weiblich | männlich</td></tr>
<tr><td>Wohnsituation</td><td>| allein | Heim | mit Angehörigen | Sonstige</td></tr>
<tr><td colspan="2">Angaben zur Erkrankung</td></tr>
<tr><td>Hauptdiagnose/Grundkrankheit<br>(palliativmedizinisch relevant)</td><td>ICD</td></tr>
<tr><td>Funktionsstatus (ECOG)</td><td>| Normale Aktivität | Gehfähig, leichte Arbeit möglich<br>| Nicht arbeitsfähig, kann > 50% der Wachzeit aufstehen<br>| Begrenzte Selbstversorgung, > 50% Wachzeit bettlägerig<br>| Pflegebedürftig, permanent bettlägerig</td></tr>
<tr><td colspan="2">Angaben zur Versorgung (Strukturqualität)</td></tr>
<tr><td>Behandlung, Begleitung<br>(bei Behandlungsbeginn<br>oder bei Übernahme des Patienten)</td><td>ambulant:<br>| SAPV-Team | Hausarzt | Ambulante Pflege<br>| Palliativarzt (QPA) | Palliativpflege (AHPP, APD)<br>| Palliativberatung (AHPB) | Ehrenamtlicher Dienst<br>stationär:<br>| KH (Allgemeinstation) | KH (Palliativstation)<br>| Heim | Stationäres Hospiz | Sonstige</td></tr>
<tr><td>Absprache / Vorlage</td><td>| Patientenverfügung | Vorsorgevollmacht | Betreuung</td></tr>
<tr><td>Datum der Erfassung</td><td>Datum</td></tr>
<tr><td colspan="2">Angaben zum Behandlungsbedarf</td></tr>
<tr><td>Schmerzen</td><td>| kein | leicht | mittel | stark</td></tr>
<tr><td>Übelkeit</td><td>| kein | leicht | mittel | stark</td></tr>
<tr><td>Erbrechen</td><td>| kein | leicht | mittel | stark</td></tr>
<tr><td>Luftnot</td><td>| kein | leicht | mittel | stark</td></tr>
<tr><td>Verstopfung</td><td>| kein | leicht | mittel | stark</td></tr>
<tr><td>Schwäche</td><td>| kein | leicht | mittel | stark</td></tr>
<tr><td>Appetitmangel</td><td>| kein | leicht | mittel | stark</td></tr>
<tr><td>Müdigkeit</td><td>| kein | leicht | mittel | stark</td></tr>
<tr><td>Pflegeprobleme wegen Wunden / Dekubitus</td><td>| kein | leicht | mittel | stark</td></tr>
<tr><td>Hilfebedarf bei Aktivitäten des tägl. Lebens</td><td>| kein | leicht | mittel | stark</td></tr>
<tr><td>Depressivität</td><td>| kein | leicht | mittel | stark</td></tr>
<tr><td>Angst</td><td>| kein | leicht | mittel | stark</td></tr>
<tr><td>Anspannung</td><td>| kein | leicht | mittel | stark</td></tr>
<tr><td>Desorientiertheit, Verwirrtheit</td><td>| kein | leicht | mittel | stark</td></tr>
<tr><td>Probleme mit Organisation der Versorgung</td><td>| kein | leicht | mittel | stark</td></tr>
<tr><td>Überforderung der Familie, des Umfeldes</td><td>| kein | leicht | mittel | stark</td></tr>
<tr><td colspan="2">Angaben zur Therapie (Prozessqualität)</td></tr>
<tr><td>Was ist der Grund des Kontaktes, was das Behandlungsziel?</td><td>Text</td></tr>
<tr><td>Opioide der WHO Stufe III</td><td>ja / nein</td></tr>
<tr><td>Steroide</td><td>ja / nein</td></tr>
<tr><td>Chemotherapie</td><td>nein / fortgesetzt / initiiert</td></tr>
<tr><td>Strahlentherapie</td><td>nein / fortgesetzt / initiiert</td></tr>
<tr><td>besonderer Aufwand mit:</td><td>Text</td></tr>
</table>

| **Angaben zum weiteren Verlauf, Abschluss (Ergebnisqualität)** | |
|---|---|
| Welches Problem konnte besonders gut gelöst werden? | Text |
| Welches Problem konnte nicht ausreichend gelöst werden? | Text |
| Datum (Entlassung / Änderung der Betreuung / Tod) | Datum |
| Therapieende | \| Verstorben \| Verlegung, Entlassung<br>\| Sonstiges: |
| Sterbeort | \| zuhause \| Heim \| Krankenhaus<br>\| Palliativstation \| Hospiz \| unbekannt |
| Zufriedenheit mit der Behandlung für die gesamte Behandlung | \| sehr schlecht \| schlecht \| mittel<br>\| gut \| sehr gut |

| II. SAPV-Struktur-Modul:<br>Erfassung bei jeder Verordnung bzw. Intensitätswechsel | |
|---|---|
| **Items** | **Antworten, Kategorien** |
| **Identifikationsnummer** | Fortlaufende Nr. wie bei Kerndatensatz |
| **Angaben zur Versorgung (Strukturqualität)** | |
| SAPV | \| Beratung \| Koordination<br>\| additive Teilversorgung \| vollständige Versorgung |
| Verordnung Datum | Datum |
| Art der Verordnung | \| Erstverordnung \| Wiederverordnung |
| Verordnung durch | \| Hausarzt \| niedergelassener Facharzt \| KH-Arzt<br>\| andere \| keine Angabe |
| Übernahme aus | \| Eigene Häuslichkeit \| Ambulantes Umfeld<br>\| Stationäre Pflegeeinrichtung \| Stationäres Hospiz<br>\| KH (Allgemeinstation) \| KH (Palliativstation) |
| Beteiligte assoziierte Partner in der SAPV-Erbringung | Ärztlich:<br>\| Hausarzt \| niedergelassener Facharzt (für_______)<br>\| KH-Arzt \| Sonstige<br><br>Pflegerisch:<br>\| Ambulanter Pflegedienst<br>\| Ambulanter Palliativpflegedienst<br>\| Stationäre Pflegeeinrichtung<br>\| Stationäres Hospiz<br><br>Ambulanter Hospizdienst:<br>\| Ambulante Palliativberatung (AHD, AHPB)<br>\| Begleitung durch Ehrenamtliche<br><br>Weitere Professionen:<br>\| Case Management \| Ernährungsberatung<br>\| Physiotherapie \| Psychologie \| Seelsorge<br>\| Sozialarbeit \| Andere:_______________ |
| Regel-Anfahrtsweg in km | |
| **Angaben zum Verlauf** | |
| Anzahl der Teambesprechungen zur Problemlösung (Definition Teambesprechung in Expose) | |
| Krankenhauseinweisungsgrund | Text |
| Datum Ende der SAPV | |
| **Angaben zum Team** | |
| Zahl der Vollzeitäquivalente (multiprofessionell) im SAPV Team | |
| Zahl der zeitgleich maximal betreuten Patienten je Versorgungsstufe | |
| Datum der Erfassung | |

Anl



Muster

sche Krankenkasse, hkk, gemeinsamer Bevollmächtigter mit Abschlussbefugnis, Verband der Ersatzkassen e.V. (vdek), Berlin, vertreten durch den Leiter der Landesvertretung Mecklenburg-Vorpommern

**SAPV_Rostock_A_7 Leistungsnachweise**

**Protokollnotiz 1**

§ 10 Abs. 4 Buchstabe a

Alternativ zum Nachweis der Zusatzbezeichnung "Palliativmedizin" kann nach Erfüllung der Voraussetzungen für einen Übergangszeitraum die Anmeldung zur Prüfung bei der Ärztekammer nachgewiesen werden.

Die Vertragspartner sind über das Prüfungsergebnis umgehend zu informieren.

**Protokollnotiz 2**

Evaluation – Struktur- und Leistungskennzahlen

(1) Die Vertragspartner evaluieren die vorliegenden Daten der SAPV-Leistung. Sie verständigen sich gemeinsam darauf, die Strukturen der Leistungen zu überprüfen und Transparenz bezüglich der Datenlage zu schaffen. Den Krankenkassen sind die hierfür notwendigen statistische Angaben (Struktur- und Leistungskennzahlen gemäß § 21 des Vertrages) zur Verfügung zu stellen.

(2) Die Vertragspartner beraten sich u. a. zu Fragen der praktischen Umsetzung, Qualitätssicherung und Bewertung der laufenden Vertragsergebnisse.

**Protokollnotiz 3**

Auf Seiten des Leistungserbringers besteht die ausdrückliche Bereitschaft, den an der vertragsärztlichen Versorgung teilnehmenden Ärzten einen Zugang zu Hospitationen zu ermöglichen.

North Rhine

# Vertrag über die Erbringung

**Spezialisierter ambulanter Palliativversorgung (SAPV) in Nordrhein**
**gemäß § 132 d SGB V i. V. m. § 37 b SGB V**

**zwischen dem Palliative-Care-Team „Muster“, Rechtsform, Sitz, vertreten durch ...**

- nachfolgend Palliative-Care-Team genannt -

und

**der Kassenärztlichen Vereinigung Nordrhein**

- nachfolgend KV genannt -

und

**der AOK Rheinland/Hamburg - Die Gesundheitskasse**
**der BKK-Vertragsarbeitsgemeinschaft Nordrhein-Westfalen (BKK-VAG NRW)**
**der IKK Nordrhein**
**der Landwirtschaftlichen Krankenkasse Nordrhein-Westfalen**
**zugleich handelnd für die Krankenkasse für den Gartenbau**
**der Knappschaft**

sowie den Ersatzkassen

**BARMER Ersatzkasse**
**Techniker Krankenkasse**
**Deutsche Angestellten-Krankenkasse**
**Kaufmännische Krankenkasse - KKH - Allianz**
**Gmünder Ersatzkasse**
**HEK - Hanseatische Krankenkasse**
**Hamburg Münchener Krankenkasse**
**hkk**

gemeinsamer Bevollmächtigter mit Abschlussbefugnis
gemäß § 212 Abs. 5 S. 6 SGB V:

**Verband der Ersatzkassen e. V. (vdek),**
vertreten durch den Leiter der Landesvertretung Nordrhein-Westfalen

- nachfolgend Krankenkassen genannt -

wird folgender Vertrag geschlossen:

## Inhaltsverzeichnis

**Präambel**

§ 1 Gegenstand und Ziele des Vertrages

§ 2 Geltungsbereich

§ 3 Anspruchsvoraussetzungen der SAPV

§ 4 Inhalt und Umfang der SAPV

§ 5 Pflichten der Vertragspartner

§ 6 Palliative-Care-Team/Versorgungskonzept

§ 7 Organisatorische, personelle und sächliche Voraussetzungen an das Palliative-Care-Team

§ 8 Weitere Koordinationsaufgaben und Anforderungen

§ 9 Qualitätssicherung und Dokumentation

§ 10 Anzeigepflichten/Mitteilungspflichten

§ 11 Verordnung im Rahmen der SAPV

§ 12 Verordnung/Genehmigung der SAPV

§ 13 Vorläufige Kostenzusage

§ 14 Grundsätze der Leistungserbringung

§ 15 Fort- und Weiterbildung

§ 16 Vergütung

§ 17 Abrechnung

§ 18 Leistungsnachweis

§ 19 Einflussnahme-, Werbungs- und Vermittlungsverbot

§ 20 Zuzahlungen

§ 21 Datenschutz, Schweigepflicht

§ 22 Statistik

§ 23 Vertrags-/Gesetzesverstöße

§ 24 Außerordentliche Kündigung

§ 25 Inkrafttreten, Ordentliche Kündigung

§ 26 Änderungen und Ergänzungen

§ 27 Salvatorische Klausel

**Anlagen**

1) Strukturerhebungsbogen des Palliative-Care-Teams „Name“
2) Nachweis und Handzeichenliste
3) Patienteninformation und Entbindung von der Schweigepflicht
4) Vergütungsvereinbarung
5) „Richtlinie des Gemeinsamen Bundesausschusses zur Verordnung von spezialisierter ambulanter Palliativversorgung“ (Spezialisierte Ambulante Palliativversorgungs-Richtlinie/SAPV-RL)
6) Gemeinsame Empfehlung der Spitzenverbände der Krankenkassen nach § 132 d Abs. 2 SGB V für die spezialisierte ambulante Palliativversorgung
7) Dokumentation
8) Vertrag über die ambulante palliativpflegerische Versorgung nach § 132 a Abs. 2 SGB V
9) Schnellbegutachtungsbogen des Medizinischen Dienstes der Krankenversicherung Nordrhein
10) Leistungsnachweis

## Präambel

Das Ziel der spezialisierten ambulanten Palliativversorgung (SAPV) ist, den Patientinnen und Patienten zu ermöglichen, die letzte Phase ihres Lebens in häuslicher Umgebung zu verbringen. Mit diesem Vertrag soll dem sterbenden Menschen ein würdevolles und selbst bestimmtes Leben in häuslicher Umgebung ermöglicht werden, daher sollen alle an der Versorgung Beteiligten rund um die Uhr eine aufeinander abgestimmte effiziente und wirksame palliativmedizinische und palliativpflegerische Versorgung erbringen. Dieser Vertrag soll eine optimale spezialisierte ambulante Palliativversorgung gewährleisten und zugleich die Lebensqualität der Betroffenen und der Angehörigen erhöhen. Versorgungsunsicherheiten, die zu unnötigen und die Versicherten stark belastenden Krankenhauseinweisungen führen, sollen durch die in der SAPV vorgehaltenen besonderen Expertisen vermieden werden.
Bedarf nach einer spezialisierten ambulanten Palliativversorgung und damit besonderen aufwändigen Versorgung besteht, soweit die anderweitigen ambulanten Versorgungsformen, z. B. die allgemeine ambulante Palliativversorgung sowie ggf. Leistungen des ambulanten Hospizdienstes, nicht oder nur unter besonderer Koordination ausreichen würden.

## § 1
## Gegenstand und Ziele des Vertrages

(1) Die SAPV gemäß § 37 b SGB V dient dem Ziel, die Lebensqualität und die Selbstbestimmung schwerstkranker Menschen zu erhalten, zu fördern und zu verbessern und ihnen ein menschenwürdiges Leben bis zum Tod in ihrer vertrauten häuslichen Umgebung oder in stationären Pflegeeinrichtungen (§ 72 Abs. 1 SGB XI) zu ermöglichen. Im Vordergrund steht anstelle eines kurativen Ansatzes die palliativmedizinische sowie palliativpflegerische Zielsetzung, Symptome und Leiden einzelfallgerecht zu lindern.

(2) Die individuellen Bedürfnisse und Wünsche der Patientin und des Patienten sowie die Belange ihrer vertrauten Personen stehen im Mittelpunkt der Versorgung. Der Patientenwille, der auch durch eine Patientenverfügung oder einem Behandlungsplan zum Ausdruck kommt und allen Beteiligten (Ärztin/Arzt, Angehörigen etc.) bekannt sein muss, ist zu beachten.

(3) Die SAPV ergänzt das bereits bestehende Versorgungsangebot, insbesondere das der Vertragsärzte, Krankenhäuser und Pflegedienste einschließlich der Versorgungsangebote der allgemeinen ambulanten palliativmedizinischen Versorgung. Je nach Umfang einer Patienten-Teilversorgung, vor allem aber bei der Vollversorgung, ersetzt die SAPV die Regelversorgung nach dem SGB V weitgehend oder vollständig. Sie kann als alleinige Beratungs- und Koordinationsleistung, additiv unterstützende Teilversorgung oder vollständige Patientenbetreuung erbracht werden. Andere Sozialleistungsansprüche bleiben unberührt.

(4) Wenn Kinder versorgt werden, müssen die Anforderungen an die ärztlichen und pflegerischen Personen und an die weiteren Fachkräfte den besonderen Belangen der Kinder gerecht werden. Darüber hinaus besteht die Möglichkeit, dass die Krankenkassen gesonderte Verträge schließen.

(5) Die Anlagen 1 bis 10 sind in der jeweils gültigen Fassung Bestandteil dieses Vertrages.

## § 2
## Geltungsbereich

(1) Dieser Vertrag gilt für die im Rubrum genannten Vertragspartner in der im Versorgungskonzept (Anlage 1) genannten Region. Ein Beitritt weiterer Vertragspartner ist nur möglich, wenn alle Vertragspartner schriftlich zustimmen.

(2) Die BKK-Vertragsarbeitsgemeinschaft NRW (BKK-VAG NRW) unterzeichnet diesen Vertrag stellvertretend für die teilnehmenden Betriebskrankenkassen im Land Nordrhein-Westfalen. Sie stellt der KV unverzüglich eine Liste der teilnehmenden Betriebskrankenkassen zur Verfügung.

## § 3
## Anspruchsvoraussetzungen der SAPV

(1) Die Leistungserbringung setzt gemäß SAPV-Richtlinie voraus, dass Versicherte

a) an einer nicht heilbaren, fortschreitenden und so weit fortgeschrittenen Erkrankung leiden, dass dadurch ihre Lebenserwartung auf wenige Tage, Wochen oder Monate begrenzt ist und

b) unter Berücksichtigung der in § 1 genannten Ziele eine besonders aufwändige Versorgung benötigen, die nach den medizinischen und pflegerischen Erfordernissen auch ambulant oder in stationären Pflegeeinrichtungen (§ 72 SGB XI) erbracht werden kann.

(2) Eine Erkrankung ist nicht heilbar, wenn nach dem allgemein anerkannten Stand der medizinischen Erkenntnisse Behandlungsmaßnahmen nicht zur Beseitigung dieser Erkrankung führen können.

(3) Sie ist fortschreitend, wenn ihr Verlauf trotz medizinischer Maßnahmen nach dem allgemein anerkannten Stand der medizinischen Erkenntnisse nicht nachhaltig aufgehalten werden kann.

(4) Eine Erkrankung ist weit fortgeschritten, wenn die Verbesserung von Symptomatik und Lebensqualität sowie die psychosoziale Betreuung im Vordergrund der Versorgung stehen und nach begründeter Einschätzung der verordnenden Ärztin/des verordnenden Arztes die Lebenserwartung auf wenige Tage, Wochen oder Monate gesunken ist.

(5) Bedarf nach einer besonders aufwändigen Versorgung besteht, soweit die anderweitigen ambulanten Versorgungsformen einschließlich der allgemeinen ambulanten Palliativversorgung sowie ggf. die Leistungen des ambulanten Hospizdienstes nicht oder nur unter besonderer Koordination ausreichen würden, um die Ziele nach § 1 Abs. 1 zu erreichen. Anhaltspunkt dafür ist das Vorliegen eines komplexen Symptomgeschehens, dessen Behandlung spezifische palliativmedizinische und/oder palliativpflegerische Kenntnisse und Erfahrungen sowie ein interdisziplinär, insbesondere zwischen Ärzten und Pflegekräften in besonderem Maße abgestimmtes Konzept voraussetzt. Ein Symptomgeschehen ist in der Regel komplex, wenn mindestens eines der nachstehenden Kriterien erfüllt ist:

- ausgeprägte Schmerzsymptomatik
- ausgeprägte neurologische/psychiatrische/psychische Symptomatik
- ausgeprägte respiratorische/kardiale Symptomatik
- ausgeprägte gastrointestinale Symptomatik
- ausgeprägte ulzerierende/exulzerierende Wunden oder Tumore
- ausgeprägte urogenitale Symptomatik

(6) Liegt ein besonders aufwändiger Versorgungsbedarf nicht vor, besteht kein Anspruch auf SAPV.

(7) Die Versicherte/der Versicherte ist in der Wahl des Leistungserbringers frei.

## § 4
## Inhalt und Umfang der SAPV

(1) Die SAPV umfasst je nach Bedarf alle Leistungen der ambulanten Krankenbehandlung, soweit diese erforderlich sind, um die in § 1 genannten Ziele zu erreichen. Sie umfasst zusätzlich die im Einzelfall erforderliche Koordination der diagnostischen, therapeutischen und pflegerischen Teilleistungen sowie die Beratung, Anleitung und Begleitung der verordnenden oder behandelnden Ärztin/des verordnenden oder behandelnden Arztes sowie der sonstigen an der allgemeinen Versorgung beteiligten Leistungserbringer, der Patientinnen/der Patienten und ihrer Angehörigen durch Leistungserbringer nach § 132 d SGB V.

(2) SAPV wird ausschließlich von Leistungserbringern nach § 132 d SGB V erbracht.

Sie wird nach Bedarf intermittierend oder durchgängig erbracht, soweit das bestehende ambulante Versorgungsangebot (§ 1 Abs. 3), insbesondere die allgemeine Palliativversorgung nicht ausreicht, um die Ziele nach § 1 Abs. 1 zu erreichen. Sie kann dem jeweiligen aktuellen Versorgungsbedarf entsprechend als

I. Beratungsleistung

   a. Telefonat bzw. persönliches Gespräch mit Betroffenen (Patientin/Patienten oder deren Angehörigen)
   b. Telefonat bzw. persönliches Gespräch mit einem der Leistungserbringer der Primärversorgung
   c. Dokumentation und Evaluation

II. Koordination der Versorgung

   a. Direkter persönlicher Kontakt erforderlich
   b. Ressourcenfokussierte Versorgungsplanung
   c. Erstellung eines Versorgungskonzeptes
   d. Vernetzung mit ambulanten und/oder stationären Leistungserbringer
   e. Dokumentation und Evaluation

III. Additiv unterstützende Teilversorgung

   a. Beinhaltet Beratungs- und Koordinationsleistung
   b. Hausbesuche erforderlich
   c. Einzelne Leistungen des in § 5 Abs. 3 der SAPV-Richtlinie des G-BA aufgeführten Leistungskataloges werden bei Bedarf erbracht
   d. Für die verordnete Teilversorgung besteht 24-Stunden-Rufbereitschaft. Die Rufbereitschaft kann nicht als alleinige Teilleistung verordnet werden.
   e. Dokumentation und Evaluation

IV. Vollständige Versorgung

   a. Hausbesuche erforderlich
   b. Beinhaltet Beratungs- und Koordinationsleistung
   c. Einzelne Leistungen des in § 5 Abs. 3 der SAPV-Richtlinie des G-BA aufgeführten Leistungskataloges werden bei Bedarf erbracht
   d. Grundpflege nach § 37 SGB V bzw. SGB XI ist nicht Bestandteil der SAPV, kann aber bei entsprechenden Voraussetzungen vom Palliative-Care-Team erbracht und über § 37 Abs. 1 SGB V oder das SGB XI abgerechnet werden

e. Für die verordnete Vollversorgung besteht 24-Stunden-Rufbereitschaft. Die Rufbereitschaft kann nicht als alleinige Teilleistung verordnet werden
f. Dokumentation und Evaluation

erbracht werden. Die Leistungen müssen ausreichend und zweckmäßig sein, dürfen das Maß des Notwendigen nicht überschreiten und sind wirtschaftlich zu erbringen.

(3) Inhalte der SAPV sind insbesondere:

- Koordination der spezialisierten ambulanten palliativmedizinischen und palliativpflegerischen Versorgung unter Einbeziehung weiterer Berufsgruppen und von Hospizdiensten im Rahmen einer multiprofessionellen Zusammenarbeit
- Symptomlinderung durch Anwendung von Medikamenten oder anderen Maßnahmen
- Apparative palliativmedizinische Behandlungsmaßnahmen (z. B. Medikamentenpumpe)
- Palliativmedizinische Maßnahmen, die nach ihrer Art, Schwere oder Komplexität eine Kompetenz erfordern, die der einer Ärztin/eines Arztes mit Zusatzweiterbildung Palliativmedizin entspricht
- Spezialisierte palliativpflegerische Leistungen, die nach ihrer Art, Schwere oder Komplexität eine Kompetenz erfordern, die der einer Pflegefachkraft mit einer curricularen Weiterbildung zu Palliative Care entspricht
- Führung eines individuellen Behandlungsplans, vorbeugendes Krisenmanagement, Bedarfsinterventionen
- Ruf-, Notfall- und Kriseninterventionsbereitschaft rund um die Uhr für die im Rahmen der SAPV betreuten Patienten zur Sicherstellung der im Rahmen der SAPV erforderlichen Maßnahmen
- Beratung, Anleitung und Begleitung der Patientinnen/der Patienten und ihrer Angehörigen zur palliativen Versorgung einschließlich Unterstützung beim Umgang mit Sterben und Tod
- Spezialisierte Beratung der betreuenden Leistungserbringer der Primärversorgung
- Psychosoziale Unterstützung im Umgang mit schweren Erkrankungen in enger Zusammenarbeit, z. B. mit Seelsorge, Sozialarbeit und ambulanten Hospizdiensten
- Organisation regelmäßiger Fallbesprechungen

- Dokumentieren und Evaluieren der wesentlichen Maßnahmen im Rahmen der SAPV

## § 5
## Pflichten der Vertragspartner

(1) Aufgabe des Palliative-Care-Teams ist es, Leistungen der SAPV durch spezialisierte Leistungserbringer, die sich zu einem Palliative-Care-Team zusammengeschlossen haben und Teil einer multiprofessionell vernetzen Versorgungsstruktur im regionalen Gesundheits- und Sozialsystem sind, zu erbringen. Ein Vergütungsanspruch besteht daher erst, wenn das Palliative-Care-Team die nachfolgend formulierten Voraussetzungen erfüllt und die Prüfung, ob das Palliative-Care-Team geeignet und bedarfsgerecht ist, durch die KV und die zuständige Krankenkasse abgeschlossen ist und dem Palliative-Care-Team das Ergebnis dieser Prüfung mitgeteilt wurde. Erst dann ist das Palliative-Care-Team berechtigt, Leistungen der SAPV zu erbringen und erhält dann die hierfür vereinbarte Vergütung.

(2) Aufgabe der KV ist es, für die Krankenkassen zu prüfen, ob das Palliative-Care-Team die personellen Anforderungen an die Qualifikation der Ärztinnen/der Ärzte erfüllt, die nachfolgend geregelt sind. Die KV übermittelt das Ergebnis der Prüfung an die Krankenkassen.

(3) Aufgabe der Krankenkasse ist es, zu prüfen, ob das Palliative-Care-Team zur Erreichung der in § 1 genannten Ziele geeignet und bedarfsgerecht ist. Die Krankenkassen prüfen die Geeignetheit des Palliative-Care-Teams anhand der nachfolgend formulierten Voraussetzungen. Darüber hinaus prüfen die Krankenkassen die Bedarfsnotwendigkeit. Um prüfen zu können, ob das Palliative-Care-Team bedarfsnotwendig ist, sind die Kriterien der Gemeinsamen Empfehlung der Spitzenverbände der Krankenkassen nach § 132 d Abs. 2 SGB V für die spezialisierte ambulante Palliativversorgung vom 23.06.2008 anzuwenden. Eine Abdeckung von <u>250.000 Einwohnern</u> pro Palliative-Care-Team ist grundsätzlich als ausreichend zu betrachten; Ausnahmen sind allenfalls in ländlichen Gebieten möglich, wenn anzunehmen ist, dass der Bedarf durch ein Palliative-Care-Team nicht ausreichend gesichert ist. Sofern eine bedarfsgerechte Versorgung von SAPV durch die bereits tätigen Palliative-Care-Teams gegeben ist, besteht kein Anspruch auf Vertragsabschluss in der jeweiligen Region.

(4) Die zuständige Krankenkasse teilt das Ergebnis der Prüfung der Geeignetheit und Bedarfsnotwendigkeit dem Palliative-Care-Team sowie der KV schriftlich mit. Sofern das Palliative-Care-Team geeignet und bedarfsgerecht ist, erhält es unter den nachfolgend formulierten Voraussetzungen die in diesem Vertrag vereinbarte Vergütung.

## § 6
## Palliative-Care-Team/Versorgungskonzept

(1) Die Leistungen der SAPV sind nur dann Vertragsgegenstand, wenn sie ausschließlich in einer multiprofessionell vernetzten Versorgungsstruktur erbracht werden. Die Personalstärke eines Palliative-Care-Teams bestimmt sich u. a. durch die Anforderungen zur Erbringung ärztlicher und pflegerischer Leistungen, der 24-Stunden-Erreichbarkeit für betreute Versicherte, der qualifizierten Vertretung in Urlaubs- und Krankheitszeiten und der regionalen strukturellen Eigenheiten, die u. a. Fahrtzeiten betreffen könnten. Sie ergibt sich dennoch grundsätzlich aus der regionalen Struktur. Die Anpassung der Personalstärke auf regionale Erfordernisse regeln die Vertragspartner. Das Palliative-Care-Team besteht aus mindestens 3 spezialisierten/qualifizierten Ärzten und mindestens 4 qualifizierten Pflegefachkräften, die fachübergreifend in Kooperation mit anderen Professionen eng zusammenarbeiten. Kooperationen mit mindestens einer Apotheke und mindestens einem ambulanten Hospizdienst, die die Voraussetzungen des § 7 Abs. 1 b) erfüllen, sind verpflichtend. Das Palliative-Care-Team ist ausschließlich oder schwerpunktmäßig in der SAPV tätig. Es gewährleistet eine weitgehende personelle Kontinuität und verfügt über eine besondere Ausstattung, die eine palliativmedizinische und palliativpflegerische Versorgung im Zusammenwirken mit den weiteren Leistungserbringern gewährleistet. Es muss in der Lage sein, in seinem gesamten Einzugsbereich sowohl ärztlich als auch pflegerisch eine aufsuchende Behandlung und Pflege sowohl tagsüber als auch nachts, an Wochenenden und Feiertagen in Form einer 24-Stunden-Bereitschaft zu gewährleisten. Die organisatorischen, personellen und sächlichen Voraussetzungen, die das Palliative-Care-Team ständig erfüllen muss, sind in § 7 dieses Vertrages definiert.

(2) Das Palliative-Care-Team hat der zuständigen Krankenkasse ein schriftliches Konzept vor Vertragsschluss vorzulegen. Das Konzept hat darzustellen, in welcher Form die Koordination im Palliative-Care-Team erfolgt. Hierzu muss das Palliative-Care-Team ein verbindlich strukturiertes und schriftlich dargelegtes Versorgungskonzept vorlegen, das alle medizinisch-pflegerischen Maßnahmen klar und eindeutig unter Beachtung von Maßnahmen zur Qualitätssicherung formuliert und darüber hinaus verdeutlicht, wie eine qualifizierte Leistungser-

bringung zu erfolgen hat, damit die in § 1 dieses Vertrages genannten Vertragsziele erreicht werden. Ein Vergütungsanspruch auf der Grundlage dieses Vertrages ist nur dann gegeben, wenn die zuständige Krankenkasse das vorgelegte Konzept für geeignet erachtet.

(3) Das Palliative-Care-Team handelt anhand eines verbindlichen, strukturierten und schriftlich dargelegten Versorgungskonzepts, das mindestens folgende Punkte beinhaltet:

- Standort des Palliative-Care-Teams
- Versorgungsgebiet
- Versorgungsprozess
- Kooperationspartner
- Kooperationsvereinbarungen im Versorgungsgebiet; verpflichtend mit mindestens einem ambulanten Hospizdienst nach § 39 a Abs. 2 SGB V, Seelsorge und Sozialarbeit zur Sicherung der psychosozialen Unterstützung sowie mindestens einer Apotheke, die die Voraussetzungen gem. § 7 Abs. 1 b) erfüllt
- Mitarbeiterkapazität (ggf. Stellenplan)
- Personelle Zuständig- und Verantwortlichkeiten (u. a. Weisungsbefugnisse, Weisungsgebundenheit u. Ä.)
- Qualifikationsnachweise des Palliative-Care-Teams gem. § 7 Abs. 2
- Sächliche Ausstattung gem. § 7 Abs. 3
- Qualitätssicherungsmaßnahmen gemäß § 9
- Konzept zur Sicherstellung der reibungslosen Versorgung mit Arznei-, Heil-, und Hilfsmitteln
- Konzept zur Sicherstellung der 24-Stunden-Rufbereitschaft
- Fallzahleinschätzung
- Fort- und Weiterbildungskonzept

## § 7
## Organisatorische, personelle und sächliche Voraussetzungen an das Palliative-Care-Team

### 1. Organisatorische Voraussetzungen

a) Das Palliative-Care-Team muss seine Organisationsform den Krankenkassen in geeigneter Form nachweisen. Hierzu ist insbesondere die Anlage 1 vollständig auszufüllen.

b) Das Palliative-Care-Team kooperiert mindestens mit einer Apotheke, um eine Lagerhaltung an Medikamenten für den Notfall sicherzustellen, die mindestens folgende Voraussetzungen erfüllt:

- Zulassung als Apotheke
- Vorhaltung von palliativmedizinisch relevanten Arzneimitteln
- Fortbildung zur Fachapothekerin/zum Fachapotheker für Offizin- oder onkologische Pharmazie

sowie mindestens mit einem ambulanten Hospizdienst, der die Voraussetzungen gem. § 39 a Abs. 2 SGB V erfüllt.

### 2. Personelle Voraussetzungen

a) Die Leistungen der SAPV werden ausschließlich durch geeignete Personen erbracht, welche die nachfolgend formulierten Qualifikationen erfüllen. Das Palliative-Care-Team stellt sicher, dass diese geeigneten Personen hauptsächlich für die Leistungen der SAPV zur Verfügung stehen. Das Palliative-Care-Team weist mindestens drei geeignete Ärztinnen/Ärzte sowie mindestens vier geeignete Pflegefachkräfte nach.

(1) Geeignete Ärztinnen/Ärzte nach Abs. 1 verfügen über folgende Qualifikationen:

- eine anerkannte Zusatzweiterbildung Palliativmedizin nach der aktuell gültigen Weiterbildung der jeweiligen Landesärztekammer (grds. 160 Std. Weiterbildung)

- Erfahrungen aus der ambulanten palliativen Behandlung von mindestens 75 Palliativpatienten, z. B. in der häuslichen Umgebung oder in einem stationären Hospiz, innerhalb der letzten drei Jahre oder aus einer mindestens einjährigen klinischen palliativmedizinischen Tätigkeit in einer Palliativabteilung in einem Krankenhaus innerhalb der letzten drei Jahre

(2) Das Palliative-Care-Team weist nach, dass mindestens vier im Rahmen dieses Vertrages tätige Pflegefachkräfte über folgende Qualifikation verfügen:

- die Erlaubnis zur Führung einer der Berufsbezeichnungen Gesundheits- und Krankenpflegerin/Gesundheits- und Krankenpfleger, Gesundheits- und Kinderkrankenpflegerin/Gesundheits- und Kinderkrankenpfleger oder Altenpflegerin/Altenpfleger (Berechtigung zur Führung der Berufsbezeichnung nach dem Gesetz über die Berufe in der Altenpflege vom 25.08.2003) entsprechend den gesetzlichen Bestimmungen in der jeweils gültigen Fassung
- den Abschluss einer Palliative-Care-Weiterbildungsmaßnahme im Umfang von mindestens 160 Stunden; die Weiterbildung hat inhaltlich den Vorgaben des Basiscurriculum Palliativpflege des Vereins zur Betreuung und Begleitung von Schwerstkranken und Tumorpatienten e. V., Pallia Med Verlag, Bonn, zu entsprechen
- Erfahrungen durch mindestens eine zweijährige praktische Tätigkeit als Pflegefachkraft in der Betreuung von Palliativpatienten in den letzten drei Jahren; davon mindestens sechsmonatige Mitarbeit in einer spezialisierten Einrichtung der Hospiz- und Palliativversorgung
- Ergänzend müssen die Voraussetzungen erfüllt sein, die gem. Anlage 8 ein ambulant palliativpflegerischer Dienst zu erfüllen hat

b) Soweit weitere Fachkräfte (z. B. Sozialarbeiterinnen/Sozialarbeiter, Sozialpädagoginnen/Sozialpädagogen, Psychologinnen/Psychologen) vertraglich eingebunden werden, z. B. im Rahmen des vorzulegenden Versorgungskonzepts als Kooperationspartner genannt sind, weist das Palliative-Care-Team nach, dass diese Fachkräfte über eine Zusatzweiterbildung Palliative Care für andere Berufsgruppen oder eine mehrjährige Erfahrung in der Palliativversorgung verfügen.

c) Eine tägliche telefonische Rund-um-die-Uhr-Erreichbarkeit für die Patientin/den Patienten, deren Angehörigen und die an der Versorgung Beteiligten ist durch das Palliative-Care-Team sicherzustellen. Die ständige Verfügbarkeit mindestens einer qualifizierten Palliativ-Ärztin/eines qualifizierten Palliativ-Arztes und einer Pflegefachkraft mit entsprechender Qualifikation gemäß Abs. 2 a) ist zu garantieren. Diese Verfügbarkeit schließt notwendige Hausbesuche ein.

**3. Sachliche Voraussetzungen**

a) Das Palliative-Care-Team hat als Mindestanforderung an die sächliche Ausstattung Folgendes vorzuhalten bzw. sicherzustellen:

- das Palliative-Care-Team hat unter der Voraussetzung, dass eine entsprechende Patienteninformation und Entbindung von der Schweigepflicht (Anlage 3) vorliegt, eine Patientendokumentation (Anlage 7) zu erstellen, die den Anforderungen der Gemeinsamen Empfehlungen der Spitzenverbände der Krankenkassen nach § 132 d Abs. 2 SGB V für die SAPV vom 23.06.2008, Punkt 6.2. entspricht
- Arzneimittel (inkl. BtM) für die Notfall/Krisenintervention
- Arzt-/Pflegekoffer/Bereitschaftstasche (Berücksichtigung der Kompatibilität der Verbrauchsmaterialien zu Medizinprodukten unterschiedlicher Hersteller, z. B. bei Portsystemen oder Infusionspumpen)
- eine geeignete administrative Infrastruktur, z. B. Büro, Kommunikationstechnik.

b) Das Palliative-Care-Team muss über eine eigenständige Adresse und geeignete Räumlichkeiten für

- die Beratung von Patientinnen/Patienten und Angehörigen
- Teamsitzungen und Besprechungen
- die Lagerhaltung von eigenen Medikamenten für Notfall- /Krisenintervention und Hilfsmitteln

verfügen. Sofern eine Aufbewahrung von Medikamenten erfolgt, die unter das Betäubungsmittelgesetz (BtM-G) fallen, ist ein BtM-Schrank (§ 15 BtM-G) erforderlich.

## 4. Nachweis der Voraussetzungen

Zum Nachweis der organisatorischen und personellen Voraussetzungen hat das Palliative-Care-Team den Krankenkassen zur Überprüfung, ob die Voraussetzungen zum Abschluss dieses Vertrages erfüllt werden, mindestens die nachfolgenden Unterlagen vor Vertragsabschluss einzureichen:

a) Nachweis der aktuell beschäftigten Mitarbeiter sowie die Qualifikationsnachweise des eingesetzten Personals inkl. Nachweis zur Dokumentation der Unterschriften/Handzeichen

b) Nachweis eines Institutionskennzeichens (IK) für das Palliative-Care-Team

c) Nachweis der Berufserfahrung für die Ärztin/den Arzt und die leitende und stellvertretende leitende Pflegefachkraft

d) bei der Rechtsform

   (1) einer Gesellschaft bürgerlichen Rechts (GbR) einen Auszug aus dem Gesellschaftsvertrag in Kopie mit Angabe der Gesellschafter, Geschäftsführung, Unternehmenszweck

   (2) einer Gesellschaft mit beschränkter Haftung (GmbH) einen Auszug aus dem notariell beurkundeten Gesellschaftsvertrag in Kopie mit Angabe der Gesellschafter, Geschäftsführung, Unternehmenszweck sowie einen beglaubigten Auszug aus dem Handelsregister des zuständigen Amtsgerichts

   (3) eines eingetragenen Vereins (e. V.) einen Auszug aus der Vereinssatzung in Kopie mit Angabe der Vorstandsmitglieder, Geschäftsführung, Vereinszweck sowie einen beglaubigten Auszug aus dem Vereinsregister

   (4) für andere Gesellschaftsformen und Körperschaften, Anstalten und Stiftungen gelten die Nachweispflichten entsprechend

e) ggf. bestehende Kooperationsverträge

## § 8
## Weitere Koordinationsaufgaben und Anforderungen

(1) An das Palliative-Care-Team werden folgende weitere Koordinationsaufgaben und Anforderungen gestellt:

- Klare Benennung und Zuordnung einer Pflegedienstleitung und deren Stellvertretung
- Klare Benennung und Zuordnung der Koordinationsfunktionen
- Zentrale Anlaufstelle
- Sicherstellung eines örtlichen abgestimmten Notfallplanes
- Koordinierung einer Vertretungsregelung und einer Rund-um-die-Uhr-Bereitschaft
- Organisation der Initial- und Folgebesuche gemäß Abs. 2 Fallbesprechungen nach Abs. 2 sowie der Qualitätssicherungsmaßnahmen nach § 9
- Das Palliative-Care-Team ist zudem verpflichtet, eine additiv unterstützende Teilversorgung in Form von ärztlicher und pflegerischer Leistung zu erbringen, und zwar in folgen Fällen:
  - Sofern eine Vertragsärztin/ein Vertragsarzt Leistungen der APV (allgemeine Palliativversorgung) erbringt und additiv unterstützende Hilfe beim Palliative-Care-Team anfordert
  - Sofern eine Ärztin/ein Arzt, die/der im stationären Hospizdienst tätig ist, beim Palliative-Care-Team additiv unterstützende Hilfe angefordert/verordnet, ist diese in Form von ärztlicher Leistung im Rahmen dieses Vertrages zu erbringen
  - Das Palliative-Care-Team ist verpflichtet, eine additiv unterstützende Teilversorgung zu erbringen, sofern Ärzte, die in stationären Pflegeeinrichtungen tätig sind, solche Leistungen beim Palliative-Care-Team anfordern/verordnen.

(2) Im Rahmen des gemeinsamen Initialbesuches durch das Palliative-Care-Team (qualifizierte Palliativ-Ärztin/qualifizierter Palliativ-Arzt und Pflegefachkraft) bei der Patientin/beim Patienten erfolgt die integrative Abstimmung der notwendigen Maßnahmen und des Behandlungs- und Therapieplanes. Gleichzeitig wird ein Zeitpunkt (innerhalb von 14 Tagen) festgelegt, an dem eine erneute gemeinsame Fallbesprechung erfolgt, um ggf. die notwendigen Maßnahmen sowie den Behandlungs- und Therapieplan anzupassen.

Die behandelnde Hausärztin/der behandelnde Hausarzt sollte beim Initialbesuch sowie bei der Fallbesprechung mit eingebunden werden.

(3) Die qualifizierte Palliativärztin/der qualifizierte Palliativ-Arzt des Palliative-Care-Teams übernimmt insbesondere folgende Aufgaben:

- Durchführung der beim Initialbesuch abgestimmten ärztlichen Maßnahmen
- Einstellung und/oder Beratung zur Schmerztherapie und Symptomkontrolle
- Verordnung von Medikamentenapplikationen über Pumpensysteme unter besonderer Berücksichtigung des Wirtschaftlichkeitsgebotes
- Beratung zur künstlichen Ernährung und Flüssigkeitsgabe in der letzten Lebensphase
- Beurteilung und Einleitung einer palliativen Wundbehandlung
- Palliativmedizinische Betreuung und Beratung von Patientinnen/Patienten und deren Angehörigen
- Unterstützung in der Aufklärung der Patientin/des Patienten, der Vermittlung „schlechter Nachrichten“ und Information über die Änderung des Therapiezieles
- bei Bedarf Beratung bei der Erstellung einer Patientenverfügung
- Beratung bei ethischen Konflikten (ethisches Fallgespräch)

(4) Für das pflegerische Handeln ist der in Anlage 8 geregelte Pflegeprozess zu beachten.

## § 9
## Qualitätssicherung und Dokumentation

(1) Das Palliative-Care-Team ist verpflichtet, ein internes Qualitätsmanagement durchzuführen. Es nimmt regelmäßig an palliativmedizinischen/-pflegerischen Fortbildungen teil und führt möglichst halbjährlich multidisziplinäre Qualitätszirkel durch, an denen auch die übrigen in der Versorgung Tätigen teilnehmen. Das Palliative-Care-Team soll sich außerdem an Maßnahmen der externen Qualitätssicherung beteiligen. Ergänzend gelten die Regelungen des § 15.

(2) Ein geeignetes Dokumentationssystem, aus dem zu jeder Zeit die notwendigen Informationen über Versicherte und die erbrachten Leistungen hervorgehen, ist sachgerecht und kontinuierlich zu führen.

Das Dokumentationssystem muss patientenbezogene Daten und – soweit vorhanden – allgemein anerkannte Indikatoren für eine externe Qualitätssicherung enthalten und eine bundesweite Evaluation ermöglichen. Es soll kompatibel zu den bestehenden Dokumentationssystemen der an der Versorgung beteiligten Leistungserbringer sein. Der Gemeinsame Datensatz zur SAPV-Dokumentation der Deutschen Gesellschaft für Palliativmedizin e. V. und des Deutschen Hospiz- und PalliativVerbandes e. V. gemäß Anlage 7 ist einzusetzen.

(3) Die Teilnahme an Supervisionen ist zu ermöglichen. Regelmäßige multiprofessionelle Fallbesprechungen sind in überschaubaren Intervallen durchzuführen; die inhaltliche und zeitliche Organisation ist den Beteiligten entsprechend der Konzeption zu überlassen.

(4) Das Palliative-Care-Team erstellt jeweils nach gemeinsam erfolgtem Initialbesuch einen individuellen Behandlungsplan, der mit den übrigen an der Versorgung beteiligten Leistungserbringern abzustimmen ist.

(5) Vor jeder Krankenhauseinweisung hat zwingend eine Begutachtung der Patientin/des Patienten durch die qualifizierte Palliativ-Ärztin/den qualifizierten Palliativ-Arzt des Palliative-Care-Teams zu erfolgen. Grds. gilt dies auch für notfallmedizinische Einweisungen. Das Wohl und der Wille der Patientin/des Patienten ist hierbei zu berücksichtigen und steht daher im Vordergrund.

(6) Das Palliative-Care-Team hat die Leistungen sorgfältig und einwandfrei auszuführen. Qualität und Wirksamkeit haben dem allgemein anerkannten Stand der medizinischen und pflegerischen Erkenntnisse zu entsprechen sowie den medizinischen Fortschritt zu berücksichtigen und alle Expertenstandards (vgl. § 113 a SGB XI) anzuwenden.

(7) Die gemeinsamen Grundsätze und Maßstäbe zur Qualität und Qualitätssicherung einschließlich des Verfahrens zur Durchführung von Qualitätsprüfungen nach §§ 112 ff SGB XI in der ambulanten Pflege sind für den pflegerischen Bereich in der jeweils gültigen Fassung Bestandteil dieses Vertrages, soweit dieser Vertrag nichts anderes vorsieht.

(8) Grundlage für die Feststellung von Qualitätsmängeln ist der Prüfbericht des Medizinischen Dienstes der Krankenversicherung nach § 115 SGB XI sowie die Stellungnahme des Leistungserbringers. Der Nachweis von Qualitätsmängeln kann auch durch andere geeignete Sachverständigengutachten geführt werden.

## § 10
## Anzeigepflichten/Mitteilungspflichten

(1) Das Palliative-Care-Team ist verpflichtet, den Krankenkassen sowie der KV alle wesentlichen Umstände innerhalb von vier Wochen schriftlich mitzuteilen, die die vertragsgemäße Durchführung dieses Vertrages sowie dessen Fortbestand berühren. Anzuzeigen sind hiernach insbesondere:

a) das Ausscheiden einer qualifizierten Ärztin/eines qualifizierten Arztes oder einer qualifizierten Pflegefachkraft

b) Änderungen der betrieblichen/organisatorischen Gegebenheiten, die im Zusammenhang mit dem Versorgungskonzept stehen

c) der Abschluss, die Kündigung bzw. Änderungen von Kooperationsvereinbarungen

d) jede Änderung der Trägerschaft/Betriebsinhaberschaft sowie der Rechtsform des Palliative-Care-Teams

e) den Umstand, dass Antrag auf Eröffnung eines Insolvenzverfahrens gegen das Palliative-Care-Team oder gegen einen Leistungserbringer gestellt wurde

f) eingeleitete strafrechtliche Ermittlungsverfahren gegen einen Palliative-Care-Team-Partner oder das Palliative-Care-Team, soweit sie im Zusammenhang mit der Vertragserfüllung stehen

(2) Die Krankenkasse ist berechtigt, zum Nachweis der organisatorischen und/oder personellen Voraussetzungen weitere Unterlagen zu verlangen.

## § 11
## Verordnungen im Rahmen der SAPV

(1) Soweit Verordnungen von Arznei-, Heil- oder Hilfsmitteln für Versicherte medizinisch notwendig sind, werden diese von der Palliativ-Ärztin/dem Palliativ-Arzt unter Verwendung der für die vertragsärztliche Versorgung geltenden Muster (Muster 13, 14, 16 und 18) auf den Namen der Versicherten/des Versicherten verordnet. Die Verwendung dieser Vordrucke ist im Rahmen der SAPV nur zulässig, solange ein gültiger Vertrag nach § 132 d Abs. 1 SGB V mit dem Palliative-Care-Team der verordnenden Palliativ-Ärztin/des verordnenden Palliativ-Arztes besteht. Die Bereitstellung und die Kosten der Vordrucke tragen die Krankenkassen. Die Verordnungen sind auf Basis der geltenden Richtlinien des Gemeinsamen Bundesausschusses auszuführen. Dabei ist das Wirtschaftlichkeitsgebot zu beachten.

(2) Das Palliative-Care-Team erhält von der KV auf Antrag eine Betriebsstättennummer, die neben der Pseudo-Arzt-Nummer 999999900 auf den zu verwendenden Vordrucken einzutragen ist, unabhängig davon, ob die verordnende Ärztin/der verordnende Arzt eine lebenslange Arztnummer besitzt oder nicht. Die Betriebsstättennummer ist neunstellig. Die Betriebsstättennummer und die Pseudo-Arztnummer dürfen nur für Verordnungen sowie für die Abrechnung gegenüber der KV im Rahmen der SAPV verwendet werden und verlieren ihre Gültigkeit mit Beendigung dieses Vertrages. Die KV stellt bei der Vergabe der Betriebsstättennummer sicher, dass eine Zuordnung zur SAPV gewährleistet ist. Die Betriebsstättennummer des Palliative-Care-Teams ist den Krankenkassen mitzuteilen.

(3) Für die im Rahmen der SAPV verordneten Arznei-, Heil- oder Hilfsmittel gelten die nach §§ 300, 302 SGB V vereinbarten Abrechnungsverfahren entsprechend. Bei der zeitgleichen Verordnung von Arznei- und Hilfsmitteln sind jeweils getrennte Vordrucke nach Muster 16 zu verwenden. Auch auf Betäubungsmittel-Rezepten sind die SAPV-Betriebsstättennummer sowie die Pseudo-Arztnummer anzugeben. Solange das BtM-Muster noch nicht angepasst ist, ist die Betriebsstättennummer in das Feld „Vertragsarztnummer“ und die Pseudo-Arztnummer in das Feld „VK gültig bis“ einzutragen.

(4) Die Vertragspartner sind sich einig, dass die in § 11 Abs. 1 bis 3 geregelten Vorgaben für Verordnungen nur solange gelten, bis zwischen der KBV und dem GKV-Spitzenverband K. d. ö. R. eine abschließende Regelung vereinbart wurde, wie Verordnungen im Rahmen der SAPV auszustellen sind; es sei denn, Krankenkassen und KV sind sich einig, dass das bisherige Vergabeverfahren beibehalten werden soll. Die Vertragspartner wollen mit diesen vorläufigen Regelungen sicherstellen, dass der Anspruch der Versicherten mit Leistungen der SAPV mit Inkrafttreten dieses Vertrages erfüllt werden kann.

(5) Die Vertragsparteien vereinbaren, dass die Wirtschaftlichkeit der Verordnungen des Palliative-Care-Teams in entsprechender Anwendung der nach § 106a, 106 Abs. 2 und 3 geltenden Regelungen von den Prüfungseinrichtungen (Prüfungsstelle, Beschwerdeausschuss) geprüft werden. Die Prüfvereinbarung der KV und der Krankenkassen(verbände) - derzeit veröffentlicht in Rheinisches Ärzteblatt 12/2007 - in ihrer jeweils gültigen Fassung ist analog anzuwenden. Das Nähere regeln KV und die Krankenkassen in einer gesonderten Vereinbarung, die für das Palliative-Care-Team verbindlich ist.

## § 12
## Verordnung/Genehmigung der SAPV

(1) Die Kostenübernahme ist vor Beginn der SAPV bei der zuständigen Krankenkasse mit dem hierfür vereinbarten Vordruck zu beantragen; soweit dieser Vordruck noch nicht vorliegt, auf der vertragsärztlichen Verordnung nach Vordruckmuster 12. Die Verordnung ist vorab von der Versicherten/vom Versicherten und dem Palliative-Care-Team auszufüllen. Das Palliative-Care-Team hat dabei detailliert die geplanten SAPV-Leistungen sowie die Häufigkeit und Dauer einzutragen. Über die SAPV-Leistungen und ihre Dauer entscheidet die Krankenkasse unter Berücksichtigung der vertragsärztlichen Verordnung. Den jeweils aktuellen Schnellbegutachtungsbogen nach Anlage 9 und die vertragsärztliche Verordnung senden das Palliative-Care-Team an die zuständige Krankenkasse.

(2) Soweit die zuständige Krankenkasse eine Begrenzung/Einschränkung der Leistungsgewährung vorgibt, ist dies vom Palliative-Care-Team zu beachten. Ändern sich - ohne Auswirkung auf die Einsatzfrequenz - für den von der zuständigen Krankenkasse bereits genehmigten Zeitraum die SAPV-Leistungen bzw. kommen Leistungen hinzu, ist für den bereits von der zuständigen Krankenkasse genehmigten Zeitraum keine neue vertragsärztliche Verordnung erforderlich. Das Palliative-Care-Team hat in diesem Fall jedoch nachweislich sicherzustellen, dass es diese geplante geänderte ärztliche Anweisung erhalten hat und umsetzt.

(3) Hält eine Krankenhausärztin/ein Krankenhausarzt die Entlassung einer Patientin/eines Patienten für möglich und ist aus ihrer/seiner Sicht SAPV erforderlich, kann die Krankenhausärztin/der Krankenhausarzt die Verordnung ausstellen, längstens jedoch für 7 Tage. Sollte die SAPV-Leistung für einen längeren Zeitraum als 7 Tage erforderlich sein, ist rechtzeitig vor Ablauf der 7 Tage mittels der vertragsärztlichen Verordnung ein Verlängerungsantrag bei der zuständigen Krankenkasse zu stellen.

(4) Über Art, Umfang und Dauer der Leistung entscheidet die zuständige Krankenkasse unter Berücksichtigung der vertragsärztlichen Verordnung. Art und Inhalt der Leistungen ergeben sich aus diesem Vertrag sowie der Vergütungsvereinbarung nach Anlage 4. Die zuständige Krankenkasse entscheidet unverzüglich über den Leistungsantrag und informiert hierüber das Palliative-Care-Team. Soweit die vertraglich verordneten Leistungen nicht oder nur zum Teil übernommen werden, informiert die zuständige Krankenkasse zusätzlich die Versicherte/den Versicherten und die Ärztin/den Arzt.

(5) Bei Annahme einer vertragsärztlichen Verordnung über SAPV-Leistungen ist die Gesamtheit der von der zuständigen Krankenkasse genehmigten Leistungen zu erbringen.

(6) Die vertragsärztliche Verordnung ist nicht übertragbar; sie gilt nur für die Person, für die sie ausgestellt ist. Änderungen und Ergänzungen einer vertragsärztlichen Verordnung bedürfen der Unterschrift der verordnenden Ärztin/des verordnenden Arztes.

(7) Kann die Durchführung der SAPV-Leistungen nicht mehr erfolgen, weil z. B. die Versicherte/der Versicherte in ein Krankenhaus eingewiesen wurde, informiert das Palliative-Care-Team unverzüglich die zuständige Krankenkasse und die behandelnde Ärztin/den behandelnden Arzt. Gleiches gilt, wenn nach initialer Aufnahme in die SAPV im Rahmen eines anlassbezogenen (z. B. Befundverbesserung) oder regelmäßig durchgeführten Re-Assessment (14-tägig) festgestellt wird, dass die besonderen Mittel der SAPV nicht mehr notwendig sind und eine Regelversorgung ausreicht.

(8) Das Palliative-Care-Team prüft im Rahmen des Eingangsassessments innerhalb von 3 Arbeitstagen nach Erhalt der Verordnung die Notwendigkeit und Eignung der besonderen Mittel der SAPV. Sofern für eine bedarfsgerechte Versorgung die Mittel der Regelversorgung ausreichen, ist die zuständige Krankenkasse

hierüber unverzüglich zu informieren. Neben der zuständigen Krankenkasse sind auch die Versicherte/der Versicherte und der Erst-Verordner über die Ergebnisse des Assessments zu informieren.

## § 13
## Vorläufige Kostenzusage

(1) Die zuständige Krankenkasse übernimmt bei Erstverordnung vom ärztlich festgelegten Leistungsbeginn an bis zur Entscheidung über die weitere Leistungserbringung die Kosten für die verordneten und vom Palliative-Care-Team erbrachten SAPV-Leistungen entsprechend der Vergütung gemäß dieses Vertrages und wenn die entsprechend der SAPV-Richtlinie ausgestellte Verordnung spätestens am dritten, der Ausstellung folgenden Arbeitstag der zuständigen Krankenkasse vorliegt und die Leistungen vertragsmäßig erbracht und dokumentiert wurden.

(2) Soweit die verordneten Leistungen nicht oder nur zum Teil übernommen werden, informiert die zuständige Krankenkasse die Versicherte/den Versicherten oder eine ihm nahe stehende Person, das Palliative-Care-Team und die verordnende Ärztin/den verordnenden Arzt.

## § 14
## Grundsätze der Leistungserbringung

(1) Das Palliative-Care-Team hat darauf zu achten, dass die Leistungen wirksam und wirtschaftlich erbracht und nur im notwendigen Umfang in Anspruch genommen werden (§ 2 Abs. 4 SGB V).

(2) Die Leistungen müssen ausreichend, zweckmäßig und wirtschaftlich sein; sie dürfen das Maß des Notwendigen nicht überschreiten. Leistungen, die nicht notwendig oder unwirtschaftlich sind, können Versicherte nicht beanspruchen, darf das Palliative-Care-Team nicht bewirken und die zuständige Krankenkasse nicht bewilligen (§ 12 Abs. 1 SGB V).

(3) Das Palliative-Care-Team gewährleistet, dass die Versicherten aller vertragsschließenden Krankenkassen nach gleichen Grundsätzen behandelt werden.

(4) Zur Optimierung der Versorgung mit Leistungen, insbesondere um einen reibungslosen und unverzüglichen Übergang von der stationären Krankenhausbehandlung zur ambulanten palliativmedizinischen und palliativpflegerischen Versorgung zu gewährleisten, hat das Palliative-Care-Team mit

a) Vertragsärzten

b) ambulanten Hospizdiensten

c) stationären Hospizen

d) Vertragskrankenhäusern

e) Rehabilitationseinrichtungen

f) und sonstigen innerhalb des Versorgungsspektrums Beteiligten

integrativ zusammenzuarbeiten.

(5) Das Palliative-Care-Team gewährleistet in seinem Versorgungsgebiet (vgl. § 6 Abs. 3 und § 2 Abs. 1), dass zur Sicherstellung einer fach- und sachgerechten spezialisierten ambulanten palliativmedizinischen und palliativpflegerischen Versorgung nach diesem Vertrag fachlich qualifiziertes Personal eingesetzt wird und eine fach- und sachgerechte ambulante palliativmedizinische und palliativpflegerische Versorgung erfolgt.

(6) Das Palliative-Care-Team trägt die fachliche Verantwortung für das eingesetzte Personal. Über den Einsatz des Personals im Einzelfall entscheidet das Palliative-Care-Team unter Beachtung der medizinischen Notwendigkeit sowie Art und Schwere der Erkrankung der Versicherten/des Versicherten im Rahmen des Wirtschaftlichkeitsgebotes. Die Erbringung der vertraglichen Leistungen hat mit möglichst geringem Wechsel des Personals zu erfolgen.

## § 15
## Fort- und Weiterbildung

(1) Das Palliative-Care-Team hat auf der Grundlage des vorgelegten Fort- und Weiterbildungskonzeptes die Mitarbeiterinnen und Mitarbeiter fortzubilden. Die Inhalte der Fortbildungen sind so auszurichten, dass die Mitarbeiterinnen/die Mitarbeiter auf dem aktuellen Stand der allgemein anerkannten ärztlich-medizinischen und pflegewissenschaftlichen Erkenntnisse sind. Leitungskräfte (Pflegedienstleitung, Stellvertretung, Teamleitung, Qualitätsbeauftragter etc.) des

Palliative-Care-Teams sind verpflichtet, externe fachbezogene Fortbildungen oder Inhouse-Schulungen von kalenderjährlich insgesamt 28 Stunden nachzuweisen. Hierbei ist darauf zu achten, dass diese Fortbildungen die Bereiche Dokumentation, Management und Pflege abdecken. Für die übrigen Mitarbeiterinnen/Mitarbeiter sind regelmäßige Fortbildungen, die sich an den Inhalten des Satzes 1 orientieren sollten, durchzuführen.

Als Nachweis dienen die entsprechenden Zertifikate bzw. Kopien der Veranstaltungsprotokolle (Teilnehmerliste).

Der Nachweis der kalenderjährlich durchgeführten Fortbildung ist bis zum 31.03. des Folgejahres den Krankenkassen vorzulegen.

(2) Werden Fortbildungen, wie in Absatz 1 beschrieben, nicht fristgerecht nachgewiesen, werden vom Folgemonat an (April) die Vergütungssätze nach Maßgabe des § 16 Abs. 3 gekürzt. Dem Palliative-Care-Team ist eine Frist zu setzen, innerhalb derer es die Fortbildung nachholen kann. Als Frist hierfür ist längstens ein Zeitraum von sechs Monaten möglich.

(3) Wird die Fortbildung vor Beendigung der Frist nachgewiesen, entfällt vom nächsten Monatsersten an die Kürzung der Vergütung.

## § 16
## Vergütung

(1) Die ärztlichen und pflegerischen bzw. kombinierten Leistungen im Rahmen der SAPV werden als Komplexpauschalen vergütet. Die Höhe der Vergütung richtet sich nach den in Anlage 4 aufgeführten Pauschalen. Mit dieser Vergütung sind alle vertraglichen Haupt- und Nebenpflichten abgegolten.

(2) Der Vergütungsanspruch besteht nur für entsprechend den Vorgaben dieses Vertrages tatsächlich erbrachte und dokumentierte Vertragsleistungen. Für nicht vertragsmäßig erbrachte und auch für nicht dokumentierte Vertragsleistungen besteht weder ein Vergütungsanspruch gegenüber dem Kostenträger noch gegenüber der Versicherten/dem Versicherten.

(3) Liegt der zuständigen Krankenkasse innerhalb von 7 Arbeitstagen ab Verordnungsbeginn zusätzlich zur Erstverordnung und dem Schnellbegutachtungsbogen kein Ergebnis über das SAPV-Eingangsassessment des Palliative-Care-Teams vor, endet der Vergütungsanspruch mit Ablauf dieses Tages. Der Leis-

tungsanspruch des Versicherten bleibt hiervon unberührt. Die zuständige Krankenkasse informiert das Palliative-Care-Team.

(4) Kommt das Palliative-Care-Team seiner Verpflichtung zum Nachweis der Fortbildung (§ 15) nicht nach, erfolgen Vergütungsabschläge für die nach diesem Vertrag zu vergütenden Leistungen in Höhe von 10 v. H. für die ersten drei Monate, danach in Höhe von 25 v. H.

(5) Mit den für die Vertragsleistungen gemäß Anlage 4 des Vertrages vereinbarten Vergütungssätzen sind zugleich sämtliche Aufwendungen des Palliative-Care-Teams abgegolten, die im unmittelbaren und mittelbaren Zusammenhang mit der jeweils vergüteten Vertragsleistung stehen.

(6) Dem Palliative-Care-Team sowie dessen Personal ist es untersagt, für Vertragsleistungen Zahlungen von der Versicherten/dem Versicherten oder einem sonstigen Dritten zu fordern oder anzunehmen.

## § 17
## Abrechnung

Die Abrechnungsvoraussetzungen sind in Anlage 4 geregelt.

## § 18
## Leistungsnachweis

(1) Jede vom Palliative-Care-Team erbrachte palliativ-ärztliche oder palliativ-pflegerische Leistung ist unmittelbar nach ihrer Durchführung im Leistungsnachweis durch die Palliativ-Ärztin/den Palliativ-Arzt bzw. die ausführende Pflegefachkraft mit entsprechender Qualifikation gemäß § 7 Abs. 2 a) einzutragen und durch Handzeichen zu bestätigen. Maschinelle Eintragungen im Leistungsnachweis sowie Vordatierungen oder Globalbestätigungen sind unzulässig. Gleiches gilt für Eintragungen im Leistungsnachweis vor Erbringung der Leistung. Eine Durchschrift des Leistungsnachweises hat auf Wunsch bei der Patientin/beim Patienten zu verbleiben. Der Leistungsnachweis hat die folgende Mindestinhalte aufzuweisen:

a) Krankenversichertennummer

b) Name und Vorname der Patientin/des Patienten

c) Geburtsdatum der Patientin/des Patienten

d) Tag der Leistungserbringung

e) Uhrzeit (Beginn und Ende) der Leistungserbringung

f) Art und Inhalt der Leistung

g) einmal monatlich eine Bestätigung durch Unterschrift eines Verantwortlichen des Palliative-Care-Teams,

h) nach erfolgter Leistungserbringung einmal monatlich Datum und Unterschrift der Patientin/des Patienten, des gesetzlichen Vertreters oder Bevollmächtigten sowie

i) Institutionskennzeichen, Name und Anschrift des Palliative-Care-Teams

(2) Als Muster dient der Leistungsnachweis entsprechend Anlage 10.

## § 19
## Einflussnahme-, Werbungs- und Vermittlungsverbot

(1) Eine Beeinflussung der verordnenden Ärztin/des verordnenden Arztes mit dem Ziel, diesen zu unwirtschaftlichen Verordnungen zu veranlassen, ist unzulässig.

(2) Die Gewährung oder Annahme von Zuwendungen gleich welcher Art (z. B. Prämien, Provisionen o. Ä.) für die Zuweisung von Versicherten an Palliative-Care-Teams ist unzulässig.

(3) Soweit Versicherte gezielt zur Inanspruchnahme dieser Leistungen aufgefordert werden, ist Werbung für die im Rahmen dieses Vertrages zur Verfügung stehenden Leistungen nicht zulässig. Das Palliative-Care-Team ist jedoch berechtigt, den Versicherten an die zuständige Krankenkasse zwecks Beratung über die Möglichkeit der Inanspruchnahme von Leistungen nach diesem Vertrag zu verweisen.

(4) Die Vermittlung an Dritte gegen Entgelt oder zur Erlangung geldwerter Vorteile ist nicht zulässig. Vermittlung im Sinne dieser Bestimmung ist auch die Weitergabe von in eigenem Namen angenommenen Pflegeaufträgen an Dritte (z. B. freie Mitarbeiter) gegen Kostenerstattung.

## § 20
## Zuzahlungen

(1) SAPV wird als Sachleistung zur Verfügung gestellt. Jegliche Zuzahlungen von der Versicherten/vom Versicherten im Rahmen der Vertragsleistungen dürfen weder gefordert noch angenommen werden.

(2) Zuzahlungen dürfen auch in dem Fall nicht verlangt werden, wenn die Verordnung bei der zuständigen Krankenkasse verspätet eingereicht wird, da Kosten erst ab Eingang der Verordnung bei der zuständigen Krankenkasse gemäß § 13 dieses Vertrages übernommen werden.

(3) Verstöße berechtigen zu Maßnahmen gemäß § 23 dieses Vertrages.

## § 21
## Datenschutz, Schweigepflicht

(1) Das Palliative-Care-Team ist verpflichtet, die Bestimmungen über den Schutz der Sozialdaten (SGB X, 2. Kapitel) zu beachten, insbesondere personenbezogene Daten nur zur Erfüllung der sich aus diesem Vertrag ergebenden Aufgaben zu erheben, zu verarbeiten oder zu nutzen.

(2) Das Palliative-Care-Team unterliegt hinsichtlich der Person der Versicherten/des Versicherten und deren/dessen Krankheiten der Schweigepflicht. Ausgenommen hiervon sind Angaben gegenüber der leistungspflichtigen Krankenkasse und/oder dem Medizinischen Dienst der Krankenkassen, soweit sie zur Erfüllung der gesetzlichen Aufgaben der zuständigen Krankenkasse erforderlich sind.

(3) Das Palliative-Care-Team hat die im Team tätigen Mitarbeiterinnen/Mitarbeiter zur Beachtung der Datenschutzbestimmungen und der Schweigepflicht zu verpflichten. Die Verpflichtung ist zu dokumentieren.

## § 22
## Statistik

1) Das Palliative-Care-Team ist verpflichtet, an Auswertungen mitzuwirken. Hierzu übermittelt es auf Anfrage halbjährlich folgende Kennzahlen:

a) Fallzahlen (Anzahl der behandelten Patienten)

b) Diagnosen

c) Versorgungsbereich

d) Zahl der Vertragsärzte

e) Zahl der Kooperationspartner

f) Zahl an der Versorgung beteiligter Angehöriger

g) Zahl der Ehrenamtlichen

h) prozentualer Anteil von Patienten, bei denen Ehrenamtliche eingesetzt werden

i) Krankenhauseinweisungen

j) prognostizierte und tatsächliche Lebenserwartung

k) prozentualer Anteil von Patienten, die in der Häuslichkeit verstorben sind

l) durchschnittliche Betreuungsdauer, durchschnittliche Fahrzeit und durchschnittliche Einsatzzeit (gegliedert nach ärztlich delegierter Leistung)

m) Einstufungen der Patienten in Pflegestufen nach dem SGB XI

## § 23
## Vertrags-/Gesetzesverstöße

(1) Erfüllt das Palliative-Care-Team seine gesetzlichen und/oder vertraglichen Pflichten nicht in der gebotenen Weise, entscheiden die Krankenkassen gemeinsam oder einzeln über geeignete Maßnahmen. Als solche kommen in Betracht:

a) Verweis
b) Abmahnung
c) Vertragsstrafe
d) Außerordentliche Kündigung des Vertrages

(2) Die Vertragsstrafe wird durch die Krankenkassen gemeinsam oder einzeln festgesetzt, und zwar insbesondere bei wiederholt fehlerhaften Abrechnungen durch

das Palliative-Care-Team in Höhe von bis zu 15.000,00 EUR. Die vorstehende Vertragsstrafe gilt für jeden einzelnen Vertragsverstoß. Der Krankenkasse/den Krankenkassen bleibt es unbenommen, daneben einen weiteren Schadensersatz geltend zu machen bzw. die Kündigungsrechte gem. § 24 wahrzunehmen.

(3) Zur Aufklärung des Sachverhalts hat das Palliative-Care-Team den Kranken-kassen/ der Krankenkasse die erforderlichen Unterlagen zu übersenden bzw. die Einsichtnahme und ggf. Ablichtungen in seinen Geschäftsräumen zu gewähren.

(4) Verstöße gegen die gesetzlichen und/oder vertraglichen Bestimmungen sind dem Palliative-Care-Team schriftlich mitzuteilen. Ihm ist die Gelegenheit zu einer Stellungnahme innerhalb angemessener Frist zu geben. Die Vertragspartner bemühen sich, eventuelle Meinungsverschiedenheiten über Vertragsverstöße gemeinsam mit dem Ziel einer gütlichen Einigung zu klären.

(5) Unabhängig von den Maßnahmen nach Absatz 1 ist der durch die Vertragsverletzung verursachte Schaden durch das Palliative-Care-Team zu ersetzen.

## § 24
## Außerordentliche Kündigung

(1) Der Vertrag kann von den vertragsschließenden Krankenkassen gemeinsam oder einzeln gegenüber dem Palliative-Care-Team ohne Einhaltung einer Kündigungsfrist gekündigt werden, wenn ein Vertragspartner seine gesetzlichen und/oder vertraglichen Pflichten gegenüber den Versicherten oder der zuständigen Krankenkasse derart gröblich verletzt, dass ein Festhalten an diesem Vertrag nicht zumutbar ist. Eine gröbliche Pflichtverletzung in diesem Sinne liegt insbesondere vor bei:

- Nichterfüllung der organisatorischen, personellen oder sächlichen Voraussetzungen gemäß § 7 über einen Zeitraum von mehr als drei Monaten
- Leistungserbringung durch dafür fachlich nicht qualifiziertes Personal entgegen § 7
- Schwerwiegende pflegebedingte Qualitätsmängel, die medizinisch oder pflegerisch festgestellt werden
- Leistungserbringung durch freie Mitarbeiter entgegen § 19 Abs. 4
- Abrechnung von Leistungen entgegen § 16 Abs. 2
- Forderungen von Versicherten entgegen § 16 Abs. 5

- Verstoß gegen § 19
- fehlender Nachweis, dass die Fortbildung nicht innerhalb der in § 15 Abs. 2 geregelten Frist von 6 Monaten nachgeholt wurde

(2) Ein Recht zur außerordentlichen Kündigung besteht ferner bei Vertragsverstößen des Palliative-Care-Teams oder eines Leistungserbringers des Palliative-Care-Teams, die zur außerordentlichen Kündigung eines mit diesem Leistungserbringer bestehenden Versorgungsvertrages berechtigen, vgl. z. B. § 74 SGB XI.

(3) Vor Ausspruch der außerordentlichen Kündigung i. S. d. Absatz 1 teilt die zuständige Krankenkasse(n) dem Palliative-Care-Team ihre Kündigungsabsicht mit und gibt/geben dem Palliative-Care-Team Gelegenheit, binnen drei Wochen nach Zugang dieser Mitteilung schriftlich zu den erhobenen Vorwürfen Stellung zu nehmen. Entscheidend zur Fristwahrung ist der Zugang der Stellungnahme des Palliative-Care-Teams bei der/den zuständigen Krankenkasse(n).

(4) Die in Absatz 3 geregelte Verpflichtung besteht nicht, wenn das Wohl der Versicherten der zuständigen Krankenkasse(n) unverzügliches Handeln der zuständigen Krankenkasse(n) erfordert, insbesondere wenn die Versorgung der Versicherten mit Leistungen nach diesem Vertrag durch das Palliative-Care-Team nicht (mehr) sichergestellt ist und dadurch Leben oder Gesundheit von Versicherten gefährdet ist (Gefahr im Verzuge).

(5) Die Kündigung muss schriftlich erfolgen.

## § 25
## Inkrafttreten, Ordentliche Kündigung

(1) Dieser Vertrag tritt einschließlich seiner Anlagen am            in Kraft.

(2) Der Vertrag kann zum Ende eines Kalenderhalbjahres mit dreimonatiger Frist durch eingeschriebenen Brief von und gegenüber jedem Vertragspartner einzeln gekündigt werden. Die Kündigung von oder gegenüber einem Vertragspartner lässt die Wirksamkeit des Vertrages im Verhältnis zu den übrigen beteiligten Vertragspartnern unberührt.

(3) Abweichend von Abs. 2 gilt für die Vergütungsvereinbarung nach Anlage 4 die dort vorgesehene besondere Kündigungsregelung.

## § 26
## Änderungen und Ergänzungen

Änderungen oder Ergänzungen des Vertrages bedürfen der Schriftform.

## § 27
## Salvatorische Klausel

(1) Sollte eine Bestimmung des Vertrages unwirksam sein oder werden oder der Vertrag unvollständig sein, so wird der Vertrag in seinem übrigen Inhalt davon nicht berührt. Die unwirksame Bestimmung wird durch eine solche Bestimmung ersetzt, die dem Sinn und Zweck der unwirksamen Bestimmung in rechtswirksamer Weise und wirtschaftlich am nächsten kommt. Gleiches gilt für etwaige Vertragslücken.

(2) Im Übrigen gilt § 59 SGB X.

# Anlage 1

**Vertrag über die Erbringung**
**Spezialisierter ambulanter Palliativversorgung (SAPV) in Nordrhein**
**gemäß § 132 d SGB V i. V. m. § 37 b SGB V**

**„Strukturerhebungsbogen des Palliative-Care-Teams „Name““**

**Strukturerhebungsbogen des Palliative-Care-Teams „Name“**

| An die zuständige Krankenkasse |
|---|
| |

Sollten sich bezüglich der nachfolgenden Angaben zu 1 - 5 Änderungen ergeben, werden diese durch das Palliative-Care-Team unverzüglich den beteiligten Vertragspartnern schriftlich mitgeteilt.

**<u>Angaben zum Palliative-Care-Team</u>**

**1. Name des Palliative-Care-Teams**

______________________________________

**Straße**

______________________________________

**Postfach**

______________________________________

**PLZ/Ort**

______________________________________

**Rechtsform**

______________________________________

**Gesetzlicher Vertreter/Funktion**

______________________________________

**Namen der qualifizierten Palliativ-Ärzte**

______________________________________

______________________________________

______________________________________

______________________________________

______________________________________

**Name der verantwortlichen Pflegefachkraft/Pflegedienstleitung (PDL)**

______________________________________

**Name der stellvertretenden Pflegedienstleitung (PDL)**

______________________________________

**Telefon-Nr./Telefax**

______________________________________

**E-Mail**

______________________________________

**Internet**

______________________________________

**Institutionskennzeichen**

______________________________________

**Betriebsstättennummer**

______________________________________

**Straße**

______________________________________

**Postleitzahl, Ort**

______________________________________

**Telefon-Nr.**

______________________________________

**Telefax-Nr.**

**2. Qualifizierte Palliativ-Ärzte/Pflegedienstleitung/stellvertretende Pflegedienstleitung**

2.1 Bitte Namen der qualifizierten Palliativ-Ärzte angeben:

______________________________________

______________________________________

______________________________________

______________________________________

______________________________________

______________________________________

2.2 Bitte Namen und Qualifikation der verantwortlichen Pflegefachkraft (Pflegedienstleitung) angeben:

______________________________________

______________________________________

2.3 wie 2.2 (aber stellvertretende verantwortliche Pflegefachkraft [Pflegedienstleitung])

______________________________________

______________________________________

**3. Versorgungskonzept**

Das verbindliche, strukturierte und schriftlich beigefügte Versorgungskonzept des Palliative-Care-Teams enthält gemäß § 6 Abs. 3 mindestens folgende Punkte:

- Standort des Palliative-Care-Teams
- Versorgungsgebiet
- Versorgungsprozess
- Kooperationspartner
- Kooperationsvereinbarungen im Versorgungsgebiet; verpflichtend mit mindestens einem ambulanten Hospizdienst nach § 39 a Abs. 2 SGB V, Seelsorge und Sozialarbeit zur Sicherung der psychosozialen Unterstützung sowie mindestens einer Apotheke, die die Voraussetzungen gem. § 7 Abs. 1 b) erfüllt
- Mitarbeiterkapazität (ggf. Stellenplan)
- Personelle Zuständig- und Verantwortlichkeiten (u. a. Weisungsbefugnisse, Weisungsgebundenheit u. a.)
- Qualifikationsnachweise des Palliative-Care-Teams gem. § 7 Abs. 2
- Sächliche Ausstattung gem. § 7 Abs. 3
- Qualitätssicherungsmaßnahmen gemäß § 9
- Konzept zur Sicherstellung der reibungslosen Versorgung mit Arznei-, Heil-, und Hilfsmitteln
- Konzept zur Sicherstellung der 24-Stunden-Rufbereitschaft
- Fallzahleinschätzung
- Fort- und Weiterbildungskonzept

**Bitte Nachweise wie im § 7 des Vertrages näher geregelt beifügen.**

# Anlage 2

**Vertrag über die Erbringung**

**Spezialisierter ambulanter Palliativversorgung (SAPV) in Nordrhein**

**gemäß § 132 d SGB V i. V. m. § 37 b SGB V**

**„Nachweis und Handzeichenliste“**

Nachweis der zur Zeit beschäftigten/tätigen qualifizierten Palliativ-Ärzte und Pflegefachkräfte
- Dokumentation der Unterschriften/Handzeichen -

**Name und Anschrift des Palliative-Care-Teams**

____________________________________________________________

| Name, Vorname | Anschrift (PLZ, Ort, Straße) freiwillige Angabe | wöchentl. Arbeitszeit (in Std.) | Beschäfti-gungsbeginn | Unterschrift des Mitarbeiters | Hand-zeichen |
|---|---|---|---|---|---|

Qualifizierte Palliativ-Ärztin/qualifizierter Palliativ-Arzt:

| | | | | | |
|---|---|---|---|---|---|
| | | | | | |
| | | | | | |
| | | | | | |
| | | | | | |
| | | | | | |

Pflegedienstleitung **seit**:

| | | | | | |
|---|---|---|---|---|---|
| | | | | | |

stellvertretende Pflegedienstleitung **seit**:

| | | | | | |
|---|---|---|---|---|---|
| | | | | | |

weitere Pflegefachkräfte, die palliativpflegerische Leistungen erbringen

| | | | | | |
|---|---|---|---|---|---|
| | | | | | |
| | | | | | |
| | | | | | |
| | | | | | |
| | | | | | |
| | | | | | |
| | | | | | |

Datum

Stempel und Unterschrift des Palliative-Care-Teams

# Anlage 3

**Vertrag über die Erbringung**
**Spezialisierter ambulanter Palliativversorgung (SAPV) in Nordrhein**
**gemäß § 132 d SGB V i. V. m. § 37 b SGB V**

**„Patienteninformation und Entbindung von der Schweigepflicht“**

# Patienteninformation und Entbindung von der Schweigepflicht

## Spezialisierte ambulante Palliativversorgung

## gem. § 132 d SGB V i. V. m. § 37 b SGB V

| Krankenkasse bzw Kostenträger | | |
|---|---|---|
| Name Vorname des Versicherten | | geb. am |
| Kassen-Nr. | Versicherten-Nr | Status |
| Betriebsstätten-Nr. | Arzt-Nr | Datum |

1. Die behandelnde Ärztin/der behandelnde Arzt des Palliative-Care-Teams hat mich darüber aufgeklärt, dass sich mein Allgemeinzustand durch spezialisierte ambulante palliativmedizinische und palliativpflegerische Maßnahmen verbessern lässt. Ich wünsche, die mir verbleibende Zeit durch qualifiziertes medizinisches und pflegerisches Zusammenwirken des mich betreuenden Palliative-Care-Teams möglichst schmerzfrei im häuslichen Umfeld und im engen Kontakt zu Angehörigen und vertrauten Personen zu erleben.

2. Hiermit erkläre ich, dass ich über die Inhalte und den Verlauf der palliativmedizinischen und -pflegerischen Versorgung, die in dem Vertrag über die Erbringung spezialisierter ambulanter Palliativversorgung (SAPV) in Nordrhein gem. § 132 d SGB V i. V. m. § 37 b SGB V geregelt sind, umfassend informiert wurde und damit einverstanden bin, dass die notwendige medizinische und pflegerische Versorgung unter Einbindung meiner Haus- bzw. Fachärztin/meines Haus- bzw. Facharztes entsprechend durchgeführt wird.

3. Datenschutzhinweis (§ 67 a Abs. 3 SGB X). Damit wir Ihren Antrag auf SAPV-Leistungen bearbeiten können, ist Ihr Mitwirken nach § 60 SGB I erforderlich. Ihre Daten sind aufgrund § 37 b SGB V i. V. m. § 92 Abs. 1 SGB V zu erheben.

Ich wünsche die Behandlung nach dem mir vom Palliative-Care-Team umfassend erläuterten Versorgungskonzept.

Ich bin damit einverstanden,

- dass meine Daten von den Personen des Palliative-Care-Teams im Rahmen des oben beschriebenen und mir erläuterten Versorgungskonzepts erhoben, verarbeitet und genutzt werden und
- dass insoweit die an der Versorgung beteiligte Ärztin/beteiligter Arzt und deren Mitarbeiter/innen von der ärztlichen Schweigepflicht entbunden sind.

______________________________

Name, Vorname

______________________________

geb. am wohnhaft in

______________________________

Ort/Datum

______________________________

Unterschrift Patientin/Patient

______________________________

Unterschrift des Palliative-Care-Teams

**- Ausfertigung für die Patientin/den Patienten -**

# Patienteninformation und Entbindung von der Schweigepflicht

## Spezialisierte ambulante Palliativversorgung

## gem. § 132 d SGB V i. V. m. § 37 b SGB V

| Krankenkasse bzw Kostenträger | | |
|---|---|---|
| Name Vorname des Versicherten | | geb. am |
| Kassen-Nr. | Versicherten-Nr | Status |
| Betriebsstätten-Nr. | Arzt-Nr | Datum |

1. Die behandelnde Ärztin/der behandelnde Arzt des Palliative-Care-Teams hat mich darüber aufgeklärt, dass sich mein Allgemeinzustand durch spezialisierte ambulante palliativmedizinische und palliativpflegerische Maßnahmen verbessern lässt. Ich wünsche, die mir verbleibende Zeit durch qualifiziertes medizinisches und pflegerisches Zusammenwirken des mich betreuenden Palliative-Care-Teams möglichst schmerzfrei im häuslichen Umfeld und im engen Kontakt zu Angehörigen und vertrauten Personen zu erleben.

2. Hiermit erkläre ich, dass ich über die Inhalte und den Verlauf der palliativmedizinischen und -pflegerischen Versorgung, die in dem Vertrag über die Erbringung spezialisierter ambulanter Palliativversorgung (SAPV) in Nordrhein gem. § 132 d SGB V i. V. m. § 37 b SGB V geregelt sind, umfassend informiert wurde und damit einverstanden bin, dass die notwendige medizinische und pflegerische Versorgung unter Einbindung meiner Haus- bzw. Fachärztin/meines Haus- bzw. Facharztes entsprechend durchgeführt wird.

3. Datenschutzhinweis (§ 67 a Abs. 3 SGB X). Damit wir Ihren Antrag auf SAPV-Leistungen bearbeiten können, ist Ihr Mitwirken nach § 60 SGB I erforderlich. Ihre Daten sind aufgrund § 37 b SGB V i. V. m. § 92 Abs. 1 SGB V zu erheben.

Ich wünsche die Behandlung nach dem mir vom Palliative-Care-Team umfassend erläuterten Versorgungskonzept.

| Ich bin damit einverstanden,<br><br>- dass meine Daten von den Personen des Palliative-Care-Teams im Rahmen des oben beschriebenen und mir erläuterten Versorgungskonzepts erhoben, verarbeitet und genutzt werden und<br>- dass insoweit die an der Versorgung beteiligte Ärztin/beteiligter Arzt und deren Mitarbeiter/innen von der ärztlichen Schweigepflicht entbunden sind. |
|---|

Name, Vorname

geb. am wohnhaft in

Ort/Datum

Unterschrift Patientin/Patient

Unterschrift des Palliative-Care-Teams

**- Ausfertigung für das Palliative-Care-Team -**

# Patienteninformation und Entbindung von der Schweigepflicht

## Spezialisierte ambulante Palliativversorgung

## gem. § 132 d SGB V i. V. m. § 37 b SGB V

| Krankenkasse bzw Kostenträger | | |
|---|---|---|
| Name Vorname des Versicherten | | geb. am |
| Kassen-Nr. | Versicherten-Nr | Status |
| Betriebsstätten-Nr. | Arzt-Nr | Datum |

1. Die behandelnde Ärztin/der behandelnde Arzt des Palliative-Care-Teams hat mich darüber aufgeklärt, dass sich mein Allgemeinzustand durch spezialisierte ambulante palliativmedizinische und palliativpflegerische Maßnahmen verbessern lässt. Ich wünsche, die mir verbleibende Zeit durch qualifiziertes medizinisches und pflegerisches Zusammenwirken des mich betreuenden Palliative-Care-Teams möglichst schmerzfrei im häuslichen Umfeld und im engen Kontakt zu Angehörigen und vertrauten Personen zu erleben.

2. Hiermit erkläre ich, dass ich über die Inhalte und den Verlauf der palliativmedizinischen und -pflegerischen Versorgung, die in dem Vertrag über die Erbringung spezialisierter ambulanter Palliativversorgung (SAPV) in Nordrhein gem. § 132 d SGB V i. V. m. § 37 b SGB V geregelt sind, umfassend informiert wurde und damit einverstanden bin, dass die notwendige medizinische und pflegerische Versorgung unter Einbindung meiner Haus- bzw. Fachärztin/meines Haus- bzw. Facharztes entsprechend durchgeführt wird.

3. Datenschutzhinweis (§ 67 a Abs. 3 SGB X). Damit wir Ihren Antrag auf SAPV-Leistungen bearbeiten können, ist Ihr Mitwirken nach § 60 SGB I erforderlich. Ihre Daten sind aufgrund § 37 b SGB V i. V. m. § 92 Abs. 1 SGB V zu erheben.

Ich wünsche die Behandlung nach dem mir vom Palliative-Care-Team umfassend erläuterten Versorgungskonzept.

Ich bin damit einverstanden,

- dass meine Daten von den Personen des Palliative-Care-Teams im Rahmen des oben beschriebenen und mir erläuterten Versorgungskonzepts erhoben, verarbeitet und genutzt werden und
- dass insoweit die an der Versorgung beteiligte Ärztin/beteiligter Arzt und deren Mitarbeiter/innen von der ärztlichen Schweigepflicht entbunden sind.

______________________________

Name, Vorname

______________________________

geb. am wohnhaft in

__________________

Ort/Datum

______________________

Unterschrift Patientin/Patient

______________________________________________

Unterschrift des Palliative-Care-Teams

**- Ausfertigung für die zuständige Krankenkasse -**

# Anlage 4

## Vergütungsvereinbarung

**zum Vertrag über die Erbringung**
**Spezialisierter ambulanter Palliativversorgung (SAPV) in Nordrhein**
**gemäß § 132 d SGB V i. V. m. § 37 b SGB V**

**zwischen dem Palliative-Care-Team „Muster“, Rechtsform, Sitz, vertreten durch ...**

- nachfolgend Palliative-Care-Team genannt -

und

**der Kassenärztlichen Vereinigung Nordrhein**

- nachfolgend KV genannt -

und

**der AOK Rheinland/Hamburg - Die Gesundheitskasse**
**der BKK-Vertragsarbeitsgemeinschaft Nordrhein-Westfalen (BKK-VAG NRW)**
**der IKK Nordrhein**
**der Landwirtschaftlichen Krankenkasse Nordrhein-Westfalen**
**zugleich handelnd für die Krankenkasse für den Gartenbau**
**der Knappschaft**

sowie den Ersatzkassen

**BARMER Ersatzkasse**
**Techniker Krankenkasse**
**Deutsche Angestellten-Krankenkasse**

**Kaufmännische Krankenkasse - KKH - Allianz**

**Gmünder Ersatzkasse**

**HEK - Hanseatische Krankenkasse**

**Hamburg Münchener Krankenkasse**

**hkk**

gemeinsamer Bevollmächtigter mit Abschlussbefugnis

gemäß § 212 Abs. 5 S. 6 SGB V:

**Verband der Ersatzkassen e. V. (vdek),**

vertreten durch den Leiter der Landesvertretung Nordrhein-Westfalen

- nachfolgend Krankenkassen genannt -

wird folgender Vertrag geschlossen:

## § 1
## Vergütung

(1) Die Vergütung der nachstehend aufgeführten Leistungen des Palliative-Care-Teams erfolgt ausschließlich auf der Grundlage einer durch eine berechtigte Ärztin/einen berechtigten Arzt ausgestellten Erst- oder Folgeverordnung für das jeweilige Behandlungsquartal im Rahmen der SAPV gemäß der Richtlinie des Gemeinsamen Bundesausschusses nach § 37 b SGB V.

(2) Die palliativärztlichen und palliativpflegerischen Leistungen im Rahmen der SAPV werden nach Maßgabe der jeweils geltenden Abrechnungsregeln als Komplexpauschalen quartalsweise durch die KV vergütet. Mit den vereinbarten Komplexpauschalen sind sämtliche im Zusammenhang mit der Leistungserbringung und der Erfüllung der Pflichten nach dem „Vertrag über die Erbringung spezialisierter ambulanter Palliativversorgung (SAPV) in Nordrhein gem. § 132 d SGB V i.V.m. § 37 b SGB V" erforderlichen Aufwendungen und Verrichtungen abgegolten. Eine zeitgleiche Abrechnung von Gebührenordnungspositionen des Einheitlichen Bewertungsmaßstabes (EBM) durch das Palliative-Care-Team über die KV ist ausgeschlossen.

(3) Ärztinnen/Ärzte und andere Leistungserbringer des Palliative-Care-Teams können Leistungen der SAPV, die vom Palliative-Care-Team nach diesem Vertrag erbracht und abgerechnet werden, nur nach dieser Vergütungsvereinbarung und nicht zusätzlich als Leistungen nach weiteren im Bezirk der KV bestehenden Verträgen, die palliativmedizinische oder palliativpflegerische Versorgung zum Gegenstand haben, erbringen oder abrechnen. Leistungen der Palliativversorgung, die außerhalb dieses Vertrages zwischen der KV oder KV Consult und den Nordrheinischen Krankenkassen vereinbart wurden, können durch an den Verträgen teilnahmeberechtigte Ärztinnen/teilnahmeberechtigte Ärzte erbracht und abgerechnet werden. Dies gilt jedoch nicht, wenn die SAPV als Vollversorgung verordnet worden ist.

(4) Die nachfolgenden Leistungen kann das vertragsteilnehmende Palliative-Care-Team für die Versorgung der Patientin/des Patienten nach diesem Vertrag erbringen und quartalsweise über die KV abrechnen:

### I. Beratungsleistungen

Beratungsleistungen sind nur abrechnungsfähig, wenn sie ausdrücklich verordnet wurden. Als Maßnahme kann die Beratung der behandelnden Ärztin/des behandelnden Arztes, der behandelnden Pflegefachkraft und der Patientin/des Patienten/der Angehörigen durch das Palliative-Care-Team notwendig sein.

Im Einzelnen werden die Leistungen wie folgt vergütet; für jede erbrachte Leistung ist Punkt c) zu erfüllen:

a. Telefonat bzw. persönliches Gespräch mit Betroffenen
(Patientin/Patient oder deren Angehörige)

**Symbolnummer 92150** **20,00 EUR**

b. Telefonat bzw. persönliches Gespräch mit einem an der Versorgung teilnehmenden Leistungserbringer außerhalb des Palliative-Care-Teams

**Symbolnummer 92151** **20,00 EUR**

c. Dokumentation und Evaluation

Je Patientin/je Patient und je Einsatz können ärztlich sowie pflegerisch je 20,00 EUR vergütet werden, maximal 80,00 EUR täglich. Die Begrenzung der Abrechnungshäufigkeiten wird die KV durch Regelungen im Abrechnungsprogramm sicherstellen.

**II. Koordination der Versorgung und Assessment**

Für die Koordination der Versorgung und das Assessment der Patientin/des Patienten können einmalig maximal 300,00 EUR je Patientin/je Patienten vergütet werden. Die Gesamtpauschale ist unterteilt in eine Koordinationspauschale und eine Assessmentpauschale mit jeweils 150,00 EUR.

Die Koordination (Koordinationspauschale) der Versorgung umfasst folgende Leistungen:

a. Direkter persönlicher Kontakt erforderlich
b. Ressourcenfokussierte Versorgungsplanung
c. Erstellung eines Versorgungskonzeptes
d. Vernetzung mit ambulanten und/oder stationären Leistungserbringer
e. Dokumentation und Evaluation

**Symbolnummer 92153** **150,00 EUR**

Das Assessment (Assessmentpauschale) der Versorgung umfasst folgende Leistungen/Informationen:

- Aufnahme der SAPV-Anamnese (körperlicher Zustand, Grunderkrankungen, Nebendiagnosen, symptomatische Belastung in Fremd- und Selbstbe-urteilung, bisherige krankheitsmodifizierende Therapie, Medikamenten-anamnese etc.)
- Palliativpflegerische Anamnese
- Psychosoziale Anamnese
- Zugehörigkeitsentscheidung: allgemeine oder spezialisierte ambulante Palliativversorgung

- Entwicklung, Koordination, Festlegung und Dokumentation des Therapiekonzeptes für den SAPV-Fall im multiprofessionellen Team unter Berücksichtigung der medizinischen, pflegerischen und sozialen Bedürfnisse der Patientin/des Patienten sowie der Angehörigen mit Formulierung des spezifisch palliativmedizinischen Behandlungsauftrages und Behandlungszieles
- Abstimmung und ggf. Modifikation des Therapiekonzeptes mit bereits involvierten Leistungserbringern/Kooperationspartnern
- Ermittlung, Information und Einbeziehung potentieller neuer Kooperationspartner zur Umsetzung des Therapiekonzeptes.

**Symbolnummer 92154** **150,00 EUR**

**III. Additiv unterstützende Teilversorgung**

Je Patientin/je Patient und je Einsatz können ärztlich sowie pflegerisch je 40,00 EUR vergütet werden, maximal 135,00 EUR täglich. Die Leistung umfasst im Einzelnen:

a. Beratungs- und Koordinationsleistung
b. Erforderliche Hausbesuche
c. Einzelne Leistungen des in § 5 Abs. 3 der SAPV-Richtlinie des G-BA aufgeführten Leistungskataloges werden bei Bedarf erbracht
d. Für die verordnete Teilversorgung besteht 24-Stunden-Rufbereitschaft in Absprache mit den Leistungserbringern der Primärversorgung. Die Rufbereitschaft kann nicht als alleinige Teilleistung verordnet werden
e. Dokumentation und Evaluation

**Symbolnummer 92155** **40,00 EUR**

Die Begrenzung der Abrechnungshäufigkeiten wird die KV durch Regelungen im Abrechnungsprogramm sicherstellen.

Eine Abrechnung der Teilversorgung neben der vollständigen Versorgung ist am gleichen Tag ausgeschlossen. Die Koordinations- und Assessmentpauschale (II.) ist neben der additiv unterstützenden Teilversorgung gesondert abrechnungsfähig. Die Beratungsleistung (I.) kann nur zusätzlich erbracht und abgerechnet werden, wenn sie ergänzend verordnet wurde und nicht Teil der additiv unterstützenden Teilversorgung ist.

Ist beispielsweise palliativpflegerische Teilversorgung verordnet, so können Beratungsleistungen, die durch die Pflegefachkräfte des Palliative-Care-Teams erbracht werden, nicht gesondert als Beratungsleistung (I.) abgerechnet werden; eine zusätzlich verordnete ärztliche Beratungsleistung durch das Palliative-Care-Team wäre hingegen gesondert abrechnungsfähig.

**IV. Vollständige Versorgung**

Je Patientin/je Patient und je Behandlungstag können 225,00 EUR vergütet werden. Die vollständige Versorgung umfasst neben den Leistungen a. bis d. immer die ärztliche und pflegerische Versorgung

a. Beratungs- und Koordinationsleistung

b. Erforderliche Hausbesuche

c. Einzelne Leistungen des in § 5 Abs. 3 der SAPV-Richtlinie des G-BA aufgeführten Leistungskataloges werden bei Bedarf erbracht

d. Grundpflege nach § 37 SGB V bzw. SGB XI ist nicht Bestandteil der SAPV, kann aber bei entsprechenden Voraussetzungen vom Palliative-Care-Team erbracht und über § 37 Abs. 1 SGB V oder das SGB XI abgerechnet werden.

**Symbolnummer 92157 225,00 EUR**

Eine Abrechnung der Teilversorgung neben der vollständigen Versorgung ist am gleichen Tag ausgeschlossen. Die Abrechnung von Beratungsleistungen (I.) neben der vollständigen Versorgung ist ausgeschlossen. Die Koordinations- und Assessmentpauschale (II.) ist neben der vollständigen Versorgung abrechnungsfähig.

Einsätze, die wegen fehlender Mitwirkung der Patientin/des Patienten nicht erbracht werden können, sind nicht abrechnungsfähig.

## § 2
## Abrechnungsverfahren, Rechnungslegung und Berichtigung

(1) Die nach diesem Vertrag zu vergütenden Leistungen werden durch das Palliative-Care-Team nach Abschluss eines Quartals mit der für den Sitz des Palliative-Care-Teams zuständigen Bezirksstelle der KV abgerechnet.

(2) Die Rechnungslegung ist quartalsweise auf gültigen Abrechnungsscheinen bzw. entsprechend in elektronischer Form vorzunehmen. Dabei sind mehrere, auch elektronische Abrechnungsscheine während eines Quartals für dieselbe Versicherte/denselben Versicherten zulasten derselben Krankenkasse als ein Behandlungsfall für die Abrechnung zusammenzuheften bzw. zuführen. Dies gilt nicht, wenn die Versicherte/ der Versicherte während des Behandlungsquartals die Krankenkasse gewechselt hat. Die Abrechnungsunterlagen sind jeweils nach Beendigung eines Kalendervierteljahres bei der zuständigen Bezirksstelle der KV einzureichen.

(3) Das Einreichen von Abrechnungsunterlagen nach Ablauf eines Jahres, vom Ende des Kalendervierteljahres an gerechnet, in dem die Leistungen erbracht worden sind, ist ausgeschlossen.

(3a) Die Leistungsnachweise gem. § 18 des Vertrages über die Erbringung von SAPV in Nordrhein (Anlage 10) sind bei den zuständigen Krankenkassen einzureichen und mit der Betriebsstättennummer des jeweiligen Palliative-Care-Teams zu versehen. Ebenfalls einzureichen bei den Krankenkassen sind die Kostenzusagen gem. §§ 12, 13 des Vertrages sowie die Nachweis- und Handzeichenliste (Anlage 2). Die Krankenkasse kann einen für den Abrechnungseingang zuständigen Adressaten benennen, der verbindlich bei dem Versand der Dokumentationsunterlagen durch das Palliative-Care-Team zu berücksichtigen ist.

(4) Die Abrechnung der Leistungen ist auf der Grundlage der Richtlinien der Kassenärztlichen Bundesvereinigung für den Einsatz von IT-Systemen in der Arztpraxis zum Zwecke der Abrechnung gemäß § 295 Abs. 4 SGB V (IT-Richtlinien), veröffentlicht im Deutschen Ärzteblatt 25/2005 sowie 12/2008, grundsätzlich mittels EDV vorzunehmen. Dabei hat die Übermittlung der Abrechnungsdaten inklusive Diagnosen, der abrechnungsbegründenden Daten einschließlich Dokumentationen und Qualitätsindikatoren sowie der zu übermittelnden Statistikdaten auf maschinenlesbaren elektronischen Medien zu erfolgen.
Für die Abrechnung mittels EDV ist die Anzeige gegenüber der zuständigen Bezirksstelle der KV erforderlich; sie ist an den Einsatz einer von der KBV zertifizierten Software gebunden. Die Voraussetzungen für eine derartige Abrechnungslegung regelt der Vorstand der KV in Richtlinien.

(5) Das Palliative-Care-Team ist für die ordnungsgemäße Abrechnung verantwortlich. Behandlungsfälle, die nicht den vorstehenden Anforderungen entsprechen, können von der KV zur Berichtigung an das Palliative-Care-Team zurückgegeben werden.

(6) Die KV erteilt für jedes Quartal einen Honorarnachweis, der unter dem Vorbehalt der Nachprüfung durch die Krankenkassen steht, mit mindestens den nachfolgenden Angaben:

a) die Vergütung von Leistungen

b) evtl. Abzüge (z. B. wegen nachträglicher sachlich-rechnerischer Berichtigung der Abrechnung etc.)

c) die Höhe des Abzuges für Verwaltungskostenbeiträge gemäß des jeweils geltenden Beschlusses der Vertreterversammlung der KV

d) der sich aus dem Kontokorrent ergebende Auszahlungsbetrag

Der Honorarnachweis wird in der Regel bis zum Ende des auf das Abrechnungsquartal folgenden vierten Monats an das Palliative-Care-Team übersandt.

(7) Auf das Vierteljahreshonorar erhält das Palliative-Care-Team monatliche Abschlagszahlungen, deren Höhe 31 % des anerkannten Gesamthonorars der letzten durch die KV fertiggestellten Quartalsabrechnung betragen soll. Die Restzahlung erfolgt nach Über-

sendung des Honorarnachweises. Bis zur erstmaligen Übersendung des Honorarnachweises können dem Palliative-Care-Team nach Aufnahme der Tätigkeit monatliche Abschlagszahlungen aufgrund der schriftlich mitgeteilten Zahl der erbrachten Leistungen/Behandlungsfälle in Höhe von 90 % des zu erwartenden Honorars nach diesem Vertrag erteilt werden. Die im Rubrum genannten Kassenverbände/ Krankenkassen verpflichten sich, auf Anforderung der KV entsprechende Zahlungen innerhalb von 10 Kalendertagen an die KV vorzunehmen.

Im Falle der Überzahlung, bei Insolvenz sowie zur Sicherung möglicher Rückforderungs- oder Schadensersatzansprüche oder künftiger Forderungen kann die Abschlags- und/oder Restzahlung reduziert oder ausgesetzt werden.

(8) Die Bezirksstellen der KV überprüfen durch automatisierte Verfahren die Abrechnungsunterlagen u. a. auf sachlich-rechnerische Richtigkeit. Dabei sind sich ergebende sachlich-rechnerische Berichtigungen (z. B. unrichtige Anwendung der Symbolnummern), soweit sie das Palliative-Care-Team beschweren, mitzuteilen. Dies gilt nicht, wenn insbesondere zwingende Bestimmungen des mit dem Palliative-Care-Teams geschlossenen Vertrages außer acht gelassen wurden und/oder die sachlich-rechnerische Berichtigung pro Quartalsabrechnung unter EUR 200 liegt.

(9) Die Leistungen des Palliative-Care-Teams werden im Formblatt 3 unter der Kontenart 406 erfasst und separat unter den in § 1 genannten Symbolnummern ausgewiesen.

(10) Die KV ist berechtigt, den üblichen Verwaltungskostenaufwand gegenüber dem teilnehmenden Palliative-Care-Team bei der Honorarabrechnung in Ansatz zu bringen.

(11) Hinsichtlich der Abrechnung durch die KV, der Zahlungstermine, der sachlich/rechnerischen Berichtigung gelten die Bestimmungen des zwischen den Krankenkassen/Krankenkassenverbänden und der KV geschlossenen Teil A (Honorarvertrag) für das jeweils aktuelle Kalenderjahr. Die Vergütung wird im KT-Viewer gesondert ausgewiesen.

(12) Die KV übermittelt den Krankenkassen die erstellten Abrechnungsdaten mittels Datenträgeraustausch entsprechend der Regelungen des jeweils geltenden Honorarvertrages.

## § 3
## Prüfung der Abrechnung durch die Krankenkassen

(1) Die von der KV erstellte Abrechnung wird gegenüber dem Palliative-Care-Team erst verbindlich, wenn die Krankenkassen innerhalb eines Jahres nach Erhalt der Abrechnungsunterlagen durch die KV bilateral gegenüber dem Palliative-Care-Team keine Berichtigung vorgenommen haben oder die Berichtigungsforderung der betreffenden Krankenkasse endgültig - ggf. durch die Sozialgerichtsbarkeit - für unbegründet erklärt worden ist. Das Palliative-Care-Team ist von einer fristgerecht gestellten Berichtigungs-

forderung einer Krankenkasse unverzüglich durch diese zu unterrichten. Einwendungen hiergegen sind bei der Krankenkasse schriftlich geltend zu machen.

(2) Honorargutschriften für Krankenkassen aufgrund abgeschlossener Berichtigungsforderungen der Krankenkassen werden der KV zur Berücksichtigung mit der nächsterreichbaren Quartalsabrechnung des Palliative-Care-Teams mitgeteilt und die Honorarforderung des Palliative-Care-Teams entsprechend reduziert und den Krankenkassen über Formblatt 3 eine Gutschrift erteilt.

## § 4
## Salvatorische Klausel

Sollten Bestimmungen dieses Vertrages unwirksam sein oder werden oder sollte sich in diesem Vertrag eine Lücke herausstellen, so wird infolgedessen die Gültigkeit der übrigen Bestimmungen des Vertrages nicht berührt. Anstelle der unwirksamen Bestimmungen oder zur Ausfüllung der Lücke ist eine angemessene Regelung zu vereinbaren, die dem am nächsten kommen soll, was die Vertragsschließenden gewollt haben oder nach Sinn und Zweck des Vertrages gewollt haben würden, soweit sie den Punkt beachtet hätten.

## § 5
## Inkrafttreten

(1) Diese Vergütungsvereinbarung tritt zum XX.XX.2009 in Kraft und ist eine Anlage zum Vertrag über die Erbringung „Spezialisierter ambulanter Palliativversorgung (SAPV) in Nordrhein gem. § 132 d SGB V i. V. m. § 37 b SGB V.

(2) Die Anlage kann unter Einhaltung einer Frist von 3 Monaten zum 30. Juni bzw. 31. Dezember eines jeden Kalenderjahres gekündigt werden. Hiervon unberührt bleibt das Recht zur außerordentlichen Kündigung.

# Anlage 5

**Vertrag über die Erbringung**
**Spezialisierter ambulanter Palliativversorgung (SAPV) in Nordrhein**
**gemäß § 132 d SGB V i. V. m. § 37 b SGB V**

**„Richtlinie des Gemeinsamen Bundesausschusses zur Verordnung von spezialisierter ambulanter Palliativversorgung (Spezialisierte Ambulante Palliativversorgungs-Richtlinie/SAPV-RL)“**

# Richtlinie

## des Gemeinsamen Bundesausschusses

### zur Verordnung von spezialisierter ambulanter Palliativversorgung (Spezialisierte Ambulante Palliativversorgungs-Richtlinie / SAPV-RL)

vom 20. Dezember 2007,
veröffentlicht im Bundesanzeiger 2008, S. 911,
in Kraft getreten am 12. März 2008

## Inhalt

§ 1 Grundlagen und Ziele 3

§ 2 Anspruchsvoraussetzungen 3

§ 3 Anforderungen an die Erkrankungen 3

§ 4 Besonders aufwändige Versorgung 4

§ 5 Inhalt und Umfang der spezialisierten ambulanten Palliativversorgung 4

§ 6 Zusammenarbeit der Leistungserbringer 5

§ 7 Verordnung von SAPV 6

§ 8 Prüfung der Leistungsansprüche durch die Krankenkasse 6

## § 1
## Grundlagen und Ziele

(1) Die spezialisierte ambulante Palliativversorgung gemäß § 37 b SGB V (SAPV) dient dem Ziel, die Lebensqualität und die Selbstbestimmung schwerstkranker Menschen zu erhalten, zu fördern und zu verbessern und ihnen ein menschenwürdiges Leben bis zum Tod in ihrer vertrauten häuslichen Umgebung oder in stationären Pflegeeinrichtungen (§ 72 Abs. 1 des Elften Buches des Sozialgesetzbuch - SGB XI) zu ermöglichen. Im Vordergrund steht anstelle eines kurativen Ansatzes die medizinisch-pflegerische Zielsetzung, Symptome und Leiden einzelfallgerecht zu lindern.

(2) Den besonderen Belangen von Kindern ist Rechnung zu tragen.

(3) Die individuellen Bedürfnisse und Wünsche der Patientin oder des Patienten sowie die Belange ihrer oder seiner vertrauten Personen stehen im Mittelpunkt der Versorgung. Der Patientenwille, der auch durch Patientenverfügungen zum Ausdruck kommen kann, ist zu beachten.

(4) Die SAPV ergänzt das bestehende Versorgungsangebot, insbesondere das der Vertragsärzte, Krankenhäuser und Pflegedienste. Sie kann als alleinige Beratungsleistung, additiv unterstützende Teilversorgung oder vollständige Patientenbetreuung erbracht werden. Andere Sozialleistungsansprüche bleiben unberührt.

## § 2
## Anspruchsvoraussetzungen

Versicherte haben Anspruch auf SAPV, wenn

a) sie an einer nicht heilbaren, fortschreitenden und so weit fortgeschrittenen Erkrankung leiden, dass dadurch ihre Lebenserwartung begrenzt ist (§ 3) und

b) sie unter Berücksichtigung der in § 1 genannten Ziele eine besonders aufwändige Versorgung (§ 4) benötigen, die nach den medizinischen und pflegerischen Erfordernissen auch ambulant oder in stationären Pflegeeinrichtungen (§ 72 SGB XI) erbracht werden kann.

## § 3
## Anforderungen an die Erkrankungen

(1) Eine Erkrankung ist nicht heilbar, wenn nach dem allgemein anerkannten Stand der medizinischen Erkenntnisse Behandlungsmaßnahmen nicht zur Beseitigung dieser Erkrankung führen können.

(2) Sie ist fortschreitend, wenn ihr Verlauf trotz medizinischer Maßnahmen nach dem allgemein anerkannten Stand der medizinischen Erkenntnisse nicht nachhaltig aufgehalten werden kann.

(3) Eine Erkrankung ist weit fortgeschritten, wenn die Verbesserung von Symptomatik und Lebensqualität sowie die psychosoziale Betreuung im Vordergrund der Versorgung stehen und nach begründeter Einschätzung der verordnenden Ärztin oder des verordnenden Arztes die Lebenserwartung auf Tage, Wochen oder Monate gesunken ist. Insbesondere bei Kindern sind die Voraussetzungen für die SAPV als Krisenintervention auch bei einer länger prognostizierten Lebenserwartung erfüllt.

## § 4
## Besonders aufwändige Versorgung

Bedarf nach einer besonders aufwändigen Versorgung besteht, soweit die anderweitigen ambulanten Versorgungsformen sowie ggf. die Leistungen des ambulanten Hospizdienstes nicht oder nur unter besonderer Koordination ausreichen würden, um die Ziele nach § 1 Abs. 1 zu erreichen. Anhaltspunkt dafür ist das Vorliegen eines komplexen Symptomgeschehens, dessen Behandlung spezifische palliativmedizinische und/oder palliativpflegerische Kenntnisse und Erfahrungen sowie ein interdisziplinär, insbesondere zwischen Ärzten und Pflegekräften in besonderem Maße abgestimmtes Konzept voraussetzt. Ein Symptomgeschehen ist in der Regel komplex, wenn mindestens eines der nachstehenden Kriterien erfüllt ist:

- ausgeprägte Schmerzsymptomatik
- ausgeprägte neurologische/psychiatrische/psychische Symptomatik
- ausgeprägte respiratorische/kardiale Symptomatik
- ausgeprägte gastrointestinale Symptomatik
- ausgeprägte ulzerierende/exulzerierende Wunden oder Tumore
- ausgeprägte urogenitale Symptomatik

## § 5
## Inhalt und Umfang der spezialisierten ambulanten Palliativversorgung

(1) Die SAPV umfasst je nach Bedarf alle Leistungen der ambulanten Krankenbehandlung soweit diese erforderlich sind, um die in § 1 Abs. 1 genannten Ziele zu erreichen. Sie umfasst zusätzlich die im Einzelfall erforderliche Koordination der diagnostischen, therapeutischen und pflegerischen Teilleistungen sowie die Beratung, Anleitung und Begleitung der verordnenden oder behandelnden Ärztin oder des verordnenden oder behandelnden Arztes sowie der sonstigen an der allgemeinen Versorgung beteiligten Leistungserbringer, der Patienten und ihrer Angehörigen durch Leistungserbringer nach § 132 d SGB V.

(2) SAPV wird ausschließlich von Leistungserbringern nach § 132 d SGB V erbracht. Sie wird nach Bedarf intermittierend oder durchgängig erbracht, soweit das bestehende ambulante Versorgungsangebot (§ 1 Abs. 4), insbesondere die allgemeine Palliativversorgung nicht ausreicht, um die Ziele nach § 1 Abs. 1 zu erreichen. Sie kann dem jeweiligen aktuellen Versorgungsbedarf entsprechend als

- Beratungsleistung
- Koordination der Versorgung
- additiv unterstützende Teilversorgung
- vollständige Versorgung

erbracht werden. Die Leistungen müssen ausreichend und zweckmäßig sein, dürfen das Maß des Notwendigen nicht überschreiten und sind wirtschaftlich zu erbringen.

(3) Inhalte der SAPV sind insbesondere:

- Koordination der spezialisierten palliativmedizinischen und palliativpflegerischen Versorgung unter Einbeziehung weiterer Berufsgruppen und von Hospizdiensten im Rahmen einer multiprofessionellen Zusammenarbeit
- Symptomlinderung durch Anwendung von Medikamenten oder anderen Maßnahmen
- Apparative palliativmedizinische Behandlungsmaßnahmen (z. B. Medikamentenpumpe)
- Palliativmedizinische Maßnahmen, die nach ihrer Art, Schwere oder Komplexität eine Kompetenz erfordern, die der einer Ärztin oder eines Arztes mit Zusatzweiterbildung Palliativmedizin entspricht

- Spezialisierte palliativpflegerische Leistungen, die nach ihrer Art, Schwere oder Komplexität eine Kompetenz erfordern, die der einer Pflegefachkraft mit einer curricularen Weiterbildung zu Palliative Care entspricht
- Führung eines individuellen Behandlungsplans, vorbeugendes Krisenmanagement, Bedarfsinterventionen
- Ruf-, Notfall- und Kriseninterventionsbereitschaft rund um die Uhr für die im Rahmen der SAPV betreuten Patienten zur Sicherstellung der im Rahmen der SAPV erforderlichen Maßnahmen
- Beratung, Anleitung und Begleitung der Patienten und ihrer Angehörigen zur palliativen Versorgung einschließlich Unterstützung beim Umgang mit Sterben und Tod
- Spezialisierte Beratung der betreuenden Leistungserbringer der Primärversorgung
- Psychosoziale Unterstützung im Umgang mit schweren Erkrankungen in enger Zusammenarbeit z. B. mit Seelsorge, Sozialarbeit und ambulanten Hospizdiensten
- Organisation regelmäßiger Fallbesprechungen
- Dokumentieren und Evaluieren der wesentlichen Maßnahmen im Rahmen der SAPV

## § 6
## Zusammenarbeit der Leistungserbringer

(1) Im Rahmen der SAPV ist zu gewährleisten, dass die an der Versorgung beteiligten Leistungserbringer die erforderlichen Maßnahmen aufeinander abgestimmt und bedarfsgerecht erbringen; die diesbezügliche Koordination ist sicherzustellen. Hierüber sind verbindliche Kooperationsvereinbarungen schriftlich oder mündlich zu treffen. Kooperationspartner ist auch der ambulante Hospizdienst, der auf Wunsch der Patientin oder des Patienten an der Versorgung beteiligt wird. Bei Bedarf und entsprechender Qualifikation kann die dauer-behandelnde Ärztin oder der dauerbehandelnde Arzt im Einzelfall Kooperationspartnerin oder Kooperationspartner werden. Das Nähere regeln die Verträge nach § 132 d SGB V.

(2) Die vorhandenen Versorgungsstrukturen sind zu beachten.

(3) Es ist zu gewährleisten, dass zwischen den an der Patientenversorgung beteiligten Leistungserbringern zeitnah alle notwendigen Informationen über die vorhergehende Behandlung unter Berücksichtigung datenschutzrechtlicher Regelungen ausgetauscht werden.

(4) Bei der SAPV ist der ärztlich und pflegerisch erforderliche Entscheidungsspielraum für die Anpassung der Palliativversorgung an die Besonderheiten des Einzelfalls zu berücksichtigen.

(5) Für die notwendigen koordinativen Maßnahmen ist vernetztes Arbeiten innerhalb der gewachsenen Strukturen der Palliativversorgung unabdingbar. Dieses ist unter Berücksichtigung medizinischer, pflegerischer, physiotherapeutischer, psychologischer, psychosozialer und spiritueller Anforderungen zur lückenlosen Versorgung über die Sektorengrenzen hinweg zu fördern und auszubauen.

## § 7
## Verordnung von SAPV

(1) SAPV wird von der behandelnden Vertragsärztin oder von dem behandelnden Vertragsarzt nach Maßgabe dieser Richtlinie verordnet. Satz 1 gilt für die Behandlung durch die Krankenhausärztin oder den Krankenhausarzt bei einer oder einem von ihr oder ihm ambulant versorgten Patientin oder Patienten entsprechend. Hält eine Krankenhausärztin oder ein Krankenhausarzt die Entlassung einer Patientin oder eines Patienten für möglich und ist aus ihrer oder seiner Sicht SAPV erforderlich, kann die Krankenhausärztin oder der Krankenhausarzt die Verordnung ausstellen, längstens jedoch für 7 Tage.

(2) Die ärztliche Verordnung erfolgt auf einem zu vereinbarenden Vordruck, welcher der Leistungserbringung nach dem jeweiligen aktuellen Versorgungsbedarf (§ 5 Abs. 2) Rechnung zu tragen hat und Angaben zur Dauer der Verordnung enthält.

## § 8
## Prüfung der Leistungsansprüche durch die Krankenkasse

Die Krankenkasse übernimmt bis zu einer Entscheidung über die weitere Leistungserbringung die Kosten für die verordneten und von den Leistungserbringern nach § 132 d SGB V erbrachten Leistungen entsprechend der vereinbarten Vergütung nach § 132 d SGB V, wenn die Verordnung gemäß § 7 Abs. 2 spätestens an dem dritten der Ausstellung folgenden Arbeitstag der Krankenkasse vorgelegt wird. Das Nähere regeln die Vertragspartner nach § 132 d SGB V.

Die SAPV-Richtlinie tritt am Tag nach der Veröffentlichung im Bundesanzeiger in Kraft.

Siegburg, den 20. Dezember 2007

Gemeinsamer Bundesausschuss
Der Vorsitzende
Hess

# Anlage 6

**Vertrag über die Erbringung**
**Spezialisierter ambulanter Palliativversorgung (SAPV) in Nordrhein**
**gemäß § 132 d SGB V i. V. m. § 37 b SGB V**

**„Gemeinsame Empfehlung der Spitzenverbände der Kranken-**
**kassen**
**nach § 132 d Abs. 2 SGB V**
**für die spezialisierte ambulante Palliativversorgung“**

## Gemeinsame Empfehlungen

des AOK-Bundesverbandes, Bonn
des BKK Bundesverbandes, Essen
des Bundesverbandes der landwirtschaftlichen Krankenkassen, Kassel
des IKK-Bundesverbandes, Bergisch Gladbach
der Knappschaft, Bochum
des Verbandes der Angestellten-Krankenkassen e.V., Siegburg
des AEV Arbeiter-Ersatzkassen-Verband e.V., Siegburg

## nach § 132 d Abs.2 SGB V

## für die spezialisierte ambulante Palliativversorgung

**vom 23.06.2008**

unter Beteiligung

des Arbeiterwohlfahrt Bundesverbandes e.V., Berlin
des Arbeitgeber- und BerufsVerbandes Privater Pflege e.V., Hannover
der Bundesarbeitsgemeinschaft Hauskrankenpflege e.V., Berlin
der Bundesarbeitsgemeinschaft der PatientInnenstellen, München
des Bundesverbandes Ambulante Dienste und Stationäre Einrichtungen e.V., Essen
der Bundesinitiative Ambulante Psychiatrische Pflege e.V., Berlin
des Bundesverbandes Deutscher Privatkliniken e.V. Berlin
des Bundesverbandes Häusliche Kinderkrankenpflege e.V., Köln
des Bundesverbandes Kinderhospiz e.V., Freiburg
des Bundesverbandes privater Anbieter sozialer Dienste e.V., Berlin
der Deutschen Arbeitsgemeinschaft Selbsthilfegruppen e.V., Gießen
des Deutschen Bundesverbandes für Pflegeberufe, Berlin
des Deutschen Caritasverbandes e.V., Freiburg
der Deutschen Gesellschaft für Palliativmedizin e.V., Berlin
der Deutschen Hospiz Stiftung, Dortmund
des Deutschen Hospiz- und PalliativVerbandes e.V., Berlin
des Deutschen Kinderhospizvereines e.V., Olpe
der Deutschen Krankenhaus-Gesellschaft, Berlin
des Deutschen Paritätischen Wohlfahrtsverbandes, Gesamtverband e.V., Berlin
des Deutschen Pflegerates, Berlin
des Deutschen Roten Kreuzes, Generalsekretariat, Berlin
des Diakonischen Werkes der EKD e.V., Berlin
der Kassenärztlichen Bundesvereinigung, Berlin
des Sozialverbandes VdK Deutschland e.V., Berlin
des Verbandes Deutscher Alten- und Behinderten Hilfe e.V., Essen
der Verbraucherzentrale Bundesverband e.V., Berlin
die Zentralwohlfahrtsstelle der deutschen Juden in Deutschland e.V., Frankfurt am Main

## 1. Zielsetzung

1.1 Die spezialisierte ambulante Palliativversorgung (SAPV) dient dem Ziel, die Lebensqualität und die Selbstbestimmung schwerstkranker Menschen zu erhalten, zu fördern und zu verbessern und ihnen ein menschenwürdiges Leben bis zum Tod in ihrer vertrauten häuslichen Umgebung oder in einer stationären Pflegeeinrichtung zu ermöglichen.

1.2 Die SAPV ist fachlich kompetent nach den allgemein anerkannten medizinischen und pflegewissenschaftlichen Erkenntnissen bedarfsgerecht und wirtschaftlich zu erbringen. Die individuellen Bedürfnisse und Wünsche der Patientin oder des Patienten sowie die Belange ihrer oder seiner vertrauten Personen stehen im Mittelpunkt der Versorgung.

1.3 Die Spitzenverbände der Krankenkassen werden die Erfahrungen mit der Umsetzung dieser Empfehlungen fortlaufend auswerten und diese erforderlichenfalls weiterentwickeln.

## 2. Zulassungsvoraussetzungen

2.1 SAPV wird von Leistungserbringern erbracht, mit denen die Krankenkasse zur Sicherung einer bedarfsgerechten Versorgung einen Vertrag geschlossen hat. Sie wird intermittierend oder durchgängig nach Bedarf als

- Beratungsleistung
- Koordination der Versorgung
- additiv unterstützende Teilversorgung
- vollständige Versorgung

erbracht. Den besonderen Belangen von Kindern und Jugendlichen ist Rechnung zu tragen.

2.2 Bei der Entwicklung einer bedarfsgerechten Versorgung sind die bereits bestehenden Strukturen so weit wie möglich einzubeziehen. Auf die bereits tätigen Leistungserbringer ist zurückzugreifen, soweit sie die erforderlichen Anforderungen erfüllen. Verträge sind nur in dem Umfang abzuschließen, wie sie für eine bedarfsgerechte Versorgung erforderlich sind. Ein Anspruch auf Vertragsabschluss besteht nicht.

2.3 Eine bedarfsgerechte Versorgung mit SAPV ist insbesondere dann gegeben, wenn sie wohnortnah ausgerichtet ist und die Palliativpatienten, die einen besonderen Versorgungsbedarf haben, der durch die allgemeine Palliativversorgung nicht gewährleistet werden kann, ausreichend und zweckmäßig mit der Leistung der SAPV versorgt werden können. Die bedarfsgerechte Versorgung zeigt sich auch darin, dass es mit der Leistung der SAPV mehr Menschen als bisher ermöglicht wird, in ihrer vertrauten häuslichen Umgebung oder in stationären Pflegeeinrichtungen ein menschenwürdiges Leben bis zum Tod zu führen.

2.4 Der Versorgungsbedarf kann in einzelnen Regionen unterschiedlich sein. Indikatoren für die Bedarfslage können insbesondere sein:

- Regionale Siedlungsstruktur
- Altersstruktur
- Epidemiologisch relevante Erkrankungen
- Demografische Entwicklung

So ist zwischen Regionen geringer, mittlerer und hoher Bevölkerungsdichte zu unterscheiden. Die Unterscheidung ist ggf. nicht allein auf der Ebene der Bundesländer durchzuführen, sondern kann bis auf die Ebene der Kreise und kreisfreien Städte vollzogen werden, um dem erheblichen Unterschied in der Bevölkerungsdichte auch innerhalb der Bundesländer gerecht zu werden. Nach derzeit vorliegenden Schätzungen haben bis zu 10 % aller Sterbenden einen solchen besonderen Versorgungsbedarf, der im Rahmen der SAPV abzudecken ist.

## 3. Inhalt und Umfang der Leistungen

3.1 Inhalt und Umfang der zu erbringenden SAPV-Leistungen ergeben sich aus der gemäß der SAPV-Richtlinie nach § 37 b SGB V in Verbindung mit § 92 Abs.1 Satz 2 Nr. 14 SGB V ausgestellten und genehmigten Verordnung. Die Krankenkasse übernimmt bis zu einer Entscheidung über die weitere Leistungserbringung die Kosten für die verordneten und von den Leistungserbringern nach § 132 d SGB V erbrachten Leistungen entsprechend der vereinbarten Vergütung nach § 132 d SGB V, wenn die Verordnung gemäß § 7 Abs. 2 der SAPV-Richtlinie spätestens an dem dritten der Ausstellung folgenden Arbeitstag der Krankenkasse vorgelegt wird.

3.2 Die Leistungen müssen ausreichend und zweckmäßig sein, dürfen das Maß des Notwendigen nicht überschreiten und sind wirtschaftlich zu erbringen. Soweit die allgemeine Palliativversorgung ausreichend ist, darf SAPV nicht erbracht werden.

3.3 Im Falle der Besserung bzw. einer Stabilisierung im Krankheitsverlauf ist der Versorgungsumfang der SAPV so weit wie möglich zu reduzieren und eine Weiterversorgung im Rahmen der allgemeinen Palliativversorgung anzustreben.

3.4 In den Vereinbarungen über die SAPV sind Regelungen zu treffen, die sicherstellen, dass für den Palliativpatienten eine reibungslose Versorgung mit Arznei- Heil- und Hilfsmitteln auch weiterhin gewährleistet ist.

## 4. Organisatorische Voraussetzungen

4.1 Die Leistungen der SAPV werden durch spezialisierte Leistungserbringer erbracht. Spezialisierte Leistungserbringer sind nach Nr. 5.2 qualifizierte Ärztinnen oder Ärzte und nach Nr. 5.3 qualifizierte Pflegefachkräfte, die orientiert an der Konzeption eines Palliativ-Care-Teams fachübergreifend ggf. auch in Kooperation mit anderen Professionen (s. Nr. 5.4) eng zusammenarbeiten. Sie erfüllen ergänzend besondere sächliche Voraussetzungen nach Nr. 4.4 und Nr. 4.5, die für eine spezialisierte palliativmedizinische und palliativpflegerische Versorgung erforderlich sind. Eine psychosoziale Unterstützung ist in enger Zusammenarbeit z.B. mit ambulanten Hospizdiensten nach § 39 a Abs. 2 SGB V, Seelsorge und Sozialarbeit zu gewährleisten.

4.2 Die spezialisierten Leistungserbringer sind Teil einer multiprofessionell vernetzten Versorgungsstruktur im regionalen Gesundheits- und Sozialsystem. Sie arbeiten mit den an der Versorgung beteiligten Leistungserbringern, wie Hausärzten und ambulanten Hospizdiensten gemäß § 39 a Abs. 2 SGB V eng zusammen (integrativer Ansatz). Mit den regelhaft an der Versorgung beteiligten Leistungserbringern sind Kooperationsvereinbarungen schriftlich abzuschließen und der Krankenkasse vorzulegen.

4.3 Die spezialisierten Leistungserbringer arbeiten nach einem verbindlichen, strukturierten und schriftlich dargelegten Konzept, in dem der inhaltliche und organisatorische Rahmen der Leistungserbringung (inkl. der personellen und sächlichen Ausstattung) sowie die Einbindung in die regionale Versorgungsstruktur beschrieben sind. Das Konzept ist der Krankenkasse vorzulegen.

4.4 Die spezialisierten Leistungserbringer haben als Mindestanforderung an die sächliche Ausstattung Folgendes vorzuhalten bzw. sicherzustellen:

- eine geeignete, aktuell geführte und für die an der Versorgung Beteiligten jederzeit zugängliche Patientendokumentation
- Arzneimittel (inkl. BtM) für die Notfall/Krisenintervention
- Arzt-/Pflegekoffer/Bereitschaftstasche (Berücksichtigung der Kompatibilität der Verbrauchsmaterialien zu Medizinprodukten unterschiedlicher Hersteller, z.B. bei Portsystemen oder Infusionspumpen)
- eine geeignete administrative Infrastruktur, z. B. Büro, Kommunikationstechnik

4.5 Die spezialisierten Leistungserbringer müssen über eine eigenständige Adresse und geeignete Räumlichkeiten für

- die Beratung von Patienten und Angehörigen
- Teamsitzungen und Besprechungen
- die Lagerhaltung von eigenen Medikamenten für Notfall-/ Krisenintervention und Hilfsmitteln

verfügen. Sofern eine Aufbewahrung von Medikamenten erfolgt, die unter das Betäubungsmittelgesetz (BtM-G) fallen, ist ein BtM-Schrank (§ 15 BtM-G) erforderlich.

## 5. Personelle Anforderungen

5.1 Die spezialisierten Leistungserbringer stellen sicher, dass das für die spezialisierte ambulante Palliativversorgung erforderliche und geeignete Personal zur Verfügung steht. Dabei ist zu berücksichtigen, dass durch den spezialisierten Leistungserbringer eine tägliche telefonische Rund-um-die-Uhr-Erreichbarkeit für die Patienten, deren Angehörigen und die an der Versorgung Beteiligten sicherzustellen ist. Die ständige Verfügbarkeit mindestens einer Ärztin/eines Arztes und/oder einer Pflegefachkraft ist zu gewährleisten. Die Verfügbarkeit schließt notwendige Hausbesuche ein.

5.2 Die nach Nr. 4.1 tätigen Ärztinnen und Ärzte verfügen über

- eine anerkannte Zusatzweiterbildung Palliativmedizin nach der aktuell gültigen Weiterbildung der jeweiligen Landesärztekammer (grds. 160 Std. Weiterbildung)

  und

- Erfahrung aus der ambulanten palliativen Behandlung von mindestens 75 Palliativpatienten, z. B. in der häuslichen Umgebung oder in einem stationären Hospiz, innerhalb der letzten drei Jahre oder aus einer mindestens einjährigen klinischen palliativmedizinischen Tätigkeit in einer Palliativabteilung in einem Krankenhaus innerhalb der letzten drei Jahre.

5.3 Die nach Nr. 4.1 tätigen Pflegefachkräfte verfügen über:

- die Erlaubnis zur Führung einer der Berufsbezeichnungen Gesundheits- und Krankenpflegerin/Gesundheits- und Krankenpfleger, Gesundheits- und Kinderkrankenpflegerin/Gesundheits- und Kinderkrankenpfleger oder Altenpflegerin/Altenpfleger (dreijährige Ausbildung aufgrund einer landesrechtlichen Regelung) entsprechend den gesetzlichen Bestimmungen in der jeweils gültigen Fassung und

- den Abschluss einer Palliative-Care-Weiterbildungsmaßnahme im Umfang von mindestens 160 Stunden

  und

- Erfahrung durch mindestens eine zweijährige praktische Tätigkeit als Pflegefachkraft in der Betreuung von Palliativpatienten in den letzten drei Jahren; davon mindestens sechsmonatige Mitarbeit in einer spezialisierten Einrichtung der Hospiz- und Palliativversorgung.

5.4 Soweit weitere Fachkräfte (z. B. Sozialarbeiterinnen/Sozialarbeiter, Sozialpädagoginnen oder Sozialpädagogen, Psychologinnen oder Psychologen) vertraglich eingebunden werden, haben diese eine Zusatzweiterbildung Palliative Care für andere Berufsgruppen oder eine mehrjährige Erfahrung in der Palliativversorgung nachzuweisen.

## 6. Qualitätssicherung

6.1 Die spezialisierten Leistungserbringer sind verpflichtet, ein internes Qualitätsmanagement durchzuführen. Sie nehmen regelmäßig an palliativ-medizinischen/-pflegerischen Fortbildungen teil und führen möglichst halbjährlich multidisziplinäre Qualitätszirkel durch, an denen auch die übrigen in der Versorgung Tätigen teilnehmen. Die spezialisierten Leistungserbringer sollen sich außerdem an Maßnahmen der externen Qualitätssicherung beteiligen.

6.2 Ein geeignetes Dokumentationssystem ist sachgerecht und kontinuierlich zu führen und auf Wunsch der Krankenkasse als Konzept vorzulegen. Das Dokumentationssystem muss patientenbezogene Daten und - soweit vorhanden - allgemein anerkannte Indikatoren für eine externe Qualitätssicherung enthalten und eine bundesweite Evaluation ermöglichen. Es soll kompatibel zu den bestehenden Dokumentationssystemen der an der Versorgung beteiligten Leistungserbringer sein.

6.3 Die Teilnahme an Supervision ist zu ermöglichen. Regelmäßige multiprofessionelle Fallbesprechungen sind in überschaubaren Intervallen durchzuführen; die inhaltliche und zeitliche Organisation ist den Beteiligten überlassen.

6.4 Die spezialisierten Leistungserbringer erstellen einen jeweils individuellen Behandlungsplan, der mit den übrigen an der Versorgung beteiligten Leistungserbringern abzustimmen ist.

# Anlage 7

**Vertrag über die Erbringung**
**Spezialisierter ambulanter Palliativversorgung (SAPV) in Nordrhein**
**gemäß § 132 d SGB V i. V. m. § 37 b SGB V**

**„Dokumentation“**

Deutsche Gesellschaft für Palliativmedizin e.V. — Deutscher Hospiz- und PalliativVerband e.V.

## Gemeinsamer Datensatz zur SAPV-Dokumentation (Version 1 / 15.01.2009)

| I. Kerndatensatz für Palliativpatienten: Erfassung zu Beginn und Ende der Palliativversorgung | |
|---|---|
| Items | Antworten, Kategorien |
| **Demographische Angaben** | |
| Geburtsdatum | Datum |
| Aufnahmedatum | Datum |
| Geschlecht | \| weiblich \| männlich |
| Wohnsituation | \| allein \| Heim \| mit Angehörigen \| Sonstige |
| **Angaben zur Erkrankung** | |
| Hauptdiagnose/Grundkrankheit (palliativmedizinisch relevant) | ICD |
| Funktionsstatus (ECOG) | \| Normale Aktivität \| Gehfähig, leichte Arbeit möglich<br>\| Nicht arbeitsfähig, kann > 50% der Wachzeit aufstehen<br>\| Begrenzte Selbstversorgung, > 50% Wachzeit bettlägerig<br>\| Pflegebedürftig, permanent bettlägerig |
| **Angaben zur Versorgung (Strukturqualität)** | |
| Behandlung, Begleitung<br>(bei Behandlungsbeginn<br>oder bei Übernahme des Patienten) | ambulant:<br>\| SAPV-Team \| Hausarzt \| Ambulante Pflege<br>\| Palliativarzt (QPA) \| Palliativpflege (AHPP, APD)<br>\| Palliativberatung (AHPB) \| Ehrenamtlicher Dienst<br>stationär:<br>\| KH (Allgemeinstation) \| KH (Palliativstation)<br>\| Heim \| Stationäres Hospiz \| Sonstige |
| Absprache / Vorlage | \| Patientenverfügung \| Vorsorgevollmacht \| Betreuung |
| Datum der Erfassung | Datum |
| **Angaben zum Behandlungsbedarf** | |
| Schmerzen | \| kein \| leicht \| mittel \| stark |
| Übelkeit | \| kein \| leicht \| mittel \| stark |
| Erbrechen | \| kein \| leicht \| mittel \|.stark |
| Luftnot | \| kein \| leicht \| mittel \| stark |
| Verstopfung | \| kein \| leicht \| mittel \| stark |
| Schwäche | \| kein \| leicht \| mittel \| stark |
| Appetitmangel | \| kein \| leicht \| mittel \| stark |
| Müdigkeit | \| kein \| leicht \| mittel \| stark |
| Pflegeprobleme wegen Wunden / Dekubitus | \| kein \| leicht \| mittel \| stark |
| Hilfebedarf bei Aktivitäten des tägl. Lebens | \| kein \| leicht \| mittel \| stark |
| Depressivität | \| kein \| leicht \| mittel \| stark |
| Angst | \| kein \| leicht \| mittel \| stark |
| Anspannung | \| kein \| leicht \| mittel \| stark |
| Desorientiertheit, Verwirrtheit | \| kein \| leicht \| mittel \| stark |
| Probleme mit Organisation der Versorgung | \| kein \| leicht \| mittel \| stark |
| Überforderung der Familie, des Umfeldes | \| kein \| leicht \| mittel \| stark |
| **Angaben zur Therapie (Prozessqualität)** | |
| Was ist der Grund des Kontaktes, was das Behandlungsziel? | Text |
| Opioide der WHO Stufe III | ja / nein |
| Steroide | ja / nein |
| Chemotherapie | nein / fortgesetzt / initiiert |
| Strahlentherapie | nein / fortgesetzt / initiiert |
| besonderer Aufwand mit: | Text |

| Angaben zum weiteren Verlauf, Abschluss (Ergebnisqualität) | |
|---|---|
| Welches Problem konnte besonders gut gelöst werden? | Text |
| Welches Problem konnte nicht ausreichend gelöst werden? | Text |
| Datum (Entlassung / Änderung der Betreuung / Tod) | Datum |
| Therapieende | \| Verstorben \| Verlegung, Entlassung<br>\| Sonstiges: |
| Sterbeort | \| zuhause \| Heim \| Krankenhaus<br>\| Palliativstation \| Hospiz \| unbekannt |
| Zufriedenheit mit der Behandlung für die gesamte Behandlung | \| sehr schlecht \| schlecht \| mittel<br>\| gut \| sehr gut |

| II. SAPV-Struktur-Modul:<br>Erfassung bei jeder Verordnung bzw. Intensitätswechsel | |
|---|---|
| Items | Antworten, Kategorien |
| Identifikationsnummer | Fortlaufende Nr. wie bei Kerndatensatz |
| Angaben zur Versorgung (Strukturqualität) | |
| SAPV | \| Beratung \| Koordination<br>\| additive Teilversorgung \| vollständige Versorgung |
| Verordnung Datum | Datum |
| Art der Verordnung | \| Erstverordnung \| Wiederverordnung |
| Verordnung durch | \| Hausarzt \| niedergelassener Facharzt \| KH-Arzt<br>\| andere \| keine Angabe |
| Übernahme aus | \| Eigene Häuslichkeit \| Ambulantes Umfeld<br>\| Stationäre Pflegeeinrichtung \| Stationäres Hospiz<br>\| KH (Allgemeinstation) \| KH (Palliativstation) |
| Beteiligte assoziierte Partner in der SAPV-Erbringung | Ärztlich:<br>\| Hausarzt \| niedergelassener Facharzt (für_______)<br>\| KH-Arzt \| Sonstige<br><br>Pflegerisch:<br>\| Ambulanter Pflegedienst<br>\| Ambulanter Palliativpflegedienst<br>\| Stationäre Pflegeeinrichtung<br>\| Stationäres Hospiz<br><br>Ambulanter Hospizdienst:<br>\| Ambulante Palliativberatung (AHD, AHPB)<br>\| Begleitung durch Ehrenamtliche<br><br>Weitere Professionen:<br>\| Case Management \| Ernährungsberatung<br>\| Physiotherapie \| Psychologie \| Seelsorge<br>\| Sozialarbeit \| Andere:_______________________ |
| Regel-Anfahrtsweg in km | |
| Angaben zum Verlauf | |
| Anzahl der Teambesprechungen zur Problemlösung (Definition Teambesprechung in Expose) | |
| Krankenhauseinweisungsgrund | Text |
| Datum Ende der SAPV | |
| Angaben zum Team | |
| Zahl der Vollzeitäquivalente (multiprofessionell) im SAPV Team | |
| Zahl der zeitgleich maximal betreuten Patienten je Versorgungsstufe | |
| Datum der Erfassung | |

# Anlage 8

**Vertrag über die Erbringung
Spezialisierter ambulanter Palliativversorgung (SAPV) in Nordrhein
gemäß § 132 d SGB V i. V. m. § 37 b SGB V**

**„Vertrag über die ambulante palliativpflegerische Versorgung
nach § 132 a Abs. 2 SGB V“**

# Vertrag
## über die ambulante palliativpflegerische Versorgung
## nach § 132 a Abs. 2 SGB V

zwischen

als Träger der -nachfolgend Träger genannt-

- nachfolgend Leistungserbringer genannt -

und

der AOK Rheinland/Hamburg - Die Gesundheitskasse
dem BKK Landesverband Nordrhein-Westfalen
der IKK Nordrhein
der Knappschaft
der Landwirtschaftlichen Krankenkasse Nordrhein-Westfalen, zugleich handelnd für die Krankenkasse für den Gartenbau,
sowie den Ersatzkassen
BARMER Ersatzkasse
Techniker Krankenkasse
Deutsche Angestellten-Krankenkasse
Kaufmännische Krankenkasse - KKH - Allianz
Gmünder Ersatzkasse
HEK - Hanseatische Krankenkasse
Hamburg Münchener Krankenkasse
hkk
gemeinsamer Bevollmächtigter mit Abschlussbefugnis
gemäß § 212 Abs. 5 S. 6 SGB V:
Verband der Ersatzkassen e. V. (vdek),
vertreten durch den Leiter der Landesvertretung Nordrhein-Westfalen

- nachfolgend Krankenkassen genannt –

wird folgender Vertrag geschlossen:

# Inhaltsverzeichnis:

Präambel
§ 1 Gegenstand des Vertrages
§ 2 Geltungsbereich
§ 3 Organisatorische Voraussetzungen
§ 4 Personelle Voraussetzungen
§ 5 Nachweis der personellen und organisatorischen Voraussetzungen
§ 6 Anzeigepflichten/Mitteilungspflichten
§ 7 Kooperation - Sicherstellung der Ehrenamtlichen
§ 8 Inhalt der Leistungen
§ 9 Materialien und Geräte
§ 10 Grundsätze der Leistungserbringung
§ 11 Qualitätssicherung
§ 12 Pflegeleitbild
§ 13 Pflegekonzept
§ 14 Stellenbeschreibungen/Einarbeitungskonzept
§ 15 Fort- und Weiterbildung
§ 16 Dienst-/Einsatzplankonzept
§ 17 Pflegeprozess
§ 18 Pflegedokumentation
§ 19 Leistungsnachweis
§ 20 Personenkreis

§ 21 Abgabe der Leistungen/Kostenübernahme/
Vergütungsanspruch
§ 22 Vergütung
§ 23 Abrechnung gemäß § 302 SGB V
§ 23 a Abrechnung vor Umsetzung des § 302 SGB V
§ 24 Rechnungslegung durch Abrechnungsstellen
§ 25 Zahlungsfrist/Beanstandung
§ 26 Ausschlussfrist
§ 27 Verbot der Einflussnahme und Vermittlungsverbot
§ 28 Datenschutz/Schweigepflicht/geschäftsmäßige
Rechtsberatung
§ 29 Pflegevertrag
§ 30 Zahlung bei Feststellung von Qualitätsmängeln
§ 31 Maßnahmen bei Gesetzes- bzw. Vertragsverstößen,
Wiedergutmachung des Schadens
§ 32 Außerordentliche Kündigung
§ 33 Auswertung
§ 34 Schiedsperson
§ 35 Beendigung des Vertrages
§ 36 In-Kraft-Treten, Kündigung
§ 37 Überleitungsregelung
§ 38 Änderungen und Ergänzungen
§ 39 Salvatorische Klausel

## Präambel

Das Ziel der ambulanten Palliativversorgung ist, Sterbenden (§ 20) zu ermöglichen, die letzte Phase ihres Lebens in häuslicher Umgebung zu verbringen. Hiermit soll dem sterbenden Menschen ein würdevolles und selbst bestimmtes Leben bis zum Ende ermöglicht werden. Basis dieses Vertrages sind die im Rahmenprogramm zur flächendeckenden Umsetzung der ambulanten palliativmedizinischen und palliativpflegerischen Versorgung in NRW vom 19.05.2005 aufgestellten Grundsätze und Maßstäbe für die ambulante Palliativversorgung. Dieser Vertrag soll die Grundlage für eine kooperative und integrative sowie palliativpflegerische Versorgung für den ambulanten Bereich im Sinne einer ganzheitlichen Versorgung sein. Um dies zu erreichen, sind alle Beteiligten bereit, eng und ohne Zeit- und Reibungsverluste zusammenzuarbeiten.

## § 1
## Gegenstand des Vertrages

Gegenstand dieses Vertrages ist die Erbringung der ambulanten palliativ-pflegerischen Versorgung für Versicherte sowie die Abrechnung und die Vergütung dieser Leistungen.

## § 2
## Geltungsbereich

(1) Dieser Vertrag gilt für den Träger und dem von ihm nachfolgend genannten Leistungserbringer mit folgender Adresse:
- Name:
- Straße:
- Ort:

(2) Dieser Vertrag gilt für die vertragsschließenden Krankenkassen sowie die Betriebskrankenkassen, die sich mit diesem Vertrag gegenüber dem Landes-verband der Betriebskrankenkassen in NRW einverstanden erklären.

## § 3
## Organisatorische Voraussetzungen

(1) Der Leistungserbringer hat durch geeignete organisatorische Maßnahmen unter Einsatz entsprechender personeller und sachlicher Mittel für die Dauer dieses Vertrages sicherzustellen, dass die von ihm betriebene Pflegeeinrichtung die Gewähr für eine leistungsfähige und wirtschaftliche palliativpflegerische Versorgung bietet. Hierzu gehört insbesondere, dass die Pflegeeinrichtung

(a) die von diesem Vertrag erfassten Leistungen umfassend erbringt,

(b) unter ständiger Verantwortung einer ausgebildeten Pflegefachkraft steht, die die Voraussetzungen nach § 4 Abs. 1 und 3 erfüllt,

(c) eine qualitativ ausreichende, gleichmäßige und konstante pflegerische Versorgung bei den von ihr betreuten Patienten gewährleistet,

(d) eine kontinuierliche palliativpflegerische Versorgung der Versicherten mit einer kalendertäglichen „rund um die Uhr“ Versorgung durch Pflegefachkräfte (§ 4 Abs. 1) sicherstellt

(e) die Ehrenamtlichen entsprechend den Vorgaben dieses Vertrages einbindet,

(f) mit einer qualifizierten Palliativärztin oder einem qualifizierten Palliativarzt (QPA) - entsprechend den Vorgaben des Rahmenprogramms zur flächen-deckenden Versorgung der ambulanten palliativmedizinischen und palliativ-pflegerischen Versorgung in NRW vom 19.05.2005 - zusammenarbeitet, *)

(g) mit dem behandelnden Hausarzt bzw. Facharzt, der hausärztliche Funktionen übernimmt, zusammenarbeitet,

(h) mit sonstigen Beteiligten (z. B. Krankenhäuser, stat. Hospize, Altenpflegeeinrichtungen, Apotheken, dem ärztlichen Notdienst, Palliativstationen) kooperiert.

(2) Die Pflegeeinrichtung muss über eigene Geschäftsräume verfügen.

***) Protokollnotiz**

## § 4
## Personelle Voraussetzungen

(1) Leistungen nach diesem Vertrag dürfen nur durch geeignete Pflegefachkräfte erbracht werden. Geeignete Pflegefachkräfte im Sinne dieses Vertrages sind Personen, die die Erlaubnis zur Führung einer der Berufsbezeichnungen Krankenschwester, Krankenpfleger, Gesundheits- und Krankenpfleger/in, 3-jährig examinierte Altenpfleger/in, Kinderkrankenschwester, Kinderkrankenpfleger oder Gesundheits- und Kinderkrankenpfleger/in besitzen.
Diese müssen den Abschluss einer 160 Stunden umfassenden Palliativ-Care-Weiterbildung nachweisen.*****)

(2) Im Palliativpflegebereich müssen mindestens 4 Vollzeitstellen mit Pflegefachkräften nach Abs. 1 besetzt werden, die für die Beschäftigung beim Leistungserbringer (§2) einen Arbeitsvertrag mit dem Träger abgeschlossen haben. Davon müssen mindestens 2 Pflegefachkräfte sozialversicherungspflichtig in Vollzeit beschäftigt sein. Die verbleibenden 2 Vollzeitstellen können auch durch eine ent-sprechende Anzahl von sozialversicherungspflichtigen Teilzeitbeschäftigten mit einer wöchentlichen Arbeitszeit von mindestens 18 Stunden besetzt werden. Inhaber oder Gesellschafter, die hauptberuflich im Palliativbereich mitarbeiten und dreijährig examiniert sind, sind Vollzeitbeschäftigten gleichgestellt. *****)

(3) Die Funktion der leitenden Pflegefachkraft muss von einer gemäß Abs. 1 qualifizierten Pflegefachkraft in Vollzeitbeschäftigung wahrgenommen werden. Der Abschluss einer 160 Stunden umfassenden Palliativ-Care-Weiterbildung ist für leitende Pflegefachkräfte nicht erforderlich, sofern der Leistungserbringer über eine Palliativ-Care-Einheit mit einer nach Abs. 1 qualifizierten Teamleitung verfügt.
Die leitende Pflegefachkraft muss nach abgeschlossener Berufsausbildung in dem erlernten Beruf zwei Jahre berufspraktische Erfahrung innerhalb der letzten fünf Jahre nachweisen. Ferner muss die leitende Pflegefachkraft den Abschluss einer Weiterbildungsmaßnahme für leitende Funktionen mit einer Mindeststundenzahl von 460 Stunden nachweisen.

Ein abgeschlossenes Studium im Fachbereich Pflege/Pflegemanagement an einer Fach-/Hochschule ersetzt die Weiterbildung. *****)

(4) Die Rahmenfrist nach Abs. 3 Satz 2 beginnt fünf Jahre vor dem Tag, zu dem die verantwortliche Pflegefachkraft bestellt werden soll. Die Rahmenfrist darf in keinem Fall acht Jahre überschreiten. Bei ausgeübter Teilzeitbeschäftigung(mindestens in einer Halbtagsbeschäftigung) verlängert sich die qualifizierende Beschäftigungszeit (nicht die Rahmenfrist) entsprechend.

Die Rahmenfrist verlängert sich um Zeiten, in denen die Fachkraft

a) wegen der Betreuung oder Erziehung eines Kindes nicht erwerbstätig war,

b) als Pflegeperson nach § 19 SGB XI eine pflegebedürftige Person wenigstens 14 Stunden wöchentlich gepflegt hat oder

c) an einem betriebswirtschaftlichen oder pflegewissenschaftlichen Studium oder einem sonstigen Weiterbildungslehrgang in der Kranken-, Alten- oder Heilerziehungspflege teilgenommen hat, soweit der Studien- oder Lehrgang mit einem nach Bundes- oder Landesrecht anerkannten Abschluss beendet worden ist.

(5) Als Stellvertretung der leitenden Pflegefachkraft ist mindestens halbtags eine Pflegefachkraft zu beschäftigen, die die Voraussetzungen des Absatzes 1 (incl. der Berufserfahrung nach Abs. 3 und 4) erfüllt. Im Falle der Abwesenheitsvertretung der leitenden Pflegefachkraft ist eine Vollzeitbesetzung durch die Stellvertretung sicherzustellen.

(6) Ein Unterschreiten der personellen Mindestbesetzung für die Dauer von mehr als drei Monaten ist der zuständigen Stelle unverzüglich anzuzeigen. Der zuständigen Stelle sind auf Verlangen unverzüglich die Qualifikations- und Sozialversicherungsnachweise für das eingesetzte Personal sowie die

Handzeichenliste nach Anlage 2 zuzusenden.

***) Protokollnotiz**

(7) Dem Leistungserbringer müssen mindestens fünfzehn geschulte Ehrenamtliche (vgl. § 7) aus den nach § 39 a SGB V geförderten Hospizdiensten zur Verfügung stehen. Die Schulung dieser Ehrenamtlichen umfasst mindestens 100 Unterrichtsstunden. Die Schulung hat nach den Vorgaben der Bundesarbeitsgemeinschaft Hospiz zur Förderung von ambulanten, teilstationären und stationären Hospizen und Palliativmedizin e. V. zu erfolgen.

## § 5
## Nachweis der personellen und organisatorischen Voraussetzungen

(1) Zum Nachweis der organisatorischen und personellen Voraussetzungen hat der Träger der zuständigen Stelle zur Überprüfung, ob die Voraussetzungen zum Abschluss dieses Vertrages erfüllt werden, die nachfolgenden Unterlagen einzureichen:

(a) Bekanntgabe der Stammdaten des Trägers und des Leistungserbringers sowie Nachweis des Vorliegens der organisatorischen und personellen Voraussetzungen zum Vertragsabschluss (Anlage 1),

(b) Nachweis der aktuell beschäftigten Mitarbeiter sowie die Qualifikationsnachweise des eingesetzten Personals incl. Nachweis zur Dokumentation der Unterschriften/Handzeichen (Anlage 2),

(c) einen aktuellen Auszug aus dem Bundeszentralregister der Generalbundesanwaltschaft für die verantwortliche Pflegefachkraft und ihre(n) Stellvertreter(in). Der Auszug darf nicht älter als drei Monate sein.

(d) Nachweis über die Mitgliedschaft bei der zuständigen Berufsgenossenschaft,

(e) Nachweis über das Bestehen einer ausreichenden Versicherung für Personen-, Sach- und Vermögensschäden in Höhe von mindestens

1,5 Mio. € sowie einer erweiterten Betriebshaftpflichtversicherung für etwaige Behandlungs-/Pflegefehler,

(f) Nachweis eines Institutionskennzeichens (IK)

(g) Nachweise der Berufserfahrung für die leitende und stellvertretende leitende Pflegefachkraft,

(h) bei juristischen Personen den aktuellen Auszug aus dem Handelsregister

(i) Bestätigung des zuständigen Gesundheitsamtes, aus der hervorgeht, dass die Aufnahme der Tätigkeit dort angezeigt wurde,

(j) Unterlagen zu einer auf den Leistungserbringer abgestimmten Strukturqualität mit dem Schwerpunkt ambulante palliativpflegerische Versorgung. Dazu gehören:

- Pflegeleitbild (§ 12),
- Pflegekonzept (§ 13),
- Muster einer Pflegedokumentation (§ 18),
- Stellenbeschreibungen (§ 14),
- Einarbeitungs-, Fort- und Weiterbildungskonzept (§§ 14 und 15) sowie ein
- Dienst- und Einsatzplankonzept (§ 16),

(k) eine schriftliche Bestätigung, dass die Unterlagen nach Buchstabe j) im Dienst umgesetzt und weiterentwickelt werden,

(l) Muster des Pflegevertrages (§ 29),

(m) Nachweis von mindestens fünfzehn geschulten Ehrenamtlichen nach § 4 Abs. 7, incl. Nachweis zur Dokumentation der Unterschriften/Handzeichen (Anlage 2a)**,**

(n) Nachweis der Zusammenarbeit mit einem/r qualifizierten Palliativarzt/-ärztin nach § 3 Buchstabe f),

(o) Kooperationsvertrag/-verträge entsprechend § 7.

(2) Die Unterlagen nach Abs. 1 bilden die wesentliche Grundlage dieses Vertrages.

Auf der Grundlage aller von dem Träger gemäß dem Absatz 1 abgegebenen Nachweise und vorgelegten Unterlagen (vgl. auch Abs. 6) entscheiden die Krankenkassen darüber, ob der betroffene Träger für seinen Leistungserbringer die Voraussetzungen zum Abschluss dieses Vertrages erfüllt. *****)

(3) Sofern alle Voraussetzungen des Vertrages erfüllt sind, wird der Vertrag mit dem Zeitpunkt wirksam, der im Bescheid der zuständigen Stelle an den Träger genannt wurde. Eine vorherige Leistungserbringung und Abrechnung ist ausgeschlossen.

(4) Der Träger verpflichtet sich, wesentliche Änderungen, insbesondere den Wegfall einer oder mehrerer Voraussetzungen nach Absatz 1 der zuständigen Stelle unverzüglich mitzuteilen. Sofern durch eine Änderung eine oder mehrere Voraussetzungen nach den §§ 3, 4 und 7 nicht mehr vollständig erfüllt werden, findet § 32 Abs. 2 Anwendung.

(5) Während der Laufzeit des Vertrages ist der Träger verpflichtet, der zuständigen Stelle im Rahmen der Anzeige nach § 6 unaufgefordert für jeden neu angestellten Mitarbeiter die hierzu einschlägigen Unterlagen gemäß § 4 sowie eine aktualisierte Nachweisliste gemäß § 5 Abs. 1 Buchst. a) einmal jährlich vorzulegen sowie auf Verlangen der zuständigen Stelle einen aktuellen Nachweis nach Maßgabe des Absatzes 1 zu erbringen.

(6) Die zuständige Stelle ist berechtigt, bei begründetem Anlass zum Nachweis der organisatorischen und personellen Voraussetzungen weitere geeignete Unterlagen zu verlangen.

(7) Alle nach diesem Vertrag beizubringenden Unterlagen sind im Original oder als Kopien einzureichen.

(8) Für räumlich getrennte Filialen, Außen- und Nebenstellen oder andere Stellen, von denen aus Pflege erbracht wird, ist ein gesonderter Vertrag erforderlich, auch dann, wenn diese zwar organisatorisch mit dem Pflegedienst verbunden, rechtlich aber nicht selbstständig sind.

***) Protokollnotiz**

## § 6
## Anzeigepflichten/Mitteilungspflichten

(1) Der Träger ist grundsätzlich verpflichtet, der zuständigen Stelle alle wesentlichen Umstände unverzüglich schriftlich mitzuteilen, die die vertragsgemäße Durchführung dieses Vertrages sowie dessen Fortbestand berühren. Unverzüglich anzuzeigen sind hiernach insbesondere:

(a) der Ausfall der leitenden Pflegefachkraft oder deren Stellvertretung (§ 4 Abs. 3 und 5) für einen drei Monate überschreitenden Zeitraum,

(b) der Wechsel der leitenden oder stellvertretenden leitenden Pflegefachkraft,

(c) jede Änderung der betrieblichen Gegebenheiten im organisatorischen Bereich (§ 3) und/oder im personellen Bereich (§ 4),

(d) der Abschluss, die Kündigung bzw. Änderungen von Kooperationsvereinbarungen,

(e) jede Änderung der Trägerschaft/Betriebsinhaberschaft sowie Rechtsform des Leistungserbringers,

(f) die Einstellung des Geschäftsbetriebes,

(g) die Eröffnung eines gegen den Geschäftsbetrieb gerichtetes Insolvenzverfahrens,

(h) die Unterschreitung der Mindestzahl von fünfzehn Ehrenamtlichen (§ 4 Abs. 7) über einen Zeitraum von mindestens 3 Monate,

(i) Wegfall der Kooperation mit einem/r qualifizierten Palliativarzt/-ärztin (§ 3 Abs. 1 Buchstabe g)) für länger als vier Wochen,

(j) polizeiliche oder staatsanwaltliche Ermittlungsverfahren.

(2) Sofern durch eine Änderung die Voraussetzungen nach § 3 und/oder § 4 nicht mehr vollständig erfüllt werden, findet § 32 Abs. 2 Anwendung.

## § 7
## Kooperation / Sicherstellung der Ehrenamtlichen

(1) Die Sicherstellung der fünfzehn Ehrenamtlichen hat durch Kooperation mit ambulanten Hospizdiensten, die nach § 39 a SGB V gefördert werden, zu erfolgen. Es ist eine schriftliche Kooperationsvereinbarung mit folgenden Mindestinhalten abzuschließen:

(a) Erklärung des/der ambulanten Hospizdienste(s), dass bei Kooperation dem Leistungserbringer ständig 15 Ehrenamtliche entsprechend den Vorgaben des § 4 Abs. 7 dieses Vertrages dem Leistungserbringer zur Verfügung stehen.

(b) Sofern Ehrenamtliche Leistungen nach dem SGB V / XI erbringen, sind diese nicht abrechnungsfähig.

(c) Kooperationen mit Ausnahme von Kooperationen nach Buchstabe a) und § 3 Abs. 1 Buchstabe f), die ihrem Zweck nach dazu dienen, fehlende organisatorische oder personelle Voraussetzungen zu

ersetzen, sind unzulässig.

(d) Kooperationen bedürfen der Zustimmung der Kostenträger.

## § 8
## Inhalt der Leistungen

Die Leistungen der ambulanten palliativpflegerischen Versorgung nach diesem Vertrag sind in der Anlage 3 beschrieben.

## § 9
## Materialien und Geräte

Der Träger hat seinen Pflegekräften im erforderlichen Umfang Arbeitsmittel (z. B. Einmalhandschuhe, Händedesinfektionsmittel) bereitzustellen, um eine qualifizierte, bedarfsgerechte und wirtschaftliche Versorgung zu gewährleisten.

Alle in der ambulanten palliativpflegerischen Versorgung eingesetzten Materialien und Geräte müssen fachlich geeignet und funktionsfähig sein sowie den Sicherheits- und anderen gesetzlichen Bestimmungen entsprechen. Mit den in der Anlage 6 genannten Vergütungen sind sämtliche, im Zusammenhang mit den im Rahmen dieses Vertrages erbrachten Verrichtungen, erforderlichen Aufwendungen abgegolten. Dies gilt auch für die Wartung/Sterilisation der in der ambulanten palliativpflegerischen Versorgung verwendeten Geräte. Ausgenommen hiervon sind verordnungsfähige Arzneien, Verband- und Hilfsmittel, die für den Versicherten verordnet und mit der Krankenkasse abgerechnet wurden.

## § 10
## Grundsätze der Leistungserbringung

(1) Der Träger hat darauf zu achten, dass die Leistungen wirksam und wirtschaftlich erbracht und nur im notwendigen Umfang in Anspruch genommen werden (§ 2 Abs. 4 SGB V).

(2) Die Leistungen müssen ausreichend, zweckmäßig und wirtschaftlich sein; sie dürfen das Maß des Notwendigen nicht überschreiten. Leistungen, die nicht notwendig oder unwirtschaftlich sind, können Versicherte nicht beanspruchen, darf der Träger nicht bewirken und die Krankenkasse nicht bewilligen (§ 12 Abs. 1 SGB V).

(3) Im Interesse der Wirtschaftlichkeit sind verordnete Leistungen der ambulanten palliativpflegerischen Versorgung sowie Leistungen aus der Pflegeversicherung, soweit möglich und sinnvoll, innerhalb eines Einsatzes zu erbringen.

(4) Der Träger gewährleistet, dass die Versicherten aller Krankenkassen nach gleichen Grundsätzen behandelt werden.

(5) Zur Optimierung der Versorgung mit Leistungen, insbesondere um einen reibungslosen und unverzüglichen Übergang von der stationären Krankenhausbehandlung zur ambulanten palliativpflegerischen Versorgung zu gewährleisten, hat der Leistungserbringer mit

(a) Vertragsärzten,

(b) ambulanten Hospizdiensten,

(c) stationären Hospizen,

(d) Vertragskrankenhäusern,

(e) Rehabilitationseinrichtungen,

(f) und sonstigen innerhalb des Versorgungsspektrums Beteiligten

(g) zusammenzuarbeiten.

Die Vereinbarungen nach § 3 Pflegegesetz NW gelten entsprechend.

(6) Der Leistungserbringer gewährleistet, dass zur Sicherstellung einer fach- und sachgerechten ambulanten palliativpflegerischen Versorgung nach diesem Vertrag fachlich qualifiziertes Personal eingesetzt wird und eine fach- und sach-gerechte ambulante palliativpflegerische Versorgung erfolgt; §§ 11 ff. des Vertrages sind zu beachten.

(7) Der Träger trägt die fachliche Verantwortung für das eingesetzte Personal. Über den Einsatz des Personals im Einzelfall entscheidet der Träger unter Beachtung der medizinischen Notwendigkeit sowie Art und Schwere der Erkrankung des Versicherten im Rahmen des Wirtschaftlichkeitsgebotes. Die Erbringung der vertraglichen Leistungen hat mit möglichst geringem Wechsel des Personals zu erfolgen.

## § 11
## Qualitätssicherung

(1) Der Leistungserbringer hat die Leistungen sorgfältig und einwandfrei auszuführen. Qualität und Wirksamkeit haben dem allgemein anerkannten Stand der medizinischen und pflegerischen Erkenntnisse zu entsprechen und den medizinischen Fortschritt zu berücksichtigen.

(2) Die Gemeinsamen Grundsätze und Maßstäbe zur Qualität und Qualitätssicherung einschließlich des Verfahrens zur Durchführung von Qualitätsprüfungen nach § 80 SGB XI in der ambulanten Pflege vom 10.07.1995 (vgl. Anlage 4) sind in der jeweils gültigen Fassung Bestandteil dieses Vertrages, soweit dieser Vertrag nichts anderes vorsieht.

## § 12
## Pflegeleitbild

(1) Der Leistungserbringer hat ein Pflegeleitbild mit einem palliativpflegerischen Schwerpunkt, das auf einem definierten Pflegemodell basiert. Dieses Pflegeleitbild macht Aussagen

(a) zum Pflege- und Betreuungsverständnis des Pflegedienstes,

(b) zur Rolle des Pflegebedürftigen und seiner Angehörigen,

(c) zur Qualifikation und Führung der Mitarbeiter,

(d) zur Einbindung der ehrenamtlichen Helfer,

(e) zur Kooperation mit anderen Leistungsanbietern,

(f) zum Qualitätsmanagement und

(g) zum Revisionsdatum.

(2) Die palliativpflegerische Arbeitsorganisation basiert auf einem System der Bezugspflege, das die Kontinuität der Pflege sowie eine patientenorientierte ganzheitliche Betreuung unterstützt.

## § 13
## Pflegekonzept

(1) Der Leistungserbringer hat ein Pflegekonzept mit einem palliativpflegerischen Schwerpunkt, in dem er Pflegestandards definiert. In diesen Standards sind alle qualitätsrelevanten Maßnahmen, Verfahren und Prozesse formuliert. Sie beinhalten mindestens

(a) deutlich formulierte Zielsetzungen,

(b) das zugrunde liegende Pflegeverständnis,

(c) Angaben über die erforderliche Qualifikation des Pflegenden,

(d) messbare Struktur-, Prozess- und Ergebniskriterien,

(e) den Namen der den Standard in Kraft setzenden Person und das Revisionsdatum.

(2) Das Pflegekonzept hat sich am Leistungsspektrum und palliativpflegefachlichen Schwerpunkt des Leistungserbringers zu orientieren. Dieses Leistungsspektrum und der palliativpflegefachliche Schwerpunkt sind vom Leistungserbringer zu definieren.

(3) Das Pflegekonzept hat einen Teil Qualitätsmanagement zu beinhalten, für das folgende Rahmenbedingungen unter Berücksichtigung des Rahmenprogrammes zur flächendeckenden Umsetzung der ambulanten palliativmedizinischen und palliativpflegerischen Versorgung in NRW vom 19.05.2005 gelten:

(a) Maßnahmen zur Sicherung der internen Struktur- Prozess- und Ergebnisqualität werden systematisch geplant, durchgeführt und überprüft. Zusätzlich beteiligt sich der Leistungserbringer an Maßnahmen der externen Qualitätssicherung.

(b) Interne Maßnahmen der Qualitätssicherung sind u. a. die Einsetzung eines Qualitätsbeauftragten, die Einrichtung eines Qualitätszirkels und die Entwicklung und Weiterentwicklung von Organisations- und Pflegestandards.

(c) Externe Maßnahmen der Qualitätssicherung können u. a. die Mitwirkung an Qualitätskonferenzen und Assessmentrunden sein.

(d) Die Durchführung von internen Qualitätsmaßnahmen und die Beteiligung an externen Qualitätssicherungsprojekten werden dokumentiert.

(e) Weiterer Bestandteil des Pflegekonzeptes ist die Einbindung aller in der Palliativpflege Tätigen (§ 4 Abs. 1) sowie die Regelungen zur Information und Kommunikation. Hier gelten folgende Rahmenbedingungen:

- Besprechungen aller in der ambulanten Palliativpflege Tätigen (§ 4 Abs. 1) finden im regelmäßigen Abstand, mindestens einmal monatlich, statt. Die Ergebnisse der Besprechungen werden incl. Teilnehmerliste protokolliert und für alle zugänglich aufbewahrt und bekannt gegeben.

- Alle pflegerelevanten Informationen werden neben der mündlichen Weitergabe auch in der Pflegedokumentation festgehalten.

- Fallbesprechungen werden innerhalb des Teams des Leistungserbringers in einem festen Turnus, mindestens alle zwei Wochen, durchgeführt und protokolliert.

## § 14
## Stellenbeschreibungen / Einarbeitungskonzept

(1) Der Leistungserbringer verfügt über Stellenbeschreibungen, in der die Verantwortungs- und Aufgabenbereiche der Mitarbeiter definiert sind.

(2) Die Einarbeitung der Mitarbeiter erfolgt auf der Grundlage eines schriftlich fixierten Einarbeitungskonzeptes.

## § 15
## Fort- und Weiterbildung

(1) Der Leistungserbringer hat ein Fort- und Weiterbildungskonzept und ist gesetzlich verpflichtet, Fortbildungen für seine Mitarbeiter/innen durchzuführen. Die Fortbildung ist nach den Rahmenempfehlungen nach § 132 a Abs. 1 SGB V auszurichten und der zuständigen Stelle auf Verlangen unter Fristsetzung nachzuweisen. Die Frist soll mindestens einen Monat betragen.

(2) Solange die o. a. Rahmenempfehlungen nicht vorliegen, sind die Inhalte der Fortbildungen so auszurichten, dass die Mitarbeiter/innen auf dem aktuellen Stand der allgemein anerkannten pflegewissenschaftlichen Erkenntnisse sind. Leitungskräfte (Pflegedienstleitung, Stellvertretung, Teamleitung, Qualitätsbeauftragter, etc.) des ambulanten Palliativdienstes sind verpflichtet, externe fach-bezogene Fortbildungen oder Inhouse-Schulungen von kalenderjährlich insgesamt 28 Stunden nachzuweisen. Hierbei ist darauf zu achten, dass diese Fortbildungen die Bereiche Dokumentation, Management und Pflege abdecken. Für die übrigen Mitarbeiter/innen sind regelmäßige Fortbildungen, die sich an den Inhalten des Satzes 1 orientieren sollten, durchzuführen. Als Nachweis dienen die entsprechenden Zertifikate bzw. Kopien der Veranstaltungsprotokolle (Teilnehmerliste).

(3) Werden Fortbildungen nicht fristgerecht nachgewiesen, werden vom Folgemonat an die Vergütungssätze nach Maßgabe des § 22 Abs. 4 gekürzt. Dem Leistungserbringer ist eine Frist zu setzen, innerhalb derer er die

Fortbildung nachholen kann. Als Frist hierfür können längstens sechs Monate vorgesehen werden. Kommt der Leistungserbringer innerhalb dieser Frist seiner Nachweispflicht nicht nach, gilt § 32 Abs. 1 Buchstabe g).

(4) Wird die Fortbildung vor Beendigung der Frist nachgewiesen, entfällt vom nächsten Monatsersten an die Kürzung der Vergütung.

## § 16
## Dienst-/Einsatzplankonzept

(1) Der Leistungserbringer hat ein Dienst-/Einsatzplankonzept zu erstellen und vorzuhalten, das in Abstimmung mit den Einsätzen der Ehrenamtlichen aufgestellt wird.

(2) Der Einsatzplan berücksichtigt die Wünsche und Bedürfnisse des Patienten und stellt eine größtmögliche Pflegekontinuität durch - nach Möglichkeit - maximal 3 verschiedene Pflegekräfte je Pflegebedürftigen sicher.

## § 17
## Pflegeprozess

(1) Grundlage des pflegerischen Handelns ist der Pflegeprozess. Die sechs Schritte des Pflegeprozesses sind:

(a) Informationssammlung / Pflegeanamnese,

(b) Erfassung von Problemen und Ressourcen,

(c) Festlegung der Pflegeziele,

(d) Planung der Pflegemaßnahmen,

(e) Durchführung und Dokumentation der Pflege sowie

(f) Beurteilung der Wirkung der Pflege auf den Patienten.

(2) Die Patienten und ihre Bezugspersonen sollen in die verschiedenen Schritte des Pflegeprozesses mit einbezogen werden.

## § 18
## Pflegedokumentation

(1) Der Leistungserbringer führt für jeden Versicherten eine detaillierte und zeitnahe Pflegedokumentation entsprechend § 12 (Pflegeleitbild / Pflegemodell). In ihr spiegelt sich der Pflegeprozess (§ 17) wieder. Sie stellt die nahtlose und aktuelle Informationssammlung bei der Überprüfung der Versorgungsmöglichkeiten des Versicherten (Prüfung der Wirtschaftlichkeit und Leistungspflicht) durch die betroffene Krankenkasse bzw. bei der Überprüfung der Abrechnung der Leistungen durch den Medizinischen Dienst der Krankenversicherung sicher.

(2) Die Pflegedokumentation beinhaltet u. a.

(a) das Patientenstammblatt,

(b) die Pflegeanamnese,

(c) die Pflegeplanung,

(d) den Pflegebericht,

(e) den Überleitungsbogen,

(f) spezielle Dokumentationsbögen, wie z. B. Vitalzeichen, Wundversorgung, Lagerungs- und Trinkprotokolle usw. sowie

(g) den Nachweis über die Art der durchgeführten Leistungen, (z. B. Injektion, Medikamentengabe), incl. Uhrzeit (Beginn des Einsatzes).

(3) Das Patientenstammblatt enthält mindestens:

(a) unveränderliche Daten (Name, Geburtsdatum usw.),

(b) das Aufnahmedatum,

(c) die Angaben zu Angehörigen/Bezugspersonen/Ansprechpartner,

(d) Angaben zur Betreuung/Beschlüsse/Verfügungen,

(e) Versicherungsdaten, Kostenübernahmeregelungen, Pflegestufe nach SGB XI,

(f) Angaben zur medizinisch/therapeutischen Versorgungssituation

(g) sowie anderen an der Versorgung Beteiligter.

(4) Die Pflegeplanung beinhaltet:

(a) den Pflegebeginn (Datum),

(b) die diagnosebezogenen/pflegerelevanten Pflegeprobleme,

(c) die Ressourcen,

(d) die Pflegeziele,

(e) die geplanten Pflegemaßnahmen/Durchführungshinweise,

(f) die Evaluation, Datum und das Handzeichen der Pflegefachkraft.

(5) Jeder Pflegeeinsatz ist zu protokollieren. Zu vermerken sind:

(a) Datum, Einsatzbeginn,

(b) erbrachte Leistungen,

(c) Abweichungen von der Pflegeplanung incl. Begründung,

(d) Handzeichen der durchführenden Pflegefachkraft und, sofern fachpflegerisch notwendig, u. a. auch

(e) der Zustand des Patienten,

(f) die gemessenen Vitalwerte,

(g) Durchführung selbstaktivierender Pflege- bzw. von Pflegeschulungsmaßnahmen,

(h) Rückspracheerfordernis beim behandelnden Arzt und dem/r qualifizierten Palliativarzt/-ärztin (§ 3 Abs. 1 Buchstabe f))

(i) Pflege-/Hilfsmittelversorgung,

(j) beigefügte pflegerische und ggf. ärztliche Befundberichte,

(6) Die Dokumentationsunterlagen sind beim Versicherten zu führen.

(7) Alle Eintragungen sind mit dem Handzeichen der Pflegefachkraft deutlich lesbar zu versehen. Die Dokumentation ist 5 Jahre aufzubewahren.

(8) Die Dokumentation von Einsätzen Ehrenamtlicher erfolgt nach den Bestimmungen zur Rahmenvereinbarung gemäß § 39 a Abs. 2 SGB V.

(9) Auf begründetes Verlangen der Krankenkasse sendet der Leistungserbringer die angeforderten Auszüge aus der Pflegedokumentation zur Überprüfung der Wirtschaftlichkeit und/oder Leistungspflicht in Kopie dieser zu.

## § 19
## Leistungsnachweis

(1) Jede erbrachte Leistung ist unmittelbar nach ihrer Durchführung im Leistungsnachweis durch die ausführende Pflegefachkraft einzutragen und durch Handzeichen zu bestätigen. Maschinelle Eintragungen im Leistungsnachweis, die vor Erbringung der Leistung vorgetragen wurden, sind kenntlich zu streichen, falls keine Leistung erbracht wurde. Vordatierungen oder Globalbestätigungen über den Empfang von Leistungen sind unzulässig. Gleiches gilt für Eintragungen im Leistungsnachweis vor Erbringung der Leistung.

Eine Durchschrift des Leistungsnachweises hat beim Versicherten zu verbleiben. Der Leistungsnachweis ist nicht Bestandteil der Pflegedokumentation, sondern eine Rechnungsanlage, die folgende Mindestinhalte aufzuweisen hat:

(a) Krankenversichertennummer,

(b) Name und Vorname des Patienten,

(c) Geburtsdatum des Patienten,

(d) Tag der Leistungserbringung,

(e) Uhrzeit (Beginn) der Leistungserbringung,

(f) Art der Leistung,

(g) je Einsatz Handzeichen der leistungserbringenden Pflegefachkraft nach § 4 sowie einmal monatlich eine Bestätigung durch Unterschrift der

verantwortlichen Pflegedienstleitung bzw. Teamleitung der Palliativ-Care-Einheit,

(h) nach erfolgter Leistungserbringung einmal monatlich Datum und Unterschrift des Patienten, des gesetzlichen Vertreters oder Bevollmächtigten sowie

(i) Institutionskennzeichen, Name und Anschrift des Leistungserbringers.

(2) Die verwendeten Leistungsnachweise haben dem Muster nach Anlage 5 zu entsprechen.

## § 20
## Personenkreis

Der Personenkreis, der Anspruch auf ambulante palliativpflegerische Versorgung nach diesem Vertrag hat, umfasst unheilbar kranke Versicherte, deren Krankheit

(a) progredient verläuft und bereits so weit fortgeschritten ist, dass lediglich eine Lebenserwartung von Wochen oder wenigen Monaten zu erwarten ist und

(b) eine ambulante palliative Versorgung notwendig macht und vom Patienten erwünscht ist.

Die Notwendigkeit einer ambulanten palliativen Versorgung kann sich insbesondere bei folgenden Krankheitsbildern im Endstadium ergeben:

(c) fortgeschrittene Krebserkrankung,

(d) Vollbild der Infektionskrankheit AIDS,

(e) Erkrankung des Nervensystems mit unaufhaltsam fortschreitenden Lähmungen,

(f) Endzustand einer chronischen Nieren-, Leber-, Herz- oder Lungenerkrankung.

Kurative Behandlungen sind bei Palliativpatienten ausgeschöpft und nicht mehr angezeigt.

## § 21
## Abgabe der Leistungen/Kostenübernahme/Vergütungsanspruch

(1) Leistungen nach diesem Vertrag dürfen für den Personenkreis nach § 20 nur aufgrund vertragsärztlicher Verordnung (Muster 12) erbracht werden.

Die Kostenübernahme ist vor Beginn der ambulanten palliativpflegerischen Versorgung bei der zuständigen Krankenkasse mit der vertragsärztlichen Verordnung (ggf. per Fax) zu beantragen. Die Verordnung (Muster 12 a. 2/E) ist vorab vom Versicherten und dem Leistungserbringer auszufüllen. Der Leistungserbringer hat dabei detailliert die geplanten Leistungen sowie die Häufigkeit und Dauer einzutragen. Über die palliativpflegerische Versorgung und ihre Dauer (ca. 28 Tage) entscheidet die Krankenkasse unter Berücksichtigung der vertragsärztlichen Verordnung. Sollte die Versorgung für einen längeren Zeitraum erforderlich sein, ist rechtzeitig vor Ablauf ein Verlängerungsantrag unter Beifügung der vertragsärztlichen Verordnung bei der zuständigen Krankenkasse zu stellen, in dem die medizinische Notwendigkeit der Verlängerung begründet wird. Sofern aus Sicht des Leistungserbringers erforderlich, ist mit der vertragsärztlichen Verordnung ein Antrag auf Pflegeleistungen nach dem SGB XI einzureichen. Den jeweils aktuellen Schnellbegutachtungsbogen sendet der Leistungserbringer mit einer Kopie der vertragsärztlichen Verordnung an den zuständigen Medizinischen Dienst der Krankenversicherung. Für Versicherte der Knappschaft und der Betriebskrankenkassen wird der jeweils aktuelle Schnellbegutachtungsbogen mit der vertragsärztlichen Verordnung direkt an die zuständige Kasse gesandt,

sofern keine anderweitige Absprache getroffen wird.*)

(2) Sofern an Wochenenden und Feiertagen die erstmalige Kostenzusage für ambulante palliativpflegerische Versorgung durch die Krankenkasse nicht vor Beginn der ambulanten palliativpflegerischen Versorgung eingeholt werden kann, ist die Kostenübernahme unter Beifügung der vertragsärztlichen Verordnung innerhalb der nächsten drei Arbeitstage (ggf. per Fax) bei der Krankenkasse zu beantragen.

(3) Soweit die Krankenkasse eine Begrenzung/Einschränkung der Leistungsgewährung vorgibt, ist dies vom Leistungserbringer zu beachten. Ändern sich - ohne Auswirkung auf die Einsatzfrequenz - für den von der Krankenkasse bereits genehmigten Zeitraum die palliativpflegerischen Leistungen (vgl. Punkt 3 und 4 der Anlage 3) bzw. kommen Leistungen hinzu, ist für den bereits von der Krankenkasse genehmigten Zeitraum keine neue vertragsärztliche Verordnung erforderlich. Der Leistungserbringer hat in diesem Fall jedoch nachweislich sicherzustellen, dass er diese geänderte ärztliche Anweisung erhalten hat und umsetzt. Sollte die ambulante palliativpflegerische Versorgung für einen längeren Zeitraum erforderlich sein, ist rechtzeitig vor Ablauf der Befristung mittels der vertragsärztlichen Verordnung (Muster 12 der Vordruckvereinbarung Ärzte/Krankenkassen) ein Verlängerungsantrag bei der Krankenkasse zu stellen, um dieser Gelegenheit zu geben, ggf. den Medizinischen Dienst der Krankenversicherung einzuschalten. Auf Anforderung ist der Krankenkasse unter Berücksichtigung der datenschutzrechtlichen Bestimmungen eine Kopie der notwendigen Blätter der Pflegedokumentation (§ 8) nachzureichen. Zur Verbesserung der Transparenz hinsichtlich der erforderlichen Leistungen soll der Leistungserbringer ergänzende Angaben der Krankenkasse übermitteln oder auf deren Anforderung zusenden.

(4) Sofern der Leistungsanspruch nach § 37 Abs. 3 SGB V nicht ausgeschlossen ist**,** die vertragsärztliche Verordnung den vertraglichen Vereinbarungen entspricht und die Verordnung innerhalb von 3 Arbeitstagen eingereicht wurde, übernimmt die Krankenkasse die Kosten längstens bis zur Entscheidung über den Leistungsantrag; der Leistungserbringer nimmt insoweit seine sich aus §

10 Abs. 1 und 2 ergebende Mitwirkungspflicht wahr. Die zuständige Krankenkasse wird den Leistungserbringer unverzüglich über ihre Leistungspflicht informieren.

***) Protokollnotiz**

(5) Über Art, Umfang und Dauer der Leistungen entscheidet die Krankenkasse unter Berücksichtigung der vertragsärztlichen Verordnung. Art und Inhalt der Leistungen ergeben sich aus der als Anlage 3 beigefügten Leistungsbeschreibung sowie der Vergütungsvereinbarung nach Anlage 6. Die zuständige Krankenkasse entscheidet unverzüglich über den Leistungsantrag und informiert hierüber den Leistungserbringer. Soweit die vertraglich verordneten Leistungen nicht oder nur zum Teil übernommen werden, informiert die Krankenkasse den Versicherten und den Arzt.

(6) Bei Annahme einer vertragsärztlichen Verordnung über ambulante palliativpflegerische Versorgung ist die Gesamtheit der von der Krankenkasse genehmigten Leistungen zu erbringen.

(7) Die vertragsärztliche Verordnung ist nicht übertragbar; sie gilt nur für die Person, für die sie ausgestellt ist. Änderungen und Ergänzungen vertragsärztlicher Verordnungen bedürfen der Unterschrift des verordnenden Arztes.

## § 22
## Vergütung

(1) Die Vergütung der Vertragsleistungen richtet sich nach Anlage 6. Mit dieser Vergütung sind auch alle vertraglichen Haupt- und Nebenpflichten abgegolten.

(2) Die Vergütung der Vertragsleistungen

(a) bei der Versorgung von Versicherten in Wohnanlagen für Senioren bzw. im Rahmen des so genannten Betreuten Wohnens, soweit der Leistungserbringer regelmäßig mehr als vier Patienten versorgt,

(b) bei der Versorgung von Versicherten in Wohnheimen und Seniorenwohnungen, deren Träger bzw. Vermieter für die Bewohner bzw. Mieter zugleich ambulante Pflegeleistungen im Sinne dieses Vertrages anbieten,

(c) bei der Versorgung von Versicherten in Wohnheimen oder Seniorenwohnungen, in denen der ambulante Pflegedienst seinen Betriebssitz hat sowie

(d) bei der Versorgung von mehr als 4 Personen in einem Haus
(e) beträgt 80% der Regelvergütung.

(3) Der Vergütungsanspruch besteht nur für entsprechend den Vorgaben dieses Vertrages tatsächlich erbrachte und dokumentierte Vertragsleistungen. Für nicht vertragsmäßig erbrachte und auch für nicht dokumentierte Vertragsleistungen (§§ 18, 19) besteht weder ein Vergütungsanspruch gegenüber dem Kostenträger noch dem Versicherten.

(4) Kommt der Träger seiner Verpflichtung zum Nachweis der Fortbildung (§ 15) nicht nach, erfolgen Vergütungsabschläge für die nach diesem Vertrag zu vergütenden Leistungen in Höhe von 10 v. H. für die ersten drei Monate, danach von 25 v. H.

(5) Einsätze, die wegen fehlender Mitwirkung der Versicherten nicht erbracht werden können, sind nicht abrechnungsfähig. Gleiches gilt für Vertragsleistungen, die von hierzu nicht gemäß § 4 Abs. 1 des Vertrages berechtigtem Personal erbracht wurden.

(6) Mit den für die Vertragsleistungen gemäß Anlage 6 des Vertrages vereinbarten Vergütungssätzen sind zugleich sämtliche Aufwendungen des Trägers/Leistungserbringers abgegolten, die im unmittelbaren und mittelbaren Zusammenhang mit der jeweils vergüteten Vertragsleistung stehen.

(7) Dem Träger/Leistungserbringer sowie dessen Personal ist es untersagt, für Vertragsleistungen Zahlungen von dem Versicherten oder einem sonstigen Dritten zu fordern oder anzunehmen.

## § 23
## Abrechnung gemäß § 302 SGB V

(1) Mit Umsetzung der für den Bereich der häuslichen Krankenpflege gemäß § 302 Abs. 2 SGB V zum 01.06.1996 in Kraft getretenen Richtlinien der Spitzenverbände der Krankenkassen über Form und Inhalt des Abrechnungsverfahrens mit "Sonstigen Leistungserbringern" werden die dortigen Regelungen (in der jeweils aktuellen Fassung) ohne weitere vertragliche Absprache für den Träger unmittelbar verbindlich und ersetzen die hierzu für das Abrechnungsverfahren nach-folgend in diesem Vertrag vorläufig getroffenen Vereinbarungen nach § 23 a.

(2) Über den Zeitpunkt der Umsetzung der Richtlinien der Spitzenverbände der Krankenkassen gemäß § 302 Abs. 2 SGB V wird die jeweilige Krankenkasse den Träger rechtzeitig informieren. Auf der Vorderseite der Leistungsnachweise sind rechts oben die Belegnummern aus der Gesamtrechnung sowie die Rechnungsnummer anzugeben. Die Leistungsnachweise sind nach Belegnummern aufsteigend sortiert anzuliefern. Dies gilt auch für Kopien der vertragsärztlichen Verordnung einschließlich Genehmigung der Krankenkasse, soweit nicht darauf verzichtet wird.

(3) Die nach diesem Vertrag zu vergütenden Leistungen sind jeweils im Folgemonat mit der Krankenkasse abzurechnen. Abgerechnet werden dürfen nur genehmigte, tatsächlich erbrachte und dokumentierte Leistungen (§§ 18 und 19). Der Leistungsnachweis (§ 19) sowie die Genehmigung der

Krankenkasse – soweit nicht darauf verzichtet wird – sind der Abrechnung beizufügen.

(4) Für jeden Leistungserbringer ist bei der Arbeitsgemeinschaft Institutionskennzeichen (SVI) beim Hauptverband der Gewerblichen Berufsgenossenschaften e. V., Alte Heerstraße 111, 53757 St. Augustin, ein Institutionskennzeichen (IK) zu beantragen. Das IK des Leistungserbringers ist im Rahmen der Abrechnung zu verwenden. Dies gilt auch, wenn die Abrechnung über eine Abrechnungsstelle/Verrechnungsstelle erfolgt.

(5) Die Abrechnungsdaten der Abrechnungsfälle umfassen insbesondere:

(a) Institutionskennzeichen (IK) der Krankenkasse,

(b) Krankenversicherten-Nr.,

(c) Versichertenstatus (Anlage 3 Abschnitt 8.1.1 der Richtlinien),

(d) Vertragsarzt-Nr.,

(e) Datum der Verordnung,

(f) Unfallkennzeichen (Anlage 3 Abschnitt 8.1.2 der Richtlinien),

(g) Kennzeichen BVG/Sonstiges (Anlage 3 Abschnitt 8.1.2.1 der Richtlinien),

(h) Rechnungsnummer mit eindeutiger Belegnummer innerhalb einer Gesamtrechnung,

(i) Genehmigungskennzeichen der jeweiligen Krankenkasse (soweit vorhanden),

(j) Datum der Genehmigung (im Format JJJJMMTT),

(k) Institutionskennzeichen des Leistungserbringers/Rechnungserstellers,

(l) Leistungserbringergruppe, bestehend aus Abrechnungscode und Tarifkennzeichen der jeweiligen Krankenkasse,

(m) Art der abgegebenen Leistung,

(n) Einzelbetrag der Leistung,

(o) Datum/Daten der Leistungserbringung,

(p) Einsatzzeit (Beginn).

(6) Leistungen gemäß § 37 Abs. 1 SGB V und Leistungen gemäß § 37 Abs. 2 SGB V sind in getrennten Dateien abzurechnen.

(7) Für Anspruchsberechtigte nach dem Bundesvertriebenengesetz (BVFG), dem Bundesentschädigungsgesetz (BEG), dem Bundesversorgungsgesetz (BVG), dem Häftlingshilfegesetz (HHG), dem Opferentschädigungsgesetz (OEG), dem Bundesseuchengesetz (BseuchG), dem Soldatenversorgungsgesetz (SVG) sowie Personen, die nach zwischenstaatlichem Krankenversicherungsrecht im Auftrag ausländischer Krankenversicherungsträger betreut werden, ist eine zusätzliche Einzelrechnung in Papierform für die Krankenkasse zu erstellen.

(8) Forderungen der Krankenkasse gegen den Träger können gegenüber demselben aufgerechnet werden.

(9) Rechnungen, die den Anforderungen nach den Absätzen 1 bis 7 nicht entsprechen, können von der Krankenkasse berichtigt oder unter Angabe von Gründen zur Berichtigung innerhalb von 20 Arbeitstagen nach Eingang zurückgegeben werden.

## § 23a
## Abrechnung vor Umsetzung des § 302 SGB V

(1) Bis zum Zeitpunkt der Umsetzung der Richtlinien der Spitzenverbände der Krankenkassen gemäß § 302 Abs. 2 SGB V erfolgt die Rechnungslegung entsprechend den Bestimmungen der nachfolgenden Absätze.

(2) Die nach diesem Vertrag zu vergütenden Leistungen sind vom Träger grundsätzlich im Folgemonat mit der Krankenkasse abzurechnen. Die Genehmigung der Krankenkasse - soweit hierauf nicht verzichtet wird - und der Leistungsnachweis (§ 19) sind beizufügen. Abgerechnet werden dürfen nur genehmigte, tatsächlich erbrachte und dokumentierte Leistungen (§§ 18 und 19).

(3) Das Rechnungsblatt muss folgende Mindestinhalte aufweisen:

(a) Krankenversichertennummer,

(b) Name und Vorname des Patienten,

(c) Geburtsdatum des Patienten,

(d) Anschrift des Patienten,

(e) Leistungszeitraum (von/bis),

(f) Art der Leistung (z. B. Injektion),

(g) Einzelpreis der Leistung,

(h) Anzahl der Leistung,

(i) Gesamtbetrag,

(j) Rechnungsnummer,

(k) Rechnungsdatum,

(l) Institutionskennzeichen des Leistungserbringers sowie

(m) Institutionskennzeichen des Zahlungsempfängers, sofern vom Leistungserbringer abweichend.

(4) Für den Abrechnungsverkehr ist das für den Leistungserbringer maßgebende Institutionskennzeichen (IK) zu verwenden, das von der Arbeitsgemeinschaft Institutionskennzeichen (SVI) beim Hauptverband der gewerblichen Berufsgenossenschaften e. V., Alte Heerstraße 111, 53757 St. Augustin, vergeben wird. Dies gilt auch, wenn die Abrechnung über eine Abrechnungsstelle/ Verrechnungsstelle erfolgt.

(5) Werden Leistungen für Versicherte sowohl mit der Pflegekasse, als auch mit der Krankenkasse abgerechnet, ist eine getrennte Rechnungslegung erforderlich.

(6) Forderungen der Krankenkasse gegen den Träger können gegenüber demselben aufgerechnet werden.

(7) Rechnungen, die den Anforderungen nach den Absätzen 1 bis 5 nicht entsprechen, können von der Krankenkasse berichtigt oder unter Angabe von Gründen zur Berichtigung innerhalb von 20 Arbeitstagen nach Eingang zurückgegeben werden.

## § 24
## Rechnungslegung durch Abrechnungsstellen

(1) Sofern die Abrechnung von dem Träger einer Abrechnungsstelle/ Verrechnungsstelle übertragen werden soll, hat der Träger unter besonderer Berücksichtigung der von ihr getroffenen technischen und organisatorischen Maßnahmen zur Sicherstellung der Maßgaben dieses Vertrages und der einschlägigen datenschutz-rechtlichen Bestimmungen die Abrechnungsstelle/Verrechnungsstelle auszuwählen.

(2) Der Träger ist verpflichtet, von allen Versicherten schriftliche Einwilligungserklärungen für die Datenweitergabe an die Abrechnungsstelle einzuholen. Die Einwilligungserklärungen sind der Krankenkasse und der Abrechnungsstelle auf Verlangen vorzulegen.

(3) Die Abtretung ist vier Wochen vorher anzuzeigen. Zahlungen an eine durch den Träger ermächtigte Abrechnungsstelle/Verrechnungsstelle setzen voraus, dass der zuständigen Krankenkasse der Vertrag und eine Ermächtigungserklärung des Leistungserbringers vorliegen. Eine weitere Ermächtigungserklärung setzt den Widerruf der zuvor erteilten Ermächtigungserklärung voraus.

(4) Der Träger verpflichtet sich, seine Forderungen gegen die Krankenkassen nicht an Dritte abzutreten, wenn und soweit die Krankenkassen gegen ihn Ansprüche wegen rückständiger Gesamtsozialversicherungsbeiträge, Überzahlungen (Rückforderungsansprüche) oder sonstiger Forderungen hat. Zur Sicherung derartiger Forderungen besteht zugunsten der Krankenkassen ein Abtretungsausschluss nach § 399 BGB. Die Vertragspartner gehen davon aus, dass die Krankenkassen aufgrund der Bestimmungen des § 354 a HGB auch im Fall einer verbotswidrigen Abtretung berechtigt sind, gegenüber dem Abrechnungszentrum die Zahlung zu verweigern und mit Ihren Ansprüchen gegen den Träger aufzurechnen.

(5) Zahlungen an eine Abrechnungsstelle/Verrechnungsstelle erfolgen mit schuldbefreiender Wirkung für die Krankenkasse, wenn die abrechnende Stelle Originalabrechnungsunterlagen einreicht, es sei denn, der zahlungspflichtigen Krankenkasse liegt bei Eingang der Originalabrechnungsunterlagen ein schriftlicher Widerruf des Leistungserbringers vor. Die schuldbefreiende Wirkung tritt auch dann ein, wenn die Rechtsbeziehung zwischen der Abrechnungsstelle/ Verrechnungsstelle und dem Träger mit einem Rechtsmangel behaftet ist. Schädigt die Abrechnungsstelle anlässlich der Abrechnung die Krankenkasse, so haften Träger und Abrechnungsstelle/Verrechnungsstelle gesamtschuldnerisch.

(6) Für Abrechnungsstellen/Verrechnungsstellen gelten die §§ 23 und 23 a entsprechend. Mit etwaigen Abrechnungsstellen/Verrechnungsstellen wird der Leistungserbringer diesbezügliche Absprachen treffen.

## § 25
## Zahlungsfrist /Beanstandung

(1) Bis zur Umsetzung der für den Bereich der häuslichen Krankenpflege gemäß § 302 Abs. 2 SGB V zum 01.06.1996 in Kraft getretenen Richtlinien der Spitzenverbände der Krankenkassen sind die Rechnungen - vorbehaltlich einer abschließenden Rechnungsprüfung - grundsätzlich innerhalb von 15 Arbeitstagen nach Eingang bei der zuständigen Krankenkasse bzw. dem genannten Rechenzentrum zu bezahlen. Bei Rechnungskürzungen gilt dies nur für den unstreitigen Rechnungsbetrag. Als Zahltag gilt der Tag der Überweisung oder der Tag der Übergabe des Überweisungsauftrages an ein Geldinstitut.

(2) Die Fälligkeit für die vertraglich geschuldete Vergütung tritt nach der gem. Abs. 1 vereinbarten Zahlungsfrist nur bei vorschriftsmäßiger Rechnungslegung (§§ 23/23 a und 24) ein.

## § 26
## Ausschlussfrist

Vertragsleistungen, für die entgegen der hierzu in § 23 Absatz 3 Satz 1 bzw. § 23 a Abs. 2 Satz 1 vereinbarten Frist eine Rechnungslegung verspätet erfolgt, können nur in begründeten Ausnahmefällen abgerechnet werden. Diese Ausnahmefälle sind nur abrechnungsfähig, wenn gegenüber der Krankenkasse innerhalb einer Ausschlussfrist von zwölf Monaten ab dem Zeitpunkt der Leistungserbringung eine Rechnungslegung erfolgt ist.

## § 27
## Verbot der Einflussnahme und Vermittlungsverbot

(1) Eine gezielte Beeinflussung der Versicherten und Ärzte, insbesondere hinsichtlich der Verordnung von Leistungen, ist unzulässig.

(2) Die Annahme von Pflegeaufträgen und deren Weitergabe (z. B. Vermittlung an Dritte gegen Entgelt oder zur Erlangung geldwerter Vorteile) ist unzulässig. Aus-genommen ist die unentgeltliche Vermittlung oder Weitergabe, wenn notwendige, aber selbst nicht ausführbare Einsätze dies erfordern.

## § 28
## Datenschutz / Schweigepflicht / geschäftsmäßige Rechtsberatung

(1) Personenbezogene Daten (Sozialdaten) darf der Leistungserbringer nur zur Erfüllung der sich aus diesem Vertrag ergebenden Aufgaben erheben, verarbeiten, bekannt geben, zugänglich machen oder sonst nutzen.

(2) Der Träger verpflichtet sich ferner, über alle bei der Durchführung des Vertrages erlangten Sozialdaten von Versicherten und sonstigen Dritten, insbesondere über medizinische und pflegerische Daten, Stillschweigen zu bewahren. Ausgenommen hiervon sind die zur Durchführung der Krankenversicherung erforderlichen Angaben gegenüber den behandelnden Ärzten, dem Medizinischen Dienst der Krankenversicherung und der

zuständigen Krankenkasse. Im Übrigen hat der Träger die Einhaltung der für ihn einschlägigen datenschutzrechtlichen Bestimmungen sicherzustellen.

(3) Der Träger ist verpflichtet, die Bestimmungen der Absätze 1 und 2 auch den ein-gesetzten Pflegekräften bekannt zu geben und deren Beachtung durch diese in geeigneter Weise (z. B. durch Hinweis im Arbeitsvertrag und/oder wiederkehrende Belehrungen) sicherzustellen.

(4) Eine gezielte Beeinflussung der Versicherten, Vertragsärzte und Krankenhäuser, insbesondere hinsichtlich der Verordnung von Leistungen, ist unzulässig. Dies gilt auch für die Rechtsberatung von Patienten/Versicherten.

## § 29
## Pflegevertrag

(1) Der Träger hat mit dem Versicherten einen schriftlichen Pflegevertrag zu schließen, in dem die Einzelheiten der Leistungserbringung, z. B. genaue Definition der Leistungen, entsprechend der Leistungsbeschreibung sowie Dauer und Menge je Kalendertag, vereinbart werden.

(2) Die Vorschriften des SGB V und die hierzu abgeschlossenen Vereinbarungen sowie die Entscheidung der Krankenkasse sind dem Vertrag zugrunde zu legen und maßgebend. Liegt die Entscheidung der Krankenkasse bei Vertragsabschluss noch nicht vor, ist der Pflegevertrag nach Bekanntwerden bzw. Rechtskraft anzupassen.

(3) Bei ordentlicher Kündigung ist eine längere Kündigungsfrist von mehr als 10 Tagen für den Versicherten unzulässig. Für den Leistungserbringer gilt eine Kündigungsfrist von mindestens 3 Wochen. Im Übrigen gilt § 120 SGB XI entsprechend.

## § 30
## Zahlung bei Feststellung von Qualitätsmängeln

(1) Grundlage für die Feststellung von Qualitätsmängeln ist der Maßnahmenkatalog des Medizinischen Dienstes der Krankenversicherung sowie die Stellungnahme des Leistungserbringers. Qualitätsmängel in diesem Sinne liegen vor, wenn

(a) das Pflegekonzept nach § 13 und/oder

(b) das Einarbeitungskonzept nach § 14 und/oder

(c) das Fort- und Weiterbildungskonzept nach § 15 und/oder

(d) das Dienst- und Einsatzplankonzept nach § 16
nicht den dort genannten Anforderungen entspricht und/oder
nicht umgesetzt wurde und/oder

(e) aus der im Rahmen der Qualitätsprüfung vorgelegten Pflegedokumentation nicht der Pflegeprozess (§ 17) erkennbar ist und/oder

(f) im Rahmen der Prüfung der Ergebnisqualität vom Medizinischen Dienst der Krankenversicherung festgestellt wurde, dass ein Patient zu Schaden (Körperverletzung) gekommen ist.

(2) Bei Feststellung von Qualitätsmängeln nach Abs. 1 Buchstabe a) bis e) kommen Maßnahmen nach § 31 Abs. 1 Buchstabe a) bis c) sowie bei Qualitätsmängeln nach Abs. 1 Buchstabe f) und wiederholten Qualitätsmängeln nach Abs. 1 Buchstabe a) bis e) Maßnahmen nach § 31 Abs. 1 Buchstabe c) und d) in Betracht.

## § 31
## Maßnahmen bei Gesetzes- bzw. Vertragsverstößen, Wiedergutmachung des Schadens

(1) Verletzt der Träger / Leistungserbringer seine gesetzlichen und/oder vertraglichen Pflichten, entscheiden die Krankenkassen gemeinsam oder einzeln nach Anhörung des betroffenen Trägers über geeignete Maßnahmen. Als solche kommen in Betracht:

(a) Auflagen,

(b) Verwarnung,

(c) Vertragsstrafe in Geld bis zu 15.000,00 €, unabhängig davon, wie viele Krankenkassen die Maßnahme verhängen,

(d) außerordentliche Kündigung des Vertrages nach § 32.

(2) Verstöße gegen die gesetzlichen und/oder vertraglichen Bestimmungen sind dem Vertragspartner schriftlich mitzuteilen. Ihm ist die Gelegenheit zu einer Stellungnahme innerhalb einer angemessenen Frist zu geben. Der Vertragspartner kann seinen Berufsverband hinzuziehen. Die Vertragspartner bemühen sich, eventuelle Meinungsverschiedenheiten über Vertragsverstöße gemeinsam mit dem Ziel einer gütlichen Einigung zu klären.

(3) Die Entscheidung wird dem Betroffenen schriftlich mitgeteilt.

(4) Unabhängig von den Maßnahmen nach Abs. 1 ist der durch die Vertragsverletzung verursachte Schaden durch den Träger zu ersetzen.

## § 32
## Außerordentliche Kündigung

(1) Der Vertrag kann von den vertragsschließenden Krankenkassen gemeinsam oder einzeln gegenüber dem Träger ohne Einhaltung einer Kündigungsfrist gekündigt werden, wenn der Träger seine gesetzlichen und/oder vertraglichen Pflichten gegenüber den Patienten oder der Krankenkasse derart gröblich verletzt, dass ein Festhalten an diesem Vertrag nicht zumutbar ist. Dies liegt insbesondere vor:

(a) bei wiederholten und/oder schwerwiegenden Verstößen gegen die vertraglichen und/oder gesetzlichen Bestimmungen,

(b) bei Abrechnung nicht und/oder teilweise nicht erbrachter Leistungen bzw. Abrechnung anderer als der erbrachten Leistungen und/oder bei wiederholter Abrechnung nicht dokumentierter Leistungen (§§ 18 und 19),

(c) bei Pflichtverletzungen, derentwegen Patienten zu Schaden kommen können bzw. gekommen sind,

(d) sofern wiederholt wesentliche Qualitätsmängel bei der Qualitätsprüfung fest-gestellt wurden (§ 30). Dabei ist unerheblich, ob die festgestellten Qualitätsmängel identisch sind oder nicht,

(e) bei einem Fehlverhalten des Leistungserbringers, das gem. § 74 SGB XI zur fristlosen Kündigung des mit ihm für den Bereich der Pflegeversicherung bestehenden Versorgungsvertrages berechtigt,

(f) bei Fehlen der Vertragsvoraussetzungen nach § 3 Abs. 1 Buchstaben b) und e), § 5 Abs. 4, § 6 Abs. 2,

(g) Das Vertragsverhältnis ist von den Krankenkassen ohne Einhaltung einer Kündigungsfrist zu kündigen, wenn der Träger des ambulanten

Palliativpflegedienstes innerhalb der Nachweisfrist (§ 15 Abs. 3) die fehlenden Fortbildungsmaßnahmen nicht nachholt,

(h) Forderung/Entgegennahme von Zahlungen für Vertragsleistungen von dem Versicherten oder einem sonstigen Dritten.

(2) Liegt eine oder mehrere der wesentlichen Vertragsvoraussetzungen (insbesondere nach den §§ 3, 4 und 7) nicht mehr vor, kann das Vertragsverhältnis von den Krankenkassen gemeinsam oder einzeln mit einer dreimonatigen Frist zum Ende eines Kalendermonats gekündigt werden.

(3) Eine außerordentliche Kündigung (§ 2) kann von den Krankenkassen gemeinsam oder einzeln ausgesprochen werden, wenn eine den Qualitätsanforderungen ent-sprechende Versorgung der Versicherten infolge innerbetrieblicher Gegebenheiten (z. B. Vorliegen von Pfändungen/Verrechnungen über die Höhe eines Monatsumsatzes der vertragsschließenden Krankenkassen hinaus) nicht zu erwarten ist.

## § 33
## Auswertung

(1) Der Leistungserbringer ist verpflichtet, an Auswertungen mitzuwirken, die im Endergebnis mindestens folgende Aussagen machen:

(a) Anzahl der behandelten Patienten,

(b) prozentualer Anteil von Patienten, die in der Häuslichkeit verstorben sind,

(c) durchschnittliche Betreuungsdauer**,** durchschnittliche Fahrzeit und durchschnittliche Einsatzzeit gegliedert nach delegierter ärztlicher Leistung, Grund- und Behandlungspflege,

(d) prozentualer Anteil von Patienten, bei denen Ehrenamtliche eingesetzt werden,

(e) Einstufungen der Patienten im SGB XI.

(2) Für die Auswertung hat der Leistungserbringer die einheitlichen Auswertungsbögen gemäß Anlage 7 zu verwenden, für die vorgegebene zeitliche Dauer auszufüllen und der zuständigen Stelle zuzusenden. Kommt der Leistungserbringer dieser Verpflichtung nicht bzw. nur teilweise nach, findet § 31 Abs. 1 Buchstabe c), ggf. auch Buchstabe d) Anwendung.

(3) Der Leistungserbringer erhält das Ergebnis der Auswertung.

## § 34
## Schiedsperson

(1) Kommt eine Einigung nach § 132 a Abs. 2 Satz 1 bis 5 SGB V nicht zu Stande und werden die Verhandlungen als gescheitert erklärt, kann der Vertragsinhalt durch eine unabhängige Schiedsperson festgesetzt werden. Die Aufnahme von Verhandlungen erfolgt spätestens innerhalb von 6 Wochen nach Inkrafttreten der Kündigung und Aufforderung zu Verhandlungen.

(2) Die Schiedsperson ist für jedes Festsetzungsverfahren neu zu bestellen. Kommt zwischen den Vertragsparteien keine Einigung über die nach Satz 1 zu bestellende Schiedsperson innerhalb von zwei Monaten nach Beginn der hierzu aufgenommenen Gespräche zu Stande, ist diese von der für die vertragsschließende Krankenkasse zuständigen Aufsichtsbehörde zu bestimmen. Das Amt beginnt, wenn sich die Schiedsperson gegenüber den Vertragspartnern schriftlich zur Amtsübernahme bereit erklärt hat. Das Amt endet, wenn die Vertragsfestsetzung verbindlich geworden ist.

(3) Die Schiedsperson wird auf Antrag eines der an ihrer Bestellung beteiligten Vertragspartners tätig; sie hat die verfahrensbeteiligten Vertragspartner jeweils mit einer Frist von mindestens vier Wochen zu einer schriftlichen

Stellungnahme aufzufordern und zu einer ersten mündlichen Anhörung einzuladen. Im Anhörungstermin hat sie auf eine gütliche Einigung hinzuwirken. Falls eine solche nicht innerhalb von vier Wochen zu Stande kommt, unterbreitet die Schiedsperson im Anschluss an einen weiteren Anhörungstermin einen schriftlichen Einigungsvorschlag. Wird der Einigungsvorschlag von den Vertragspartnern nicht innerhalb von vier Wochen nach seinem Zugang angenommen, bestimmt die Schiedsperson einen Termin zur mündlichen Verhandlung zur Festsetzung der Vertragsinhalte.

(4) Bei der Vertragsfestsetzung ist die Schiedsperson an die von den Verfahrensbeteiligten gestellten Anträgen gebunden. Vertragsinhalte und Vergütungssysteme, die bisher nicht Gegenstand von Verhandlungen waren, sind nur dann Gegenstand einer Festsetzung, wenn die Vertragspartner insoweit ihr Einverständnis erklären.

(5) Die Kosten des Schiedsverfahrens tragen die Vertragspartner zu gleichen Teilen. Die Erstattung eigener Kosten der Vertragspartner entfällt.

## § 35
## Beendigung des Vertrages

(1) Das Vertragsverhältnis endet, ohne dass es hierzu einer besonderen Kündigung bedarf

(a) mit Einstellung des Geschäftsbetriebes,

(b) mit dem Tod des Trägers (Fall eines einzelunternehmerisch betriebenen Pflegedienstes) *) oder

(c) mit dem Wechsel der Trägerschaft des Leistungserbringers (z. B. durch Veräußerung des Palliativdienstes).

(d) Ein zu Beendigung des Vertrages führender Trägerwechsel liegt nicht vor bei Übertragung von Beteiligungen des Trägers, der Aufnahme von Gesellschaften und Gesellschaftsaustritten. Den Kostenträgern steht jedoch in diesen Fällen ein Sonderkündigungsrecht entsprechend § 32 Abs. 2 dann zu, wenn hierdurch die nach diesem Vertrag erforderlichen Strukturvoraussetzungen nicht nur vorüber-gehend entfallen. Als vorübergehend gilt ein Zeitraum von drei Monaten.

***) Protokollnotiz**

## § 36
## In-Kraft-Treten, Kündigung

(1) Dieser Vertrag tritt einschließlich seiner Anlagen am in Kraft.

(2) Der Vertrag kann zum Ende eines Kalendervierteljahres mit dreimonatiger Frist durch eingeschriebenen Brief von und gegenüber jedem Vertragspartner einzeln gekündigt werden. Die Kündigung von oder gegenüber einem Vertragspartner lässt die Wirksamkeit des Vertrages im Verhältnis zu den übrigen beteiligten Vertragspartnern unberührt.

(3) Abweichend von Abs. 2 gilt für die Vergütungsvereinbarung nach Anlage 6 die dort vorgesehene besondere Kündigungsregelung.

## § 37
## Überleitungsregelung

(1) Dieser Vertrag tritt an die Stelle der zwischen den Landesverbänden der Kranken/Pflegekassen und den ambulanten Palliativdiensten (Anlage 8) im Rahmen des Modellprojektes zur Finanzierung von palliativ-medizinisch und pflegerisch tätigen Hausbetreuungsdiensten in Nordrhein-Westfalen geschlossenen Verträge über die ambulante palliativpflegerische Versorgung auf Basis des § 132 a SGB V i. V. m. § 72 SGB XI.

Voraussetzung hierfür ist der Nachweis:

(a) des Vorliegens der personellen Voraussetzungen (§ 5 Abs. 1 Buchstaben a), b), m) und o) / Anlage 2),

(b) der Kooperation mit einem oder mehreren ambulanten Hospizdiensten zur Sicherstellung der Ehrenamtlichen (§ 7 / Anlage 2a) und

(c) über die Zusammenarbeit mit einem/r qualifizierten Palliativarzt/-ärztin nach § 3 Abs. 1 Buchstabe f) bzw. der zzt. bestehenden Übergangsregelung ent-sprechend dem Rahmenprogramm zur flächendeckenden Umsetzung der ambulanten palliativmedizinischen und palliativpflegerischen Versorgung in NRW vom 19.05.2005. *)

***) Protokollnotiz**

## § 38
## Änderungen und Ergänzungen

Änderungen oder Ergänzungen des Vertrages bedürfen der Schriftform.

## § 39
## Salvatorische Klausel

Sollten einzelne Bestimmungen dieses Vertrages unwirksam sein oder werden, so wird die Gültigkeit des Vertrages im Übrigen hiervon nicht berührt. Anstelle der unwirksamen Bestimmung soll dann eine Vereinbarung treten, die dem Zweck, der mit der unwirksamen Bestimmung verfolgt wird, am nächsten kommt.

<u>Protokollnotiz zu § 3 Abs. 1 Buchstabe f) und § 37 Buchstabe c):</u>
Die Zusammenarbeit mit einem/r qualifizierten Palliativarzt/-ärztin (§ 3 Abs. 1 Buchstabe f) ist spätestens innerhalb von 3 Monaten nach der erstmaligen Bekanntgabe einer entsprechenden Liste für die Region, in der der Leistungserbringer seinen Sitz hat, sicherzustellen. Die Bekanntgabe erfolgt

entweder durch die Landesverbände der Krankenkassen, dem Medizinischen Dienst der Krankenkassen oder der kassenärztlichen Vereinigung Nordrhein bzw. Westfalen-Lippe. Der Nachweis der Zusammenarbeit mit einem qualifizierten Palliativarzt/-ärztin (§ 3 Abs. 1 Buchstabe f) ist unaufgefordert der zuständigen Stelle zuzusenden. In der Übergangszeit ist die Zusammenarbeit mit einem/r palliativmedizinisch erfahrenen Arzt/Ärztin der zuständigen Stelle nachzuweisen.

Protokollnotiz zu § 4 Abs. 1:
Die Weiterbildung hat inhaltlich den Vorgaben des Basiscurriculum Palliativ Pflege des Vereins zur Betreuung und Begleitung von Schwerstkranken und Tumorpatienten e. V., Pallia Med Verlag, Bonn, zu entsprechen.

Protokollnotiz zu § 4 Abs. 2:
Hat der Leistungserbringer nur 4 Vollzeitkräfte nach § 4 Abs. 2 und scheidet eine Voll- oder Teilzeitkraft, die sozialversicherungspflichtig beim Leistungserbringer beschäftigt war, aus, ist der Leistungserbringer verpflichtet, innerhalb von neun Monaten nach Ausscheiden der Pflegefachkraft nach § 4 Abs. 2 die Beschäftigung einer neuen Voll- bzw. Teilzeitkraft nach § 4 Abs. 2 nachzuweisen, da andernfalls § 32 Abs. 2 Anwendung findet. Befindet sich die neue Pflegefachkraft noch in der palliativpflegerischen Weiterbildung (vgl. § 4 Abs. 1 2. Absatz), sind die von ihr erbrachten Leistungen nur nach diesem Vertrag abrechnungsfähig, wenn diese Pflegefachkraft innerhalb der letzten 5 Jahre über eine mindestens einjährige berufspraktische Erfahrungszeit in Vollzeit im palliativpflegerischen Bereich verfügt und der zuständigen Stelle nachgewiesen hat. Bei einer ausgeübten sozialversicherungspflichtigen Teilzeitbeschäftigung (min. 18 Stunden wöchentlich) verlängert sich die einjährige berufspraktische Erfahrungszeit entsprechend. Die zuständige Stelle informiert den Träger, ab welchem Zeitpunkt eine Erbringung und Abrechnung von Leistungen nach diesem Vertrag durch die o.g. Pflegefachkraft erfolgen kann. Die Erbringung und Abrechnung von Leistungen nach diesem Vertrag ist in diesem Falle frühestens mit Vorliegen der o.g. Nachweise bei der zuständigen Stelle möglich. Andernfalls sind die erbrachten Leistungen nur nach dem mit der entsprechenden Krankenkasse vereinbarten Vertrag über die Erbringung häuslicher Krankenpflege nach § 132 a Abs. 2 SGB V abrechnungsfähig. Es findet § 5 Abs. 4

und § 6 Abs. 2 Anwendung.

Protokollnotiz zu § 4 Abs. 3:
Die leitende Pflegefachkraft nach § 4 Abs. 3 kann entweder die Pflegedienstleitung bzw. bei großen Pflegediensten die für den Palliativbereich zuständige Teamleitung sein. Für die stellvertretende Pflegefachkraft gilt dies entsprechend.

Protokollnotiz zu § 5 Abs. 2:
Bei der Entscheidung ist auch auf die persönliche Eignung der verantwortlichen Pflegefachkräfte abzustellen. Insbesondere bei vorausgegangener schwerer oder wiederholter Rechtsverletzung ist den Krankenkassen die Gründung eines Vertragsverhältnisses nicht zuzumuten.

Protokollnotiz zu § 21 Abs. 1:
Es sind die zwischen den Landesverbänden der Kranken-/Pflegekassen in Nordrhein-Westfalen und dem Medizinischen Dienst der Krankenversicherung abgestimmten Schnellbegutachtungsbögen (SGB V/SGB XI) zu verwenden.

Protokollnotiz zu § 35 Abs. 1 Buchstabe b):
Im Falle des Todes des Trägers ist der Erbe berechtigt, - sofern die Leistungen entsprechend diesem Vertrag erbracht werden - die folgenden 2 Kalendermonate mit den vertragsschließenden Krankenkassen abzurechnen.

Anlagen:

2. Nachweis/Handzeichenliste der zzt. beschäftigten Mitarbeiter (§ 5 Abs. 1 Buchstabe b))

2 a. Nachweis/Handzeichenliste der Ehrenamtlichen (§ 5 Abs. 1 Buchstabe m))

3. Inhalt der Leistungen (§ 8)

4. Qualitätssicherung (§ 11 ff.)

5. Leistungsnachweis (§ 19)

6. Vergütungsvereinbarung (§ 22)

7. Auswertungsbögen (§ 33)

Bergisch Gladbach, Bochum, Dortmund, Düsseldorf, Essen, den

AOK Rheinland/Hamburg
- Die Gesundheitskasse

BKK Landesverband
Nordrhein-Westfalen

IKK Nordrhein

Knappschaft

Landwirtschaftliche Krankenkasse NRW

Verband der Ersatzkassen e. V. (vdek)
Der Leiter der Landesvertretung
Nordrhein-Westfalen

Die Anlagen sind dieser Vertragsausführung nicht beigefügt.

# Anlage 9

**Vertrag über die Erbringung**
**Spezialisierter ambulanter Palliativversorgung (SAPV) in Nordrhein**
**gemäß § 132 d SGB V i. V. m. § 37 b SGB V**

**„Schnellbegutachtungsbogen des Medizinischen Dienstes der Krankenversicherung Nordrhein“**

# MDK-Schnellbegutachtungsbogen für die SAPV nach § 37 b SGB V
## - wird vom Palliative-Care-Team ausgefüllt -

## A. Basisdaten Patient, Aktuelle Versorgungssituation

| AOK | BKK | IKK | LKK | Vdek | Knapp | | Palliative-Care-Team |
|---|---|---|---|---|---|---|---|

_______________________________________
(Name der Kasse)

Der Schnellbegutachtungsbogen ist mit der vertragsärztlichen Verordnung direkt an die jeweils zuständige Krankenkasse zu senden.

Name, Vorname: ______________________

Adresse: ______________________

______________________

Telefon-Nr: __________ Geburtsdatum: __________

______________________

Pflegeversicherung

☐ keine ☐ beantragt ☐ Stufe I ☐ Stufe II ☐ Stufe III

☐ Härtefall ☐ unbekannt ☐ Höherstufung beantragt

Name: ______________________

Adresse: ______________________

______________________

Telefon-Nr: __________ Fax: __________

E-Mail: ______________________

Datum: __________ Stempel / Unterschrift: __________

Angehörige / private Pflegepersonen
(bitte jeweils Namen, Adresse und Tel.-Nr. angeben)

| | |
|---|---|
| 1. | |
| 2. | |

Beteiligen sich die Angehörigen / private Pflegepersonen an der Versorgung? ☐ Ja ☐ Nein

Falls ja, welche Leistungen werden übernommen: ______________________

Hauptdiagnose: ______________________
(bitte umfassend angeben mit Stadium der Erkrankung)

ambulante Versorgungssituation
*(alles Zutreffende ankreuzen)*

☐ Hausarzt

☐ Facharzt/Palliativarzt

☐ Sozialstation / Pflegedienst

☐ Palliative-Care-Team

☐ private Pflegeperson(en)

☐ Ambulanter Hospizdienst

Kurative Behandlungen abgeschlossen?

☐ Ja ☐ Nein ☐ Unklar

Behandlungsansatz jetzt rein palliativ?

☐ Ja ☐ Nein ☐ Unklar

Aufklärung über begrenzte Lebenserwartung?

☐ Ja ☐ Nein ☐ Unklar

Patientenverfügung

☐ Ja ☐ Nein

☐ Wird noch bis zum ________ erarbeitet.

Palliativer Behandlungsplan

☐ Ja ☐ Nein

☐ Wird noch bis zum ________ erarbeitet.

Erfolgen bei Bedarf auch nachts Einsätze?

☐ Ja ☐ Nein

## B. Schmerzsymptomatik

Besteht ein ausgeprägtes Schmerzsymptomgeschehen, welches eine Schmerztherapie mit multidimensionaler Therapiekontrolle erforderlich macht?

☐ Ja ☐ Nein

## C. Weitere ausgeprägte und extreme Symptome

☐ Angst ☐ Motorische Unruhe ☐ Atemnot ☐ Blutungen ☐ Übelkeit / Erbrechen ☐ Durchfall ☐ Exulcerationen

☐ ------------------------------------------------------------------------------------------------

## D. Psychosoziale Unterstützung

☐ Psychosoziale Unterstützung durch______________________

☐ Beratung in ethischen Fragen durch ______________________

☐ Psychosoziale Unterstützung ist nicht erforderlich, da ______________________

Anlage: Kopie der vertragsärztlichen Verordnung

# MDK-Stellungnahme
# zum
# MDK-Schnellbegutachtungsbogen für die SAPV nach § 37 b SGB V

Name, Vorname: ______________________________

Adresse: ______________________________

Geb.-Datum: ______________________________

Angehörige/private Pflegepersonen sind vorhanden und beteiligen sich an der Versorgung:

☐ Ja ☐ Nein

falls ja, welche Leistungen werden übernommen: ______________________________

______________________________

Sind häusliche Krankenpflege und/oder andere Leistungen (z. B. ärztliche Leistungen) im Rahmen der allgemeinen Palliativversorgung ausreichend?

☐ Ja ☐ Nein

falls ja, welche Leistungen sind für welchen Zeitraum erforderlich: ______________________________

______________________________

Liegen die besonderen Voraussetzungen für die Erbringung der SAPV vor?

☐ Ja ☐ Nein

falls ja, ab wann: ______________________________

medizinische Begründung: (bitte immer angeben) ______________________________

Weitere Unterlagen sind notwendig:

☐ Ja ☐ Nein

falls ja, welche: ______________________________

**Sonstiges**: ______________________________

______________________________

| Datum: | Stempel / Unterschrift: |
| --- | --- |
| ______________________________ | ______________________________ |

# Anlage 10

**Vertrag über die Erbringung**
**Spezialisierter ambulanter Palliativversorgung (SAPV) in Nordrhein**
**gemäß § 132 d SGB V i. V. m. § 37 b SGB V**

**„Leistungsnachweis“**

**Anlage 10 - Vertrag über die Erbringung Spezialisierter ambulanter Palliativversorgung in Nordrhein gemäß § 132 d SGB V i. V. m. § 37 b SGB V**

| | | | | | |
|---|---|---|---|---|---|
| **Leistungsnachweis:** | Krankenkasse: | Monat/Jahr: .......... ............... | | **Palliative-Care-Team** | **IK-Nr.** |
| **Name, Vorname:** | | KV-Nr. | Pflegestufe: I II III IIII<br>Geb.-Datum | | **Telefon** |

| Art | Datum / Zeit | 1 | 2 | 3 | 4 | 5 | 6 | 7 | 8 | 9 | 10 | 11 | 12 | 13 | 14 | 15 | 16 | 17 |
|---|---|---|---|---|---|---|---|---|---|---|---|---|---|---|---|---|---|---|
| | Uhrzeit | | | | | | | | | | | | | | | | | |
| | Uhrzeit | | | | | | | | | | | | | | | | | |
| | Uhrzeit | | | | | | | | | | | | | | | | | |
| | Uhrzeit | | | | | | | | | | | | | | | | | |
| | Uhrzeit | | | | | | | | | | | | | | | | | |
| | Uhrzeit | | | | | | | | | | | | | | | | | |
| | Uhrzeit | | | | | | | | | | | | | | | | | |
| | Uhrzeit | | | | | | | | | | | | | | | | | |
| | Uhrzeit | | | | | | | | | | | | | | | | | |
| | Uhrzeit | | | | | | | | | | | | | | | | | |

| Art | Datum / Zeit | 18 | 19 | 20 | 21 | 22 | 23 | 24 | 25 | 26 | 27 | 28 | 29 | 30 | 31 | Anzahl Komplexe | Komplex-preis | Gesamt-preis |
|---|---|---|---|---|---|---|---|---|---|---|---|---|---|---|---|---|---|---|
| | Uhrzeit | | | | | | | | | | | | | | | | | |
| | Uhrzeit | | | | | | | | | | | | | | | | | |
| | Uhrzeit | | | | | | | | | | | | | | | | | |
| | Uhrzeit | | | | | | | | | | | | | | | | | |
| | Uhrzeit | | | | | | | | | | | | | | | | | |
| | Uhrzeit | | | | | | | | | | | | | | | | | |
| | Uhrzeit | | | | | | | | | | | | | | | | | |
| | Uhrzeit | | | | | | | | | | | | | | | | | |
| | Uhrzeit | | | | | | | | | | | | | | | | | |
| | Uhrzeit | | | | | | | | | | | | | | | | | |

Ich bestätige die vertragsgemäße Ausführung der o.a. Leistungen:

Datum: Unterschrift Verantwortlicher Palliative-Care-Team:

Datum: Unterschrift Patient/gesetzl. Vertreter oder Bevollmächtigter

Rhineland-Palatinate

# Vertrag nach § 132 d SGB V über die spezialisierte ambulante palliativmedizinische Versorgung von unheilbar erkrankten Patienten im häuslichen Umfeld

**zwischen**

.......................................
.......................................
.......................................

im Folgenden als „SAPV-Leistungserbringer“ bezeichnet

**und**

der AOK - Die Gesundheitskasse in Rheinland-Pfalz, Eisenberg

dem BKK-Landesverband Rheinland-Pfalz und Saarland, Mainz
*handelnd für die nach Vertragsabschluss gegenüber dem BKK-Landesverband beitretenden Betriebskrankenkassen*

der IKK Südwest, Saarbrücken

der Knappschaft, Regionaldirektion Saarbrücken

der LKK Hessen, Rheinland-Pfalz und Saarland, Speyer
zugleich handelnd als Landesverband für die Krankenkasse für den Gartenbau

den Ersatzkassen
- Barmer GEK,
- Techniker Krankenkasse (TK),
- Deutsche Angestellten-Krankenkasse (Ersatzkasse),
- KKH-Allianz (Ersatzkasse),
- HEK – Hanseatische Krankenkasse,
- hkk,

gemeinsamer Bevollmächtigter mit Abschlussbefugnis:
Verband der Ersatzkassen e.V. (vdek),
vertreten durch den Leiter der vdek-Landesvertretung Rheinland-Pfalz

im Folgenden als „Krankenkassen“ bezeichnet

Muster

| §§ | Inhalt | Seite |
|---|---|---|
| | Präambel | 3 |
| 1 | Gegenstand des Vertrags / Versorgungsregion | 4 |
| 2 | Grundlagen und Ziele des Vertrags | 4 |
| 3 | Anspruchsvoraussetzungen der Versicherten / Patienten | 5 |
| 4 | Inhalt und Umfang der Leistungserbringung | 5 |
| 5 | Teilnahmevoraussetzungen für den SAPV-Leistungserbringer | 5 |
| 6 | Versorgungskonzept | 6 |
| 7 | Anzeigepflichten / Mitteilungspflichten | 7 |
| 8 | Verordnung / Genehmigung der SAPV | 7 |
| 9 | Kostenzusage | 8 |
| 10 | Verordnung von Arznei-, Heil- und Hilfsmitteln im Rahmen der SAPV | 9 |
| 11 | Qualitätssicherungsmaßnahmen | 9 |
| 12 | Sicherstellungsauftrag / sonstige Versorgungsverträge | 11 |
| 13 | Vergütung und Abrechnung | 11 |
| 14 | Institutionskennzeichen | 13 |
| 15 | Datenschutz / Schweigepflicht | 13 |
| 16 | Vertragsverstöße | 14 |
| 17 | Schlussbestimmungen | 14 |
| 18 | Inkrafttreten und Kündigung | 14 |
| | *<u>Anlagen</u>* | |
| *1* | *Richtlinien des Gemeinsamen Bundesausschusses* | *16* |
| *2* | *Empfehlungen der Spitzenverbände* | *24* |
| *3* | *Vergütungsvereinbarung und Leistungsbeschreibung* | *30* |
| *3-1* | *Datensatzbeschreibung zur Abrechnung* | *34* |
| *3-2* | *Abrechnungsbogen* | *41* |
| *3-3* | *Erfassungsbogen, Muster* | *42* |
| *4* | *Leitfaden zur Konzeption der SAPV* | *52* |
| *5* | *Ergebnis Assessment (Krankenkasse / Verordner), Muster* | *54* |
| *6* | *Leistungsnachweis, Muster* | *56* |
| *7* | *Einverständniserklärung Versicherter, Muster* | *58* |
| *8* | *Dokumentation / Eingangsassessment[1], Muster* | *59* |
| *9* | *Strukturerhebungsbogen, Muster* | *68* |


Muster

[1] Diese Anlage ist ggf. an die Evaluations- / Dokumentationsvorgaben auf Bundesebene anzupassen

## Präambel

Die spezialisierte ambulante Palliativversorgung (SAPV) dient dem Ziel, die Lebensqualität und die Selbstbestimmung schwerstkranker Menschen zu erhalten, zu fördern und zu verbessern und ihnen ein menschenwürdiges Leben bis zum Tod in ihrer vertrauten häuslichen Umgebung oder in stationären Pflegeeinrichtungen, Einrichtungen der Eingliederungshilfe sowie stationären Hospizen zu ermöglichen. Die SAPV-Leistungen für Kinder- und Jugendliche werden gesondert geregelt.

Versorgungsunsicherheiten, die zu unnötigen und die Versicherten stark belastenden Krankenhauseinweisungen führen, sollen durch die in der SAPV vorgehaltenen besonderen Fachkenntnisse vermieden werden. Symptome und Leiden sollen einzelfallgerecht und nach den Wünschen der Versicherten durch palliativmedizinisch-palliativpflegerische Maßnahmen gelindert oder behoben werden.

Die Versorgung des Versicherten/der Versicherten bedarf einer besonderen SAPV-Fachkompetenz. Sie muss darüber hinaus ausreichend und zweckmäßig sein, sie orientiert sich an dem individuellen Hilfebedarf des Versicherten, darf das Maß des Notwendigen nicht überschreiten und muss wirtschaftlich erbracht werden.

Der Versicherte/die Versicherte und möglichst auch sein/ihr soziales Umfeld (seine vertrauten Personen) sollen in die Versorgung mit einbezogen und über die Krankheit und den Krankheitsverlauf aufgeklärt und bei psychosozialen / seelischen Problemen unterstützt werden.

Anspruch auf die Leistung der SAPV und damit einer besonderen (aufwändigen) Versorgung besteht, soweit die anderweitigen ambulanten ärztlichen und pflegerischen Versorgungsformen (z.B. häusliche Krankenpflege) sowie ggf. die Leistungen des ambulanten Hospizdienstes nicht oder nur unter besonderer Koordination ausreichen würden. Die SAPV ergänzt insoweit das bestehende Versorgungsangebot, insbesondere das der Vertragsärzte, Krankenhäuser und Pflegedienste.

Muster

## § 1
## Gegenstand des Vertrages / Versorgungsregion

(1) Gegenstand des Vertrages ist die Erbringung der spezialisierten ambulanten Palliativversorgung gem. § 37 b SGB V in Verbindung mit § 132d SGB V.

(2) Dieser Vertrag dient der Sicherstellung der bedarfsgerechten individuellen Versorgung des/der anspruchsberechtigten Versicherten mit SAPV in dem im Versorgungskonzept ausgewiesenen Versorgungsgebiet durch den SAPV-Leistungserbringer, im Rahmen seiner Kapazitäten. Die Bedarfsgerechtigkeit einer Region bedeutet u.a., dass eine SAPV - Versorgung qualitativ gleichwertig bei unterschiedlichen Bevölkerungsdichten zu erfolgen hat.

(3) Die Versorgungsregion dieses Vertrags betrifft die Region XX im Bundesland Rheinland-Pfalz. Für Versicherte in dieser Region übernimmt der SAPV-Leistungserbringer eine Versorgungsverpflichtung; Versicherte aus anderen Versorgungsregionen können versorgt werden.

Muster

## § 2
## Grundlagen und Ziele des Vertrages

(1) Bestandteil dieses Vertrages sind die Richtlinien des Gemeinsamen Bundesausschusses zur Verordnung von spezialisierter ambulanter Palliativversorgung vom 20. Dezember 2007 in der jeweils gültigen Fassung. Weitere Grundlage sind die Gemeinsamen Empfehlungen der Spitzenverbände der Krankenkassen nach § 132d SGB V für die spezialisierte ambulante Palliativversorgung vom 23. Juni 2008 in der jeweils gültigen Fassung (Anlagen 1 und 2), soweit in diesem Vertrag nichts Abweichendes geregelt ist.

(2) Mit diesem Vertrag erstreben die Vertragspartner eine effektive und fachlich angemessene, qualitätsgesicherte Versorgung von Palliativpatienten in ihrem persönlichen Umfeld. Die bestehenden und bewährten Versorgungsstrukturen in Rheinland-Pfalz, insbesondere die auf ehrenamtlicher Arbeit basierenden ambulanten Hospizdienste werden ergänzt.

(3) Die spezialisierte ambulante Palliativversorgung gemäß § 37 b SGB V (SAPV) dient dem Ziel die Lebensqualität und die Selbstbestimmung Schwerstkranker zu erhalten, zu fördern und zu verbessern und ihnen ein menschenwürdiges Leben bis zum Tod in ihrer vertrauten häuslichen Umgebung zu ermöglichen. Im Vordergrund steht anstelle eines kurativen Ansatzes die medizinisch-pflegerische Zielsetzung, Symptome und Leiden einzelfallgerecht zu lindern.

(4) Die erforderliche Multiprofessionalität, die sektorenübergreifende, von Behandlungsbrüchen möglichst freie Versorgung, die gemeinsame Dokumentation sowie die Qualitätsentwicklung sind unabdingbare Voraussetzung für eine fachlich angemessene humane effektive hospizlich-palliative Versorgung.

## § 3
## Anspruchsvoraussetzungen der Versicherten / Patienten

(1) Die Anspruchsvoraussetzungen der Versicherten / Patienten auf spezialisierte ambulante Palliativversorgung ergeben sich aus den Richtlinien des Gemeinsamen Bundesausschusses zur Verordnung von spezialisierter ambulanter Palliativversorgung (Anlage 1).

(2) Ein Anspruch auf SAPV liegt in der Regel nicht vor, wenn die multiprofessionelle Zusammenarbeit der an der Versorgung des Versicherten Beteiligten nicht erforderlich ist, um das Versorgungs- / Behandlungsziel zu erreichen; dies ist insbesondere der Fall, wenn die Leistungen einzelner an der Versorgung Beteiligter jeweils für sich ausreichend sind.

(3) Die individuelle Versorgung der/des Versicherten gem. SAPV – RL wird nicht unterbrochen sofern der die Versicherte einzelne Maßnahmen der Versorgung ablehnt. Der Anspruch auf Versorgung mit SAPV bleibt hierdurch unberührt.

(4) Der / die Versicherte bleibt in der Wahl der Leistungserbringer frei.

Muster

## § 4
## Inhalt und Umfang der Leistungserbringung

(1) Inhalt und Umfang der Leistungserbringung im Rahmen der SAPV ergeben sich aus den Richtlinien des Gemeinsamen Bundesausschusses zur Verordnung von spezialisierter ambulanter Palliativversorgung (Anlage 1).

(2) Details zur Leistungsbeschreibung regelt die Vergütungsvereinbarung (Anlage 3).

## § 5
## Teilnahmevoraussetzungen für den SAPV-Leistungserbringer

(1) Leistungen im Rahmen der SAPV werden von SAPV-Leistungserbringern im Sinne der Gemeinsamen Empfehlungen der Spitzenverbände der Krankenkassen nach §132 d SGB V (Anlage 2) erbracht. Hierzu zählen qualifizierte Ärzte, qualifizierte Pflegefachkräfte sowie ggfls. weitere qualifizierte Fachkräfte.

(2) Qualifikationen und Aufgaben der SAPV-Leistungserbringer ergeben sich aus den Gemeinsamen Empfehlungen der Spitzenverbände der Krankenkassen nach § 132 d SGB V (Anlage 2) für die spezialisierte ambulante Palliativversorgung, sofern in diesem Vertrag nichts Abweichendes geregelt ist. Übergangsregelungen können in den Einzelverträgen mit den SAPV-Leistungserbringern vereinbart werden, sofern hierfür eine Notwendigkeit besteht.

(3) Als Voraussetzungen zur Erbringung von spezialisierter ambulanter Palliativversorgung gelten für den SAPV-Leistungserbringer folgende Kriterien:

a) Kenntnis und Akzeptanz des Vertragsinhalts

b) Konzeption des SAPV-Leistungserbringers zur spezialisierten palliativmedizinischen Versorgung gemäß den Kriterien der Anlage 4; diese muss vor Vertragsschluss zur Prüfung bei den vertragschließenden Krankenkassen eingereicht werden

c) Bereitschaft des SAPV-Leistungserbringers zur notwendigen Hausbesuchstätigkeit im Rahmen dieses Vertrages bei SAPV-Patienten

d) verantwortungsbewusster Umgang mit veranlassten GKV-Leistungen, insbesondere im Hinblick auf das Wirtschaftlichkeitsgebot gemäß § 12 SGB V und beim Einsatz von Arzneimitteln. Eine unverzügliche Versorgung mit Hilfsmitteln ist von den Krankenkassen zu gewährleisten.

e) Bereitschaft zum multiprofessionellen und interdisziplinären Gespräch und zur Abstimmung mit den anderen Leistungserbringern zur weiteren Versorgung der jeweiligen Patienten

## § 6
## Versorgungskonzept

(1) Der SAPV-Leistungserbringer handelt als einheitlicher Leistungserbinger im Sinne der Konzeption eines Palliative-Care-Teams (PCT) und eines verbindlichen, strukturierten und schriftlich dargelegten Versorgungskonzeptes.

(2) Das Versorgungskonzept formuliert alle palliativmedizinisch-palliativpflegerischen Maßnahmen klar und eindeutig unter Beachtung von Maßnahmen zur Qualitätssicherung (§ 11). Es muss darüber hinaus verdeutlichen, wie eine qualifizierte Leistungserbringung zu erfolgen hat, damit die in § 2 dieses Vertrages genannten Vertragsziele erreicht werden können.

(3) Die regionalen Netzwerkstrukturen, in die sich der SAPV Leistungserbringer im Rahmen der Vernetzung integriert, sind vor Vertragsabschluss vom SAPV-Leistungserbringer zu beschreiben.

(4) Das Versorgungskonzept ist den Krankenkassen vorab zur Verfügung zu stellen. Die Vertragsziele gemäß § 2 dieses Vertrages werden mit dem Versorgungskonzept umgesetzt und erfüllt.

(5) Das Versorgungskonzept beinhaltet insbesondere folgende Aspekte:

a. Standort des Leistungserbringers,
b. Versorgungsgebiet,
c. Versorgungsprozess,
d. Kooperationspartner,
e. Kooperationsvereinbarungen mit im Rahmen des Netzwerkes an der Versorgung Beteiligten im Versorgungsgebiet (z. B. ambulante Hospizdienste, Seelsorge) zur Sicherung der psychosozialen Unterstützung,
f. Mitarbeiterkapazität (Stellenplan),
g. Personelle Zuständig- und Verantwortlichkeiten der Mitarbeiter der SAPV-Leistungserbringer (u.a. Weisungsbefugnisse, Weisungsgebundenheit),
h. Qualifikationsnachweise der Personen gemäß § 5 Abs. 2,
i. sächliche Ausstattung,
j. Qualitätssicherungsmaßnahmen gemäß § 11,
k. Konzept zur Sicherstellung der reibungslosen Versorgung mit Arznei-, Heil- und Hilfsmitteln,
l. Konzept zur Sicherstellung der psychosozialen und seelischen Unterstützung,
m. Konzept zur Sicherstellung der 24-Stunden-Rufbereitschaft,

(6) Die Leistungserbringung ist durch den SAPV-Leistungserbringer in Form geeigneter Maßnahmen unter Einsatz entsprechender personeller und sächlicher Mittel für die Dauer des Vertrages sicherzustellen. Die Krankenkassen sind umgehend zu informieren, wenn die Voraussetzungen zur Leistungserbringung nicht mehr erfüllt sind.

(7) Der SAPV-Leistungserbringer ist weisungsunabhängig.

(8) Die Personalstärke eines Teams bestimmt sich u.a. durch die Anforderungen zur Erbringung ärztlicher und pflegerischer Leistungen, der 24-Stunden-Erreichbarkeit für betreute Versicherte, der qualifizierten Vertretung in Urlaubs- und Krankheitszeiten und der regionalen strukturellen Gegebenheiten.

(9) Der SAPV-Leistungserbringer trägt die Verantwortung für das Versorgungskonzept.

Muster

## § 7
## Anzeigepflichten / Mitteilungspflichten

(1) Der SAPV-Leistungserbringer ist verpflichtet, den Krankenkassen anstehende Änderungen wesentlicher Umstände im Versorgungskonzept schriftlich innerhalb von 4 Wochen mitzuteilen. Dies sind insbesondere:

a. jede Änderung der betrieblichen/organisatorischen Gegebenheiten

b. Änderungen von Kooperationsvereinbarungen (Neuabschlüsse, Kündigung, inhaltliche Änderungen)

c. Strafrechtliche Maßnahmen gegen den SAPV-Leistungserbringer im Zusammenhang mit der Leistungserbringung

d. personelle Änderungen im SAPV-Leistungserbringer.

(2) Die Krankenkassen sind berechtigt, bei begründetem Anlass zum Nachweis der organisatorischen und/oder personellen Voraussetzungen weitere Unterlagen zur Einsichtnahme zu verlangen. Der SAPV-Leistungserbringer ist verpflichtet, diese zur Verfügung zu stellen.

Muster

## § 8
## Verordnung / Genehmigung der SAPV

(1) Art, Umfang und Dauer der vom SAPV-Leistungserbringer zu erbringenden Leistungen ergeben sich aus der von der jeweiligen Krankenkasse genehmigten Verordnung der behandelnden Vertragsärztin/des behandelnden Vertragsarztes oder der Krankenhausärztin/des Krankenhausarztes.

(2) Die Verordnung ist nicht übertragbar. Sie gilt nur für die Person, für die sie ausgestellt ist.

(3) Die Annahme von Aufträgen zur Versorgung anspruchsberechtigter Personen dieses Vertrages und deren Weitergabe (Vermittlung an Dritte gegen Entgelt oder zur Erlangung geldwerter Vorteile) gilt als Verstoß gemäß § 16. Vermittlung im Sinne dieser Bestimmung ist auch die Weitergabe von in eigenem Namen ange- nommenen Aufträgen zur Versorgung anspruchsberechtigter Personen dieses Vertrages an Dritte gegen Kostenerstattung. Vorstehendes gilt nicht für die Weitergabe an Mitglieder des SAPV-Teams oder an Kooperationspartner gem. § 6.

(4) Findet im Verordnungszeitraum eine Veränderung der Patientensituation statt, die eine Anpassung ermöglicht (Verringerung des Leistungsspektrums) oder nötig macht (Erhöhung des Leistungsspektrums) ist die SAPV-Leistung durch den SAPV-Leistungserbringer entsprechend und unverzüglich anzupassen. Eine erneute Verordnung innerhalb des ursprünglichen Geltungszeitraumes ist nicht erforderlich.
Änderungen und Ergänzungen der Verordnung bedürfen gemäß der Vordruck- vereinbarung der erneuten Unterschrift des Arztes mit Stempel und Datumsangabe (Muster 63).
Vergütungsrelevante Änderungen der SAPV-Leistungen sind der Krankenkasse unverzüglich mitzuteilen.

(5) Leistungen der SAPV werden von der jeweiligen Krankenkasse nur genehmigt, wenn eine ordnungsgemäß ausgefüllte ärztliche Verordnung gemäß der zwischen den Spitzenverbänden der Krankenkassen und der Kassenärztlichen Bundesvereinigung geschlossenen Vereinbarung vorliegt. Der/die Versicherte oder sein/ihr gesetzlicher Vertreter oder die Angehörigen hat die Genehmigung der Verordnung schriftlich auf der Verordnung zu beantragen und eigenhändig zu unterzeichnen.

(6) Die Unterschrift auf dem Genehmigungsformular durch den SAPV-Leistungserbringer bzw. dessen Mitarbeiter ist ausgeschlossen. Kann die Unterschrift nicht erbracht werden, weil der Versicherte bzw. sein gesetzlicher Vertreter nicht in der Lage ist zu unterschreiben, bleibt das Unterschriftsfeld auf der Verordnung frei. Der Leistungserbringer gibt dort oder auf einer Anlage zur Verordnung die Gründe hierfür an.

(7) Die SAPV-Richtlinie ist für die Verordnung sowie für die Genehmigung durch die Krankenkasse maßgeblich. Dort nicht aufgeführte Inhalte bedürfen einer nachvollziehbaren Begründung, damit sie von den Krankenkassen genehmigt werden können.

(8) Der verordnende Arzt/die verordnende Ärztin legt nach aktuellem Versorgungsbedarf die Dauer und den Umfang der SAPV-Leistungen der Verordnung fest. Auch die Krankenhausärztin/der Krankenhausarzt kann Leistungen verordnen. Sollte eine Versorgung für einen längeren Zeitraum erforderlich sein, ist rechtzeitig eine Folgeverordnung bei der zuständigen Krankenkasse einzureichen.

(9) Die Verordnung für einen zurückliegenden Zeitraum ist nicht zulässig. Zulässig ist es jedoch, den Verordnungsumfang in nicht voraussehbaren Krisensituationen dem aktuellen Bedarf anzupassen. Die Änderung des Verordnungsumfanges ist der zuständigen Krankenkasse vom SAPV-Leistungserbringer unverzüglich mit Begründung mitzuteilen.

(10) Der SAPV-Leistungserbringer prüft schnellstmöglich jedoch spätestens innerhalb von 3 Arbeitstagen nach Erhalt der Verordnung die Notwendigkeit und Eignung der besonderen Mittel der SAPV. Sofern für eine bedarfsgerechte Versorgung die Mittel der Regelversorgung ausreichen, ist die zuständige Krankenkasse hierüber unverzüglich zu informieren. Neben der Krankenkasse sind auch der/die Versicherte / bzw. sein gesetzlicher Vertreter und der Verordner über die Ergebnisse der Prüfung zu informieren.
Stellt sich im Nachhinein z.B. im Rahmen einer MDK-Begutachtung heraus, dass die leistungsbegründenden Faktoren, um die Mittel der SAPV in Anspruch zu nehmen, zum Zeitpunkt der SAPV-Leistungserbringung nicht vorlagen, so besteht kein Anspruch, gegen diesen Vertrag abzurechnen. Sollte sich im Nachhinein herausstellen, dass die SAPV Versorgung nicht in dem Maße erforderlich war, wie sie vom SAPV-Leistungserbringer erbracht wurde, so können nur die notwendigen und zweckmäßigen SAPV-Leistungen abgerechnet werden.

Muster

(11) Kann die Durchführung der verordneten Leistung nicht mehr erfolgen, z. B. weil der/die Versicherte in ein Krankenhaus eingewiesen wurde, informiert der SAPV-Leistungserbringer die zuständige Krankenkasse und den behandelnden Arzt unverzüglich. Gleiches gilt, wenn nach initialer Aufnahme in die SAPV, im Rahmen einer SAPV-Leistungserbringung mit direktem Patientenkontakt festgestellt wird, dass die besonderen Mittel der SAPV nicht mehr notwendig sind und die Mittel der Regelversorgung ausreichen.

(12) Umfasst die Dauer der Verordnung einen längeren Zeitraum als die Leistungserbringung am Versicherten notwendig oder möglich ist, kann nur dieser Zeitraum abgerechnet werden. Die Gültigkeitsdauer der SAPV-Verordnung endet automatisch mit dem Tod der / des Versicherten.

## § 9
## Kostenzusage

(1) Die Krankenkasse übernimmt vom ärztlich festgelegten Leistungsbeginn an bis zu einer Entscheidung über die weitere Leistungserbringung die Kosten für die verordneten und vom Leistungserbringer erbrachten Leistungen entsprechend der Vergütung gemäß dieses Vertrages, wenn die ordnungsgemäß ausgefüllte Verordnung spätestens am dritten der Ausstellung folgenden Arbeitstag (ggf. vorab per Fax) der Krankenkasse vorliegt. Sie informiert hierüber den Leistungserbringer.

(2) Soweit die verordneten Leistungen nicht oder nur zum Teil übernommen werden, informiert die Krankenkasse den/die Versicherte/n oder eine ihm nahe stehende Person, den Leistungserbringer und den verordnenden Arzt.

(3) Sofern die Verordnung verspätet eingereicht wird, werden die Kosten erst ab Eingangsdatum übernommen.

(4) Liegt der Krankenkasse innerhalb von 7 Arbeitstagen ab Verordnungsbeginn zusätzlich zur Erstverordnung kein Ergebnis über das Eingangsassessment/die Prüfung des SAPV-Leistungserbringers und eine Einschätzung der notwendigen SAPV Leistungen vor, endet die Kostenzusage mit Ablauf dieses Tages. Die Krankenkasse informiert den/die Versicherte/n oder eine ihm nahe stehende Person, den SAPV-Leistungserbringer und den verordnenden Arzt.

## § 10
## Verordnung von Arznei-, Heil- und Hilfsmitteln im Rahmen der SAPV

(1) Die Verordnung von Arznei-, Heil- und Hilfsmitteln erfolgt ausschließlich unter Verwendung der für die vertragsärztliche Versorgung geltenden Vordrucke (Muster 13, 14, 16 und 18). Bei der (zeitgleichen) Verordnung von Arzneimitteln einerseits und Hilfsmitteln andererseits sind jeweils getrennte Vordrucke nach Muster 16 zu verwenden.

(2) Die Verordnungen sind auf Basis der geltenden Richtlinien des G-BA auszuführen. Dabei ist das Wirtschaftlichkeitsgebot nach § 12 SGB V zu beachten. Soweit generische Substanzen verfügbar sind, sind diese grundsätzlich zu verordnen. Haben Krankenkassen Rabattverträge nach § 130a Abs. 8 SGB V abgeschlossen, sind diese grundsätzlich zu beachten.

(3) Die Belieferung von verordneten Hilfsmitteln erfolgt grundsätzlich im Rahmen des jeweiligen Hilfsmittelverfahrens der einzelnen Krankenkasse. Damit eine schnelle Sicherstellung der Belieferung realisiert werden kann, sollte die leistungspflichtige Krankenkasse über die Notwendigkeit der Hilfmittelverordnung sofort informiert werden.

(4) Für die im Rahmen der SAPV verordneten Arznei-, Heil- und Hilfsmittel gelten die nach §§ 300, 302 SGB V vereinbarten Abrechnungsverfahren entsprechend.

(5) Die Vordrucke nach Abs. 1 dürfen im Rahmen der SAPV nur von den qualifizierten Palliativärzten des SAPV-Leistungserbringers verwendet werden, wenn der SAPV Leistungserbringer einen rechtsgültigem Vertrag nach § 132 d Abs. 1 SGB V hat. Auf jeder Verordnung im Rahmen der SAPV sind die SAPV-spezifische Betriebsstätten-Nummer des SAPV-Leistungserbringers und die Pseudo-Arztnummer des SAPV-Leistungserbringers (333333300) anzugeben. Diese Nummern dürfen nur für Verordnungen im Rahmen der SAPV auf Basis einer gültigen SAPV-Verordnung verwendet werden.

(6) Auch auf Betäubungsmittel-Rezepten sind die SAPV Betriebsstätten-Nummer sowie die Pseudo-Arztnummer anzugeben. Solange das BtM-Muster noch nicht angepasst ist, ist die Betriebsstätten-Nummer in das Feld „Vertragsarztnummer“ und die Pseudo-Arztnummer in das Feld „VK gültig bis“ einzutragen.[2]

(7) Die Betriebsstättennummer und die Pseudoarztnummer müssen vom SAPV-Leistungserbringer gemäß den Bestimmungen der Vereinbarung zwischen dem GKV-Spitzenverband und der Kassenärztlichen Bundesvereinigung über die Vergabe von Betriebsstätten-Nummern und einer Pseudo-Arztnummer an Leistungserbringer der spezialisierten ambulanten Palliativversorgung (SAPV) gemäß § 132 d Abs. 1 SGB V zur Verordnung von Arznei-, Heil- und Hilfsmitteln im Rahmen der SAPV (SAPV-BSNR-Vereinbarung), in der jeweils gültigen Fassung, beantragt und den Krankenkassen mitgeteilt werden.

(8) Der SAPV-Leistungserbringer erstattet den Krankenkassen die Kosten für die Vergabe der Betriebsstätten-Nummer, die Bearbeitung von diesbezüglichen Änderungsmeldungen sowie die Führung und Bereitstellung des Verzeichnisses nach Nr. 3 Absatz 3 der in Abs. 7 genannten Vereinbarung zwischen dem GKV-Spitzenverband und der Kassenärztlichen Bundesvereinigung.

## § 11
## Qualitätssicherungsmaßnahmen

(1) Die SAPV-Leistungserbringer sind verpflichtet, ein internes Qualitätsmanagement durchzuführen. Sie nehmen regelmäßig an palliativmedizinischen/-pflegerischen Fortbildungen teil und führen möglichst halbjährlich multidisziplinare Qualitätszirkel durch, an denen auch die übrigen in der Versorgung Tätigen teilnehmen. Die SAPV-Leistungserbringer sollen sich außerdem an Maßnahmen der externen Qualitätssicherung beteiligen.

(2) Regelmäßige Teamsitzungen sowie multiprofessionelle Fallbesprechungen sind in regelmäßigen Intervallen durchzuführen. Die inhaltliche und zeitliche Organisation ist den Beteiligten überlassen. Die Teilnahme an der Supervision ist zu ermöglichen.

Muster

[2] Regelung zur Weiterverwendung von nicht aufgebrauchten BtM i.R.d. Vorhaltung (z.B. eines Notfallkoffers) auf Bundesebene notwendig => MASGFF RLP hat Prüfung zugesichert

(3) Der SAPV-Leistungserbringer erstellt einen jeweils individuellen Hilfeplan, der mit den übrigen an der Versorgung beteiligten Leistungserbringern abzustimmen ist.

(4) Ein geeignetes Dokumentationssystem ist sachgerecht und kontinuierlich zu führen. Es muss patientenbezogene Daten und soweit vorhanden allgemein anerkannte Indikatoren für eine externe Qualitätssicherung enthalten und eine bundesweite Evaluation ermöglichen.

(5) Die SAPV-Dokumentation gemäß Anlage 8 ist allen an der spezialisierten palliativmedizinischen Versorgung des jeweiligen Versicherten beteiligten Leistungserbringern zugänglich zu machen.

(6) Der SAPV-Leistungserbringer hat eine Patientendokumentation zu führen, aus der zu jeder Zeit die notwendigen Informationen über den Versicherten und die erbrachten Leistungen hervorgehen. Die Patientendokumentation wird regelmäßig von den Mitarbeitern des SAPV-Leistungserbringers aktualisiert. Die Dokumentation entspricht den Anforderungen nach Abs. 7.

(7) Die Dokumentation beinhaltet insbesondere:

a) ein Versichertenstammblatt (z.B. mit Angaben zum/zur Versicherten und Angehörigen, Hausarzt/ärztin, Pflegedienst, Diagnosen, Absprachen, Behandlungsort, Medikation, Maßnahmen, u.w.)

b) informationsberechtigte Personen u. zeichnungsberechtigte Personen

c) Assessment gemäß Anlagen 5 und 8

d) Hilfeplan und Durchführungsdokumentation

e) Nachweise über die durchgeführten Maßnahmen in den Bereichen Beratung, Koordination, Pflege, Medizin, psychosoziale Unterstützung (Datum, Uhrzeit, Dauer), Leistungsnachweis gemäß Anlage 6

f) einen Notfallplan / vorausschauenden Behandlungsplan

g) Untersuchungsbefunde

h) das vertragsärztliche Verordnungsblatt für die Dokumentation der medizinischen Diagnostik und Therapie

i) Dokumentaion der Beurteilung und Anpassung der medizinischen, pflegerischen und psychosozialen Maßnahmen

(9) Jede an dem/der Versicherten bzw. für dessen/deren Angehörige erbrachte Leistung wird durch die behandelnde qualifizierte Person am Tag der Leistungserbringung dokumentiert. Diese ist vom Versicherten, seinem/ihrem gesetzlicher Vertreter oder den berechtigten Angehörigen gegenzuzeichnen.

(10) Sämtliche zur Dokumentation gehörenden Dokumente sind Eigentum des SAPV-Leistungserbringers. Die Dokumente, die zum abgestimmten Handeln der an der pflegerischen und medizinischen versorgung beteilgten Personen erforderlich sind, verbleiben jedoch während des Versorgungszeitraumes durchgängig beim Versicherten.

(11) Die SAPV-Dokumentation gemäß Anlage 8 ist dem MDK/SMD und – soweit der Versicherte eingewilligt hat (§ 67 b Abs. 1 SGB X) – der Krankenkasse auf Anforderung zugänglich zu machen.

(12) Die Aufbewahrungsfrist für die Dokumentation beträgt 10 Jahre nach Ende des Kalenderjahres der Leistungserbringung und obliegt dem SAPV-Leistungserbringer.

Muster

## § 12
## Sicherstellungsauftrag / sonstige Versorgungsverträge

(1) Durch den Vertrag über die „spezialisierte palliativmedizinische Versorgung von unheilbar erkrankten Patienten im häuslichen Umfeld“ und dessen Durchführung wird der Sicherstellungsauftrag der Kassenärztlichen Vereinigung nicht in Frage gestellt bzw. eingegrenzt.

(2) Durch den Vertrag über die „spezialisierte palliativmedizinische Versorgung von unheilbar erkrankten Patienten im häuslichen Umfeld“ und dessen Durchführung bleiben die im übrigen bestehenden Versorgungsverträge der anderen Leistungserbringer mit den Krankenkassen unberührt.

(3) Den an der spezialisierten ambulanten Palliativversorgung teilnehmenden Versicherten bleibt das Recht auf Teilnahme an der vertragsärztlichen Versorgung erhalten.

(4) Die Vertragspartner sind sich darüber einig, dass – soweit Versorgung im Rahmen des Sicherstellungsauftrags bzw. im Rahmen bestehender Versorgungsverträge erfolgt – diese mit der Gesamtvergütung bzw. der im jeweiligen Versorgungsvertrag vereinbarten Vergütung abgegolten ist.
Lediglich die besonderen Leistungen der spezialisierten ambulanten Palliativversorgung in diesem Vertrag werden vom teilnehmenden Leistungserbringer außerhalb des Sicherstellungsauftrags der Kassenärztlichen Vereinigung bzw. außerhalb der bestehenden Versorgungsverträge geleistet.

Muster

## § 13
## Vergütung und Abrechnung

(1) Die Vergütung der erbrachten Leistungen nach diesem Vertrag erfolgt nach der Vergütungsvereinbarung (Anlage 3).

(2) Die Sätze der Vergütungsvereinbarung gelten auch dann, wenn die Krankenkasse im Ausnahmefall aus versicherungsrechtlichen Gründen (z.B. Kassenwechsel) nur einen Teil der Leistungen vergütet. Zuzahlungen dürfen im Rahmen der SAPV-Leistungen nach diesem Vertrag nicht gefordert werden. Soweit für die Vergütungen nach diesem Vertrag Umsatzsteuerpflicht besteht, ist diese mit dem vereinbarten Betrag abgegolten.

(3) Die Rechnungen über die nach diesem Vertrag zu vergütenden SAPV-Leistungen sind vom SAPV-Leistungserbringer nach Leistungserbringung quartalsweise, sofern mit der einzelnen Krankenkasse nichts Abweichendes vereinbart ist, bei den zuständigen Krankenkassen oder den von ihnen benannten Abrechnungsstellen einzureichen.
Die vom Leistungserbringer durchgeführten Leistungen sind im Leistungsnachweis bzw. Durchführungskontrollblatt zu dokumentieren und je Einsatz durch den die Leistung erbringenden Mitarbeiter abzuzeichnen.
Bei elektronischer Datenerfassung muss die Zuordnung der Leistungen zu dem sie erbringenden Mitarbeiter eindeutig sein.
Der Leistungsnachweis bzw. das Durchführungskontrollblatt ist monatlich durch den/die Versicherte/n bzw. einer von ihm/ihr beauftragten Person zu bestätigen.
Eine Bestätigung im Voraus ist unzulässig. In Ausnahmefällen, die entsprechend zu kennzeichnen und zu begründen sind, bleibt das Unterschriftenfeld frei.
Der Leistungsnachweis ist als rechnungsbegründende Unterlage im Original den Krankenkassen zu übermitteln. Alternativ kann das Durchführungskontrollblatt in Kopie mit der Originalunterschrift des Versicherte/n bzw. einer von ihm/ihr beauftragten Person als rechnungsbegründende Unterlage den Krankenkassen übermittelt werden.

(4) Für die Abrechnung der Leistungen nach diesem Vertrag gilt die Richtlinie des GKV-Spitzenverbandes über Form und Inhalt des Abrechnungsverfahrens mit sonstigen Leistungserbringern nach § 302 Abs. 2 SGB V in der jeweils gültigen Fassung.[3]

(5) Nach § 303 Abs. 3 SGB V dürfen die Krankenkassen Abrechnungen der SAPV-Leistungserbringer nur vergüten, wenn die jeweils gesetzlich vorgeschriebenen Daten nach § 2 Abs. 1 Buchstabe a) (Abrechnungsdaten) und d) (Gesamtaufstellung der Abrechnung) der Abrechnungsrichtlinien im zuge-

[3] Umsetzung Abrechnungsverfahren nach § 302 SGB V, solange keine bundeseinheitl. Vorgaben f. DTA

lassenen Umfang maschinenlesbar oder auf maschinell verwertbaren Datenträgern angegeben oder übermittelt und die Daten nach Buchstaben b) (Urbelege) und d) (Leistungszusagen) sowie Leistungsnachweise in der dem SAPV-Leistungserbringer vorliegenden Form vorgelegt worden sind.

(6) Die Leistungserbringer sind verpflichtet,

a) in den Abrechnungsunterlagen die im Rahmen der multiprofessionellen Zusammenarbeit erbrachten Leistungen unter Angabe des jeweiligen Leistungserbringers nach Art, Menge und Preis einschließlich des Tagesdatums und des Zeitpunkts (z.B. morgens, mittags, abends) der Leistungserbringung aufzuzeichnen und zu übermitteln,

b) in den Abrechnungsunterlagen die Institutionskennzeichen (IK) der jeweils beteiligten Leistungserbringer aufzuzeichnen und zu übermitteln,

c) die KV-Nummer des/der Versicherten aufzuzeichnen und zu übermitteln.

(7) Die Krankenkasse ist verpflichtet, die bei ihnen oder von ihnen benannten Abrechnungsstellen eingereichten Rechnungen, innerhalb von 28 Tagen nach Zugang zu begleichen. Bei Zahlung durch Überweisung gilt die Frist als gewahrt, wenn der Auftrag innerhalb dieser Frist dem Geldinstitut erteilt wurde.

(8) Der SAPV-Leistungserbringer ist zu einer ordnungsgemäßen Abrechnung der erbrachten Leistungen gegenüber den Kostenträgern verpflichtet. Bei sachlichen oder rechnerischen Fehlern kann die Krankenkasse oder deren Abrechnungsstelle den fehlerhaften Teil der Rechnung beanstanden und zur Berichtigung zurückgeben. Im Übrigen bleibt die Verpflichtung zur fristgerechten Zahlung des nicht beanstandeten Teils der Rechnung bestehen.

(9) Beanstandungen müssen innerhalb von sechs Monaten nach Rechnungseingang geltend gemacht werden. Schadensersatzansprüche bleiben hiervon unberührt.

(10) Jeder neue Vertragspartner ist verpflichtet, sich vor der erstmaligen Datenlieferung bei der Datenstelle der Krankenkassen anzumelden. Dies gilt auch, wenn ein Abrechnungszentrum mit der Erstellung der Abrechnung beauftragt wurde.

(11) Die Rechnungstellung hat spätestens innerhalb von 12 Monaten zu erfolgen (Ausschlussfrist). Eine private Abrechnung dieser SAPV-Leistungen gegenüber dem Versicherten ist nicht möglich.

(12) Überträgt der SAPV-Leistungserbringer die Abrechnung einer Abrechnungsstelle, so hat er die Krankenkasse unverzüglich schriftlich zu informieren. Der Krankenkasse ist der Beginn und das Ende der Abrechnung und der Name der beauftragten Abrechnungsstelle mitzuteilen. Es ist eine Erklärung des SAPV-Leistungserbringers beizufügen, dass die Zahlungen der Krankenkasse an die beauftragte Abrechnungsstelle mit schuldbefreiender Wirkung erfolgt. Der SAPV-Leistungserbringer ist verpflichtet, selbst dafür zu sorgen, dass mit dem der Krankenkasse mitgeteilten Ende der Abrechnung keine diesen Zeitpunkt überschreitende Inkassovollmacht oder Abtretungserklärung zugunsten der der Krankenkasse gemeldeten Abrechnungsstelle mehr besteht. Das Abrechnungszentrum ist verpflichtet, sich ebenfalls gem. Abs. 4 zum maschinellen Datenaustausch anzumelden. Abrechnungszentren liefern die Abrechnung ausschließlich auf maschinell verwertbaren Datenträgern.

(13) Sofern die Rechnungslegung einer Abrechnungsstelle gemäß Absatz 12 übertragen werden soll, ist der Auftragnehmer unter besonderer Berücksichtigung der von ihm getroffenen technischen und organisatorischen Maßnahmen zur Sicherstellung der Maßgaben dieses Vertrages und des § 6 Abs. 1 BDSG durch den SAPV-Leistungserbringer auszuwählen. Die getroffene Vereinbarung über Datenschutz und Datensicherung beim Auftragnehmer (Abrechnungsstelle) ist der Krankenkasse vorzulegen.

Muster

## § 14
## Institutionskennzeichen

(1) Der SAPV-Leistungserbringer verfügt gemäß § 293 SGB V über ein Institutionskennzeichen (IK), das er bei der Abrechnung mit den Krankenkassen verwendet.

Das IK ist vom SAPV-Leistungserbringer bei der Sammel- und Verteilungsstelle IK der Arbeitsgemeinschaft Institutionskennzeichen (SVI), Alte Heerstr. 111, 53757 St. Augustin, zu beantragen.

Änderungen der unter dem IK gespeicherten Daten sind der SVI unverzüglich mitzuteilen. Mitteilungen an die Krankenkassen oder ihre mit der Abrechnungsprüfung beauftragten Dienstleister werden nicht berücksichtigt.

(2) Das gegenüber den Krankenkassen eingesetzte IK ist den jeweiligen Krankenkassen sowohl bei Vertragsabschluss als auch bei Änderungen mitzuteilen. Abrechnungen mit den Krankenkassen erfolgen ausschließlich unter diesem IK.

Das IK ist in jeder Abrechnung anzugeben. Abrechnungen ohne IK oder mit fehlerhaftem IK werden von den Krankenkassen abgewiesen. Gleiches gilt für Abrechnungen mit einem der Krankenkasse unbekannten IK.

Die unter dem gegenüber den Krankenkassen verwandten IK bei der SVI gespeicherten Angaben, einschließlich der Bank- und Kontoverbindung sind verbindlich für die Abrechnungsbegleichung durch die Krankenkassen. Andere Bank- und Kontoverbindungen werden von den Krankenkassen bei der Abrechnung nicht berücksichtigt.

Muster

## § 15
## Datenschutz / Schweigepflicht

(1) Der SAPV-Leistungserbringer ist verpflichtet, die Bestimmungen über den Schutz der Sozialdaten (SGB X 2. Kapitel) zu beachten, insbesondere personenbezogene Daten nur zur Erfüllung der sich aus diesem Vertrag ergebenden Aufgaben zu verarbeiten, bekanntzugeben, zugänglich zu machen oder sonst zu nutzen.

(2) Der SAPV-Leistungserbringer unterliegt hinsichtlich der Person des Versicherten und dessen Krankheiten der Schweigepflicht. Ausgenommen hiervon sind Angaben gegenüber der Krankenkasse, soweit sie zur Erfüllung der gesetzlichen Aufgaben der Krankenkassen erforderlich sind. Ausgenommen sind außerdem Angaben gegenüber den übrigen an der Versorgung Beteiligten im Rahmen ihrer Tätigkeit im Sinne dieses Vertrags.

(3) Der SAPV-Leistungserbringer hat seine Mitarbeiter zur Beachtung der Datenschutzbestimmungen und der Schweigepflicht zu verpflichten.

(4) Der SAPV-Leistungserbringer sichert Datentransparenz gegenüber der Krankenkasse im Rahmen der rechtlichen Grundlagen zum Zwecke der Abrechnung und Versorgungsevaluation sowie -analyse zu.

(5) Versicherte, die an der spezialisierten ambulanten Palliativversorgung teilnehmen, bzw. deren gesetzliche Betreuer sind über die Inhalte der spezialisierten ambulanten Palliativversorgung ausführlich zu informieren und haben ihr Einverständnis zur Teilnahme an dieser Versorgungsform schriftlich zu erklären.

(6) Mit der Erklärung gemäß Anlage 7 können Versicherte die an der jeweiligen spezialisierten ambulanten Palliativversorgung beteiligten Leistungserbringer von der Schweigepflicht entbinden, soweit dies im Rahmen dieser Versorgung erforderlich ist und erteilen ihre Einwilligung zur Datenerhebenung / -verarbeitung / -nutzung zum Zwecke der Evaluation und ggf. späteren Veröffentlichung von anonymisierten Evaluationsergebnissen.

## § 16
## Vertragsverstöße

(1) Bei Verstößen einzelner SAPV-Leistungserbringer gegen Vertragspflichten kann die Krankenkasse eine Verwarnung bzw. Abmahnung aussprechen. Im Falle von schweren Vertragsverstößen kann die Krankenkasse dem jeweiligen SAPV-Leistungerbringer fristlos kündigen.

(2) Als schwere Vertragsverstöße gelten insbesondere

a) grobe Verletzungen der gesetzlichen oder vertraglichen Verpflichtungen gegenüber den Patienten oder der Krankenkasse, so dass ein Festhalten an dem Vertrag nicht zumutbar ist, dies gilt insbesondere dann,

- wenn ein Patient infolge grober Pflichtverletzung gesundheitlichen Schaden erleidet bzw. durch eine grobe Pflichtverletzung konkrete gesundheitliche Schädigung droht oder
- wenn verordnete Leistungen nicht erbracht und gegenüber der Krankenkasse abgerechnet werden oder
- wenn der Leistungserbringer Aufträge annimmt und gegen Entgelt oder zur Erlangung anderer geldwerter Vorteile an Dritte (Vermittlung) weitergibt,
- wenn nicht nur vorübergehend (länger als 3 Monate) die fachlichen und/oder personellen Voraussetzungen gemäß dieses Vertrages nicht erfüllt werden. In besonderen begründeten Ausnahmefällen kann die Krankenkasse von Sanktionen absehen.
- wenn Straftaten vorliegen, die einer Tätigkeit im Bereich der SAPV entgegenstehen.

b) Forderungen bzw. Annahme von Zahlungen für vertragsärztlich verordnete und von der Krankenkasse genehmigte Leistungen von Versicherten.

Muster

## § 17
## Schlussbestimmungen

(1) Änderungen dieses Vertrages bedürfen der Schriftform. Sind einzelne Bestimmungen dieses Vertrages nichtig, so bleibt hiervon die Wirksamkeit des übrigen Vertrages unberührt. Nichtige Vertragsbestimmungen sind unter Wahrung des Grundsatzes der Vertragstreue neu zu regeln.

(2) Sollten einzelne Regelungen dieses Vertrags unwirksam sein oder unwirksam werden, wird die Wirksamkeit der übrigen Regelungen nicht berührt. Die Vertragspartner sind sich darüber einig, dass in einem solchen Fall, die unwirksame Bestimmung durch eine dieser in Interessenlage und Bedeutung (Regelungsgehalt und -intention) möglichst nahekommende Regelung zu ersetzen ist.

## § 18
## Inkrafttreten und Kündigung

(1) Dieser Vertrag tritt am XX in Kraft.

(2) Der Vertrag kann von jedem Vertragspartner mit einer Frist von 3 Monaten zum Ende eines Kalendervierteljahres schriftlich gekündigt werden.

(3) Sollten einzelne Bestimmungen dieses Vertrages unwirksam sein oder unwirksam werden, wird die Wirksamkeit der übrigen Regelungen nicht berührt. Dies gilt auch, wenn Gesetzesänderungen auf Inhalte dieses Vertrages einwirken. Die Vertragsparteien verpflichten sich für diesen Fall, unverzüglich in Verhandlungen über einen neuen Vertrag bzw. neue einzelne Bestimmungen einzutreten.

Eisenberg/Mainz/Saarbrücken/Speyer/xxx,

SAPV-Leistungserbringer

. . . . . . . . . . . . . . . . . . . . . . . . . . .

AOK – Die Gesundheitskasse in Rheinland-Pfalz

. . . . . . . . . . . . . . . . . . . . . . . . . . .
Walter Bockemühl
Vorstandsvorsitzender

Verband der Ersatzkassen e.V. (vdek),

. . . . . . . . . . . . . . . . . . . . . . . . . . .
Leiter der vdek-Landesvertretung Rheinland-Pfalz

BKK-Landesverband
Rheinland-Pfalz und Saarland

. . . . . . . . . . . . . . . . . . . . . . . . . . .
Raimund Nossek
Vorstand

Knappschaft,
Regionaldirektion Saarbrücken

. . . . . . . . . . . . . . . . . . . . . . . . . . .
Armin Beck
Leiter der Regionaldirektion

IKK Südwest

. . . . . . . . . . . . . . . . . . . . . . . . . . .
Frank Spaniol
Vorstand

Landwirtschaftliche Krankenkasse
Hessen, Rheinland-Pfalz und
Saarland

. . . . . . . . . . . . . . . . . . . . . . . . . . .

Muster

*Anlage 1 – Richtlinie des Gemeinsamen Bundesausschusses*
*Stand: 12. März 2008*

**Richtlinie**
**des Gemeinsamen Bundesausschusses**

**zur Verordnung von spezialisierter ambulanter**
**Palliativversorgung**

**(Spezialisierte Ambulante Palliativversorgungs-Richtlinie / SAPV-RL)**

vom 20. Dezember 2007,
veröffentlicht im Bundesanzeiger 2008, S. 911,
in Kraft getreten am 12. März 2008

**i.d.F. ab 16.04.2010**

## Inhalt

| | | |
|---|---|---|
| § 1 | Grundlagen und Ziele | 3 |
| § 2 | Anspruchsvoraussetzungen | 3 |
| § 3 | Anforderungen an die Erkrankungen | 3 |
| § 4 | Besonders aufwändige Versorgung | 4 |
| § 5 | Inhalt und Umfang der spezialisierten ambulanten Palliativversorgung | 4 |
| § 6 | Zusammenarbeit der Leistungserbringer | 5 |
| § 7 | Verordnung von SAPV | 6 |
| § 8 | Prüfung der Leistungsansprüche durch die Krankenkasse | 6 |

## § 1 Grundlagen und Ziele

(1) Die spezialisierte ambulante Palliativversorgung gemäß § 37b SGB V (SAPV) dient dem Ziel, die Lebensqualität und die Selbstbestimmung schwerstkranker Menschen zu erhalten, zu fördern und zu verbessern und ihnen ein menschenwürdiges Leben bis zum Tod in ihrer vertrauten häuslichen oder familiären Umgebung zu ermöglichen. Im Vordergrund steht anstelle eines kurativen Ansatzes die medizinisch-pflegerische Zielsetzung, Symptome und Leiden einzelfallgerecht zu lindern

(2) SAPV kann im Haushalt des schwerstkranken Menschen oder in seiner Familie oder in stationären Pflegeeinrichtungen (§72 Abs. 1 des Elften Buches Sozialgesetzbuch - SGB XI) erbracht werden. Darüber hinaus kann SAPV auch erbracht werden

- in Einrichtungen der Eingliederungshilfe für behinderte Menschen im Sinne von § 55 SGB XII und der Kinder- und Jugendhilfe im Sinne von § 34 SGB VIII
- an weiteren Orten, an denen
  - sich der schwerstkranke Mensch in vertrauter häuslicher oder familiärer Umgebung dauerhaft aufhält und
  - diese versorgung zuverlässig erbracht werden kann

wenn und soweit nicht andere Leistungsträger zur Leistung verpflichtet sind.

(3) In stationären Hospizen besteht ein Anspruch auf die Teilleistung der erforderlichen ärztlichen Versorgung im Rahmen der SAPV, wenn die ärztliche Versorgung im Rahmen der vertragsärztlichen Versorgung aufgrund des besonders aufwändigen Versorgungsbedarfs (siehe § 4) nicht ausreicht.

(4) Den besonderen Belangen von Kindern ist Rechnung zu tragen.

(5) Die individuellen Bedürfnisse und Wünsche der Patientin oder des Patienten sowie die Belange ihrer oder seiner vertrauten Personen stehen im Mittelpunkt der Versorgung. 2Der Patientenwille, der auch durch Patientenverfügungen zum Ausdruck kommen kann, ist zu beachten.

(6) Die SAPV ergänzt das bestehende Versorgungsangebot, insbesondere das der Vertragsärzte, Krankenhäuser und Pflegedienste. Sie kann als alleinige Beratungsleistung, additiv unterstützende Teilversorgung oder vollständige Patientenbetreuung erbracht werden. Andere Sozialleistungsansprüche bleiben unberührt.

## § 2 Anspruchsvoraussetzungen

Versicherte haben Anspruch auf SAPV, wenn

- sie an einer nicht heilbaren, fortschreitenden und so weit fortgeschrittenen Erkrankung leiden, dass dadurch ihre Lebenserwartung begrenzt ist (§ 3) und
- sie unter Berücksichtigung der in § 1 genannten Ziele eine besonders aufwändige Versorgung (§ 4) benötigen, die nach den medizinischen und pflegerischen Erfordernissen auch ambulant oder an den in § 1 Abs. 2 und 3 genannten Orten erbracht werden kann.

## § 3 Anforderungen an die Erkrankungen

(1) Eine Erkrankung ist nicht heilbar, wenn nach dem allgemein anerkannten Stand der medizinischen Erkenntnisse Behandlungsmaßnahmen nicht zur Beseitigung dieser Erkrankung führen können.

(2) Sie ist fortschreitend, wenn ihr Verlauf trotz medizinischer Maßnahmen nach dem allgemein anerkannten Stand der medizinischen Erkenntnisse nicht nachhaltig aufgehalten werden kann.

(3) Eine Erkrankung ist weit fortgeschritten, wenn die Verbesserung von Symptomatik und Lebensqualität sowie die psychosoziale Betreuung im Vordergrund der Versorgung stehen und nach begründeter Einschätzung der verordnenden Ärztin oder des verordnenden Arztes die Lebenserwartung auf Tage, Wochen oder Monate gesunken ist. Insbesondere bei Kindern sind die Voraussetzungen für die SAPV als Krisenintervention auch bei einer länger prognostizierten Lebenserwartung erfüllt.

## § 4 Besonders aufwändige Versorgung

Bedarf nach einer besonders aufwändigen Versorgung besteht, soweit die anderweitigen ambulanten Versorgungsformen sowie ggf. die Leistungen des ambulanten Hospizdienstes nicht oder nur unter besonderer Koordination ausreichen würden, um die Ziele nach § 1 Abs. 1 zu erreichen. Anhaltspunkt dafür ist das Vorliegen eines komplexen Symptomgeschehens, dessen Behandlung spezifische palliativmedizinische und / oder palliativpflegerische Kenntnisse und Erfahrungen sowie ein interdisziplinär, insbesondere zwischen Ärzten und Pflegekräften in besonderem Maße abgestimmtes Konzept voraussetzt. Ein Symptomgeschehen ist in der Regel komplex, wenn mindestens eines der nachstehenden Kriterien erfüllt ist:

- ausgeprägte Schmerzsymptomatik
- ausgeprägte neurologische / psychiatrische / psychische Symptomatik
- ausgeprägte respiratorische / kardiale Symptomatik
- ausgeprägte gastrointestinale Symptomatik
- ausgeprägte ulzerierende / exulzerierende Wunden oder Tumore
- ausgeprägte urogenitale Symptomatik

## § 5 Inhalt und Umfang der spezialisierten ambulanten Palliativversorgung

(1) Die SAPV umfasst je nach Bedarf alle Leistungen der ambulanten Krankenbehandlung soweit diese erforderlich sind, um die in § 1 Abs. 1 genannten Ziele zu erreichen. Sie umfasst zusätzlich die im Einzelfall erforderliche Koordination der diagnostischen, therapeutischen und pflegerischen Teilleistungen sowie die Beratung, Anleitung und Begleitung der verordnenden oder behandelnden Ärztin oder des verordnenden oder behandelnden Arztes sowie der sonstigen an der allgemeinen Versorgung beteiligten Leistungserbringer, der Patienten und ihrer Angehörigen durch Leistungserbringer nach § 132d SGB V.

(2) SAPV wird ausschließlich von Leistungserbringern nach § 132d SGB V erbracht, die in einer interdisziplinären Versorgungsstruktur, bestehend insbesondere aus qualifizierten Ärzten und Pflegefachkräften unter Beteiligung der ambulanten Hospizdienste und ggf. der stationären Hospize, organisiert sind. Sie wird nach Bedarf intermittierend oder durchgängig erbracht, soweit das bestehende ambulante Versorgungsangebot (§ 1 Abs. 4), insbesondere die allgemeine Palliativversorgung nicht ausreicht, um die Ziele nach § 1 Abs. 1 zu erreichen. Sie kann dem jeweiligen aktuellen Versorgungsbedarf entsprechend als

- Beratungsleistung,
- Koordination der Versorgung,
- additiv unterstützende Teilversorgung
- vollständige Versorgung
- erbracht werden. Die Leistungen müssen ausreichend und zweckmäßig sein, dürfen das Maß des Notwendigen nicht überschreiten und sind wirtschaftlich zu erbringen.

(3) Inhalte der SAPV sind insbesondere:

- Koordination der spezialisierten palliativmedizinischen und palliativpflegerischen Versorgung unter Einbeziehung weiterer Berufsgruppen und von Hospizdiensten im Rahmen einer multiprofessionellen Zusammenarbeit
- Symptomlinderung durch Anwendung von Medikamenten oder anderen Maßnahmen
- apparative palliativmedizinische Behandlungsmaßnahmen (z. B. Medikamentenpumpe)
- palliativmedizinische Maßnahmen, die nach ihrer Art, Schwere oder Komplexität eine

Kompetenz erfordern, die der einer Ärztin oder eines Arztes mit Zusatzweiterbildung Palliativmedizin entspricht

- spezialisierte palliativpflegerische Leistungen, die nach ihrer Art, Schwere oder Komplexität eine Kompetenz erfordern, die der einer Pflegefachkraft mit einer curricularen Weiterbildung zu Palliative Care entspricht
- Führung eines individuellen Behandlungsplans, vorbeugendes Krisenmanagement, Bedarfsinterventionen
- Ruf-, Notfall- und Kriseninterventionsbereitschaft rund um die Uhr für die im Rahmen der SAPV betreuten Patienten zur Sicherstellung der im Rahmen der SAPV erforderlichen Maßnahmen
- Beratung, Anleitung und Begleitung der Patienten und ihrer Angehörigen zur palliativen Versorgung einschließlich Unterstützung beim Umgang mit Sterben und Tod
- spezialisierte Beratung der betreuenden Leistungserbringer der Primärversorgung
- psychosoziale Unterstützung im Umgang mit schweren Erkrankungen in enger Zusammenarbeit z. B. mit Seelsorge, Sozialarbeit und ambulanten Hospizdiensten
- Organisation regelmäßiger Fallbesprechungen
- Dokumentieren und Evaluieren der wesentlichen Maßnahmen im Rahmen der SAPV

## § 6 Zusammenarbeit der Leistungserbringer

(1) Im Rahmen der SAPV ist zu gewährleisten, dass die an der Versorgung beteiligten Leistungserbringer die erforderlichen Maßnahmen aufeinander abgestimmt und bedarfsgerecht erbringen; die diesbezügliche Koordination ist sicherzustellen. Hierüber sind verbindliche Kooperationsvereinbarungen schriftlich oder mündlich zu treffen.
Kooperationspartner ist auch der ambulante Hospizdienst, der auf Wunsch der Patientin oder des Patienten an der Versorgung beteiligt wird.
Bei Bedarf und entsprechender Qualifikation kann die dauerbehandelnde Ärztin oder der dauerbehandelnde Arzt im Einzelfall Kooperationspartnerin oder Kooperationspartner werden. Das Nähere regeln die Verträge nach § 132d SGB V.

(2) Die vorhandenen Versorgungsstrukturen sind zu beachten.

(3) Es ist zu gewährleisten, dass zwischen den an der Patientenversorgung beteiligten Leistungserbringern zeitnah alle notwendigen Informationen über die vorhergehende Behandlung unter Berücksichtigung datenschutzrechtlicher Regelungen ausgetauscht werden.

(4) Bei der SAPV ist der ärztlich und pflegerisch erforderliche Entscheidungsspielraum für die Anpassung der Palliativversorgung an die Besonderheiten des Einzelfalls zu berücksichtigen.

(5) Für die notwendigen koordinativen Maßnahmen ist vernetztes Arbeiten innerhalb der gewachsenen Strukturen der Palliativversorgung unabdingbar. Dieses ist unter Berücksichti-

gung medizinischer, pflegerischer, physiotherapeutischer, psychologischer, psychosozialer und spiritueller Anforderungen zur lückenlosen Versorgung über die Sektorengrenzen hinweg zu fördern und auszubauen.

## § 7 Verordnung von SAPV

(1) SAPV wird von der behandelnden Vertragsärztin oder von dem behandelnden Vertragsarzt nach Maßgabe dieser Richtlinie verordnet. Satz 1 gilt für die Behandlung durch die Krankenhausärztin oder den Krankenhausarzt bei einer oder einem von ihr oder ihm ambulant versorgten Patientin oder Patienten entsprechend. Hält eine Krankenhausärztin oder ein Krankenhausarzt die Entlassung einer Patientin oder eines Patienten für möglich und ist aus ihrer oder seiner Sicht SAPV erforderlich, kann die Krankenhausärztin oder der Krankenhausarzt die Verordnung ausstellen, in der Regel jedoch längstens für 7 Tage.

(2) Die ärztliche Verordnung erfolgt auf einem zu vereinbarenden Vordruck, der der Leistungserbringung nach dem jeweiligen aktuellen Versorgungsbedarf (§ 5 Abs. 2) Rechnung zu tragen hat und Angaben zur Dauer der Verordnung enthält.

## § 8 Prüfung der Leistungsansprüche durch die Krankenkasse

Die Krankenkasse übernimmt bis zu einer Entscheidung über die weitere Leistungserbringung die Kosten für die verordneten und von den Leistungserbringern nach § 132d SGB V erbrachten Leistungen entsprechend der vereinbarten Vergütung nach § 132d SGB V, wenn die Verordnung gemäß § 7 Abs. 2 spätestens an dem dritten der Ausstellung folgenden Arbeitstag der Krankenkasse vorgelegt wird. Das Nähere regeln die Vertragspartner nach § 132d SGB V.

Die SAPV-Richtlinie tritt am Tag nach der Veröffentlichung im Bundesanzeiger in Kraft.

Siegburg, den 20. Dezember 2007

Gemeinsamer Bundesausschuss

Der Vorsitzende

Hess

*Anlage 2 – Empfehlungen der Spitzenverbände*
*Stand: 23. Juni 2008*

**Gemeinsame Empfehlungen**

des AOK-Bundesverbandes, Bonn,
des BKK Bundesverbandes, Essen,
des Bundesverbandes der landwirtschaftlichen Krankenkassen, Kassel,
des IKK-Bundesverbandes, Bergisch Gladbach,
der Knappschaft, Bochum,
des Verbandes der Angestellten-Krankenkassen e.V., Siegburg,
des AEV Arbeiter-Ersatzkassen-Verband e.V., Siegburg

**nach § 132d Abs.2 SGB V**
**für die spezialisierte ambulante Palliativversorgung**
**vom 23.06.2008**

unter Beteiligung

des Arbeiterwohlfahrt Bundesverbandes e.V., Berlin,
des Arbeitgeber- und BerufsVerbandes Privater Pflege e.V., Hannover,
der Bundesarbeitsgemeinschaft Hauskrankenpflege e.V., Berlin,
der Bundesarbeitsgemeinschaft der PatientInnenstellen, München,
des Bundesverbandes Ambulante Dienste und Stationäre Einrichtungen e.V., Essen,
der Bundesinitiative Ambulante Psychiatrische Pflege e.V., Berlin,
des Bundesverbandes Deutscher Privatkliniken e.V. Berlin,
des Bundesverbandes Häusliche Kinderkrankenpflege e.V., Köln,
des Bundesverbandes Kinderhospiz e.V., Freiburg,
des Bundesverbandes privater Anbieter sozialer Dienste e.V., Berlin,
der Deutschen Arbeitsgemeinschaft Selbsthilfegruppen e.V., Gießen,
des Deutschen Bundesverbandes für Pflegeberufe, Berlin,
des Deutschen Caritasverbandes e.V., Freiburg,
der Deutschen Gesellschaft für Palliativmedizin e.V., Berlin,
der Deutschen Hospiz Stiftung, Dortmund,
des Deutschen Hospiz- und PalliativVerbandes e.V., Berlin,
des Deutschen Kinderhospizvereines e.V., Olpe,
der Deutschen Krankenhaus-Gesellschaft, Berlin,
des Deutschen Paritätischen Wohlfahrtsverbandes, Gesamtverband e.V., Berlin,
des Deutschen Pflegerates, Berlin,
des Deutschen Roten Kreuzes, Generalsekretariat, Berlin,
des Diakonischen Werkes der EKD e.V., Berlin,
der Kassenärztlichen Bundesvereinigung, Berlin,
des Sozialverbandes VdK Deutschland e.V., Berlin,
des Verbandes Deutscher Alten- und Behinderten Hilfe e.V., Essen,
der Verbraucherzentrale Bundesverband e.V., Berlin,
die Zentralwohlfahrtsstelle der deutschen Juden in Deutschland e.V., Frankfurt am Main

**1. Zielsetzung**

1.1 Die spezialisierte ambulante Palliativversorgung (SAPV) dient dem Ziel, die Lebensqualität und die Selbstbestimmung schwerstkranker Menschen zu erhalten, zu fördern und zu verbessern und ihnen ein menschenwürdiges Leben bis zum Tod in ihrer vertrauten häuslichen Umgebung oder in einer stationären Pflegeeinrichtung zu ermöglichen.

1.2 Die SAPV ist fachlich kompetent nach den allgemein anerkannten medizinischen und pflegewissenschaftlichen Erkenntnissen bedarfsgerecht und wirtschaftlich zu erbringen. Die individuellen Bedürfnisse und Wünsche der Patientin oder des Patienten sowie die Belange ihrer oder seiner vertrauten Personen stehen im Mittelpunkt der Versorgung

1.3 Die Spitzenverbände der Krankenkassen werden die Erfahrungen mit der Umsetzung dieser Empfehlungen fortlaufend auswerten und diese erforderlichenfalls weiterentwickeln.

**2. Zulassungsvoraussetzungen**

2.1 SAPV wird von Leistungserbringern erbracht, mit denen die Krankenkasse zur Sicherung einer bedarfsgerechten Versorgung einen Vertrag geschlossen hat. Sie wird intermittierend oder durchgängig nach Bedarf als

- Beratungsleistung,
- Koordination der Versorgung,
- additiv unterstützende Teilversorgung,
- vollständige Versorgung

erbracht. Den besonderen Belangen von Kindern und Jugendlichen ist Rechnung zu tragen.

2.2 Bei der Entwicklung einer bedarfsgerechten Versorgung sind die bereits bestehenden Strukturen so weit wie möglich einzubeziehen. Auf die bereits tätigen Leistungserbringer ist zurückzugreifen, soweit sie die erforderlichen Anforderungen erfüllen. Verträge sind nur in dem Umfang abzuschließen, wie sie für eine bedarfsgerechte Versorgung erforderlich sind. Ein Anspruch auf Vertragsabschluss besteht nicht.

2.3 Eine bedarfsgerechte Versorgung mit SAPV ist insbesondere dann gegeben, wenn sie wohnortnah ausgerichtet ist und die Palliativpatienten, die einen besonderen Versorgungsbedarf haben, der durch die allgemeine Palliativversorgung nicht gewährleistet werden kann, ausreichend und zweckmäßig mit der Leistung der SAPV versorgt werden können. Die bedarfsgerechte Versorgung zeigt sich auch darin, dass es mit der Leistung der SAPV mehr Menschen als bisher ermöglicht wird, in ihrer vertrauten häuslichen Umgebung oder in stationären Pflegeeinrichtungen ein menschenwürdiges Leben bis zum Tod zu führen.

2.4 Der Versorgungsbedarf kann in einzelnen Regionen unterschiedlich sein. Indikatoren für die Be-

darfslage können insbesondere sein,

- die regionale Siedlungsstruktur,
- die Altersstruktur,
- epidemiologisch relevante Erkrankungen sowie
- die demografische Entwicklung.

So ist zwischen Regionen geringer, mittlerer und hoher Bevölkerungsdichte zu unterscheiden. Die Unterscheidung ist ggf. nicht allein auf der Ebene der Bundesländer durchzuführen, sondern kann bis auf die Ebene der Kreise und kreisfreien Städte vollzogen werden, um dem erheblichen Unterschied in der Bevölkerungsdichte auch innerhalb der Bundesländer gerecht zu werden. Nach derzeit vorliegenden Schätzungen haben bis zu 10 Prozent aller Sterbenden einen solchen besonderen Versorgungsbedarf, der im Rahmen der SAPV abzudecken ist.

**3. Inhalt und Umfang der Leistungen**

3.1 Inhalt und Umfang der zu erbringenden SAPV-Leistungen ergeben sich aus der gemäß der SAPV-Richtlinie nach § 37b SGB V in Verbindung mit § 92 Abs.1 Satz 2 Nr. 14 SGB V ausgestellten und genehmigten Verordnung. Die Krankenkasse übernimmt bis zu einer Entscheidung über die weitere Leistungserbringung die Kosten für die verordneten und von den Leistungserbringern nach § 132d SGB V erbrachten Leistungen entsprechend der vereinbarten Vergütung nach § 132d SGB V, wenn die Verordnung gemäß § 7 Abs. 2 der SAPV-Richtlinie spätestens an dem dritten der Ausstellung folgenden Arbeitstag der Krankenkasse vorgelegt wird.

3.2 Die Leistungen müssen ausreichend und zweckmäßig sein, dürfen das Maß des Notwendigen nicht überschreiten und sind wirtschaftlich zu erbringen. Soweit die allgemeine Palliativversorgung ausreichend ist, darf SAPV nicht erbracht werden.

3.3 Im Falle der Besserung bzw. einer Stabilisierung im Krankheitsverlauf ist der Versorgungsumfang der SAPV so weit wie möglich zu reduzieren und eine Weiterversorgung im Rahmen der allgemeinen Palliativversorgung anzustreben.

3.2 In den Vereinbarungen über die SAPV sind Regelungen zu treffen, die sicherstellen, dass für den Palliativpatienten eine reibungslose Versorgung mit Arznei- Heil- und Hilfsmitteln auch weiterhin gewährleistet ist.

**4. Organisatorische Voraussetzungen**

4.1 Die Leistungen der SAPV werden durch spezialisierte Leistungserbringer erbracht. Spezialisierte Leistungserbringer sind nach Nr. 5.2 qualifizierte Ärztinnen oder Ärzte und nach Nr. 5.3 qualifizierte Pflegefachkräfte, die orientiert an der Konzeption eines Palliativ-Care-Teams fachübergreifend ggf. auch in Kooperation mit anderen Professionen (s. Nr. 5.4) eng zusammenarbeiten. Sie erfüllen ergänzend besondere sächliche Voraussetzungen nach Nr. 4.4 und Nr. 4.5, die für eine spe-

zialisierte palliativmedizinische und palliativpflegerische Versorgung erforderlich sind. Eine psychosoziale Unterstützung ist in enger Zusammenarbeit z.B. mit ambulanten Hospizdiensten nach § 39a Abs. 2 SGB V, Seelsorge und Sozialarbeit zu gewährleisten.

4.2 Die spezialisierten Leistungserbringer sind Teil einer multiprofessionell vernetzten Versorgungsstruktur im regionalen Gesundheits- und Sozialsystem. Sie arbeiten mit den an der Versorgung beteiligten Leistungserbringern, wie Hausärzten und ambulanten Hospizdiensten gemäß § 39a Abs. 2 SGB V eng zusammen (integrativer Ansatz). Mit den regelhaft an der Versorgung beteiligten Leistungserbringern sind Kooperationsvereinbarungen schriftlich abzuschließen und der Krankenkasse vorzulegen.

4.3 Die spezialisierten Leistungserbringer arbeiten nach einem verbindlichen, strukturierten und schriftlich dargelegten Konzept, in dem der inhaltliche und organisatorische Rahmen der Leistungserbringung (inkl. der personellen und sächlichen Ausstattung) sowie die Einbindung in die regionale Versorgungsstruktur beschrieben sind. Das Konzept ist der Krankenkasse vorzulegen.

4.4 Die spezialisierten Leistungserbringer haben als Mindestanforderung an die sächliche Ausstattung Folgendes vorzuhalten bzw. sicherzustellen:

- eine geeignete, aktuell geführte und für die an der Versorgung
- Beteiligten jederzeit zugängliche Patientendokumentation
- Arzneimittel (inkl. BtM) für die Notfall/Krisenintervention
- Arzt-/Pflegekoffer/Bereitschaftstasche (Berücksichtigung der Kompatibilität der Verbrauchsmaterialien zu Medizinprodukten unterschiedlicher Hersteller, z.B. bei Portsystemen oder Infusionspumpen)
- eine geeignete administrative Infrastruktur, z. B. Büro, Kommunikationstechnik.

4.5 Die spezialisierten Leistungserbringer müssen über eine eigenständige Adresse und geeignete Räumlichkeiten für

- die Beratung von Patienten und Angehörigen
- Teamsitzungen und Besprechungen
- die Lagerhaltung von eigenen Medikamenten für Notfall- / Krisenintervention
- und Hilfsmitteln

verfügen. Sofern eine Aufbewahrung von Medikamenten erfolgt, die unter das Betäubungsmittelgesetz (BtM-G) fallen, ist ein BtM-Schrank (§ 15 BtM-G) erforderlich

**5. Personelle Anforderungen**

5.1 Die spezialisierten Leistungserbringer stellen sicher, dass das für die spezialisierte ambulante Palliativversorgung erforderliche und geeignete Personal zur Verfügung steht. Dabei ist zu berücksichtigen, dass durch den spezialisierten Leistungserbringer eine tägliche telefonische Rund-um-die-Uhr-Erreichbarkeit für die Patienten, deren Angehörigen und die an der Versorgung Beteiligten sicherzustellen ist. Die ständige Verfügbarkeit mindestens einer Ärztin/eines Arztes und/oder einer Pflegefachkraft ist zu gewährleisten. Die Verfügbarkeit schließt notwendige Hausbesuche ein.

5.2 Die nach Nr. 4.1 tätigen Ärztinnen und Ärzte verfügen über

- eine anerkannte Zusatzweiterbildung Palliativmedizin nach der aktuell gültigen Weiterbildung der jeweiligen Landesärztekammer (grds. 160 Std. Weiterbildung)

und

- Erfahrung aus der ambulanten palliativen Behandlung von mindestens 75 Palliativpatienten, z. B. in der häuslichen Umgebung oder in einem stationären Hospiz, innerhalb der letzten drei Jahre oder aus einer mindestens einjährigen klinischen palliativmedizinischen Tätigkeit in einer Palliativabteilung in einem Krankenhaus innerhalb der letzten drei Jahre

5.3 Die nach Nr. 4.1 tätigen Pflegefachkräfte verfügen über:

- die Erlaubnis zur Führung einer der Berufsbezeichnungen Gesundheits- undKrankenpflegerin / Gesundheits- und Krankenpfleger, Gesundheits- und Kinderkrankenpflegerin / Gesundheits- und Kinderkrankenpfleger oder Altenpflegerin / Altenpfleger (dreijährige Ausbildung aufgrund einer landesrechtlichen Regelung) entsprechend den gesetzlichen Bestimmungen in der jeweils gültigen Fassung und

- den Abschluss einer Palliative-Care-Weiterbildungsmaßnahme im Umfang von mindestens 160 Stunden

und

- Erfahrung durch mindestens eine zweijährige praktische Tätigkeit als Pflegefachkraft in der Betreuung von Palliativpatienten in den letzten drei Jahren; davon mindestens sechsmonatige Mitarbeit in einer spezialisierten Einrichtung der Hospiz- und Palliativversorgung.

5.4 Soweit weitere Fachkräfte (z.B. Sozialarbeiterinnen / Sozialarbeiter, Sozialpädagoginnen oder Sozialpädagogen, Psychologinnen oder Psychologen) vertraglich eingebunden werden, haben diese eine Zusatzweiterbildung Palliative Care für andere Berufsgruppen oder eine mehrjährige Erfahrung in der Palliativversorgung nachzuweisen.

**6. Qualitätssicherung**

6.1 Die spezialisierten Leistungserbringer sind verpflichtet, ein internes Qualitätsmanagement durchzuführen. Sie nehmen regelmäßig an palliativmedizinischen/-pflegerischen Fortbildungen teil und führen möglichst halbjährlich multidisziplinäre Qualitätszirkel durch, an denen auch die übrigen in der Versorgung Tätigen teilnehmen. Die spezialisierten Leistungserbringer sollen sich außerdem an Maßnahmen der externen Qualitätssicherung beteiligen.

6.2 Ein geeignetes Dokumentationssystem ist sachgerecht und kontinuierlich zu führen und auf Wunsch der Krankenkasse als Konzept vorzulegen. Das Dokumentationssystem muss patientenbezogene Daten und - soweit vorhanden - allgemein anerkannte Indikatoren für eine externe Qualitätssicherung enthalten und eine bundesweite Evaluation ermöglichen. Es soll kompatibel zu den bestehenden Dokumentationssystemen der an der Versorgung beteiligten Leistungserbringer sein.

6.3 Die Teilnahme an Supervision ist zu ermöglichen. Regelmäßige multiprofessionelle Fallbesprechungen sind in überschaubaren Intervallen durchzuführen; die inhaltliche und zeitliche Organisation ist den Beteiligten überlassen.

6.4 Die spezialisierten Leistungserbringer erstellen einen jeweils individuellen Behandlungsplan, der mit den übrigen an der Versorgung beteiligten Leistungserbringern abzustimmen ist.

*Anlage 3 – Vergütung und Leistungsbeschreibung*

**Abschnitt I**
**Beschreibung der besonderen SAPV-Leistungen**

Die besonderen Leistungen der spezialisierten ambulanten Palliativversorgung werden in vier Versorgungsebenen erbracht:

**Ebene 0:**

Stellt sich im Rahmen des Eingangsassessments heraus, dass die leistungsbegründenden Faktoren der SAPV nicht erfüllt sind und keine SAPV notwendig ist, so kann für die Erhebung des Eingangsassessments, das Übermitteln der Ergebnisse und die ggf. notwendige Information weiterer Behandler eine einmalige Pauschale je Verordnung abgerechnet werden. Weitere SAPV-Leistungen können parallel nicht abgerechnet werden. Werden SAPV-Leistungen innerhalb 28 Tagen notwendig, erfolgt eine Verrechnung mit der Vergütung der Ebene 1.

**Ebene 1: Beratungsleistung**

a) Erhebung des Eingangsassessments gemäß Anlage 5 einschließlich Prüfung der Verordnungsintensität im persönlichen Gespräch mit Betroffenen (Kranken und/oder deren Angehörige)
b) Spezialisierte Beratung des Patienten, seines Umfeldes oder der betreuenden Leistungserbringer der Primärversorgung bezogen auf die physischen, psychischen, sozialen und spirituellen Bedürfnisse des Patienten und seines Umfeldes.
c) Dokumentation und Evaluation der Beratung gemäß § 11 im jeweils erforderlichen Umfang

**Ebene 2: Koordination der Versorgung**

a) beinhaltet die Beratungsleistung gemäß Ebene 1
b) Fortlaufende Koordination der spezialisierten palliativmedizinischen und palliativpflegerischen Versorgung unter Einbeziehung weiterer Berufsgruppen im Rahmen einer multiprofessionellen Zusammenarbeit. Dazu werden auch viermal jährlich Qualitätszirkel bzw. Netzwerkkonferenzen durchgeführt.
c) Erstellung, Abstimmung und ggf. Anpassung einer ressourcenfokussierten Hilfeplanung einschließlich einer Notfallplanung
d) Sicherstellung der Umsetzung der Hilfe- und Notfallplanung, Monitoring (Regel-Hausbesuche nach Ebene 3 und 4), Verlaufskontrolle
e) 24-Stunden-Rufbereitschaft für den betreffenden Patienten.
f) Dokumentation und Evaluation gemäß § 11 dieses Vertrags im jeweils erforderlichen Umfang

**Ebene 3: Additiv unterstützende Teilversorgung**

a) beinhaltet die Beratungs- und Koordinationsleistung gemäß Ebenen 1 und 2
b) Der Umfang der spezialisierten Leistungen richtet sich nach dem Bedarf des Betroffenen und kann einzelne Leistungen des SAPV-Spektrums, wie unter der Vollversorgung aufgeführt, umfassen.
c) Palliativmedizinische Maßnahmen, die nach ihrer Art, Schwere oder Komplexität eine Kompetenz erfordern, die der einer Ärztin oder eines Arztes mit Zusatzweiterbildung Palliativmedizin gemäß Anlagen 1 und 2 entspricht.
d) Spezialisierte palliativpflegerische Leistungen, die nach ihrer Art, Schwere oder Komplexität eine Kompetenz erfordern, die der einer Pflegefachkraft mit einer curricularen Weiterbildung zu Palliative Care gemäß Anlagen 1 und 2 entspricht.
e) Die spezialisierten Leistungen der SAPV werden als multiprofessionelle Komplexleistungen erbracht.

f) Dokumentation und Evaluation gemäß § 11 dieses Vertrags im jeweils erforderlichen Umfang

Additiv unterstützende Teilversorgung findet statt, wenn entweder der betreuende Hausarzt oder der betreuende Pflegedienst Teile der Behandlung, die spezielle palliativmedizinische oder palliativpflegerische Kompetenz erfordern, an das SAPV-Team abgegeben hat. Neben der im Rahmen der SAPV übernommenen Behandlungspflege ist dann diese Behandlungspflege gemäß § 37 SGB V durch einen anderen Pflegedienst nicht abrechenbar; Ansprüche nach SGB XI bleiben hiervon unberührt.

**Ebene 4: Vollständige Versorgung**

a) beinhaltet die Beratungs- und Koordinationsleistung gemäß Ebenen 1 und 2

b) umfasst sämtliche palliativmedizinischen und palliativpflegerischen Leistungen des in § 5 Abs. 3 der SAPV-Richtlinie des G-BA aufgeführten Leistungskatalogs für den betroffenen Patienten, soweit diese nach ihrer Art, Schwere oder Komplexität eine fachliche Qualifikation gemäß Anlagen 1 und 2 erfordern.

c) Dokumentation und Evaluation gemäß § 11 dieses Vertrags im jeweils erforderlichen Umfang

Vollständige Versorgung findet statt, wenn die Primärversorger (Hausarzt und Pflegedienst) die Behandlung, die spezielle palliativmedizinische oder palliativpflegerische Kompetenz erfordert, an das SAPV-Team abgegeben haben. Neben der im Rahmen der SAPV übernommenen Behandlungspflege ist dann diese Behandlungspflege gemäß § 37 SGB V durch einen anderen Pflegedienst nicht abrechenbar; Ansprüche nach SGB XI bleiben hiervon unberührt.

## Abschnitt II
## Vergütung der besonderen SAPV-Leistungen

(1) Die besonderen Leistungen der spezialisierten ambulanten Palliativversorgung in diesem Vertrag werden von den teilnehmenden spezialisierten Leistungserbringern außerhalb des Sicherstellungsauftrags der KV bzw. außerhalb der bestehenden Versorgungsverträge geleistet und von den Krankenkassen gesondert vergütet. Daneben ist die Abrechnung von Leistungen i.R.d. vertragsärztlichen Versorgung und die Abrechnung von Leistungen der Behandlungspflege gemäß § 37 SGB V ausgeschlossen; bei einer Doppelabrechnung (KV/Behandlungspflege und SAPV) entfällt der Vergütungsanspruch aus dieser SAPV-Versorgung. Überzahlungen werden mit Folgezahlungen verrechnet.

(2) Die Leistungen müssen ausreichend, zweckmäßig und wirtschaftlich sein; sie dürfen das Maß des Notwendigen nicht überschreiten. Leistungen, die nicht notwendig oder unwirtschaftlich sind, können Versicherte nicht beanspruchen, dürfen die Leistungserbringer nicht bewirken und die Krankenkassen nicht bewilligen.

(3) Inhalt dieser besonderen Leistungen der spezialisierten ambulanten Palliativversorgung ist der – über das Maß der Regelversorgung und allgemeinen Palliativversorgung hinausgehende – besondere Beratungs- / Betreuungs- / Koordinations- und Dokumentationsaufwand für die nach § 3 des Vertrags über die spezialisierte ambulante palliativmedizinische Versorgung von unheilbar erkrankten Patienten im häuslichen Umfeld anspruchsberechtigten Versicherten.

(4) Grundsätzlich gilt, dass SAPV nur dann und nur solange erbracht wird, wie eine Versorgung im Rahmen der Primärversorgung / allgemeine Palliativversorgung nicht ausreicht. Die SAPV ist ausschließlich vorgesehen als terminale Betreuung von Patienten, die keine kurative Therapie mehr erhalten. Im Falle der mittel- und langfristigen Besserung bzw. einer Stabilisierung im Krankheitsverlauf ist der Versorgungsumfang der SAPV so weit wie möglich zu reduzieren und eine Weiterversorgung im Rahmen der allgemeinen Palliativversorgung anzustreben.

(5) Die Vergütung für die besonderen Leistungen der spezialisierten ambulanten Palliativversorgung durch den spezialisierten Leistungserbringer beträgt*[1]

| | | bei Aufenthalt des Patienten im häuslichen Umfeld | bei Aufenthalt des Patienten in einer stationären Pflegeeinrichtung | bei Aufenthalt des Patienten in einem stationären Hospiz |
|---|---|---|---|---|
| für die Leistungen der **Ebene 0** | einmalig je Patient;<br>werden SAPV-Leistungen zu einem späteren Zeitpunkt notwendig, erfolgt eine Verrechnung mit den Vergütungssätzen der Ebene 1 | 50,00 € | 50,00 € | 25,00 € |
| für die Leistungen der **Ebene 1** (Beratung) | einmalig je Patient unabhängig von der Zahl der Verordnungstage (Eingangsassessment); bei Kindern (Versicherte bis zum vollendeten 18. Lebensjahr) kann die Beratung / Koordination erneut zur Abrechnung gelangen, wenn zwischen dem Tag der letzten Leistungserbringung und einer erneuten Verordnung mindestens 28 Tage liegen.<br>Für den ärztlichen und/oder pflegerischen Hausbesuch zur Erhebung des Eingangsassessments, sind neben der Vergütung der Ebene 1 die gesondert gekennzeichneten Vergütungssätze der Ebenen 3 und 4 abrechenbar. | 143,50 € | 143,50 € | entfällt |
| zusätzlich zu Ebene 1 für die Leistungen der **Ebene 2** (Koordination der Versorgung) *[2] | je Tag an dem mindestens eine der in der Leistungsbeschreibung gemäß Ebene 2 aufgeführten Koordinationsleistungen durchgeführt und dokumentiert wurden, inkl. Morgenbesprechungen, Fallkonferenzen / Telefonate etc.;<br>die Leistungen gemäß Ebene 2 e) und f) sind in dieser Vergütung enthalten zusätzlich Vergütungssätze der Ebenen 3 und 4 für den wöchentlichen Monitoringbesuch und Aktiveinsätze in der Rufbereitschaft abrechenbar | 54,60 € | 54,60 € | entfällt |
| zusätzlich zu Ebene 1 und 2 für die Leistungen der **Ebene 3** (additiv unterstützende Teilversorgung) und **Ebene 4** (vollständige Versorgung) inklusive Fahrzeiten, Fahrtkosten | für jeden ärztlichen Hausbesuch (auch Hausbesuch zum Erstassessment der Ebene 1 oder Monitoringbesuche der Ebene 2 oder Aktiveinsätze im Rufdienst) mind. 20'-45' Besuchszeit beim Patienten | 81,00 € | 81,00 € | 81,00 € |
| | für jeden pflegerischen Hausbesuch(auch Hausbesuch zum Erstassessment der Ebene 1 oder Monitoringbesuche der Ebene 2 oder Aktiveinsätze im Rufdienst) mind. 20'-45' Besuchszeit beim Patienten | 55,00 € | 55,00 € | entfällt |
| | Zusatzentgelt für weitere 15' Hausbesuchszeit für ärztliche Hausbesuche, auch im Rahmen der Rufbereitschaft; abrechenbar ab Vollendung der 1.' | 15,00 € | 15,00 € | 15,00 € |

| | | | | |
|---|---|---|---|---|
| | Zusatzentgelt für weitere 15’ Hausbesuchszeit für pflegerische Hausbesuche, auch im Rahmen der Rufbereitschaft; abrechenbar ab Vollendung der 1.’ | 10,00 € | 10,00 € | entfällt |
| | Abschlag für ärztliche Hausbesuche, auch im Rahmen der Rufbereitschaft bei Unterschreitung der Mindestbesuchszeit von 20’ beim Patienten | - 15,00 € | - 15,00 € | - 15,00 € |
| | Abschlag für pflegerische Hausbesuche, auch im Rahmen der Rufbereitschaft bei Unterschreitung der Mindestbesuchszeit von 20’ beim Patienten | - 10,00 € | 10,00 € | entfällt |

*[1] in allen Leistungen wurden Sach- und Gemeinkosten berücksichtigt

*[2] Die Pauschale ist abhängig von der betreuten Patientenzahl und deren Verweildauer. Der aufgeführte Wert bezieht sich auf 150 Gesamtpatienten (30 Beratung, 120 Koordination / Teilversorgung / Vollversorgung) pro Jahr und eine durchschnittliche Verweildauer von 28 Tagen. Mit der Pauschale der Ebene 2 berücksichtigen wir die regionalen Gegebenheiten. Dies bedeutet, dass diese je nach Fallzahl/Verweildauer steigt oder sinkt. Damit ein SAPV-Team arbeitsfähig ist und die Leistung wirtschaftlich erbracht werden kann, muss ein SAPV-Team mindestens eine Versorgungsregion mit 150-200 SAPV-Patienten betreuen. Sollten die Krankenkassen zu einem späteren Zeitpunkt verpflichtet werden, in einer Versorgungsregion mehrere Leistungsanbieter zuzulassen, so richtet sich die Vergütung dieses Leistungskomplexes, für alle Leistungserbringer der Versorgungsregion, nach der Fallzahl/Verweildauer in der gesamten Versorgungsregion und nicht nach der Fallzahl/Verweildauer des einzelnen Leistungserbringers.

(6) Mit den Vergütungssätzen nach Absatz 5 ist die gesamte spezialisierte ambulante Palliativversorgung einschließlich Fahrtkosten / Wegegeldern in der vereinbarten Versorgungsregion abgegolten.
Erfolgt im Verordnungszeitraum eine Verlegung ins Krankenhaus, kann für den Aufnahmetag ins Krankenhaus keine Tagespauschale abgerechnet werden. Eine erneute Abrechnung der Tagespauschale ist wieder möglich ab dem Entlassungstag aus dem Krankenhaus. Ausnahme: weist der SAPV-Leistungserbringer am Aufnahmetag ins Krankenhaus einen Einsatz nach, kann für diesen Tag auch eine Tagespauschale abgerechnet werden.
Wird ein Versicherter in ein Krankenhaus, eine stationäre Pflegeeinrichtung oder ein stationäres Hospiz verlegt, so ist dies unmittelbar der jeweiligen Krankenkasse mitzuteilen. Soweit für die Vergütungen nach diesem Vertrag Umsatzsteuerpflicht besteht, ist diese mit dem vereinbarten Betrag abgegolten.

(7) Die Abrechnung der besonderen Leistungen der spezialisierten ambulanten Palliativversorgung erfolgt durch den spezialisierten Leistungserbringer nach Maßgabe der §§ 12 bis 14 des Vertrags über die spezialisierte ambulante palliativmedizinische Versorgung von unheilbar erkrankten Patienten im häuslichen Umfeld.

(8) Der SAPV-Leistungserbringer bestätigt mit jedem Abrechnungsfall gegenüber der zuständigen Krankenkasse, dass die erbrachten und abgerechneten SAPV-Leistungen ausschließlich durch sozialversicherungspflichtig beschäftigte Palliativpflegekräfte/sonstige Fachkräfte des jeweiligen SAPV-Leistungserbringers erbracht wurden. Bestandteil jeder Abrechnung ist die Datenlieferung gemäß Anhang 1 dieser Anlage 3. Der SAPV-Leistungserbringer liefert gegenüber allen vertragschließenden Krankenkassen jeweils zum 15.01., 15.04., 15.07. und 15.10. eine kassenübergreifende Leistungsdatenstatistik des Vorquartals gemäß Anhang 1 dieser Anlage 3 in pseudonymisierter Form.

(9) Abweichend von § 18 des Vertrages gem. § 132d SGB V über die spezialisierte ambulante palliativmedizinische Versorgung von unheilbar erkrankten Patienten im häuslichen Umfeld ist die Laufzeit dieser Vergütungsvereinbarung (Anlage 3) befristet auf ein Jahr, beginnend mit dem Vertragsbeginn. Die Vereinbarung dieser Vergütungssätze erfolgt ohne Präjudizwirkung für Folgevereinbarungen, insbesondere ohne Anerkenntnis der dieser Vergütung zu Grunde liegenden Kalkulation.

*Anhang 1 zur Anlage 3*

# Datensatzbeschreibungen zur Vergütungsvereinbarung des Vertrags nach § 132 d SGB V über die spezialisierte ambulante palliativmedizinische Versorgung von unheilbar erkrankten Patienten im häuslichen Umfeld

## Erläuterungen

Die Abrechnungsunterlagen sind vom SAPV-Leistungserbringer im Wege elektronischer Datenübertragung oder maschinell verwertbar auf Datenträgern (Disketten oder CD) an die Krankenkassen zu übermitteln. Vor der Übermittlung müssen die Datensätze verschlüsselt werden. Die Vertragspartner einigen sich auf eine mit den Datenschützern abgestimmte Form der Verschlüsselung (z. B. PGP).

Die Daten sind als Textdatei mit **;** als Feldbegrenzungszeichen ohne Textbegrenzungszeichen zu liefern. Zur Trennung der Datensätze wird der Zeilenumbruch verwendet. Ein Kopfdatensatz mit der Feldbezeichnung wird nicht mitgeliefert. Datumsfelder sind generell im Format „DD.MM.YYYY“ zu liefern. Die Diagnosen im Datensatz 1 und Datensatz 2 sind nach der Internationalen Klassifikation der Krankheiten in der jeweiligen vom Deutschen Institut für medizinische Dokumentation und Information im Auftrag des BMG herausgegebenen deutschen Fassung zu verschlüsseln.

## Datensatz 1 – SAPV-Abrechnung

| Nr | Feldinhalt | Datentyp | Feldgröße | Format | Pflichtfeld | Erläuterungen |
|---|---|---|---|---|---|---|
| 1 | BSNR des SAPV-Leistungserbringers | NUM | 9 | | ja | IK-Nummer des SAPV-Stützpunktes |
| 2 | SAPV-Rechnungsnummer | TEXT | 19 | #-000 | ja | Die SAPV-Rechnungsnummer setzt sich aus der SAPV-Fall-Nummer, Bindestrich und einer fortlaufenden 3stelligen Nummer zusammen:<br>BSP: AOK0001-001<br>Die SAPV ist der Primärschlüssel beim Leistungserbringer und bildet zusammen mit der BSNR des SAPV-Leistungserbringers den Primärschlüssel bei den Krankenkassen. |
| 3 | SAPV-Fall-Nummer | TEXT | 15 | | ja | BSP: AOK0001 |
| 4 | Kassennummer | NUM | 7 | | optional | Kassennummer wie auf der Verordnung / KV-Karte des Versicherten abgebildet<br>Kann fehlen, wenn nicht bekannt |
| 5 | Kasse | TEXT | 50 | | ja, wenn Kassennummer fehlt | AOK, BARMER-GEK, DAK, TK, KKH-Allianz, HEK, hkk, IKK Südwest, Privat,.... |
| 6 | Versichertennummer | TEXT | 15 | | optional | wie auf der Verordnung / KV-Karte des Versicherten abgebildet<br>Kann fehlen, wenn nicht bekannt |
| 7 | Name | TEXT | 50 | | ja, wenn Versichertennummer fehlt | |

| Nr | Feldinhalt | Datentyp | Feldgröße | Format | Pflichtfeld | Erläuterungen |
|---|---|---|---|---|---|---|
| 8 | Vorname | TEXT | 25 | | ja, wenn Versicherten-nummer fehlt | |
| 9 | Geb.Datum | DATUM | | DD.MM.YYYY | ja | |
| 10 | Geschlecht | NUM | 1 | | ja | 0 = männlich<br>1 = weiblich |
| 11 | Pflegestufe bei Abrechnung der Verordnung/Beendigung der SAPV | NUM | 1 | | Ja, wenn bekannt | 0= keine Pflegestufe<br>1= Pflegestufe 1<br>2= Pflegestufe 2<br>3= Pflegestufe 3<br>4= Pflegestufe 3+<br>5= unbekannt<br><br>wird bei Abschluss des Falles erhoben, sonst Angabe evtl. nicht korrekt, da während der Betreuung eine Pflegestufe bzw. Höhergruppierung der Pflegestufe beantragt wurde |
| 12 | Abrechnungszeitraum Beginn | DATUM | | DD.MM.YYYY | ja | |
| 13 | Abrechnungszeitraum Ende | DATUM | | DD.MM.YYYY | ja | |
| 14 | BSNR Verordner | NUM | 9 | | ja | |
| 15 | LANR Verordner | NUM | 9 | | ja | |
| 16 | Art der Verordnung | NUM | 1 | | ja | 1= Erstverordnung<br>2= Folgeverordnung |
| 17 | Verordnung von | NUM | 1 | | ja | 1= Hausarzt<br>2= Facharzt<br>3= Klinikarzt<br>0= unbekannt |
| 18 | Verordnungsdatum | DATUM | | DD.MM.YYYY | ja | |
| 19 | Verordnungszeitraum von | DATUM | | DD.MM.YYYY | ja | wenn kein Verordnungsdatum bekannt ist, ist das Feld mit dem Wert 31.12.9999 zu füllen |
| 20 | Verordnungszeitraum bis | DATUM | | DD.MM.YYYY | ja | wenn kein Verordnungsdatum bekannt ist, ist das Feld mit dem Wert 31.12.9999 zu füllen |
| 21 | Anzahl tatsächliche Betreuungstage (= Abrechnungstage) des SAPV Teams im Verordnungszeitraum | NUM | 3 | | ja | Anzahl Abrechnungstage<br>0= Standardwert |
| 22 | ICD 10-Hauptdiagnose | TEXT | 10 | | ja | ICD 10, Bsp. I63.3 |

| Nr | Feldinhalt | Datentyp | Feldgröße | Format | Pflichtfeld | Erläuterungen |
|---|---|---|---|---|---|---|
| **Welches komplexe Symptomgeschehen wurde auf der Verordnung angegeben** | | | | | | |
| 23 | ausgeprägte Schmerzsymptomatik | NUM | 1 | | ja | 1= war angekreuzt<br>0= war nicht angekreuzt |
| 24 | ausgeprägte gastrointestinale Symptomatik | NUM | 1 | | ja | 1= war angekreuzt<br>0 = war nicht angekreuzt |
| 25 | ausgeprägte neurologische/ psychiatrische/ psychische Symptomatik | NUM | 1 | | ja | 1= war angekreuzt<br>0= war nicht angekreuzt |
| 26 | ausgeprägte respiratorische/kardiale Symptomatik | NUM | 1 | | ja | 1= war angekreuzt<br>0= war nicht angekreuzt |
| 27 | ausgeprägte urogenitale Symptomatik | NUM | 1 | | ja | 1= war angekreuzt<br>0= war nicht angekreuzt |
| 28 | ausgeprägte ulzerierende/exulzeriere nde Wunden und Tumore | NUM | 1 | | ja | 1= war angekreuzt0= war nicht angekreuzt |
| 29 | Sonstiges | NUM | 1 | | ja | 1= war angekreuzt<br>0= war nicht angekreuzt |
| **Verordnete Leistung** | | | | | | |
| 30 | Beratung | NUM | 1 | | ja | 1= war angekreuzt<br>0= war nicht angekreuzt |
| 31 | Koordination | NUM | 1 | | ja | 1= war angekreuzt<br>0= war nicht angekreuzt |
| 32 | Teilversorgung | NUM | 1 | | ja | 1= war angekreuzt<br>0= war nicht angekreuzt |
| 33 | Vollversorgung | NUM | 1 | | ja | 1= war angekreuzt<br>0= war nicht angekreuzt |
| 34 | keine Angabe | NUM | 1 | | ja | 1= keine Leistung angekreuzt auf der Verordnung<br>0= war nicht angekreuzt |
| **Tatsächlicher SAPV Bedarf** | | | | | | |
| 35 | kein SAPV-Bedarf | NUM | 1 | | ja | 1= kein SAPV-Bedarf<br>0= Standardwert |
| 36 | Beratung | NUM | 3 | | ja | Anzahl Tage, Bsp. "3", wenn 3 Tage Beratung<br>0= Standardwert |
| 37 | Koordination | NUM | 3 | | ja | Anzahl Tage, Bsp. "3", wenn 3 Tage Beratung und Koordination<br>0= Standardwert |
| 38 | Teil-/Vollversorgung | NUM | 3 | | ja | Anzahl Tage, Bsp. "3", wenn 3 Tage Beratung Koordination, Teil-/Vollversorgung.<br>0= Standardwert |

| Nr | Feldinhalt | Datentyp | Feldgröße | Format | Pflichtfeld | Erläuterungen |
|---|---|---|---|---|---|---|
| 39 | Anzahl der beteiligten Ehrenamtlichen | NUM | 3 | | optional | Soweit nicht im Datensatz SAPV-Leistungserbringer vorhanden |
| 40 | Anzahl der beteiligten Angehörigen | NUM | 3 | | optional | Soweit nicht im Datensatz SAPV-Leistungserbringer vorhanden |
| 41 | Anzahl der sonstigen beteiligten Leistungserbringer | NUM | 3 | | optional | soweit nicht im Datensatz SAPV-Leistungserbringer vorhanden |
| 42 | Therapieende | NUM | 1 | | ja | 1= verstorben<br>2= Verlegung und damit Beendigung der SAPV<br>3= Entlassung<br>4= Wechsel SAPV-Leistungserbringer<br>5= Sonstiges<br>6= Patient noch in laufender Behandlung<br>7= Patient ruhend<br>Wenn zum Zeitpunkt der Datenabfrage noch kein Therapieende - dann Angabe 6 oder 7 |
| 43 | Sterbeort des Versicherten | NUM | 1 | | ja, wenn Patient verstirbt | 1= zu Hause<br>2= Alten-/Pflegeheim<br>3= stationäres Hospiz<br>4= Palliativstation<br>5= Krankenhaus<br>6= sonstige Heime/Einrichtungen<br>7= unbekannt<br>8= Sonstige<br><br>wenn der Versicherte im Verordnungszeitraum der SAPV verstirbt, muss hier eine Angabe erfolgen, es muss auch eine Angabe erfolgen, wenn der Patient in der Klinik/Pallitivstation verstirbt, obwohl durch diese Aufenthalte die SAPV unterbrochen wird |

## Beispiel „SAPV_ABRECHNUNG.csv“:

745112363;A2010_001-001;A2010_001;7310373;;123456789;;;03.01.1923;0;2;01.11.2010;30.11.2010;4819
11300;773223403;1;1;02.11.2010;03.11.2010;30.11.2010;10;C83.2;1;0;1;0;0;1;0;1;1;0;1;0;0;1;2;2;0;1;0;1;1

## Datensatz 2 – SAPV-Diagnosen

| Nr | Feldinhalt | Datentyp | Feldgröße | Format | Pflichtfeld | Erläuterungen |
|---|---|---|---|---|---|---|
| 1 | BSNR der SAPV | NUM | 9 | | ja | IK-Nummer des SAPV-Stützpunktes |
| 2 | SAPV-Rechnungsnummer | TEXT | 24 | | ja | |
| 3 | ICD 10 | TEXT | 10 | | ja | Bsp. I63.3 |
| 4 | BSNR Verordner | NUM | 9 | | ja | |
| 5 | LANR Verordner | NUM | 9 | | ja | |
| 6 | Datum der Verordnung | DATUM | | DD.MM.YYYY | ja | |

**Beispiel „SAPV_DIAGNOSEN.csv“:**
745112363;A2010_001-001;C83.2;481911300;773223403;02.11.2010
745112363;A2010_001-001;E14.7;481911300;773223403;02.11.2010
745112363;A2010_001-001;J44.09;481911300;773223403;02.11.2010

## Datensatz 3 – weitere Leistungserbringer, die außerhalb des SAPV-Teams eingesetzt werden bei der Versorgung des Patienten

| Nr | Feldinhalt | Datentyp | Feldgröße | Format | Pflichtfeld | Erläuterungen |
|---|---|---|---|---|---|---|
| 1 | BSNR der SAPV | NUM | 9 | | ja | IK-Nummer des SAPV-Stützpunktes |
| 2 | SAPV-Rechnungsnummer | TEXT | 24 | | ja | |
| 3 | Art des Leistungserbringers | NUM | 1 | | ja | 0= sonstige Leistungserbringer<br>1= Hausarzt<br>2= Facharzt<br>3= Klinikarzt<br>4= Psychotherapeut<br>5= Pflegedienst<br>6= Physiotherapeut<br>7= amb. Hospizdienst<br>8= Alten-/Pflegeheim<br>9= Krankenhaus |
| 4 | BSNR / IKNR | NUM | 9 | | Optional | |
| 5 | LANR | NUM | 9 | | ja, wenn ärztliches Personal | bedingtes Pflichtfeld: handelt es sich bei dem Leistungserbringer um ärztliches Personal ist die Lebenslange Arztnummer des beteiligten Arztes anzugeben |
| 6 | Name des Leistungserbringes | TEXT | 100 | | ja, wenn BSNR / IKNR fehlt | bedingtes Pflichtfeld: Der Name des Leistungserbringers ist anzugeben, wenn die Kassennummer nicht bekannt ist |
| 7 | Vorname des Leistungserbringes | TEXT | 50 | | ja, wenn BSNR / IKNR fehlt | bedingtes Pflichtfeld: Der Vorname des Leistungserbringers ist anzugeben, wenn die Kassennummer nicht bekannt ist und es sich bei dem Leistungserbringer um eine natürliche Person handelt |
| 8 | Strasse /HNR des Leistungserbringes | TEXT | 25 | | ja, wenn BSNR / IKNR fehlt | bedingtes Pflichtfeld: Die Strasse des Leistungs-erbringers ist anzugeben, wenn die Kassennummer nicht bekannt ist |

| Nr | Feldinhalt | Datentyp | Feldgröße | Format | Pflichtfeld | Erläuterungen |
|---|---|---|---|---|---|---|
| 9 | PLZ des Leistungserbringes | TEXT | 5 | | ja, wenn BSNR / IKNR fehlt | bedingtes Pflichtfeld: Die PLZ des Leistungserbringers ist anzugeben, wenn die Kassennummer nicht bekannt ist |
| 10 | Ort des Leistungserbringes | TEXT | 25 | | ja, wenn BSNR / IKNR fehlt | bedingtes Pflichtfeld: Der Ort des Leistungserbringers ist anzugeben, wenn die Kassennummer nicht bekannt ist |
| 11 | Datum der Leistungserbringung Beginn / ab | DATUM | | DD.MM.YYYY | ja | |
| 12 | Datum der Leistungserbringung Ende / bis | DATUM | | DD.MM.YYYY | optional | |

## Beispiel „SAPV_LE.csv“:

745112363;A2010_001-001;1;481911300;773223403;;;;;;03.11.2010
745112363;A2010_001-001;5;;;Wichtig;Willi;Musterstr. 1;12345;Musterstadt;03.11.2010

## Datensatz 4 – SAPV-Hausbesuche/Fahrten

| Nr | Feldinhalt | Datentyp | Feldgröße | Format | Pflichtfeld | Erläuterungen |
|---|---|---|---|---|---|---|
| 1 | BSNR der SAPV | NUM | 9 | | ja | IK-Nummer des SAPV-Stützpunktes |
| 2 | SAPV-Rechnungsnummer | TEXT | 20 | | ja | |
| 3 | Fahrzeit (Min.) einfach vom Standort des SAPVT zum Patienten | NUM | 3 | | ja | |
| 4 | Art der Fahrten | | 1 | | ja | 1= Pflege<br>2= Arzt<br>3= sonstige SAPV-Fachkräfte |
| 5 | Datum der Fahrt | DATUM | | DD.MM.YYYY | ja | |
| 6 | Gesamtbetreuungszeit vor Ort beim Patienten in Minuten (ohne Fahrzeit) | NUM | 6 | | ja | |

## Beispiel „SAPV_FAHRTEN.csv“:

745112363;A2010_001-001;15;3;2;240
745112363;A2010_001-001;15;2;1;60
745112363;A2010_001-001;15;1;3;90

## Datensatz 5 – SAPV-Stationär

| Nr | Feldinhalt | Datentyp | Feldgröße | Format | Pflichtfeld | Erläuterungen |
|---|---|---|---|---|---|---|
| 1 | BSNR der SAPV | NUM | 9 | | ja | IK-Nummer des SAPV-Stützpunktes |
| 2 | SAPV-Rechnungsnummer | TEXT | 20 | | ja | |
| 3 | Art der Einrichtung | | 1 | | ja | 1= Krankenhaus<br>2= Reha-Klinik<br>3= Alten-/Pflegeheim<br>4= sonstige Heime<br>5= stationäre Hoszip<br>6= sonstige |
| 4 | IKNR | NUM | 9 | | optional | IKNR ist anzugeben; dass Feld ist optional wenn die IKNR des beteiligten Leistungserbringers nicht bekannt ist |
| 5 | Name des Leistungserbringes | TEXT | 100 | | ja, wenn IKNR fehlt | bedingtes Pflichtfeld: Der Name des Leistungserbringers ist anzugeben, wenn die IKNR nicht bekannt ist |
| 6 | Strasse /HNR des Leistungserbringes | TEXT | 25 | | ja, wenn IKNR fehlt | bedingtes Pflichtfeld: Der Name des Leistungserbringers ist anzugeben, wenn die IKNR nicht bekannt ist |
| 7 | PLZ des Leistungserbringes | TEXT | 5 | | ja, wenn IKNR fehlt | bedingtes Pflichtfeld: Der Name des Leistungserbringers ist anzugeben, wenn die IKNR nicht bekannt ist |
| 8 | Ort des Leistungserbringes | TEXT | 25 | | ja, wenn IKNR fehlt | bedingtes Pflichtfeld: Der Name des Leistungserbringers ist anzugeben, wenn die IKNR nicht bekannt ist |
| 9 | Art der Einweisung | NUM | 1 | | ja | 1= Einweisung<br>2= Verlegung |
| 10 | Behandlungszeitraum von | DATUM | | DD.MM.YYYY | Ja | |
| 11 | Behandlungszeitraum bis | DATUM | | DD.MM.YYYY | ja | Pflichtfeld, ist der stationäre Aufenthalt noch laufend, ist der Wert „31.12.9999“ zu übermitteln. |

### Beispiel „SAPV_STATIONAER.csv“:

745112363;A2010_001-001;1;260730207;;;;;1;02.11.2010;02.11.2010

## Datensatz 6 – SAPV-Teambesprechungen

| Nr | Feldinhalt | Datentyp | Feldgröße | Format | Pflichtfeld | Erläuterungen |
|---|---|---|---|---|---|---|
| 1 | BSNR der SAPV | NUM | 9 | | ja | |
| 2 | SAPV-Rechnungsnummer | TEXT | 20 | | ja | |
| 3 | Datum der Teambesprechung | DATUM | | DD.MM.YYYY | ja | |
| 4 | Durchschnittl. Dauer in Minuten für den Patient | NUM | 3 | | ja | |
| 5 | Anzahl der Mitarbeiter | NUM | 3 | | ja | |

### Beispiel „SAPV_TEAM.csv“:

745112363;A2010_001-001;02.11.2010;30;3
745112363;A2010_001-001;03.11.2010;20;3

Krankenkasse bzw. Kostenträger

Name. Vorname des Versicherten

geb. am

Kassen-Nr. | Versicherten-Nr. | Status

Betriebsstätten-Nr

# SAPV- Abrechnungsbogen

**Abrechnungs-IK:**

Stempel

**Rechnungs-Nr.:** SAPV-Fall-Nr -

☐ **Erstverordnung** ☐ **Folgeverordnung** **vom** **bis**

**Abrechnungszeitraum:** **bis**

| Gebührenposition | Häusliches Umfeld | Stat. Pflege-einrichtung | Stat. Hospiz | Anzahl | Summe |
|---|---|---|---|---|---|
| **Ebene 0** (keine SAPV) | ☐ 50,00 EUR | ☐ 50,00 EUR | ☐ 50,00 EUR | 1 | |
| **Ebene 1** (Beratung) | ☐ 143,50 EUR | ☐ 143,50 EUR | → | | |
| **Ebene 2** (Koordination der Versorgung) | ☐ 54,60 EUR | → | → | | |
| | | ☐ 54,60 EUR | → | | |
| **Ärztlicher Hausbesuch** | ☐ 81,00 EUR | → | → | | |
| | | ☐ 81,00 EUR | → | | |
| | | | ☐ 81,00 EUR | | |
| **Pflegerischer Hausbesuch** | ☐ 55,00 EUR | → | → | | |
| | | ☐ 55,00 EUR | → | | |
| **Zusatzentgelt** für weitere 15' Hausbesuchszeit für **ärztl.** Hausbesuche; abrechenbar ab Vollendung der 1.' | ☐ 15,00 EUR | → | → | | |
| | | ☐ 15,00 EUR | → | | |
| | | | ☐ 15,00 EUR | | |
| **Zusatzentgelt** für weitere 15' Hausbesuchszeit für **pfleg.** Hausbesuche; abrechenbar ab Vollendung der 1.' | ☐ 10,00 EUR | → | → | | |
| | | ☐ 10,00 EUR | → | | |
| **Abschlag** für **ärztl.** Hausbesuche bei Unterschreitung der Mindestbesuchszeit von 20' | ☐ -15,00 EUR | → | → | | – |
| | | ☐ -15,00 EUR | → | | – |
| | | | ☐ -15,00 EUR | | – |
| **Abschlag** für **pfleg.** Hausbesuche bei Unterschreitung der Mindestbesuchszeit von 20' | ☐ -10,00 EUR | → | → | | – |
| | | ☐ -10,00 EUR | → | | – |
| | | | | **Gesamtbetrag:** | |

Die vollständige Datenlieferung zur Abrechnung wurde am übermittelt.
Wir bestätigen, dass die erbrachten und abgerechneten SAPV-Leistungen ausschließlich durch sozialversicherungspflichtig beschäftigte Palliativpflegekräfte/sonstige Fachkräfte des SAPV-Leistungserbringers erbracht wurden.

Stand: 14.01.2011

Datum, Unterschrift

*Anhang 3 zur Anlage 3*

## SAPV-Abrechnungstool „SAPV-Patientenakte.xlt“

Bild 1: Erfassung der Patientendaten:

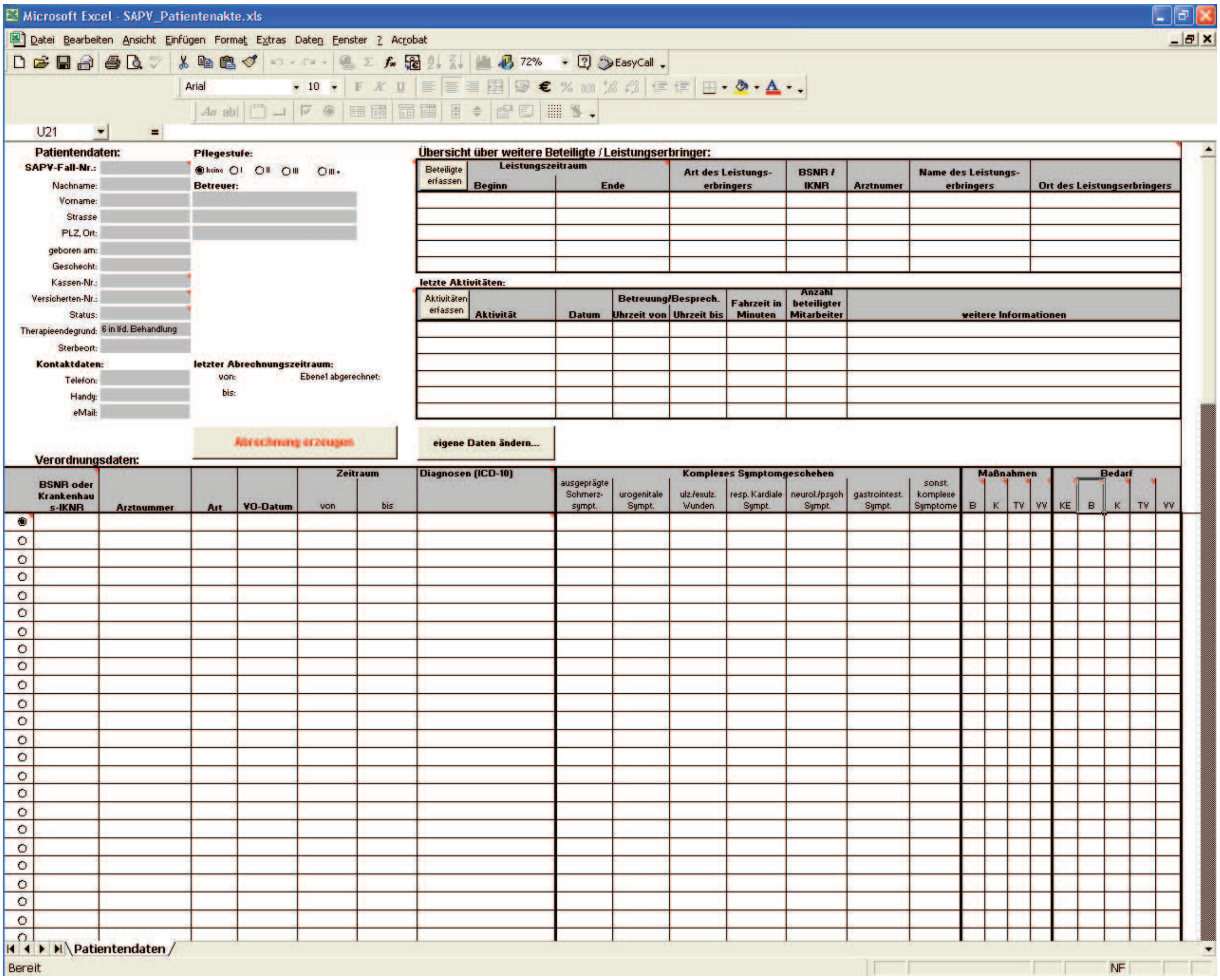

Bild 2 Erfassung Leistungserbringer:

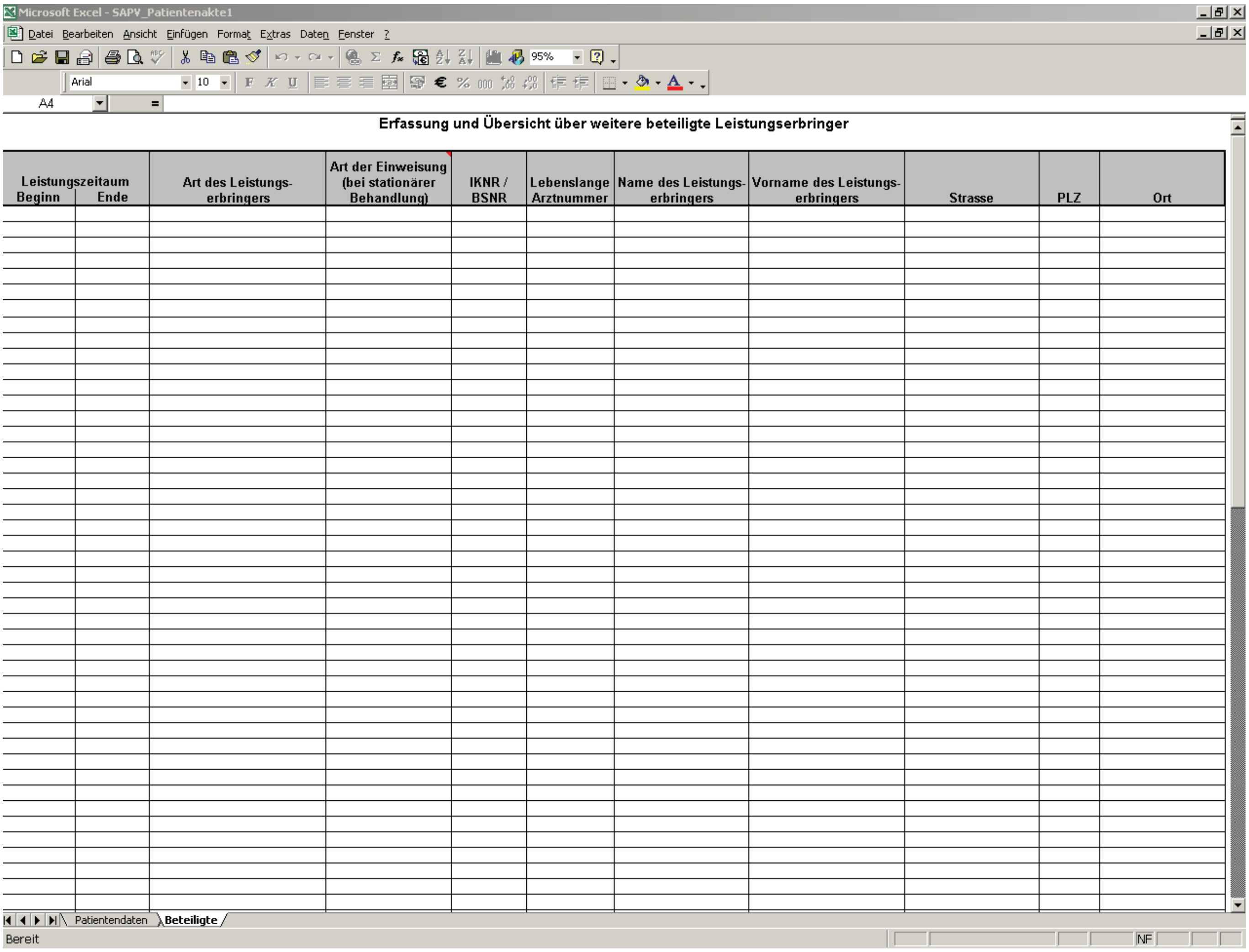


**Erfassung und Übersicht über weitere beteiligte Leistungserbringer**

| Leistungszeitraum Beginn | Ende | Art des Leistungs-erbringers | Art der Einweisung (bei stationärer Behandlung) | IKNR / BSNR | Lebenslange Arztnummer | Name des Leistungs-erbringers | Vorname des Leistungs-erbringers | Strasse | PLZ | Ort |
|---|---|---|---|---|---|---|---|---|---|---|
| | | | | | | | | | | |

Bild 3 Erfassung durchgeführter Aktivitäten:

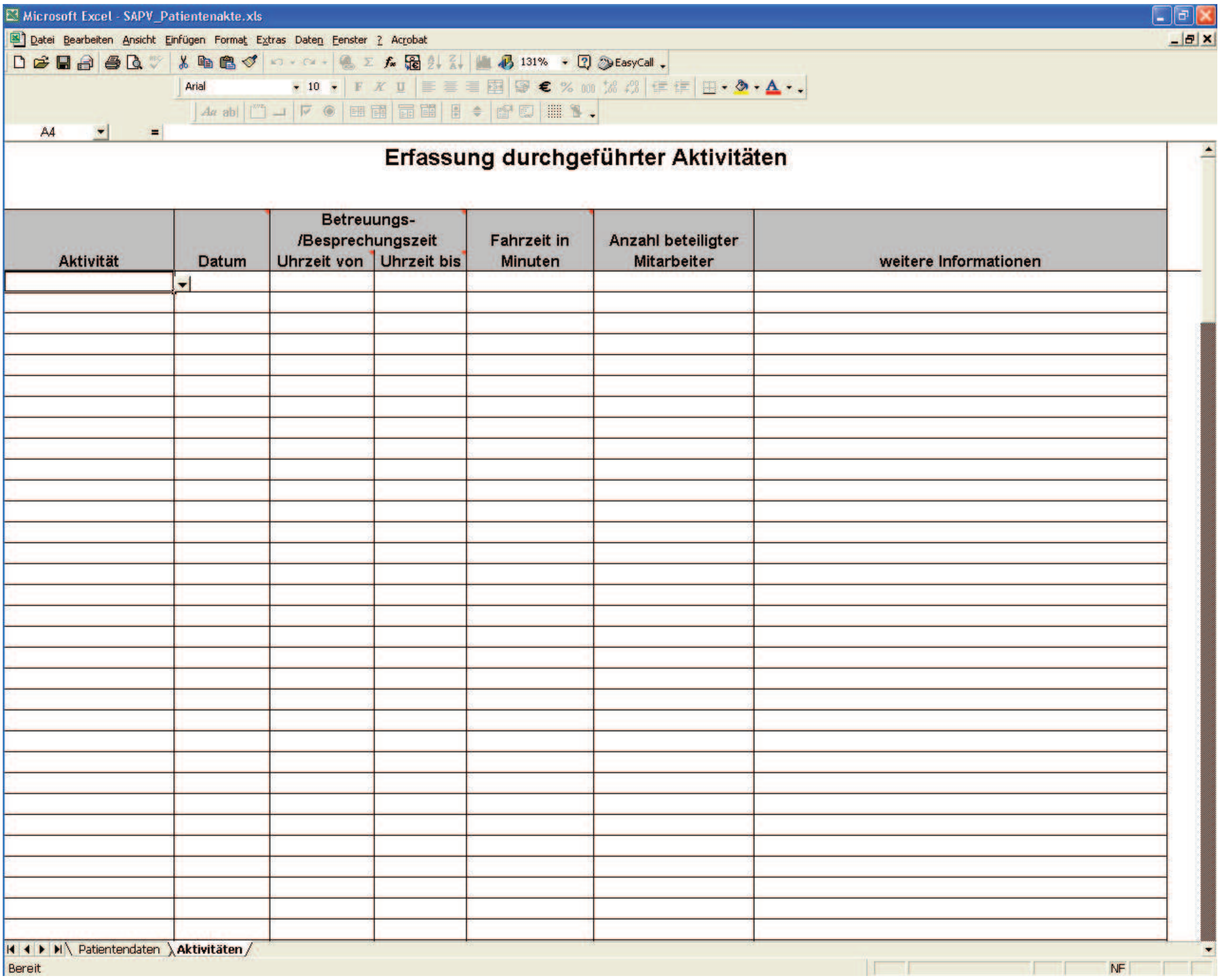

# Dokumentation der Excel-Anwendung „SAPV-Patientenakte.xlt“

## Inhalt:

1 Informationen zur Anwendung .......... 46
2 Starten der Anwendung .......... 46
3 Tabellenblatt Patientendaten .......... 46
3.1 Verordnungsdaten .......... 46
3.1.1 Muster 63: .......... 48
3.2 Erfassung und Übersicht weiterer Beteiligter .......... 49
3.3 Aktivitäten erfassen und anzeigen .......... 49
3.4 Schaltfläche „eigene Daten ändern...“ .......... 50
3.4.1 Schaltfläche „Abrechnung erzeugen“ .......... 50
4 Abrechnung erzeugen .......... 50
4.1 Tabellenblatt Leistungsnachweis .......... 50
4.1.1 Mögliche Korrekturen im Leistungsnachweis .......... 50
4.1.2 Schaltfläche „Abrechnungsbogen anzeigen/ausblenden“ .......... 51
4.1.3 Schalftfäche „Leistungsnachweis und Abrechnungsbogen drucken und Datensätze erzeugen“ .......... 51

## Informationen zur Anwendung

Das Tool „SAPV-Patientenakte.xlt“ soll SAPV-Leistungserbringer ohne eigene Softwarelösung bei der Dokumentation und Abrechnung der SAPV-Leistung unterstützen. Bei der Anwendung handelt es sich um eine Excel-Vorlage. Für jeden Patienten muss auf Grundlage der Excel-Vorlage „SAPV-Patientenakte.xlt“ eine eigen Excel-Datei erstellt und gespeichert werden. Der ordnungsgemäße datenschutzrechtliche Umgang, die ordnungsgemäße Sicherung der Daten und die ordnungsgemäße Abrechnung der Leistung obliegt dem Leistungserbringer.

## Starten der Anwendung

Speichern Sie zunächst die „SAPV-Patientenakte.XLT“ im Vorlagenverzeichnis. Standardmäßig befinden sich die Benutzervorlagendateien am folgenden Speicherort:

C:\Dokumente und Einstellungen*Benutzername*\Anwendungsdaten\Microsoft\Vorlagen

Die Benutzervorlage lässt sich nun über das Menü Datei/Neu... (STRG+N) auswählen. Beim ersten Starten der Benutzervorlage wird man in einem Pop-Up-Formular aufgefordert, die Eigenen Daten zu erfassen. Die Eingabe ist nur beim ersten mal erforderlich. Die Eigenen Daten werden beim Schließen des Pop-Up-Fensters in der Datei „C:\sapv.xml“ abgelegt und bei jedem öffnen der Datei neu ausgelesen. Jetzt wird das Excelblatt zur Erfassung der Patientendaten angezeigt.

<u>Hinweis:</u>
Änderungen an den Eigenen Daten haben Auswirkungen auf alle Dateien, die auf der Grundlage der Vorlage „SAPV-Patientenakte.XLT“ erstellt wurden.

## Tabellenblatt Patientendaten

Im Excelblatt Patientendaten werden die Stammdaten des Patienten erfasst. Zunächst ist eine SAPV-Fall-Nr. zu vergeben. Dieses Identifikationskennzeichen muss für jeden Patienten neu vergeben werden und darf aus Zahlen, Textzeichen und Sonderzeichen bestehen. Die SAPV-Fall-Nr. darf 15 Zeichen nicht überschreiten. Die nachfolgenden Daten können der SAPV-Verordnung entnommen werden: Nachname, Vorname, Strasse PLZ, Ort, geboren am. Im Eingabefeld „Geschlecht“ stehen die Parameter „männlich“ oder „weiblich“ in einer Auswahlliste zur Verfügung. Die Felder „Kassen-Nr.“ und „Versicherten-Nr“ sind Pflichtfelder. Die Informationen zur Kassen-Nr., Versicherten-Nr. und Versicherten-Status sind auf der Verordnung der SAPV-Leistung oder auf der Versichertenkarte des Patienten abgebildet. Zum Tag der Erstellung der Anwendung sind alle in Rheinland-Pfalz gültigen Kassennummern hinterlegt und werden nach der Eingabe in der Zelle rechts neben der Nummer angezeigt. Ist die Kassennummer vereinzelt nicht bekannt, kann ausnahmsweise der Kurzname der Kasse im Feld „Kassen-Nr.“ erfasst werden. Die Zelle ist auf 7 Zeichen begrenzt.

Mit der Abrechnung der Leistung ist im Abrechnungsdatensatz grundsätzlich ein Therapieendegrund anzugeben. Die gültigen Therapieendegründe können in der Auswahlliste der Zelle gewählt werden. Als Standardwert ist der Wert „6 in lfd. Behandlung“ vorgegeben. Ist der Patient verstorben, ist der Sterbeort anzugeben. Auch hier sind die Werte in einer Auswahlliste vorgegeben. Die Kontaktdaten Telefon, Handy und eMail des Patienten/Angehörigen und die Betreuerdaten dienen lediglich zur eigenen Information und sind <u>kein</u> Bestandteil des Abrechnungsdatensatzes.

**Verordnungsdaten**

Grundlage der Leistungserbringung und der späteren Abrechnung der Leistung ist die Verordnung Muster 63 (s. nächste Seite) durch einen behandelnden Arzt. Daher ist es erforderlich, die Verordnungsinformationen der Abrechnung und dem Leistungsnachweis zuzuordnen. Für die Erfassung der Verordnungsdaten sind die Zeilen 22 bis 84 reserviert. Pro Verordnung ist eine Zeile vorgesehen. Folgende Daten der Verordnung müssen erfasst werden:

- **Betriebsstättennummer** (BSNR) des verordnenden Arztes. Wurde die Verordnung von einem Krankenhausarzt ausgestellt, der nicht über eine BSNR als ermächtigter Arzt verfügt, ist die IKNR des Krankenhauses anzugeben.
- **Arztnummer**, bzw. lebenslange Arztnummer (LANR) des verordnenden Arztes. Liegt die LANR auf der Verordnung nicht vor (z. B. bei einer Verordnung durch einen Krankenhausarzt) kann das Feld leer bleiben.
- **Art der Verordnung** muss einen Wert der Auswahlliste enthalten. Zur Verfügung stehen Erstverordnung oder Folgeverordung.
- **Verordnungsdatum** ist das Datum der Verordnung und steht
- **Verordnungszeitraum** ist das Beginndatum und das Endedatum, das auf der Verordnung angegeben ist
- **Diagnosen** sind die Diagnosen, die auf der Verordnung angegeben wurden. Die Diagnosen sind nach der Inter-nationalen Klassifikation der Krankheiten in der jeweiligen vom Deutschen Institut für medizinische Dokumentation und Information (www.dimdi.de) im Auftrag des BMG herausgegebenen deutschen Fassung zu verschlüsseln. Die einzelnen Diagnosen müssen durch Semikolon getrennt werden. Beispiel: I63.1;M53.9
- **Komplexes Symptomgeschehen**. Die Felder können mit „Ja“ oder „Nein“ aus der Auswahlliste gefüllt werden. Es reicht aus, die angegebenen Felder der Verordnung mit „Ja“ zu füllen.
- Folgende **Maßnahmen** können auf der Verordnung angekreuzt sein: Beratung (B), Koordination (K), Teilversorgung (TV) und Vollversorgung (VV). Die entsprechenden Felder können aus der Auswahlliste mit den Werten „Ja“ oder „Nein“ belegt werden. Es reicht aus, die angekreuzten Maßnahmen mit dem Wert „Ja“ zu füllen.
- Nach Durchführung des Eingangsassessment sollte der **Bedarf** der Leistung festgelegt werden. Hier können die Felder KE (keine Leistung erforderlich), B (Beratung), K (Koordination), TV (Teilversorgung) und VV (Vollversorgung) mit dem Wert „Ja“ oder „Nein“ belegt werden. Es reicht aus, den erforderlichen Bedarf mit dem Wert „Ja“ zu kennzeichnen.

*Muster 63:*

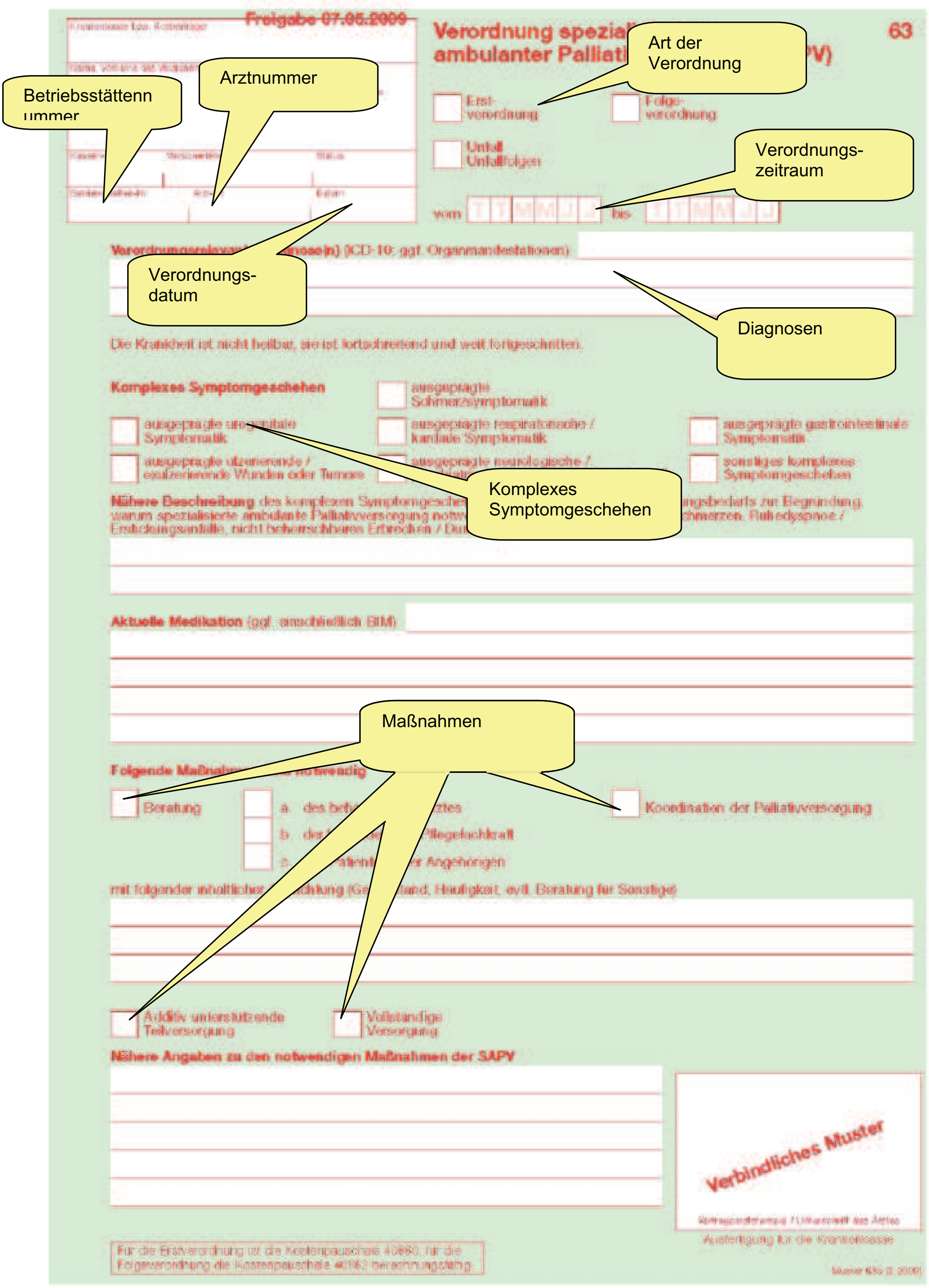
Freigabe 07.05.2009
Verordnung spezia[...] ambulanter Palliati[...]
63
Art der Verordnung
Arztnummer
Betriebsstättennummer
Erst-verordnung
Folge-verordnung
Unfall Unfallfolgen
Verordnungs-zeitraum
vom T T M M J J bis T T M M J J
Verordnungs-datum
(ICD-10: ggf. Organmanifestationen)
Diagnosen
Die Krankheit ist nicht heilbar, sie ist fortschreitend und weit fortgeschritten.
Komplexes Symptomgeschehen
ausgeprägte Schmerzsymptomatik
ausgeprägte urogenitale Symptomatik
ausgeprägte respiratorische / kardiale Symptomatik
ausgeprägte gastrointestinale Symptomatik
ausgeprägte ulzerierende / exulzerierende Wunden oder Tumore
ausgeprägte neurologische /
sonstiges komplexes Symptomgeschehen
Komplexes Symptomgeschehen
Nähere Beschreibung des komplexen Symptomgeschehens
Aktuelle Medikation (ggf. einschließlich BtM)
Maßnahmen
Folgende Maßnahmen sind notwendig
Beratung
Koordination der Palliativversorgung
mit folgender inhaltlicher
Additiv unterstützende Teilversorgung
Vollständige Versorgung
Nähere Angaben zu den notwendigen Maßnahmen der SAPV
Verbindliches Muster
Vertragsarztstempel / Unterschrift des Arztes
Ausfertigung für die Krankenkasse

**Erfassung und Übersicht weiterer Beteiligter**

Der Datensatz zur Abrechnung sieht vor, dass weitere am Behandlungsgeschehen beteiligte Leistungserbringer übermittelt werden. Des weiteren dienen diese Daten zur eigenen Information, um die Aufgaben der Koordination und Beratung wahrnehmen zu können. In den Zellen H5 bis S9 werden bis zu fünf beteiligte Leistungserbringer angezeigt. Die Liste ist nach dem Beginndatum der Leistung absteigend sortiert. Eine Erfassung der Leistungserbringer in diesem Tabellenblatt ist nicht vorgesehen. Nach betätigen der Schalftfläche „Beteiligte erfassen“ wird ein Tabellenblatt „Beteiligte“ geöffnet. Hier können die Daten weiterer beteiligter Leistungserbringer erfasst werden. Die Erfassung umfasst

- den Leistungszeitraum,
- die Art des Leistungserbringers, die in der Auswahlliste der Zelle hinterlegt ist,
- Betriebsstättennummer (BSNR) oder Institutionskennzeichen (IKNR) soweit vorhanden oder bekannt,
- die lebenslange Arztnummer, wenn es sich um einen Arzt handelt,
- Namen des Leistungserbringers,
- Vornamen des Leistungserbringers, wenn es sich um eine Person handelt
- Strasse des Leistungserbringers,
- Postleitzahl und Ort des Leistungserbringers.

Beim Verlassen des Tabellenblattes wird das Blatt zur besseren Übersicht automatisch ausgeblendet. Die Tabelle wird nach dem Beginndatum des Leistungszeitraumes sortiert, die Zeilen 4 bis 9 werden in der Übersicht über weitere Beteiligte im Tabellenblatt „Patientendaten“ angezeigt.

**Aktivitäten erfassen und anzeigen**

In den Zellen H13 bis N18 werden die letzten sechs Aktivitäten angezeigt. Die Erfassung der Aktivitäten dienen zum einen der eigenen Dokumentation und Übersicht über den Patienten. Zum anderen wird ein Teil der Aktivitäten bereits in den Leistungsnachweis zur Abrechnung eingetragen.

Die Erfassung der Aktivitäten erfolgt über die Schaltfläche „Aktivitäten erfassen“. Nach einem Klick auf die Schaltfläche wird ein Tabellenblatt zur Erfassung der Aktivitäten geöffnet. Hier können

- die Art der Aktivität,
- das Datum,
- der Beginn und das Ende der Betreungszeit (Uhrzeit),
- die gefahrenen Kilometer,
- die Anzahl der beteiligten Mitarbeiter,
- und ein Feld für weitere freie Informationen

erfasst werden.

Die Art der Aktivität ist mit folgenden Werten in einer Auswahlliste vorbelegt:
10 Beratung des Patienten
11 Beratung Hausarzt
12 Beratung Leistungserbringer
13 Beratung von Angehörigen
14 Dokumentation
15 Erhebung Eingangsassessment
16 Erstellung Notfallplan
30 Hausbesuch Arzt
40 Hausbesuch Pflegekraft
80 Hausbesuch sonstige Kraft
70 Hospizbesuch Arzt
82 Hospizbesuch sonstige Kraft
50 Pflegeheimbesuch Arzt
60 Pflegeheimbesuch Pflegekraft
81 Pflegeheimbesuch sonstige Kraft

90 sonstige
17 Teambesprechung

Anhand der ersten Zahl der Aktivität werden im Leistungsnachweis folgende Einträge erzeugt:
1 = Koordination)
3 = ärztl. Hausbesuch
4 = pfleg. Hausbesuch
5 = ärztl. Besuch (Pflege)
6 = pfleg. Besuch (Pflege)
7 = ärztl. Hospizbesuch

Des weiteren wird aus den erfassten Aktivitäten die Datensätze für den Datensatz 4 – SAPV-Fahrten des Anhang 1 der Anlage 3 – Vergütung und Leistungsbeschreibung des SAPV-Vertrages beim Drucken der Abrechnung erzeugt, wenn es sich bei der Aktivität um einen Besuch (3, 4, 5, 6, 7) des SAPV-Teams handelt. Aus den erfassten Daten der Aktivität „17 Teambesprechung“ wird beim Drucken der Abrechnung die Datensätze nach Datensatz 6 – SAPV-Teambesprechungen erstellt.

**Schaltfläche „eigene Daten ändern...“**
Mit der Schaltfläche „eigene Daten ändern...“ wird ein Pop-Up-Fenster mit den zu Beginn erfassten eigenen Daten angezeigt. Im Pop-Up-Fenster können die Daten angepasst werden. Beim Schließen des Fensters werden die Informationen in die „C:\sapv.xml“ geschrieben und stehen dort für alle angelegten SAPV-Excel-Dateien zur Verfügung.

Hinweis:
Änderungen an den Eigenen Daten haben Auswirkungen auf alle Dateien, die auf der Grundlage der Vorlage „SAPV-Patientenakte.XLT“ erstellt wurden.

*Schaltfläche „Abrechnung erzeugen“*
Beim Betätigen der Schaltfläche „Abrechnung erzeugen“ werden, für die gewählte Verordnung (Auswahlknopf aktiviert) Leistungsnachweis und Abrechnungsformular erzeugt. Die Abrechnung kann erst erzeugt werden, wenn die Arbeitsmappe gespeichert wurde. Es wird empfohlen, die Arbeitsmappen für die einzelnen Patienten in der gleichen Struktur in einem eigenen Verzeichnis abzuspeichern.

## Abrechnung erzeugen

Über die Schaltfläche „Abrechnung erzeugen“ im Tabellenblatt „Patientendaten“ werden Leistungsnachweis und Abrechnungsformular erzeugt. Beim Erzeugen des Leistungsnachweises wird eine 3stellige fortlaufende Nummer vergeben. Jeder erzeugte Leistungsnachweis und Abrechnungsbogen wird im Namen mit dieser Nummer ergänzt: Tabellenblatt: Leistungsnachweis_001, Abrechnung_001. Die 3stellige Nummer bildet zusammen mit der SAPV-Fallnummer die SAPV-Rechnungsnummer.

**Tabellenblatt Leistungsnachweis**
In der Zelle L2 kann nach der Erzeugung des Leistungsnachweises der Abrechnungsmonat gewählt werden. In der Auswahlliste werden alle Monate (im Format MM/JJJJ) des Zeitraums der gewählten Verordnung angezeigt. Nachdem der Abrechnungsmonat gewählt wurde, werden in den Zeilen 9 bis 57 des Tabellenblattes erfasste Daten des Tabellenblatts Aktivitäten mit den entsprechenden Uhrzeiten gefüllt.

*Mögliche Korrekturen im Leistungsnachweis*
Das Programm ermittelt beim Erzeugen der Abrechnungsdaten, ob sich der Patient am Tag des Beginns des Verordnungszeitraums in einer Pflegeeinrichtung befand. Daher wird entweder der Abrechnungskomplex „1 Koordination (häusl.)“ oder „2 Koordination (Pflege)“ mit Daten der Aktivitäten befüllt. Tatsächlich kann aber in Einzelfällen während des gesamten Abrechnungszeitraums ein Wechsel zwischen der Unterbringung in einer stationären

Pflegeeinrichtung und einer häuslichen Unterbringung stattfinden. Hier ist eine manuelle Korrektur des Leistungsnachweises erforderlich.

Des weiteren ist es möglich, dass an einem Leistungstag mehrere Besuche der SAPV-Pflegekraft oder des SAPV-Arztes notwendig waren. Diese zusätzlichen Hausbesuche können in den Zeilen 16 bis 18, 30 bis 32, 44 bis 46 und 58 bis 60 ergänzt werden.

*Schaltfläche „Abrechnungsbogen anzeigen/ausblenden“*

Um die Anzahl der Tabellenblätter zur besseren Übersicht zu begrenzen, wird das Tabellenblatt Abrechnung standardmäßig ausgeblendet. Um ggf. vor dem Druck der Abrechnung und des Leistungsnachweises die Abrechnung zu überprüfen, kann das Tabellenblatt Abrechnung eingeblendet werden. Ist das Tabellenblatt bereits eingeblendet, wird es beim Betätigen der Schaltfläche ausgeblendet.

*Schalftfäche „Leistungsnachweis und Abrechnungsbogen drucken und Datensätze erzeugen“*

Durch einen Klick auf die Schaltfläche wird der aktive Leistungsnachweis und das dazugehörige Tabellenblatt „Abrechnung“ gedruckt. Im Anschluss werden die Dateien zur Abrechnung entsprechend der Datensatzbeschreibung (Anhang 1 der Anlage 3 – Vergütung und Leistungsbeschreibung des SAPV-Vertrages) erzeugt. Da nach der Erzeugung der Datensätze keine Korrektur mehr möglich ist, ist es sinnvoll, Leistungsnachweis und Abrechnungsbogen vor dem Druck zu überprüfen.

*Anlage 4*

# *Leitfaden für einen Antrag / Konzeption zur spezialisierten ambulanten Palliativversorgung gem. § 132d SGB V in Rheinland-Pfalz*

| ***Inhalt der Konzeption „SAPV“*** | ***Kurzbeschreibung, was zu diesem Punkt angegeben werden soll*** |
|---|---|
| → *Projekt / Netzwerk* | Bezeichnung des spezialisierten Leistungserbringers |
| → *Inhalt des Projekts* | Beschreiben Sie hier bitte den Inhalt Ihres Versorgungsmodells. Aus welchen „klassischen“ Bereichen werden Leistungen miteinander verzahnt. Worin besteht die Versorgungsverbesserung zur bisherigen Versorgung. Welche verbindlichen Regelungen zur Zusammenarbeit der Kooperationspartner untereinander sind vorgesehen.<br><br>Zu den nachfolgenden Punkten sollten Sie in Ihrer Konzeption mindestens Stellung nehmen:<br><br>➢ Standort des Leistungserbringers,<br>➢ Versorgungsgebiet,<br>➢ Versorgungsprozess,<br>➢ Kooperationspartner,<br>➢ Kooperationsvereinbarungen mit im Rahmen des Netzwerkes an der Versorgung Beteiligten im Versorgungsgebiet (z. B. ambulante Hospizdienste, Seelsorge) zur Sicherung der psychosozialen Unterstützung,<br>➢ Mitarbeiterkapazität (Stellenplan),<br>➢ Personelle Zuständig- und Verantwortlichkeiten im SAPV-Team (u. a. Weisungsbefugnisse, Weisungsgebundenheit),<br>➢ Qualifikationsnachweise der beteiligten Personen,<br>➢ sächliche Ausstattung,<br>➢ Qualitätssicherungsmaßnahmen,<br>➢ wie wird die reibungslose Versorgung mit Arznei-, Heil- und Hilfsmitteln sichergestellt,<br>➢ wie wird die psychosoziale und seelische Unterstützung sichergestellt,<br>➢ wie wird die 24-Stunden-Rufbereitschaft sichergestellt. |

| | |
|---|---|
| → *Region* | Geben Sie uns bitte die Versorgungsregion an, in welcher die SAPV realisiert und der Versorgungsauftrag übernommen werden soll. |
| → *Kooperationspartner* | Nennen Sie bitte alle Kooperationspartner, die an der SAPV mitwirken sollen rsp. mit welchen der Vertrag geschlossen werden soll. Welchen Zulassungsstatus bringen diese Kooperationspartner in die SAPV ein. Beschreiben Sie die regionalen Netzwerkstrukturen, in die Sie sich als SAPV-Leistungserbringer integrieren. |
| → *Rechtsformen* | Gibt es in Ihrer geplanten SAPV eine Rechtsform, wie zum Beispiel ein Verein, GbR, GmbH, Genossenschaft, AG, etc.? Geben Sie uns dies bitte konkret an. |
| → *Ansprechpartner* | Wer ist für die Krankenkassen in Rheinland-Pfalz primärer Ansprechpartner für Ihr SAPV-Konzept? |
| → *Qualitätsmanagementtools* | Welche Qualitätsmanagementtools kommen in Ihrem Konzept zum Einsatz (wie Leitlinien, allgemeine und/oder medizinische Qualitätsindikatoren, Qualitätszirkel, Fallkonferenzen, Fort- und Weiterbildung der Leistungserbringer etc.)? |
| → *Qualitätsmessung* | Wie soll in Ihrem Konzept die SAPV-Versorgungsqualität gemessen werden. |
| → *Organisatorische Anforderungen / wirtschaftliche Verantwortung sowie Prozessverantwortung und Controlling* | Zu den organisatorischen Anforderungen der SAPV sollte festgelegt / definiert sein, wie eine Zuordnung der wirtschaftlichen Verantwortung und der Prozessverantwortung der Leistungserbringer definiert sein soll.<br>Sie müssen hier angeben, wie die Zusammenarbeit der Kooperationspartner untereinander geregelt sein soll und wie interne organisatorische Abläufe der SAPV transparent dargestellt werden können.<br>Wir bitten auch um Angabe, ob und welchem Umfang ein internes Controlling angedacht ist. |
| → *Datentransparenz gegenüber den Krankenkassen in Rheinland-Pfalz* | In welchem Format können Sie Ihre Daten aus der SAPV den Krankenkassen in Rheinland-Pfalz elektronisch zur Verfügung zu stellen. |
| → *Mein Projekt / Konzept ist attraktiv, weil ...* | Beschreiben Sie hier in kurzen Worten bitte die Attraktivität Ihres SAPV-Versorgungsprojektes |

## *Ihr Konzept schicken Sie bitte an*

| | |
|---|---|
| AOK – Die Gesundheitskasse in Rheinland-Pfalz<br>Bereich 4.2.1.2<br>Frau Alexandra Schanzenbach<br>Virchowstr. 30<br>67304 Eisenberg | Vdek e.V.<br>Landesvertretung Rheinland-Pfalz<br>Bereich SAPV<br>Wilhelm-Theodor-Römheld-Straße 22<br>55130 Mainz |
| BKK-LKK-Arbeitsgemeinschaft<br>Referat Pflege<br>Essenheimer Str. 126<br>55128 Mainz | IKK Südwest<br>Herrn Lars Poppe<br>Berliner Promenade 1<br>66111 Saarbrücken |
| Vdek e.V.<br>Landesvertretung Saarland<br>Bereich SAPV<br>Talstr. 30<br>66119 Saarbrücken | Knappschaft<br>Regionaldirektion Saarbrücken<br>Bereich SAPV<br>St. Johanner Str. 46-48<br>66111 Saarbrücken |

*Anlage 5 –Ergebnis Assessment (Krankenkasse und Verordner)*

<table>
<tr><td rowspan="6">Name<br>Vorname<br>Straße, Wohnort<br><br>Tel.:<br>Gesetzl. Vertreter:<br><br><br>Informationsberechtigter Angehöriger:</td><td>1. SAPV-Fall-Nr.</td><td colspan="2"></td></tr>
<tr><td>2. Geburts-datum</td><td colspan="2"></td></tr>
<tr><td>3. Geschlecht</td><td>☐ weiblich</td><td>☐ männlich</td></tr>
<tr><td>4. Datum: Erst-Verordnung</td><td>Datum:<br>bis</td><td>Umfang:<br>□ B □ K □ TV □ VV</td></tr>
<tr><td>4. Datum: Folge-verordnung</td><td>Datum:<br>bis</td><td>Umfang:<br>□ B □ K □ TV □ VV</td></tr>
<tr><td colspan="3">SAPV-Team:<br><br>Hausarzt:<br><br>Häusl. Krankenpflege:</td></tr>
</table>

**ICD 10:** ☐ ☐☐ ☐☐

**Kurative Behandlungen sind abgeschlossen:** □ ja □ nein

Die **Mittel der Regelversorgung/der allgemeinen Palliativversorgung reichen nicht** aus weil,________________________________________

**Komplexes Symptomgeschehen nach SAPV – RL**: □ liegt vor □ liegt nicht vor
Zutreffende Bereiche ankreuzen – die Ausprägung **muss** beschrieben werden:

☐ ausgeprägte Schmerzsymptomatik____________________________

☐ ausgeprägte respiratorische/kardiale Symptomatik____________________

☐ ausgeprägte gastrointestinale Symptomatik________________________

☐ ausgeprägte urogenitale Symptomatik___________________________

☐ ausgeprägte ulzerierende/exulzerierende Wunden/Tumore________________

☐ ausgeprägte neurologische/psychiatrische/psychische Symptomatik____________

☐ Sonstiges komplexes Symptomgeschehen:_________________________

Der Patient / die Patientin benötigt die Leistungen der SAPV: □ ja □ nein

**Folgende SAPV-Leistungen sind notwendig**:

□ **Beratung** zu folgendem Sachverhalt________________________________
In die Beratung werden folgende Personen/Leistungserbringer einbezogen:____________
____________________________________________________

□ **Koordination** folgender Versorgungsnotwendigkeiten:________________________
____________________________________________________
____________________________________________________

**Behandlungsplan muss abgestimmt werden mit folgenden Leistungserbringern**:__________

☐ Hausarzt ☐ Apotheke ☐ Pflegeeinrichtung ☐ Physiotherapeut
☐ Facharzt ☐ Sanitätshaus ☐ Hospiz ☐ Logopädie
☐ Kinderarzt ☐ Sonstige:______________________________________________________________

**Behandlungsplan ist**: ☐ konsentiert ☐ teilweise konsentiert ☐ muss noch abgestimmt werden

☐ Notfallplan erstellt ☐ Rufbereitschaft organisiert

□ **Teilversorgung** folgender

pflegerischer Leistungen____________________________________________________________________

____________________________________________________________________________________

medizinischer Leistungen____________________________________________________________________

____________________________________________________________________________________

Geschätzter zeitlicher Aufwand/Tag:________________________________________min.
Häufigkeit/Woche:________________________________
geschätzte Fahrtzeiten:_______________________________________min.

□ **Vollversorgung** folgender

pflegerischer Leistungen____________________________________________________________________

____________________________________________________________________________________

medizinischer Leistungen____________________________________________________________________

____________________________________________________________________________________

Geschätzter zeitlicher Aufwand/Tag:________________________________________min.
Häufigkeit/Woche:__________________________________
geschätzte Fahrtzeiten:_______________________________________min.

**SAPV-Verordnung:**

□ Es besteht kein Bedarf an SAPV. Die Mittel der Regelversorgung reichen aus.

□ SAPV –Verordnung liegt nach Anspruch und Bedarf vor.

□ SAPV – Verordnung muss gemäß dem oben genannten Leistungsbedarf folgendermaßen

____________________________________________________________________________________

__________________________________________________________________________angepasst werden.

□ SAPV – Folgeverordnung muss nach dem oben genannten Leistungsbedarf bis zum ________ vorliegen.

Assessment durchgeführt am: ________________________________

Unterschrift der / des Durchführenden: _________________________________

*Anlage 6 (Muster)*

| **Leistungsnachweis:** | Krankenkasse: | Monat/Jahr | Pflegestufe: ☐ I ☐ II ☐ III ☐ III-+ | **SAPV-Team (Anschrift)** | **IK-Nr.** |
|---|---|---|---|---|---|
| **Name, Vorname** | | Vers. -Nr. | Geb.-Datum: | | **Telefon:** |

| **Datum** | **1.** | | | **2.** | | | **3.** | | | **4.** | | | **5.** | | | **6.** | | | **7.** | | | **8.** | | |
|---|---|---|---|---|---|---|---|---|---|---|---|---|---|---|---|---|---|---|---|---|---|---|---|---|
| Leistungssummer gemäß Anlage 3 | Uhrzeit von / bis | HZ SAPV | HZ Vers. | Uhrzeit von / bis | HZ SAPV | HZ Vers. | Uhrzeit von / bis | HZ SAPV | HZ Vers. | Uhrzeit von / bis | HZ SAPV | HZ Vers. | Uhrzeit von / bis | HZ SAPV | HZ Vers. | Uhrzeit von / bis | HZ SAPV | HZ Vers. | Uhrzeit von / bis | HZ SAPV | HZ Vers. | Uhrzeit von / bis | HZ SAPV | HZ Vers. |
| | | | | | | | | | | | | | | | | | | | | | | | | |
| | | | | | | | | | | | | | | | | | | | | | | | | |
| | | | | | | | | | | | | | | | | | | | | | | | | |
| | | | | | | | | | | | | | | | | | | | | | | | | |
| | | | | | | | | | | | | | | | | | | | | | | | | |
| | | | | | | | | | | | | | | | | | | | | | | | | |
| | | | | | | | | | | | | | | | | | | | | | | | | |
| | | | | | | | | | | | | | | | | | | | | | | | | |
| | | | | | | | | | | | | | | | | | | | | | | | | |
| | | | | | | | | | | | | | | | | | | | | | | | | |

| **Datum** | **8.** | | | **9.** | | | **10.** | | | **11.** | | | **12.** | | | **13.** | | | **14.** | | | **15.** | | |
|---|---|---|---|---|---|---|---|---|---|---|---|---|---|---|---|---|---|---|---|---|---|---|---|---|
| Leistungssummer gemäß Anlage 3 | Uhrzeit von / bis | HZ SAPV | HZ Vers. | Uhrzeit von / bis | HZ SAPV | HZ Vers. | Uhrzeit von / bis | HZ SAPV | HZ Vers. | Uhrzeit von / bis | HZ SAPV | HZ Vers. | Uhrzeit von / bis | HZ SAPV | HZ Vers. | Uhrzeit von / bis | HZ SAPV | HZ Vers. | Uhrzeit von / bis | HZ SAPV | HZ Vers. | Uhrzeit von / bis | HZ SAPV | HZ Vers. |
| | | | | | | | | | | | | | | | | | | | | | | | | |
| | | | | | | | | | | | | | | | | | | | | | | | | |
| | | | | | | | | | | | | | | | | | | | | | | | | |
| | | | | | | | | | | | | | | | | | | | | | | | | |
| | | | | | | | | | | | | | | | | | | | | | | | | |
| | | | | | | | | | | | | | | | | | | | | | | | | |
| | | | | | | | | | | | | | | | | | | | | | | | | |
| | | | | | | | | | | | | | | | | | | | | | | | | |
| | | | | | | | | | | | | | | | | | | | | | | | | |
| | | | | | | | | | | | | | | | | | | | | | | | | |

| Datum | 16. | | | 17. | | | 18. | | | 19. | | | 20. | | | 21. | | | 22. | | | 23. | | |
|---|---|---|---|---|---|---|---|---|---|---|---|---|---|---|---|---|---|---|---|---|---|---|---|---|
| Leistungsummer gemäß Anlage 3 | Uhrzeit von / bis | HZ SAPV | HZ Vers. | Uhrzeit von / bis | HZ SAPV | HZ Vers. | Uhrzeit von / bis | HZ SAPV | HZ Vers. | Uhrzeit von / bis | HZ SAPV | HZ Vers. | Uhrzeit von / bis | HZ SAPV | HZ Vers. | Uhrzeit von / bis | HZ SAPV | HZ Vers. | Uhrzeit von / bis | HZ SAPV | HZ Vers. | Uhrzeit von / bis | HZ SAPV | HZ Vers. |
| | | | | | | | | | | | | | | | | | | | | | | | | |
| | | | | | | | | | | | | | | | | | | | | | | | | |
| | | | | | | | | | | | | | | | | | | | | | | | | |
| | | | | | | | | | | | | | | | | | | | | | | | | |
| | | | | | | | | | | | | | | | | | | | | | | | | |
| | | | | | | | | | | | | | | | | | | | | | | | | |
| | | | | | | | | | | | | | | | | | | | | | | | | |
| | | | | | | | | | | | | | | | | | | | | | | | | |
| | | | | | | | | | | | | | | | | | | | | | | | | |
| | | | | | | | | | | | | | | | | | | | | | | | | |

| Datum | 24. | | | 25. | | | 26. | | | 27. | | | 28. | | | 29. | | | 30. | | | 31. | | |
|---|---|---|---|---|---|---|---|---|---|---|---|---|---|---|---|---|---|---|---|---|---|---|---|---|
| Leistungsummer gemäß Anlage 3 | Uhrzeit von / bis | HZ SAPV | HZ Vers. | Uhrzeit von / bis | HZ SAPV | HZ Vers. | Uhrzeit von / bis | HZ SAPV | HZ Vers. | Uhrzeit von / bis | HZ SAPV | HZ Vers. | Uhrzeit von / bis | HZ SAPV | HZ Vers. | Uhrzeit von / bis | HZ SAPV | HZ Vers. | Uhrzeit von / bis | HZ SAPV | HZ Vers. | Uhrzeit von / bis | HZ SAPV | HZ Vers. |
| | | | | | | | | | | | | | | | | | | | | | | | | |
| | | | | | | | | | | | | | | | | | | | | | | | | |
| | | | | | | | | | | | | | | | | | | | | | | | | |
| | | | | | | | | | | | | | | | | | | | | | | | | |
| | | | | | | | | | | | | | | | | | | | | | | | | |
| | | | | | | | | | | | | | | | | | | | | | | | | |
| | | | | | | | | | | | | | | | | | | | | | | | | |
| | | | | | | | | | | | | | | | | | | | | | | | | |
| | | | | | | | | | | | | | | | | | | | | | | | | |
| | | | | | | | | | | | | | | | | | | | | | | | | |

| Anzahl Komplexe gesamt | Komplexe Preis einzeln | Gesamtpreis |
|---|---|---|
| | | |
| | | |
| | | |
| | | |
| | | |

| | |
|---|---|
| Ich bestätige die vertragsmäßige Ausführung der o.a. Leistungen:<br><br>Datum:<br><br>Unterschrift Verantwortlicher SAPV-Team: | Datum:<br><br>Unterschrift Patient/gesetzl. Vertreter od. Bevollmächtigter |

*Anlage 7 (Muster)*

# Einverständnis- /Teilnahmeerklärung (Versicherter)

## Meine Personalien:

| | |
|---|---|
| *ggf. Eindruck oder Aufkleber aus Praxissoftware* | Name |
| | Vorname |
| | Adresse |
| | Geburtsdatum |
| | KV-Nr. |
| gesicherte Behandlungsdiagnosen (nach ICD 10) | |
| mein Hausarzt (Name und Anschrift) | |

**Hiermit erkläre ich meine Teilnahme an der**

**„spezialisierten ambulanten Palliativversorgung“.**

**Die entsprechenden Informationen zum Betreuungskonzept habe ich erhalten.**

Mir ist bekannt, dass in diesem Betreuungskonzept über meine Behandlung durch Ärzte, Pflegepersonal und andere Leistungserbringer in dieser Versorgungsform Aufzeichnungen gemacht werden. Durch mein Vertrauensverhältnis zu Ärzten und Pflegepersonal, etc. enthalten diese Aufzeichnungen Angaben, die meine Privatsphäre betreffen und somit einer gesetzlichen **Geheimhaltungspflicht** unterliegen.

Von mir in Patientenbefragungen in **Zusammenarbeit mit Kooperationspartnern (wie z. B.: Frau / Herrn Doktor, Krankenpflegekraft, etc.)** gemachte Angaben dürfen in dieser Versorgung dokumentiert und an die Leistungserbringer und meine Krankenkasse im jeweils erforderlichen Umfang ggf. für eine Projektauswertung / -analyse weitergeleitet und ausgewertet werden.

Ich erkläre ausdrücklich, dass meine Teilnahme an dieser **„spezialisierten ambulanten Palliativversorgung“** freiwillig ist.

Mir ist bekannt, dass ich meine Teilnahmeerklärung schriftlich widerrufen kann.

**Datum / Unterschrift der / des Versicherten, ggf. gesetzlicher Vertreter oder Betreuer**

*Anlage 8 (Muster)*

*Name:*
*Vorname:*
*Straße:*
*Wohnort:*
*Tel.:*
*Handy:*

***Bevollmächtigte Personen / Gesetzl. Betreuer:*** ☐ ja ☐ nein

Name:
Bezug:
Telefonnummer:

| | |
|---|---|
| **1. ID-NR** | |
| **2. Geburtsdat.** | |
| **3. Geschlecht** | ☐ weiblich ☐ männlich |
| **4. Nationalität:** | |
| | Dolmetscher benötigt: ☐ ja ☐ nein |
| **5. Konfession:** | |
| **6. Erstverordnung** | Datum bis: Umfang: ☐ B ☐K ☐ TV ☐ VV |
| **7. Folgeverordnung** | Datum bis: Umfang: ☐ B ☐K ☐ TV ☐ VV |

**8b Tumorausbreitung / Metastasen**

☐ Hirn ☐ Leber
☐ Lunge ☐ Knochen
☐ Sonstige:

**8a Diagnosen / Probleme: wenn keine ICD bitte Text eintragen**

**Hauptdiagnose- ICD-10** ☐ ☐☐ ☐☐

**Nebendiagnose-ICD-10** ☐ ☐☐ ☐☐

☐ internistische Erkrankung ____________________
☐ neurologische Erkrankung ____________________
☐ Sonstige Erkrankung ____________________

**9. Absprachen/Vorlagen**

☐ Notfallplan:________________________________
☐ Patientenverfügung: ________________________________
☐ Vorsorgevollmacht: ________________________________
☐ Betreuung: ________________________________
☐ Sonstiges (Bitte ausformulieren):____________________

**10. Behandlungsort Aktuell / geplant**

☐ zu Hause
 ☐ allein - Hausnotruf ☐ ja ☐ nein ☐ beantragt:
 ☐ mit versorgenden Angehörigen

☐ Pflegeheim
☐ stationäres Hospiz, angemeldet am:____________________
☐ Krankenhaus - Palliativstation
☐ Krankenhaus - Allgemeinstation
☐ Sonstiger:________________________________

**11. Aktuelle medizinische/pflegerische Behandler**

☐ Hausarzt ________________________________
☐ Facharzt ________________________________
☐ Palliativarzt (QPA)
☐ Pflegedienst ________________________________
☐ ambulantes Hospiz ________________________________
☐ Palliativberatung(AHPB) ☐ Ehrenamtlicher Dienst
☐ Sonstige (bitte angeben)____________________
________________________________

**12. Pflegestufe**

☐ keine ☐ Stufe I ☐ Stufe II ☐ Stufe III ☐ Stufe III +

☐ beantragt am: ☐ Widerspruch eingelegt am: ☐ Widerspruch abgelehnt am:

☐ Höherstufung beantragt am: ☐ MDK – Termin am:

**13. Schwerbehindertenausweis:**

☐ ja ☐ Aktenzeichen: ________________________________
☐ nein ☐ Grad der Behinderung:________________________________
☐ beantragt am:________________ ☐ Verschlimmerungsantrag gestellt am: ____________________

**14. Sonstiges**

Zuzahlungsbefreiung beantragt ☐ ja, am______________(Datum) ☐ genehmigt______________(Jahr)

| **Patient: Krankenversicherung** | |
|---|---|
| Krankenkasse: | Versicherten-Nr.: |
| | Familienversichert über: |
| ☐selbstversichert | Name Vorname des Mitglieds: |
| Ansprechpartner Krankenkasse: | Zuständige Geschäftsstelle: |
| Tel.: | |

| **Patient: gesetzl. Vertreter / sorgeberechtigte Personen** | | ■ **keine** |
|---|---|---|
| Name u. Geburtsname: | | Vorname: |
| Straße | | Bezug zum Patient: |
| PLZ, Wohnort | Tel.: | Handy: |
| Bemerkungen: | | |
| Name u. Geburtsname: | | Vorname: |
| Straße: | | Bezug zum Patient: |
| PLZ, Wohnort: | Tel.: | Handy: |
| Bemerkungen: | | |

| **Angehöriger: zeichnungsberechtigt / bevollmächtigt** | | ■ **keine** |
|---|---|---|
| Name: | Vorname: | Bezug zum Pat.: |
| Straße: | PLZ: | Ort: |
| Telefon: | | Handy: |
| Name | Vorname: | Bezug zum Pat.: |
| Straße | PLZ: | Ort: |
| Telefon | | Handy: |
| Bemerkungen: | | |

| **Angehörige: informationsberechtigt** | | ■ **keine** |
|---|---|---|
| Name: | Vorname | Bezug zum Pat.: |
| Straße: | PLZ: | Ort: |
| Telefon: | | Handy: |
| Bemerkungen: | | |

## SAPV - Team

| | | |
|---|---|---|
| SAPV-Team in: | | **Telefon:** |
| Straße: | | |
| Kontaktperson ( Arzt / Ärztin ) | | Telefon: |
| | | Handy: |
| Kontaktperson ( Pflege ) | | Telefon: |
| | | Handy: |
| Kontaktperson (Sozialarbeit ) | | Telefon: |
| | | Handy |

## Hausärztliche / Fachärztliche Versorgung Regelversorgung

| | | |
|---|---|---|
| Name Praxis | | Ansprechpartner |
| Straße | PLZ | Ort |
| Telefon | | Handy |
| Bemerkungen (bitte angeben, ob Haus- oder Facharzt über eine palliativmed. Weiterbildung verfügt): | | |
| Name | | Ansprechpartner |
| Straße | PLZ | Ort |
| Telefon | | Handy |
| Bemerkungen | | |

## *betreuender Pflegedienst ( HKP)* ■ kein

| | | |
|---|---|---|
| Name Dienst | | Ansprechpartner |
| Straße | PLZ | Ort |
| Telefon | | Handy |
| Bemerkungen (bitte angeben, ob Haus- oder Facharzt über eine palliativmed. Weiterbildung verfügt): | | |

## Hospiz ■ kein

| | | |
|---|---|---|
| Name | | |
| Straße | PLZ | Ort |
| Telefon | | Handy |
| Bemerkungen | | |

## Krankenhaus ■ kein

| | | |
|---|---|---|
| Name | PLZ | Ort |
| Telefon | | |
| Name | PLZ | Ort |
| Telefon | | |
| Bemerkungen | | |

| **Apotheke** | | | ■ **keine** | | |
|---|---|---|---|---|---|
| **Name**<br>Straße<br>Telefon | PLZ | | Ort | | |
| **Bemerkungen** | | | | | |
| **Sanitätshaus** | | | ■ **kein** | | |
| **Name**<br>Straße<br>Telefon | PLZ | | Ort | | |
| **Sonstige Leistungserbringer**<br><br>**(z.B. Psychologe /Dolmetscher / Physiotherapeuten / Dienst zur Organisation der parenteralen Ernährung)** | | | ■ **keine** | | |
| **Name:**<br>**Telefon:**<br>**Bemerkungen:** | | | | | |
| **Name:**<br>**Telefon:**<br>**Bemerkungen:** | | | | | |
| **Name:**<br>**Telefon:**<br>**Bemerkungen:** | | | | | |

Erhebung durchgeführt von:__________________________ Datum:___________________________

(Berufsbezeichnung)_________________________________ Unterschrift:_______________________

## 15. aktuelle Versorgungssituation Medizin und Pflege

☐ **Shunt** ________________________________

☐ Shuntversorgung ________________________ ____

☐ **Port**________________________________

☐ Portnadel________________________________

☐ Parenterale Ernährung____________________________

☐ Portversorgung_____________________________

☐ **PEG**________________________________

☐ Enterale Ernährung ______________________________

☐ PEG Versorgung____________________________

☐ **AP** ________________________________

☐ AP Versorgung____________________________

________________________________

☐ **Darmspülung, therapeutische Abführmaßnahmen**

________________________________

☐ **Urostoma**________________________________

☐ Urostomaversorgung____________________________

☐ **Strahlentherapie**____________________________

☐ **Chemotherapie**______________________________

☐ **Opioide der WHO Stufe III** ☐ **Steroide**

☐ **Wundversorgung**______________________________

________________________________

________________________________

☐ **Lagerungsbehandlung (z.B. Spezialbett)**____________

________________________________

☐ **Delegationsvereinbarung**____________________________

________________________________

________________________________

☐ **Tracheostoma**____________________________

☐ Tracheostomaversorgung______________________

☐ **Absaugen**______________________________

☐ **Beatmen**______________________________

☐ **Sauerstoffgabe**____________________________

☐ **Inhalation**______________________________

☐ **Injektionen / Infusionen / Schmerzpumpe**

________________________________

________________________________

☐ **Transfusion (EK/TK)**________________________

________________________________

☐ **Ascitespunktion**____________________________

☐ **Pleuraerguss**______________________________

☐ **Pleurapunktion**____________________________

☐ **Transurethraler Blasenkatheter, letzter Wechsel:**

________________________________

CH:________________________________

☐ **Grundpflege**______________________________

________________________________

________________________________

☐ **Behandlungspflege**__________________________

________________________________

________________________________

☐ **hauswirtschaftliche Versorgung**______________

________________________________

☐ **Sonstiges**______________________________

________________________________

## 16. Physikalische und andere Therapien

☐ Krankengymnastik / Physiotherapie__________________________

☐ Lymphdrainage________________________________

☐ Ergotherapie________________________________

☐ Naturheilverfahren______________________________

☐ Logopädie______________________________

☐ Psychologische Betreuung__________________

☐ Sonstiges______________________________

________________________________

## 17. Hilfsmittel

| | Leihgabe von | Eigentum | Beantragt am: | | Leihgabe von | Eigentum | Beantragt am |
|---|---|---|---|---|---|---|---|
| ☐ Rollstuhl | | | | ☐ Duschhocker | | | |
| ☐ Gehwagen/Rollator | | | | ☐ Absauggerät | | | |
| ☐ Pflegebett | | | | ☐ Sauerstoff-konzentrator | | | |
| ☐ elektr. verstellbarer Einlegerahmen | | | | | | | |
| ☐ Ernährungs-pumpe | | | | ☐ Toilettenstuhl | | | |
| ☐ Beatmungsgerät | | | | ☐ Toilettensitz-erhöhung | | | |
| ☐ Infusionspumpe | | | | ☐ Badewannenlifter | | | |
| ☐ PCA Pumpe | | | | ☐ Antidekubitus-matratze | | | |
| ☐ Gehhilfe/Gehstock | | | | ☐ Sonstiges | | | |

## 18.Symptomklassifikation

**Einschätzung unter Anwendung der vereinbarten Legende**

| | | kein | leicht | mittel | stark | Nicht be-urteilbar | Kommentar |
|---|---|---|---|---|---|---|---|
| **Gastrointestinale Symptome** | | | | | | | |
| Appetitmangel | | ☐ | ☐ | ☐ | ☐ | ☐ | |
| Mundtrockenheit | | ☐ | ☐ | ☐ | ☐ | ☐ | |
| Schluckbeschwerden | | ☐ | ☐ | ☐ | ☐ | ☐ | |
| Blutungen | | ☐ | ☐ | ☐ | ☐ | ☐ | |
| Mucositis | WHO | ☐ | ☐ | ☐ | ☐ | ☐ | |
| Übelkeit | WHO | ☐ | ☐ | ☐ | ☐ | ☐ | |
| Erbrechen | WHO | ☐ | ☐ | ☐ | ☐ | ☐ | |
| Obstipation | WHO | ☐ | ☐ | ☐ | ☐ | ☐ | |
| Diarrhoe | WHO | ☐ | ☐ | ☐ | ☐ | ☐ | |
| Kachexie | | ☐ ja | | ☐ nein | | | |
| **Urogenitale Symptome** | | | | | | | |
| Harnverhalt | | ☐ ja | | ☐ nein | | ☐ | |
| Urininkontinenz | | ☐ ja | | ☐ nein | | ☐ | |
| Hämaturie | | ☐ ja | | ☐ nein | | ☐ | |
| Pyurie | | ☐ ja | | ☐ nein | | ☐ | |
| Renale Ödeme | | ☐ ja | | ☐ nein | | ☐ | |
| Renale Hypertonie | | ☐ ja | | ☐ nein | | ☐ | |
| Oligurie | | ☐ ja | | ☐ nein | | ☐ | |
| Polyurie | | ☐ ja | | ☐ nein | | ☐ | |
| Anurie | | ☐ ja | | ☐ nein | | ☐ | |
| **Neurologische Symptome** | | | | | | | |
| Schwierigkeiten bei Bewegung | | ☐ | ☐ | ☐ | ☐ | ☐ | |
| Spastik | | ☐ ja | | ☐ nein | | ☐ | |
| Kontraktur | | ☐ ja | | ☐ nein | | ☐ | |
| Lähmungen | | ☐ ja | | ☐ nein | | ☐ | |
| Myoklonien | | ☐ ja | | ☐ nein | | ☐ | |
| Kribbeln, Taubheit: Hände/Füße | | ☐ ja | | ☐ nein | | ☐ | |
| Krampfanfälle | | ☐ ja | ☐ nein | ☐ fokal | ☐ generalisiert | | |
| Unruhe | | ☐ ja | | ☐ nein | | ☐ | |
| Somnolenz | | ☐ ja | | ☐ nein | | ☐ | |

| | kein | leicht | mittel | stark | Nicht beurteilbar | Kommentar |
|---|---|---|---|---|---|---|
| Verstehen | □ | □ | □ | □ | □ | |
| Verständlichkeit | □ | □ | □ | □ | □ | |
| Soziale Interaktion | □ | □ | □ | □ | □ | |
| Problemlösen | □ | □ | □ | □ | □ | |
| Gedächtnis/Lernfähigkeit/ Orientierung | □ | □ | □ | □ | □ | |
| Desorientiertheit | | | | | | |
| ▪ Zur Person | □ ja | | □ nein | | □ | |
| ▪ Zum Ort | □ ja | | □ nein | | □ | |
| ▪ Zur Zeit | □ ja | | □ nein | | □ | |
| ▪ Zur Situation | □ ja | | □ nein | | □ | |
| **Emotionale Symptome** | | | | | | |
| Angst | □ ja | | □ nein | | □ | |
| Konzentrationsschwierigkeiten | □ ja | | □ nein | | □ | |
| Besorgtheit | □ ja | | □ nein | | □ | |
| Anspannung, Aufregung, | □ ja | | □ nein | | □ | |
| Nervosität, Reizbarkeit | □ ja | | □ nein | | □ | |
| Traurigkeit, Antriebsarmut | □ ja | | □ nein | | □ | |
| Depressivität | □ ja | | □ nein | | □ | |
| **Respiratorische / Kardiale Symptome** | | | | | | |
| Dyspnoe | □ ja | | □ nein | | □ | |
| Apnoe | □ ja | | □ nein | | □ | |
| Zyanose | □ ja | | □ nein | | □ | |
| Husten | □ ja | | □ nein | | □ | |
| Hypersekretion/-salivtation | □ ja | | □ nein | | □ | |
| Rasseln | □ ja | | □ nein | | □ | |
| Hämaptoe | □ ja | | □ nein | | □ | |
| Tachycardie | □ ja | | □ nein | | □ | |
| Bradycardie | □ ja | | □ nein | | □ | |
| Arrhythmie | □ ja | | □ nein | | □ | |
| Hypertonie | □ ja | | □ nein | | □ | |
| Nykturie | □ ja | | □ nein | | □ | |
| Ödeme | □ ja | | □ nein | | □ | |
| **Hautaffektion** | | | | | | |
| Juckreiz | □ ja | | □ nein | | □ | |
| Schwitzen | □ ja | | □ nein | | □ | |
| Ekzeme | □ ja | | □ nein | | □ | |
| Wunden | □ ja | | □ nein | | □ | |
| ▪ Lokalisation | ____ | | | | | |
| ▪ Größe | ____ | | | | | |
| ▪ Wundphase | ____ | | | | | |

| | | | | | Kommentar |
|---|---|---|---|---|---|
| Dekubitus ( nach Seiler ) | 0 | 1 | 2 | 3 4 | |
| ▪ Lokalisation | □ Ferse | | □ sacral | | |
| | □ Ellenbogen | | □ Sonstige:_______________ | | |
| ▪ Größe | ___________________ | | | | |
| ▪ Wundphase | ___________________ | | | | |
| Hautbild allgemein: | | | | | |
| □ ikterisch | □ Radiodermatitis | | | | |
| □ trocken | □ Sonstiges:______________________________ | | | | |
| Schleimhäute: | | | | | |
| □ Mund____________________ | □ Darmschleimhaut______________________ | | | | |
| □ Rachen______________________________ | | | | | |
| □ Sonstige______________________________ | | | | | |

| | | | Nicht beurteilbar | Kommentar |
|---|---|---|---|---|
| **Körpertemperatur** | | | | |
| Fieber | □ ja | □ nein | □ | |
| **Allgemeinbefinden** | | | | |
| Müdigkeit | □ ja | □ nein | □ | |
| Schlafstörung | □ ja | □ nein | □ | |
| Schwäche | □ ja | □ nein | □ | |
| **Hämatopoetisches System der Blutgefäße** | | | | |
| Manifeste Blutungen | □ ja | □ nein | □ | |
| Hämatome | □ ja | □ nein | □ | |
| Petechien | □ ja | □ nein | □ | |
| Blässe | □ ja | □ nein | □ | |

**Schmerzsymptome**

Visuelle Analogskala

| ☐ kein | ☐ leicht | | | ☐ mittel | | | | ☐ stark | | |
|---|---|---|---|---|---|---|---|---|---|---|
| 0 | 1 | 2 | 3 | 4 | 5 | 6 | 7 | 8 | 9 | 10 |

Lokalisation: ______________________________

Besonderheiten: ______________________________

| | | | | |
|---|---|---|---|---|
| **Probleme mit der Organisation der Versorgung** | □ ja | □ nein | □ | |
| **Überforderung der Familie/des Umfeldes** | □ ja | □ nein | □ | |
| **Sonstiges:** | □ ja | □ nein | □ | |

| 19. ECOG Leistungsstatus | | KARNOFSKY Index | |
|---|---|---|---|
| □ 0 | Normale, uneingeschränkte Aktivität, wie vor der Erkrankung | □100 | Normalzustand, keine Beschwerden keine manifeste Erkrankung |
| | | □ 90 | Minimale Krankheitssymptome |
| □ 1 | Einschränkung bei körperlicher Anstrengung, aber gehfähig; leichte körperliche Arbeit möglich | □ 80 | Normale Leistungsfähigkeit mit Anstrengung |
| | | □ 70 | Eingeschränkte Leistungsfähigkeit , arbeitsunfähig, kann sich alleine versorgen |
| □ 2 | Gehfähig, Selbstversorgung möglich, aber nicht arbeitsfähig; kann mehr als 50% der Wachzeit aufstehen | □ 60 | Gelegentliche fremde Hilfe |
| | | □ 50 | Krankenpflegerische und ärztliche Hilfe, nicht dauernd bettlägrig |
| □ 3 | Nur begrenzte Selbstversorgung möglich, 50% oder mehr der Wachzeit an Bett oder Stuhl gebunden | □ 40 | Bettlägrig, spezielle Pflege erforderlich |
| | | □ 30 | Schwer krank, Krankenhauspflege notwendig |
| □ 4 | Völlig pflegebedürftig, keinerlei Selbstversorgung möglich; völlig an Bett oder Stuhl gebunden | □ 20 | Krankenhauspflege und supportive Maßnahmen erforderlich |
| | | □ 10 | Moribund, Krankheit schreitet schnell fort |
| □ 5 | Tod | □ 0 | Tod |

**20. stationäre Behandlung notwendig:**

- ☐ Krankenhaus
- ☐ Stationäres Hospiz
- ☐ Heimunterbringung
- ☐ Sonstige:________________________________

**21. Gründe für die Verlegung:**

- ☐ Verschlechterung AZ
- ☐ Verschlechterung EZ
- ☐ unzureichende Schmerztherapie
- ☐ unzureichende Symptomkontrolle
- ☐ Sonstiges:____________________________________________________
- ☐ Palliative Chemotherapie
- ☐ Palliative Bestrahlung
- ☐ Geplanter Klinikaufenthalt
- ☐ geplante Kurzzeitmaßnahme

**22. Weitere Angaben zum Assessment**

Was ist der Grund des Kontaktes, was das Behandlungsziel? (Freitext)

Besonderer Aufwand mit (Freitext):

**23. Erhebung durchgeführt von:**

- ☐ SAPV-Arzt
- ☐ SAPV-Pflegekraft
- ☐ SAPV-Psychosozialer Mitarbeiter
- ☐ Name:_______________________________

Dauer des Assessments:_____________________________________________

Fahrzeit.______________ Anfahrtsweg einfach in km:________________

Zuständiger Koordinator:______________________________________

**24. Weitere Angaben zum Verlauf**

Welches Problem konnte besonders gut gelöst werden? (Freitext)

Anzahl der Teambesprechungen zur Problemlösung

________

Welches Problem konnte nicht ausreichend gelöst werden? (Freitext)

**Therapieende:**

- ☐ verstorben
- ☐ Verlegung
- ☐ Entlassung/Wechsel SAPV Leistungserbringer
- ☐ Sonstiges:________________________________

**Sterbeort**

- ☐ zu Hause
- ☐ Alten-Pflegeheim
- ☐ Stationäres Hospiz
- ☐ Palliativstation
- ☐ Krankenhaus
- ☐ Sonstige Heime/Einrichtungen
- ☐ unbekannt
- ☐ Sonstige:____________

*Anlage 9 (Muster)*

## Strukturerhebungsbogen

Ausgefüllt mit den entsprechenden Anlagen an die Krankenkasse senden.

An die zuständige Krankenkasse

Sollten sich bezüglich der nachfolgenden Angaben Änderungen ergeben, werden diese durch den SAPV-Leistungserbringer unverzüglich den beteiligten Vertragspartnern schriftlich mitgeteilt.

1. Angaben zum SAPV-Leistungserbringer

Name des SAPV-Leistungserbringers

Straße

Postfach

PLZ/Ort

Träger der Einrichtung

Rechtsform

- ❑ Einzelunternehmen
- ❑ GbR
  Auszug aus dem Gesellschaftsvertrag mit Angabe der Gesellschafter, Geschäftsführung und Unternehmenszweck in Kopie anliegend
- ❑ GmbH
  Auszug aus dem notariell beurkundeten Gesellschaftsvertrag mit Angabe der Gesellschafter, Geschäftsführung, Unternehmenszweck in Kopie anliegend
- ❑ eingetragener Verein (e.V.)

Auszug aus der Vereinssatzung in Kopie mit Angabe der Vorstandsmitglieder, Geschäftsführung, Vereinszweck in Kopie anliegend
beglaubigter Auszug aus dem Vereinsregister anliegend

❑ andere:
entsprechende Nachweise anliegend beigefügt.

___________________________
Gesellschafter

___________________________
Gesetzlicher Vertreter/Funktion

___________________________
Ansprechpartner für Krankenkassen

| | |
|---|---|
| Telefon | E-Mail |
| Telefax | Internet |
| Institutionskennzeichen | Betriebsstättennummer |

Betriebshaftpflicht
Abschluss einer ausreichenden Betriebshaftpflicht für Personen-, Sach- und Vermögensschäden, die regelmäßig an die aktuelle Betriebsgröße angepasst wird.

❑ Antrag in Kopie anliegend ❑ Versicherungsschein in Kopie anliegend

Abgeschlossen am:______________________________
Vers.-Nr.:______________________________________
bei:___________________________________________

2. Qualifizierte Palliativ-Ärzte/qualifizierte Pflegefachkräfte/weitere qualifizierte Fachkräfte (Qualifikationsnachweise gemäß des Vertrages sind in der Anlage beizufügen)

2.1 Bitte die Namen der qualifizierten Palliativ-Ärzte angeben

______________________________________________
______________________________________________
______________________________________________
______________________________________________
______________________________________________

❑ jeweils Nachweis der Anmeldung zur Sozialversicherung
❑ jeweils Fotokopien zur Führung der Berufsbezeichnung
❑ jeweils Fotokopien zum Nachweis der Weiterbildung Palliativmedizin im Umfang von 160 Stunden
❑ jeweils Fotokopien zum Nachweis der Berufspraxis und ggf. weiterer Fort- und Weiterbildungsbescheinigungen
❑ jeweils Bestätigung des Trägers, aus der hervorgeht, dass mit dem Träger ein Arbeitsvertrag abgeschlossen wurde und der Einsatz beim Leistungserbringer bestätigt wird. (Fotokopie der Arbeitsverträge beilegen)
❑ jeweils Auszüge vom Bundesamt für Justiz – polizeiliches Führungszeugnis (nicht älter als drei Monate) beifügen.

2.2 Bitte die Namen und Qualifikation der qualifizierten Pflegefachkräfte angeben:

____________________
____________________
____________________
____________________
____________________
____________________
____________________
____________________
____________________
____________________

❑ jeweils Nachweis der Anmeldung zur Sozialversicherung
❑ jeweils Fotokopien zur Führung der Berufsbezeichnung
❑ jeweils Fotokopien zum Nachweis der Palliative-Care-Weiterbildung im Umfang von 160 Stunden
❑ jeweils Fotokopien zum Nachweis der Berufspraxis und ggf. weiterer Fort- und Weiterbildungsbescheinigungen
❑ jeweils Bestätigung des Trägers, aus der hervorgeht, dass mit dem Träger ein Arbeitsvertrag abgeschlossen wurde und der Einsatz beim Leistungserbringer bestätigt wird. (Fotokopie der Arbeitsverträge beilegen)
❑ jeweils Auszüge vom Bundesamt für Justiz – polizeiliches Führungszeugnis (nicht älter als drei Monate) beifügen.

2.3 Bitte den Namen und Qualifikation der weiteren qualifizierten Fachkräfte angeben:

____________________
____________________
____________________
____________________

❑jeweils Nachweis der Anmeldung zur Sozialversicherung
❑ jeweils Fotokopien zur Führung der Berufsbezeichnung
❑ jeweils Fotokopien zum Nachweis der Palliative-Care-Weiterbildung im Umfang von 160 Stunden
❑ jeweils Fotokopien zum Nachweis der Berufspraxis und ggf. weiterer Fort- und Weiterbildungsbescheinigungen

❑ jeweils Bestätigung des Trägers, aus der hervorgeht, dass mit dem Träger ein Arbeitsvertrag abgeschlossen wurde und der Einsatz beim Leistungserbringer bestätigt wird. (Fotokopie der Arbeitsverträge beilegen)
❑ jeweils Auszüge vom Bundesamt für Justiz – polizeiliches Führungszeugnis (nicht älter als drei Monate) beifügen.

2.4 Handzeichenliste
Die Handzeichenliste über das nach diesem Vertrag eingesetzte Personal mit Namen, Berufbezeichnung/Zusatzqualifikation, wöchentlicher Arbeitszeit und Handzeichen ist ausgefüllt für jeden Mitarbeiter beizufügen.

2.5 Personelle Zuständigkeit und Verantwortlichkeit im SAPV-Team (u.a. Weisungsgebundenheit, Weisungsbefugnisse), Organigramm beifügen

3. Versorgungskonzept

Das verbindliche, strukturierte und schriftlich beigefügte Versorgungskonzept des SAPV-Teams enthält mindestens folgende Punkte:

3.1 Standort des SAPV-Leistungserbringers,

Der SAPV-Leistungserbringer muss bestätigen, dass er über eine eigenständige Adresse und geeignete Räumlichkeiten verfügt

❑ zur Beratung von Patienten und Angehörigen

❑ für Teamsitzungen und Besprechungen

❑ die Lagerhaltung von eigenen Medikamenten für Notfall-/Krisenintervention und Hilfsmitteln
❑ für die Aufbewahrung von Medikamenten, die unter das Betäubungsmittelgesetz fallen, ist ein BtM-Schrank (§15 BtM-G) erforderlich
Ggf. eine Kopie der Baupläne mit der Ausweisung der o.g. Erfordernisse dem Konzept beifügen.

3.2 Versorgungsgebiet, bitte mit PLZ und Orten ausweisen (ggf. Karte beifügen)

3.3 Versorgungsprozess, Darstellung wer wird wann, wie, vom wem in den Versorgungsprozess integriert

Wer trägt die Prozessverantwortung in der Zusammenarbeit der Kooperationspartner untereinander, interne/externe organisatorische Abläufe der SAPV müssen transparent dargestellt werden

3.4 Kooperationspartner, beiliegende Liste ausgefüllt übersenden
Kooperationsvereinbarungen mit im Rahmen des Netzwerkes (vgl. § 1 Abs. 3 und § 3 Abs. 3) mit an der Versorgung Beteiligten im Versorgungsgebiet (z. B. ambulante Hospizdienste, Seelsorge, u.s.w.) zur Sicherung der psychosozialen Unterstützung sind beizufügen.

3.5 Mitarbeiterkapazität (Stellenplan) ist auf der beigefügten Handzeichenliste auszuweisen

3.6 sächliche Ausstattung

- eine geeignete, aktuell geführte und für die an der Versorgung Beteiligten jederzeit zugängliche Patientendokumentation (Muster beifügen)
- Arzneimittel (inkl. BtM) für die Notfall-/Krisenintervention werden in ausreichender Menge vorgehalten (Arzneimittelliste und Liste der Bevorratungsmenge beilegen)
- Arzt-/Pflegekoffer/Bereitschaftstasche (Berücksichtigung der Kompatibilität der Verbrauchsmaterialien zu Medizinprodukten unterschiedlicher Hersteller, z.B. bei Portsystemen oder Infusionspumpen) werden vorgehalten (Liste mit Anzahl der Arzt-/Pflegekoffer/Bereitschaftstasche, der vorgehaltenen Verbrauchsmaterialien beifügen)
- eine geeignete administrative Infrastruktur, z. B. Büro, Kommunikationstechnik (vorhandene Struktur beschreiben, Anzahl Telefone, Handy, Anrufbeantworter, EDV Ausstattung etc.)

3.7 Qualitätssicherungsmaßnahmen gemäß § 11, zusätzlich Ausführungen welche Qualitätsmanagementtools in Ihrem Konzept zum Einsatz kommen (wie Leitlinien/Standards, allgemeine und/oder medizinische/pflegerische Qualitätsindikatoren, Qualitätszirkel, Fallkonferenzen, Fort- und Weiterbildung der Leistungserbringer etc.)?

3.8 Ein Konzept, das darstellt wie eine Sicherstellung der reibungslosen Versorgung mit Arznei-, Heil- und Hilfsmitteln gewährleistet wird, ist beizufügen.

3.9 Ein Konzept, das die Sicherstellung der psychosozialen und seelischen Unterstützung darstellt ist beizulegen.

3.10 Konzept zur Sicherstellung der 24-Stunden-Rufbereitschaft, schriftliche Darstellung wie und von wem diese sichergestellt wird, ist beizufügen.

Ort, Datum, Stempel/Unterschrift des SAPV-Leistungserbringers

Saxony-Anhalt

# Rahmenvertrag

zur Erbringung und Abrechnung von SAPV-Leistungen
von Vertragsärzten in Sachsen-Anhalt

zwischen den

Palliativzentren

| | | |
|---|---|---|
| Städtisches Klinikum Dessau<br>Auenweg 38,<br>06847 Dessau-Roßlau | Johanniter Zentrum für Medizinische Versorgung in der Altmark GmbH,<br>Wendstraße 31,<br>39576 Stendal | Medizinisches Zentrum Harz GmbH<br>Ilsenburger Straße 15<br>38855 Wernigerode |

(nachfolgend PZ)

und der

KV-Mangement-Gesellschaft mbH
vertreten durch die Geschäftsführerin,
Frau Gabriele Wenzel,
Doctor-Eisenbart-Ring 2,
39120 Magdeburg

(nachfolgend KV-M-GmbH)

auf der Grundlage des IV-Vertrages zur SAPV gemäß §§ 132 d Absatz 1, 37 b SGB V der AOK Sachsen-Anhalt, der Knappschaft, der Landwirtschaftlichen Krankenkasse und der IKK gesund plus mit oben angegebenem PZ.

## Präambel

Mit diesem Rahmenvertrag gewährleistet die KV-M-GmbH dem PZ die ordnungsgemäße und zeitnahe patientenbezogene Abrechnung von spezialisierter ambulanter Palliativversorgung (SAPV-Leistungen), die durch Vertragsärzte ausgehend von einer Verordnung des Musters 63 auf Grundlage des jeweilig abgestimmten Behandlungsplanes mit den Palliativ-Care-Teams (im weiteren PCT) erbracht werden.

In diesem Rahmenvertrag sind mögliche Leistungsinhalte der Vertragsärzte bezogen auf die SAPV-Versorgung auf Grundlage des entsprechendes IV-Vertrages mit der AOK Sachsen-Anhalt, der Knappschaft, der Landwirtschaftlichen Krankenkasse und der IKK gesund plus einheitlich und abschließend definiert sowie mit den dazugehörigen einheitlichen Vergütungssätzen mit Geltung für Sachsen-Anhalt hinterlegt.

Ausschließlicher Ansprechpartner des PZ betreffend die ordnungsgemäße Leistungserbringung der SAPV-Leistungen durch Vertragsärzte auf der Grundlage dieses Rahmenvertrages sowie deren korrekte und ordnungsgemäße Abrechung ist die KV-M-GmbH, die ihre Zahlungen nach Rechnungslegung gegenüber dem PZ von diesem mit befreiender Wirkung erhält.

Die KV-M-GmbH informiert die Vertragsärzte über die Geltung dieses Rahmenvertrages im Einklang mit den Konditionen des zugrunde liegenden IV-Vertrages mit den oben angegebenen Krankenkassen unter Berücksichtigung der Richtlinie des gemeinsamen Bundesausschusses zur Verordnung von spezialisierter ambulanter Palliativversorgung sowie der gemeinsamen Empfehlungen nach § 132 d Absatz 2 SGB V.

Zentrales Ziel dieser Rahmenvereinbarung ist eine zeitnahe, möglichst reibungslose Umsetzung des gesetzlichen Auftrages zur SAPV im Flächenland Sachsen-Anhalt, die nur durch die Einbeziehung von Vertragsärzten im Rahmen einer additiven Teilversorgung auf der Grundlage klarer und transparenter Rahmenbedingungen auf möglichst unbürokratischem Weg, aber mit einem hohen Verbindlichkeitsgrad, zu realisieren ist.

## § 1
## Aufgabenverteilung und Verfahrensablauf innerhalb der IV-SAPV

**(1)** Die SAPV-Versorgung wird ausgelöst mit Verordnung des Musters 63 durch den Hausarzt/Facharzt (im weiteren einheitlich Vertragsarzt). Zwischen Vertragsarzt und dem PZ, dem vertraglich der gesetzliche SAPV-Sicherstellungsauftrag der in der Präambel aufgeführten Krankenkassen gemäß §§ 132 d Absatz 1, 37 b SGB V obliegt, ist eine Aufgabenverteilung sowie ein Verfahrensablauf gemäß der Anlage 1 zu diesem Rahmenvertrag vereinbart.

**(2)** Ohne zeitliche Verzögerung ausgehend von der Verordnung des Musters 63 vereinbaren der verordnende Vertragsarzt und das PZ ein sogenanntes Überleitungskonsil in der Häuslichkeit des Patienten, in Pflegeheimen ggf. auch vorab am Krankenhausbett (im weiteren häusliches Überleitungskonsil), in dem ein bindender, patientenbezogener SAPV-Behandlungsplan mit nachfolgenden Inhalten vereinbart wird:

- Festlegung der individuellen SAPV-Versorgungsinhalte im Sinne des betroffenen Patienten
- Austausch der notwendigen patientenbezogenen Befunderhebungen und Diagnostik auch bereichsübergreifend, wie im Rahmen der Regelversorgung indiziert
- Abstimmung des besonderen Betreuungs-/Koordinationsaufwandes unter Berücksichtigung des gesetzlichen SAPV-Versorgungsauftrages unter Einbeziehung aller nichtärztlichen Leistungserbringer (Pflegedienste usw.)
- Absicherung der Medikation und notwendiger weiterer Verordnungen (Heil- und Hilfsmittel, Schmerztherapeutika, Betäubungsmittel usw.) unter Absicherung des Versorgungsweges für den Patienten
- Organisation und Absicherung der ggf. notwendigen SAPV-Hausbesuche durch den Vertragsarzt, abgestimmt mit dem PCT im Zuständigkeitskreis des PZ
- Abstimmung und Organisation einer SAPV-Notfallversorgung durch den Vertragsarzt (24-Stunden-Rufbereitschaft)
- Informationsaustausch/Dokumentation des SAPV-Behandlungsverlaufs sowie der Vertragsarztkontakte in der Patientenhäuslichkeit

Zuständig für die Organisation der im häuslichen Überleitungskonsil konkretisierten SAPV-Versorgungsinhalte ist das PZ bzw. das bei diesem angesiedelte PCT.

**(3)** Der Behandlungsplan nach Absatz 2 hat Festlegungen zwischen Vertragsarzt und PCT auf der Grundlage der Anlage 2, bezogen auf die Notwendigkeit von SAPV-Hausbesuchen und/oder einer SAPV-Notfallversorgung durch den Vertragsarzt zu beinhalten. Diese Festlegungen werden auf der sogenannten Frequenz-Übersicht, wie als Muster und Kopiervorlage für das PZ als Anlage 5 aufgeführt ist, fortlaufend seitens des Vertragsarztes mit Datumsangabe unter Unterschriftsleistung dokumentiert. Diese Anlage 5 dient neben der damit verbundenen individuellen SAPV-Aufgabenverteilung auch der Absicherung einer korrekten und ordnungsgemäßen Abrechnung der erbrachten Leistungen durch Vertragsärzte bezogen auf die SAPV durch die KV-M-GmbH.

**(4)** Alle seitens der Vertragsärzte erbrachten Leistungen, beruhend auf dem abgestimmten individuellen Behandlungsplan nach Absatz 2, werden gegenüber dem PZ durch die KV-M-GmbH abgerechnet. Eine Rechnungslegung der Vertragsärzte für Leistungen der SAPV unmittelbar gegenüber dem PZ bzw. den die IV-SAPV abschließenden Krankenkassen ist ausgeschlossen.

## § 2
## Zuständigkeitsbereiche des PZ der AOK Sachsen-Anhalt, der Knappschaft, der Landwirtschaftlichen Krankenkasse und der IKK gesund plus

**(1)** Die Zuständigkeit des PZ nach diesem Rahmenvertrag ergibt sich aus Anlage 3 mit den darin aufgeführten Ansprechpartnern, denen die Verordnung gemäß Muster 63 unverzüglich zu übermitteln ist. Bei Unvollständigkeiten bzw. Änderungen der Kontaktdaten der Ansprechpartner sind diese unverzüglich der KV-M-GmbH mitzuteilen.

**(2)** Das PZ/PCT ist unmittelbarer Ansprechpartner für die SAPV-Versorgung, ausgehend von den die Verordnungen nach Muster 63 ausstellenden Vertragsärzten.

## § 3
## Feststellung des Bedarfs zur spezialisierten ambulanten Palliativversorgung

**(1)** Auf der Grundlage der Richtlinien des Gemeinsamen Bundesausschusses zur SAPV besteht ein Bedarf nach SAPV-Versorgung, soweit anderweitige ambulante Versorgungsformen nicht mehr ausreichen. Anhaltspunkt dafür ist das Vorliegen eines komplexen Symptomgeschehens, dessen Behandlung spezifische palliativmedizinische und/oder palliativpflegerische Kenntnisse und Erfahrungen sowie ein interdisziplinär, insbesondere zwischen Ärzten und Pflegekräften in besonderem Maße abgestimmtes Konzept voraussetzt.

**(2)** Für diese Feststellung wird eine Klassifikation im Kanrofsky-Index (siehe Anlage 4) von unter 40 % für das Vorliegen eines komplexen Symptomgeschehens zugrunde gelegt. Nach diesem Index müssen mindestens zwei Symptome der in Anlage 4 beigefügten Übersicht vorliegen. Nur Patienten, die über diese komplexe Ausprägung des Symptomgeschehens verfügen, haben einen Anspruch auf SAPV-Versorgung.

## § 4
## Vergütung und Rechnungslegung durch die KV-M-GmbH gegenüber dem PZ und dem Vertragsarzt

**(1)** Die von Vertragsärzten gemäß diesem Rahmenvertrag erbrachten SAPV-Leistungen werden nach dem einheitlichen Gebührenordnungskatalog der Anlage 2 von der KV-M-GmbH mit dem PZ monatlich auf Grundlage der Frequenz-Übersicht nach Anlage 5 abgerechnet.

**(2)** Die Anlage 5 wird von dem PZ, der KV-M-GmbH wie auch dem leistungserbringenden Vertragsarzt monatlich, spätestens am siebten Werktag nach Monatsende, ggf. auch früher, im Falle des Versterbens des SAPV-Patienten, in Kopie (ggf. maschinell lesbar) zur Verfügung gestellt. Die Originale der Anlage 5 bleiben beim PZ.

**(3)** Vor Übermittlung der Anlage 5 prüft das PZ die Frequenz-Übersicht auf sachlich-rechnerische Richtigkeit. Bei ggf. bestehenden Implausibilitäten klärt das PZ diese mit dem Vertragsarzt, kennzeichnet diese abgestimmten Korrekturen auf der Anlage 5 und nutzt zur Erläuterung das dafür vorgesehene Begründungsfeld. Sofern eine einvernehmliche Klärung durch das PZ mit dem Vertragsarzt nicht möglich sein sollte, übernimmt die KV-M-GmbH die Bearbeitung und Klärung auf der Grundlage der in diesem Sinne angeführten Erläuterungen des PZ im Begründungsfeld der übermittelten Anlage 5.

**(4)** Der Vertragsarzt erhält auf der Grundlage der übermittelten Frequenz-Übersicht durch das PZ die Möglichkeit, in seiner Abrechnung gegenüber der KVSA auf dem entsprechenden vertragsärztlichen Abrechnungsschein des Patienten die SAPV-Pseudoziffern der Anlage 2 einzutragen. Der Vertragsarzt erhält die erbrachten SAPV-Leistungen, wie auf Anlage 5 dokumentiert und in seiner Abrechnung über die entsprechenden Pseudoziffern der Anlage 2 gekennzeichnet, im üblichen Bearbeitungsrhythmus der vertragsärztlichen Abrechnung durch die KVSA mit der Restzahlung unter Abzug des aktuellen Verwaltungskostensatzes, wie er auch für die KVSA Geltung hat, vergütet. Die SAPV-Leistungen werden in der Quartalsabrechnung und auf dem Kontoauszug des Vertragsarztes, die er von der KVSA erhält, nachvollziehbar ausgewiesen.

**(5)** Die KV-M-GmbH nutzt die Anlage 5 zur Rechnungslegung der SAPV-Leistungserbringung, wie sie vom Vertragsarzt erbracht wurde, gegenüber dem PZ. Mit individueller patientenbezogener Rechnungslegung durch die KV-M-GmbH erfolgt eine Zahlung des PZ mit befreiender Wirkung zu Gunsten der leistungserbringenden Vertragsärzte gegenüber der KV-M-GmbH binnen 28 Tagen nach Rechnungseingang.

## § 5
## Information und Gewährleistung der rechtlichen und vertraglichen IV-SAPV Versorgungsinhalte

**(1)** Die KV-M-GmbH gewährleistet dem PZ die ordnungsgemäße Leistungserbringung der Vertragsärzte, die gemeinsam mit dem PZ/PCT eine vertraglich konkretisierte SAPV-Versorgung im Flächenland Sachsen-Anhalt realisieren. Gegebenfalls auftretende organisatorische, fachliche wie auch abrechnungstechnische Probleme, die nicht einvernehmlich zwischen dem PZ und den Vertragsärzten klärbar waren, werden von der KV-M-GmbH gelöst. Die KV-M-GmbH gewährleistet die ordnungsgemäße Abwicklung auf Grundlage dieses Rahmenvertrages.

**(2)** Die KV-M-GmbH informiert die Vertragsärzte in Sachsen-Anhalt über diesen Rahmenvertrag, seine Voraussetzungen und Konditionen.

## § 6
## Datenschutz, ärztliche Schweigepflicht

Die Vertragspartner verpflichten sich bei Erhebung, Verarbeitung, Speicherung und Weitergabe personenbezogener Daten, die datenschutzrechtliche Schweigepflicht zu beachten.

## § 7
## Inkrafttreten, Laufzeit, Kündigung

**(1)** Der Vertrag tritt zum 1. März 2010 in Kraft. Er kann von den Vertragspartnern halbjährlich in Schriftform mit einer Frist von 3 Monaten zum Ende des Kalenderhalbjahres, erstmalig zum 30. September 2011 mit Wirkung zum 31. Dezember 2011, gekündigt werden.

**(2)** Im Falle der Kündigung können SAPV-Leistungen des Vertragsarztes auf der Grundlage eines abgestimmten Behandlungsplanes bis zum letzten Tag der Laufzeit des Rahmenvertrages erbracht werden. Diese Leistungen werden maximal bis zum letzten Tag seitens des PZ auf der Grundlage dieses Rahmenvertrages vergütet.

**(3)** Eine Kündigung aus wichtigem Grund ist stets zulässig. Um einen wichtigen Grund handelt es sich, wenn eine der Vertragsparteien schwerwiegend gegen die obliegenden Verpflichtungen aus diesem Rahmenvertrag verstößt, so dass die erforderliche Vertrauensgrundlage unwiderbringbar zerrüttet ist.

## § 8
## Salvatorische Klausel

Sollten eine oder mehrere Bestimmungen dieses Vertrages unwirksam sein oder werden, bleibt die Wirksamkeit des übrigen Vertrages hiervon unberührt, es sei denn, dass die unwirksame Bestimmung für eine Vertragspartei derart wesentlich war, dass ihr ein Festhalten an diesem Vertrag nicht zugemutet werden kann. In allen anderen Fällen werden die Vertragspartner die unwirksamen Bestimmungen durch Regelungen ersetzen, die dem mit der unwirksamen Bestimmung Gewollten am nächsten kommen.

| | |
|---|---|
| Datum: | Datum: |
| ______________________________ | ______________________________ |
| KV-Management-Gesellschaft mbH | Medizinisches Zentrum Harz GmbH<br>Wernigerode |

**Anlage 1**

# Verfahrensablauf - von der Verordnung der SAPV zur Realisierung des individuellen Versorgungsplanes

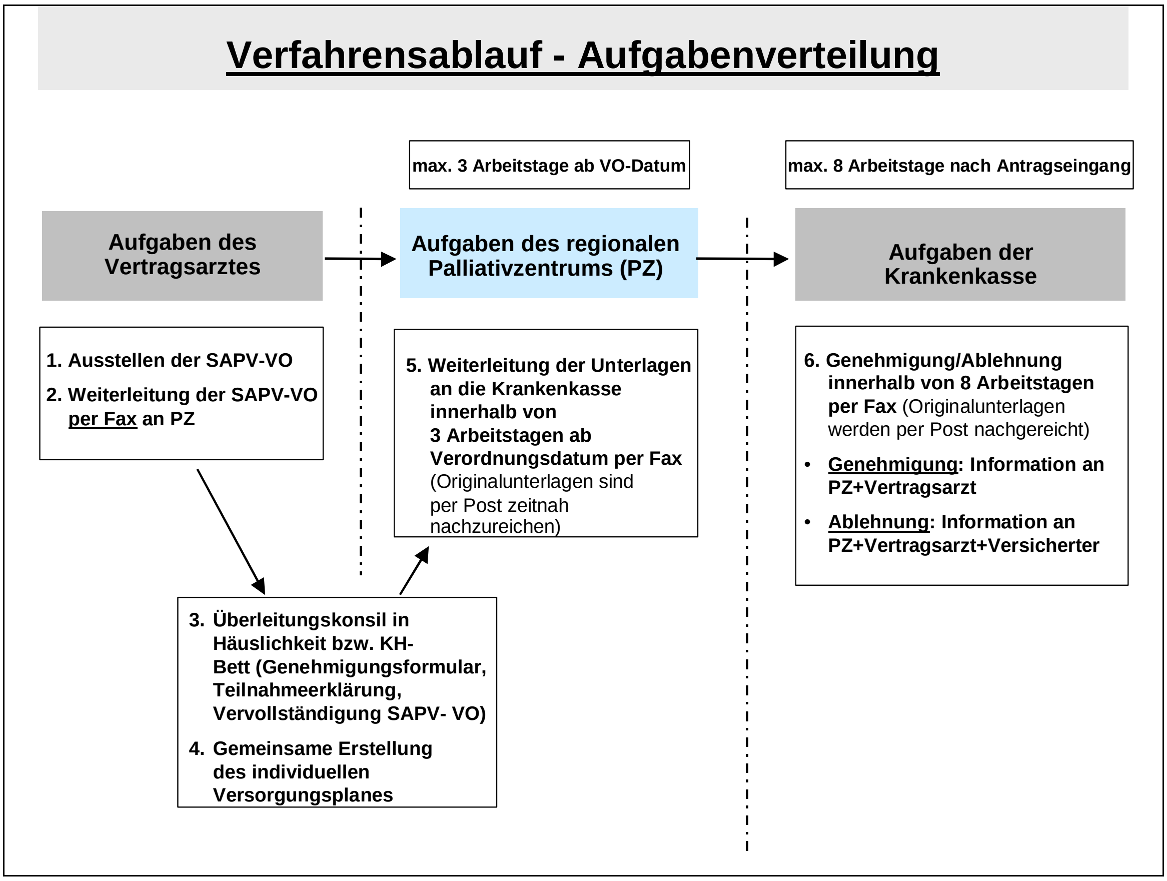

## 2.1 Vergütung der additiven Teilversorgung

| | Leistungsart | Leistungsinhalt | Häufigkeit | Abrechnungs-voraussetzung | Pseudo-Ziffer | SAPV-Zuschlag |
|---|---|---|---|---|---|---|
| 1. | **Überleitungskonsil** in der Häuslichkeit des Patienten (ggf. auch am Krankenhausbett, stationärem Hospiz), gemeinsam mit dem Palliativ-Care-Team und den Angehörigen unter Abstimmung des individuellen Versorgungsplanes mit Verbindlichkeit für alle ärztlichen und nichtärztlichen Leistungserbringer | - Abstimmung und Festlegung der individuellen SAPV-Versorgungs-inhalte und des Betreuungs-aufwandes mit allen Leistungs-erbringern<br>- Dokumentation<br>- besondere Praxisorganisation<br>- Absicherung der Medikation und notwendiger weiterer Verordnungen<br>- Organisation einer Rund-um-die-Uhr hausärztlichen / fachärztlichen Rufbereitschaft<br>- Austausch der patientenbezogenen Befunderhebungen und Diagnostik auch bereichsübergreifend | einmalig pro Patient | EBM: Hausbesuch / Wegepauschale plus Zuschlag SAPV | **91030** | **67,50 €** |
| 2. | **SAPV-Hausbesuch** abgestimmt mit PZ | - notwendige hausärztliche / fachärztliche Symptomkontrolle<br>- Anpassung / Prüfung der Medikation der Verordnungen etc. | jeweils | EBM: Hausbesuch / Wegepauschale plus SAPV-Zuschlag | **91031** | **25,00 €** |
| 3. | **SAPV-Notfall** außerhalb des organisierten Bereitschaftsdienstes | - Absicherung / Abstimmung und Organisation einer individuellen SAPV-Rufbereitschaft durch den Vertragsarzt | jeweils | EBM: Hausbesuch / Wegepauschale plus SAPV-Zuschlag | **91032** | **25,00 €** |

**Anmerkung: binnen 3 Tagen muss Verordnungsformular zur Genehmigung bei der Krankenkasse vorliegen; demnach binnen 8 Tagen wird Genehmigung ausgesprochen; d.h. max. 11 Tage „Vorfinanzierung“ des PZs zu o.a. Konditionen auch außerhalb einer Gewährung über Tagespauschale möglich**

**Anlage 2**

## 2.2 SAPV-Versorgung durch qualifizierte Haus- und Fachärzte als verantwortliche Ärzte des Palliativ-Care-Teams des PZ’s (Dienstleistung) bezogen auf den einzelnen SAPV-Patienten

| | Art | Leistungsinhalte des vertragsärztlichen Dienstleisters für das PZ | Qualifikation | Häufigkeit | Pseudoziffer | SAPV-Zuschläge |
|---|---|---|---|---|---|---|
| 1. | **Überleitungskonsil** in der Häuslichkeit des Patienten gemeinsam mit dem den Patienten in der GKV behandelnden (Hausarzt/Facharzt) als ärztlich verantwortliches Mitglied des P-C-T des PZ | • Abstimmung der individuellen SAPV-Versorgungsinhalte mit dem Hausarzt/Facharzt sowie verantwortliche Versorgungsplanerstellung für alle Leistungserbringer der SAPV für den genehmigten Zeitraum | Zusatzbezeichnung PA<br>Basiskurs PA<br>internes Curriculum* | **Einmalig, in der ersten Woche** (patientenbezogen) | 91035 A<br>91035 B<br>91035 C | 100,00 €<br>90,00 €<br>60,00 € |
| 2 | **Koordinierungs- und Konsiliarpauschale** | • verantwortliche Koordination des individuellen SAPV-Versorgungsplanes, Prüfung auf Aktualität der med. und pflegerischen Festlegungen in der SAPV zwischen jeweiligen Hausarzt/Facharzt und PZ sowie allen SAPV-Leistungserbringern;<br>• ggf. auch umfassend weiteres Konsil in der Häuslichkeit mit dem jeweiligen Hausarzt/ Facharzt/ SAPV-Leistungserbringer | Zusatzbezeichnung PA<br>Basiskurs PA<br>internes Curriculum* | **Kalenderwochen-pauschale/ab der zweiten Woche** (patientenbezogen) | 91036 A<br>91036 B<br>91036 C | 150,00 €<br>120,00 €<br>100,00 € |
| 3. | **ggf. SAPV-Notfall** der nur durch den Vertragsarzt als PZ-Palliativ-Arzt des P-C-T und nicht durch Hausarzt/Facharzt des SAPV-Patienten erbracht werden kann oder ggf. **SAPV-Hausbesuch** durch den Vertragsarzt als PZ-Palliativ-Arzt des P-C-T, der nicht durch Hausarzt/Facharzt erbracht werden kann | • Vor-Ort-SAPV-Realisierung einer Bereitschaft durch spezialisierten Palliativarzt des PZ aufgrund<br>• notwendiger spezialisierter SAPV-Systemkontrollen oder<br>• Anpassung/Prüfung der Medikation der Verordnung etc. nur möglich durch PZ-Palliativmediziner | Zusatzbezeichnung PA<br>Basiskurs PA<br>internes Curriculum* | **jeweils bei Sicherstellungs-bedarf** (patientenbezogen) | 91037 A<br>91037 B<br>91037 C | 70,00 €<br>60.00 €<br>50,00 € |
| 4. | **Rufbereitschaft für das PZ** | • Absicherung der 24 Std.-Rufbereitschaft für das PZ/ständiges telefonisches Konsil gegenüber Hausarzt/Facharzt des SAPV-Patienten und ggf. den Angehörigen | Zusatzbezeichnung PA<br>Basiskurs PA<br>internes Curriculum* | **Kalenderwochen-pauschale** (patientenunabhängig ab einem eingeschriebenen SAPV-Patienten) | 91038 A<br>91038 B<br>91038 C | 200,00 €<br>150,00 €<br>120,00 € |

* internes Curriculum: Die Vertragspartner beabsichtigen die Vergabe eines internen Fortbildungscurriculums SAPV, welches von den Inhalten und auch von der Organisation her von diesem Vertragspartner gesteuert wird.

**Anlage 3**

## Zuständigkeitsbereiche und Ansprechpartner

| Region Dessau-Roßlau, Anhalt-Bitterfeld, Wittenberg | Region Altmarkkreis Salzwedel, Stendal, Jerichower Land | Region Harz, Mansfeld Südharz |
|---|---|---|
| Medizinisches Versorgungszentrum des Städtischen Klinikums Dessau gGmbH<br>Spezialisierte Ambulante Palliativversorgung<br>Herr Petrowsky<br>Auenweg 38<br>06847 Dessau-Roßlau<br><br>Telefon: 0174/ 938 1471<br>Fax: 0340/ 501 12 53<br>Email: r.petrowsky@mvzdessau.de<br><br>**Versorgungsbeginn:**<br>Dessau-Roßlau: 1. April 2009<br>Anhalt-Bitterfeld: unbekannt<br>Wittenberg: unbekannt | Johanniter Zentren für medizinische Versorgung in der Altmark GmbH:<br>Frau Ines Pericie<br>Wendstraße 31<br>39576 Stendal<br>Tel. 03931 218381<br>FAX: 03931 661252<br>Email: ines.pericie@jgsb.johanniter.de<br><br>oder<br><br>Frau Dr. Ulrike Mohren<br>(von 8.00 Uhr bis 12.00 Uhr)<br>Johanniter-Krankenhaus Genthin-Stendal gGmbH<br>Wendstraße 31<br>39576 Stendal<br>Tel. 03931 661255 oder 661251<br>Fax: 03931 661252<br>Email: ulrike.mohren@jksdl.de | Medizinisches Zentrum Harz GmbH<br>Palliativzentrum<br>Frau Simone Franz<br>Ilsenburger Straße 15<br>38855 Wernigerode<br>Tel.: 03943 614590<br>FAX: 03943 614591<br>Email: simone.franz@harz-klinikum.de<br>sapv@mvz-harz.de |

**Anlage 4**

# Klassifikation im Karnofsky Index

| **Symptomatik** | **Ausprägung** | | |
|---|---|---|---|
| *respiratorische/kardiale Symptomatik* | **leicht** | **mittel** | **schwer** |
| Dyspnoe | ☐ | ☐ | ☐ |
| Hämoptoe (Bluthusten) | ☐ | ☐ | ☐ |
| NYHA (Stufe II-IV) | ☐ | ☐ | ☐ |
| sonstiges ................................... | ☐ | ☐ | ☐ |
| *neurologische/psychiatrische/psychische Symptomatik* | | | |
| Quantitative Bewusstseinsstörungen | ☐ | ☐ | ☐ |
| Hirndrucksymptome | ☐ | ☐ | ☐ |
| Spastik | ☐ | ☐ | ☐ |
| Myoklonus | ☐ | ☐ | ☐ |
| Muskelkämpfe | ☐ | ☐ | ☐ |
| Depression | ☐ | ☐ | ☐ |
| Psychotische Syndrome | ☐ | ☐ | ☐ |
| sonstiges ....................................... | ☐ | ☐ | ☐ |
| *ulzerierende/exulzerierende Wunden oder Tumore* | | | |
| Lokalisation…………………………… | ☐ | ☐ | ☐ |
| Lokalisation……………………...…… | ☐ | ☐ | ☐ |
| Lokalisation…………………………… | ☐ | ☐ | ☐ |
| *gastrointestinale Symptomatik* | | | |
| Anorexie/Kachexie | ☐ | ☐ | ☐ |
| Mukositis | ☐ | ☐ | ☐ |
| Dysphagie | ☐ | ☐ | ☐ |
| Erbrechen | ☐ | ☐ | ☐ |
| Hämatemesis | ☐ | ☐ | ☐ |
| Ikterus | ☐ | ☐ | ☐ |
| Ileus | ☐ | ☐ | ☐ |
| Aszites | ☐ | ☐ | ☐ |
| Diarrhoe | ☐ | ☐ | ☐ |
| Fisteln | ☐ | ☐ | ☐ |
| sonstiges ........................................ | ☐ | ☐ | ☐ |
| *urogenitale Symptomatik* | | | |
| Harnwegsinfekt | ☐ | ☐ | ☐ |
| Dysurie | ☐ | ☐ | ☐ |
| Blasentenesmen | ☐ | ☐ | ☐ |
| Hämaturie | ☐ | ☐ | ☐ |
| Vaginale Blutung und Ausfluss | ☐ | ☐ | ☐ |
| sonstiges ........................................ | ☐ | ☐ | ☐ |

Saxony/Thuringia

**Vertrag gemäß § 132 d SGB V**

**über die spezialisierte ambulante Palliativversorgung**

Zwischen

**Ärzten und Vertretern der Pflege nach § 1 dieses Vertrages**
**oder**
**juristische Person: Musterverein, GmbH etc.**

**mit Sitz**

**Adresse**

- nachfolgend SAPV-Leistungserbringer genannt -

und

folgenden Krankenkassen

MUSTER

**AOK PLUS - Die Gesundheitskasse für Sachsen und Thüringen.**
Augustinerstraße 38 in 99084 Erfurt
zugleich handelnd für die Landwirtschaftliche Krankenkasse Mittel- und Ostdeutschland sowie die Krankenkasse für den Gartenbau

**BKK-Landesverband Ost, Landesrepräsentanz Sachsen**
Tiergartenstraße 32 in 01219 Dresden

**IKK Sachsen**
Arndtstraße 13 in 01099 Dresden

**Knappschaft, Regionaldirektion Chemnitz**
Jagdschänkenstraße 50 in 09117 Chemnitz

Barmer Ersatzkasse
Techniker Krankenkasse (TK)
Deutsche Angestellten-Krankenkasse (Ersatzkasse)
KKH – Allianz (Ersatzkasse)
Gmünder ErsatzKasse (GEK)
HEK - Hanseatische Krankenkasse
Hamburg Münchener Krankenkasse
hkk

Gemeinsamer Bevollmächtigter mit Abschlussbefugnis:
**Verband der Ersatzkassen e. V. (vdek),**
**vertreten durch die Leiterin der Landesvertretung Sachsen**
Glacisstraße 4 in 01099 Dresden

- nachfolgend Krankenkassen genannt -

**wird zur Durchführung spezialisierter ambulanter Palliativversorgung**

**Folgendes vereinbart:**

# Inhaltsverzeichnis


| | | Seite: |
|---|---|---|
| Präambel | | 3 |
| § 1 | Vertragspartner | 3 |
| § 2 | Ziele des Vertrages | 4 |
| § 3 | Versorgungsgebiet | 5 |
| § 4 | Inhalt und Umfang der Leistungen | 5 |
| § 5 | Anspruchsberechtigte | 6 |
| § 6 | Kostenübernahme | 6 |
| § 7 | Grundsätze für die Leistungserbringung | 8 |
| § 8 | Versorgungskonzept | 9 |
| § 9 | Personelle Voraussetzungen | 10 |
| § 10 | Leistungsfähigkeit | 11 |
| § 11 | Kooperationen | 12 |
| § 12 | Sächliche Voraussetzungen | 13 |
| § 13 | Sicherstellung der Hilfsmittelversorgung | 13 |
| § 14 | Verordnung von Arznei-, Hilfs- und Heilmitteln | 14 |
| § 15 | Mitteilungspflichten | 15 |
| § 16 | Dokumentation | 15 |
| § 17 | Qualitätssicherung | 16 |
| § 18 | Prüfung der Qualität der Leistungserbringung | 17 |
| § 19 | Datenschutz | 17 |
| § 20 | Haftung | 18 |
| § 21 | Vergütung | 18 |
| § 22 | Leistungsnachweis | 19 |
| § 23 | Abrechnung der Leistungen | 20 |
| § 24 | Beanstandungen | 21 |
| § 25 | Vertragsverstöße | 21 |
| § 26 | Vertragsanpassungen | 22 |
| § 27 | In-Kraft-Treten | 22 |
| § 28 | Kündigung | 22 |
| § 29 | Salvatorische Klausel | 23 |


MUSTER

## Präambel

Die spezialisierte ambulante Palliativversorgung (im Weiteren SAPV) im Sinne von § 37 b SGB V soll die Lebensqualität und Selbstbestimmung unheilbar kranker Menschen mit nur noch geringer Lebenserwartung von Tagen, Wochen oder Monaten erhalten, fördern bzw. verbessern und ihnen ein menschenwürdiges Leben bis zum Tod in ihrer vertrauten häuslichen Umgebung ermöglichen. Im Vordergrund steht anstelle eines kurativen Ansatzes die medizinisch-pflegerische Zielsetzung, Symptome und Leiden einzelfallbezogen und dem Wunsch des Versicherten entsprechend zu lindern. Die individuellen Bedürfnisse dieser Menschen sowie die Belange der vertrauten Personen stehen im Mittelpunkt der Versorgung.

Die SAPV wird auf Grundlage einer multiprofessionellen, sektorenübergreifenden Zusammenarbeit als ärztliche und pflegerische Komplexleistung sichergestellt und beinhaltet die in diesem Zusammenhang notwendigen Beratungs- und Koordinationsleistungen. Sie dient der Vermeidung von medizinisch nicht notwendigen Krankenhausaufenthalten der anspruchsberechtigten Versicherten, der im Bedarfsfall erforderlichen speziellen palliativmedizinischen Intervention im Krisenfall sowie der psychosozialen Unterstützung des Versicherten und seiner Angehörigen. Die SAPV ergänzt das allgemeine Angebot der palliativmedizinischen und palliativpflegerischen Versorgung der Primärversorger (u. a. Vertragsärzte, Krankenhäuser, Pflegedienste mit Vertrag nach § 132 a Abs. 2 SGB V und/oder § 72 SGB XI für ambulante Pflege, ambulante Hospizdienste, stationäre Pflegeeinrichtungen, Einrichtungen der Eingliederungshilfe für behinderte Menschen und der Kinder- und Jugendhilfe).

MUSTER

Den besonderen Belangen von Kindern und Jugendlichen wird bei der Versorgung Rechnung getragen.

## § 1
## Vertragspartner

Dieser Vertrag gilt ausschließlich für die nachfolgend aufgeführten Vertragspartner und Einrichtungen.

Vertragspartner sind die im Rubrum genannten Krankenkassen und die Betriebskrankenkassen, die dieser Vereinbarung beitreten

sowie

*Arzt/ Ärzte/ Krankenhaus u.ä.*

*vertreten durch...*

*Pflegefachkräfte/ Pflegedienst,*

*vertreten durch...*

(im Weiteren SAPV-Leistungserbringer[1])

Der SAPV-Leistungserbringer tritt als Organisationseinheit auf.

---

[1] Im Vertrag wird aus Gründen der Vereinfachung durchgehend nur die männliche Form verwendet. Gemeint sind jeweils gleichberechtigt männliche und weibliche Personen.

Dieser Vertrag gilt ausschließlich für den SAPV-Leistungserbringer an dem im Rubrum aufgeführten Sitz. Für räumlich getrennte Filialen, Außenstellen oder Nebenstellen, von denen aus SAPV erbracht wird, ist ein gesonderter Vertrag erforderlich.

## § 2
## Ziele des Vertrages

(1) Ziel dieses Vertrages ist ausschließlich die über die allgemeine Palliativversorgung hinausgehende Versorgung von schwerstkranken Menschen, die eine besonders aufwändige Versorgung mit spezialisierter ambulanter Palliativmedizin bzw. –pflege gemäß § 37 b SGB V (SAPV) benötigen. Diese Versorgung erfolgt in der vertrauten Umgebung des häuslichen oder/und familiären Bereiches.

(2) Generelle Grundlage für die Versorgung und Bestandteil dieses Vertrages sind

- die „Richtlinie des Gemeinsamen Bundesausschusses zur Verordnung von spezialisierter ambulanter Palliativversorgung“ (Anlage 1 - Spezialisierte Ambulante Palliativversorgungs-Richtlinie, im Weiteren SAPV-RL) vom 20. Dezember 2007 und
- die „Gemeinsamen Empfehlungen der Spitzenverbände der Krankenkassen nach § 132 d Abs. 2 SGB V für die spezialisierte ambulante Palliativversorgung“ vom 23. Juni 2008 (Anlage 2 - im Weiteren SAPV-Empfehlungen)

MUSTER

in ihrer jeweils aktuellen Fassung.

(3) Die SAPV wird vom SAPV-Leistungserbringer als ärztliche und pflegerische Komplexleistung nach den Regelungen dieses Vertrages unter Beachtung der gesetzlichen Vorschriften durchgeführt. Der SAPV-Leistungserbringer hat dafür Sorge zu tragen, dass die an der Versorgung Beteiligten zusammenarbeiten und die erforderlichen Maßnahmen aufeinander abgestimmt und bedarfsgerecht erbringen.

(4) Der SAPV-Leistungserbringer hat unter Berücksichtigung des § 1 Abs. 3 der SAPV-RL eine bedarfsgerechte und gleichmäßige, dem allgemein anerkannten Stand der medizinischen, pflegerischen, palliativen und technischen Erkenntnisse sowie dem medizinischen Fortschritt entsprechende Versorgung zu gewährleisten. Die SAPV ist ausreichend, wirtschaftlich und zweckmäßig zu erbringen und darf das Maß des Notwendigen nicht überschreiten. Bei der Leistungserbringung soll ein Personalwechsel möglichst vermieden und die Versorgung ressourcen- und angehörigenorientiert erbracht werden.

(5) Die SAPV ist eine Versorgungsform, die das bestehende Versorgungsangebot, insbesondere der Vertragsärzte, Krankenhäuser und Pflegedienste, ergänzt, damit im Rahmen einer vorausschauenden Therapie Krisen und medizinisch nicht notwendige Krankenhauseinweisungen weitgehend vermieden werden. Sie ist eine Leistung, die in der Regel sporadisch und phasenweise notwendig ist.

Die Vertragspartner verbinden mit diesem Vertrag die Erwartung, dass die Krankenhauseinweisungen und stationären Aufenthalte für die vom Vertrag betroffenen Patienten reduziert werden.

## § 3
## Versorgungsgebiet

(1) Zur Sicherstellung einer bedarfsgerechten, wohnortnahen und dem gesetzlichen Zweck entsprechenden Versorgung sowie zur Erfüllung der Leistungsverpflichtung der Krankenkassen wird ein vertraglicher Versorgungsbereich festgelegt. Innerhalb dieses Versorgungsbereichs ist der SAPV-Leistungserbringer zur Sicherstellung der Versorgung am Wohnort des Versicherten im Rahmen seiner Kapazitäten[2] verpflichtet. Die Festlegung des Versorgungsbereichs begründet keinen Anspruch auf (alleinige) Versorgung in dem Versorgungsbereich durch den betreffenden SAPV-Leistungserbringer.

Der vertragliche Versorgungsbereich des SAPV-Leistungserbringers umfasst die Region:

……………………………………………………………………………………………………
……………………………………………………………………………………………………

(2) Die Wahlfreiheit der Versicherten wird durch diese Regelungen nicht eingeschränkt.

(3) Zur Sicherstellung der wohnortnahen Versorgung hat der SAPV-Leistungserbringer in der Regel zu gewährleisten, dass jederzeit bei den zu betreuenden Versicherten eine Krisenintervention in der Häuslichkeit innerhalb von 45 min erfolgen kann.[3]

MUSTER

## § 4
## Inhalt und Umfang der Leistungen

(1) Allgemeiner Inhalt und Umfang der Leistungen ergeben sich aus § 5 der SAPV-RL. Die Tätigkeit des SAPV-Leistungserbringers umfasst den speziellen ärztlichen, pflegerischen, psychosozialen und besonderen zeitlichen, organisatorischen, koordinativen und kommunikativen Aufwand, der für die Versorgung der anspruchsberechtigten Versicherten erforderlich ist. Die SAPV wird dem aktuellen patientenindividuellen Versorgungsbedarf entsprechend intermittierend oder durchgängig als

- Beratungsleistung,
- Koordination der Versorgung,
- additiv unterstützende Teilversorgung oder
- vollständige Versorgung

erbracht.

(2) Die Aufgabe des SAPV-Leistungserbringers umfasst insbesondere die

- Koordination der spezialisierten palliativmedizinischen und palliativpflegerischen Versorgung unter Einbeziehung weiterer Berufsgruppen und von Hospizdiensten im Rahmen einer multiprofessionellen Zusammenarbeit,
- Symptomlinderung durch Anwendung von Medikamenten oder anderen Maßnahmen,
- apparative palliativmedizinische Behandlungsmaßnahmen (z. B. Medikamentenpumpe),

---

[2] Die Krankenkassen sind rechtzeitig vor dem Erreichen der Kapazitätsgrenze zu informieren.

[3] Sofern regionale Gegebenheiten eine Anpassung der Interventionszeit erforderlich machen, ist dies zwischen den Vertragspartnern bei Einhaltung der Qualitätsanforderungen zu vereinbaren.

- palliativmedizinische Maßnahmen, die nach ihrer Art, Schwere oder Komplexität eine Kompetenz erfordern, die der eines Arztes mit Zusatzweiterbildung Palliativmedizin entspricht,
- spezialisierte palliativpflegerische Leistungen, die nach ihrer Art, Schwere oder Komplexität eine Kompetenz erfordern, die der einer Pflegefachkraft mit einer curricularen Weiterbildung zu Palliative Care entspricht,
- Führung eines individuellen Behandlungsplans, vorbeugendes Krisenmanagement, Bedarfsinterventionen,
- Ruf-, Notfall- und Kriseninterventionsbereitschaft rund um die Uhr für den im Rahmen der SAPV betreuten Versicherten zur Sicherstellung der im Rahmen der SAPV erforderlichen Maßnahmen,
- Beratung, Anleitung und Begleitung des Versicherten und seiner Angehörigen zur palliativen Versorgung einschließlich Unterstützung beim Umgang mit Sterben und Tod,
- spezialisierte Beratung der betreuenden Leistungserbringer der Primärversorgung,
- psychosoziale Unterstützung im Umgang mit schweren Erkrankungen in enger Zusammenarbeit z. B. mit Seelsorge, Sozialarbeit und ambulanten Hospizdiensten,
- Organisation regelmäßiger Fallbesprechungen sowie
- Dokumentation und Evaluation der wesentlichen Maßnahmen im Rahmen der SAPV.

MUSTER

(3) Der SAPV-Leistungserbringer gewährleistet im Rahmen dieses Vertrages gegenüber den betreuten Versicherten und den Krankenkassen unter Beachtung des einschlägigen Berufsrechts eine dem allgemeinen Stand der fachlich-medizinischen Erkenntnisse entsprechende qualitätsgesicherte, wirksame, ausreichende, zweckmäßige und wirtschaftliche Versorgung des Versicherten.

## § 5
## Anspruchsberechtigte

Versicherte haben gemäß § 37 b SGB V Anspruch auf SAPV, wenn sie die Voraussetzungen der §§ 2 bis 4 der SAPV-RL in der jeweils geltenden Fassung erfüllen. Dies gilt insbesondere für die Versicherten,

- die an einer nicht heilbaren, fortschreitenden und so weit fortgeschrittenen Erkrankung leiden, dass dadurch ihre Lebenserwartung auf nur wenige Tage, Wochen und Monate begrenzt ist und denen im Rahmen der allgemeinen Palliativversorgung kein würdevolles Sterben in der häuslichen Umgebung ermöglicht werden kann,
- bei denen nach ärztlichem Ermessen kurative Maßnahmen nicht mehr indiziert bzw. von den Versicherten nicht mehr gewünscht sind,
- die unter Berücksichtigung der in der SAPV-RL genannten Ziele eine besonders aufwändige Versorgung benötigen, die nach den medizinischen und pflegerischen Erfordernissen in der häuslichen Umgebung der Versicherten erbracht werden kann und
- von denen diese Versorgung gewünscht wird.

## § 6
## Kostenübernahme

(1) Voraussetzung für die Leistungsgewährung und Leistungserbringung ist eine ordnungsgemäß ausgestellte Verordnung (Muster 63) zu Lasten der zuständigen Krankenkasse. Die näheren Bestimmungen ergeben sich aus den gesetzlichen Regelungen, den SAPV-RL und SAPV-Empfehlungen und den Bestimmungen dieses Vertrages. Rückwirkende Verordnungen sind grundsätzlich nicht zulässig.

(2) Die Verordnung ist nicht übertragbar.

(3) Art, Umfang und Dauer der vom SAPV-Leistungserbringer durchzuführenden Maßnahmen ergeben sich aus der Verordnung des behandelnden Vertragsarztes oder des im Rahmen dieses Vertrages ambulant tätigen Krankenhausarztes. Die Verordnung eines nicht ambulant tätigen Krankenhausarztes kann längstens für die Dauer von 7 Kalendertagen erfolgen. Die vom Versicherten durch Vorlage der Verordnung beantragten Leistungen (Rückseite der Verordnung) bedürfen der Genehmigung durch die Krankenkasse. Die Verordnung kann der Krankenkasse zur Sicherstellung einer zeitnahen Bearbeitung vorab per Fax vorgelegt werden. Die Grundsätze des datenschutzgerechten Fax-Einsatzes sind zu beachten.

Ist in begründeten Fällen, die nicht vom SAPV-Leistungserbringer zu vertreten sind, die Unterschrift des Versicherten oder seines gesetzlichen Vertreters unter der Verordnung zu diesem Zeitpunkt noch nicht möglich, wird die Unterschrift unverzüglich nachgereicht. Kann eine Unterzeichnung der Verordnung nicht erfolgen, ist eine entsprechende Begründung anzugeben. Die Unterschrift durch den Leistungserbringer bzw. dessen Mitarbeiter ist ausgeschlossen. § 13 SGB X bleibt unberührt.

(4) Änderungen oder Ergänzungen der vertragsärztlichen Verordnung zur SAPV bedürfen einer erneuten Arztunterschrift mit Stempel und Datumsangabe.

MUSTER

(5) Die zuständige Krankenkasse übernimmt gemäß § 8 SAPV-RL vom ärztlich festgelegten Leistungsbeginn an bis zur Entscheidung über die Genehmigung die Kosten für die vom SAPV-Leistungserbringer durchgeführten Leistungen (vorläufige Kostenübernahme), wenn die ordnungsgemäß ausgefüllte Verordnung (Original, nicht Fax) spätestens an dem dritten der Ausstellung folgenden Arbeitstag der Krankenkasse vorliegt.

Die Vertragspartner sind sich darüber einig, dass bis zur Genehmigung des Leistungsantrages nur die für den Versicherten nachweislich notwendigen Hausbesuchsleistungen zur Krisen-/Notfallversorgung erbracht und vergütet werden. Die Leistungen des Erstbesuches gemäß § 7 Abs. 6 sind in diesem Zusammenhang mit zu erbringen.

Die vorläufige Kostenübernahme gilt nicht, sofern der SAPV-Leistungserbringer vor Leistungsbeginn hätte erkennen können, dass ein Leistungsanspruch gemäß § 37 b SGB V in Verbindung mit den SAPV-RL für den betreffenden Versicherten nicht gegeben ist.

(6) Die Krankenkasse informiert den Arzt, den SAPV-Leistungserbringer und den Versicherten unverzüglich über die getroffene Leistungsentscheidung. Liegt der zuständigen Krankenkasse innerhalb von 7 Werktagen ab Verordnungsbeginn zusätzlich zur Erstverordnung schriftlich kein Ergebnis über das im Rahmen des Erstbesuches durchgeführten Assessments gemäß § 16 Abs. 2 a) Satz 2 in Verbindung mit § 7 Abs. 5 und 6 einschließlich der Angabe zum Datum des erfolgten Erstbesuches vor, endet die Kostenübernahme mit Ablauf dieses Tages. Satz 1 gilt entsprechend.

(7) Die zuständige Krankenkasse kann zur Prüfung der Leistungspflicht den Medizinischen Dienst der Krankenversicherung (im Weiteren MDK) nach Maßgabe der gesetzlichen Regelungen einschalten. Der MDK kann zur Prüfung der Verordnung weitere Unterlagen-vom SAPV-Leistungserbringer anfordern; diese sind zeitnah zur Verfügung zu stellen.

(8) Der SAPV-Leistungserbringer übermittelt die geforderten Unterlagen nach diesem Vertrag an die Krankenkasse bzw. den MDK.

## § 7
## Grundsätze für die Leistungserbringung

(1) Der SAPV-Leistungserbringer hat grundsätzlich dafür Sorge zu tragen, dass Verordnungen kooperierender ambulanter Vertragsärzte, Krankenhausärzte bzw. Palliativärzte (vgl. § 11 Abs. 3) vor deren Ausstellung gemeinsam mit ihm abgestimmt werden.

(2) Wurde die Erstverordnung nicht durch einen kooperierenden Arzt im Sinne des Absatzes1 ausgestellt, nimmt ein Palliativarzt des SAPV-Leistungserbringers Kontakt zum verordnenden Arzt auf und klärt den konkreten Versorgungsbedarf vor Beginn der Leistungserbringung ab. Stellt dieser Palliativarzt des SAPV-Leistungserbringers dabei fest, dass die Anspruchsvoraussetzungen für eine SAPV beim Versicherten nicht gegeben sind und dass eine angemessene Versorgung im Rahmen der allgemeinen Palliativversorgung (Primärversorgung) sichergestellt werden kann, berät er den verordnenden Arzt zur Einleitung geeigneter Maßnahmen.

(3) Der SAPV-Leistungserbringer hat sich bei Notwendigkeit einer Folgeverordnung grundsätzlich vorher mit dem verordnenden Arzt abzustimmen.

(4) Im Zusammenhang mit den Regelungen der Abs. 1 bis 3 wirkt der SAPV-Leistungserbringer darauf hin, dass die verordnenden Ärzte die von der zuständigen Krankenkasse oder dem MDK erteilten Hinweise zur Ausstellung der Verordnung beachten und die an sie herangetragenen Fragen unverzüglich beantworten.

MUSTER

(5) Der SAPV-Leistungserbringer hat eine Rückführung des Versicherten in die Primärversorgung zu organisieren, wenn er während seiner Tätigkeit feststellt, dass der Leistungsanspruch des Versicherten gemäß § 37 b SGB V nicht oder zum aktuellen Zeitpunkt nicht mehr besteht, Abs. 2 Satz 1 und 2 gilt entsprechend (vgl. § 2 Abs. 1).

(6) Die Leistungserbringung beginnt grundsätzlich mit der Durchführung des Erstbesuches. Dieser ist spätestens nach Erhalt der Verordnung innerhalb von 2 Werktagen von einem Palliativarzt gemäß § 9 Abs. 1 a) und Abs. 2 durchzuführen und kann in Begleitung einer Palliativpflegefachkraft gemäß § 9 Abs. 1 b) und Abs. 3 erfolgen.

Bei zwingend notwendigen Hausbesuchen zur Krisenintervention vor Durchführung eines Erstbesuches sind die Leistungen nach Abs. 7 Sätze 2 und 3 zu integrieren.

(7) Der Erstbesuch findet in der Regel in der Häuslichkeit des Versicherten statt. Während des Erstbesuches ist ein Assessment gemäß § 16 Abs. 2 a) zu erstellen. Der SAPV-Leistungserbringer hat dabei die für die Versorgung notwendigen Einwilligungserklärungen (z. B. die Entbindung des behandelnden Arztes von der Schweigepflicht gegenüber dem SAPV-Leistungserbringer) vom Versicherten oder seinem gesetzlichen Vertreter einzuholen. Empfohlen wird die Verwendung des beigefügten Musters gemäß Anlage 9. Bei erkennbarem Bedarf soll der SAPV-Leistungserbringer den Versicherten über die Möglichkeiten der Beantragung von Pflegeleistungen informieren.

(8) Sofern der Leistungsantrag nicht durch den SAPV-Leistungserbringer bei der Krankenkasse eingereicht wurde, wird ihm die Verordnung von der Krankenkasse per Fax unter Beachtung der Grundsätze des datenschutzgerechten Fax-Einsatzes zur Verfügung gestellt.

(9) Die Ablehnung einzelner Maßnahmen im Rahmen der SAPV durch den Versicherten führt nicht zum Abbruch der Leistungserbringung, soweit der Anspruch gemäß § 37 b SGB V sowie der SAPV-RL weiterhin gegeben ist.

(10) Die SAPV ruht im genehmigten Verordnungszeitraum für die Dauer eines vollstationären Krankenhausaufenthaltes bzw. für die Dauer der Versorgung in der Primärversorgung.

(11) Versicherte, welche in stationären Hospizen versorgt werden, haben Anspruch auf die Teilleistung der erforderlichen ärztlichen Versorgung im Rahmen der spezialisierten ambulanten Palliativversorgung.

(12) Die SAPV kann in einer stationären Pflegeeinrichtung nach § 72 Abs. 2 SGB XI von dem SAPV-Leistungserbringer nur in dem Maße erbracht werden, wie der Versorgungsbedarf des Versicherten die Leistungsverpflichtung der Pflegeeinrichtung und die Verpflichtungen der weiteren an der allgemeinen Palliativversorgung beteiligten Leistungserbringer überschreitet.

(13) Ist eine Weiterversorgung des Versicherten nicht möglich (z. B. wenn die Häuslichkeit außerhalb des Versorgungsbereiches verlegt wird), nimmt der SAPV-Leistungserbringer unverzüglich Kontakt mit der zuständigen Krankenkasse auf.

(14) Die Annahme von Verordnungen und deren Weitergabe bzw. Vermittlung an Dritte gegen Entgelt oder zur Erlangung geldwerter Vorteile ist unzulässig. Vermittlung im Sinne dieser Bestimmung ist auch die Weitergabe von in eigenem Namen angenommenen Aufträgen an Dritte gegen Kostenerstattung.

MUSTER

## § 8
## Versorgungskonzept

(1) Zur Sicherstellung der geforderten Qualität der Leistungserbringung arbeitet der SAPV-Leistungserbringer nach einem verbindlichen, strukturierten und schriftlich dargelegten Versorgungskonzept. In diesem Konzept sind der inhaltliche und organisatorische Rahmen der Leistungserbringung, orientiert an der Konzeption eines Palliativ-Care-Teams beschrieben. Das Konzept ist Bestandteil dieses Vertrages und den Krankenkassen in der jeweils aktuellen Fassung vorzulegen.

(2) Das Versorgungskonzept beinhaltet mindestens folgende Schwerpunkte:

1. Allgemeine Angaben zum Antragsteller/ Leistungserbringer (u.a. Rechtsform; Historie; Einbindung in die regionale Versorgungsstruktur; Standort und Versorgungsregion)

2. Strukturelle und organisatorische Grundlagen für die Leistungserbringung (Darlegungen zur sächlichen und personellen Ausstattung einschl. Qualifikationsanforderungen, Organigramm/ Stellenplan beifügen, zu den Regelungen zur Zuständigkeit und den Verantwortlichkeiten – Weisungsbefugnisse/-bindungen, zu den bestehenden Kooperationsvereinbarungen),

3. Vorstellungen zur SAPV-Leistung (Darlegungen zu Zielgruppe, zu Inhalt und Umfang der Leistungserbringung einschl. ausführlicher und nachvollziehbarer Vorstellungen zur Abgrenzung zwischen allgemeiner und spezialisierter Palliativversorgung),

4. Versorgungsprozess (Darlegungen zur Organisation/ Koordination der Tätigkeit, zur Planung einer 24-stündigen Ruf- und Einsatzbereitschaft, zur Dienstplanung (Musterplanung beifügen), zu den Bürozeiten, zu den Aufgaben und zur Einbindung der Kooperationspartner, zur Sicherstellung der Heil-, Hilfs- und Arzneimittelversorgung und zum Mengengerüst (Fallzahlschätzung prospektiv p. a.),

5. Maßnahmen zur Qualitätssicherung/ Weiterentwicklung der Versorgungsstrukturen (Darlegungen zu multiprofessionellen Teambesprechungen, Supervisionen, Qualitätszirkel, zum Fortbildungsplan einschl. Art und Umfang, zur Dokumentation (Muster der Patientendokumentation beifügen), zu den geplanten Aktivitäten innerhalb der regionalen Versorgungsstruktur, zur Teilnahme an der bundeseinheitlichen Evaluation und Dokumentation, zu weiteren statistischen Datenerhebungen für die vertragsschließenden Krankenkassenverbände zur Weiterentwicklung des Vertrages).

## § 9
## Personelle Voraussetzungen

(1) Für die Dauer des Vertrages ist vom SAPV-Leistungserbringer sicherzustellen und jederzeit nachzuweisen, dass

a) mindestens 5 qualifizierte Ärzte (im Weiteren Palliativärzte), welche die Qualifikation nach Abs. 2 nachweisen können und die ihren Tätigkeitsschwerpunkt in der Versorgung von Palliativpatienten haben, für die SAPV nach diesem Vertrag zur Verfügung stehen.

   aa) Zwei dieser Palliativärzte müssen jeweils mit einer Arbeitszeit von mindestens 19 Stunden/Woche nachweisbar und klar abgegrenzt ausschließlich für die Erbringung der SAPV nach diesem Vertrag zur Verfügung stehen und sind mit diesem Vertrag zur Leistungserbringung direkt verpflichtet. Eine vertragliche Bindung über Kooperationsverträge ist unzulässig.

MUSTER

   bb) Für drei der unter a) genannten Palliativärzte kann eine vertragliche Bindung über eine Kooperationsvereinbarung nach § 11 in Betracht kommen.

b) mindestens 4 qualifizierte Pflegefachkräfte (im Weiteren Palliativpflegefachkräfte) in Vollzeit[4], welche die Qualifikation nach Abs. 3 besitzen, beim SAPV-Leistungserbringer beschäftigt sind und der Leistungserbringung nach diesem Vertrag zur Verfügung stehen. Davon können 2 Vollzeitkräfte auch im Rahmen eines Stellensplittings tätig werden. Die Arbeitszeit muss dabei mindestens die Hälfte der regelmäßigen Wochenarbeitszeit einer Vollzeitkraft[4] betragen. Eine vertragliche Bindung über Kooperationsverträge für diese Kräfte ist unzulässig.

(2) Der Palliativarzt muss über folgende Qualifikationen verfügen:

   a) eine anerkannte Zusatzweiterbildung Palliativmedizin nach der aktuell gültigen Weiterbildungsordnung der zuständigen Landesärztekammer (grundsätzlich 160 Std. Weiterbildung)

   und

   b) Erfahrung aus der palliativen ambulanten Behandlung von nachweislich mindestens 75 Palliativpatienten, z. B. in der häuslichen Umgebung oder in einem stationären Hospiz, innerhalb der letzten drei Jahre oder aus einer mindestens 1-jährigen klinischen palliativmedizinischen Tätigkeit in einer Palliativabteilung.

---
[4] Mit Vollzeit ist eine regelmäßige wöchentliche Arbeitszeit von 40 h gemeint, sofern im Einzelfall nicht andere tarifrechtliche Rahmenbedingungen bestehen.

Die vertraglich verpflichteten Palliativärzte weisen entsprechende arbeitsvertragliche Regelungen nach, welche die SAPV-Tätigkeit in dem beschriebenen Umfang zu ihren sonstigen Tätigkeiten abgrenzt. Niedergelassene Palliativärzte legen eine entsprechende Verpflichtungserklärung zum geforderten Tätigkeitsumfang vor.

(3) Die Palliativpflegefachkraft muss über folgende Qualifikationen verfügen:

a) die Erlaubnis zur Führung einer der Berufsbezeichnungen Gesundheits- und Krankenpfleger, Gesundheits- und Kinderkrankenpfleger oder Altenpfleger (3-jährige Ausbildung aufgrund der bundes- oder landesrechtlichen Regelung) entsprechend den gesetzlichen Bestimmungen in der jeweils gültigen Fassung,

b) den Abschluss einer Palliative-Care-Weiterbildungsmaßnahme im Umfang von mindestens 160 Stunden und

c) Erfahrung durch eine mindestens 2-jährige praktische Tätigkeit als Pflegefachkraft in der Betreuung von Palliativpatienten in den letzten drei Jahren; davon mindestens sechsmonatige Mitarbeit in einer spezialisierten Einrichtung der Hospiz- und Palliativversorgung.

MUSTER

(4) Sofern Kinder und Jugendliche versorgt werden, müssen die Anforderungen an die ärztlichen und pflegerischen SAPV-Leistungserbringer und an die weiteren Fachkräfte dem besonderen Bedarf von Kindern und Jugendlichen gerecht werden.

**§ 10**
**Leistungsfähigkeit**

(1) Der SAPV-Leistungserbringer sichert eine tägliche 24-stündige telefonische Ruf- und Einsatzbereitschaft für den Versicherten, deren Angehörige bzw. alle an der Versorgung Beteiligten ab (vgl. § 3). Er sorgt dafür, dass ständig mindestens ein Palliativarzt und eine Palliativpflegefachkraft mit Qualifikation gemäß § 9 Abs. 2 bzw. 3 einsatzbereit sind und bei Bedarf Hausbesuche absichern.

(2) Der SAPV-Leistungserbringer ist Teil einer multiprofessionell vernetzten regionalen Versorgungsstruktur, gewährleistet eine psychosoziale Unterstützung der Versicherten und seiner Angehörigen und arbeitet mit Vertretern der ambulanten Hospizdienste, der Seelsorge und der Sozialarbeit zusammen.

(3) Der SAPV-Leistungserbringer organisiert und koordiniert die Leistungserbringung unter besonderer Berücksichtigung des § 6 der SAPV-RL in geeigneter Weise. Er stellt eine größtmögliche Kontinuität bei der Versorgung sicher, damit die Leistungen für den Versicherten von möglichst wenigen Personen (Grundsatz der Bezugspflege) erbracht werden. Die Kommunikation aller an der Versorgung Beteiligten erfolgt bedarfsgerecht und unter Nutzung effektiver Kommunikationswege.

(4) Der SAPV-Leistungserbringer bzw. seine Mitarbeiter sind an keine Weisungen von-Leistungserbringern anderer Bereiche bzw. Einrichtungen gebunden. Die Palliativpflegefachkräfte gemäß § 9 Abs. 3 sind während ihrer SAPV-Tätigkeit allein gegenüber dem jeweils zuständigen Palliativarzt weisungsgebunden.

(5) Der SAPV-Leistungserbringer hat die Erfüllung der personellen, sächlichen und sonstigen Voraussetzungen zum Vertragsabschluss gemäß Anlage 3 (Strukturerhebungsbogen) vor Vertragsabschluss nachzuweisen. Der Einsatz freier Mitarbeiter bzw. als vertraglich gebundener Kooperationspartner ist nur möglich, soweit die personelle Mindest-

besetzung des § 9 Abs. 1 davon nicht betroffen wird und eine fachliche, disziplinarische und organisatorische Einbindung (u. a. in eine verbindliche Dienstplanung) in die Tätigkeit des SAPV-Teams erfolgt.

(6) Der SAPV-Leistungserbringer ist verpflichtet sicherzustellen, dass mit Vertragsabschluss und während der gesamten Laufzeit des Vertrages eine Betriebs- und Berufshaftpflichtversicherung für Personen-, Sach- und Vermögensschäden in ausreichender Höhe besteht. Dies gilt auch für die SAPV-Tätigkeit der kooperierenden Partner. Bei Änderungen der Betriebsgröße ist die Versicherung anzupassen. Der Versicherer ist auf Anforderung der Krankenkasse mit Namen und Adresse zu benennen.

## § 11
## Kooperationen

(1) Der SAPV-Leistungserbringer weist vor Abschluss dieses Vertrages mindestens folgende Kooperationsvereinbarungen nach:

- mit einem ambulanten Hospizdienst und
- mit mindestens einem Krankenhaus im Versorgungsgebiet oder versorgungsnahem Gebiet (vorzugsweise Krankenhäuser mit Palliativstation)

MUSTER

Die Kooperation mit einem Krankenhaus soll insbesondere der effizienten Überleitung des Versicherten aus dem Krankenhaus in die Häuslichkeit bzw. der reibungslosen Aufnahme in das Krankenhaus bei entsprechend dringlichem Bedarf dienen. Im Rahmen der Kooperation ist ein geeignetes Überleitungsmanagement zu vereinbaren.

(2) Zur Sicherstellung einer qualitätsgerechten und effizienten Versorgung der Versicherten sollte der SAPV-Leistungserbringer im Sinne des § 6 der SAPV-RL darüber hinaus mit den übrigen an der Versorgung beteiligten Leistungserbringern kooperieren, soweit in diesem Vertrag nichts anderes geregelt ist. Dies betrifft auch die Zusammenarbeit mit einem Sozialarbeiter, Sozialpädagogen oder Psychologen, der eine Zusatzweiterbildung Palliative Care für andere Berufsgruppen oder praktische Erfahrungen in der Palliativversorgung nachweisen kann.

(3) Zur Sicherstellung der geforderten Versorgungsqualität soll mit den Haus- und Vertragsärzten, welche Versicherte im Versorgungsgebiet betreuen, kooperiert werden.

Die Zusammenarbeit ist insbesondere auf
- die Abstimmung des Haus- und Vertragsarztes mit dem Palliativarzt vor den Verord nungen der SAPV und vor Krankenhauseinweisungen,
- die Prüfung und gemeinsame Festlegung der notwendigen Versorgung während der SAPV,
- die Abstimmung des Hilfsmittelbedarfes unter Berücksichtigung des § 13 sowie
- die Nutzung effektiver Kommunikationswege

zu richten.

(4) Die Regelungen in den Kooperationsverträgen sind in Übereinstimmung mit den Verpflichtungen dieses Vertrages zu vereinbaren. Es sind mindestens der Leistungsinhalt und -umfang, die Teilnahme an Qualitätssicherungsmaßnahmen und der Umfang von Fortbildungen sowie Festlegungen für eine ausreichende Betriebs- und Berufshaftpflichtversicherung zu regeln.

(5) Die Krankenkassen können in begründeten Fällen die Anerkennung geschlossener Kooperationsvereinbarungen versagen, wenn Anforderungen dieses Vertrages nicht erfüllt werden.

(6) Der SAPV-Leistungserbringer führt Übersichten zu den aktuell bestehenden Kooperationsvereinbarungen mit Angabe zum Beginn/Ende der Vereinbarung gemäß Anlagen 4 und 5. Die Übersichten sowie die Kooperationsverträge sind auf Anforderung der Krankenkasse vorzulegen.

## § 12
## Sächliche Voraussetzungen

(1) Der SAPV-Leistungserbringer hat als Mindestanforderung an die sächliche Ausstattung Folgendes vorzuhalten bzw. sicherzustellen:

a) eine geeignete, aktuell geführte und für die an der Versorgung Beteiligten jederzeit zugängliche Patientendokumentation,

b) eine ausreichende und geeignete Mobilität zur zeitnahen häuslichen Versorgung der Patienten,

c) Arzneimittel (inkl. Betäubungsmittel - im Weiteren BTM) und Hilfsmittel[5] für die Notfall/Krisenintervention,

d) Arzt-/Pflegekoffer/Bereitschaftstasche (Berücksichtigung der Kompatibilität der Verbrauchsmaterialien zu Medizinprodukten unterschiedlicher Hersteller, z. B. bei Portsystemen und Infusionspumpen) sowie

e) eine geeignete administrative Infrastruktur, z. B. Büro, Kommunikationstechnik.

MUSTER

(2) Der SAPV-Leistungserbringer verfügt über eine eigenständige Adresse und geeignete Räumlichkeiten für

- die Beratung von Patienten und Angehörigen,
- Teamsitzungen und Besprechungen sowie
- die Lagerung von eigenen Medikamenten für Notfall- / Krisenintervention und für Hilfsmittel[5].

Die BTM sind gemäß den Regelungen des Betäubungsmittelgesetzes in einem BTM-Schrank aufzubewahren.

## § 13
## Sicherstellung der Hilfsmittelversorgung

(1) Für die Versorgung des Versicherten mit Hilfsmitteln hat der SAPV-Leistungserbringer geeignete Maßnahmen einzuleiten. Die Belieferung der notwendigen Hilfsmittel erfolgt grundsätzlich im Rahmen des jeweiligen Hilfsmittelverfahrens der einzelnen Krankenkasse.[5]

(2) Die Vereinbarungen der zuständigen Krankenkasse nach § 127 SGB V sind einzuhalten.[5]

---

[5] Zu den entsprechenden Verträgen hat sich der SAPV-Leistungserbringer bei der zuständigen Krankenkasse zu erkundigen.

(3) Der SAPV-Leistungserbringer kann mit grundsätzlich einem von ihm ausgewählten zur Versorgung berechtigten Hilfsmittelleistungserbringer eine Kooperationsvereinbarung auch zu Lasten einer Krankenkasse schließen, soweit diese ihn hierzu ermächtigt.

## § 14
## Verordnung von Arznei-, Hilfs- und Heilmitteln im Rahmen der SAPV

(1) Der Palliativarzt des SAPV-Leistungserbringers verordnet die notwendigen Arznei-, Hilfs- und Heilmittel unter Verwendung der Muster 13, 14, 16 bzw. 18 auf den Namen des Versicherten. Bei (zeitgleicher) Verordnung von Arzneimitteln einerseits und Hilfsmitteln andererseits sind jeweils getrennte Vordrucke nach Muster 16 zu verwenden.

(2) Der SAPV-Leistungserbringer benötigt für die Verordnung von Arznei,- Hilfs- und Heilmitteln eine Betriebsstättennummer. Diese Betriebsstättennummer ist nur für Verordnungen im Rahmen der SAPV auf Basis einer gültigen SAPV-Verordnung zu verwenden. Die Beantragung erfolgt nach den Maßgaben der Vereinbarung zwischen dem GKV-Spitzenverband sowie der Kassenärztlichen Bundesvereinigung (KBV). Die dabei anfallenden Kosten sind vom SAPV-Leistungserbringer zu übernehmen. Über die vergebene Betriebsstättennummer sind die vertragsschließenden Krankenkassen unverzüglich zu informieren. Die Betriebsstättennummer verliert ihre Gültigkeit mit Beendigung des Vertrages. Die Krankenkassen behalten sich eine entsprechende Meldung an die KBV vor.

MUSTER

(3) Zur eindeutigen Abgrenzung der Verordnungen im Rahmen der SAPV von der sonstigen vertragsärztlichen Versorgung ist die einheitliche Pseudo-Arztnummer 333333300 in den Vordruck einzutragen. Diese Pseudo-Arztnummer ist nur für Verordnungen im Rahmen der SAPV auf Basis einer gültigen SAPV-Verordnung zu verwenden.

(4) Auf dem Verordnungsformular für Betäubungsmittel sind die SAPV-Betriebsstättennummer in das Feld „Vertragsarztnummer“ und die Pseudo-Arztnummer in das Feld „ VK gültig bis“ einzutragen. Diese Regelung gilt bis zur Anpassung des Formulars durch die Bundesebene.

(5) Für die Leistungserbringung gilt das Wirtschaftlichkeitsgebot gemäß § 12 SGB V und die gesetzlichen Regelungen der §§ 31 bis 34 SGB V i. V. m. den Richtlinien nach § 92 Abs. 1 Satz 2 Nr. 6 SGB V in ihrer jeweils aktuellen Fassung. Eine Nichtbeachtung dieser Regelungen kann zu entsprechender Kürzung des Honorars nach vorheriger Anhörung des Leistungserbringers führen.

(6) Die Krankenkassen behalten sich vor, die Palliativärzte des SAPV-Leistungserbringers zu den Arzneimittelverordnungen zu beraten.

(7) Für die im Rahmen der SAPV verordneten Arznei-, Heil-, und Hilfsmittel gelten die nach §§ 300 und 302 SGB V vereinbarten Abrechnungsverfahren entsprechend.

## § 15
## Mitteilungspflichten

(1) Der SAPV-Leistungserbringer ist verpflichtet, der Krankenkasse alle Umstände unverzüglich schriftlich zu melden, welche die betrieblichen und organisatorischen Gegebenheiten betreffen, insbesondere Änderungen zum Versorgungskonzept gemäß § 8, Nichteinhaltung der Voraussetzungen bzw. Änderungen zu den Angaben in Anlage 3 (Strukturerhebungsbogen gemäß § 10 Abs. 5 in Verbindung mit §§ 9, 11, 12).

(2) Der SAPV-Leistungserbringer hat bis zum Ende des folgenden Quartals für die Wiederherstellung der vertraglichen Voraussetzungen zu sorgen und die entsprechenden Nachweise zu erbringen. Das gilt nicht für den Fall des § 28 Absatz 2 Satz 2.

(3) Er nutzt für die Meldungen gemäß Abs. 1 und 2 die jeweils zutreffenden Meldebögen der Anlagen 3 a bis c sowie 4 und 5.

(4) Der SAPV-Leistungserbringer informiert die Vertragspartner unverzüglich über eingeleitete polizeiliche und staatsanwaltliche Ermittlungsverfahren, die für eine ordnungsgemäße Vertragserfüllung und seine Geeignetheit zur Leistungserbringung nach diesem Vertrag von Bedeutung sind.

## § 16
## Dokumentation

(1) Der SAPV-Leistungserbringer ist zur Dokumentation des Leistungsgeschehens verpflichtet. Das Dokumentationssystem muss patientenbezogene Daten und - soweit vorhanden - allgemein anerkannte Indikatoren für eine externe Qualitätssicherung enthalten und eine bundesweite Evaluation ermöglichen. Es soll so weit wie möglich kompatibel zu den bestehenden Dokumentationssystemen der Netzwerkpartner sein.

MUSTER

(2) Die Dokumentation ist sachgerecht und kontinuierlich zu führen. Die Dokumentation umfasst mindestens

a) Assessment

Das Assessment spiegelt die Situation des Versicherten wieder, u.a. das aktuelle Symptomgeschehen sowie dessen Schwere und Verlauf. Das Assessment erfolgt den allgemein anerkannten palliativmedizinischen und palliativpflegerischen Erkenntnissen entsprechend. Im Ergebnis stellt der SAPV-Leistungserbringer fest, ob der jeweilige Leistungsfall der SAPV oder der allgemeinen Palliativversorgung zuzuordnen ist.

Das Assessment erfolgt

- beim Erstbesuch,
- im Zusammenhang mit einer Folgeverordnung, ggf. auch Erstverordnung,
- sofern sich Änderungen im Versorgungsumfang des Versicherten (z. B. auch bei einer Rückführung in die allgemeine Palliativversorgung) ergeben und
- auf Anforderung der Krankenkasse und des MDK.

Sofern im Vertrag nichts anderes geregelt ist, ist der Assessmentbogen dem MDK auf Anforderung der Krankenkasse oder des MDK zuzusenden. Die Übermittlung per Fax ist möglich und erfolgt unter Beachtung der Grundsätze des datenschutzgerechten Fax-Einsatzes. Erfolgt die Übermittlung über die Krankenkasse, so ist der Bogen in einem verschlossenen Umschlag zu senden und der Umschlag mit dem Vermerk zu versehen „Nur durch den MDK zu öffnen“.

b) Patientendokumentation bestehend aus

- Versichertenstammblatt,
- Behandlungsplanung,
- Durchführungsnachweis,
- Notfallplanung (vorausschauende Medikation, Maßnahmenplanung),
- Befunde/ärztliches Verordnungsblatt,

- Berichtebogen (Beurteilung der eingeleiteten Maßnahmen),
- Überleitungsbogen (z. B. für das Krankenhaus),
- bei Bedarf: z. B. Wundprotokoll, Lagerungs-/Bewegungsplan, Ernährungs-/ Trinkpläne und –protokolle, Vitalzeichenblatt.

Die versorgungsrelevanten Dokumentationsunterlagen sind, von begründeten Ausnahmefällen (z. B. Krankenhausaufenthaltszeiten) abgesehen, beim Versicherten zu führen und zu hinterlegen, insbesondere sofern ein Primärleistungserbringer in die ambulante Versorgung des Versicherten eingebunden ist. In jedem Fall muss beim Versicherten mindestens ein Krisenbogen geführt werden, aus welchem die aktuelle Situationsbeschreibung zum Gesundheitszustand des Versicherten, die für den Not-/Krisenfall vorgesehenen Maßnahmen bzw. notwendigen Informationen sowie der telefonische Kontakt des SAPV-Leistungserbringers hervorgehen.

c) Dokumentation der Daten zur bundesweiten Evaluation

Die Dokumentation erfolgt nach den jeweils aktuellen Festlegungen des Gemeinsamen Bundesausschusses. Weitergehende Regelungen bleiben den vertragsschließenden Krankenkassen und Krankenkassenverbänden vorgehalten.

d) Datenstatistik

MUSTER

Der SAPV-Leistungserbringer erhebt zur Weiterentwicklung des Vertrages kontinuierlich die Daten gemäß Anlage 6. Er sendet diese jährlich einmal jeweils bis zum 28. Februar für die im Vorjahr abgeschlossenen Leistungsfälle bzw. auf Anforderung innerhalb von einem Monat der Krankenkasse oder dem zuständigen Landesverband zu.

Die Dokumentationsunterlagen sind nach Abschluss der Versorgung des Versicherten noch mindestens 10 Jahre beim SAPV-Leistungserbringer aufzubewahren.

## § 17
## Qualitätssicherung

(1) Der SAPV-Leistungserbringer trägt die Verantwortung für die qualitätsgerechte Leistungserbringung. Er ist verpflichtet, das im Versorgungskonzept dargestellte interne Qualitätsmanagement umzusetzen und die fachliche Kompetenz der vertraglich in die SAPV der Versicherten eingebunden Palliativpflegefachkräfte durch geeignete und geplante Fort- oder Weiterbildungen sicherzustellen. Die an der Versorgung Beteiligten sollen sich an Maßnahmen der externen Qualitätssicherung beteiligen. Die Maßnahmen zur Sicherung der ärztlichen Versorgungsqualität richten sich dabei nach § 135 a Abs. 2 SGB V.

(2) Maßnahmen zur Qualitätssicherung sind insbesondere:

- die interdisziplinär abgestimmte, individuelle Behandlungsplanung,
- die Führung eines geeigneten Dokumentationssystems,
- Vorhaltung und Studium von Fachliteratur,
- die am Bedarf orientierte Dienst-, Personaleinsatz- und Tourenplanung,
- kontinuierliche Dienstberatungen,
- regelmäßige multiprofessionelle Fallbesprechungen,
- regelmäßige fachspezifische und sektorenübergreifende regionale Qualitätszirkel mit den an der Patientenversorgung Beteiligten (mind. halbjährlich),

- Supervision,
- Teilnahme an den multidisziplinären Netzwerktreffen,
- Umsetzung des Fort- und Weiterbildungsplanes,
- Abstimmungen von Schnittstellen (Hilfsmittel, Hausarzt, Krankenhaus, Pflegedienst usw.) sowie
- statistische Erhebungen.

Die Dokumentation dieser Maßnahmen erfolgt in geeigneter Weise z. B. durch Teilnehmerlisten, Tagesordnungen und Protokolle. Sie ist 10 Jahre aufzubewahren.

## § 18
## Prüfung der Qualität der Leistungserbringung

Die Krankenkassen prüfen die Voraussetzungen, Art und Umfang der Leistungen sowie die ordnungsgemäße Abrechnung der Leistungen. Sie können dazu auch den MDK beauftragen (§ 275 SGB V). Die Prüfung kann gleichzeitig auch der Prüfung der Qualität der Leistungserbringung dienen.

Die Prüfungen erfolgen entweder unangemeldet in den Räumlichkeiten des SAPV-Leistungserbringers, des Versicherten oder nach Vorlage der angeforderten Unterlagen bei der Krankenkasse bzw. dem MDK.

MUSTER

In die Prüfung werden insbesondere folgende Unterlagen einbezogen:

- Dokumentationsunterlagen gemäß § 16 Abs. 2 a) und 2 b),
- Dienst-/Personaleinsatz- und Tourenpläne,
- Unterlagen/Nachweise zu den qualitätssichernden Maßnahmen gemäß § 17 sowie
- Abrechnungsunterlagen gemäß §§ 22 und 23
- Versorgungskonzept.

Die entsprechenden Unterlagen sind dem MDK bzw. der Krankenkasse unmittelbar zur Prüfung vorzulegen bzw. bei Anforderung der Krankenkasse oder dem MDK zeitnah zu übermitteln.

Die Übersendung/ Übergabe von Dokumentationsunterlagen gemäß § 16 Abs. 2 a) und 2 b) an die Krankenkasse oder den zuständigen Landesverband erfolgt in einem verschlossenen Umschlag mit der Kennzeichnung „Nur durch den MDK zu öffnen“.

Prüfungen in der Häuslichkeit des Versicherten sind nur zulässig, sofern der Versicherte oder seine Angehörigen einwilligen.

## § 19
## Datenschutz

(1) Der SAPV-Leistungserbringer ist verpflichtet, die gesetzlichen und vertraglichen Bestimmungen über den Schutz der patientenbezogenen Daten in der jeweils geltenden Fassung einzuhalten. Er ist dazu verpflichtet, den Schutz der personenbezogenen Daten sicherzustellen. Personenbezogene Daten dürfen nur zur Erfüllung der sich aus diesem Vertrag ergebenden Aufgaben erhoben, verarbeitet und genutzt werden.

(2) Der SAPV-Leistungserbringer unterliegt hinsichtlich der persönlichen Daten der Versicherten dem Datenschutz. Ausgenommen hiervon sind nach den einschlägigen gesetzlichen Vorschriften in der jeweils geltenden Fassung Angaben gegenüber den beteiligten behandelnden Ärzten und sonstigen Leistungserbringern, dem MDK und den Krankenkassen, soweit diese zur Erfüllung der gesetzlichen Aufgaben und zur Umsetzung dieses Vertrages erforderlich sind. Der SAPV-Leistungserbringer verpflichtet seine Mitarbeiter und Kooperationspartner zur Beachtung der Schweigepflicht und der Datenschutzbestimmungen.

## § 20
## Haftung

(1) Allgemeiner Inhalt, Umfang und Art der Leistungen ergeben sich aus den vertraglichen Regelungen in Verbindung mit der jeweils gültigen Fassung der SAPV-RL und den Regelungen des SGB V. Der SAPV-Leistungserbringer gewährleistet im Rahmen dieser Vereinbarung gegenüber den Krankenkassen und den Versicherten unter Beachtung des einschlägigen Berufsrechts eine dem allgemeinen Stand der fachlich-medizinischen Erkenntnisse entsprechende qualitätsgesicherte, wirksame, ausreichende, zweckmäßige und wirtschaftliche Versorgung der Versicherten.

MUSTER

(2) Im Rahmen des Betreuungsverhältnisses ist der SAPV-Leistungserbringer mit der Übernahme der Betreuung auch dem Versicherten gegenüber zur Sorgfalt nach den Vorschriften des bürgerlichen Vertragsrechts verpflichtet. Insbesondere bei Personenschäden (Schäden an Leben, Körper und Gesundheit) und Behandlungsfehlern haftet der SAPV-Leistungserbringer uneingeschränkt nach den gesetzlichen und vertraglichen Regelungen. Die medizinisch-fachliche Verantwortung obliegt dem betreuenden SAPV-Leistungserbringer und wird durch die Regelungen dieses Vertrages nicht berührt.

(3) Weitergehende Ansprüche der Krankenkassen, insbesondere nach § 116 SGB X, sind durch die vorangehenden Regelungen nicht ausgeschlossen.

## § 21
## Vergütung

(1) Die ärztlichen und pflegerischen bzw. kombinierten Leistungen der SAPV werden versichertenbezogen vergütet.

Die Vergütung sowie zugehörige Leistungsinhalte sind in einer gesonderten Vergütungs- und Leistungsvereinbarung (Anlage 7) geregelt. Mit der Vergütung sind sämtliche Vertragsleistungen und Aufwendungen abgegolten. Eine Abrechnung der Leistungen, die nach den Regelungen dieses Vertrages erbracht und vergütet werden, gegenüber der Kassenärztlichen Vereinigung als vertragsärztliche Leistungen des EBM ist ausgeschlossen (Doppelabrechnung). Im Falle solcher Doppelabrechnungen verringert sich die Vergütung dieses Vertrages entsprechend den gegenüber der Kassenärztlichen Vereinigung abgerechneten Leistungen und es erfolgt eine Rückforderung durch die Krankenkasse gem. Absatz 8.

(2) Die Vergütungs- und Leistungsvereinbarung gilt auch für die Versorgung von Versicherten durch den SAPV-Leistungserbringer, deren Häuslichkeit außerhalb des in § 3 Abs. 1 festgelegten Versorgungsgebietes liegt. Der Versicherte hat einen einmaligen Leistungsanspruch auf SAPV. Der Leistungsfall beginnt mit Genehmigung des ersten Leistungsantrages SAPV und endet mit dem Abschluss der Versorgung des Versicherten. Der Leistungsfall umfasst ggf. auch mehrere Verordnungen und Unterbrechungszeiträume.

(3) Zeiten, in denen die SAPV ruht (Krankenhausaufenthalte) sind nicht auf die Betreuungsdauer anzurechnen.

(4) Für alle Leistungen nach diesem Vertrag dürfen Zuzahlungen weder verlangt noch angenommen werden; ausgenommen sind Zuzahlungen nach SGB V. Für die Einziehung der Zuzahlungen nach dem SGB V finden die diesbezüglichen allgemeinen Regelungen des SGB V Anwendung.

(5) Der Vergütungsanspruch ist von der Erfüllung sämtlicher in diesem Vertrag geregelter sachlicher, personeller, formaler und inhaltlicher Voraussetzungen abhängig. Für Leistungen, die unter Missachtung vertraglicher Bestimmungen erbracht wurden, steht dem SAPV-Leistungserbringer keine Vergütung zu. Dies gilt auch dann, wenn die Leistungen im Übrigen ordnungsgemäß erbracht wurden.

(6) Kosten von Leistungen und Versorgungen, die ohne Vorliegen einer genehmigten Verordnung erbracht werden, können weder gegenüber dem Versicherten noch gegenüber der zuständigen Krankenkasse nach diesem Vertrag abgerechnet werden. § 6 Abs. 5 dieses Vertrages in Verbindung mit § 8 der SAPV-RL bleibt unberührt.

(7) Sofern die Krankenkasse Zahlungen geleistet hat, auf die der SAPV-Leistungserbringer keinen Anspruch hat, ist die Krankenkasse berechtigt, diese Beträge unter Angabe von Gründen zurückzufordern und/oder mit anderen Forderungen des Leistungserbringers zu verrechnen.

MUSTER

(8) Weitergehende Schadensersatzansprüche, insbesondere wegen der Verletzung vertraglicher Pflichten, werden durch diese Regelungen nicht ausgeschlossen.

## § 22
## Leistungsnachweis

(1) Der SAPV-Leistungserbringer hat die nach diesem Vertrag erbrachten Leistungen nachvollziehbar in einem Leistungsnachweis aufzuzeigen (Anlage 8 – Muster). Dieser beinhaltet:

- Angaben des SAPV-Leistungserbringers (Name, Anschrift, Institutionskennzeichen),
- Angaben zum Versicherten (Name, Geburtsdatum, Krankenversichertennummer),
- die an der Vergütungssystematik orientierten Angaben zu
    - Ort, Art und Menge der erbrachten Leistungen sowie
    - Datum und Uhrzeit der Leistungserbringung.

(2) Alle vom SAPV-Leistungserbringer durchgeführten Leistungen sind im Leistungsnachweis bei jedem Hausbesuch von dem/der jeweiligen Palliativarzt bzw. Palliativpflegefachkraft durch Handzeichen, entsprechend der in der Einrichtung hinterlegten Handzeichenliste sowie durch einen Palliativarzt des SAPV-Leistungserbringers im Gesamten gegenzuzeichnen. Quittierungen im Voraus sind unzulässig.

(3) Die Handzeichenliste gemäß Abs. 2 ist getrennt für Vertragsärzte, Krankenhausärzte, Palliativpflegefachkräfte zu führen und bei der Erstabrechnung mit einzureichen. Bei Änderungen wird die aktuelle Liste der Rechnung beigefügt.

(4) Der vollständig ausgefüllte Leistungsnachweis (Muster Anlage 8) stellt die Grundlage für die Abrechnung dar. Bei der Erbringung einer vollständigen Versorgung können weitere Leistungsnachweise genutzt werden (z. B. für die Dokumentation häuslicher Krankenpflege). Die Anforderungen der Abs. 1 bis 3 sind dabei ebenfalls einzuhalten.

## § 23
## Abrechnung der Leistungen

(1) Der SAPV-Leistungserbringer rechnet die erbrachten Leistungen monatlich bei der Krankenkasse ab. Die ärztlichen Verordnungen mit Genehmigungsvermerk sowie der vom Palliativarzt bestätigte Leistungsnachweis (vgl. § 2 Abs. 2) sind der Abrechnung beizufügen.

(2) Der SAPV-Leistungserbringer rechnet die Leistungen nach diesem Vertrag ausschließlich unter einem Institutionskennzeichen (IK) gemäß § 293 SGB V ab. Dieses ist vom SAPV-Leistungserbringer bei der Sammel- und Verteilungsstelle IK der Arbeitsgemeinschaft Institutionskennzeichen (SVI), Alte Heerstraße 111 in 53757 St. Augustin zu beantragen und im Vorfeld des Vertragsabschlusses nachzuweisen. Änderungen der unter dem IK gespeicherten Daten sind unverzüglich der SVI mitzuteilen.

(3) Es sind grundsätzlich Sammelrechnungen zu erstellen, die Folgendes beinhalten:

- das Institutionskennzeichen, Name und Anschrift der abrechnenden Stelle,
- die Krankenversicherungsnummer der Leistungsempfänger,
- die maßgeblichen Diagnosen verschlüsselt nach der internationalen Klassifikation der Krankheiten in der jeweiligen vom BMG herausgegebenen deutschen Fassung (ICD-10-GM) so spezifisch wie möglich und mit Angabe der Zusatzkennzeichen zur Diagnosesicherheit und Seitigkeit (soweit zutreffend)
- Art, Zeitraum und Preis der erbrachten Leistung und
- neben den Zwischenrechnungssummen für jeden Versicherten die Gesamtsumme der Sammelrechnung.

MUSTER

(4) Überträgt der SAPV-Leistungserbringer die Abrechnung einer Abrechnungsstelle, so hat er die Krankenkasse unverzüglich schriftlich zu informieren. Es sind der Beginn und das Ende der Abrechnung sowie der Name der beauftragten Abrechnungsstelle mitzuteilen. Außerdem ist eine Erklärung des SAPV-Leistungserbringers beizufügen, dass die Zahlungen an die beauftragte Abrechnungsstelle mit schuldbefreiender Wirkung erfolgen. Der SAPV-Leistungserbringer ist verpflichtet, selbst dafür zu sorgen, dass mit dem den Krankenkassen mitgeteilten Ende der Abrechnung keine diesen Zeitpunkt überschreitende Inkasso-Vollmacht oder Abtretungserklärung mehr besteht. Das Ende der Abrechnung ist den Krankenkassen schriftlich durch die Abrechnungsstelle oder den sonstigen Forderungsinhaber zu bestätigen.

(5) Sofern die Rechnungslegung einer Abrechnungsstelle übertragen werden soll, ist der Auftragnehmer (Abrechnungsstelle) unter besonderer Berücksichtigung der datenschutzrechtlichen Geeignetheit sowie der von ihm getroffenen technischen und organisatorischen Maßnahmen zum Datenschutz und Datensicherheit, durch den SAPV-Leistungserbringer auszuwählen.

(6) Die Bezahlung der Rechnungen erfolgt innerhalb von 28 Kalendertagen nach Eingang der vollständigen Rechnungsunterlagen bei der zuständigen Krankenkasse oder der von ihr benannten Abrechnungsstelle. Bei Zahlung durch Überweisung gilt die Frist als gewahrt, wenn der Auftrag innerhalb dieser Frist dem Geldinstitut erteilt wurde.

(7) Zahlungen erfolgen unter dem Vorbehalt einer abschließenden sachlichen und rechnerischen Prüfung, spätere Rechnungsberichtigungen bleiben daher vorbehalten. Bei Unvollständigkeit, Differenzen bzw. begründeten Beanstandungen der Abrechnung kann die zuständige Krankenkasse die beanstandete Einzelrechnung an den Leistungserbringer bzw. die Abrechnungsstelle zurückgeben und die Sammelrechnung um die beanstandete Position kürzen. Gleiches gilt, soweit aufgrund von Beanstandungen nur un-

strittige Teilbeträge einer Einzelrechnung gezahlt werden können. Die Zahlungsfrist wird in diesen Fällen gehemmt, sofern innerhalb der Frist nach Abs. 6 eine entsprechende schriftliche Information an den SAPV-Leistungserbringer erfolgt ist.

(8) Für die Form und den Inhalt der Abrechnung gelten die Richtlinien einschließlich der technischen Anlagen zum elektronischen Datenträgeraustausch (DTA) gemäß § 302 Abs. 2 SGB V in der jeweils gültigen Fassung. Die den DTA einführende Krankenkasse informiert den SAPV-Leistungserbringer mindestens 6 Monate vor Einführung über den geplanten Umsetzungstermin. Erfolgt die maschinell verwertbare Datenübermittlung nicht zum festgelegten Zeitpunkt aus Gründen, die der Leistungserbringer zu vertreten hat, hat die zuständige Krankenkasse die mit der Nacherfassung verbundenen Kosten dem SAPV-Leistungserbringer durch eine pauschale Rechnungskürzung bis zur Höhe von 5 % v. H. des Rechnungsbetrages in Rechnung zu stellen. Die Rechnung wird entsprechend gekürzt. Die den DTA einführende Krankenkasse informiert den SAPV-Leistungserbringer rechtzeitig über den Umsetzungstermin.

(9) Über die Regelungen der Abs. 1 bis 8 hinaus können die Krankenkassen einzeln oder teilweise gemeinsam im Einvernehmen mit dem SAPV-Leistungserbringer weitere Regelungen als besondere Anlage zu diesem Vertrag treffen.

MUSTER

## § 24
## Beanstandungen

(1) Abrechnungsbeanstandungen müssen von der zuständigen Krankenkasse innerhalb von 12 Monaten nach Rechnungseingang erhoben werden. § 25 bleibt unberührt. Einsprüche gegen Beanstandungen können innerhalb von einem Monat nach Mitteilung geltend gemacht werden.

(2) Forderungen aus Vertragsleistungen können von Seiten des SAPV-Leistungserbringers nach Ablauf eines Jahres, gerechnet vom Ende des Monats in dem die Leistung erbracht wurde, nicht mehr erhoben werden (Ausschlussfrist).

(3) Bei Unstimmigkeiten in der Abrechnung hat die zuständige Krankenkasse das Recht, den beanstandeten Betrag einzubehalten oder zurückzufordern.

(4) Die zuständige Krankenkasse kann ihre Forderungen gegen den SAPV-Leistungserbringer mit Forderungen des SAPV-Leistungserbringers oder der beauftragten Abrechnungsstelle/Verrechnungsstelle aufgrund von erbrachten SAPV-Leistungen unter Angabe von Art, Höhe und Grund aufrechnen.

(5) Forderungen gegenüber der zuständigen Krankenkasse können ohne deren Zustimmung nicht an Dritte abgetreten werden.

## § 25
## Vertragsverstöße

(1) Ergeben sich für eine Krankenkasse Umstände, die den Verdacht eines Verstoßes gegen Pflichten aus diesem Vertrag begründen, nimmt die Krankenkasse zur einvernehmlichen Klärung Kontakt zum Leistungserbringer auf, um die Problematik nach Möglichkeit einvernehmlich zu klären.

(2) Sofern der Sachverhalt nicht unverzüglich in einem kurzfristig einzuberufenden Gespräch geklärt und der Verdacht ausgeräumt werden kann, hat der SAPV-Leistungserbringer innerhalb von 3 Wochen, nach schriftlicher Übermittlung des Sach-

verhaltes durch die Krankenkasse, die Sachlage aus seiner Sicht schriftlich darzulegen. Sofern die Art und Schwere des Sachverhaltes es erfordern, sind auch kürzere Fristen möglich.

(3) Bestätigt sich im Klärungsverfahren der Vertragsverstoß, können die Krankenkassen vertragsrelevante Maßnahmen ergreifen, sofern nicht vorher mit dem SAPV-Leistungsbringer gemeinsam eine einvernehmliche Lösung herbeigeführt werden konnte. Über den Sachverhalt werden die vertragsschließenden Krankenkassenverbände durch die bearbeitende Krankenkasse informiert.

(4) Äußert sich der SAPV-Leistungserbringer nicht oder nicht fristgemäß, können die Krankenkassen mit sofortiger Wirkung Maßnahmen einleiten. Geeignete Maßnahmen sind insbesondere eine Abmahnung bzw. eine Vergütungskürzung nach billigem Ermessen bis zur Beseitigung des Vertragsverstoßes oder bei schweren, wiederholten oder vorsätzlichen Vertragsverstößen die außerordentliche Kündigung. Bei den in Absatz 5 genannten schweren Vertragsverstößen bedarf es nach der Stellungnahme vor einer Kündigung keiner Abmahnung. Weitergehende Ansprüche (z. B. Schadensersatz und Rückforderungsansprüche wegen unberechtigt gezahlter Vergütungen, vgl. § 21 Abs. 8) der Vertragspartner bleiben davon unberührt.

(5) Schwere Vertragsverstöße sind beispielsweise:
- wiederholte oder vorsätzliche Abrechnung von nicht erbrachten oder nicht vertragsgemäß erbrachten Leistungen,
- Doppelabrechnungen im Sinne § 21 Abs. 1 Satz 4,
- Pflichtverletzungen in deren Folge der Versicherte zu Schaden kommt,
- fortgesetzte Verstöße gegen vertragliche Verpflichtungen oder Mitwirkungspflichten
- fortgesetzt starke Häufung von SAPV-Verordnungen, welche nicht genehmigungsfähig waren.

MUSTER

## § 26
## Vertragsanpassungen

Die Vertragspartner sind an einer konstruktiven Zusammenarbeit interessiert. Sind Vertragsänderungen z. B. aufgrund der Entwicklung der Versorgungsabläufe notwendig, prüfen die Vertragspartner gemeinsam den Handlungs- und Regelungsbedarf. Die Erhebungen gemäß § 16 Abs. 2 d) sind nach Möglichkeit mit einzubeziehen.

## § 27
## In-Kraft-Treten

(1) Der Vertrag tritt am ..... in Kraft.

(2) Die Anlagen 1 bis 9, einschließlich der eingereichten Meldebögen 3 a bis c sowie Übersichten 4 und 5 sind Bestandteil dieses Vertrages.

## § 28
## Kündigung

(1) Dieser Vertrag kann von jedem Vertragspartner mit einer Frist von 12 Monaten ohne Angabe von Gründen zum Ende eines Kalendermonates gekündigt werden (ordentliche Kündigung).

(2) Für den Fall eines schweren Verstoßes nach § 25 Abs. 5 haben die Vertragspartner das Recht, diesen Vertrag mit sofortiger Wirkung zu kündigen (außerordentliche Kündigung). Scheidet einer der im Rubrum genannten SAPV-Leistungserbringer aus, endet zu diesem Zeitpunkt das Vertragsverhältnis, ohne dass es einer Kündigung bedarf. Mit Zustimmung aller Vertragspartner ist eine Fortführung des Vertrages möglich, sofern die Einhaltung der vertraglichen Verpflichtungen gewährleistet wird. Die Folgen einer gesetzlichen Rechtsnachfolge bleiben von dieser Regelung unberührt.

Das Recht zur außerordentlichen Kündigung besteht darüber hinaus auch, wenn der SAPV-Leistungserbringer zahlungsunfähig ist bzw. über sein Vermögen das Insolvenzverfahren eröffnet ist.

(3) Die Anlage 7 (Vergütungs- und Leistungsvereinbarung) kann unabhängig von einer Kündigung des Vertrages ohne Angabe von Gründen gekündigt werden. Die Einzelheiten sind in der Vergütungs- und Leistungsvereinbarung geregelt. Bei einer Kündigung des Vertrages erlischt mit Ablauf der Kündigungsfrist die Vergütungsvereinbarung ohne das dafür eine gesonderte Kündigung erforderlich ist. Dies gilt auch, wenn in der Vergütungsvereinbarung eine anderslautende Kündigungsfrist vereinbart wurde.

(4) Für alle gemäß dieses Vertrages begonnenen und im Zeitpunkt der Vertragsbeendigung noch nicht abgeschlossenen Betreuungsverhältnisse wird die jeweilige Betreuung nach diesem Vertrag auch über das Vertragsende hinaus durchgeführt und vergütet.

MUSTER

(5) Die Kündigung bedarf der Schriftform.

## § 29
## Salvatorische Klausel

Sollten einzelne Bestimmungen dieses Vertrages nichtig sein oder durch gesetzliche Neuregelungen ganz oder teilweise unwirksam werden, so wird hierdurch die Wirksamkeit dieses Vertrages im Übrigen nicht berührt. Tritt ein solcher Fall ein, verständigen sich die Vertragspartner unverzüglich über notwendige Neuregelungen.

Anlagen:

| | |
|---|---|
| 1 | SAPV-RL |
| 2 | Empfehlung |
| 3 | Strukturerhebungsbogen |
| 3 a) | Meldebogen Leistungserbringer |
| 3 b) | Meldebogen Palliativärzte |
| 3 c) | Meldebogen Palliativpflegefachkräfte |
| 4 | Kooperationspartnerübersicht für Palliativpflegefachkräfte/Palliativärzte |
| 5 | Kooperationspartnerübersicht für weitere Kooperationspartner |
| 6 | Datenstatistik |
| 7 | Leistungsbeschreibung und Vergütungsvereinbarung |
| 8 | Leistungsnachweis |
| 9 | Muster Einwilligungserklärung |

_______________
Ort, Datum

_________________________
Träger der Einrichtung

_________________________
AOK PLUS – Die Gesundheitskasse
für Sachsen und Thüringen.

_________________________
BKK-Landesverband Ost,
Landesrepräsentanz Sachsen

_________________________
IKK Sachsen

MUSTER

_________________________
Verband der Ersatzkassen e. V.
Die Leiterin der Landesvertretung
Sachsen

_________________________
Knappschaft, Regionaldirektion Chemnitz

Schleswig-Holstein

# Vertrag über spezialisierte ambulante Palliativversorgung von Schwerstkranken in der Lebensendphase nach §§ 132 d ff SGB V

zwischen

**der AOK Schleswig-Holstein - Die Gesundheitskasse, Kiel,**

**dem IKK-Landesverband Nord**
**(mit Wirkung für die IKK Nord), Schwerin**

**der Landwirtschaftlichen Krankenkasse für Schleswig-Holstein und Hamburg,**

**der Knappschaft,**

**den nachfolgend benannten Ersatzkassen in Schleswig-Holstein**

- **Barmer Ersatzkasse (BARMER), Wuppertal**
- **Techniker Krankenkasse (TK), Hamburg**
- **Deutsche-Angestellten-Krankenkasse (DAK), Hamburg**
- **KKH - Allianz, Hannover**
- **Gmünder ErsatzKasse (GEK), Schwäbisch Gmünd**
- **HEK - Hanseatische Krankenkasse (HEK), Hamburg**
- **Hamburg Münchener Krankenkasse (Hamburg Münchener), Hamburg**
- **hkk, Bremen**

**gemeinsamer Bevollmächtigter mit Abschlussbefugnis:**
**Verband der Ersatzkassen e.V., Siegburg (vdek),**
**vertreten durch den Leiter der Landesvertretung**
**Schleswig-Holstein**

(nachfolgend Krankenkassen genannt)

und

## (Einrichtungen oder Personen, die die SAPV erbringen)

nachfolgend spezialisierter Leistungserbringer genannt

Mustervertrag

## Inhaltsverzeichnis:


§ 1 Zielsetzung

§ 2 Anspruchsvoraussetzungen

§ 3 Anforderungen an die Erkrankungen

§ 4 Versorgungsbedarf

§ 5 Besonders aufwändige Versorgung

§ 6 Verordnung von SAPV

§ 7 Prüfung der Leistungsansprüche durch die Krankenkasse

§ 8 Inhalt und Umfang der Leistungen

§ 9 Verordnung von Arzneimitteln im Rahmen der SAPV

§ 10 Organisatorische Voraussetzungen

§ 11 Personelle Anforderungen

§ 12 Qualitätssicherung/Dokumentation

§ 13 Vertragsregion

§ 14 Vergütung und Rechnungslegung

§ 15 Zuzahlungen

§ 16 Datenschutz

§ 17 Inkrafttreten und Kündigung

§ 18 Salvatorische Klausel

Protokollnotizen


## Anlagen:


| | |
|---|---|
| Anlage 1 | Regionales Versorgungskonzept SAPV |
| Anlage 2 | Nachweis über Sicherstellung einer reibungslosen Versorgung der SAPV-Patienten mit Arznei- Heil- und Hilfsmitteln |
| Anlage 3 | Kooperationsvereinbarungen des Vertragspartners |
| Anlage 4 | Nachweis über die besondere Qualifikation der Ärztinnen und Ärzte |
| Anlage 5 | Nachweis über die besondere Qualifikation der Pflegefachkräfte |
| Anlage 6 | Nachweis über die besondere Qualifikation der Kooperationspartner |
| Anlage 7 | Dokumentation: Erfassungs- und Überleitungsbogen |
| Anlage 8 | Verlaufsdokumentation |
| Anlage 9 | Vertragsregion, in der die SAPV sicherzustellen ist |
| Anlage 10 | Vergütung |
| Anlage 11 | Verordnungsmuster SAPV |
| Anlage 12 | Fragebogen zu SAPV nach § 37b SGB V |
| Anlage 13 | Antrag auf Vergabe einer SAPV-Betriebsstättennummer. |


Mustervertrag

## § 1
## Zielsetzung

(1) Die spezialisierte ambulante Palliativversorgung gemäß § 37 b SGB V (SAPV) dient dem Ziel, die Lebensqualität und die Selbstbestimmung schwerstkranker Menschen zu erhalten, zu fördern und zu verbessern und ihnen ein menschenwürdiges Leben bis zum Tod in ihrer vertrauten häuslichen Umgebung oder in stationären Pflegeeinrichtungen (§ 72 Abs. 1 SGB XI) zu ermöglichen.

Dabei müssen folgende Versorgungsziele erreicht werden:

- Integration des Patienten in ein umfassendes Versorgungsnetz mit dem Ziel, die Lebensqualität in der Lebensendphase zu erhalten.
- Konsequente Schmerz- und Symptomkontrolle
- Fallbezogene Erreichbarkeit 24 Stunden-„Rund-um-die-Uhr“
- Effektive und wirtschaftliche Behandlung
- Einbindung aller Ressourcen in ein Behandlungskonzept unter physischen, psycho-sozialen und spirituellen Gesichtspunkten
- Verbesserung der Schnittstelle der ambulanten und stationären Versorgung
- prioritär ambulante Versorgung im Sinne des Patienten nach dem Prinzip ambulant vor stationär und somit
- Reduzierung von ungeplanten Krankenhausbehandlungen
- Unterstützung und Begleitung der Angehörigen
- Stärkung der Fürsorgekompetenz der Familie
- Koordination der Versorgung des Patienten

Im Vordergrund steht anstelle eines kurativen Ansatzes die medizinisch-pflegerische Zielsetzung, Symptome und Leiden einzelfallgerecht zu lindern.

(2) Den besonderen Belangen von Kindern ist Rechnung zu tragen. Dies wird durch einen separaten Vertrag, der landesweit die Spezialisierte Ambulante Pädiatrische Palliativversorgung (SAPPV) regelt, sichergestellt. Vorhandene, pädiatrisch ausgerichtete Versorgungsstrukturen sind durch Kooperationen entsprechend dort zu integrieren.

(3) Die individuellen seelischen, körperlichen und spirituellen Bedürfnisse und Wünsche der Patientin oder des Patienten sowie die emotionalen Ressourcen und konkreten realen Möglichkeiten ihrer oder seiner vertrauten Personen stehen im Mittelpunkt der Versorgung.

Der Patientenwille, der auch durch Vorsorgevollmachten und Patientenverfügungen zum Ausdruck kommen kann, ist zu beachten.

(4) Die SAPV ergänzt das bestehende Versorgungsangebot, insbesondere das der Vertragsärzte, Krankenhäuser und Pflegedienste.

Sie kann als alleinige Beratungsleistung, Koordination der Versorgung, additiv unterstützende Teilversorgung oder vollständige Versorgung erbracht werden.

Andere Sozialleistungsansprüche bleiben unberührt.

(5) Grundlage des Vertrages sind:

- die „Richtlinie des Gemeinsamen Bundesausschusses zur Verordnung von spezialisierter ambulanter Palliativversorgung“ (Spezialisierte Ambulante Palliativversorgungs-Richtlinie/SAPV-RL) vom 20. Dezember 2007 und

- die „Gemeinsamen Empfehlungen der Spitzenverbände der Krankenkassen nach §

Mustervertrag

132 d Abs. 2 SGB V für die spezialisierte ambulante Palliativversorgung“ vom 23.06.2008

in ihrer jeweils aktuellen Fassung.

## § 2
## Anspruchsvoraussetzungen

Versicherte haben Anspruch auf SAPV, wenn

1. sie an einer nicht heilbaren, fortschreitenden und so weit fortgeschrittenen Erkrankung leiden, dass dadurch ihre Lebenserwartung auf Tage, Wochen oder Monate begrenzt ist (§ 3) und

2. sie unter Berücksichtigung der in § 1 genannten Ziele eine besondere aufwändige Versorgung (§ 5) benötigen, die nach den medizinischen und pflegerischen Erfordernissen auch ambulant oder in stationären Pflegeeinrichtungen (§ 72 SGB XI) erbracht werden. Stationäre Hospize sind keine stationären Pflegeeinrichtungen im Sinne des § 72 SGB XI sondern selbstständige Einrichtungen gemäß § 39a SGB V als integraler Bestandteil einer vernetzten Versorgungsstruktur.

## § 3
## Anforderungen an die Erkrankungen

(1) Eine Erkrankung ist nicht heilbar, wenn nach dem allgemein anerkannten Stand der medizinischen Erkenntnisse Behandlungsmaßnahmen nicht zur Beseitigung dieser Erkrankung führen können.

(2) Sie ist fortschreitend, wenn ihr Verlauf trotz medizinischer Maßnahmen nach dem allgemein anerkannten Stand der medizinischen Erkenntnisse nicht nachhaltig aufgehalten werden kann.

(3) Eine Erkrankung ist weit fortgeschritten, wenn die Verbesserung von Symptomatik und Lebensqualität sowie die psychosoziale Betreuung im Vordergrund der Versorgung stehen und nach begründeter Einschätzung der verordnenden Ärztin oder des verordnenden Arztes die Lebenserwartung auf Tage, Wochen oder Monate gesunken ist.

Insbesondere bei Kindern sind die Voraussetzungen für die SAPV als Krisenintervention auch bei einer länger prognostizierten Lebenserwartung erfüllt.

## § 4
## Versorgungsbedarf

(1) Eine bedarfsgerechte Versorgung mit SAPV ist insbesondere dann gegeben, wenn sie wohnortnah ausgerichtet ist und die Palliativpatienten, die einen besonderen Versorgungsbedarf haben, der durch die allgemeine Palliativversorgung nicht gewährleistet werden kann, ausreichend und zweckmäßig mit der Leistung der SAPV versorgt werden können. Von der Allgemeinen Palliativversorgung (APV) unterscheidet sich die SAPV insbesondere dadurch, dass
- ➢ die nicht heilbare Erkrankung so weit fortgeschritten ist, dass die Lebenserwartung absehbar begrenzt ist und
- ➢ eine besonders aufwändige Versorgung benötigt wird, die auch ambulant in der eigenen Häuslichkeit oder in stationären Pflegeeinrichtungen erbracht werden kann und
- ➢ mindestens eines der in § 4 der SAPV-Richtlinien genannten komplexen Symptomgeschehen vorliegt.

Mustervertrag

Die bedarfsgerechte Versorgung zeigt sich auch darin, dass es mit der Leistung der SAPV mehr Menschen als bisher ermöglicht wird, in ihrer vertrauten häuslichen Umgebung oder in stationären Pflegeeinrichtungen ein menschenwürdiges Leben bis zum Tod zu führen.

(2) Der Versorgungsbedarf kann in einzelnen Regionen unterschiedlich sein Indikatoren für die Bedarfslage können insbesondere sein,

- die regionale Siedlungsstruktur,
- die Altersstruktur,
- epidemiologisch relevante Erkrankungen sowie
- die demografische Entwicklung.

(3) Bei der Entwicklung einer bedarfsgerechten Versorgung sind die bereits bestehenden Strukturen so weit wie möglich einzubeziehen. Auf die bereits tätigen Leistungserbringer ist zurückzugreifen, soweit sie die erforderlichen Anforderungen erfüllen. Verträge sind nur in dem Umfang abzuschließen, wie sie für eine bedarfsgerechte Versorgung i. S. d. SAPV-Bedarfsplanung für das Land Schleswig-Holstein erforderlich sind. Ein Anspruch auf Vertragsabschluss besteht nicht.

## § 5
## Besonders aufwändige Versorgung

Bedarf nach einer besonders aufwändigen Versorgung besteht, soweit die anderweitigen ambulanten Versorgungsformen sowie ggf. die Leistungen des ambulanten Hospizdienstes nicht oder nur unter besonderer Koordination ausreichen würden, um die Ziele nach § 1 zu erreichen.

Anhaltspunkt dafür ist das Vorliegen eines komplexen Symptomgeschehens, dessen Behandlung spezifische palliativmedizinische und / oder palliativpflegerische Kenntnisse und Erfahrungen sowie ein interdisziplinär, insbesondere zwischen Ärzten und Pflegekräften in besonderem Maße abgestimmtes Konzept voraussetzt.

Ein Symptomgeschehen ist in der Regel komplex, wenn mindestens eines der nachstehenden Kriterien erfüllt ist:

- ausgeprägte Schmerzsymptomatik
- ausgeprägte neurologische /psychiatrische / psychische Symptomatik
- ausgeprägte respiratorische / kardiale Symptomatik
- ausgeprägte gastrointestinale Symptomatik
- ausgeprägte ulzerierende / exulzeriende Wunden oder Tumore
- ausgeprägte urogenitale Symptomatik
- sonstiges komplexes Symptomgeschehen

## § 6
## Verordnung von SAPV

(1) SAPV wird von der behandelnden Vertragsärztin oder von dem behandelnden Vertragsarzt verordnet.

Satz 1 gilt für die Behandlung durch die Krankenhausärztin oder den Krankenhausarzt bei einer oder einem von ihr oder ihm ambulant versorgten Patientin oder Patienten entsprechend.

Hält eine Krankenhausärztin oder ein Krankenhausarzt die Entlassung einer Patientin oder eines Patienten für möglich und ist aus ihrer oder seiner Sicht SAPV er-

Mustervertrag

forderlich, kann die Krankenhausärztin oder der Krankenhausarzt die Verordnung ausstellen, längstens jedoch für 7 Tage.

(2) Die ärztliche Verordnung erfolgt auf dem Vordruck, der der Leistungserbringung nach dem jeweiligen aktuellen Versorgungsbedarf (§ 1 Abs. 4) Rechnung trägt und Angaben zur Dauer der Verordnung enthält. Der in Anlage 11 abgebildete Vordruck ist zu verwenden.

(3) Der verordnende Arzt soll vom spezialisierten Leistungserbringer darauf hingewiesen werden, dass die auf der Verordnung angegebenen ICD-Schlüssel/Diagnosen auch in der Abrechnung des Verordners aufgeführt werden.

(4) Der/die Versicherte oder sein gesetzlicher Vertreter hat die Genehmigung der Verordnung schriftlich auf der Rückseite der Verordnung (Muster 63) zu beantragen. Die Unterschrift durch den Vertragsnehmer bzw. dessen Mitarbeiter ist ausgeschlossen. Kann die Unterschrift nicht erbracht werden , weil der Versicherte bzw. sein gesetzlicher Vertreter nicht in der Lage ist zu unterschreiben, bleibt das Unterschriftenfeld auf der Verordnung frei. Der Vertragsnehmer gibt dort oder auf einer Anlage zur Verordnung die Gründe hierfür (z. Bsp. Unzumutbarkeit) an.

## § 7
## Prüfung der Leistungsansprüche durch die Krankenkasse

(1) Inhalt und Umfang der zu erbringenden SAPV-Leistungen ergeben sich aus der gemäß der SAPV-Richtlinie nach § 37b SGB V in Verbindung mit § 92 Abs.1 Satz 2 Nr. 14 SGB V ausgestellten und genehmigten Verordnung. Die Krankenkasse übernimmt bis zu einer Entscheidung über die weitere Leistungserbringung die Kosten für die verordneten und von den Leistungserbringern nach § 132d SGB V erbrachten Leistungen entsprechend der vereinbarten Vergütung nach § 132d SGB V, wenn die Verordnung gemäß § 7 Abs. 2 der SAPV-Richtlinie spätestens an dem dritten der Ausstellung folgenden Arbeitstag der Krankenkasse vorgelegt wird.

(2) Nach Eingang der Verordnung füllt der spezialisierte Leistungserbringer den Fragebogen zur SAPV nach § 37b SGB V (s. Anlage 12) aus und fügt ihn der Falldokumentation bei. Im Falle einer MDK-Begutachtung ist der Fragebogen zur Verfügung zu stellen. Bei Verlängerungsanträgen sind die wesentlichen Änderungen im Symtomgeschehen hinzuzufügen.

(3) Dem zuständigen Medizinischen Dienst der Krankenversicherung (MDK) sind auf Verlangen alle benötigten Auskünfte, die zur Beurteilung der Leistungspflicht der Krankenkassen benötigt werden, zu erteilen.

(4) Der Krankenkasse obliegt die Überprüfung der leistungsrechtlichen Voraussetzungen der SAPV-Bedürftigkeit. Besteht aus Sicht der Krankenkasse in Einzelfällen Anlass, die Notwendigkeit und Dauer der SAPV zu überprüfen, so kann sie vor Beauftragung des MDK unter Angabe des Überprüfungsanlasses eine kurze Stellungnahme des SAPV-Leistungserbringers zur Frage der Versorgungs-/Pflegesituation des Palliativpatienten anfordern.

(5) Bestehen aus Sicht des MDK Bedenken gegen den Fortbestand der leistungsrechtlichen Voraussetzungen bezüglich der (weiteren) Notwendigkeit und Dauer der SAPV, so werden diese gegenüber dem verantwortlichen spezialisierten Leistungserbringer bzw. dem Träger der Einrichtung dargelegt und mit diesem erörtert. In einem solchen Fall werden der spezialisierte Leistungserbringer und die Träger der Einrichtung über das Ergebnis der Überprüfung und die daraus resultierende Entscheidung der Krankenkasse informiert.

Mustervertrag

## § 8
## Inhalt und Umfang der Leistungen

(1) SAPV wird von Leistungserbringern erbracht, mit denen die Krankenkasse zur Sicherung einer bedarfsgerechten Versorgung diesen Vertrag geschlossen hat. Sie wird intermittierend oder durchgängig nach Bedarf als

- Beratungsleistung,
- Koordination der Versorgung,
- additiv unterstützende Teilversorgung,
- vollständige Versorgung

erbracht. Den besonderen Belangen von Kindern und Jugendlichen ist Rechnung zu tragen.

(2) Die Leistungen müssen ausreichend und zweckmäßig sein, dürfen das Maß des Notwendigen nicht überschreiten und sind wirtschaftlich zu erbringen. Soweit die allgemeine Palliativversorgung ausreichend ist, darf SAPV nicht erbracht werden. Dazu prüft der spezialisierte Leistungserbringer im Rahmen des Eingangsassessments innerhalb von drei Arbeitstagen nach Erhalt der Verordnung die Notwendigkeit und Eignung der besonderen Mittel der SAPV. Sofern für eine bedarfsgerechte Versorgung die Mittel der Regelversorgung ausreichen, ist die Krankenkasse hierüber unverzüglich zu informieren. Neben der Krankenkasse sind auch der Versicherte und der Erstverordner über die Ergebnisse des Assessments zu informieren.

(3) Im Falle der Besserung bzw. einer Stabilisierung im Krankheitsverlauf ist der Versorgungsumfang der SAPV so weit wie möglich zu reduzieren und eine Weiterversorgung im Rahmen der allgemeinen Palliativversorgung anzustreben.

(4) Die Sicherstellung der reibungslosen Versorgung mit Arznei, Heil- und Hilfsmitteln in Krisensituationen und am Wochenende ist vom spezialisierten Leistungserbringer zu organisieren. In der Anlage 2 ist dies den Krankenkassen mittels entsprechender Kooperationsvereinbarungen nachzuweisen.

## § 9
## Verordnung von Arznei-, Heil- und Hilfsmitteln im Rahmen der SAPV

(1) Der qualifizierte Palliativarzt des Leistungserbringers verordnet die notwendigen Arznei-, Heil- und Hilfsmittel unter Verwendung der regulären Verordnungsvordrucke (Muster 13, 14, 16 und 18) auf den Namen des Versicherten unter Beachtung des Wirtschaftlichkeitsgebotes nach § 12 SGB V. Die nach den §§ 300, 302 SGB V vereinbarten Abrechnungsverfahren gelten entsprechend.

(2) Der qualifizierte Palliativarzt benötigt für die Abrechnung der extrabudgetäre verordneten Arznei-, Heil- und Hilfsmittel eine gesonderte Betriebsstättennummer (SAPV-BSNR). Diese wird von der Kassenärztlichen Bundesvereinigung (KBV) an den spezialisierten Leistungserbringer (PCT) vergeben. Der Leistungserbringer ist verpflichtet, sich um den Erhalt der Betriebsstättennummer unter Verwendung des in Anlage 13 abgebildeten Antragsformulars zu kümmern. Die AOK Schleswig-Holstein ist über die vergebene SAPV-BSNR zu informieren.

(3) Bei der Verordnung von Arznei-, Heil- und Hilfsmitteln im Rahmen der SAPV wird auf den zu verwendenden Vordrucken - zusätzlich zur SAPV-BSNR - im entsprechenden Feld einheitlich die Pseudo-Arzt-Nummer 333333300 eingetragen. Die SAPV-BSNR und die

Mustervertrag

Pseudo-Arztnummer dürfen nur für Verordnungen im Rahmen der SAPV auf Basis einer gültigen SAPV-Verordnung (Muster 63) verwendet werden.

(4) Auch auf den BtM-Rezepten sind die SAPV-BSNR und die Pseudo-Arztnummer anzugeben. Solange das BtM-Muster noch nicht angepasst ist, ist die SAPV-BSNR in das Feld „Vertragsarztnummer“ und die Pseudo-Arztnummer in das Feld „VK gültig bis“ einzutragen.

(5) Bei der (zeitgleichen) Verordnung von Arzneimitteln einerseits und Hilfsmitteln andererseits sind jeweils getrennte Vordrucke nach Muster 16 zu verwenden.

(6) Die Kassenverbände organisieren, dass Arzneiverordnungsblätter (Muster 16) seitens des Vordruckverlages mit der für das SAPV-Team vergebenen Betriebsstätten-Nummer codiert und nur an vertragsgebundene Ärzte der SAPV ausgegeben werden.

## § 10
## Organisatorische Voraussetzungen

(1) Die Leistungen der SAPV werden durch spezialisierte Leistungserbringer erbracht. Spezialisierte Leistungserbringer sind nach § 11 Abs. 2 qualifizierte Ärztinnen oder Ärzte und nach § 11 Abs. 3 qualifizierte Pflegefachkräfte, die orientiert an den Voraussetzungen der §§ 10 und 11 dieses Vertrages ggf. auch in Kooperation mit anderen Professionen (s. § 11 Abs. 4) eng zusammenarbeiten. Sie erfüllen ergänzend besondere sächliche Voraussetzungen nach Abs. 4 und Abs. 5, die für eine spezialisierte palliativmedizinische und palliativpflegerische Versorgung erforderlich sind. Eine psychosoziale Unterstützung ist in enger Zusammenarbeit z.B. mit ambulanten Hospizdiensten nach § 39a Abs. 2 SGB V, Seelsorge und Sozialarbeit zu gewährleisten.

(2) Die spezialisierten Leistungserbringer sind Teil einer zu koordinierenden multiprofessionell vernetzten Versorgungsstruktur im regionalen Gesundheits- und Sozialsystem. Sie arbeiten mit den an der Versorgung beteiligten Leistungserbringern, wie Hausärzten und ambulanten Hospizdiensten gemäß § 39a Abs. 2 SGB V eng zusammen (integrativer Ansatz). Mit den regelhaft an der Versorgung beteiligten Leistungserbringern sind Kooperationsvereinbarungen als Voraussetzung für Leistungserbringung und Vergütung schriftlich abzuschließen und den Krankenkassen vorzulegen (s. Anlage 3).

(3) Die spezialisierten Leistungserbringer arbeiten nach einem verbindlichen, strukturierten und schriftlich dargelegten Konzept, in dem der inhaltliche und organisatorische Rahmen der Leistungserbringung (inkl. der personellen und sächlichen Ausstattung) sowie die Einbindung in die regionale Versorgungsstruktur beschrieben sind. Das Konzept ist der Krankenkasse vorzulegen. (s. Anlage 1)

(4) Die spezialisierten Leistungserbringer haben als Mindestanforderung an die sächliche Ausstattung Folgendes vorzuhalten bzw. sicherzustellen:

- eine geeignete, aktuell geführte und für die an der Versorgung Beteiligten jederzeit zugängliche Patientendokumentation
- Arzneimittel (inkl. Betäubungsmittel) für die Notfall/Krisenintervention
- Arzt-/Pflegekoffer/Bereitschaftstasche (Berücksichtigung der Kompatibilität der Verbrauchsmaterialien zu Medizinprodukten unterschiedlicher Hersteller, z.B. bei Portsystemen oder Infusionspumpen)
- eine geeignete administrative Infrastruktur, z. B. Büro, Kommunikationstechnik.

(5) Die spezialisierten Leistungserbringer müssen über eine eigenständige Adresse und geeignete Räumlichkeiten für

Mustervertrag

- die Beratung von Patienten und Angehörigen
- Teamsitzungen und Besprechungen
- die Lagerhaltung von eigenen Medikamenten für Notfall- / Krisenintervention und Hilfsmitteln

verfügen. Sofern eine Aufbewahrung von Medikamenten erfolgt, die unter das Betäubungsmittelgesetz (BtM-G) fallen, ist ein BtM-Schrank (§ 15 BtM-G) erforderlich.

.

## § 11
## Personelle Anforderungen

(1) Der spezialisierte Leistungserbringer stellt sicher, dass das für die spezialisierte ambulante Palliativversorgung erforderliche und geeignete Personal zur Verfügung steht. Dabei ist zu berücksichtigen, dass durch den spezialisierten Leistungserbringer eine fallbezogene Erreichbarkeit 24 Stunden-„Rund-um-die-Uhr" für die Patienten, deren Angehörigen und die an der Versorgung Beteiligten sicherzustellen ist. Die ständige Erreichbarkeit mindestens einer Ärztin/eines Arztes und/oder einer Pflegefachkraft ist zu gewährleisten. Die Erreichbarkeit schließt ggf. notwendige Hausbesuche ein.

(2) Die nach § 8 Abs.1 tätigen Ärztinnen und Ärzte verfügen über

- eine anerkannte Zusatzweiterbildung Palliativmedizin nach der aktuell gültigen Weiterbildung der jeweiligen Landesärztekammer (grds. 160 Std. Weiterbildung)

und

- Erfahrung aus der ambulanten palliativen Behandlung von mindestens 75 Palliativpatienten, z. B. in der häuslichen Umgebung und/oder in einem stationären Hospiz, innerhalb der letzten drei Jahre oder aus einer mindestens einjährigen klinischen palliativmedizinischen Tätigkeit in einer Palliativabteilung in einem Krankenhaus innerhalb der letzten drei Jahre.(Nachweis s. Anlage 4)

(3) Die nach § 8 Abs. 1 tätigen Pflegefachkräfte verfügen über:

- die Erlaubnis zur Führung einer der Berufsbezeichnungen Gesundheits- und Krankenpflegerin / Gesundheits- und Krankenpfleger, Gesundheits- und Kinderkrankenpflegerin / Gesundheits- und Kinderkrankenpfleger oder Altenpflegerin / Altenpfleger (dreijährige Ausbildung aufgrund einer landesrechtlichen Regelung) entsprechend den gesetzlichen Bestimmungen in der jeweils gültigen Fassung und

- den Abschluss einer Palliative-Care-Weiterbildungsmaßnahme im Umfang von mindestens 160 Stunden

  und

- Erfahrung durch mindestens eine zweijährige praktische Tätigkeit als Pflegefachkraft in der Betreuung von Palliativpatienten in den letzten drei Jahren; davon mindestens sechsmonatige Mitarbeit in einer spezialisierten Einrichtung der Hospiz- und Palliativversorgung. Dazu zählt auch die in einem Palliativnetz/PCT erbrachte und vergleichbar geleistete Tätigkeit. (Nachweis s. Anlage 5)

(4) Soweit weitere Fachkräfte (z.B. Sozialarbeiterinnen / Sozialarbeiter, Sozialpädago-

Mustervertrag

ginnen oder Sozialpädagogen, Psychologinnen oder Psychologen) vertraglich eingebunden werden, haben diese eine Zusatzweiterbildung Palliative Care für andere Berufsgruppen oder eine mehrjährige Erfahrung in der Palliativversorgung nachzuweisen. (Nachweis s. Anlage 6)

## § 12
## Qualitätssicherung/Dokumentation

(1) Die spezialisierten Leistungserbringer sind verpflichtet, ein internes Qualitätsmanagement durchzuführen. Sie nehmen regelmäßig an palliativmedizinischen/-pflegerischen Fortbildungen teil und führen möglichst halbjährlich multidisziplinäre Qualitätszirkel durch, an denen auch die übrigen in der Versorgung Tätigen teilnehmen. Die spezialisierten Leistungserbringer sollen sich außerdem an Maßnahmen der externen Qualitätssicherung beteiligen.

(2) Ein geeignetes Dokumentationssystem ist sachgerecht und kontinuierlich zu führen und auf Wunsch der Krankenkasse als Konzept vorzulegen. Das Dokumentationssystem muss patientenbezogene Daten und - soweit vorhanden - allgemein anerkannte Indikatoren für eine externe Qualitätssicherung enthalten und eine bundesweite Evaluation ermöglichen. Es soll kompatibel zu den bestehenden Dokumentationssystemen der an der Versorgung beteiligten Leistungserbringer sein.

Zusätzlich finden die im Folgenden dargestellten Dokumentationsinstrumente Anwendung.

- Erfassungs- und Überleitungsbogen (s. Anlage 7) mit Inhalten zu medizinischen, pflegerischen und psychosozialen Gesichtspunkten. Ziel ist es, in kurzer Zeit einen umfangreichen Überblick über die verschiedenen Gesichtspunkte der Versorgung des Schwerstkranken zu erhalten.

- Verlaufsdokumentation (s. Anlage 8) mit standardisierten Inhalten zu Qualität, Intensität und Verlauf von Schmerz und anderen belastendenden Symptomen, sowie Verlaufsdokumentation der medikamentösen Dauertherapien und Bedarfsmedikation (siehe gemeinsames Dokumentationssystem in Zusammenarbeit mit der Diako in Flensburg).

Zusätzlich werden in dieser Patientenakte die Besuche durch den Hausarzt, das Pflegeteam wie auch allen Mitwirkenden im Versorgungsnetz dokumentiert.

(3) Die Teilnahme an Supervision ist zu ermöglichen. Regelmäßige multiprofessionelle Fallbesprechungen sind in überschaubaren Intervallen durchzuführen; die inhaltliche und zeitliche Organisation ist den Beteiligten überlassen.

(4) Die spezialisierten Leistungserbringer erstellen einen jeweils individuellen Behandlungsplan, der mit den übrigen an der Versorgung beteiligten Leistungserbringern abzustimmen ist.

(5) Dem MDK sind, auf Nachfrage der Krankenkassen, alle erforderlichen Unterlagen vorzulegen, um die Struktur- bzw. Prozessqualität des Leistungserbringers bewerten zu können insbesondere um die gesetzlich vorgeschriebenen und vom GKV-Spitzenverband Bund angekündigten Evaluationen durchführen zu können.

## § 13
## Vertragsregion

Der spezialisierte Leistungserbringer verpflichtet sich, die Versorgung in der Vertragsregion (siehe Anlage 9) gemäß § 10 Abs. 1 sicherzustellen. Eine flächendeckende Versor-

Mustervertrag

gung ist anzustreben. Die bedarfsgerechte Versorgung für Schleswig-Holstein ergibt sich aus der in Anlage 9 vorangestellten Übersicht.

## § 14
## Vergütung und Rechnungslegung

(1) Die ärztlichen und pflegerischen bzw. kombinierten Leistungen im Rahmen der SAPV werden als Komplexpauschalen vergütet. Die Höhe der Vergütung richtet sich nach den in Anlage 10 aufgeführten Pauschalen.

(2) Einsätze, die wegen fehlender Mitwirkung des Versicherten nicht erbracht werden können, sind nicht abrechungsfähig.

(3) Mit den Komplexpauschalen nach Anlage 10 sind alle Aufwendungen abgegolten, die im Zusammenhang mit der SAPV-Leistung stehen. Eine zeitgleiche Abrechnung von Gebührenordnungspositionen des Einheitlichen Bewertungsmaßstabes (EBM) über die Kassenärztliche Vereinigung ist daneben ausgeschlossen.

(4) Die Vergütung erfolgt ausschließlich gegenüber dem Leistungserbringer auf Basis eines einheitlich abgestimmten Leistungsnachweises, den die Krankenkassen den spezialisierten Leistungserbringern zur Verfügung stellen.

(5) Die Rechnungsstellung erfolgt über den spezialisierten Leistungserbringer oder über eine von ihm beauftragte Person oder Institution. Die Bezahlung erfolgt innerhalb von 28 Tagen nach Rechnungseingang, soweit Leistungspflicht besteht. Die Frist beginnt mit Eingang der vollständigen Abrechnungsunterlagen gemäß Abs. 6 bei der Krankenkasse bzw. einer von ihr genannten Stelle. Bei Zahlung durch Überweisung gilt die Frist als gewahrt, wenn der Auftrag innerhalb dieser Zeit dem Geldinstitut erteilt wurde. Bei Überschreitung des Zahlungsziels können Verzugszinsen in Höhe von 2 Prozentpunkten über dem jeweils gültigen Basiszinssatz p. a. berechnet werden. Der Ersatz eines weitergehenden Verzugsschadens, insbesondere vorgerichtlicher Anwaltskosten, ist ausgeschlossen; prozessuale Kostenerstattungsansprüche bleiben hiervon unberührt. Zu Unrecht bereits gezahlte Leistungen sind innerhalb eines Monats der Krankenkasse zu erstatten; Sätze 4 und 5 gelten entsprechend.

(6) Die Abrechnung hat folgende Bestandteile:

- Abrechnungsdaten
- Urbelege (Verordnungsblätter, im Original),
- ggf. Leistungszusagen der Krankenkassen im Original,
- Leistungsnachweis im Original
- Gesamtaufstellung der Abrechnung (Gesamtrechnung, ggf. Sammelrechnung)

und auch folgende Angaben zu beinhalten:

- Krankenversicherungsnummer des Palliativpatienten
- Diagnosen des Patienten
- LANR und "SAPV-BSNR" / Institutionskennzeichen des Verordners
- Institutionskennzeichen des spezialisierten Leistungserbringer
- die konkret erbrachte Leistung der SAPV gem. Anlage 10
- Zeitraum der Leistungserbringung

(7) Die Rechnungslegung erfolgt auf der Grundlage der §§ 301 ff SGB V im Wege des elektronischen Datenaustauschs. Sollte die nach S. 1 vorgesehene Übermittlung nicht erfolgen können, so gelten die nach dieser Regelung bestehenden inhaltli-

Mustervertrag

chen Vorgaben auch für die anderen Formen der Rechnungslegung. Die Parteien verpflichten sich in diesem Fall zur Herbeiführung des nach S. 1 dieses Absatzes vorgesehenen Regelfalls.

## § 15
## Zuzahlungen

(1) SAPV wird als Sachleistung zur Verfügung gestellt. Jegliche Zuzahlungen vom Versicherten im Rahmen der Vertragsleistungen dürfen weder gefordert noch angenommen werden; die Zuzahlungspflicht nach § 28 Abs. 4 SGB V (Praxisgebühr) bleibt davon unberührt.

(2) Zuzahlungen dürfen auch in dem Fall nicht verlangt werden, wenn die Verordnung bei der Krankenkasse verspätet eingereicht wird, da Kosten erst ab Eingang der Verordnung bei der Krankenkasse gemäß § 7 dieses Vertrages übernommen werden. (Eingang der Verordnung spätestens an dem dritten der Ausstellung folgenden Arbeitstag)

## § 16
## Datenschutz

(1) Bei der Durchführung und Dokumentation der Behandlung sowie bei der Weitergabe von Verwaltungsdaten und medizinischen Daten bleiben die ärztlichen Schweigepflicht, das Sozialgeheimnis und die datenschutzrechtlichen Vorschriften unberührt und sind von allen Vertragspartnern zu beachten. Der spezialisierte Leistungserbringer und seine Kooperationspartner treffen hierfür die erforderlichen technischen und organisatorischen Maßnahmen.

(2) Die Weitergabe und Nutzung der die Versicherten betreffenden Behandlungsdaten und Befunde bedürfen der schriftlichen Einwilligung der Versicherten.

## § 17
## Inkrafttreten und Kündigung

(1) Die Vereinbarung tritt ab dem xx.xx.2009 in Kraft. Sie kann jeweils zum 30.09. zum Ende des Kalenderjahres gekündigt werden, frühestens jedoch zum 31.12.2009. Die Kündigung kann auch von oder gegenüber jeder einzelnen Krankenkasse/jedem einzelnen Krankenkassenverband ausgesprochen werden.

(2) Patienten, die vor Wirksamwerden der Kündigung in die spezialisierte ambulante Palliativversorgung nach diesem Vertrag aufgenommen worden sind, deren Behandlung zum Zeitpunkt der Beendigung des Vertrages jedoch noch nicht beendet ist, werden nach Maßgabe dieses Vertrages behandelt.

(3) Die außerordentliche Kündigung dieses Vertrages aus wichtigem Grund ist möglich. Ein wichtiger Grund liegt insbesondere dann vor wenn:

- durch eine gesetzliche Änderung, aufgrund von Rechtssprechung oder durch behördliche, insbesondere aufsichtsrechtliche Maßnahmen die Durchführung der angestrebten Vereinbarungen bzw. die Erreichung der angestrebten Ziele für die Vertragspartner rechtlich unmöglich oder den Krankenkassen untersagt wird,

- die Voraussetzungen dieser spezialisierten ambulanten Palliativversorgung aus wesentlichen medizinisch-wissenschaftlichen oder tatsächlichen Gründen entfallen.

Mustervertrag

## § 18
## Salvatorische Klausel

(1) Nebenabreden zu dieser Vereinbarung bestehen nicht. Änderungen und/oder Ergänzungen bedürfen der Schriftform. Dies gilt auch für den Verzicht auf dieses Schriftformerfordernis selbst.

(2) Sollte eine Bestimmung dieses Vertrages unwirksam sein oder werden oder die Vereinbarung Lücken enthalten, so bleibt die Rechtswirksamkeit der übrigen Bestimmungen hiervon unberührt. Die unwirksame oder fehlende Bestimmung wird durch eine solche Bestimmung ersetzt, die dem Sinn und Zweck unwirksamer Bestimmungen in rechtswirksamer Weise und wirtschaftlich am nächsten kommt. Gleiches gilt für etwaige Vertragslücken.

(3) Alle Änderungen dieses Vertrages müssen schriftlich erfolgen und von allen Parteien unterzeichnet werden. Mündliche Abreden sind unwirksam.

(4) Die allgemeinen Grundsätze über die Aufklärung des Versicherten bleiben unberührt.

Mustervertrag

**Vertragsunterschriften:**

Ort, den ______________________________________

Spezialisierter Leistungserbringer

Kiel, den ______________________________________

AOK Schleswig-Holstein - Die Gesundheitskasse -

Kiel, den ______________________________________

Verband der Ersatzkassen e.V. (vdek)
Der Leiter der Landesvertretung Schleswig-Holstein

Kiel, den ______________________________________

Landwirtschaftliche Krankenkassen Schleswig-Holstein und Hamburg

Schwerin, den ______________________________________

IKK-Landesverband Nord

Hamburg, den ______________________________________

Knappschaft, Fachbereich See-Krankenversicherung

Mustervertrag

**Anlage 1**

**Regionales Versorgungskonzept SAPV**

Mustervertrag

**Anlage 2**

**<u>Nachweis über Sicherstellung einer reibungslosen Versorgung der SAPV-Patienten mit Arznei- Heil- und Hilfsmitteln</u>**

Mustervertrag

**Anlage 3**

**Kooperationsvereinbarungen des Vertragspartners**

Mustervertrag

**Anlage 4**

**Nachweis über die besondere Qualifikation der Ärztinnen und Ärzte**

Mustervertrag

**Anlage 5**

**Nachweis über die besondere Qualifikation der Pflegefachkräfte**

Mustervertrag

**Anlage 6**

**Nachweis über die besondere Qualifikation der Kooperationspartner**

Mustervertrag

**Anlage 7**

**Dokumentation: Erfassungs- und Überleitungsbogen**

Mustervertrag

**Anlage 8**

**Verlaufsdokumentation (z.B. die schon für Schleswig-Holstein abgestimmte Dokumentation der 14 PCTs)**

Mustervertrag

**Anlage 9**

**Zusammenfassung vorhandener Palliativ Care Teams zur flächendeckenden Versorgung in Schleswig-Holstein in Vertragsregionen SAPV 1 - 8**

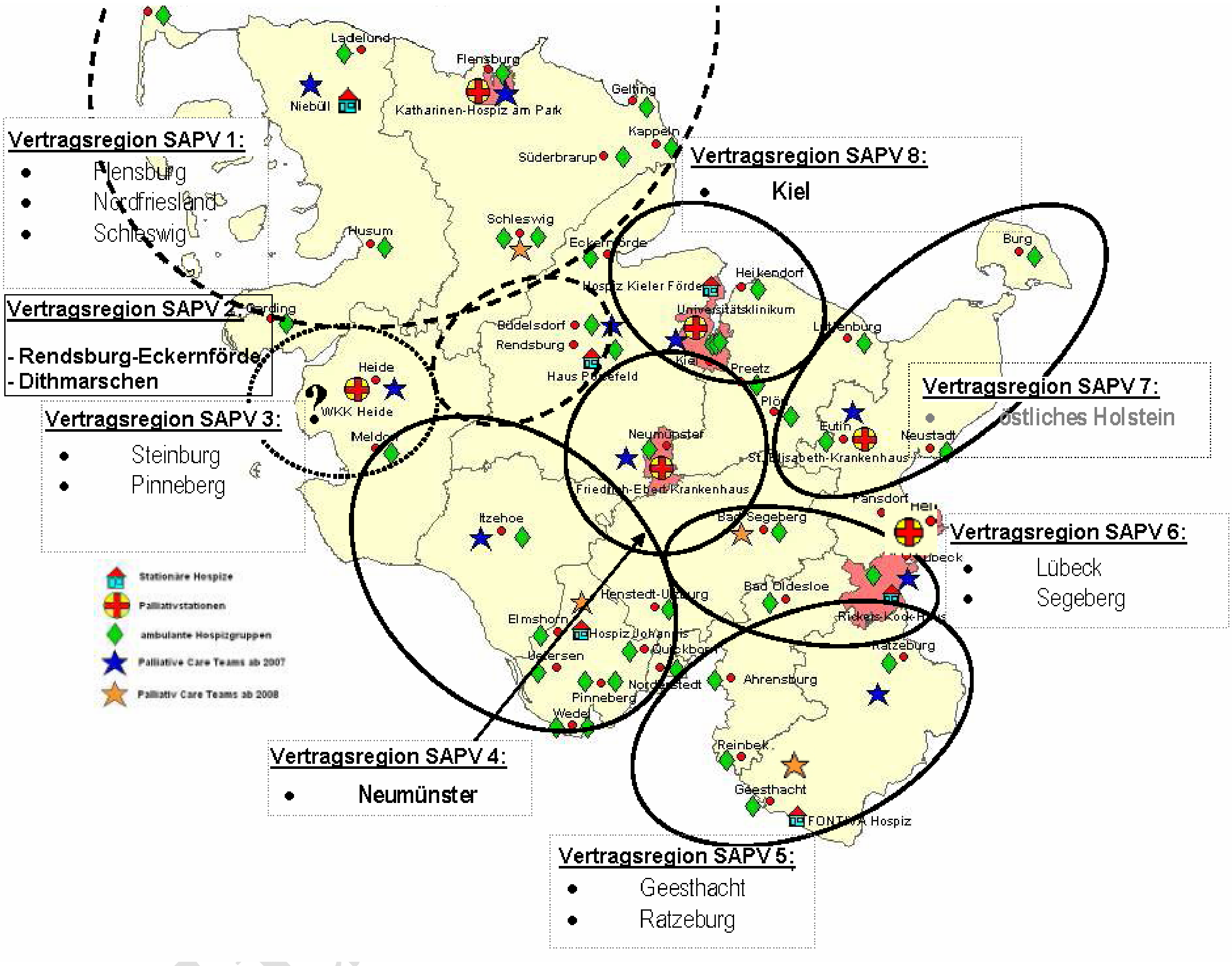


**Vertragsregion, in der die SAPV sicherzustellen ist:**

**Anlage 10**

# Vergütungstabelle SAPV

| Leistung | Komplex-/Tagespauschale in Euro |
|---|---|
| Beratung und Koordination | 200,-€ (Komplexpauschale einmal pro Fall) |
| Additiv unterstützende Teilversorgung | 90,-€ |
| Vollständige Versorgung | 180,-€ |
| Für die ersten sieben Tage der Verordnung, maximal jedoch bis zum Versterben bzw. Ausscheiden der Patientin/des Patienten aus der SAPV, wird die SAPV im verordneten Umfang vergütet. | |

## 1. Beratung und Koordination:

Beratung und anschließende Koordination/Fallmanagement werden in einer Komplexpauschale/Fall zusammengefasst - unabhängig vom erforderlichen Umfang der SAPV. Handelt es sich - entgegen der Verordnung - nicht um SAPV, sondern reichen die Mittel der Regelversorgung und allgemeinen Palliativversorgung aus, koordiniert der spezialisierte Leistungserbringer die Überführung in die Regelversorgung (z.B. Kontaktaufnahme mit den behandelnden Haus- und Fachärzten). Auch in diesen Fällen wird der volle Pauschalbetrag vergütet.

Wurden während eines SAPV-Falles für den Zeitraum von 28 Tagen keine Leistungen erbracht, ist die sich anschließende Verordnung als „Erstverordnung" anzusehen, mit der Konsequenz, dass erneut die Komplexpauschale für „Beratung und Koordination" abgerechnet werden darf.

## 2. Additiv unterstützende Teilversorgung:

Grundsätzlich als Einsatz im Sinne eines Hausbesuches, wenn Beratung und Koordination zur Gewährleistung der SAPV nicht mehr ausreichen. Einzelne Leistungen des in § 5 Abs. 3 der SAPV-Richtlinie des G-BA aufgeführten Leistungskatalogs werden bei Bedarf erbracht.

In erster Linie sind dies:

- Hausbesuche durch das PCT (von Ärzten und/oder Pflegefachkräften ggf. Case-Managern nach Bedarf)
- Ärztliche und/oder pflegerische Leistungen
- Einbeziehung anderer Akteure / Anleitung am Krankenbett
- Krisenvorbeugung / -intervention
- Dokumentation und Evaluation
- der ggf. durch die SAPV bedingte Mehraufwand im Bereich der HKP-Leistungen (§ 37 SGB V).

Die Erbringung und Abrechnung von HKP-Leistungen (§ 37 SGB V) für Grunderkrankungen, die bereits vor der SAPV erbracht wurden, ist nur in Abstimmung mit dem SAPV-Team möglich.

Mustervertrag

## 3. Vollversorgung:

Die vollständige Versorgung beinhaltet die Leistungen der Additiv unterstützenden Teilversorgung. Alle Leistungen des in § 5 Abs. 3 der SAPV-Richtlinie des G-BA aufgeführten Leistungskataloges werden bei Bedarf erbracht, d.h. die SAPV umfasst im Rahmen der Vollversorgung alle Leistungen der ambulanten Krankenbehandlung.

Auch in der Vollversorgung bleiben die Leistungserbringer der Primärversorgung, die bereits vor der SAPV-Verordnung andere, nicht die SAPV auslösende, Erkrankungen und Versorgungsnotwendigkeiten behandelt bzw. abgedeckt haben, für den in diesem Zusammenhang weiter bestehenden Versorgungsbedarf eingebunden. Dies sind in der Regel der Hausarzt und der bereits versorgende Pflegedienst. Die Erbringung von HKP-Leistungen (§ 37 SGB V) hat in Abstimmung mit dem spezialisierten Leistungserbringer zu erfolgen.

Die Vollversorgung umfasst folglich:

- die Leistungen der Additiv unterstützenden Teilversorgung
- ggf. alle in § 5 Abs. 3 der Richtlinie aufgeführten Leistungen
- Dokumentation und Evaluation
- den ggf. durch die SAPV bedingten Mehraufwand im Bereich der HKP-Leistungen (§ 37 SGB V).

Anmerkungen zur Vergütung:

Die Tagespauschalen werden von den Krankenkassen mit befreiender Wirkung jeweils an den regionalen SAPV-Vertragspartner gezahlt. Die Pauschalen für die Teil- und Vollversorgung werden jeweils nur dann gezahlt, wenn der aus der Verordnung hervorgehende Leistungsumfang auch tatsächlich medizinisch/pflegerisch notwendig war und lt. Leistungsnachweis am zu vergütenden Tag erbracht wurde. Mit den Vergütungsbeträgen sind sämtliche im Zusammenhang mit den nach diesem Vertrag erbrachten SAPV-Leistungen sowie sämtliche anfallenden Aufwendungen abgegolten.

Die relative Verteilung der Pauschalen (z.B. ärztliche und pflegerische Einsätze) obliegt dem SAPV-Vertragspartner.

Arznei-, Heil- und Hilfsmittel (auch Verbrauchsmaterialien und sonstige Gerätekosten) sind unter Angabe der gesondert für das PCT/den spezialisierten Leistungserbringer vergebenen Betriebsstättennummer (BSNR) und der Pseudo-Arztnummer: 3333333 00 zu verordnen.

Die zeitgleiche Abrechnung von Gebührenordnungspositionen des Einheitlichen Bewertungsmaßstabes (EBM) über die Kassenärztliche Vereinigung ist neben den SAPV-Pauschalen durch die am Versorgungsnetz teilnehmenden Palliativärzte ausgeschlossen.

Mit dem Ableben der Patientin/des Patienten enden alle Vergütungsansprüche nach dieser Vereinbarung.

**Ansprüche nach dem SGB XI bleiben unberührt.**

Mustervertrag

**Revisionsklausel:**
Zum 30.06.2010 wird diese Vergütungsregelung einer Neubewertung unterzogen. Ziel ist es, gewonnene Erfahrungswerte hinsichtlich Kostendeckung der Pauschalen, aber auch hinsichtlich der Kalkulierbarkeit der unterschiedlichen Leistungsinhalte (Frequenz/Personaleinsatz/Dauer) und damit eines SAPV-Falles (∅ Gesamtkosten/∅ Verweildauer) entsprechend zu berücksichtigen und ggf. eine Anschlussregelung zur Vergütung in Kraft zu setzen.

Mustervertrag

Anlage 11

**Verordnungsmuster SAPV (Version laut Formularkommission)**

| Krankenkasse bzw. Kostenträger | | |
|---|---|---|
| Name, Vorname des Versicherten geb. am | | |
| Kassen-Nr. | Versicherten-Nr. | Status |
| Betriebsstätten-Nr. | Arzt-Nr. | Datum |

## Verordnung spezialisierter ambulanter Palliativversorgung (SAPV) 63

☐ Erst-verordnung ☐ Folge-verordnung

☐ Unfall Unfallfolgen

vom T T M M J J bis T T M M J J

**Verordnungsrelevante Diagnose(n)** (ICD-10; ggf. Organmanifestationen) ____________________

Die Krankheit ist nicht heilbar, sie ist fortschreitend und weit fortgeschritten.

**Komplexes Symptomgeschehen**

☐ ausgeprägte Schmerzsymptomatik

☐ ausgeprägte urogenitale Symptomatik

☐ ausgeprägte respiratorische / kardiale Symptomatik

☐ ausgeprägte gastrointestinale Symptomatik

☐ ausgeprägte ulzerierende / exulzerierende Wunden oder Tumore

☐ ausgeprägte neurologische / psychiatrische / psychische Symptomatik

☐ sonstiges komplexes Symptomgeschehen

**Nähere Beschreibung** des komplexen Symptomgeschehens und des besonderen Versorgungsbedarfs zur Begründung, warum spezialisierte ambulante Palliativversorgung notwendig ist (z. B. therapierefraktäre Schmerzen, Ruhedyspnoe / Erstickungsanfälle, nicht beherrschbares Erbrechen / Durchfälle)

**Aktuelle Medikation** (ggf. einschließlich BtM) ____________________

**Folgende Maßnahmen sind notwendig**

☐ Beratung

☐ a. des behandelnden Arztes

☐ b. der behandelnden Pflegefachkraft

☐ c. des Patienten / der Angehörigen

☐ Koordination der Palliativversorgung

mit folgender inhaltlicher Ausrichtung (Gegenstand, Häufigkeit, evtl. Beratung für Sonstige)

☐ Additiv unterstützende Teilversorgung

☐ Vollständige Versorgung

**Nähere Angaben zu den notwendigen Maßnahmen der SAPV**

Vertragsarztstempel / Unterschrift des Arztes

Für die Erstverordnung ist die Kostenpauschale 40860, für die Folgeverordnung die Kostenpauschale 40862 berechnungsfähig.

Muster 63 (4.2009)

## Antrag des Versicherten

Die spezialisierte ambulante Palliativversorgung wird wie vom behandelnden Arzt verordnet beantragt.

Datenschutzhinweis (§ 67a Abs. 3 SGB X): Damit wir Ihren Antrag auf SAPV-Leistungen bearbeiten können, ist Ihr Mitwirken nach § 60 SGB I erforderlich. Ihre Daten sind im vorliegenden Falle aufgrund § 37b SGB V i. V. m. § 92 Abs. 1 SGB V zu erheben.

Datum T T M M J J

Unterschrift des Versicherten / Vertretungsberechtigten

## Angaben des Leistungserbringers für die SAPV

Die verordnete spezialisierte ambulante Palliativversorgung wird für den Zeitraum vom T T M M J J bis T T M M J J erbracht.

Name des Leistungserbringers / ggf. Stempel

Anschrift (Straße, Hausnummer, PLZ, Wohnort) / ggf. Stempel

Institutionskennzeichen des Leistungserbringers

Ansprechpartner (Name)

Telefonnummer

Fax-Nummer

E-Mail

Datum T T M M J J

Stempel / Unterschrift des SAPV-Leistungserbringers

## Genehmigung der Krankenkasse

Die spezialisierte ambulante Palliativversorgung wird nach den vereinbarten Sätzen

☐ entsprechend der Verordnung übernommen

☐ in folgendem Umfang übernommen

Die Genehmigung der Krankenkasse ist der Abrechnung beizufügen. Endet die Notwendigkeit vor Ablauf des angegebenen Zeitraums, so erlischt damit auch die Kostenverpflichtung.

Sollte die SAPV über den letzten Bewilligungstag hinaus erforderlich sein, so ist **rechtzeitig vor Ablauf** des genehmigten Zeitraums eine erneute Verordnung bei der Krankenkasse einzureichen.

*Auszufüllen, soweit von Seiten der Krankenkasse Bedarf besteht*

Name, Vorname des Versicherten

Straße, Hausnummer, PLZ, Wohnort

Versichertennummer

Geburtsdatum T T M M J J J J

Datum T T M M J J

Stempel / Unterschrift der Krankenkasse

**Anlage 12**

# Fragebogen zu SAPV nach § 37b SGB V



□ **Erstantrag**

□ **Verlängerungsantrag:** SAPV seit:

**Krankenkasse:**

**Name:** **Vorname:** **Geb.-Datum:**

**Adresse:**

**Pflegeversicherung:** □ keine □ beantragt □ St.1 □ St.2 □ St.3 □ Härtefall □ Höherstufung

**Ambulante Versorgung durch:** Name:

□ Hausarzt

□ Palliativarzt

□ Pflegedienst

□ Private Pflegeperson

□ Ambulanter Hospizdienst

**Diagnose mit Erstdiagnose und jetzigem Stadium:**

**Metastasen:**

**Begleiterkrankungen:**

| | | | |
|---|---|---|---|
| **Bisherige Therapien:** | operativ | □ ja □ nein | wann: |
| | Chemo | □ ja □ nein | wann: |
| | Radiatio | □ ja □ nein | wann: |

**Letzte KH-Behandlung:** wann: wo:

## Fragebogen zu SAPV nach § 37b SGB V



**Name:** **Vorname:** **Geb.-Datum:**

**Ausgeprägte Symptomkontrolle, welche?:**

**Aktuelle Medikation:** □ oral □ i.v. □ i.m. □ s.c. □ Infusion □ Pumpe □ Inhalation

- regelmäßig

- bei Bedarf

**Andere erforderliche Behandlungen:**

□ KG □ Lymphdrainage □ Chemotherapie □ Radiatio □ Atemtherapie

**Erforderliche besondere palliativpflegerische Maßnahmen:**

□ Port □ ZVK □ PEG Magensonde □ Sauerstoffgabe □ DK/Zystofix □ Anus praeter

□ Urostoma □ Tracheostoma □ Lagerung

□ Wundbehandlung mit Beschreibung

**Psychosoziale Betreuung:**

- durch:
- Familie/soziales Umfeld:

- Patientenverfügung: □ ja □ nein

**Ort, Datum** **Name, Unterschrift des SAPV PCT** **Name, Unterschrift des feststellenden Arztes**

**Anlage 13**

## Antrag auf Vergabe einer SAPV-spezifischen Betriebsstättennummer für Leistungserbringer nach § 132 d Abs. 1 SGB V

### Leistungserbringer nach § 132 d Abs. 1 SGB V

Name: ........................................................
Ansprechpartner: ........................................................
Anschrift: ........................................................
........................................................
Telefon: ........................................................
Fax: ........................................................
E-Mail: ........................................................
Vertragsbeginn: ........................................................

............................................. ........................................................
Ort, Datum Unterschrift des Leistungserbringers

### Bescheinigung der Antragsberechtigung

Krankenkasse: ........................................................
Name des Ansprechpartners: ........................................................
Anschrift: ........................................................
........................................................
Telefon: ........................................................
Fax: ........................................................
E-Mail: ........................................................

............................................. ........................................................
Ort, Datum Unterschrift der Krankenkasse

.............................................
Stempel der Krankenkasse

Mustervertrag

**Änderungsmeldung zur Vergabe einer SAPV-spezifischen Betriebsstättennummer für Leistungserbringer nach § 132 d Abs. 1 SGB V**

Betriebsstätten-Nummer: ......................................................
Name: ..........................................................................
Anschrift: ..........................................................................
..........................................................................

**Nachfolgende Änderungen ergeben sich ab:** ...........................

Name: ..........................................................................
Ansprechpartner: ..........................................................................
Anschrift: ..........................................................................
..........................................................................
Telefon: ..........................................................................
Fax: ..........................................................................
E-Mail: ..........................................................................

Vertragsende: ..........................................................................

............................................... ...........................................................
Ort, Datum Unterschrift des Leistungserbringers

**Bei Änderungsmeldung durch die Krankenkasse:**

............................................... ...........................................................
Ort, Datum Unterschrift der Krankenkasse

...............................................
Stempel der Krankenkasse

Mustervertrag

Westfalia-Lippe

# Vereinbarung

**zur Umsetzung der ambulanten palliativmedizinischen Versorgung
von unheilbar erkrankten Patienten im häuslichen Umfeld**

**Die AOK Westfalen-Lippe - Die Gesundheitskasse –**

**die BKK-Vertragsarbeitsgemeinschaft Nordrhein-Westfalen (VAG)
- handelnd für die teilnehmenden BKKn -**

**die SIGNAL IDUNA IKK**

**die Landwirtschaftliche Krankenkasse Nordrhein-Westfalen**

**die Knappschaft**

**und die Ersatzkassen**

- Barmer Ersatzkasse
- Techniker Krankenkasse
- Deutsche Angestellten-Krankenkassen
- Kaufmännische Krankenkasse – KKH
- Gmünder ErsatzKasse – GEK
- HEK – Hanseatische Krankenkasse
- hkk

**gemeinsamer Bevollmächtigter mit Abschlussbefugnis
gem. § 212 Abs. 5 S. 5 SGB V:
Verband der Ersatzkassen e. V. (vdek), vertreten durch den Leiter
der Landesvertretung Nordrhein-Westfalen, Geschäftsstelle Westfalen-Lippe,
Dortmund**

**- nachstehend Krankenkassen genannt -**

**sowie die**

**Kassenärztliche Vereinigung Westfalen-Lippe
(KVWL)**

**schließen unter Berücksichtigung des § 132 d SGB V
die nachstehende Vereinbarung**

**Inhaltsverzeichnis:**


| | |
|---|---|
| § 1 | Geltungsbereich, Gegenstand und Zielsetzung |
| § 2 | Zielgruppe der zu versorgenden Patienten |
| § 3 | Teilnahmevoraussetzungen für Haus- und Fachärzte |
| § 4 | Aufgaben der teilnehmenden Haus- und Fachärzte |
| § 5 | Strukturqualität des regionalen PKD |
| § 6 | Aufgaben des regionalen PKD |
| § 7 | Regionaler palliativmedizinischer Qualitätszirkel |
| § 8 | Gemeinsamer Ausschuss |
| § 9 | Verzeichnis der teilnehmenden Ärzte des Regionalen PKD |
| § 10 | Vergütung |
| § 11 | Abrechnung |
| § 12 | Vertragsmaßnahmen |
| § 13 | Datenschutz |
| § 14 | Haftung |
| § 15 | Sonstige Bestimmungen |
| § 16 | Inkrafttreten / Kündigung |


**Anlagen:**


| | |
|---|---|
| Anlage 1 | Teilnahmeerklärung für Hausärzte-/Fachärzte |
| Anlage 2 | Teilnahmeerklärung für qualifizierte Palliativ-Ärzte |
| Anlage 3 | Teilnahmeerklärung des Versicherten |
| Anlage 3 a | Stammdatenblatt |
| Anlage 4 | Palliativ-Dokumentation |
| Anlage 5 | Vergütungsregelung für die teilnehmenden Haus-/Fachärzte |
| Anlage 6 | Vergütungsregelung für die qualifizierten Palliativ-Ärzte |
| Anlage 7 | Feedback-Bogen |
| Anlage 8 | Liste der teilnehmenden Betriebskrankenkassen |


. . .

## Präambel

Unheilbar erkrankte Menschen wünschen oftmals nicht in der teilweise als anonym erlebten Umgebung eines Krankenhauses zu sterben, sondern in eigener häuslicher Umgebung. Das Sterben zuhause stellt heute allerdings eher eine Ausnahme dar. Die Vertragspartner stimmen in der Einschätzung überein, dass die Voraussetzungen für ein Sterben zuhause durch zielgerichtetes Zusammenwirken von Haus- und Fachärzten sowie qualifizierten Pflegediensten verbessert werden können. Mit dieser Vereinbarung setzen die Vertragspartner das nordrhein-westfälische „Rahmenprogramm zur flächendeckenden palliativmedizinischen und palliativpflegerischen Versorgung Nordrhein-Westfalen“ in Westfalen-Lippe um. Die dadurch geschaffenen Leistungsstrukturen beinhalten neben der spezialisierten ambulanten Palliativversorgung im Sinne des § 37 b SGB V auch die allgemeine ambulante Palliativversorgung. Das Angebot palliativmedizinischer Leistungen nach dieser Vereinbarung wird im Sinne des § 132 d SGB V durch bereits bestehende Verträge nach § 132 a SGB V für Palliativpflegedienste sinnvoll und bedarfsgerecht ergänzt. Die im Jahr 2009 gewonnenen Erkenntnisse zur allgemeinen und spezialisierten ambulanten Palliativversorgung werden für die Weiterentwicklung dieser Vereinbarung im 1. Quartal 2010 ausgewertet.

## § 1
## Geltungsbereich, Gegenstand und Zielsetzung

(1) Diese Vereinbarung gilt für Haus-/Fachärzte die nach § 3 Abs. 2 teilnehmen, den regionalen palliativmedizinischen Konsiliardiensten (PKD) nach § 5 Abs. 3 und für Versicherte der Krankenkassen, die ihre Teilnahme nach § 4 Abs. 2 erklärt haben, sowie die vertragsschließenden Krankenkassen. Die teilnehmenden Betriebskrankenkassen sind der Übersicht nach Anlage 8 zu entnehmen.

. . .

(2) Gegenstand der Vereinbarung ist die ambulante Versorgung unheilbar kranker Menschen in der letzten Phase ihres Lebens. Diese Vereinbarung regelt eine kooperative integrative palliativmedizinische Versorgung. Der behandelnde Vertragsarzt übernimmt dabei grundsätzlich die zentrale Rolle und Funktion des koordinierenden Haus-/Facharztes. Sofern eine spezialisierte palliativmedizinische Betreuung geboten ist, steht der PKD nach § 5 hierfür zur Verfügung.

(3) Ziel der Vereinbarung ist es, eine ambulante medizinische Versorgung unheilbar erkrankter Patienten in der letzten Phase ihres Lebens in häuslicher Umgebung zu sichern, ihre Lebensqualität unter Berücksichtigung des Krankheitsstadiums zu verbessern und ihnen ein menschenwürdiges Sterben zu Hause zu ermöglichen, wann immer dies möglich ist und gewünscht wird. Weitere Ziele sind die Schaffung strukturierter Behandlungsabläufe sowie die Vermeidung medizinisch nicht zweckmäßiger Interventionen und nicht notwendiger Krankenhausaufnahmen. Zur häuslichen Umgebung gehört auch die Versorgung in einer stationären Pflegeeinrichtung.

## § 2
## Zielgruppe und Teilnahme der zu versorgenden Patienten

(1) Im Fokus der Palliativ-Versorgung stehen Patientinnen und Patienten mit einer nicht heilbaren progredienten und weit fortgeschrittenen Erkrankung mit begrenzter Lebenserwartung, die an den körperlichen Symptomen dieser Erkrankung und den mit ihr einhergehenden psychosozialen und spirituellen Problemen leiden. Palliativ-Patienten im Sinne dieser Vereinbarung sind Patientinnen und Patienten,

- die an einer unheilbaren Krankheit leiden, die so weit fortgeschritten ist, dass lediglich eine Lebenserwartung von wenigen Tagen, Wochen oder Monaten zu erwarten ist,
- bei denen kurative Behandlungen ausgeschöpft und im Sinne des Patienten nicht mehr angezeigt sind,
- bei denen eine ambulante Palliativ-Versorgung im häuslichen Umfeld möglich und gewünscht ist und
- bei denen eine Krankenhausbehandlung im Sinne des § 39 SGB V nicht erforderlich ist.

. . .

(2) Die Notwendigkeit einer ambulanten Palliativ-Versorgung kann sich insbesondere bei folgenden Krankheitsbildern ergeben:

- Fortgeschrittene Krebserkrankung
- Vollbild der Infektionskrankheit AIDS
- Erkrankung des Nervensystems mit unaufhaltsamen fortschreitenden Lähmungen
- Endzustand einer chronischen Nieren-, Leber-, Herz- oder Lungenerkrankung.

(3) Die Teilnahme des Patienten an dieser Versorgung ist freiwillig. Sie setzt voraus, dass sich der behandelnde Arzt davon überzeugt hat, dass die Voraussetzungen nach Absatz 1 erfüllt sind.

## § 3
## Teilnahmevoraussetzungen für Haus- und Fachärzte

(1) Die Teilnahme an dieser Vereinbarung ist für Haus- und Fachärzte freiwillig. Sie setzt ein besonderes Interesse an der palliativmedizinischen Versorgung sowie Erfahrungen in der Palliativmedizin voraus. Die interessierten Ärzte erhalten Informationen über den regionalen PKD und die Abläufe der palliativmedizinischen Versorgung nach dieser Vereinbarung.
**Protokollnotiz**

(2) Die teilnahmeinteressierten Haus- und Fachärzte erklären ihren Beitritt gegenüber der KVWL schriftlich. Mit der Beitrittserklärung (Anlage 1) akzeptieren die teilnahmeberechtigten Haus- und Fachärzte die Inhalte dieser Vereinbarung. Die KVWL informiert die Verbände der Krankenkassen und den jeweiligen regionalen PKD in Listenform (Arztnummer, Name, Praxisanschrift, Beginn der Teilnahme) über die teilnehmenden Haus- und Fachärzte.

(3) Der Haus-/Facharzt kann seine Teilnahme an dieser Vereinbarung schriftlich mit einer Frist von sechs Wochen zum Quartalsende gegenüber der KVWL kündigen; er informiert darüber parallel den regionalen PKD.

. . .

## § 4
## Aufgaben der teilnehmenden Haus- und Fachärzte

(1) Der teilnehmende Haus- oder Facharzt übernimmt als der vom Palliativ-Patienten gewählte Arzt die Behandlung des Patienten und koordiniert die Einbeziehung weiterer Leistungserbringer im Rahmen des integrierten kooperativen Versorgungskonzepts. Er soll sich dabei an den vorhandenen regionalen Versorgungsstrukturen orientieren. Dem teilnehmenden Haus-/Facharzt nach Satz 1 obliegt die Einschreibung des Palliativ-Patienten im Sinne des § 2 Abs. 1. Soweit der teilnehmende Haus-/Facharzt die Behandlung und Koordination nach Satz 1 nicht selbst übernimmt, schreibt er seinen Patienten ein und überweist ihn in dessen Einvernehmen an den regionalen PKD nach § 5 zur Weiterbehandlung (vgl. § 6 Abs. 1 Satz 3).

(2) Der teilnehmende Haus-/Facharzt lässt die Teilnahme-/Einwilligungserklärung des Patienten nach Anlage 3 von diesem, ggf. seinem in häuslicher Gemeinschaft lebenden Angehörigen oder seinem Betreuer, unterschreiben. Die Teilnahmeerklärung wird gemeinsam mit den Stammdaten nach Anlage 3 a unmittelbar per Telefax an den regionalen PKD gesendet; dieser reicht die Teilnahmeerklärung umgehend an die zuständige Krankenkasse weiter; für die teilnehmenden Betriebskrankenkassen gilt Anlage 8. Das Original der Teilnahmeerklärung wird vom teilnehmenden Haus-/Facharzt archiviert und auf Verlangen der zuständigen Krankenkassen zur Verfügung gestellt. Eine Verordnung ist daneben nicht erforderlich. Die Teilnahme beginnt mit dem Tag der Einschreibung und endet mit dem Eingang der schriftlichen Kündigung bei der Krankenkasse bzw. mit dem Tod des Versicherten. Der teilnehmende Haus-/Facharzt nach Absatz 1 Satz 4 informiert den regionalen PKD zeitgleich mit der Einschreibung (Beginn der Teilnahme des Patienten) über die notwendigen medizinischen Daten (Anlage 3 a) per Telefax.

...

(3) Der teilnehmende Haus-/Facharzt nach Absatz 1 übernimmt folgende Aufgaben:

- Koordination diagnostischer, therapeutischer und pflegerischer Maßnahmen.
- Sofern Maßnahmen der Behandlungspflege von Angehörigen oder Mitbewohnern nicht geleistet werden können, Verordnung von häuslicher Krankenpflege. Sind für palliativpflegerische Maßnahmen besondere Kenntnisse erforderlich, kann palliativpflegerische Krankenpflege verordnet werden; die Krankenkassen teilen der KVWL die zugelassenen Palliativpflegedienste in Westfalen-Lippe mit.
- Einstellung und/oder Beratung zur Schmerztherapie und Symptomkontrolle; bei der Arzneimitteltherapie sind die Empfehlungen der gemeinsamen Arbeitsgruppe Arzneimittelvereinbarung von KVWL und Verbänden der Krankenkassen zu berücksichtigen.
- Verordnung von Medikamentenapplikationen über Pumpensysteme unter besonderer Berücksichtigung des Wirtschaftlichkeitsgebotes.
- Beratung zur künstlichen Ernährung und Flüssigkeitsgabe in der letzten Lebensphase.
- Beurteilung und Einleitung einer palliativmedizinischen Wundbehandlung.
- Koordination bei der Inanspruchnahme unterschiedlicher Leistungserbringer und Versorgungsebenen.
- Grundsätzlich telefonische Rückkopplung mit den qualifizierten Palliativ-Ärzten vor einer stationären Einweisung aufgrund einer palliativmedizinischen Krisensituation.
- Betreuung und Beratung des Palliativpatienten sowie dessen Angehöriger.
- Information des regionalen PKD über den Todestag.
- Ausfüllen eines Feedback-Bogens zur Beurteilung der Tätigkeit des regionalen PKD jeweils zum Ende der Behandlung und dessen Weiterleitung an den PKD.
- Informationen des Palliativpatienten über seine Erreichbarkeit am Wochenende bzw. Feiertag.

Darüber hinaus sollen sich die teilnahmeberechtigten Haus- und Fachärzte weiterhin palliativmedizinisch fortbilden. Dazu sollen sie insbesondere an den regelmäßig angebotenen Qualitätszirkeln nach § 7 mit palliativmedizinischen Inhalten teilnehmen.

. . .

## § 5
## Strukturqualität des regionalen PKD

(1) Für einen Planungsbereich bilden mindestens drei palliativmedizinisch qualifizierte Ärzte (Zusatzweiterbildung „Palliativ-Medizin“ im Umfang von zurzeit 160 Stunden nach der Weiterbildungsordnung einer Landesärztekammer) mit ausreichender Erfahrung in der Behandlung von Palliativpatienten (mindestens 75 Patienten oder einjährige klinische Tätigkeit in einer Palliativabteilung eines Krankenhauses in den letzten 3 Jahren) einen regionalen PKD. Der PKD beschäftigt mindestens eine 3-jährig examinierte Pflegefachkraft mit Abschluss einer Palliativ-Care-Weiterbildung im Umfang von zurzeit 160 Stunden als Koordinationskraft.
**Protokollnotiz**

(2) Anforderungen für die Teilnahme am PKD sind ferner:

- Verpflichtung zur Sicherstellung einer Rund-um-die-Uhr-Erreichbarkeit
- Verpflichtung zur fach- und ggf. sektorübergreifenden bedarfsgerechten Kooperation (vgl. § 5, 6 SAPV Richtlinie)
- Führung der standardisierten Dokumentation (vgl. Anlage 4)
- Leitung/Unterstützung des palliativmedizinisches Qualitätszirkels nach § 7
- Kontinuierliche Fortbildung in Palliativmedizin und Schmerztherapie.

(3) Die teilnahmeinteressierten Palliativmediziner im Sinne des Absatzes 1 beantragen die Teilnahme an diesem Vertrag als PKD mit Nachweis der jeweiligen Qualifikationen und dem Nachweis der Beschäftigung einer palliativpflegerisch qualifizierten Koordinationskraft nach Absatz 1 gegenüber der KVWL mit dem Formular nach Anlage 2 schriftlich. Hiermit akzeptieren die Mitglieder des PKD zugleich die Inhalte dieser Vereinbarung. Die KVWL informiert die Geschäftsstelle der Arbeitsgemeinschaft der Verbände der Krankenkassen in Listenform (Arztnummer, Name, Praxisanschrift, Beginn der Teilnahme) über die Mitglieder des PKD.

. . .

(4) Mitglieder des PKD können ihre Teilnahme an diesem Vertrag mit einer Frist von drei Monaten zum Ende des Kalenderhalbjahres gegenüber der KVWL kündigen. Sofern die Mindestgröße des PKD nach Absatz 1 durch einen Austritt eines Mitgliedes unterschritten wird, ist dieser innerhalb eines halben Jahres fachgerecht nach zu besetzen. Übergangsweise können hierfür auch Palliativärzte mit Weiterbildung aus einem anderen Planungsbereich eingesetzt werden.

## § 6
## Aufgaben des regionalen PKD

(1) Der regionale PKD wird insbesondere auf Anforderung des behandelnden Haus-/ Facharztes oder eines Krankenhausarztes zur Ein-/Überleitung (in) eine(r) ambulante(n) palliativmedizinische(n) Versorgung tätig. Er wird beratend oder in solchen Fällen mitbehandelnd tätig, in denen spezielle palliativmedizinische Kenntnisse für die Symptomkontrolle oder Schmerztherapie erforderlich sind. In Fällen des § 4 SAPV-RL übernimmt der regionale PKD die weitere Behandlung und zeigt diese gem. §§ 7, 8 SAPV-RL der zuständigen Krankenkasse an; die Aufgaben nach § 4 Abs. 3 gelten entsprechend.

(2) Der regionale PKD stellt für die teilnehmenden Haus-/Fachärzte und die Patienten bzw. ihre Angehörigen eine Rund-um-die-Uhr-Erreichbarkeit auch am Wochenende sicher. Die Verfügbarkeit mindestens eines palliativmedizinisch qualifizierten Arztes des PKD auch für Hausbesuche ist zu gewährleisten. Für erforderliche Koordinationsleistungen setzt der PKD eine Koordinationskraft mit palliativpflegerischer Qualifikation ein. Diese koordiniert u.a. die Überleitung in die ambulante palliativmedizinische Betreuung und steuert die fach-/schnittstellengerechte häusliche Versorgung zur Vermeidung unnötiger Rettungsdiensteinsätze und Krankenhausaufnahmen. Sie unterstützt auch eine bedarfsgerechte Einbeziehung der regionalen ambulanten Hospizdienste.

. . .

(3) Zu den Aufgaben der regionalen PKD gehören unter anderem:

- Kontaktaufnahme mit dem teilnehmenden Haus-/Facharzt nach Eingang der Unterlagen gemäß § 4 Abs. 2 Satz 2.

- Auf Wunsch: Beratung des teilnehmenden Haus-/Facharztes bei der

  a) Einstellung zur Schmerztherapie und Symptomkontrolle
  b) Verordnung von Medikamentenapplikationen über Pumpensysteme unter besonderer Berücksichtigung des Wirtschaftlichkeitsgebotes
  c) künstlichen Ernährung und Flüssigkeitsgabe in der letzten Lebensphase
  d) Beurteilung und Einleitung einer palliativen Wundbehandlung
  e) gezielten Einbeziehung eines qualifizierten Palliativpflegedienstes im Sinne des Stufenschemas nach § 4 Abs. 3 2. Spiegelstrich.

- Führen der Dokumentation nach Anlage 4.

- Weitere Aufgaben können auf Wunsch des teilnehmenden Haus-/Facharztes im Einzelfall sein:

  - Palliativmedizinische Mitbehandlung von Patienten bzw. Beratung von Patienten und Angehörigen
  - Unterstützung bei der Aufklärung der Patienten, der Vermittlung „schlechter Nachrichten“ und Information über die Änderung des Therapiezieles
  - Beratung bei der Erstellung einer Patientenverfügung
  - Beratung bei ethischen Konflikten (ethisches Fallgespräch).

- Weiterbehandlung nach Absatz 1 Satz 3.
- Auswertung der Feedbackbögen nach Anlage 7.

. . .

(4) Die Mitglieder des regionalen PKD nehmen an palliativmedizinischen Fortbildungen teil. Sie übernehmen die Leitung des palliativmedizinischen Qualitätszirkels nach § 7.

(5) Bei ausschließlich vom PKD betreuten SAPV-Patienten füllt dieser den Feedbackbogen nach Anlage 7 mit den relevanten Angaben aus. Das Ergebnis aller Feedbackbögen wird den teilnehmenden Haus- und Fachärzten und der KVWL jeweils zum Ende eines Jahres zur Verfügung gestellt, diese informiert die Verbände der Krankenkassen über die vorliegenden Ergebnisse.

## § 7
## Regionaler palliativmedizinischer Qualitätszirkel

(1) Der regionale PKD bildet zusammen mit den teilnehmenden Haus-/Fachärzten einen palliativmedizinischen Qualitätszirkel. Hierin tauschen sich die Teilnehmer regelmäßig über Erfahrungen und Optimierungsmöglichkeiten aus. Der regionale palliativmedizinische Qualitätszirkel wird von Mitgliedern des regionalen PKD geleitet und tritt mindestens einmal im Halbjahr zusammen.

(2) Inhalt und Ziel der multidisziplinären Qualitätszirkel ist insbesondere die sukzessive Entwicklung von Behandlungsleitlinien bezogen auf die relevantesten Erkrankungsbilder von Palliativ-Patienten mit Beschreibung der Schnittstellen und der Aufgabenverteilung der nach dieser Vereinbarung beteiligten Leistungserbringer u. a. für folgende Schwerpunkte:

- Schnittstellen/Anlässe zur Einbeziehung des qualifizierten Palliativ-Ärzte
- Schnittstellen/Anlässe zur Einbeziehung von Pflegediensten mit Weiterbildung in Palliativ-Care
- Systematischer und abgestufter Einsatz von Arznei-, Heil- und Hilfsmitteln
- Kriterien für die Notwendigkeit der Einweisung von Palliativ-Patienten in ein Krankenhaus.

. . .

Ferner ist ein geeignetes Verfahren zur Schmerzmessung und der daraus abzuleitenden medikamentösen Maßnahmen für eine sachgerechte Schmerztherapie abzustimmen. Dieses Verfahren ist so anzulegen, dass auch Angehörige sowie ggf. zu beteiligende Pflegedienste die Schmerzmessung in enger Abstimmung mit dem teilnehmenden Haus- bzw. Facharzt vornehmen können. Die in den Qualitätszirkeln entwickelten Umsetzungsempfehlungen werden der KVWL und den Verbänden der Krankenkassen zur Kenntnis zugeleitet und ggf. im Gemeinsamen Ausschuss nach § 8 beraten.

## § 8
## Gemeinsamer Ausschuss

(1) Zur Weiterentwicklung der Versorgungskonzeption, Klärung von Auslegungsfragen und Beilegung von Meinungsverschiedenheiten wird ein Gemeinsamer Ausschuss gebildet.

(2) Der Gemeinsame Ausschuss wird paritätisch mit Vertretern der KVWL und der Krankenkassen besetzt. Die KVWL kann beratend Vertreter der PKD hinzuziehen. Eine Einberufung erfolgt bei Bedarf.

(3) Die Vertragspartner werden insbesondere die Versorgungen von schwerstbetroffenen SAPV-Patienten eng begleiten. Vorgelegte Kasuistiken werden zeitnah im Gemeinsamen Ausschuss beraten, um die Versorgung ggf. bereits während der Laufzeit dieser Vereinbarung zu optimieren.

(4) Der Gemeinsame Ausschuss berät einstimmig über Maßnahmen nach § 12, die die KVWL umsetzt.

## § 9
## Verzeichnis der teilnehmenden Ärzte und der regionalen PKD

Über die teilnehmenden und ausscheidenden Fach-/Hausärzte nach § 3 sowie der Mitglieder der regionalen PKD nach § 5 führt die KVWL jeweils getrennt ein Verzeichnis. Sie stellt den Verbänden der Krankenkassen - soweit Änderungen eingetreten sind - die aktuelle Fassung dieser Verzeichnisse in regelmäßigen Abständen in elektronischer Form zur Verfügung.

## § 10
## Vergütung

(1) Die ärztlichen Leistungen werden nach Maßgabe der jeweils gültigen regionalen Euro-Gebührenordnung und der jeweils geltenden Abrechnungsregelungen von der KVWL vergütet, soweit im Folgenden keine abweichende Regelung getroffen wird. Die Vergütungen nach Absatz 2 werden von den beteiligten Krankenkassen außerhalb der morbiditätsbedingten Gesamtvergütung nach § 85 SGB V gezahlt.

(2) Für den besonderen Aufwand, der mit der Durchführung dieser Vereinbarung für die teilnehmenden Vertragsärzte entsteht, erhalten

- die teilnehmenden Haus-/Fachärzte eine Vergütung nach Maßgabe der Anlage 5
- der regionale PKD eine Vergütung nach Maßgabe der Anlage 6.

(3) Neben den in den Anlagen 5 und 6 genannten Pauschalen ist eine Abrechnung gleicher Leistungsinhalte nach der regionalen Euro-Gebührenordnung und/oder nach Verträgen zur hausarztzentrierten Versorgung nach § 73 b SGB V nicht zulässig.

## § 11
## Abrechnung

(1) Die teilnehmenden Vertragsärzte beauftragen die KVWL mit der quartalsweisen Abrechnung der erbrachten Leistungen nach § 10 Abs. 2. Diese stellt den Krankenkassen die Vergütung nach § 10 Abs. 2 über KT-Viewer (Ebene 6) gesondert in Rechnung.

(2) Die KVWL rechnet die Pauschalen nach Anlage 6 Ziffer 1 quartalsweise unter Berücksichtigung der Fallzahlstaffeln mit den Krankenkassen ab.

(3) Die KVWL erhebt für die Abrechnung nach § 11 den satzungsgemäßen Verwaltungskostensatz.

. . .

## § 12
## Vertragsmaßnahmen

(1) Verstößt ein teilnehmender Vertragsarzt gegen seine Verpflichtungen nach dieser Vereinbarung löst dies folgende Maßnahmen aus:

- Auf Veranlassung eines Partners dieser Vereinbarung und Beratung im Gemeinsamen Ausschuss nach § 8 die schriftliche Aufforderung an den Vertragsarzt, die vertraglichen Verpflichtungen einzuhalten; hierbei kann auf die Möglichkeit weiterer Maßnahmen (Spiegelstrich zwei bzw. drei) hingewiesen werden.
- Keine extrabudgetäre Vergütung oder nachträgliche Rückforderungen von bereits abgerechneten Vergütungen nach Beratung im Gemeinsamen Ausschuss nach § 8.
- Sofortige Beendigung der Teilnahme nach §§ 3 oder 5, wenn die eingegangenen Verpflichtungen nach dieser Vereinbarung trotz mindestens einer o.a. Aufforderung weiterhin nicht eingehalten werden. Über den Ausschluss entscheidet der Gemeinsame Ausschuss nach § 8.

(2) Disziplinarrechtliche Maßnahmen sowie die Verpflichtung zur Wiedergutmachung eines entstandenen Schadens bleiben unberührt.

## § 13
## Datenschutz

Für die Erhebung, Verarbeitung und Nutzung von personenbezogenen Daten gelten die Regelungen über die Einhaltung der ärztlichen Schweigepflicht nach der Berufsordnung und dem allgemeinen Strafrecht sowie für die verschiedenen Phasen der Verarbeitung personenbezogener Daten und der Datensicherheit die Vorschriften

- des Bundesdatenschutzgesetzes,
- der landesrechtlichen Regelungen zum Datenschutz (Gesundheitsdatenschutzgesetz NW) und
- die Spezialvorschriften für die Datenverarbeitung (Gesetz zum Schutz personenbezogener Daten im Gesundheitswesen)

. . .

in der jeweils gültigen Fassung. Zu den im Zusammenhang mit der Durchführung der integrierten Versorgung erhobenen Daten gibt der Patient vorab auf der vertraglich vereinbarten Teilnahmeerklärung (Anlage 3) seine freiwillige schriftliche Einwilligung zur Übermittlung und Verwendung dieser Behandlungsdaten ausschließlich zu Zwecken der Durchführung der in dieser Vereinbarung geregelten Versorgungsleistungen ab.

## § 14
## Haftung

(1) Die Haftung richtet sich nach den allgemeinen gesetzlichen Bestimmungen.

(2) Aus dieser Vereinbarung können gegen die Krankenkassen keine haftungsrechtlichen Ansprüche hergeleitet werden. Für den Fall, dass eine Krankenkasse dennoch haftungsrechtlich in Anspruch genommen wird, stellen die für die Versorgungsaufgaben nach dieser Vereinbarung jeweils zuständigen Beteiligten für von ihnen zu vertretende haftungsbegründende Umstände die Krankenkassen im Innen- und Außenverhältnis von der Haftung frei.

## § 15
## Sonstige Bestimmungen

(1) Die Vertragspartner werden gemeinsam prüfen, inwieweit die Ziele nach § 1 Abs. 3 erreicht worden sind. Die regionalen PKD stellen hierzu die Dokumentationen nach Anlage 4 und die Auswertung der Anlage 7 zur Verfügung. Ferner bereitet die KVWL die GKV-Abrechnungsdaten je PKD sowie der Hausbesuche auf. Die Krankenkassen werten ergänzend Daten über veranlasste Leistungen und Krankenhausbehandlung aus.

(2) Für teilnehmende Haus-/Fachärzte bzw. Mitglieder des regionalen PKD, die nachweislich aufgrund einer intensiveren Betreuung von Palliativ-Patienten die Toleranzgrenzen der Wirtschaftlichkeitsprüfung überschreiten, stellen die Krankenkassen geeignete Verordnungsdaten zur Verfügung, um ihnen so den Nachweis der sachlichen Notwendigkeit zu erleichtern.

. . .

(3) In begründeten Einzelfällen gewährt der PKD dem MDK WL zu Prüfzwecken Einsicht in die Patientenakten.

(4) Sollte eine Bestimmung dieser Vereinbarung unwirksam sein oder werden oder die Vereinbarung unvollständig sein, so wird die Vereinbarung in ihren übrigen Inhalten davon nicht berührt. Die unwirksame oder fehlende Bestimmung wird durch eine solche Bestimmung ersetzt, die dem Sinn und Zweck unwirksamer Bestimmungen in rechtswirksamer Weise und wirtschaftlich am nächsten kommt. Gleiches gilt für etwaige Vertragslücken.

## § 16
## In-Kraft-Treten/Kündigung

(1) Die Vereinbarung tritt zum 01.04.2009 in Kraft. Sie gilt im Sinne einer Übergangsregelung bis zum 31.03.2010. Die Vertragspartner werden vorher rechtzeitig die Verhandlungen über eine Anschlussvereinbarung aufnehmen. Dabei werden sowohl Preishöhe als auch Preisstruktur – unter Berücksichtigung der dann vorliegenden Erkenntnisse zum Umsetzungsstand der SAPV – Gegenstand der Verhandlungen sein.

(2) Das Recht zur außerordentlichen Kündigung dieser Vereinbarung aus wichtigem Grund bleibt unberührt.

(3) Soweit die Vergütungen nach diesem Vertrag durch Änderung des Einheitlichen Bewertungsmaßstabes berührt werden, verständigen sich die Vertragspartner unverzüglich über eine Anpassung der Vergütungsregelung. Falls eine Anpassung nicht möglich ist, besteht ein außerordentliches Kündigungsrecht für die Vertragspartner.

...

Bochum, Dortmund, Essen, Münster, 30.04.2009

Kassenärztliche Vereinigung
Westfalen-Lippe

. . . . . . . . . . . . . . . . . . . . . . . .
Dr. Thamer
1. Vorsitzender des Vorstandes

AOK Westfalen-Lippe

. . . . . . . . . . . . . . . . . .
Litsch
Vorstandsvorsitzender

BKK-Landesverband NW

. . . . . . . . . . . . . . . . . . . . . . . .
Jörg Hoffmann
Vorsitzender des Vorstandes

BKK-Vertragsarbeitsgemeinschaft NW

. . . . . . . . . . . . . . . . . . . . . . . .
Lutz Kaiser
Vorsitzender des Vertragsausschusses

SIGNAL IDUNA IKK

. . . . . . . . . . . . . . . . . .
Terlohr
Stellv. Vorstandsvorsitzender

Landwirtschaftliche
Krankenkasse NRW

. . . . . . . . . . . . . . . . . .
Döge
Direktor

. . .

Knappschaft

. . . . . . . . . . . . . . . . . .
Stadié
Direktor

Verband der Ersatzkassen e. V. (vdek)

. . . . . . . . . . . . . . . . . .

Der Leiter der Landesvertretung
Nordrhein-Westfalen,
Geschäftsstelle Westfalen-Lippe

**Protokollnotiz zu § 3**

Die Vertragspartner setzen sich dafür ein, dass sich die teilnehmenden Haus- und Fachärzte im Rahmen ihrer Weiterbildung nach § 95 d SGB V auch mit dem Schwerpunkt Palliativmedizin befassen.

**Protokollnotiz zu § 5 Abs. 1 – Übergangsregelung -**

Verfügt ein Mitglied des regionalen PKD mit Weiterbildung in der Palliativmedizin noch nicht über die erforderliche Erfahrung in der Behandlung von Palliativpatienten, kann dieser die Behandlung von 75 Palliativpatienten innerhalb einer Frist von 2 Jahren vom Beginn dieser Vereinbarung an gegenüber der KVWL nachweisen.

Für die Anlaufphase kann der regionale PKD zunächst mit mindestens zwei Ärzten mit palliativmedizinischer Weiterbildung einer Landesärztekammer sowie mindestens zwei weiteren Ärzten mit Erfahrung in der Behandlung von Palliativpatienten und erfolgreich abgeschlossener Teilnahme an einer 40stündigen Kursweiterbildung „Palliativmedizinische Grundversorgung" (Curriculum der DGP und BÄK), die eine Weiterbildung in der Palliativmedizin (Nachweis Anmeldung Ärztekammer) begonnen haben, gebildet werden. Der Abschluss der Weiterbildung bzw. die notwendigen Nachweise über die Erfahrung in der Behandlung von Palliativpatienten bzw. klinische Erfahrungen sind innerhalb von 2 Jahren vom Beginn dieser Vereinbarung an gegenüber der KVWL nachzuweisen.

Kann ein teilnehmender Palliativarzt seine Weiterbildung mangels ausreichender Weiterbildungskapazitäten nicht zeitgerecht abschließen, können die Vertragspartner die Frist einvernehmlich verlängern.

Für die Anlaufphase muss ab 100 Palliativpatienten mindestens eine Halbtagskraft nach § 5 Absatz 1 Satz 2 nachgewiesen werden.

## Anlage 6

**zur Vereinbarung über die ambulante palliativmedizinische Versorgung von unheilbar erkrankten Patienten im häuslichen Umfeld**

## Vergütung der spezialisierten Versorgung durch den palliativmedizinischen Konsiliardienst (PKD)

Der PKD nach § 6 erhält für die nach dieser Vereinbarung zu erfüllenden Aufgaben die Vergütungen nach Ziffern 1, 2, 7 und 9. Der QPA eines PKD nach § 6 erhält für die nach dieser Vereinbarung zu erfüllenden Aufgaben die Vergütungen nach Ziffern 3, 4, 5, 6, 8, 10, 11 und 12.

1. Pauschale für die Koordinierung der Versorgung zur Sicherung einer durchgehend ambulanten Betreuung eingeschriebener Palliativpatienten einschließlich der Unterstützung und Anleitung von Angehörigen, um diesen eine eigenständige Betreuung des Palliativpatienten zu Hause zu ermöglichen.

   Die Pauschale beinhaltet die Bereitstellung des palliativmedizinischen Konsiliardienstes einschließlich der erforderlichen personellen und sachlichen Ressourcen, insbesondere einer Pflegefachkraft mit Palliativ-Care-Weiterbildung sowie

   a) die Ergänzung der palliativmedizinischen Betreuung der Patienten durch Haus-/Fachärzte, insbesondere die von diesen angeforderte palliativmedizinische Beratung und Koordination.

   b) die als Ergänzung der haus-/fachärztlichen Betreuung der Patienten insbesondere vom Hausarzt angeforderte palliativmedizinische Mitbehandlung durch den Palliativarzt des PKD (Teilversorgung) einschließlich angeforderte konsiliarische Besuche.

   c) die vollständige Übernahme der palliativmedizinischen Versorgung von Patienten mit komplexem hausärztlich-ambulant nicht beherrschbarem Symptomgeschehen (SAPV-Vollversorgung), insbesondere bei
      - Schmerzkrisen trotz Einstellung nach WHO III, Atemnotanfällen, therapierefraktärem Erbrechen, Krampfanfall, Darmverschlussproblematik
      - notwendigen intensivmedizinischen Interventionen (Punktionen, Infusionen i.v.) mit dokumentiertem, besonders hohem Koordinationsaufwand und besonderer Bereitschaft zur Vermeidung von Notfallaufnahmen einschließlich Dokumentation nach Anlage 4a sowie
      - erforderlichenfalls tageweiser Bereitstellung einer 24. Std. Präsenz durch eine qualifizierte Pflegekraft.

   d) Persönlicher Austausch zwischen Koordinatorin/Koordinator und Arztpraxis

   SNR 91511 (einmal je Patient) 525,00 EUR

2. Durchführung eines Erstassessments durch eine(n) nach § 5 Abs. 4 qualifizierte Koordinator/in des PKD im Rahmen eines persönlichen Kontaktes im selbstgewählten Umfeld eines eingeschriebenen Palliativpatienten (Besuch) von mindestens 45 bis 60 Minuten Dauer (beim Patienten). Es beinhaltet folgende Leistungen:

   (a) Einschätzung von bestehenden Symptomen und der individuellen palliativen Bedarfe in mindestens fünf Bereichen der Palliativversorgung (z.B Symptomintensität, psychosoziale Belastung,

Selbsthilfefähigkeit, soziale Situation/Einbindung, Alltagskompetenz) mit einem standardisierten Messverfahren beim jeweiligen Patienten (z.B. Basisbogen nach Hope MIDOS)

(b) Einschätzung der Versorgungssituation (z.B. Notwendigkeit von Pflegediensten, ambulante Hospizdienste, Hilfsmittelversorgung)

(c) Einschätzung der psychosozialen Situationen (z.B. Notwendigkeit von Psychoonkologen)

(d) Einschätzung über die notwendige weitere Therapieerwartung (z.B. Maßnahmen bei Verschlechterung usw.)

(e) Einschätzung der spirituellen Haltung

(f) Einschätzung der Angehörigensituation (z.B. Ehegatte, Kinder mit erhöhtem Betreuungsbedarf, Überforderung der Angehörigen usw.) und möglicher ethisch divergierender Haltungen (z.B. Erwartungen zu aktiver Sterbehilfe usw.)

(g) Planung ggf. notwendiger weiterer Routinekontakte (Telefon oder Hausbesuch) in Abstimmung mit dem Patienten und/oder Angehörigen am Ende des Besuchs.

(h) Bericht an den QPA des PKD zur Festlegung eines Versorgungs-/Therapieplans (z.B. über die schmerztherapeutische Einstellung) und ggf. notwendiger weiterer Maßnahmen.

(i) Information des koordinierenden Haus-/Facharztes über das Ergebnis des Erstassessments

Das Erstassessment darf nur von teilnehmenden PKDs abgerechnet werden, die über die nach § 5 Abs. 4 geforderte Stellenzahl hinaus eine zusätzliche, in Vollzeit beschäftigte Koordinationskraft angestellt und gegenüber der KVWL nachgewiesen haben. Die Feststellung hierüber trifft die KVWL entsprechend dem in § 5 Abs. 4 festgelegten Verfahren zum 01.04. eines Kalenderjahres und erteilt den in Frage kommenden PKDs eine Abrechnungsgenehmigung. Die Abrechnungsgenehmigung ist zu widerrufen, sofern die Zahl der Koordinatoren/innen wegen höherer Patientenzahlen nach § 5 Abs. 4 anzupassen ist, die vorgenannte Voraussetzung für die Abrechnung der folgenden SNR vom PKD aber nicht wieder hergestellt wird. Für das 4. Quartal 2017 und das 1. Quartal 2018 trifft die KVWL die Entscheidung auf der Basis der durchschnittlichen Patientenzahlen des Jahres 2016 unmittelbar nach Wirksamwerden dieser Vereinbarung.

SNR 91520 (einmal je Patient) 60,00 EUR

Zuschlag zur SNR 91520 für die Anfahrt zur Erbringung eines Erstassessments in der selbstgewählten Häuslichkeit des eingeschriebenen Palliativpatienten

bei einer Entfernung von über 25 Kilometern gerechnet auf die einfache Strecke - SNR 91521 25,00 EUR

oder

bei einer Entfernung von über 50 Kilometern gerechnet auf die einfache Strecke - SNR 91522 50,00 EUR

Der Zuschlag nach SNR 91521 oder 91522 ist nur einmalig zum Erstassessment nach SNR 91520 abrechnungsfähig (keine doppelte Abrechnungsfähigkeit für Hin- und Rückfahrt). Bei Erbringung des Erstassessments für mehrere Palliativpatienten an einem Ort sind die SNR 91521 oder 91522 nur einmalig ansatzfähig.

Die Ergebnisse des Erstassessments und die Angaben über die Anfahrt zum Patienten (Versichertennummer, Tag, von (Anschrift)…, bis (Anschrift)…, Kilometerangabe des direkten Weges) sind vom PKD zu dokumentieren. Die Dokumentation der Angaben über die Anfahrt ist bei Ansatz der SNR 91521 bzw. SNR 91522 der KVWL auf Anforderung und ggf. zur Weiterleitung an die Krankenkasse vorzulegen. Die Dokumentation der Ergebnisse des Erstassessments ist im Einzelfall auf Anforderung der Krankenkassen dem MDK vorzulegen.

3. Pauschale für einen erforderlichen Hausbesuch mit patientenbezogenem Zeitumfang von bis zu 45 Minuten (daneben sind Gebührenordnungspositionen nach dem EBM nicht abrechnungsfähig) im Sinne der

| | |
|---|---|
| - Ziffer 01410, SNR 91512 | 45,00 EUR |
| - Ziffer 01411, SNR 91513 | 75,00 EUR |
| - Ziffer 01412, SNR 91514 | 90,00 EUR |
| - Ziffer 01415, SNR 91518 | 85,00 EUR |
| - Ziffer 01413, SNR 91519 | 25,00 EUR |

4. Pauschale für einen erforderlichen Hausbesuch mit patientenbezogenem Zeitaufwand von mehr als 45 Minuten (daneben sind Gebührenordnungspositionen nach dem EBM nicht abrechnungsfähig) im Sinne der

| | |
|---|---|
| - Ziffer 01410, SNR 91512S | 65,00 EUR |
| - Ziffer 01411, SNR 91513S | 95,00 EUR |
| - Ziffer 01412, SNR 91514S | 110,00 EUR |
| - Ziffer 01415, SNR 91518S | 105,00 EUR |
| - Ziffer 01413, SNR 91519S | 35,00 EUR |

5. Pauschale für einen erforderlichen Hausbesuch mit patientenbezogenem Zeitaufwand von mehr als 90 Minuten (daneben sind Gebührenordnungspositionen nach dem EBM nicht abrechnungsfähig) im Sinne der

| | |
|---|---|
| - Ziffer 01410, SNR 91512T | 85,00 EUR |
| - Ziffer 01411, SNR 91513T | 115,00 EUR |
| - Ziffer 01412, SNR 91514T | 130,00 EUR |
| - Ziffer 01415, SNR 91518T | 125,00 EUR |
| - Ziffer 01413, SNR 91519T | 55,00 EUR |

6. Zuschlag zu den Hausbesuchen nach Ziffern 3, 4 und 5, wenn vom PKD im Rahmen des Hausbesuches Punktionen (Aszites- bzw. Pleurapunktion, Anlage zentraler Venenkatheter sowie transurethraler und suprapubischer Blasenkatheter) durchgeführt werden

   SNR 91515 — 25,00 EUR

7. Pauschale für eine SAPV-Vollversorgung nach Ziffer 1 c ab dem 4. Tag nach Beginn der Palliativbehandlung (3. auf den Tag der Einschreibung folgender Tag), sofern eine durchgängige ambulante Betreuung gewährleistet ist und durch den PKD (Palliativarzt oder Palliativpflegekraft) persönlich erbracht und nach Anlage 4a angezeigt wird,

   für die ersten 14 Tage der Vollversorgung je Behandlungstag/Patient (gerechnet vom frühestmöglichen Abrechnungsbeginn im Sinne des 1. Halbsatzes)
   SNR 91516 — 120,00 EUR

   vom 15. Tag der Vollversorgung an je Behandlungstag/Patient
   SNR 91517 — 60,00 EUR

   Die Abrechnung der SNR 91516 und 91517 ist insgesamt auf 28 Behandlungstage je Patient begrenzt. Sie kann intermittierend oder durchgängig erbracht werden. Eine intermittierende Leistungserbringung ist nicht gegeben, wenn die Vollversorgung an Einzeltagen mit zeitlichen Unterbrechungen (gesplittet) erbracht wird. Eine ggf. notwendige Verlängerung der Vollversorgung ist mit einer aussagekräftigen Begründung spätestens 1 Woche vor Ablauf der zeitlichen Befristung mit dem Vordruck nach Anlage 4a gegenüber der zuständigen Krankenkasse zu beantragen. Die Krankenkasse teilt dem PKD innerhalb von 2 Wochen Ihre Entscheidung mit. Im Falle einer Ablehnung endet die Abrechnungsfähigkeit der SNR 91517 bei fristgemäßer Beantragung (Satz 4) mit dem Tag des Eingangs des Ablehnungsschreibens der Krankenkasse beim PKD. Die KVWL, Geschäftsbereich Verträge, erhält einen Durchdruck der Entscheidung der Krankenkasse.

   Der Ansatz der SNR setzt die unmittelbare Erbringung palliativmedizinischer-/pflegerischer Leistungen des PKD für den Palliativpatienten am Tag der Abrechnung dieser Symbolnummer sowie einen durchschnittlichen Umfang der Versorgung von 1,5 Stunden beim Patienten voraus. Neben der Vollversorgung ist die Abrechnung eines Hausbesuches je Tag/Patient möglich (eine SNR der Ziffern 3, 4 und 5); eine Verordnung von Pallicare ist ausgeschlossen. Diese Pauschale ist nicht abrechenbar, wenn der Patient innerhalb der letzten 14 Tage vor dem Versterben stationär in ein Krankenhaus aufgenommen wurde. Ausgenommen hiervon sind Krankenhausaufnahmen, die nicht im Zusammenhang mit der Palliativversorgung stehen sowie in Krisensituationen, die vom PKD im Rahmen der Vollversorgung nicht mehr beherrscht werden können.

Zur Vergütung palliativmedizinischer Leistungen für Hospizpatienten gelten folgende Regelungen:

8. Durchführung eines palliativmedizinischen Assessments zu Beginn der Behandlung (Eingangsassessment) zur Klärung des palliativmedizinischen Status (z.B. Schmerzanamnese, Symptomintensität, Lebensqualität, Stimmung, soziale und psychosoziale Situation)

   einmal je Patient, SNR 92010 — 95,00 EUR
   (diese Ziffer ist nicht abrechnungsfähig, wenn der Patient bereits vor Aufnahme in das Hospiz durch denselben Palliativarzt zu Hause palliativmedizinisch versorgt wurde)

9. Koordinations- und Bereitschaftspauschale für die Organisation ambulanter Behandlungsmaßnahmen und eine 24-stündige Rufbereitschaft für Hospizpatienten

   einmal je Patient, SNR 92011 — 175,00 EUR
   (diese Ziffer ist nicht abrechnungsfähig, wenn der Patient bereits vor Aufnahme in das Hospiz durch denselben PKD zu Hause palliativmedizinisch versorgt wurde)

10. Für den Besuch eines Patienten in einem stationären Hospiz wird von den Krankenkassen eine Pauschale (daneben sind Gebührenordnungspositionen nach dem EBM nicht abrechnungsfähig) für folgende EBM-Ziffern gezahlt:

    - Ziffer 01410, SNR 92012 — 45,00 EUR
    - Ziffer 01411, SNR 92013 — 75,00 EUR
    - Ziffer 01412, SNR 92014 — 90,00 EUR

11. Für den Besuch weiterer Patienten in einem stationären Hospiz im Anschluss an die Leistung nach Ziffer 10 wird von den Krankenkassen eine Pauschale (daneben sind Gebührenordnungspositionen nach dem EBM nicht abrechnungsfähig) für folgende EBM-Ziffer gezahlt:

    - Ziffer 01413, SNR 92016 — 25,00 EUR

12. Zuschlag zu den Besuchen, wenn in diesem Rahmen eine Punktion (Aszites- bzw. Pleurapunktion, Anlage zentraler Venenkatheter sowie transurethraler und suprapubischer Blasenkatheter) durchgeführt wird.

    SNR 92015 — 25,00 EUR

Eine Abrechnung von Leistungen der Ziffern 1 bis 7 ist für stationäre Hospizpatienten nicht möglich.

Die Abrechnungsfähigkeit der Ziffern 1 bis 7 ist auf den Betreuungszeitraum nach § 2 Abs. 5 in Verbindung mit § 6 Abs. 2 begrenzt.

Die Vergütungspauschalen nach den Ziffern 1, 2, 7 und 9 werden durch den PKD mit der KVWL ausschließlich über eine von dieser vergebenen Pseudo-Arztnummer elektronisch abgerechnet.

**Für die am Vertrag teilnehmenden PKDs/QPAs ist die Abrechnung der Gebührenordnungspositionen 37300, 37302, 37305, 37306, 37314, 37317, 37318 und 37320 der regionalen Gebührenordnung ausgeschlossen.**
