# Supplementary material for: Structural characteristics and contractual terms of specialist palliative homecare in Germany
Source: BMC Palliat Care. 2023 Oct 31;22:166. doi: 10.1186/s12904-023-01274-6 (PMC10617175; doi:10.1186/s12904-023-01274-6)
Supplement: Supplementary file 2 — Additional file 2: Additional tables (Table 1. Variables assessed in the Wegweiser Hospiz- und Palliativversorgung databank. Variables in italics were used in the latent class analysis. Table 2. Detailed description of institutional affiliations of physicians and nurses in SPHC (n=196)). [file 12904_2023_1274_MOESM2_ESM.docx]

Additional file 2

Table 1: Variables assessed in the *Wegweiser Hospiz- und Palliativversorgung* databank. Variables in italics were used in the latent class analysis.

| **Area** | **Variable** | | **Values** |
| --- | --- | --- | --- |
|  | Year of establishment | | none |
|  | Federal state | | Baden-Württemberg Bavaria Berlin Brandenburg Bremen Hamburg Hesse Mecklenburg-Western Pomerania Lower Saxony North Rhine Rhineland Palatinate Saarland Saxony Saxony Anhalt Schleswig Holstein Thuringia Westphalia |
| Staff members total | Staff members (FTE) | | none |
|  | Staff members (number) | | none |
|  | Volunteers (number) | | none |
| Physician staff members | Physicians (FTE) | | none |
|  | Physicians (number) | | none |
|  | Physicians with professional training in palliative medicine | | none |
|  | Physicians with 100% activity in palliative care | | none |
|  | Physicians with 51-99% activity in palliative care | | none |
|  | Physicians with 1-49% activity in palliative care | | none |
|  | *Physicians employed by SPHC team* | | none |
|  | *Physicians employed by hospital* | | none |
|  | *Physicians from private practices* | | none |
|  | *Physicians from other structures* | | none |
| Nurse staff members | Nurses (FTE) | | none |
|  | *Nurses (number)^1^* | | none |
|  | Nurses with professional training in palliative care | | none |
|  | Nurses with 100% activity in palliative care | | none |
|  | Nurses with 51-99% activity in palliative care | | none |
|  | Nurses with 1-49% activity in palliative care | | none |
|  | *Nurses employed by SPHC team* | | none |
|  | *Nurses employed by hospital* | | none |
|  | *Nurses from nursing services* | | none |
|  | *Nurses from other structures* | | none |
| Other professions | *Psychologist^2^* | | none |
|  | *Religious worker^2^* | | none |
|  | *Social worker^2^* | | none |
|  | Other staff members | | none |
|  | Specification of "other" | | none |
| Organisational management | Physician | | yes/no |
|  | Nurse | | yes/no |
|  | Other staff | | yes/no |
|  | Specification of "other" | | none |
| Medical management | General physician | | yes/no |
|  | Hospital physician | | yes/no |
|  | Hospital physician specialisation | | none |
|  | Medical specialist | | yes/no |
|  |  | Specialisation | none |
|  | Specialised palliative homecare physician | | yes/no |
|  |  | Specialisation | none |
| Organisation of care | *Organisation of coordination and patient care^3^* | | Centralised coordination and patient care Centralised coordination, decentralised patient care Decentralised coordination and patient care Physicians and nurses work separately Other |
|  | Specification of "other" | | none |
| 24-hour service | Physician | | yes/no |
|  | Nurse | | yes/no |
|  | Social worker | | yes/no |
|  | Other professions | | yes/no |
| Assessment by | Physician | | yes/no |
|  | Nurse | | yes/no |
|  | Social worker | | yes/no |
|  | Other | | none |
| Patient data in 2016 | Number of patients in 2016 | | none |
|  | Number of those deceased | | none |
|  | w/ oncological diseases | | none |
|  | Minimum length of stay | | none |
|  | Mean length of stay | | none |
|  | Maximum length of stay | | none |
|  | Median length of stay | | none |
|  | Average distance to patient (kilometers) | | none |
|  | Average driving time to patient (minutes) | | none |
| Cooperation with | SPHC team | | yes/no |
|  | Inpatient hospice | | yes/no |
|  | Hospital palliative care support team | | yes/no |
|  | Palliative care unit | | yes/no |
|  | Tumor center | | yes/no |
|  | Volunteer hospice service | | yes/no |
|  | Hospital | | yes/no |
|  | Nursing home | | yes/no |
|  | General/specialist practice | | yes/no |
|  | Mobile nursing service | | yes/no |
|  | Specialist mobile nursing service | | yes/no |
|  | Psychologist/psychotherapist | | yes/no |
|  | Social worker | | yes/no |
|  | Pharmacy | | yes/no |
|  | Physiotherapist | | yes/no |
|  | Other | | yes/no |
| Cooperation contract with | SPHC team | | yes/no |
|  | Inpatient hospice | | yes/no |
|  | Hospital palliative care support team | | yes/no |
|  | Palliative care unit | | yes/no |
|  | Tumor center | | yes/no |
|  | Volunteer hospice service | | yes/no |
|  | Hospital | | yes/no |
|  | Nursing home | | yes/no |
|  | General/specialist practice | | yes/no |
|  | Mobile nursing service | | yes/no |
|  | Specialist mobile nursing service | | yes/no |
|  | Psychologist/psychotherapist | | yes/no |
|  | Social worker | | yes/no |
|  | Pharmacy | | yes/no |
|  | Physiotherapist | | yes/no |
|  | Other | | yes/no |
| ^1^ For LCA, we performed a median split.  ^2^ Information on team members from the psychosocial professions was aggregated to “psychosocial profession in team” for LCA.  ^3^ For LCA, we only used the categories “centralised coordination and care” and “centralised coordination, decentralised care”. | | | |

Table 2: Detailed description of institutional affiliations of physicians and nurses in SPHC (n=196).

| **Item** | **Categories** | **Physicians** | | **Nurses** | |
| --- | --- | --- | --- | --- | --- |
|  |  | Number of teams | % | Number of teams | % |
| Physicians/nurses institutional affiliations in team | Exclusively from hospital | 11 | 5.6 | 16 | 8.2 |
|  | Exclusively from private practice/nursing care service | 41 | 20.9 | 31 | 15.8 |
|  | Exclusively from team | 25 | 12.8 | 78 | 39.8 |
|  | Exclusively from other structures | 3 | 1.5 | 5 | 2.6 |
|  | Hospital and private practice/nursing care service | 35 | 17.9 | 1 | 0.5 |
|  | Hospital and team | 14 | 7.1 | 14 | 7.1 |
|  | Private practice/nursing care service and team | 1 | 0.5 | 18 | 9.2 |
|  | Private practice/nursing care service and other structures | 14 | 7.1 | 5 | 2.6 |
|  | Team and other structures | 12 | 6.1 | 3 | 1.5 |
|  | Hospital, private practice/nursing care service, SPHC | 2 | 1.0 | 4 | 2.0 |
|  | Hospital, private practice/nursing care service, other structures | 12 | 6.1 | 3 | 1.5 |
|  | Private practice/nursing care service, team and other structures | 6 | 3.1 | 4 | 2.0 |
|  | All | 5 | 2.6 | 1 | 0.5 |
|  | Missing | 7 | 3.6 | 13 | 6.6 |
