# Supplementary material for: Structural characteristics and contractual terms of specialist palliative homecare in Germany
Source: BMC Palliat Care. 2023 Oct 31;22:166. doi: 10.1186/s12904-023-01274-6 (PMC10617175; doi:10.1186/s12904-023-01274-6)
Supplement: Supplementary file 3 — Additional file 3. SAVOIR LCA statistical codes. [file 12904_2023_1274_MOESM3_ESM.pdf]

Additional file 3: SAVOIR LCA statistical codes

```r
setwd("path") # add the path of the data

library(Hmisc)
library(poLCA)

#Read data

d<-spss.get("20190208_SAVOIR-Strukturdatensatz.sav", use.value.labels = F)


d.sub<-d[,c("korr.Ärzte.Klinik",
            "korr.Ärzte.Praxis",
            "korr.Ärzte.SAPV",
            "korr.Ärzte.andere.Strukturen",
            "Klass.Anzahl.PK",
            "korr.GKP.Klinik",
            "korr.GKP.Pflegedienste",
            "korr.GKP.SAPV",
            "korr.GKP.andere.strukturen",
            "korr.weitere.psycho.ja",
            "Kat.Orga.Struktur"
)]

d.sub<-d.sub+1

f <- as.matrix(d.sub) ~ 1

#LCA for different classes

lc1<-poLCA(f, d.sub ,nclass = 1, nrep=10)

lc2<-poLCA(f, d.sub ,nclass = 2, nrep=10)

lc3<-poLCA(f, d.sub ,nclass = 3, graphs=T, nrep=10)

lc4<-poLCA(f, d.sub, nclass=4,graphs = T, nrep=10, maxiter=10000)

lc5<-poLCA(f, d.sub, nclass=5, nrep=10)
```
